# Catalyst-Controlled Divergent Annulation Reactions of Allenoates and Fully Substituted 1,3-Indandione Derived Electron-Deficient Alkenes

**DOI:** 10.1021/acs.joc.6c00628

**Published:** 2026-06-24

**Authors:** Wu-Dong Yu, I-Ting Chen, Ting-Hung Chu, Po-Heng Lin, Jeng-Liang Han

**Affiliations:** Department of Chemistry, 34916 National Chung Hsing University, Taichung 402202, Taiwan

## Abstract

Two catalyst-controlled, regiodivergent annulation protocols for allenoates with fully substituted 1,3-indandione derived electron-deficient alkenes have been developed, furnishing a series of spiroindane-1,3-diones and 3,4-dihydro-2*H*-pyran derivatives in high yields. Plausible mechanistic pathways for both annulation reactions are proposed: spiroindane-1,3-diones are obtained via a [3 + 2] annulation, while 3,4-dihydro-2*H*-pyran derivatives arise from a [4 + 2] annulation.

## Graphical abstract

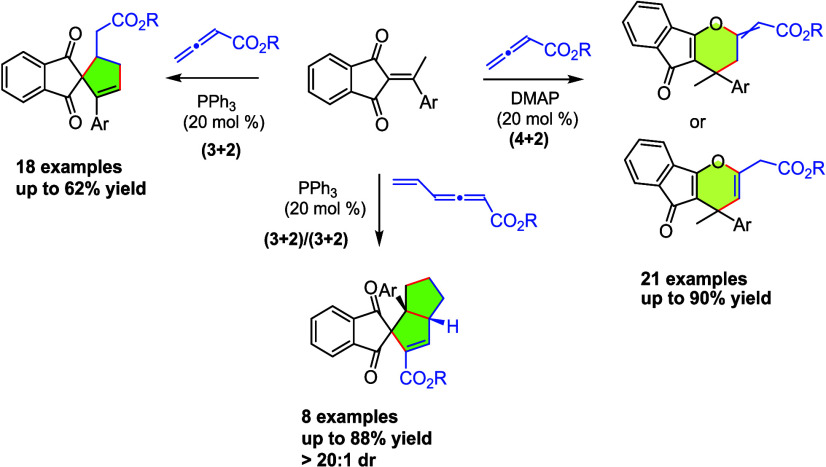

## Introduction

The spiroindane-1,3-diones are widely found in natural products, pharmaceutical molecules, and bioactive compounds (Figure 1). For example, fredericamycin A is an antitumor compound with antibiotic properties.^1^ The highly rigid spirocyclic structure allows it to interact with specific enzymatic targets. Biphenyl-based spirocyclic ketone **1** are recognized as new anticancer agents.^2^ In addition, pyrans are useful building blocks that have a wide range of remarkable biological activities.^3^ Like racemate warfarin^4^ and annulin B,^5^ they show good anticoagulant medication and indoleamine 2,3-dioxygenase (IDO) inhibition activity, respectively. In recent decades, an increasing number of chemists have devoted efforts to the synthesis of such molecules. Among various reported methodologies for synthesizing the spiroindane-1,3-dione skeletons,^6^ organocatalytic domino reactions using readily accessible 2-arylidene Indane-1,3-diones have been demonstrated as a powerful strategy.^7^ Moreover, organocatalytic [4 + 2] annulations of allenoates with activated olefins will be most efficient way for the synthesis of pyran structural motif.^8,9^

**Figure 1:**
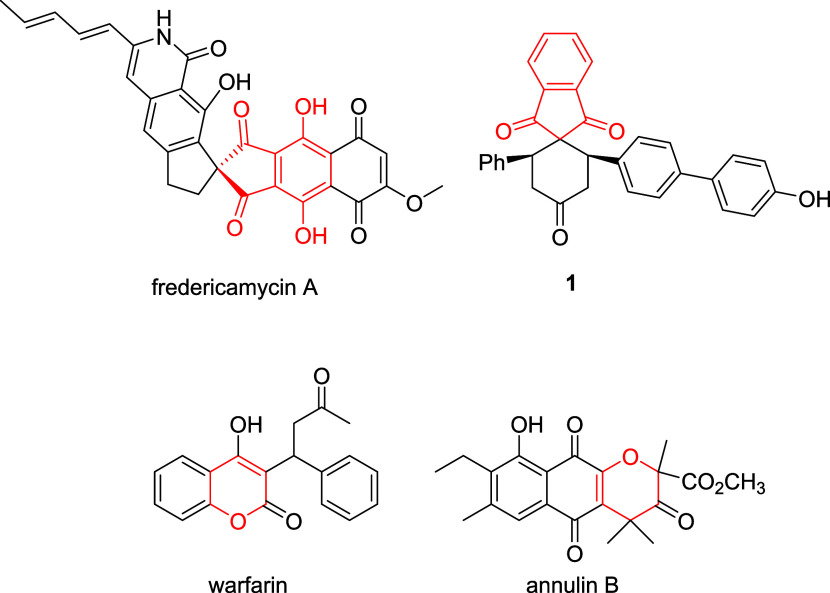
Examples of pharmaceuticals and bioactive molecules containing spiroindane-1,3-dione and pyran scaffolds.

Although significant progress has been made in the synthesis of these compounds, their practical applications are still limited by factors such as the difficulty in preparing starting materials, harsh reaction conditions, and the high cost of catalysts. Therefore, developing efficient synthetic methods for spirocyclic compounds and pyran derivatives using readily available catalysts and substrates under mild conditions remains a significant challenge.

Allenes and allenoates are easily accessible building blocks and exhibit high activity toward electrophiles, especially the Lewis base catalyzed annulation reaction with allenoates have emerged as powerful synthetic tools in the rapid construction of a variety of complex structures under mild conditions.^10^ Recently, phosphine- ^11,12^ and nitrogen- ^8,9,13^ containing Lewis base catalyzed annulation reactions of allenoates has been largely demonstrated by the preparation of biologically active natural products and pharmaceutically interesting substances. It was found that the nature of these Lewis base catalysts has a pronounced effect on these reactions, which often allows for orthogonal reactivities and connectivities when utilizing different catalyst principles,^14^ thus allowing for diversity-oriented approaches as well.^15^ For example, the annulation reaction of allenoates **2** with 2-arylidene-1,3-indanediones **3** generated different annulation products when different Lewis base catalysts were used (Scheme 1A). In the presence of DABCO, this reaction yields the formal (4 + 2) cycloaddition products (*E*)-**4** and **5**, whereby the location of the double bond depends on the reaction conditions.^9c^ In contrast, the use of PPh_3_ gives the regioisomeric (3 + 2) cycloaddition products **6**.^7d,16^ However, the examples of Lewis base-dependent divergent annulation reaction of allenoates **2** with tetra-substituted Michael acceptors are rare. In 2011, Shi and co-workers reported a phosphine catalyzed [3 + 2] cycloaddition of isatin-derived α,β-unsaturated diesters with allenoates **2**, while the amine catalyst gave [4 + 2] annulation adducts selectively (Scheme 1B).^17^

**Scheme 1:**
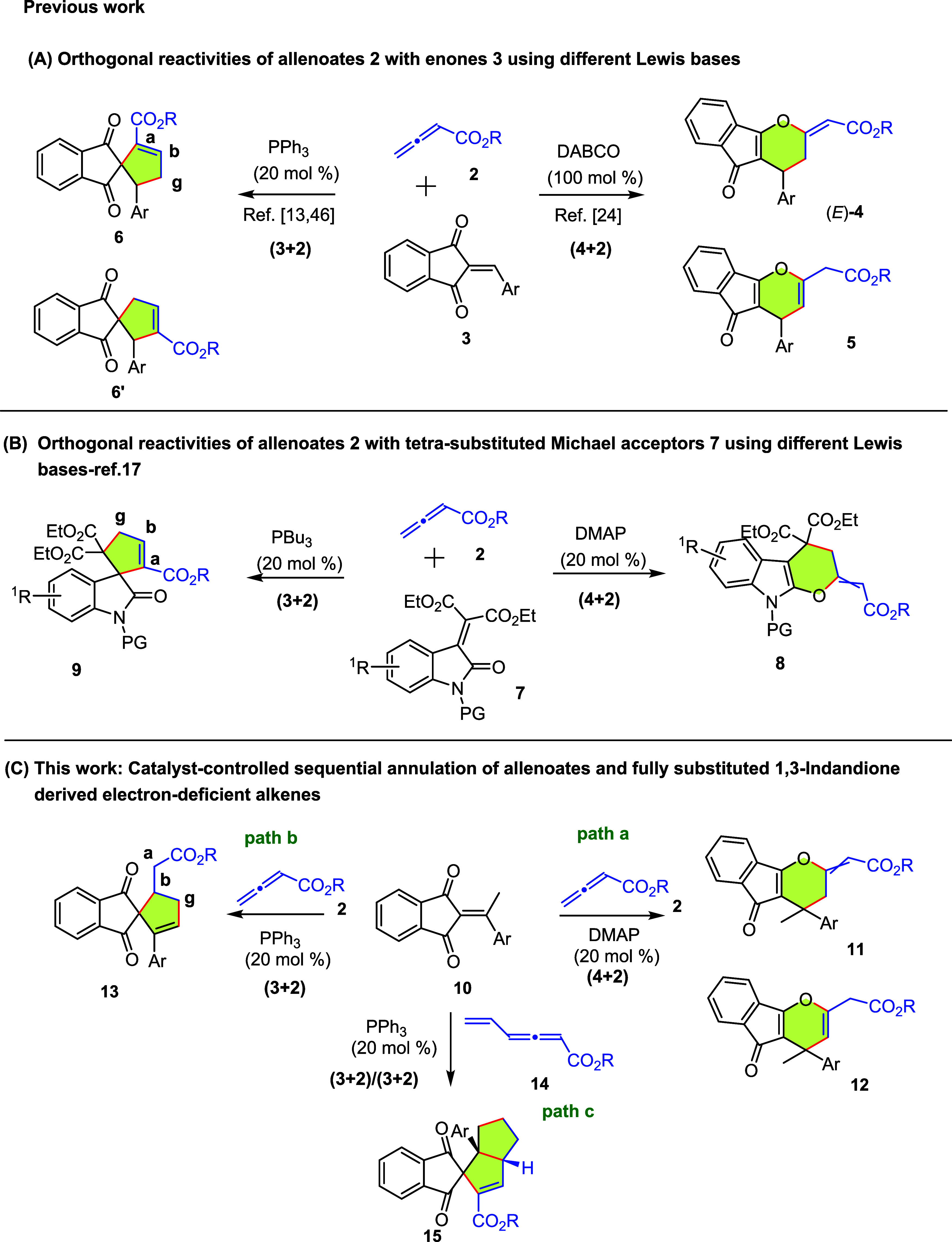
Orthogonal Reactivities of Allenoates **2** with Enones **3** Using Different Lewis Bases (A); Orthogonal Reactivities of Allenoates **2** with Tetra-Substituted Michael Acceptors **7** Using Different Lewis Bases (B); and the Herein Reported Catalyst-Controlled Sequential Annulation of Allenoates and Fully Substituted 1,3-Indandione Derived Electron-Deficient Alkenes (C)

Hence, inspired by the above-mentioned work, we envisioned that fully substituted enones **10** will proceed different annulation reaction pathways with allenoates **2** when different Lewis base catalysts were used (Scheme 1C). In this regard, we recognized that enones **10** indeed reacted with allenoates **2** in the presence of DMAP and the formal (4 + 2) cycloaddition products with a quaternary center **11** and **12** were formed in good to high yields (Scheme 1C, path a). When enones **10** reacted with allenoates **2** with PPh_3_ as the catalyst, the unusual [3 + 2] cycloaddition products **13** were generated in moderate to high yields (Scheme 1C, path b). In general, the [3 + 2] cycloaddition of enones with allenoates **2** gave the annulation products with bondings at α and γ positions. However, the [3 + 2] cycloaddition products **13** were obtained through β and γ linkages. Moreover, we observed that more complex spiroindane-1,3-diones containing a fused carbocycle were synthesized when γ-vinyl allenoates **14** were used and PPh_3_ was the catalyst (Scheme 1C, path c). Continuing our efforts on developing efficient vinylogous reactions of 1,3-indanedione derivatives,^18^ we herein describe a catalyst-controlled protocol for these three annulation reactions, their substrate scope, and plausible reaction mechanism to rationalize of the site-selectivity reaction pathway.

## Results and Discussion

We first focused our effort on the investigation of tertiary amines for their ability to catalyze the reaction of 1,3-indanedione **10a** with allenoate **2a**. On the basis of previous work,^9c,17^ the expected [4 + 2] annulation product **11a** was obtained in 67% total reaction yield (*Z*-**11a**: 44%, *E*-**11a**: 23%) when 20 mol % of DMAP was used as the catalyst (Table 1, entry 1). Neither DABCO nor DBU provided any reactivity in this reaction (Table 1, entries 2 and 3). We then chose DMAP as the catalyst and examined the solvent effects (Table 1, entries 4–7). Among the tested solvents, THF was found to be the solvent of choice, improving the total reaction yield to 76% (Table 1, entry 7). Lowing the reaction temperature to 40 °C had almost the same results as in 60 °C (Table 1, entries 8 and 9). The survey on the reaction concentration indicated that a further improvement in reaction yield when 2 mL of THF was used (Table 1, entries 10 and 11). In order to maximize the total reaction yield of **11a**, we attempted to elevate the reaction temperature to 80 °C; however, the solvent had to change to high boiling point solvent like toluene. Encouragingly, the total reaction yield of **11a** was increased to 92% (Table 1, entry 12). The investigation showed that there was no positive effect observed when increasing the catalyst loading from 20 to 50 and 100 mol % (Table 1, entries 13 and 14). Hence, the final optimal conditions were chosen by conducting the reaction at 80 °C in 2 mL of toluene with 20 mol % DMAP (Table 1, entry 12).

**Table 1:**
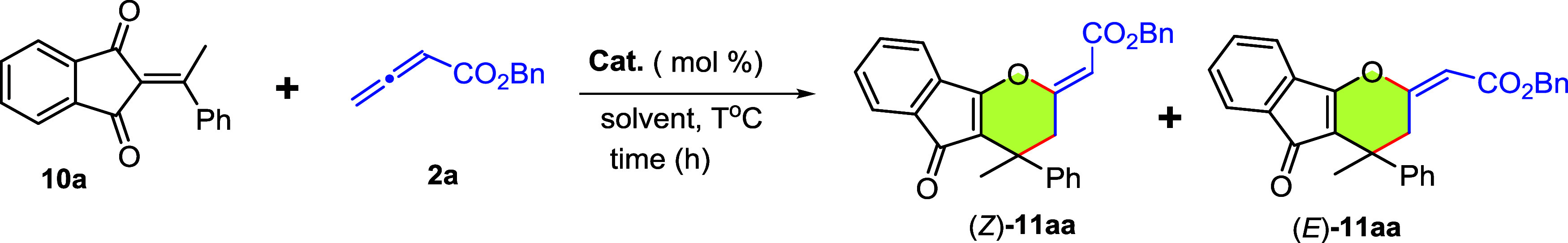
Optimization of the Reaction Conditions for the [4 + 2] Annulation of **10a** and **2a** Catalyzed by Nitrogen-Containing Lewis Bases.^a^

entry	**Cat.**(mol %)	temp. (°C)	solvent (mL)	time (h)	yield (%)*Z*/*E*-**11a** ^b^
1	DMAP (20)	60	toluene (3)	48	44/23
2	DABCO (20)	60	toluene (3)	72	trace
3	DBU (20)	60	toluene (3)	72	trace
4	DMAP (20)	60	CH_3_CN (3)	48	trace
5	DMAP (20)	60	EA (3)	48	35/23
6	DMAP (20)	60	1,2-DCE (3)	24	36/22
7	DMAP (20)	60	THF (3)	48	49/27
8	DMAP (20)	40	THF (3)	48	48/27
9	DMAP (20)	20	THF (3)	48	45/24
10	DMAP (20)	40	THF (2)	48	50/29
11	DMAP (20)	40	THF (1)	48	34/21
12	DMAP (20)	80	toluene (2)	48	60/32
13	DMAP (50)	80	toluene (2)	48	53/13
14	DMAP (100)	80	toluene (2)	48	57/2

a The reaction was carried out by using 0.10 mmol of **10a**, 0.15 mmol of **2a**, and catalyst (20–100 mol %) in solvent (1–3 mL) at 20–80 °C for 24–72h.

b Isolated yields.

With the optimal conditions in hand, the evaluation of substrate scope applying 1,3-indanediones **10** and allenoates **2** under the standard reaction conditions was performed. As shown in Scheme 2, the reaction proceeded smoothly regardless of the steric and electronic properties of the substituents on the aromatic ring, providing the corresponding [4 + 2] annulation products **11ab**–**11ag** in good to high yields. In addition, 1,3-indanedione **10h** showed relatively high reactivity and provided the product **11ah** in high yield (90% total yield). A larger scale reaction (1 mmol) of compounds **10e** and **2a** could smoothly take place to give product **11ae** with the almost same reaction yield and diastereoselectivity. The change of ester groups at the allenoates **2** have some effects on the reaction activity. The 3-nitrophenyl allenoate **2c** gave the corresponding [4 + 2] annulation product **11ca** in moderate yield due to its poor solubility in toluene. The *t*-butyl allenoate **2k** also delivered the annulation product **11ka** in moderate yield because the steric effect on the reactivity.

**Scheme 2:**
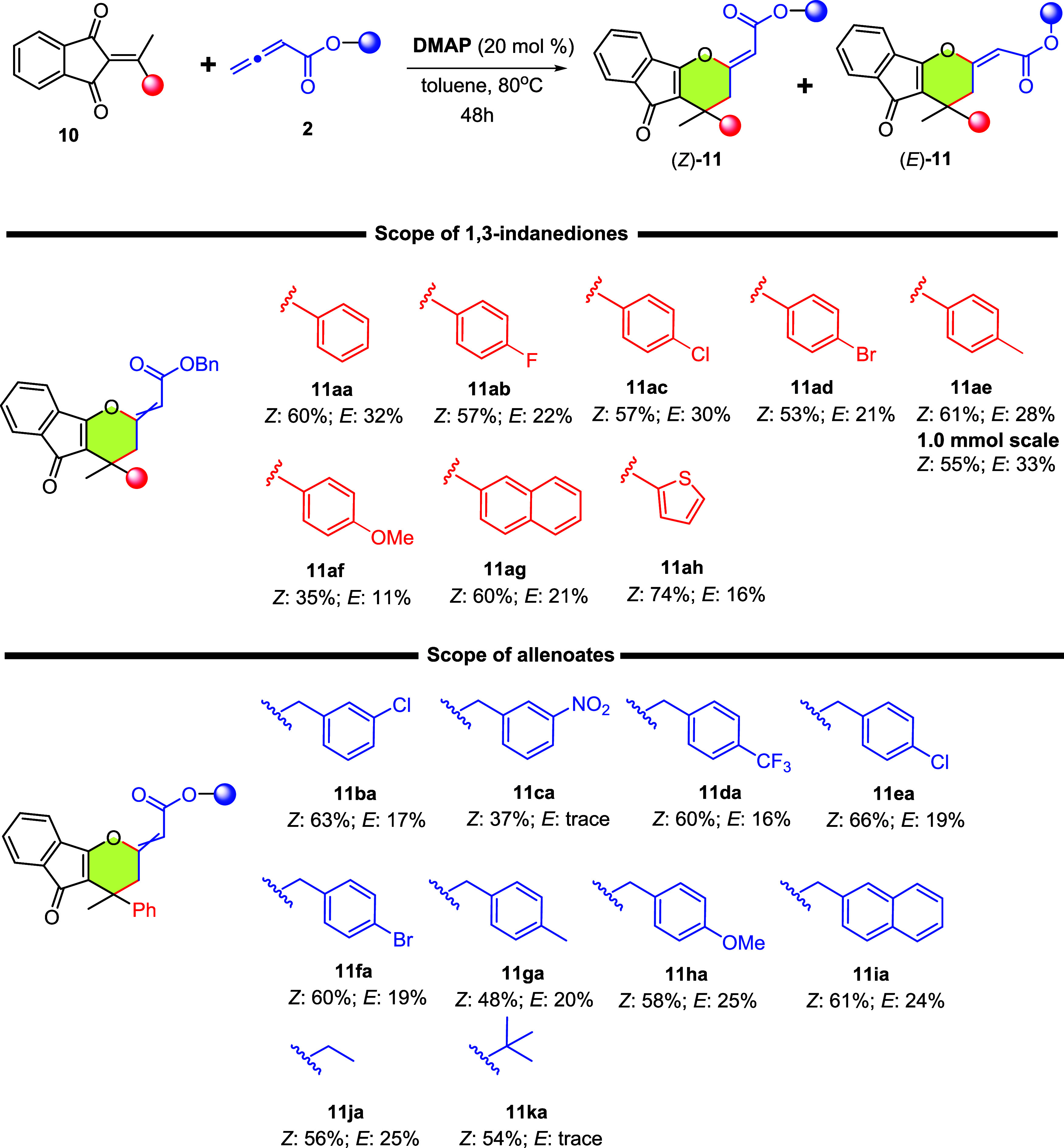
Scope of [4 + 2] Annulation Products.^a^,^b^

a Unless otherwise noted, the reaction was carried out by using 0.1 mmol of **10**, 0.15 mmol of **2**, and 20 mol % of DMAP in 2 mL of toluene at 80 °C for 48h.
b Isolated yields.

However, other allenoates worked well with **10a** and provided the corresponding products **11ba**-**11ja** in good to high yields regardless of the electronic properties of the substituents on the aromatic ring. The structure of **11ad** (CCDC 2479629), including the assignment of the olefin as the *E* isomer, was determined by single-crystal X-ray structural analysis.^19^ With the advantage of the crystallographic structure of **11ad**, all other product structures were deduced by referring to that.

We next started to investigate the scope of applying various dialkyl-substituted 1,3-indanediones **10i**–**m**. As shown in Scheme 3, intriguingly, the endocyclic [4 + 2] annulation products **12** were obtained when the reaction with allenoate **2a**. Reaction of dimethyl-1,3-cyclohexanodiones **10i** and **2a** gave the product **12a** in 71% reaction yield. Application of the cyclic ring-substituted 1,3-indanediones **10j**–**m** with allenoate **2a** only led to products **12c** and **12d** with 77% and 71% reaction yields, respectively. No annulation products were observed when cyclopentyl- and cycloheptyl-substituted 1,3-indanediones (**10j** and **10m**) were used.

**Scheme 3:**
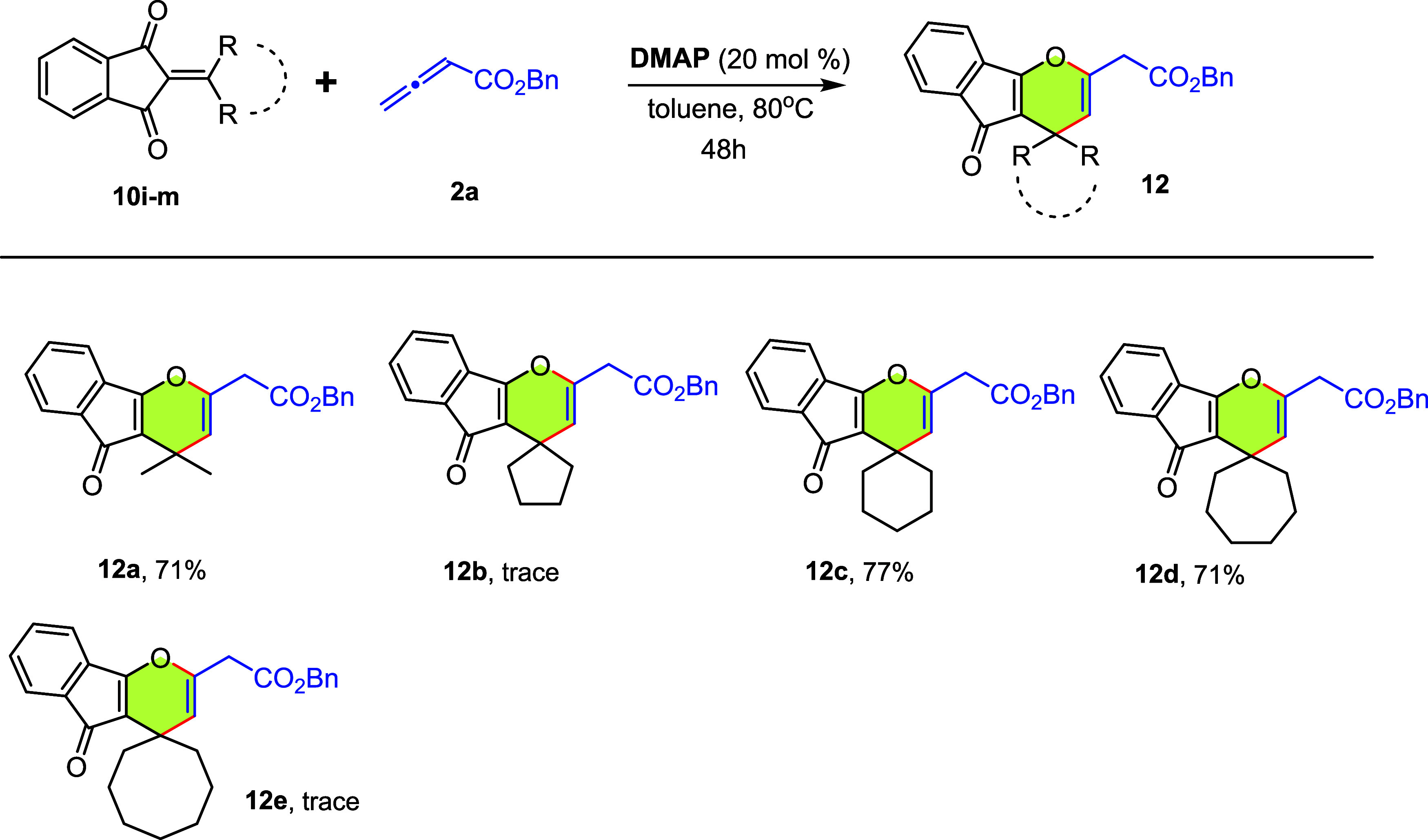
Scope of [4 + 2] Annulation Products Using Dialkyl-Substituted 1,3-Indanediones.^a^,^b^

a Unless otherwise noted, the reaction was carried out by using 0.1 mmol of **10i**–**m**, 0.15 mmol of **2**, and 20 mol % of DMAP in 2 mL of toluene at 80 °C for 48h.
b Isolated yields.

In comparison to amines, phosphines display a markedly different behavior in the reactions of allenoates with electrophilic partners. Hence, the investigation commenced with the reaction of 1,3-indanedione **10a** with allenoate **2a** catalyzed by PPh_3_ at 110 °C for 24h (Table 2, entry 1). To our delight, the [3 + 2] cycloaddition product **13aa** was obtained in 35% yield (Table 2, entry 1). The evaluation of reaction temperature showed that reaction yield could be increased to 47% when the reaction conducted at 60 °C for 48h (Table 2, entries 2–5). Increasing the amounts of **2a** did not improve the reaction yield (Table 2, entries 6 and 7). Among the tested solvents, like CH_3_CN, CH_2_Cl_2_, EA, MeOH, THF, PhCF_3_, *m*-xylene or mesitylene resulted in low efficiency, while the original toluene still gave a better result (Table 2, entries 8–15). Increasing the reaction concentration from 0.05 to 0.1 M showed lower product yield outcome (Table 2, entry 16). In contrast, decreasing the reaction concentration from 0.05 to 0.033 M improved the reaction yield (Table 2, entry 17). The next investigation showed that there was no positive effect observed when increasing the catalyst loading from 20 to 30 mol % (Table 2, entry 18), whereas reducing the catalyst amount to 10 mol % resulted in a slight decrease of the product yield (Table 2, entry 19). Finally, the optimal conditions were selected by conducting the reaction at 60 °C in toluene with 20 mol % of PPh_3_ at 0.033 M substrate concentration (Table 2, entry 17).

**Table 2:**
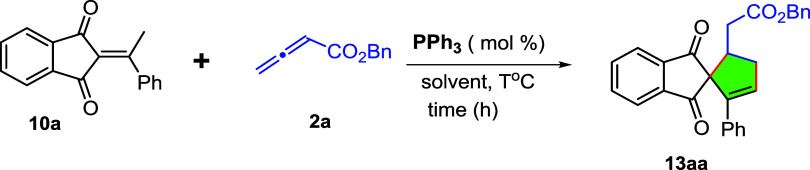
Optimization of the Reaction Conditions for the [3 + 2] Annulation of **10a** and **2a** Catalyzed by Monophosphine Lewis Bases.^a^

entry	**Cat.**(mol %)	temp.	solvent (mL)	time (h)	yield (%)
		(°C)			**13aa** ^b^
1	PPh_3_ (20)	110	toluene (2)	24	35
2	PPh_3_ (20)	85	toluene (2)	24	41
3	PPh_3_ (20)	60	toluene (2)	48	47
4	PPh_3_ (20)	50	toluene (2)	48	28
5	PPh_3_ (20)	rt	toluene (2)	48	trace
6^c^	PPh_3_ (20)	60	toluene (2)	48	36
7^d^	PPh_3_ (20)	60	toluene (2)	48	35
8	PPh_3_ (20)	60	CH_3_CN (2)	48	trace
9	PPh_3_ (20)	60	CH_2_Cl_2_ (2)	48	trace
10	PPh_3_ (20)	60	EA (2)	48	34
11	PPh_3_ (20)	60	MeOH (2)	48	trace
12	PPh_3_ (20)	60	THF (2)	24	34
13	PPh_3_ (20)	60	PhCF_3_ (2)	48	35
14	PPh_3_ (20)	60	*m*-xylene (2)	48	42
15	PPh_3_ (20)	60	mesitylene (2)	48	35
16	PPh_3_ (20)	60	toluene (1)	48	32
17	PPh_3_ (20)	60	toluene (3)	48	50
18	PPh_3_ (30)	60	toluene (3)	48	47
19	PPh_3_ (10)	60	toluene (3)	48	42

a The reaction was carried out by using 0.10 mmol of **10a**, 0.15 mmol of **2a**, and catalyst (20–100 mol %) in solvent (1–3 mL) at 20–110 °C for 24–72h.

b Isolated yields.

c 2.5 equiv of **2a**.

d 3.5 equiv of **2a**.

Having identified the optimal reaction conditions, we set out to examine the scope and the limitations of the Lewis base catalyzed [3 + 2] annulation. It was intriguing to find that this reaction demonstrated a wide scope of substrates, providing products **13** with good yields. A diverse array of 1,3-indanediones **10** with different substituents bearing halide, electron-donating, or electron-withdrawing groups of the phenyl ring, were amenable to this strategy, delivering the desired products **13aa**–**13ah** in moderate to good yields. Notably, the substrates bearing 4-Cl, 4-Br or 4-OMe substituents on the phenyl ring (**10c**, **10d** and **10f**) could also afford the desired products (**13ac**, **13ad** and **13af**) albeit with decreased yields (36%, 28% and 33% respectively). The structure of **13ac** (CCDC 2479639) was confirmed by single-crystal X-ray structural analysis.^19^ A larger scale reaction (1 mmol) of compounds **10e** and **2a** could smoothly take place to give product **13ae** with a slightly decreased reaction yield. Moreover, the change of ester groups at the allenoates **2** also worked well with **10a** and provided the corresponding products **13ba**-**11ka** in moderate to good yields. The 4-methoxyphenyl allenoate **2h** and *t*-butyl allenoate **2k** gave the corresponding [3 + 2] annulation product **13ha** and **13ka** in moderate yields (28% and 22%).

Recently, the γ-vinyl allenoates had been shown to undergo [3 + 2]/ [3 + 2] sequential annulation with alkylidenemalononitriles enabled by phosphine catalysis.^11a,20^ Therefore, we then carried out the annulation reactions of 1,3-indanediones **10** with γ-vinyl allenoate **14** catalyzed by Lewis base PPh_3_. As shown in Scheme 5, a broad range of spiroindane-1,3-diones **15** containing a bicyclic­[3,3,0]­octane scaffold can be synthesized in good to high yields (58–88%) under the same standard conditions in Scheme 4. The relative configuration of **15c** (CCDC 2479640) was revealed by X-ray diffraction analysis, and the configurations of other products were assigned by analogy (Scheme 5).^19^

**Scheme 4:**
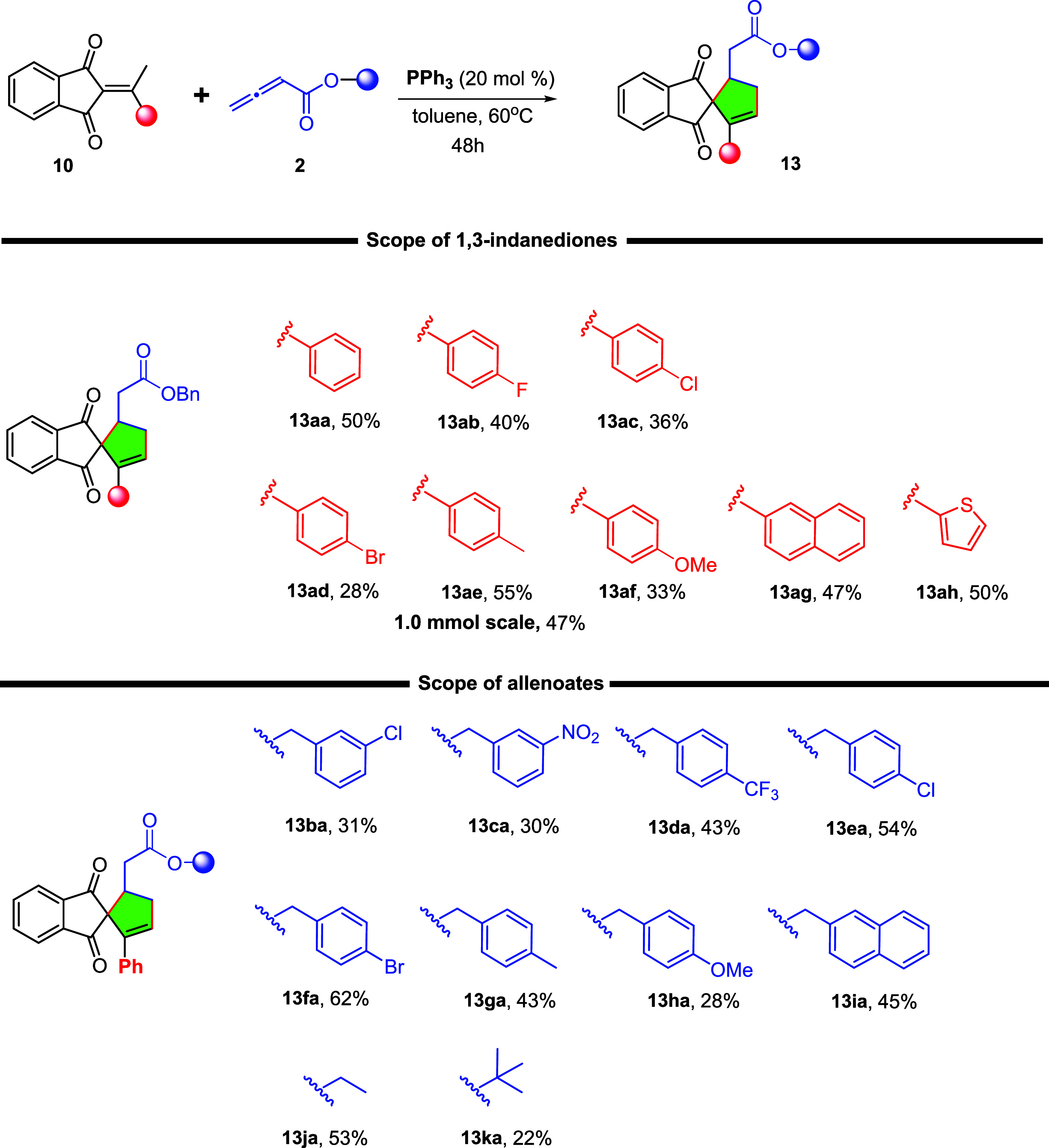
Scope of [3 + 2] Annulation Products.^a^,^b^

a Unless otherwise noted, the reaction was carried out by using 0.1 mmol of **10**, 0.15 mmol of **2**, and 20 mol % of PPh_3_ in 2 mL of toluene at 60 °C for 48h.
b Isolated yields.

**Scheme 5:**
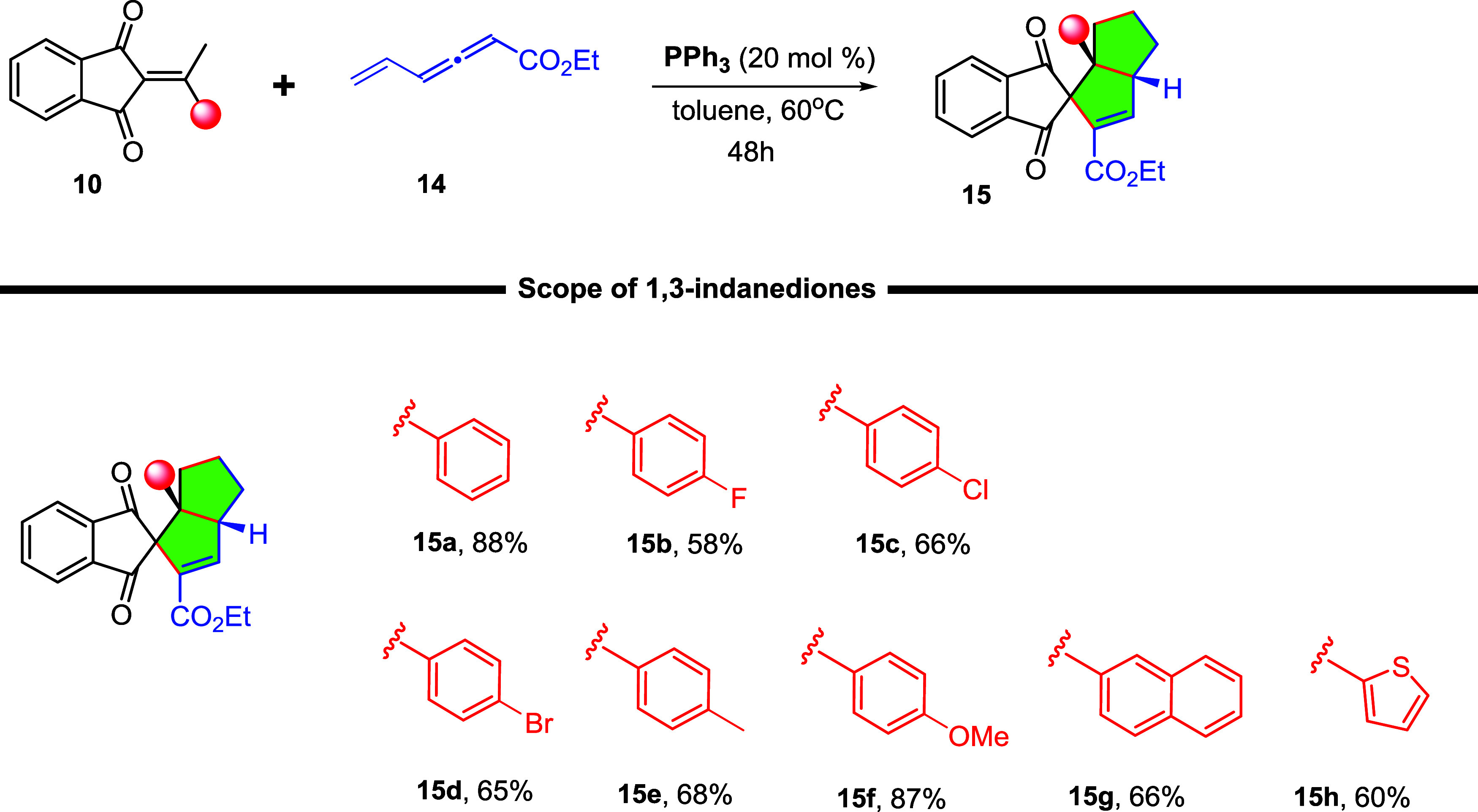
Scope of [3 + 2]/[3 + 2] Annulation Products^a^,^b^

a Unless otherwise noted, the reaction was carried out by using 0.1 mmol of **10**, 0.15 mmol of **2**, and 20 mol % of PPh_3_ in 2 mL of toluene at 60 °C for 48h.
b Isolated yields. *dr* was determined from ^1^H NMR of the crude reaction mixture, and in all cases >20:1 dr were observed.

To illustrate the usefulness of of annulation products **11** and **13**, the postfunctionalization of products (*Z*)-**11aa** and **13aa** were carried out, providing reduction products **16** and **17** in 67% and 60% yields, respectively (Scheme 6). The diastereoselectivity was high (>20:1) beacuse the hydride attacked the sterically less-hindered face and provided the high stereoselective products. (See the Supporting Information for the relative configuration determination)

**Scheme 6:**
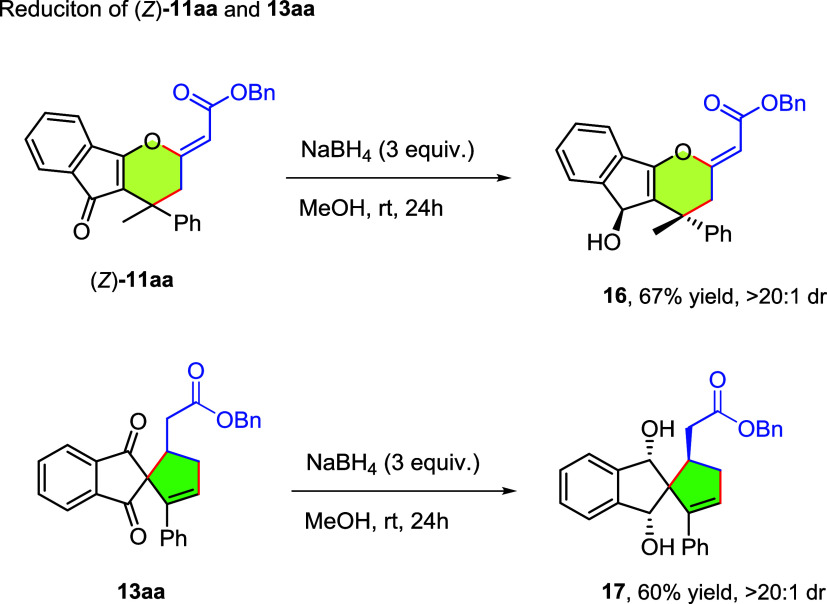
Further Transformation

On the basis of earlier reports^9c,17,20,21^ and our experimental results, possible mechanism processes under the catalysis of phosphine- and nitrogen-containing Lewis bases are proposed in Scheme 7. In the case of the DMAP-catalyzed reaction,^9c,17^ the catalyst reacts with the allenoate **2a** to generate a zwitterionic intermediate **A**, which serves as a nucleophile for the subsequent Michael addition with **10a** to give intermediate **B**. The following cyclization produces [4 + 2] annulation product **11aa** and regenerates the DMAP catalyst. In the PPh_3_-catalyzed phathway,^20,21^ the methyl group of **10a** is believed to be deprotonated by the zwitterion **D** first to form the carbanion because of its acidic property. The formed carbanion could react with intermediate **D-1** via the γ-addition and generated zwitterion **F**. The subsequent intramolecular cyclization yielded [3 + 2] annulation compound **13aa**. For the PPh_3_-catalyzed (3 + 2)/(3 + 2) annulation reaction^11a,20^ of 1,3-indanediones **10a** with γ-vinyl allenoate **14**, the plausible mechanism is shown in Scheme 8. Initially, nucleophilic addition of phosphine to γ-vinyl allenoates would generate the zwitterionic intermediate **G**, which would abstract hydrogen from a pronucleophile **10a** to yield nucleophile **10a-1** and acyclic diene intermediate **G-1**. The ε-C position of **G-1** was electrophilic and induced the 1,7-addition to form the intermediate **H**. Then subsequent stepwise proton shifts of **H** gave rise to the key intermediate **I**, in which the following intramolecular 1,4-addition of **I** happened and produced intermediate **J**, which finished the first (3 + 2) annulation process. The subsequent another intramolecular umpolung addition (the second (3 + 2) annulation) to yield **K**. Finally, the desired product **15a** was obtained through proton transfer and 1,2-elimination of phosphine catalyst via intermediate **L**.

**Scheme 7:**
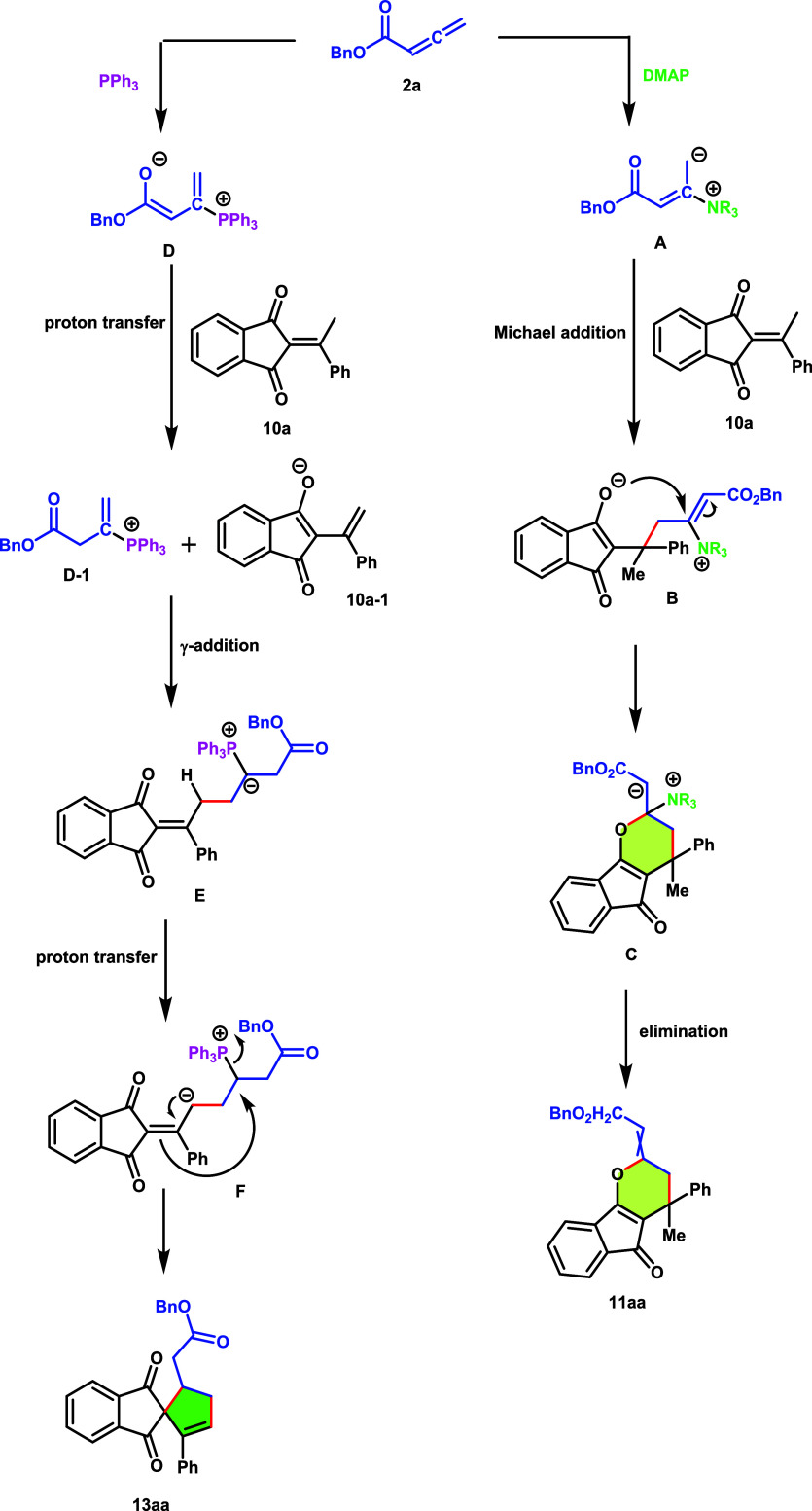
Proposed Mechanisms for the Lewis Base Catalyzed Reactions of 1,3-Indanedione **10a** with Allenoate **2a**

**Scheme 8:**
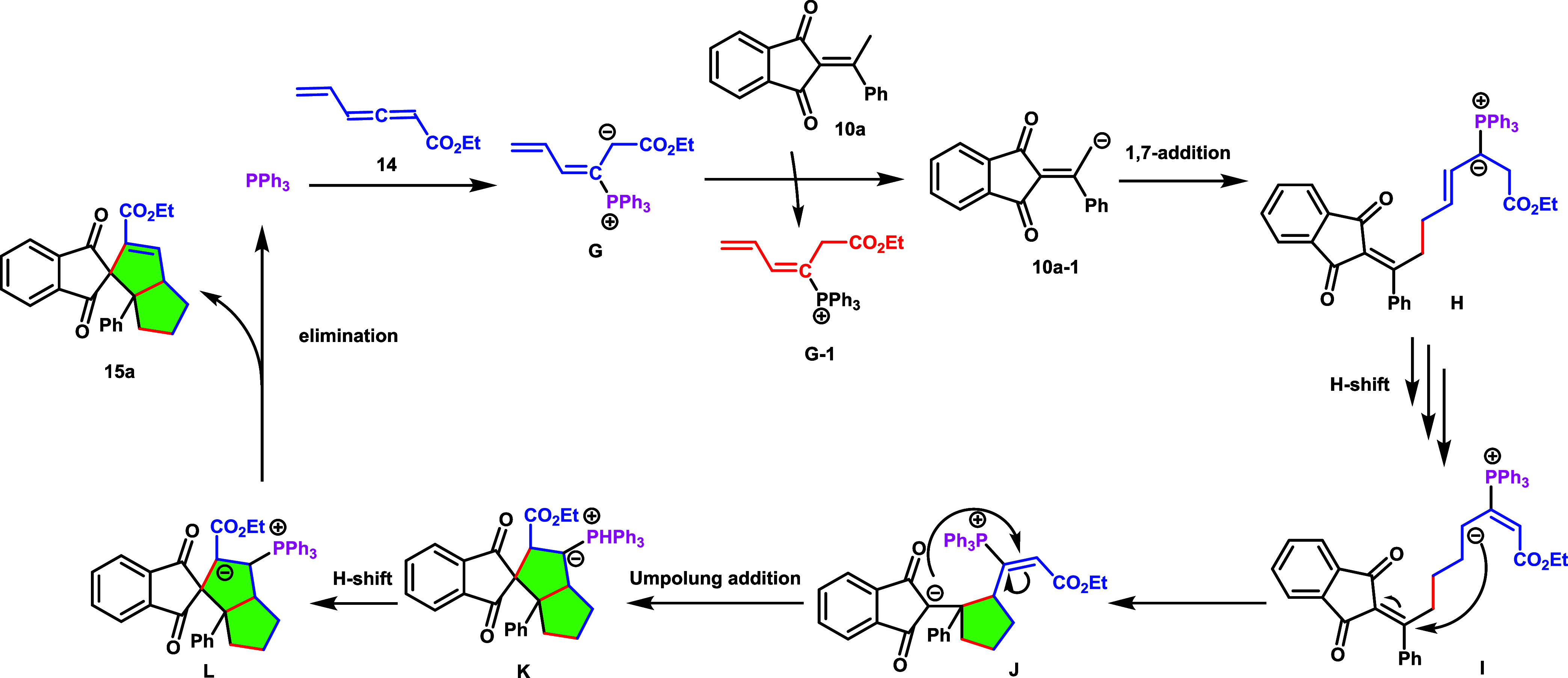
A Plausible Reaction Mechanism for the Lewis Base Catalyzed Reaction of 1,3-Indanedione **10a** with γ-Vinyl Allenoate **14**

To gain further mechanistic insights into the process, we conducted DFT calculations performed DFT calculations at the M06–2X-D3/def2-TZVPP/SMD (Toluene, SAS)//M06–2*X*/6–31G (d,p)/SDM (Toluene, SAS) theoretical level.

On the basis of catalytic cycles listed in Scheme 7, a comprehensive depiction of the energy profiles for this cascade reaction is provided in Figure 2. For the DMAP-catalyzed reaction, the Michael addition of intermediate **A** to **10a** proceeds with a lower activation barrier (6.0 kcal/mol) than the deprotonation of **10a** by intermediate **A** (11.9 kcal/mol). Therefore, the [4 + 2] annulation pathway is kinetically favored, leading to the formation of product **11aa** (Figure 2a).

**Figure 2:**
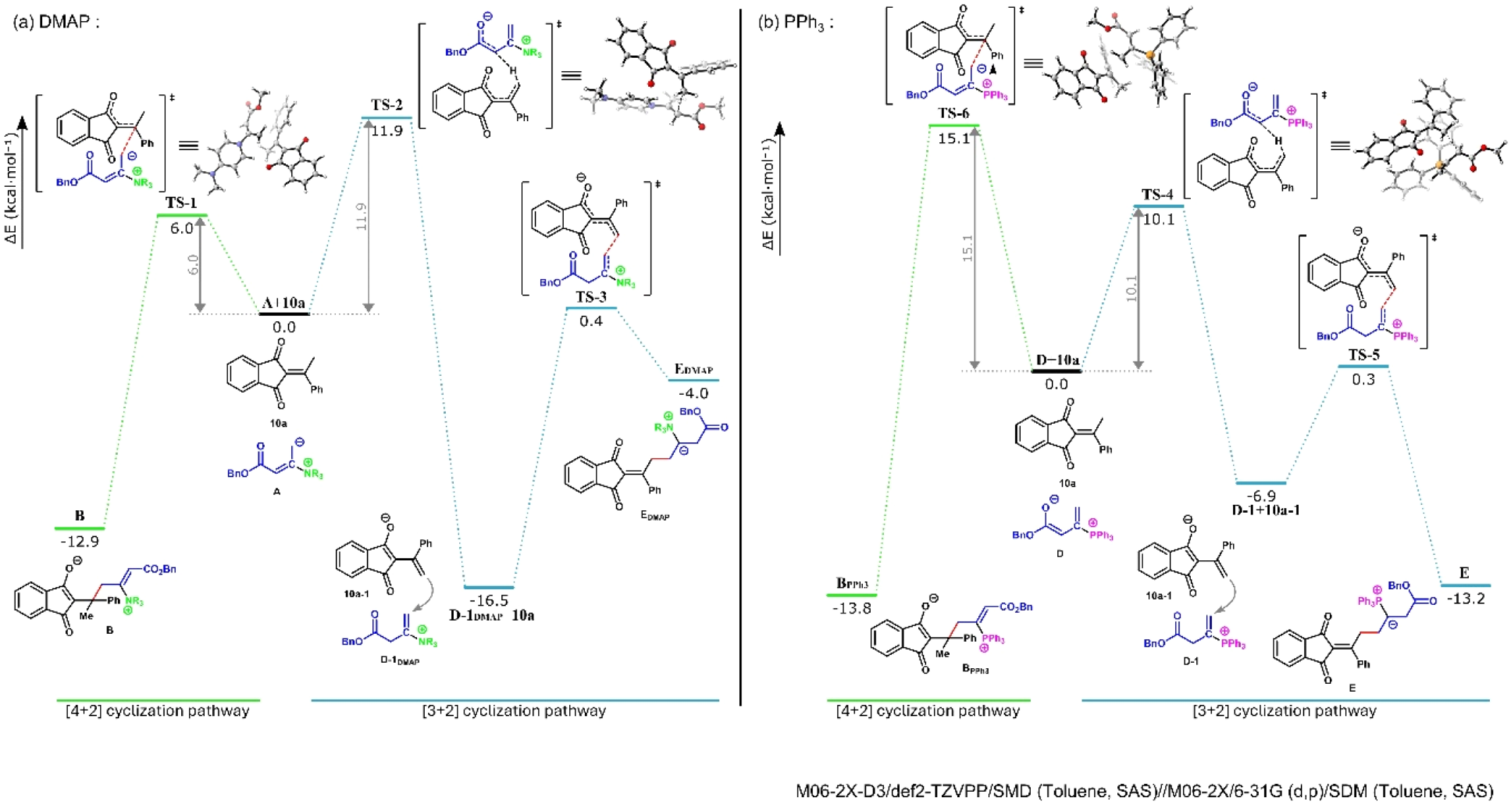
Energy profiles for the DMAP- and PPh_3_-catalyzed reactions of **10a** and **2a**. The relative free energies are given in kcal mol^–1^.

In contrast, for the PPh3-catalyzed reaction, deprotonation of **10a** by intermediate **A** occurs with a lower activation barrier (10.1 kcal/mol) than the Michael addition pathway (15.1 kcal/mol). Consequently, the [3 + 2] annulation pathway is preferred, affording product **13aa** as the major product.

## Conclusion

In summary, a detailed investigation on the different reactivity patterns shown by phosphine- and nitrogen-containing catalysts in the reactions of allenoates with fully substituted 1,3-indandione derived electron-deficient alkenes was accomplished. Under the catalysis of nitrogen-containing Lewis base (DMAP), the formal (4 + 2) annulations occurred and produced 3,4-dihydro-2*H*-pyran derivatives **11** and **12** with a quaternary center in good to high yields. With PPh_3_ as the catalyst, an unusual [3 + 2] annulation proceeded smoothly to afford spiroindane-1,3-diones **13** in moderate to high yields. Moreover, we observed that more complex spiroindane-1,3-diones containing a fused carbocycle could be synthesized via a (3 + 2)/(3 + 2) annulation reaction when γ-vinyl allenoate **14** were used with PPh_3_ as the catalyst. These studies provide an opportunity for diverse synthesis of a variety of 1,3-indandiones and indanones from the same starting materials. We believe that these new libraries of 1,3-indandione derivatives will facilitate their further biological investigation.

### Experimental Section

All reaction solvents were purified before use. Proton nuclear magnetic resonance (^1^H NMR) spectra were recorded on a commercial instrument at 400 MHz. Carbon-13 nuclear magnetic resonance (^13^C­{^1^H } NMR) spectra were recorded at 100 MHz. The proton signal for residual nondeuterated solvent (δ 7.26 for CHCl_3_) was used as an internal reference for ^1^H NMR spectra. For ^13^C­{^1^H} NMR spectra, chemical shifts are reported relative to the δ 77.0 resonance of CDCl_3_. Coupling constants are reported in Hz. Melting points were determined on a BUCHI B-545 melting point apparatus and are uncorrected. High resolution mass spectra were recorded on a commercial high-resolution mass spectrometer with TOF analyzer. The single crystal was measured by Bruker D8 VENTURE X-ray Single Crystal Diffractometer. Analytical thin-layer chromatography (TLC) was performed on silica gel 60 F254 precoated plates with visualization under UV light. Column chromatography was generally performed using 40–63 μm (230–400 mesh) silica gel, typically using a 50–100:1 weight ratio of silica gel to crude product.

### General Procedure for the Synthesis of **11** and **12**

In a 7 mL glass vial, 1,3-indandiones **10** (0.1 mmol, 1.0 equiv), allenoates **2** (0.15 mmol, 1.5 equiv) and DMAP (2.44 mg, 0.02 mmol, 20 mol %) were dissolved in 2.0 mL toluene and stirred for 48 h at 80 °C (oil bath). The solvent was removed in vacuo, then the reaction mixture was purified by column chromatography (SiO_2_, Hexanes: EtOAc, 30:1 to 20:1 to 10:1) to obtain the pure products **11** and **12**.

### Scale-Up Procedure for the Synthesis of Compound **11ae**

In a 25 mL glass vial, 1,3-indandiones **10e** (262 mg, 1.0 mmol, 1.0 equiv), **2a** (261 mg, 1.5 mmol, 1.5 equiv) and DMAP (24.4 mg, 0.2 mmol, 20 mol %) were dissolved in 20 mL toluene and stirred for 48 h at 80 °C (oil bath). The solvent was removed in vacuo, then the reaction mixture was purified by column chromatography (SiO_2_, Hexanes: EtOAc, 30:1 to 20:1 to 10:1) to obtain the pure products (*Z*)-**11** (240 mg, 55% yield) and (*E*)-**11**(144 mg, 33% yield).

### General Procedure for the Synthesis of **13**

In a 7 mL glass vial, 1,3-indandiones **10** (0.1 mmol, 1.0 equiv), allenoates **2** (0.15 mmol, 1.5 equiv) and PPh_3_ (5.24 mg, 0.02 mmol, 20 mol %) were dissolved in 3.0 mL toluene and stirred for 48 h at 60 °C (oil bath). The solvent was removed in vacuo, then the reaction mixture was purified by column chromatography (SiO_2_, Hexanes: THF, 12:1 to 8:1) to obtain the pure products **13**.

### General Procedure for the Synthesis of **15**

In a 7 mL glass vial, 1,3-indandiones **10** (0.1 mmol, 1.0 equiv), allenoate **14** (0.15 mmol, 1.5 equiv) and PPh_3_ (5.24 mg, 0.02 mmol, 20 mol %) were dissolved in 2.0 mL toluene and stirred for 48 h at 60 °C (oil bath). The solvent was removed in vacuo, then the reaction mixture was purified by column chromatography (SiO_2_, Hexanes: EtOAc, 20:1 to 10:1) to obtain the pure products **15**.

### Benzyl-(*E*)-2-(4-Methyl-5-Oxo-4-Phenyl-4,5-Dihydroindeno­[1,2-*b*]­Pyran-2­(3*H*)-Ylidene)­Acetate ((*E*)-**11aa**)

Purified by silica gel column chromatography eluting with Hexane/EA 30:1 to 20:1; 32% yield (13.5 mg); Yellow oil; ^1^H NMR (400 MHz, CDCl_3_): δ 7.47 (d, *J* = 6.7 Hz, 1H), 7.41–7.24 (m, 11H), 7.21–7.17 (m, 2H), 5.93 (d, *J =* 1.7 Hz, 1H), 5.20 (s, 2H), 4.49 (d, *J =* 15.2 Hz, 1H), 2.74 (dd, *J =* 15.2, 1.7 Hz, 1H), 1.82 (s, 3H); ^13^C­{^1^H } NMR (101 MHz, CDCl_3_): δ 191.8, 169.1, 165.7, 165.1, 144.9, 136.0, 135.8, 132.3, 132.0, 130.3, 128.5, 128.4, 128.1, 128.0, 126.5, 125.7, 121.7, 118.0, 115.6, 104.0, 66.0, 37.7, 37.4, 27.3; HRMS (ESI-TOF) *m*/*z*: [M + H]^+^ calcd. for C_28_H_23_O_4_: 423.1591, Found: 423.1594.

### Benzyl-(*Z*)-2-(4-Methyl-5-Oxo-4-Phenyl-4,5-Dihydroindeno­[1,2-*b*]­Pyran-2­(3*H*)-Ylidene)­Acetate ((*Z*)-**11aa**)

Purified by silica gel column chromatography eluting with Hexane/EA 20:1 to 10:1; 60% yield (25.5 mg); Yellow soild; m.p.: 93–94 °C; ^1^H NMR (400 MHz, CDCl_3_): δ 7.46–7.35 (m, 6H), 7.33–7.29 (m, 6H), 7.24–7.20 (m, 1H), 7.12–7.07 (m, 1H), 5.31 (d, *J =* 1.3 Hz, 1H), 5.20 (s, 2H), 2.90 (d, *J =* 14.8 Hz, 1H), 2.72 (dd, *J =* 14.7, 1.5 Hz, 1H), 1.80 (s, 3H); ^13^C­{^1^H } NMR (101 MHz, CDCl_3_): δ 191.4, 169.3, 163.3, 159.6, 144.4, 135.9, 135.7, 132.3, 132.0, 130.2, 128.45, 128.38, 128.3, 128.1, 126.6, 125.6, 121.5, 118.4, 114.7, 102.4, 66.0, 43.5, 37.3, 26.3; HRMS (ESI-TOF) *m*/*z*: [M + H]^+^ calcd. for C_28_H_23_O_4_: 423.1591, Found: 423.1597.

### Benzyl-(*E*)-2-(4-(4-Fluorophenyl)-4-Methyl-5-Oxo-4,5-Dihydroindeno­[1,2-*b*]­Pyran-2­(3*H*)-Ylidene)­Acetate ((*E*)-**11ab**)

Purified by silica gel column chromatography eluting with Hexane/EA 30:1 to 20:1; 22% yield (9.6 mg); Yellow oil; ^1^H NMR (400 MHz, CDCl_3_): δ 7.47–7.45 (m, 1H), 7.41–7.28 (m, 9H), 7.21–7.19 (m, 1H), 6.93–6.87 (m, 2H), 5.91 (d, *J* = 1.5 Hz, 1H), 5.18 (s, 2H), 4.41 (d, *J* = 15.2 Hz, 1H), 2.72 (dd, *J* = 15.2, 1.7 Hz, 1H), 1.77 (s, 3H); ^13^C­{^1^H }­NMR (101 MHz, CDCl_3_): δ 191.9, 169.3, 165.8, 164.9, 162.7 (d, *J* = 245.0 Hz), 140.7, 136.0, 135.8, 132.5, 132.0, 130.5, 128.6, 128.3, 128.1, 127.6 (d, *J* = 7.9 Hz), 121.9, 118.2, 115.4, 115.3 (d, *J* = 21.0 Hz), 104.3, 66.2, 37.7, 37.4, 27.5; ^19^F NMR (376 MHz, CDCl_3_): δ −116.3; HRMS (ESI-TOF) *m*/*z*: [M + H]^+^ calcd. for C_28_H_22_O_4_F: 441.1497, Found: 441.1495.

### Benzyl-(*Z*)-2-(4-(4-Fluorophenyl)-4-Methyl-5-Oxo-4,5-Dihydroindeno­[1,2-*b*]­Pyran-2­(3*H*)-Ylidene)­Acetate ((*Z*)-**11ab**)

Purified by silica gel column chromatography eluting with Hexane/EA 20:1 to 10:1; 57% yield (25.8 mg); Yellow oil; ^1^H NMR (400 MHz, CDCl_3_): δ 7.42–7.34 (m, 6H), 7.32–7.22 (m, 5H), 7.06–7.03 (m, 1H), 6.99–6.93 (m, 2H), 5.26 (d, *J* = 1.3 Hz, 1H), 5.16 (s, 2H), 2.81 (d, *J* = 14.8 Hz, 1H), 2.70 (dd, *J* = 14.8, 1.5 Hz, 1H), 1.75 (s, 3H); ^13^C­{^1^H } NMR (101 MHz, CDCl_3_): δ 191.5, 169.6, 163.4, 162.7 (d, *J* = 245.8 Hz), 159.5, 140.2 (d, *J* = 2.9 Hz), 135.9, 135.7, 132.5, 132.0, 130.5, 128.5, 128.5, 128.2, 127.5 (d, *J* = 8.0 Hz), 121.8, 118.7, 115.5 (d, *J* = 20.9 Hz), 114.6, 102.8, 66.3, 44.0, 37.1, 26.5; ^19^F NMR (376 MHz, CDCl_3_): δ −115.9; HRMS (ESI-TOF) *m*/*z*: [M + H]^+^ calcd. for C_28_H_22_O_4_F: 441.1497, Found: 441.1499.

### Benzyl-(*E*)-2-(4-(4-Chlorophenyl)-4-Methyl-5-Oxo-4,5-Dihydroindeno­[1,2-*b*]­Pyran-2­(3*H*)-Ylidene)­Acetate ((*E*)-**11ac**)

Purified by silica gel column chromatography eluting with Hexane/EA 30:1 to 20:1; 30% yield (13.9 mg); Yellow oil; ^1^H NMR (400 MHz, CDCl_3_): δ 7.47–7.45 (m, 1H), 7.41–7.31 (m, 7H), 7.29–7.25 (m, 2H), 7.20–7.16 (m, 3H), 5.90 (d, *J =* 1.5 Hz, 1H), 5.17 (s, 2H), 4.40 (d, *J =* 15.2 Hz, 1H), 2.71 (dd, *J =* 15.2, 1.7 Hz, 1H), 1.75 (s, 3H); ^13^C­{^1^H } NMR (101 MHz, CDCl_3_): δ 191.9, 169.4, 165.8, 164.7, 143.6, 136.0, 135.8, 132.5, 132.4, 132.0, 130.6, 128.9, 128.6, 128.6, 128.3, 128.3, 128.2, 127.4, 121.9, 118.2, 115.2, 104.4, 66.2, 37.5, 37.4, 27.3; HRMS (ESI-TOF) *m*/*z*: [M + H]^+^ calcd. for C_28_H_22_O_4_Cl: 457.1201, Found: 457.1200.

### Benzyl-(*Z*)-2-(4-(4-Chlorophenyl)-4-Methyl-5-Oxo-4,5-Dihydroindeno­[1,2-*b*]­Pyran-2­(3*H*)-Ylidene)­Acetate ((*Z*)-**11ac**)

Purified by silica gel column chromatography eluting with Hexane/EA 20:1 to 10:1; 57% yield (26.1 mg); Yellow oil; ^1^H NMR (400 MHz, CDCl_3_): δ 7.44–7.34 (m, 6H), 7.32–7.29 (m, 2H), 7.27–7.22 (m, 4H), 7.09–7.04 (m, 1H), 5.27 (d, *J =* 1.3 Hz, 1H), 5.18 (s, 2H), 2.82 (d, *J =* 14.8 Hz, 1H), 2.72 (dd, *J =* 14.7, 1.5 Hz, 1H), 1.76 (s, 3H); ^13^C­{^1^H } NMR (101 MHz, CDCl_3_): δ 191.4, 169.7, 163.4, 159.3, 143.1, 135.9, 135.8, 132.7, 132.6, 132.1, 130.5, 128.7, 128.6, 128.5, 128.3, 127.3, 121.8, 118.8, 114.3, 102.9, 66.3, 43.8, 37.2, 26.3; HRMS (ESI-TOF) *m*/*z*: [M + H]^+^ calcd. for C_28_H_22_O_4_Cl: 457.1201, Found: 457.1197.

### Benzyl-(*E*)-2-(4-(4-Chlorophenyl)-4-Methyl-5-Oxo-4,5-Dihydroindeno­[1,2-*b*]­Pyran-2­(3*H*)-Ylidene)­Acetate ((*E*)-**11ad**)

Purified by silica gel column chromatography eluting with Hexane/EA 30:1 to 20:1; 21% yield (10.4 mg); Yellow oil; ^1^H NMR (400 MHz, CDCl_3_): δ 7.47–7.45 (m, 1H), 7.41–7.31 (m, 9H), 7.23–7.18 (m, 3H), 5.91 (d, *J =* 1.5 Hz, 1H), 5.18 (s, 2H), 4.41 (d, *J =* 15.2 Hz, 1H), 2.71 (dd, *J =* 15.2, 1.7 Hz, 1H), 1.76 (s, 3H); ^13^C­{^1^H } NMR (101 MHz, CDCl_3_): δ 191.9, 169.4, 165.8, 164.7, 144.1, 135.9, 135.7, 132.5, 132.0, 131.5, 130.6, 128.6, 128.3, 128.2, 127.8, 121.9, 120.6, 118.2, 115.1, 104.4, 66.2, 37.6, 37.3, 27.3; HRMS (ESI-TOF) *m*/*z*: [M + Na]^+^ calcd. for C_28_H_21_O_4_BrNa: 523.0515, Found: 523.0523.

### Benzyl-(*Z*)-2-(4-(4-Chlorophenyl)-4-Methyl-5-Oxo-4,5-Dihydroindeno­[1,2-*b*]­Pyran-2­(3*H*)-Ylidene)­Acetate ((*Z*)-**11ad**)

Purified by silica gel column chromatography eluting with Hexane/EA 20:1 to 10:1; 53% yield (26.8 mg); Yellow oil; ^1^H NMR (400 MHz, CDCl_3_): δ 7.43–7.36 (m, 8H), 7.34–7.29 (m, 2H), 7.17 (d, *J* = 8.6 Hz, 2H), 7.09–7.04 (m, 1H), 5.28 (d, *J =* 1.4 Hz, 1H), 5.18 (s, 2H), 2.82 (d, *J =* 14.8 Hz, 1H), 2.71 (dd, *J =* 14.8, 1.5 Hz, 1H), 1.75 (s, 3H); ^13^C­{^1^H }­NMR (101 MHz, CDCl_3_): δ 191.4, 169.7, 163.4, 159.3, 143.6, 135.9, 135.8, 132.6, 132.1, 131.7, 130.5, 128.6, 128.5, 128.3, 127.7, 121.8, 120.8, 118.8, 114.2, 103.0, 66.3, 43.8, 37.3, 26.3; HRMS (ESI-TOF) *m*/*z*: [M + H]^+^ calcd. for C_28_H_22_O_4_Br: 501.0696, Found: 501.0698.

### Benzyl-(*E*)-2-(4-Methyl-5-Oxo-4-(*p*-Tolyl)-4,5-Dihydroindeno­[1,2-*b*]­Pyran-2­(3*H*)-Ylidene)­Acetate ((*E*)-**11ae**)

Purified by silica gel column chromatography eluting with Hexane/EA 30:1 to 20:1; 28% yield (12.1 mg); Yellow oil; ^1^H NMR (400 MHz, CDCl_3_): δ 7.45 (d, *J* = 7.1 Hz, 1H), 7.41–7.30 (m, 7H), 7.24 (d, *J* = 8.2 Hz, 2H), 7.18 (d, *J* = 6.2 Hz, 1H), 7.04 (d, *J* = 8.0 Hz, 2H), 5.90 (d, *J* = 1.6 Hz, 1H), 5.19 (s, 2H), 4.43 (d, *J* = 15.3 Hz, 1H), 2.72 (dd, *J* = 15.3, 1.7 Hz, 1H), 2.28 (s, 3H), 1.76 (s, 3H); ^13^C­{^1^H }­NMR (101 MHz, CDCl_3_): δ 192.0, 169.1, 165.9, 165.3, 142.1, 136.2, 136.1, 135.9, 132.4, 132.1, 130.4, 129.2, 128.6, 128.2, 128.1, 125.7, 121.8, 118.0, 115.9, 104.1, 66.1, 37.5, 37.5, 27.5, 20.9; HRMS (ESI-TOF) *m*/*z*: [M + H]^+^ calcd. for C_29_H_25_O_4_: 437.1747, Found: 437.1745.

### Benzyl-(*Z*)-2-(4-Methyl-5-Oxo-4-(*p*-Tolyl)-4,5-Dihydroindeno­[1,2-*b*]­Pyran-2­(3*H*)-Ylidene)­Acetate ((*Z*)-**11ae**)

Purified by silica gel column chromatography eluting with Hexane/EA 20:1 to 10:1; 61% yield (26.7 mg); Yellow solid; m.p.: 110–111 °C; ^1^H NMR (400 MHz, CDCl_3_): δ 7.43–7.36 (m, 5H), 7.32–7.28 (m, 2H), 7.19 (d, *J* = 8.3 Hz, 2H), 7.10 (d, *J* = 8.5 Hz, 2H), 7.06–7.02 (m, 1H), 5.28 (d, *J* = 1.3 Hz, 1H), 5.18 (s, 2H), 2.87 (d, *J* = 14.7 Hz, 1H), 2.71 (dd, *J* = 14.7, 1.5 Hz, 1H), 2.30 (s, 3H), 1.76 (s, 3H); ^13^C­{^1^H }­NMR (101 MHz, CDCl_3_): δ 191.6, 169.4, 163.6, 159.9, 141.5, 136.4, 136.1, 135.9, 132.4, 132.2, 130.3, 129.3, 128.6, 128.5, 128.2, 125.6, 121.7, 118.6, 115.1, 102.5, 66.2, 43.9, 37.2, 26.5, 20.9; HRMS (ESI-TOF) *m*/*z*: [M + H]^+^ calcd. for C_29_H_25_O_4_: 437.1747, Found: 437.1745.

### Benzyl-(*E*)-2-(4-(4-Methoxyphenyl)-4-Methyl-5-Oxo-4,5-Dihydroindeno­[1,2-*b*]­Pyran-2­(3*H*)-Ylidene)­Acetate ((*E*)-**11af**)

Purified by silica gel column chromatography eluting with Hexane/EA 30:1 to 20:1; 11% yield (5.1 mg); Yellow oil; ^1^H NMR (400 MHz, CDCl_3_): δ 7.44 (d, *J* = 7.6 Hz, 1H), 7.39–7.28 (m, 7H), 7.24 (d, *J* = 2.8 Hz, 3H), 7.16 (d, *J* = 6.8 Hz, 1H), 6.74 (d, *J* = 8.8 Hz, 2H), 5.89 (d, *J* = 1.6 Hz, 1H), 5.17 (s, 2H), 4.40 (d, *J* = 15.2 Hz, 1H), 3.74 (s, 2H), 2.68 (dd, *J* = 15.2, 1.8 Hz, 1H), 1.75 (s, 3H); ^13^C­{^1^H } NMR (101 MHz, CDCl_3_): δ 192.0, 169.1, 165.9, 165.3, 158.1, 137.1, 136.2, 135.9, 132.4, 132.1, 130.4, 128.6, 128.4, 128.2, 128.1, 126.9, 121.8, 118.0, 116.0, 113.8, 104.1, 66.1, 55.1, 37.7, 37.2, 27.5; HRMS (ESI-TOF) *m*/*z*: [M + Na]^+^ calcd. for C_29_H_24_O_5_Na: 475.1516, Found: 475.1505.

### Benzyl-(*Z*)-2-(4-(4-Methoxyphenyl)-4-Methyl-5-Oxo-4,5-Dihydroindeno­[1,2-*b*]­Pyran-2­(3*H*)-Ylidene)­Acetate ((*Z*)-**11af**)

Purified by silica gel column chromatography eluting with Hexane/EA 20:1 to 10:1; 35% yield (15.7 mg); Yellow oil; ^1^H NMR (400 MHz, CDCl_3_): δ 7.43–7.35 (m, 5H), 7.32–7.28 (m, 3H), 7.22 (d, *J* = 8.8 Hz, 2H), 7.06–7.04 (m, 1H), 6.82 (d, *J* = 8.8 Hz, 2H), 5.27 (d, *J* = 1.3 Hz, 1H), 5.18 (s, 2H), 3.76 (s, 3H), 2.84 (d, *J* = 14.7 Hz, 1H), 2.70 (dd, *J* = 14.7, 1.5 Hz, 1H), 1.75 (s, 3H); ^13^C­{^1^H }­NMR (101 MHz, CDCl_3_): δ 191.6, 169.4, 163.6, 160.0, 158.2, 136.6, 136.1, 135.9, 132.4, 132.2, 130.3, 128.6, 128.5, 128.2, 126.9, 121.7, 118.6, 115.1, 113.9, 102.6, 66.2, 55.2, 44.1, 36.9, 26.6; HRMS (ESI-TOF) *m*/*z*: [M + H]^+^ calcd. for C_29_H_25_O_5_: 453.1697, Found: 453.1694.

### Benzyl-(*E*)-2-(4-Methyl-4-(Naphthalen-2-yl)-5-Oxo-4,5-Dihydroindeno­[1,2-*b*]­Pyran-2­(3*H*)-Ylidene)­Acetate ((*E*)-**11ag**)

Purified by silica gel column chromatography eluting with Hexane/EA 30:1 to 20:1; 21% yield (9.8 mg); Yellow solid; m.p.: 134–135 °C; ^1^H NMR (400 MHz, CDCl_3_): δ 7.66–7.63 (m, 3H), 7.54–7.52 (m, 1H), 7.47 (dd, *J =* 8.7, 2.0 Hz, 1H), 7.41–7.39 (m, 1H), 7.33–7.23 (m, 9H), 7.12–7.10 (m, 1H), 5.82 (d, *J =* 1.5 Hz, 1H), 5.13 (d, *J =* 12.4 Hz, 1H), 5.08 (d, *J =* 12.4 Hz, 1H), 4.54 (d, *J =* 15.3 Hz, 1H), 2.69 (dd, *J =* 15.3, 1.7 Hz, 1H), 1.78 (s, 3H); ^13^C­{^1^H }­NMR (101 MHz, CDCl_3_): δ 192.0, 169.4, 166.0, 165.2, 142.4, 136.1, 135.9, 133.2, 132.4, 132.1, 130.5, 128.6, 128.3, 128.24, 128.18, 127.3, 125.9, 125.7, 124.8, 124.0, 121.9, 118.1, 115.7, 104.2, 66.2, 38.1, 37.3, 27.4; HRMS (ESI-TOF) *m*/*z*: [M + H]^+^ calcd. for C_32_H_25_O_4_: 473.1747, Found: 473.1750.

### Benzyl-(*Z*)-2-(4-Methyl-4-(Naphthalen-2-yl)-5-Oxo-4,5-Dihydroindeno­[1,2-*b*]­Pyran-2­(3*H*)-Ylidene)­Acetate ((*Z*)-**11ag**)

Purified by silica gel column chromatography eluting with Hexane/EA 20:1 to 10:1; 60% yield (28.5 mg); Yellow oil; ^1^H NMR (400 MHz, CDCl_3_): δ 7.81–7.77 (m, 3H), 7.70 (d, *J =* 2.0 Hz, 1H), 7.49–7.33 (m, 11H), 7.13–7.10 (m, 1H), 5.30 (d, *J =* 1.2 Hz, 1H), 5.17 (s, 2H), 3.01 (d, *J =* 14.8 Hz, 1H), 2.78 (dd, *J =* 14.8, 1.5 Hz, 1H), 1.88 (s, 3H); ^13^C­{^1^H}­NMR (101 MHz, CDCl_3_): δ 191.6, 169.7, 163.4, 159.7, 141.8, 136.0, 135.8, 133.2, 132.5, 132.2, 130.4, 128.5, 128.4, 128.2, 128.1, 127.4, 126.1, 125.9, 124.4, 124.1, 121.8, 118.7, 114.7, 102.7, 66.2, 43.8, 37.7, 26.4; HRMS (ESI-TOF) *m*/*z*: [M + H]^+^ calcd. for C_32_H_25_O_4_: 473.1747, Found: 473.1747.

### Benzyl-(*E*)-2-(4-Methyl-5-Oxo-4-(Thiophen-2-yl)-4,5-Dihydroindeno­[1,2-*b*]­Pyran-2­(3*H*)-Ylidene)­Acetate ((*E*)-**11ah**)

Purified by silica gel column chromatography eluting with Hexane/EA 30:1 to 20:1; 16% yield (6.8 mg); Yellow solid; m.p.: 132–133 °C; ^1^H NMR (400 MHz, CDCl_3_): δ 7.46–7.44 (m, 1H), 7.40–7.31 (m, 7H), 7.22–7.20 (m, 1H), 7.10 (dd, *J =* 5.0, 1.3 Hz, 1H), 6.87–6.83 (m, 2H), 5.97 (d, *J =* 1.7 Hz, 1H), 5.20 (d, *J =* 12.5 Hz, 1H), 5.16 (d, *J =* 12.4 Hz, 1H), 4.37 (d, *J =* 15.2 Hz, 1H), 2.79 (dd, *J =* 15.2, 1.8 Hz, 1H), 1.87 (s, 3H); ^13^C­{^1^H } NMR (101 MHz, CDCl_3_): δ 191.2, 169.0, 165.7, 164.4, 150.1, 135.9, 135.8, 132.4, 132.0, 130.6, 128.6, 128.3, 128.2, 126.7, 123.9, 123.3, 121.9, 118.3, 115.0, 105.0, 66.2, 39.0, 35.5, 28.0; HRMS (ESI-TOF) *m*/*z*: [M + H]^+^ calcd. for C_26_H_21_O_4_S: 429.1155, Found: 429.1153.

### Benzyl-(*Z*)-2-(4-Methyl-5-Oxo-4-(Thiophen-2-yl)-4,5-Dihydroindeno­[1,2-*b*]­Pyran-2­(3*H*)-Ylidene)­Acetate ((*Z*)-**11ah**)

Purified by silica gel column chromatography eluting with Hexane/EA 20:1 to 10:1; 74% yield (31.6 mg); Yellow solid; m.p.: 127–128 °C; ^1^H NMR (400 MHz, CDCl_3_): δ 7.43–7.36 (m, 6H), 7.32–7.29 (m, 2H), 7.14 (dd, *J* = 5.1, 1.2 Hz, 1H), 7.07–7.05 (m, 1H), 6.90–6.88 (m, 1H), 6.85–6.84 (m, 1H), 5.33 (s, 1H), 5.20 (s, 2H), 2.85 (d, *J* = 14.7 Hz, 1H), 2.79 (d, *J* = 14.7 Hz, 1H), 1.86 (s, 3H); ^13^C­{^1^H } NMR (101 MHz, CDCl_3_): δ 191.0, 169.1, 163.4, 159.2, 149.6, 135.8, 132.5, 132.1, 130.5, 128.6, 128.5, 128.2, 126.8, 124.0, 123.7, 121.8, 118.8, 114.2, 103.3, 66.3, 44.9, 35.6, 27.3; HRMS (ESI-TOF) *m*/*z*: [M + H]^+^ calcd. for C_26_H_21_O_4_S: 429.1155, Found: 429.1155.

### 3-Chlorobenzyl-(*E*)-2-(4-Methyl-5-Oxo-4-Phenyl-4,5-Dihydroindeno­[1,2-*b*]­Pyran-2­(3*H*)-Ylidene)­Acetate ((*E*)-**11ba**)

Purified by silica gel column chromatography eluting with Hexane/EA 30:1 to 20:1; 17% yield (7.8 mg); Yellow oil; ^1^H NMR (400 MHz, CDCl_3_): δ 7.48–7.45 (m, 1H), 7.41–7.29 (m, 7H), 7.28–7.13 (m, 5H), 5.90 (d, *J =* 1.6 Hz, 1H), 5.14 (s, 2H), 4.44 (d, *J =* 15.3 Hz, 1H), 2.73 (dd, *J =* 15.3, 1.7 Hz, 1H), 1.80 (s, 3H); ^13^C­{^1^H } NMR (101 MHz, CDCl_3_): δ 191.9, 169.2, 165.7, 165.5, 144.9, 137.9, 136.1, 134.4, 132.4, 132.1, 130.4, 129.8, 128.5, 128.4, 128.0, 126.7, 126.0, 125.8, 121.8, 118.1, 115.7, 103.8, 65.1, 37.8, 37.6, 27.4; HRMS (ESI-TOF) *m*/*z*: [M + H]^+^ calcd. for C_28_H_22_O_4_Cl: 457.1201, Found: 457.1201.

### 3-Chlorobenzyl-(*Z*)-2-(4-Methyl-5-Oxo-4-Phenyl-4,5-Dihydroindeno­[1,2-*b*]­Pyran-2­(3*H*)-Ylidene)­Acetate ((*Z*)-**11ba**)

Purified by silica gel column chromatography eluting with Hexane/EA 20:1 to 10:1; 63% yield (28.5 mg); Yellow oil; ^1^H NMR (400 MHz, CDCl_3_): δ 7.43 (ddd, *J =* 7.4, 4.3, 1.5 Hz, 2H), 7.35–7.23 (m, 9H), 7.22–7.21 (m, 1H), 7.14–7.12 (m, 1H), 5.28 (d, *J =* 1.4 Hz, 1H), 5.14 (s, 2H), 2.90 (d, *J =* 14.8 Hz, 1H), 2.74 (dd, *J =* 14.8, 1.2 Hz, 1H), 1.79 (s, 3H); ^13^C­{^1^H}­NMR (101 MHz, CDCl_3_): δ 191.6, 169.4, 163.3, 160.1, 144.4, 137.8, 136.0, 134.4, 132.5, 132.1, 130.4, 129.8, 128.6, 128.3, 128.2, 126.8, 126.3, 125.7, 121.8, 118.5, 114.9, 102.2, 65.2, 43.8, 37.5, 26.5; HRMS (ESI-TOF) *m*/*z*: [M + H]^+^ calcd. for C_28_H_22_O_4_Cl: 457.1201, Found: 457.1201.

### 3-Nitrobenzyl-(*Z*)-2-(4-Methyl-5-Oxo-4-Phenyl-4,5-Dihydroindeno­[1,2-*b*]­Pyran-2­(3*H*)-Ylidene)­Acetate ((*Z*)-**11ca**)

Purified by silica gel column chromatography eluting with Hexane/EA 20:1 to 10:1; 37% yield (17.2 mg); Yellow oil; ^1^H NMR (400 MHz, CDCl_3_): δ 8.25 (s, 1H), 8.19–8.17 (m, 1H), 7.69 (d, *J =* 7.7 Hz, 1H), 7.55–7.51 (m, 1H), 7.43–7.41 (m, 1H), 7.32–7.28 (m, 6H), 7.20–7.15 (m, 2H), 5.28 (s, 1H), 5.23 (d, *J =* 2.6 Hz, 2H), 2.89 (d, *J =* 14.6 Hz, 1H), 2.74 (d, *J =* 14.8 Hz, 1H), 1.78 (s, 3H); ^13^C­{^1^H } NMR (101 MHz, CDCl_3_): δ 191.5, 169.4, 163.0, 160.7, 148.3, 144.4, 138.1, 136.0, 134.0, 132.5, 132.1, 130.5, 129.6, 128.7, 126.9, 125.7, 123.1, 122.9, 121.9, 118.5, 115.0, 101.8, 64.6, 43.9, 37.6, 26.5; HRMS (ESI-TOF) *m*/*z*: [M + H]^+^ calcd. for C_28_H_22_O_6_N: 468.1442, Found: 468.1442.

### 4-(Trifluoromethyl)­Benzyl-(*E*)-2-(4-Methyl-5-Oxo-4-Phenyl-4,5-Dihydroindeno­[1,2-*b*]­Pyran-2­(3*H*)-Ylidene)­Acetate ((*E*)-**11da**)

Purified by silica gel column chromatography eluting with Hexane/EA 30:1 to 20:1; 16% yield (7.7 mg); Yellow solid; m.p.: 114–115 °C; ^1^H NMR (400 MHz, CDCl_3_): δ 7.63 (d, *J =* 8.0 Hz, 2H), 7.47–7.43 (m, 3H), 7.38–7.33 (m, 4H), 7.25–7.17 (m, 4H), 5.91 (d, *J =* 1.5 Hz, 1H), 5.22 (s, 2H), 4.44 (d, *J =* 15.3 Hz, 1H), 2.73 (dd, *J =* 15.2, 1.7 Hz, 1H), 1.79 (s, 3H); ^13^C­{^1^H }­NMR (101 MHz, CDCl_3_): δ 191.9, 169.1, 165.7, 144.9, 139.9, 136.1, 132.5, 132.1, 130.5, 128.5, 128.2 (q, *J =* 21.7 Hz) 128.0, 126.8 (q, *J =* 286.6 Hz), 126.7, 125.8, 125.5 (q, *J =* 3.8 Hz), 121.9, 118.1, 115.7, 103.7, 65.1, 37.8, 37.6, 27.4; ^19^F NMR (376 MHz, CDCl_3_): δ −62.5 (s, 3F); HRMS (ESI-TOF) *m*/*z*: [M + H]^+^ calcd. for C_29_H_22_O_4_F_3_: 491.1465, Found: 491.1466.

### 4-(Trifluoromethyl)­Benzyl-(*Z*)-2-(4-Methyl-5-Oxo-4-Phenyl-4,5-Dihydroindeno­[1,2-*b*]­Pyran-2­(3*H*)-Ylidene)­Acetate ((*Z*)-**11da**)

Purified by silica gel column chromatography eluting with Hexane/EA 20:1 to 10:1; 60% yield (29.1 mg); Yellow solid; m.p.: 105–106 °C; ^1^H NMR (400 MHz, CDCl_3_): δ 7.64 (d, *J =* 8.0 Hz, 2H), 7.51 (d, *J =* 8.0 Hz, 2H), 7.45–7.43 (m, 1H), 7.32–7.29 (m, 6H), 7.25–7.19 (m, 1H), 7.08–7.06 (m, 1H), 5.29 (d, *J =* 1.4 Hz, 1H), 5.22 (s, 2H), 2.91 (d, *J =* 14.8 Hz, 1H), 2.75 (dd, *J =* 14.8, 1.5 Hz, 1H), 1.79 (s, 3H); ^13^C­{^1^H}­NMR (101 MHz, CDCl_3_): δ 191.5, 169.4, 163.3, 160.3, 144.4, 139.9, 136.0, 132.4, 132.1, 130.5, 130.0 (q, *J =* 33.3 Hz), 128.7, 128.4, 126.9, 126.8 (q, *J =* 292.1 Hz), 125.8, 125.5 (q, *J =* 3.3 Hz), 121.9, 118.5, 115.0, 102.2, 65.2, 43.9, 37.6, 26.5; ^19^F NMR (376 MHz, CDCl_3_): δ −62.4 (s, 3F); HRMS (ESI-TOF) *m*/*z*: [M + H]^+^ calcd. for C_29_H_22_O_4_F_3_: 491.1465, Found: 491.1463.

### 4-Chlorobenzyl-(*E*)-2-(4-Methyl-5-Oxo-4-Phenyl-4,5-Dihydroindeno­[1,2-*b*]­Pyran-2­(3*H*)-Ylidene)­Acetate ((*E*)-**11ea**)

Purified by silica gel column chromatography eluting with Hexane/EA 30:1 to 20:1; 19% yield (8.5 mg); Yellow solid; m.p.: 133 °C; ^1^H NMR (400 MHz, CDCl_3_): δ 7.40–7.38 (m, 1H), 7.30–7.25 (m, 6H), 7.21–7.11 (m, 6H), 5.81 (d, *J =* 1.5 Hz, 1H), 5.06 (s, 2H), 4.36 (d, *J =* 15.3 Hz, 1H), 2.65 (dd, *J =* 15.3, 1.7 Hz, 1H), 1.72 (s, 3H); ^13^C­{^1^H}­NMR (101 MHz, CDCl_3_): δ 191.9, 169.2, 165.8, 165.4, 144.9, 136.1, 134.4, 134.1, 132.4, 132.1, 130.4, 129.5, 128.7, 128.5, 126.7, 125.8, 121.8, 118.1, 115.7, 103.9, 65.2, 37.8, 37.6, 27.4; HRMS (ESI-TOF) *m*/*z*: [M + H]^+^ calcd. for C_28_H_22_O_4_Cl: 457.1201, Found: 457.1202.

### 4-Chlorobenzyl-(*Z*)-2-(4-Methyl-5-Oxo-4-Phenyl-4,5-Dihydroindeno­[1,2-*b*]­Pyran-2­(3*H*)-Ylidene)­Acetate ((*Z*)-**11ea**)

Purified by silica gel column chromatography eluting with Hexane/EA 20:1 to 10:1; 66% yield (29.9 mg); Yellow oil; ^1^H NMR (400 MHz, CDCl_3_): δ 7.44–7.42 (m, 1H), 7.37–7.29 (m, 10H), 7.23–7.21 (m, 1H), 7.04–7.02 (m, 1H), 5.27 (d, *J =* 1.3 Hz, 1H), 5.13 (s, 2H), 2.89 (d, *J =* 14.8 Hz, 1H), 2.73 (dd, *J =* 14.8, 1.5 Hz, 1H), 1.79 (s, 3H); ^13^C­{^1^H } NMR (101 MHz, CDCl_3_): δ 191.5, 169.4, 163.3, 160.0, 144.4, 135.9, 134.4, 134.1, 132.4, 132.1, 130.4, 129.8, 128.7, 128.6, 126.8, 125.7, 121.8, 118.5, 114.8, 102.3, 65.3, 43.8, 37.5, 26.5; HRMS (ESI-TOF) *m*/*z*: [M + H]^+^ calcd. for C_28_H_22_O_4_Cl: 457.1201, Found: 457.1200.

### 4-Bromobenzyl-(*E*)-2-(4-Methyl-5-Oxo-4-Phenyl-4,5-Dihydroindeno­[1,2-*b*]­Pyran-2­(3*H*)-Ylidene)­Acetate ((*E*)-**11fa**)

Purified by silica gel column chromatography eluting with Hexane/EA 30:1 to 20:1; 19% yield (9.7 mg); Yellow solid; m.p.: 139–140 °C; ^1^H NMR (400 MHz, CDCl_3_): δ 7.51–7.45 (m, 3H), 7.37–7.33 (m, 4H), 7.26–7.18 (m, 6H), 5.88 (d, *J =* 1.5 Hz, 1H), 5.11 (s, 2H), 4.43 (d, *J =* 15.3 Hz, 1H), 2.72 (dd, *J =* 15.3, 1.7 Hz, 1H), 1.79 (s, 3H); ^13^C­{^1^H } NMR (101 MHz, CDCl_3_): δ 191.9, 169.2, 165.8, 165.4, 144.9, 136.1, 134.9, 132.4, 132.1, 131.7, 130.4, 129.8, 128.5, 126.7, 125.8, 122.2, 121.8, 118.1, 115.7, 103.9, 65.3, 37.8, 37.6, 27.3; HRMS (ESI-TOF) *m*/*z*: [M + H]^+^ calcd. for C_28_H_22_O_4_Br: 501.0696, Found: 501.0695.

### 4-Bromobenzyl-(*Z*)-2-(4-Methyl-5-Oxo-4-Phenyl-4,5-Dihydroindeno­[1,2-*b*]­Pyran-2­(3*H*)-Ylidene)­Acetate ((*Z*)-**11fa**)

Purified by silica gel column chromatography eluting with Hexane/EA 20:1 to 10:1; 60% yield (30.2 mg); Yellow solid; m.p.: 128–129 °C; ^1^H NMR (400 MHz, CDCl_3_): δ 7.46 (d, *J =* 8.2 Hz, 2H), 7.40–7.38 (m, 1H), 7.32–7.22 (m, 8H), 7.18–7.14 (m, 1H), 6.98 (dd, *J =* 6.1, 1.8 Hz, 1H), 5.22 (d, *J =* 1.3 Hz, 1H), 5.07 (s, 2H), 2.85 (d, *J =* 14.7 Hz, 1H), 2.68 (dd, *J =* 14.8, 1.6 Hz, 1H), 1.74 (s, 3H); ^13^C­{^1^H } NMR (101 MHz, CDCl_3_): δ 191.5, 169.4, 163.3, 160.0, 144.4, 136.0, 134.9, 132.4, 132.1, 131.7, 130.4, 130.1, 128.6, 126.8, 125.7, 122.2, 121.8, 118.5, 114.9, 102.3, 65.4, 43.8, 37.5, 26.5; HRMS (ESI-TOF) *m*/*z*: [M + H]^+^ calcd. for C_28_H_22_O_4_Br: 501.0696, Found: 501.0695.

### 4-Methylbenzyl-(*E*)-2-(4-Methyl-5-Oxo-4-Phenyl-4,5-Dihydroindeno­[1,2-*b*]­Pyran-2­(3*H*)-Ylidene)­Acetate ((*E*)-**11ga**)

Purified by silica gel column chromatography eluting with Hexane/EA 30:1 to 20:1; 20% yield (8.6 mg); Yellow oil; ^1^H NMR (400 MHz, CDCl_3_): δ 7.47–7.45 (m, 1H), 7.37–7.32 (m, 4H), 7.24–7.17 (m, 8H), 5.88 (d, *J =* 1.5 Hz, 1H), 5.14 (s, 2H), 4.45 (d, *J =* 15.3 Hz, 1H), 2.73 (dd, *J =* 15.3, 1.7 Hz, 1H), 2.37 (s, 3H), 1.79 (s, 3H); ^13^C­{^1^H } NMR (101 MHz, CDCl_3_): δ 192.0, 169.2, 165.9, 165.0, 145.0, 138.1, 136.2, 132.8, 132.4, 132.1, 130.4, 129.2, 128.5, 128.3, 126.6, 125.9, 121.8, 118.1, 115.7, 104.3, 66.1, 37.8, 37.5, 27.4, 21.2; HRMS (ESI-TOF) *m*/*z*: [M + H]^+^ calcd. for C_29_H_25_O_4_: 437.1747, Found: 437.1749.

### 4-Methylbenzyl-(*Z*)-2-(4-Methyl-5-Oxo-4-Phenyl-4,5-Dihydroindeno­[1,2-*b*]­Pyran-2­(3*H*)-Ylidene)­Acetate ((*Z*)-**11ga**)

Purified by silica gel column chromatography eluting with Hexane/EA 20:1 to 10:1; 48% yield (20.8 mg); Yellow oil; ^1^H NMR (400 MHz, CDCl_3_): δ 7.44–7.41 (m, 1H), 7.33–7.28 (m, 8H), 7.23–7.19 (m, 3H), 7.11–7.05 (m, 1H), 5.27 (d, *J =* 1.4 Hz, 1H), 5.14 (s, 2H), 2.89 (d, *J =* 14.8 Hz, 1H), 2.72 (dd, *J =* 14.8, 1.5 Hz, 1H), 2.39 (s, 3H), 1.79 (s, 3H); ^13^C­{^1^H } NMR (101 MHz, CDCl_3_): δ 191.6, 169.5, 163.6, 159.6, 144.5, 138.1, 136.0, 132.8, 132.4, 132.2, 130.3, 129.2, 128.6, 126.8, 125.8, 121.7, 118.7, 114.8, 102.7, 66.2, 43.8, 37.5, 26.5, 21.2; HRMS (ESI-TOF) *m*/*z*: [M + H]^+^ calcd. for C_29_H_25_O_4_: 437.1747, Found: 437.1747.

### 4-Methoxybenzyl-(*E*)-2-(4-Methyl-5-Oxo-4-Phenyl-4,5-Dihydroindeno­[1,2-*b*]­Pyran-2­(3*H*)-Ylidene)­Acetate ((*E*)-**11ha**)

Purified by silica gel column chromatography eluting with Hexane/EA 30:1 to 20:1; 25% yield (11.4 mg); Yellow solid; m.p.: 123–124 °C; ^1^H NMR (400 MHz, CDCl_3_): δ 7.46 (d, *J =* 6.9 Hz, 1H), 7.38–7.15 (m, 10H), 6.90 (d, *J =* 8.1 Hz, 2H), 5.87 (d, *J =* 1.7 Hz, 1H), 5.13 (d, *J =* 12.4 Hz, 1H), 5.09 (d, *J =* 12.2 Hz, 1H), 4.44 (d, *J =* 15.2 Hz, 1H), 3.82 (s, 3H), 2.73 (dd, *J =* 15.1, 1.8 Hz, 1H), 1.79 (s, 3H); ^13^C­{^1^H} NMR (101 MHz, CDCl_3_): δ 191.9, 169.2, 165.9, 164.9, 159.6, 145.0, 136.1, 132.4, 132.1, 130.4, 130.0, 129.5, 128.5, 127.9, 126.6, 125.9, 121.8, 118.1, 115.7, 113.9, 104.3, 66.0, 55.3, 37.8, 37.5, 27.4; HRMS (ESI-TOF) *m*/*z*: [M + H]^+^ calcd. for C_29_H_25_O_5_: 453.1697, Found: 453.1698.

### 4-Methoxybenzyl-(*Z*)-2-(4-Methyl-5-Oxo-4-Phenyl-4,5-Dihydroindeno­[1,2-*b*]­Pyran-2­(3*H*)-Ylidene)­Acetate ((*Z*)-**11ha**)

Purified by silica gel column chromatography eluting with Hexane/EA 20:1 to 10:1; 58% yield (26.3 mg); Yellow oil; ^1^H NMR (400 MHz, CDCl_3_): δ 7.39–7.37 (m, 1H), 7.32–7.22 (m, 8H), 7.18–7.14 (m, 1H), 7.02–6.97 (m, 1H), 6.89–6.85 (m, 2H), 5.21 (d, *J =* 1.3 Hz, 1H), 5.07 (s, 2H), 3.78 (s, 3H), 2.84 (d, *J =* 14.8 Hz, 1H), 2.67 (dd, *J =* 14.8, 1.5 Hz, 1H), 1.73 (s, 3H); ^13^C­{^1^H } NMR (101 MHz, CDCl_3_): δ 191.6, 169.5, 163.6, 159.6, 159.6, 144.5, 136.0, 132.4, 132.1, 130.34, 130.29, 128.6, 127.9, 126.8, 125.7, 121.7, 118.6, 114.8, 113.9, 102.7, 66.0, 55.2, 43.7, 37.5, 26.5; HRMS (ESI-TOF) *m*/*z*: [M + H]^+^ calcd. for C_29_H_25_O_5_: 453.1697, Found: 453.1697.

### Naphthalen-2-Ylmethyl-(*E*)-2-(4-Methyl-5-Oxo-4-Phenyl-4,5-Dihydroindeno­[1,2-*b*]­Pyran-2­(3*H*)-Ylidene)­Acetate ((*E*)-**11ia**)

Purified by silica gel column chromatography eluting with Hexane/EA 30:1 to 20:1; 24% yield (11.1 mg); Yellow oil; ^1^H NMR (400 MHz, CDCl_3_): δ 7.87–7.83 (m, 4H), 7.52–7.44 (m, 4H), 7.39–7.30 (m, 4H), 7.23–7.14 (m, 4H), 5.94 (d, *J =* 1.5 Hz, 1H), 5.35 (s, 2H), 4.47 (d, *J =* 15.2 Hz, 1H), 2.74 (dd, *J =* 15.3, 1.7 Hz, 1H), 1.80 (s, 3H); ^13^C­{^1^H } NMR (101 MHz, CDCl_3_): δ 192.0, 169.2, 165.9, 165.2, 145.0, 136.1, 133.2, 133.1, 133.1, 132.4, 132.1, 130.4, 128.5, 128.4, 127.9, 127.7, 127.2, 126.6, 126.31, 126.28, 125.8, 125.7, 121.8, 118.1, 115.7, 104.1, 66.3, 37.8, 37.5, 27.4; HRMS (ESI-TOF)*m*/*z*: [M + H]^+^ calcd. for C_32_H_25_O_4_: 473.1747, Found: 473.1751.

### Naphthalen-2-Ylmethyl-(*Z*)-2-(4-Methyl-5-Oxo-4-Phenyl-4,5-Dihydroindeno­[1,2-*b*]­Pyran-2­(3*H*)-Ylidene)­Acetate ((*Z*)-**11ia**)

Purified by silica gel column chromatography eluting with Hexane/EA 20:1 to 10:1; 61% yield (28.9 mg); Yellow oil; ^1^H NMR (400 MHz, CDCl_3_): δ 7.89–7.83 (m, 4H), 7.55–7.50 (m, 3H), 7.40 (dt, *J =* 7.1, 0.9 Hz, 1H), 7.39–7.28 (m, 4H), 7.24–7.19 (m, 2H), 7.02 (ddd, *J =* 7.8, 7.1, 1.1 Hz, 1H), 6.87 (dt, *J =* 7.2, 0.9 Hz, 1H), 5.35 (s, 2H), 5.31 (d, *J =* 1.3 Hz, 1H), 2.89 (d, *J =* 14.8 Hz, 1H), 2.71 (dd, *J =* 14.8, 1.5 Hz, 1H), 1.78 (s, 3H); ^13^C­{^1^H } NMR (101 MHz, CDCl_3_): δ 191.6, 169.4, 163.6, 159.8, 144.5, 135.9, 133.2, 133.11, 133.06, 132.3, 132.0, 130.3, 128.6, 128.4, 127.9, 127.7, 127.6, 126.8, 126.3, 126.2, 125.7, 121.6, 118.5, 114.7, 102.6, 66.4, 43.7, 37.5, 26.5; HRMS (ESI-TOF) *m*/*z*: [M + H]^+^ calcd. for C_32_H_25_O_4_: 473.1747, Found: 473.1750.

### Ethyl-(*E*)-2-(4-Methyl-5-Oxo-4-Phenyl-4,5-Dihydroindeno­[1,2-*b*]­Pyran-2­(3*H*)-Ylidene)­Acetate ((*E*)-**11ja**)

Purified by silica gel column chromatography eluting with Hexane/EA 30:1 to 20:1; 25% yield (9.0 mg); Yellow oil; ^1^H NMR (400 MHz, CDCl_3_): δ 7.47–7.45 (m, 1H), 7.40–7.24 (m, 6H), 7.21–7.15 (m, 2H), 5.84 (d, *J =* 1.6 Hz, 1H), 4.43 (d, *J =* 15.3 Hz, 1H), 4.18 (qd, *J =* 7.1, 1.7 Hz, 2H), 2.73 (dd, *J =* 15.2, 1.7 Hz, 1H), 1.79 (s, 3H), 1.28 (t, *J =* 7.1 Hz, 3H); ^13^C­{^1^H } NMR (101 MHz, CDCl_3_): δ 192.0, 169.3, 166.1, 164.6, 145.1, 136.2, 132.4, 132.1, 130.4, 128.5, 126.6, 125.9, 121.8, 118.1, 115.7, 104.5, 60.3, 37.8, 37.5, 27.4, 14.2.; HRMS (ESI-TOF) *m*/*z*: [M + H]^+^ calcd. for C_23_H_21_O_4_: 361.1434, Found: 361.1433.

### Ethyl-(*Z*)-2-(4-Methyl-5-Oxo-4-Phenyl-4,5-Dihydroindeno­[1,2-*b*]­Pyran-2­(3*H*)-Ylidene)­Acetate ((*Z*)-**11ja**)

Purified by silica gel column chromatography eluting with Hexane/EA 20:1 to 10:1; 56% yield­(20.0 mg); Yellow solid; m.p.: 130–131 °C; ^1^H NMR (400 MHz, CDCl_3_): δ 7.45–7.27 (m, 8H), 7.23–7.19 (m, 1H), 5.22 (s, 1H), 4.19 (q, *J =* 7.1 Hz, 2H), 2.88 (d, *J =* 14.7 Hz, 1H), 2.72 (dd, *J =* 14.7, 1.1 Hz, 1H), 1.79 (s, 3H), 1.31 (t, *J =* 7.1 Hz, 3H); ^13^C­{^1^H } NMR (101 MHz, CDCl_3_): δ 191.6, 169.6, 163.7, 159.3, 144.6, 136.2, 132.5, 132.2, 130.4, 128.6, 126.8, 125.8, 121.8, 118.6, 114.9, 102.9, 60.2, 43.9, 37.6, 26.5, 14.3; HRMS (ESI-TOF) *m*/*z*: [M + H]^+^ calcd. for C_23_H_21_O_4_: 361.1434, Found: 361.1434.

### *tert*-Butyl-(*Z*)-2-(4-Methyl-5-Oxo-4-Phenyl-4,5-Dihydroindeno­[1,2-*b*]­Pyran-2­(3*H*)-Ylidene)­Acetate ((*Z*)-**11ka**)

Purified by silica gel column chromatography eluting with Hexane/EA 20:1 to 10:1; 54% yield (20.8 mg); Yellow solid; m.p.: 182–183 °C; ^1^H NMR (400 MHz, CDCl_3_): δ 7.40–7.22 (m, 8H), 7.19–7.15 (m, 1H), 5.10 (d, *J =* 1.4 Hz, 1H), 2.81 (d, *J =* 14.7 Hz, 1H), 2.64 (dd, *J =* 14.7, 1.4 Hz, 1H), 1.73 (s, 3H), 1.47 (s, 9H); ^13^C­{^1^H } NMR (101 MHz, CDCl_3_): δ 191.7, 169.8, 163.0, 158.1, 144.7, 136.3, 132.4, 132.3, 130.4, 128.6, 126.7, 125.9, 121.7, 118.6, 114.9, 104.8, 80.6, 43.9, 37.5, 28.2, 26.6; HRMS (ESI-TOF) *m*/*z*: [M + H]^+^ calcd. for C_25_H_25_O_4_: 389.1747, Found: 389.1742.

### Benzyl-2-(4,4-Dimethyl-5-Oxo-4,5-Dihydroindeno­[1,2-*b*]­Pyran-2-yl)­Acetate (**12a**)

Purified by silica gel column chromatography eluting with Hexane/EA 20:1 to 10:1; 71% yield (25.4 mg); Yellow solid; m.p.: 114–115 °C; ^1^H NMR (400 MHz, CDCl_3_): δ 7.42–7.31 (m, 6H), 7.25–7.20 (m, 2H), 6.99–6.94 (m, 1H), 5.36 (s, 1H), 5.19 (s, 2H), 2.37 (s, 2H), 1.29 (s, 6H). ^13^C­{^1^H } NMR (101 MHz, CDCl_3_): δ 191.6, 168.5, 163.5, 160.4, 136.1, 135.8, 132.3, 132.2, 129.9, 128.5, 128.4, 128.2, 128.1, 121.3, 118.2, 116.1, 102.5, 66.1, 43.3, 29.9, 26.6; HRMS (ESI-TOF) *m*/*z*: [M + Na]^+^ calcd. for C_23_H_20_O_4_Na: 383.1254, Found: 383.1258.

### Benzyl-2-(5′-Oxo-5′*H*-Spiro­[Cyclohexane-1,4′-Indeno­[1,2-*b*]­Pyran]-2′-yl)­Acetate (**12c**)

Purified by silica gel column chromatography eluting with Hexane/EA 30:1 to 20:1; 77% yield (30.7 mg); Yellow oil; ^1^H NMR (400 MHz, CDCl_3_): δ 7.44–7.37 (m, 6H), 7.26–7.23 (m, 2H), 6.99–6.97 (m, 1H), 5.37 (s, 1H), 5.21 (s, 2H), 2.62 (s, 2H), 2.14 (td, *J =* 13.0, 3.9 Hz, 2H), 1.70–1.62 (m, 4H), 1.46–1.37 (m, 4H); ^13^C­{^1^H } NMR (101 MHz, CDCl_3_): δ 191.8, 168.3, 163.7, 160.8, 136.1, 135.9, 132.3, 132.2, 130.0, 128.6, 128.5, 128.2, 121.4, 118.2, 118.1, 102.3, 66.2, 41.0, 38.2, 36.8, 30.3, 23.2; HRMS (ESI-TOF) *m*/*z*: [M + Na]^+^ calcd. for C_26_H_24_O_4_Na: 423.1567, Found: 423.1566.

### Benzyl-2-(5′-Oxo-5′*H*-Spiro­[Cycloheptane-1,4′-Indeno­[1,2-*b*]­Pyran]-2′-yl)­Acetate (**12d**)

Purified by silica gel column chromatography eluting with Hexane/EA 20:1 to 10:1; 71% yield (29.2 mg); Yellow oil; ^1^H NMR (400 MHz, CDCl_3_): δ 7.44–7.35 (m, 6H), 7.26–7.24 (m, 2H), 6.98–6.96 (m, 1H), 5.36 (s, 1H), 5.21 (s, 2H), 2.47 (s, 2H), 2.12 (ddd, *J =* 14.8, 10.5, 1.5 Hz, 2H), 1.81–1.77 (m, 2H), 1.66–1.45 (m, 8H); ^13^C­{^1^H } NMR (101 MHz, CDCl_3_): δ 191.4, 169.2, 163.5, 159.8, 136.6, 136.0, 135.9, 133.4, 132.4, 132.3, 130.3, 129.0, 128.6, 128.6, 128.5, 128.2, 127.5, 126.4, 121.7, 118.6, 113.6, 103.0, 66.3, 42.0, 35.6, 24.9; HRMS (ESI-TOF) *m*/*z*: [M + Na]^+^ calcd. for C_27_H_26_O_4_Na: 437.1723, Found: 437.1722.

### Benzyl-2-(1′,3′-Dioxo-2-Phenyl-1′,3′-Dihydrospiro­[Cyclopentane-1,2′-Inden]-2-en-5-yl)­Acetate (**13aa**)

Purified by silica gel column chromatography eluting with Hexane/THF 12:1 to 8:1; 50% yield (21.5 mg); Yellow oil; ^1^H NMR (400 MHz,CDCl_3_): δ 7.97–7.93 (m, 2H), 7.85–7.80 (m, 2H), 7.34–7.29 (m, 3H), 7.20–7.17 (m, 2H), 7.11–7.03 (m, 3H), 6.93–6.90 (m, 2H), 6.42 (t, *J =* 2.5 Hz, 1H), 4.81 (s, 2H), 3.47 – 3.43 (m, 1H), 2.93 (ddd, *J =* 16.8, 8.3, 2.8 Hz, 1H), 2.60–2.46 (m, 3H); ^13^C­{^1^H } NMR (101 MHz, CDCl_3_): δ 202.3, 201.1, 171.3, 142.8, 142.1, 141.7, 135.8, 135.7, 135.3, 134.6, 134.1, 128.4, 128.23, 128.18, 128.1, 127.5, 126.3, 123.3, 71.0, 66.2, 44.6, 38.4, 35.4; HRMS (ESI-TOF) *m*/*z*: [M + H]^+^ calcd. for C_28_H_23_O_4_: 423.1591, Found: 423.1574.

### Benzyl-2-(2-(4-Fluorophenyl)-1′,3′-Dioxo-1′,3′-Dihydrospiro­[Cyclopentane-1,2′-Inden]-2-en-5-yl)­Acetate (**13ab**)

Purified by silica gel column chromatography eluting with Hexane/THF 12:1 to 8:1; 40% yield (17.6 mg); Yellow oil; ^1^H NMR (400 MHz, CDCl_3_): δ 7.96–7.92 (m, 2H), 7.85–7.80 (m, 2H), 7.35–7.29 (m, 3H), 7.19–7.16 (m, 2H), 6.91–6.87 (m, 2H), 6.77–6.73 (m, 2H), 6.34 (t, *J* = 2.5 Hz, 1H), 4.80 (s, 2H), 3.49–3.41 (m, 1H), 2.92 (ddd, *J* = 16.9, 8.4, 2.8 Hz, 1H), 2.60–2.47 (m, 3H); ^13^C­{^1^H } NMR (101 MHz, CDCl_3_): δ 202.3, 201.2, 171.4, 162.1 (d, *J* = 247.1 Hz), 142.8, 141.8, 141.2, 136.0, 135.9, 135.3, 134.4, 130.9 (d, *J* = 3.3 Hz), 128.5, 128.3, 128.22, 128.15, 123.3, 115.2 (d, *J* = 21.6 Hz), 71.2, 66.3, 44.5, 38.4, 35.4; ^19^F NMR (376 MHz, CDCl_3_): δ −114.1 (s, 1F); HRMS (ESI-TOF) *m*/*z*: [M + H]^+^ calcd. for C_28_H_22_O_4_F: 441.1497, Found: 441.1485.

### Benzyl-2-(2-(4-Chlorophenyl)-1′,3′-Dioxo-1′,3′-Dihydrospiro­[Cyclopentane-1,2′-Inden]-2-en-5-yl)­Acetate (**13ac**)

Purified by silica gel column chromatography eluting with Hexane/THF 12:1 to 8:1; 36% yield (16.6 mg); Yellow oil; ^1^H NMR (400 MHz, CDCl_3_): δ 7.97–7.93 (m, 2H), 7.86–7.80 (m, 2H), 7.35–7.29 (m, 3H), 7.17–7.16 (m, 2H), 7.03 (d, *J =* 8.3 Hz, 2H), 6.85 (d, *J =* 8.3 Hz, 2H), 6.40 (t, *J =* 2.5 Hz, 1H), 4.81 (s, 2H), 3.48–3.40 (m, 1H), 2.93 (ddd, *J =* 17.0, 8.4, 2.8 Hz, 1H), 2.60–2.46 (m, 3H); ^13^C­{^1^H } NMR (101 MHz, CDCl_3_): δ 202.1, 201.0, 171.3, 142.8, 141.8, 141.1, 136.0, 135.9, 135.3, 135.0, 133.4, 133.2, 128.5, 128.3, 128.2, 127.7, 123.4, 71.0, 66.3, 44.7, 38.5, 35.4; HRMS (ESI-TOF) *m*/*z*: [M + H]^+^ calcd. for C_28_H_22_O_4_Cl: 457.1201, Found: 457.1189.

### Benzyl-2-(2-(4-Bromophenyl)-1′,3′-Dioxo-1′,3′-Dihydrospiro­[Cyclopentane-1,2′-Inden]-2-en-5-yl)­Acetate (**13ad**)

Purified by silica gel column chromatography eluting with Hexane/THF 12:1 to 8:1; 28% yield (14.1 mg); Yellow oil; ^1^H NMR (400 MHz, CDCl_3_): δ 7.97–7.93 (m, 2H), 7.86–7.80 (m, 2H), 7.33–7.28 (m, 3H), 7.21–7.16 (m, 4H), 6.8 (dt, *J* = 8.6, 2.5 Hz, 2H), 6.41 (t, *J =* 2.5 Hz, 1H), 4.81 (s, 2H), 3.48–3.40 (m, 1H), 2.92 (ddd, *J =* 16.9, 8.3, 2.9 Hz, 1H), 2.59–2.46 (m, 3H); ^13^C­{^1^H } NMR (101 MHz, CDCl_3_): δ 202.1, 201.0, 171.3, 142.8, 141.7, 141.1, 136.0, 135.9, 135.3, 135.1, 133.7, 131.4, 128.5, 128.2, 123.4, 121.6, 71.0, 66.3, 44.7, 38.5, 35.4; HRMS (ESI-TOF) *m*/*z*: [M + H]^+^ calcd. for C_28_H_22_O_4_Br: 501.0696, Found: 501.0684.

### Benzyl-2-(1′,3′-Dioxo-2-(*p*-Tolyl)-1′,3′-Dihydrospiro­[Cyclopentane-1,2′-Inden]-2-en-5-yl)­Acetate (**13ae**)

Purified by silica gel column chromatography eluting with Hexane/THF 12:1 to 8:1; 55% yield (24.1 mg); Yellow oil; ^1^H NMR (400 MHz, CDCl_3_): δ 7.97–7.94 (m, 2H), 7.84–7.82 (m, 2H), 7.11–7.03 (m, 7H), 6.93–6.90 (m, 2H), 6.42 (t, *J =* 2.6 Hz, 1H), 4.77 (s, 2H), 3.48–3.40 (m, 1H), 2.92 (ddd, *J =* 16.9, 8.3, 2.9 Hz, 1H), 2.60–2.43 (m, 3H), 2.33 (s, 3H); ^13^C­{^1^H } NMR (101 MHz, CDCl_3_): δ 202.6, 201.5, 171.5, 143.0, 142.3, 142.0, 137.5, 136.0, 135.9, 135.5, 133.5, 131.9, 129.1, 128.6, 128.4, 128.3, 126.4, 123.5, 123.4, 71.1, 66.4, 44.9, 38.5, 35.6, 21.1; HRMS (ESI-TOF) *m*/*z*: [M + H]^+^ calcd. for C_29_H_25_O_4_: 437.1747, Found: 437.1736.

### Benzyl-2-(2-(4-Methoxyphenyl)-1′,3′-Dioxo-1′,3′-Dihydrospiro­[Cyclopentane-1,2′-Inden]-2-en-5-yl)­Acetate (**13af**)

Purified by silica gel column chromatography eluting with Hexane/THF 12:1 to 8:1; 33% yield (14.9 mg); Yellow oil; ^1^H NMR (400 MHz, CDCl_3_): δ 7.97–7.93 (m, 2H), 7.83–7.81 (m, 2H), 7.33–7.28 (m, 3H), 7.19–7.17 (m, 2H), 6.88–6.83 (m, 2H), 6.61–6.57 (m, 2H), 6.32 (t, *J* = 2.5 Hz, 1H), 4.81 (d, *J* = 1.7 Hz, 2H), 3.66 (s, 3H), 3.48–3.40 (m, 1H), 2.91 (ddd, *J* = 16.7, 8.3, 2.8 Hz, 1H), 2.60–2.45 (m, 3H); ^13^C­{^1^H } NMR (101 MHz, CDCl_3_): δ 202.6, 201.4, 171.4, 159.0, 142.9, 141.8, 141.7, 135.8, 135.7, 135.4, 132.7, 128.5, 128.23, 128.15, 127.6, 127.3, 123.3, 113.7, 71.1, 66.2, 55.1, 44.7, 38.4, 35.5; HRMS (ESI-TOF) *m*/*z*: [M + H]^+^ calcd. for C_29_H_25_O_5_: 453.1697, Found: 453.1688.

### Benzyl-2-(2-(Naphthalen-2-yl)-1′,3′-Dioxo-1′,3′-Dihydrospiro­[Cyclopentane-1,2′-Inden]-2-en-5-yl)­Acetate (**13ag**)

Purified by silica gel column chromatography eluting with Hexane/THF 12:1 to 8:1; 47% yield (22.0 mg); Yellow oil; ^1^H NMR (400 MHz, CDCl_3_): δ 7.99–7.97 (m, 2H), 7.84–7.82 (m, 2H), 7.66 (d, *J =* 6.8 Hz, 1H), 7.58 (d, *J =* 9.1 Hz, 1H), 7.42–7.30 (m, 6H), 7.22–7.19 (m, 4H), 6.56 (t, *J =* 2.5 Hz, 1H), 4.84 (s, 2H), 3.57–3.49 (m, 1H), 3.00 (ddd, *J =* 16.9, 8.4, 2.8 Hz, 1H), 2.67–2.52 (m, 3H); ^13^C­{^1^H } NMR (101 MHz, CDCl_3_): δ 202.5, 201.4, 171.4, 143.0, 142.2, 141.8, 136.0, 135.8, 135.4, 134.9, 132.9, 132.6, 132.2, 128.5, 128.3, 128.2, 128.0, 127.9, 127.4, 126.1, 126.0, 125.5, 125.3, 124.7, 123.4, 123.4, 71.1, 66.3, 44.7, 38.6, 35.5, 30.3; HRMS (ESI-TOF) *m*/*z*: [M + H]^+^ calcd. for C_32_H_25_O_4_: 473.1747, Found: 473.1755.

### Benzyl-2-(1′,3′-Dioxo-2-(Thiophen-2-yl)-1′,3′-Dihydrospiro­[Cyclopentane-1,2′-Inden]-2-en-5-yl)­Acetate (**13ah**)

Purified by silica gel column chromatography eluting with Hexane/THF 12:1 to 8:1; 50% yield (21.3 mg); Yellow oil; ^1^H NMR (400 MHz, CDCl_3_): δ 8.03 (d, *J* = 6.8 Hz, 1H), 7.95 (d, *J* = 6.9 Hz, 1H), 7.89–7.82 (m, 2H), 7.33–7.29 (m, 3H), 7.19–7.17 (m, 2H), 6.99 (d, *J =* 5.1 Hz, 1H), 6.64 (t, *J* = 4.1 Hz, 1H), 6.45 (t, *J =* 2.7 Hz, 1H), 6.24 (d, *J =* 3.6 Hz, 1H), 4.81 (s, 2H), 3.50–3.42 (m, 1H), 2.94 (ddd, *J =* 17.2, 8.4, 2.9 Hz, 1H), 2.60–2.46 (m, 3H); ^13^C­{^1^H } NMR (101 MHz, CDCl_3_): δ 202.1, 200.9, 171.3, 143.0, 141.9, 137.5, 136.0, 135.9, 135.3, 135.2, 133.6, 128.5, 128.2, 128.1, 127.0, 124.6, 123.8, 123.4, 123.3, 70.6, 66.3, 44.5, 38.5, 35.4; HRMS (ESI-TOF) *m*/*z*: [M + H]^+^ calcd. for C_26_H_21_O_4_S: 429.1155, Found: 429.1147.

### 3-Chlorobenzyl-2-(1′,3′-Dioxo-2-Phenyl-1′,3′-Dihydrospiro­[Cyclopentane-1,2′-Inden]-2-en-5-yl)­Acetate (**13ba**)

Purified by silica gel column chromatography eluting with Hexane/THF 12:1 to 8:1; 31% yield (14.3 mg); Yellow oil;^1^H NMR (400 MHz, CDCl_3_): δ 7.95–7.93 (m, 2H), 7.84–7.80 (m, 2H), 7.29–7.22 (m, 3H), 7.15–7.03 (m, 5H), 6.92–6.90 (m, 2H), 6.41 (t, *J =* 2.5 Hz, 1H), 4.77 (s, 2H), 3.47 – 3.41 (m, 1H), 2.93 (ddd, *J =* 16.8, 8.4, 2.9 Hz, 1H), 2.62–2.50 (m, 3H); ^13^C­{^1^H } NMR (101 MHz, CDCl_3_): δ 202.3, 201.2, 171.3, 142.8, 142.3, 141.8, 137.4, 135.9, 135.8, 134.6, 134.3, 134.1, 129.8, 128.5, 128.4, 128.3, 128.1, 127.6, 126.4, 126.2, 126.1, 123.3, 71.0, 65.3, 44.5, 38.5, 35.4; HRMS (ESI-TOF) *m*/*z*: [M + H]^+^ calcd. for C_28_H_22_O_4_Cl: 457.1201, Found: 457.1183.

### 3-Nitrobenzyl-2-(1′,3′-Dioxo-2-Phenyl-1′,3′-Dihydrospiro­[Cyclopentane-1,2′-Inden]-2-en-5-yl)­Acetate (**13ca**)

Purified by silica gel column chromatography eluting with Hexane/THF 12:1 to 8:1; 30% yield (14.1 mg); Yellow oil; ^1^H NMR (400 MHz, CDCl_3_): δ 8.16 (dt, *J =* 7.6, 2.1 Hz, 1H), 8.02 (s, 1H), 7.95–7.91 (m, 2H), 7.84–7.79 (m, 2H), 7.54–7.48 (m, 2H), 7.08–7.03 (m, 3H), 6.91–6.89 (m, 2H), 6.40 (t, *J =* 2.5 Hz, 1H), 4.89 (s, 2H), 3.48–3.41 (m, 1H), 2.94 (ddd, *J =* 16.8, 8.4, 2.8 Hz, 1H), 2.61–2.54 (m, 3H); ^13^C­{^1^H } NMR (101 MHz, CDCl_3_): δ 202.3, 201.2, 171.3, 142.8, 142.3, 141.8, 137.4, 135.9, 135.8, 134.6, 134.1, 134.0, 129.6, 128.3, 127.6, 126.4, 123.3, 123.2, 122.9, 71.0, 64.8, 44.3, 38.4, 35.3; HRMS (ESI-TOF) *m*/*z*: [M + H]^+^ calcd. for C_28_H_22_O_6_N: 468.1442, Found: 468.1425.

### 4-(Trifluoromethyl)­Benzyl-2-(1′,3′-Dioxo-2-Phenyl-1′,3′-Dihydrospiro­[Cyclopentane-1,2′-Inden]-2-en-5-yl)­Acetate (**13da**)

Purified by silica gel column chromatography eluting with Hexane/THF 12:1 to 8:1; 43% yield (21.1 mg); Yellow oil; ^1^H NMR (400 MHz, CDCl_3_): δ 7.94–7.88 (m, 2H), 7.82–7.77 (m, 2H), 7.56 (d, *J =* 8.0 Hz, 2H), 7.29 (d, *J =* 8.0 Hz, 2H), 7.10–7.03 (m, 3H), 6.92–6.89 (m, 2H), 6.40 (t, *J =* 2.5 Hz, 1H), 4.86 (s, 2H), 3.50 – 3.42 (m, 1H), 2.94 (ddd, *J =* 16.8, 8.4, 2.8 Hz, 1H), 2.62–2.53 (m, 3H); ^13^C­{^1^H } NMR (101 MHz, CDCl_3_): δ 202.3, 201.3, 171.3, 142.8, 142.3, 141.8, 139.4, 135.9, 135.7, 134.6, 134.1, 130.3 (q, *J =* 32.9 Hz), 128.3, 128.1, 127.6, 126.6 (q, *J =* 274.6 Hz), 126.4, 125.5 (q, *J =* 3.6 Hz), 123.3, 71.0, 65.2, 44.4, 38.5, 35.3. ^19^F NMR (376 MHz, CDCl_3_): δ −62.5 (s, 3F); HRMS (ESI-TOF) *m*/*z*: [M + H]^+^ calcd. for C_29_H_22_F_3_O_4_: 491.1465, Found: 491.1445.

### 4-Chlorobenzyl-2-(1′,3′-Dioxo-2-Phenyl-1′,3′-Dihydrospiro­[Cyclopentane-1,2′-Inden]-2-en-5-yl)­Acetate (**13ea**)

Purified by silica gel column chromatography eluting with Hexane/THF 12:1 to 8:1; 54% yield (24.9 mg); Yellow oil; ^1^H NMR (400 MHz, CDCl_3_): δ 7.95–7.90 (m, 2H), 7.84–7.79 (m, 2H), 7.27 (d, *J* = 8.5 Hz, 2H), 7.12–7.03 (m, 5H), 6.91 (dd, *J* = 7.8, 1.8 Hz, 2H), 6.40 (t, *J =* 2.6 Hz, 1H), 4.77 (s, 2H), 3.49–3.41 (m, 1H), 2.92 (ddd, *J =* 16.8, 8.3, 2.8 Hz, 1H), 2.61–2.49 (m, 3H); ^13^C­{^1^H } NMR (101 MHz, CDCl_3_): δ 202.3, 201.2, 171.3, 142.8, 142.2, 141.7, 135.9, 135.7, 134.6, 134.13, 134.05, 133.9, 129.5, 128.6, 128.3, 127.6, 126.3, 123.3, 70.9, 65.4, 44.5, 38.4, 35.3; HRMS (ESI-TOF) *m*/*z*: [M + H]^+^ calcd. for C_28_H_22_O_4_Cl: 457.1201, Found: 457.1208.

### 4-Bromobenzyl-2-(1′,3′-Dioxo-2-Phenyl-1′,3′-Dihydrospiro­[Cyclopentane-1,2′-Inden]-2-en-5-yl)­Acetate (**13fa**)

Purified by silica gel column chromatography eluting with Hexane/THF 12:1 to 8:1; 62% yield (31.4 mg); Yellow oil; ^1^H NMR (400 MHz, CDCl_3_): δ 7.94–7.90 (m, 2H), 7.83–7.80 (m, 2H), 7.44–7.41 (m, 2H), 7.08–7.03 (m, 5H), 6.92–6.89 (m, 2H), 6.40 (t, *J =* 2.3 Hz, 1H), 4.75 (s, 2H), 3.48–3.40 (m, 1H), 2.92 (ddd, *J =* 16.8, 8.4, 2.6 Hz, 1H), 2.61–2.48 (m, 3H); ^13^C­{^1^H } NMR (101 MHz, CDCl_3_): δ 202.3, 201.2, 171.3, 142.8, 142.3, 141.7, 135.8, 135.7, 134.6, 134.4, 134.1, 131.6, 129.8, 128.3, 127.6, 126.4, 123.3, 122.2, 71.0, 65.4, 44.4, 38.4, 35.4; HRMS (ESI-TOF) *m*/*z*: [M + H]^+^ calcd. for C_28_H_22_O_4_Br: 501.0696, Found: 501.0703.

### 4-Methylbenzyl-2-(1′,3′-Dioxo-2-Phenyl-1′,3′-Dihydrospiro­[Cyclopentane-1,2′-Inden]-2-en-5-yl)­Acetate (**13ga**)

Purified by silica gel column chromatography eluting with Hexane/THF 12:1 to 8:1; 43% yield (18.8 mg); Yellow oil;^1^H NMR (400 MHz, CDCl_3_): δ 7.97–7.94 (m, 2H), 7.85–7.80 (m, 2H), 7.13–7.03 (m, 7H), 6.93–6.90 (m, 2H), 6.42 (t, *J =* 2.6 Hz, 1H), 4.77 (s, 2H), 3.48 – 3.40 (m, 1H), 2.92 (ddd, *J =* 16.8, 8.3, 2.8 Hz, 1H), 2.60–2.43 (m, 3H), 2.33 (s, 3H); ^13^C­{^1^H }­NMR (101 MHz, CDCl_3_): δ 202.4, 201.2, 171.4, 142.9, 142.2, 141.8, 138.1, 135.9, 135.7, 134.7, 134.2, 132.4, 129.2, 128.34, 128.30, 127.6, 126.4, 123.4, 123.3, 71.0, 66.2, 44.8, 38.4, 35.4, 21.2; HRMS (ESI-TOF) *m*/*z*: [M + H]^+^ calcd. for C_29_H_25_O_4_: 437.1747, Found: 437.1732.

### 4-Methoxybenzyl-2-(1′,3′-Dioxo-2-Phenyl-1′,3′-Dihydrospiro­[Cyclopentane-1,2′-Inden]-2-en-5-yl)­Acetate (**13ha**)

Purified by silica gel column chromatography eluting with Hexane/THF 12:1 to 8:1; 28% yield (12.2 mg); Yellow oil; ^1^H NMR (400 MHz, CDCl_3_): δ 7.97–7.94 (m, 2H), 7.85–7.81 (m, 2H), 7.14–7.03 (m, 5H), 6.93–6.90 (m, 2H), 6.85–6.81 (m, 2H), 6.42 (t, *J =* 2.5 Hz, 1H), 4.75 (s, 2H), 3.79 (s, 3H), 3.48–3.40 (m, 1H), 2.91 (ddd, *J =* 16.8, 8.3, 2.8 Hz, 1H), 2.59–2.42 (m, 3H); ^13^C­{^1^H } NMR (101 MHz, CDCl_3_): δ 202.3, 201.2, 171.4, 159.5, 142.8, 142.1, 141.7, 135.8, 135.7, 134.6, 134.2, 130.0, 128.2, 127.52, 127.47, 126.3, 123.31, 123.27, 113.8, 71.0, 66.0, 55.2, 44.7, 38.4, 35.4; HRMS (ESI-TOF) *m*/*z*: [M + H]^+^ calcd. for C_29_H_25_O_5_: 453.1697, Found: 453.1681.

### Naphthalen-2-Ylmethyl-2-(1′,3′-Dioxo-2-Phenyl-1′,3′-Dihydrospiro­[Cyclopentane-1,2′-Inden]-2-en-5-yl)­Acetate (**13ia**)

Purified by silica gel column chromatography eluting with Hexane/THF 12:1 to 8:1; 45% yield (21.2 mg); Yellow oil; ^1^H NMR (400 MHz, CDCl_3_): δ 7.92–7.78 (m, 5H), 7.75–7.64 (m, 3H), 7.52–7.47 (m, 2H), 7.29 (dt, *J =* 8.3, 1.5 Hz, 1H), 7.08–7.04 (m, 3H), 6.93–6.90 (m, 2H), 6.4 (t, *J* = 2.0 Hz, 1H), 4.98 (s, 2H), 3.51– 3.47 (m, 1H), 2.94 (ddd, *J =* 16.9, 8.3, 2.8 Hz, 1H), 2.65–2.55 (m, 3H); ^13^C­{^1^H } NMR (101 MHz, CDCl_3_): δ 202.4, 201.3, 171.4, 142.8, 142.2, 141.7, 135.8, 135.7, 134.6, 134.2, 133.0, 132.8, 128.3, 128.0, 127.9, 127.63, 127.55, 127.3, 126.34, 126.27, 125.7, 123.2, 71.0, 66.4, 44.6, 38.5, 35.4; HRMS (ESI-TOF) *m*/*z*: [M + H]^+^ calcd. for C_32_H_25_O_4_: 473.1747, Found: 473.1750.

### Ethyl-2-(1′,3′-Dioxo-2-Phenyl-1′,3′-Dihydrospiro­[Cyclopentane-1,2′-Inden]-2-en-5-yl)­Acetate (**13ja**)

Purified by silica gel column chromatography eluting with Hexane/THF 12:1 to 8:1; 53% yield (18.9 mg); Yellow oil; ^1^H NMR (400 MHz, CDCl_3_): δ 8.00–7.95 (m, 2H), 7.86–7.82 (m, 2H), 7.09–7.03 (m, 3H), 6.93–6.90 (m, 2H), 6.42 (t, *J =* 2.5 Hz, 1H), 3.84–3.78 (m, 2H), 3.48–3.40 (m, 1H), 2.93 (ddd, *J =* 16.8, 8.3, 2.9 Hz, 1H), 2.60–2.41 (m, 3H), 1.04 (t, *J =* 7.1 Hz, 3H); ^13^C­{^1^H } NMR (101 MHz, CDCl_3_): δ 202.4, 201.3, 171.5, 142.2, 141.8, 135.9, 135.7, 134.7, 134.2, 128.3, 127.6, 126.4, 123.34, 123.30, 71.0, 60.4, 44.7, 38.4, 35.5, 13.9; HRMS (ESI-TOF) *m*/*z*: [M + H]^+^ calcd. for C_23_H_21_O_4_: 361.1434, Found: 361.1443.

### *tert*-Butyl-2-(1′,3′-Dioxo-2-Phenyl-1′,3′-Dihydrospiro­[Cyclopentane-1,2′-Inden]-2-en-5-yl)­Acetate (**13ka**)

Purified by silica gel column chromatography eluting with Hexane/THF 12:1 to 8:1; 22% yield (8.6 mg); Yellow oil; ^1^H NMR (400 MHz, CDCl_3_): δ 8.00–7.96 (m, 2H), 7.86–7.82 (m, 2H), 7.09–7.03 (m, 3H), 6.94–6.91 (m, 2H), 6.44 (t, *J =* 2.5 Hz, 1H), 3.44–3.36 (m, 1H), 2.91 (ddd, *J =* 16.9, 8.3, 2.8 Hz, 1H), 2.55 (ddd, *J =* 16.9, 8.7, 2.3 Hz, 1H), 2.46–2.34 (m, 2H), 1.20 (s, 9H); ^13^C­{^1^H } NMR (101 MHz, CDCl_3_): δ 201.5, 170.9, 143.1, 142.2, 141.8, 135.8, 135.6, 134.8, 134.4, 128.3, 127.5, 126.3, 123.5, 123.3, 80.7, 71.0, 45.1, 38.3, 36.7, 27.7; HRMS (ESI-TOF) *m*/*z*: [M + H]^+^ calcd. for C_25_H_25_O_4_: 389.1747, Found: 389.1757.

### Ethyl-1,3-Dioxo-6a′-Phenyl-1,3,3a′,5′,6′,6a′-Hexahydro-4′*H*-Spiro­[Indene-2,1′-Pentalene]-2′-Carboxylate (**15a**)

Purified by silica gel column chromatography eluting with Hexane/EA 20:1 to 10:1; 88% yield (34.0 mg); Yellow solid; m.p.: 125–126 °C; ^1^H NMR (400 MHz, CDCl_3_): δ 8.03 (d, *J* = 9.7 Hz, 1H), 7.77 (t, *J* = 7.5 Hz, 1H), 7.62 (t, *J* = 7.4 Hz, 1H), 7.47 (d, *J* = 7.6 Hz, 1H), 7.18 (d, *J* = 2.2 Hz, 1H), 7.00–6.95 (m, 3H), 6.91–6.88 (m, 2H), 4.26 (d, *J* = 7.1 Hz, 1H), 4.03–3.90 (m, 2H), 2.79–2.77 (m, 1H), 2.01 – 1.71 (m, 4H), 1.30–1.24 (m, 1H), 0.99 (t, *J* = 7.1 Hz, 3H); ^13^C­{^1^H }­NMR (101 MHz, CDCl_3_): δ 199.81, 199.77, 163.2, 154.1, 143.3, 142.2, 140.3, 135.1, 135.0, 133.4, 127.8, 127.3, 126.6, 122.8, 122.7, 71.7, 68.4, 60.7, 51.9, 37.2, 27.9, 24.1, 13.6; HRMS (ESI-TOF) *m*/*z*: [M + H]^+^ calcd. for C_25_H_23_O_4_: 387.1591, Found: 387.1597.

### Ethyl-6a′-(4-Fluorophenyl)-1,3-Dioxo-1,3,3a′,5′,6′,6a′-Hexahydro-4′*H*-Spiro­[indene-2,1′-Pentalene]-2′-Carboxylate (**15b**)

Purified by silica gel column chromatography eluting with Hexane/EA 20:1 to 10:1; 58% yield (23.5 mg); Yellow solid; m.p.: 132–133 °C; ^1^H NMR (400 MHz, Acetone-D_6_): δ 8.02 (dt, *J =* 7.7, 1.0 Hz, 1H), 7.94–7.90 (m, 1H), 7.78 (td, *J =* 7.4, 1.1 Hz, 1H), 7.47 (dt, *J =* 7.6, 1.0 Hz, 1H), 7.15 (d, *J =* 2.3 Hz, 1H), 6.99–6.96 (m, 2H), 6.77–6.72 (m, 2H), 4.22 (dt, *J =* 9.4, 3.1 Hz, 1H), 3.98–3.86 (m, 2H), 2.71 (ddd, *J =* 13.1, 11.6, 7.1 Hz, 1H), 2.00–1.95 (m, 1H), 1.88–1.80 (m, 2H), 1.75–1.67 (m, 1H), 1.26–1.14 (m, 1H), 0.97 (t, *J =* 7.1 Hz, 3H); ^13^C­{^1^H } NMR (101 MHz, Acetone-D_6_): δ 199.0, 162.8, 162.6 (d, *J* = 244.2 Hz), 153.7, 146.6, 143.5, 142.4, 136.8 (d, *J* = 3.3 Hz), 135.7, 135.6, 135.5, 133.6, 129.65, 129.57, 123.3, 122.7, 122.5, 114.5 (d, *J* = 21.5 Hz), 71.7, 67.5, 60.4, 52.5, 37.3, 30.3, 27.6, 23.9, 13.2; ^19^F NMR (376 MHz, Acetone-D_6_): δ −117.5 (s, 1F); HRMS (ESI-TOF) *m*/*z*: [M + H]^+^ calcd. for C_25_H_22_O_4_F: 405.1497, Found: 405.1490.

### Ethyl-6a′-(4-Chlorophenyl)-1,3-Dioxo-1,3,3a′,5′,6′,6a’-Hexahydro-4′*H*-Spiro­[Indene-2,1′-Pentalene]-2′-Carboxylate (**15c**)

Purified by silica gel column chromatography eluting with Hexane/EA 20:1 to 10:1; 66% yield (27.9 mg); Yellow solid; m.p.: 137–138 °C; ^1^H NMR (400 MHz, CDCl_3_): δ 8.03 (d, *J* = 7.6 Hz, 1H), 7.78 (t, 1H), 7.67 (t, *J* = 7.4 Hz, 1H), 7.53 (d, *J* = 7.6 Hz, 1H), 7.15 (d, *J* = 2.2 Hz, 1H), 6.96 (d, *J* = 8.2 Hz, 2H), 6.84 (d, *J* = 8.2 Hz, 2H), 4.20 (d, *J* = 9.3 Hz, 1H), 4.02–3.90 (m, 2H), 2.82–2.74 (m, 1H), 2.02–1.94 (m, 1H), 1.87–1.70 (m, 3H), 1.31–1.19 (m, 1H), 0.99 (t, *J* = 7.1 Hz, 3H); ^13^C­{^1^H } NMR (101 MHz, CDCl_3_): δ 199.7, 199.6, 163.0, 153.8, 143.2, 142.1, 139.1, 135.4, 135.2, 133.5, 132.5, 128.7, 128.0, 123.0, 122.8, 71.5, 67.8, 60.8, 52.2, 37.2, 27.9, 24.1, 13.6; HRMS (ESI-TOF) *m*/*z*: [M + Na]^+^ calcd. for C_25_H_21_O_4_ClNa: 443.1021, Found: 443.1015.

### Ethyl-6a’-(4-Bromophenyl)-1,3-Dioxo-1,3,3a’,5′,6′,6a′-Hexahydro-4′*H*-Spiro­[Indene-2,1′-Pentalene]-2′-Carboxylate (**15d**)

Purified by silica gel column chromatography eluting with Hexane/EA 20:1 to 10:1; 65% yield (30.3 mg); Yellow solid; m.p.: 133–134 °C; ^1^H NMR (400 MHz, CDCl_3_): δ 8.04 (dt, *J* = 7.6, 0.9 Hz, 1H), 7.84–7.75 (m, 1H), 7.68 (td, *J* = 7.4, 1.0 Hz, 1H), 7.54 (dt, *J* = 7.6, 0.9 Hz, 1H), 7.15 (d, *J* = 2.3 Hz, 1H), 7.11 (d, *J* = 8.4 Hz, 2H), 6.78 (d, *J* = 8.7 Hz, 2H), 4.19 (dt, *J* = 9.9, 2.9 Hz, 1H), 4.00–3.90 (m, 2H), 2.82–2.74 (m, 1H), 1.99–1.94 (m, 1H), 1.87–1.73 (m, 3H), 1.28–1.19 (m, 2H), 0.99 (t, *J* = 7.1 Hz, 3H); ^13^C­{^1^H } NMR (101 MHz, CDCl_3_): δ 199.7, 199.5, 163.0, 153.7, 143.3, 142.1, 139.7, 135.4, 135.3, 133.5, 130.9, 129.1, 123.1, 122.8, 120.7, 71.4, 67.9, 60.8, 52.2, 37.2, 27.9, 24.1, 13.6; HRMS (ESI-TOF) *m*/*z*: [M + H]^+^ calcd. for C_25_H_22_O_4_Br: 465.0696, Found: 465.0700.

### Ethyl-1,3-Dioxo-6a’-(*p*-Tolyl)-1,3,3a’,5′,6′,6a’-Hexahydro-4′*H*-Spiro­[Indene-2,1′-Pentalene]-2′-Carboxylate (**15e**)

Purified by silica gel column chromatography eluting with Hexane/EA 20:1 to 10:1; 68% yield (27.2 mg); Yellow solid; m.p.: 149–150 °C; ^1^H NMR (400 MHz, CDCl_3_): δ 8.04 (d, *J* = 7.7 Hz, 1H), 7.77 (td, *J* = 7.5, 1.0 Hz, 1H), 7.63 (td, *J* = 7.4, 1.0 Hz, 1H), 7.50 (d, *J* = 7.6 Hz, 1H), 7.17 (d, *J* = 2.3 Hz, 1H), 6.80 (s, 2H), 6.76 (s, 2H), 4.23 (dt, *J* = 9.8, 2.9 Hz, 1H), 4.00–3.93 (m, 2H), 2.80–2.73 (m, 1H), 2.14 (s, 3H), 2.04–1.95 (m, 1H), 1.90–1.69 (m, 3H), 1.32–1.22 (m, 1H), 0.99 (t, *J* = 7.1 Hz, 3H); ^13^C­{^1^H } NMR (101 MHz, CDCl_3_): δ 199.9, 163.2, 154.2, 143.4, 142.3, 137.3, 136.2, 135.1, 134.9, 133.5, 128.5, 127.2, 122.9, 122.7, 60.7, 52.1, 37.3, 28.0, 24.1, 20.7, 13.6; HRMS (ESI-TOF) *m*/*z*: [M + H]^+^ calcd. for C_26_H_25_O_4_: 401.1747, Found: 401.1760.

### Ethyl-6a’-(4-Methoxyphenyl)-1,3-Dioxo-1,3,3a’,5′,6′,6a′-Hexahydro-4′*H*-Spiro­[Indene-2,1′-Pentalene]-2′-Carboxylate (**15f**)

Purified by silica gel column chromatography eluting with Hexane/EA 20:1 to 10:1; 87% yield (36.2 mg); Yellow solid; m.p.: 141–142 °C; ^1^H NMR (400 MHz, CDCl_3_): δ 8.02 (dt, *J* = 7.6, 0.9 Hz, 1H), 7.76 (td, *J* = 7.4, 1.1 Hz, 1H), 7.63 (td, *J* = 7.4, 1.1 Hz, 1H), 7.50 (dt, *J* = 7.6, 1.0 Hz, 1H), 7.16 (d, *J* = 2.2 Hz, 1H), 6.82 (d, *J* = 8.6 Hz, 2H), 6.51 (d, *J* = 8.6 Hz, 2H), 4.20 (dt, *J* = 9.6, 2.9 Hz, 1H), 4.01–3.92 (m, 2H), 3.65 (s, 3H), 2.73 (ddd, *J* = 12.7, 11.6, 6.9 Hz, 1H), 2.02–1.69 (m, 5H), 1.00 (t, *J* = 7.1 Hz, 3H); ^13^C­{^1^H } NMR (101 MHz, CDCl_3_): δ 199.9, 163.2, 158.0, 154.1, 143.3, 142.3, 135.1, 135.0, 133.4, 132.1, 128.5, 122.9, 122.7, 113.1, 71.9, 67.8, 60.7, 55.0, 52.3, 37.1, 27.9, 24.1, 13.7; HRMS (ESI-TOF) *m*/*z*: [M + H]^+^ calcd. for C_26_H_25_O_5_: 417.1697, Found: 417.1704.

### Ethyl-6a’-(Naphthalen-2-yl)-1,3-Dioxo-1,3,3a’,5′,6′,6a’-Hexahydro-4′*H*-Spiro­[Indene-2,1′-Pentalene]-2′-Carboxylate (**15g**)

Purified by silica gel column chromatography eluting with Hexane/EA 20:1 to 10:1; 66% yield (26.1 mg); Yellow solid; m.p.: 152–153 °C; ^1^H NMR (400 MHz, CDCl_3_): δ 8.10 (dt, *J* = 7.6, 0.9 Hz, 1H), 7.77 (td, *J* = 7.5, 1.1 Hz, 1H), 7.63–7.53 (m, 3H), 7.46 (d, *J* = 2.1 Hz, 1H), 7.38–7.34 (m, 3H), 7.23 (d, *J* = 2.2 Hz, 1H), 6.87 (d, *J* = 8.6 Hz, 1H), 4.42 (dt, *J* = 9.7, 2.9 Hz, 1H), 4.02–3.95 (m, 2H), 2.88 (ddd, *J* = 13.1, 11.7, 7.1 Hz, 1H), 2.10–1.74 (m, 5H), 1.00 (t, *J* = 7.1 Hz, 3H); ^13^C­{^1^H } NMR (101 MHz, CDCl_3_): δ 200.0, 199.6, 163.2, 154.1, 143.4, 142.2, 137.9, 135.2, 135.0, 133.5, 132.7, 131.8, 127.9, 127.3, 127.2, 126.4, 126.0, 125.8, 125.4, 123.0, 122.7, 71.6, 68.7, 60.8, 52.1, 37.2, 28.0, 24.1, 13.6; HRMS (ESI-TOF) *m*/*z*: [M + H]^+^ calcd. for C_29_H_24_O_4_: 436.1669, Found: 436.1673.

### Ethyl-1,3-Dioxo-6a’-(Thiophen-2-yl)-1,3,3a’,5′,6′,6a’-Hexahydro-4′*H*-Spiro­[Indene-2,1′-Pentalene]-2′-Carboxylate (**15h**)

Purified by silica gel column chromatography eluting with Hexane/EA 20:1 to 10:1; 60% yield (26.0 mg); Yellow solid; m.p.: 137–138 °C; ^1^H NMR (400 MHz, CDCl_3_): δ 8.00 (dt, *J* = 7.6, 1.0 Hz, 1H), 7.76 (td, *J* = 7.4, 1.1 Hz, 1H), 7.66 (td, *J* = 7.4, 1.1 Hz, 1H), 7.57 (dt, *J* = 7.6, 1.1 Hz, 1H), 7.13 (d, *J* = 2.2 Hz, 1H), 6.91 (d, *J* = 5.1 Hz, 1H), 6.68–6.66 (m, 1H), 6.52 (d, *J* = 3.6 Hz, 1H), 4.07 (dt, *J* = 9.7, 2.9 Hz, 1H), 4.03–3.93 (m, 2H), 2.74–2.69 (m, 1H), 2.08–2.03 (m, 1H), 1.93–1.88 (m, 1H), 1.84–1.73 (m, 2H), 1.43–1.36 (m, 1H), 1.0 (t, *J* = 7.1 Hz, 3H); ^13^C­{^1^H } NMR (101 MHz, CDCl_3_): δ 199.1, 198.9, 163.1, 153.3, 145.0, 143.2, 142.1, 135.1, 133.3, 126.5, 124.2, 122.9, 122.8, 72.2, 64.8, 60.8, 56.0, 38.5, 27.9, 24.7, 13.7; HRMS (ESI-TOF) *m*/*z*: [M + H]^+^ calcd. for C_23_H_21_O_4_S: 393.1155, Found: 393.1167.

### Benzyl-(*Z*)-2-(5-Hydroxy-4-Methyl-4-Phenyl-4,5-Dihydroindeno­[1,2-*b*]­Pyran-2­(3*H*)-Ylidene)­Acetate ((*Z*)-**16**)

In a 7 mL glass vial, (*Z*)-**11** (42 mg, 0.1 mmol) was dissolved in 2.0 mL MeOH and then NaBH_4_ (113 mg, 0.3 mmol) was added at rt. The mixture was stirred for 24 h at room temperature. After the reaction completed (confirmed by TLC), the residue was diluted with saturated NH_4_Cl solution, and the aqueous solution was extracted with DCM. The combined organic layers were dried over Na_2_SO_4_, and the solvent was removed in vacuo. The reaction mixture was purified by column chromatography (SiO_2_, Hexanes: EtOAc, 20:1 to 10:1) to obtain the pure product **16** (28.4 mg, 67%).

*dr* > 20:1; Yellow oil; ^1^H NMR (400 MHz, CDCl_3_): δ 7.59–7.47 (m, 1H), 7.42–7.25 (m, 12H), 7.24–7.19 (m, 1H), 5.27 (s, 1H), 5.18 (s, 2H), 5.01 (d, *J =* 1.4 Hz, 1H), 2.75 (dd, *J =* 14.5, 1.5 Hz, 1H), 2.67 (d, *J =* 14.5 Hz, 1H), 1.80 (s, 3H); ^13^C­{^1^H } NMR (101 MHz, CDCl_3_): δ 164.2, 161.4, 150.1, 144.9, 144.1, 136.2, 135.0, 128.7, 128.5, 128.3, 128.0, 127.2, 126.9, 125.9, 123.4, 122.3, 118.3, 73.7, 65.8, 45.3, 37.8, 26.0; HRMS (ESI-TOF) *m*/*z*: [M + H]^+^ calcd. for C_28_H_25_O_4_: 425.1747, Found: 425.1744.

### Benzyl-2-(1′,3′-Dihydroxy-2-Phenyl-1′,3′-Dihydrospiro­[Cyclopentane-1,2′-Inden]-2-en-5-yl)­Acetate (**17**)

In a 7 mL glass vial, **13aa** (42 mg, 0.1 mmol) was dissolved in 2.0 mL MeOH and then NaBH_4_ (113 mg, 0.3 mmol) was added at rt. The mixture was stirred for 24 h at room temperature. After the reaction completed (confirmed by TLC), the residue was diluted with saturated NH_4_Cl solution, and the aqueous solution was extracted with DCM. The combined organic layers were dried over Na_2_SO_4_, and the solvent was removed in vacuo. The reaction mixture was purified by column chromatography (SiO_2_, Hexanes: EtOAc, 10:1 to 5:1) to obtain the pure product **17** (25.4 mg, 60%).

*dr* > 20:1; Yellow oil; ^1^H NMR (400 MHz, CDCl_3_): δ 7.37–7.17 (m, 9H), 7.01–6.98 (m, 1H), 6.97–6.90 (m, 2H), 6.73–6.71 (m, 2H), 6.16 (t, *J =* 2.6 Hz, 1H), 5.16 (s, 2H), 5.13 (s, 1H), 4.94 (s, 1H), 3.23–3.20 (m, 1H), 2.90–2.71 (m, 3H), 2.34 (ddd, *J =* 16.8, 7.6, 2.3 Hz, 1H), 1.95 (s, 2H); ^13^C­{^1^H } NMR (101 MHz, CDCl_3_): δ 172.8, 143.7, 142.6, 142.2, 137.9, 137.3, 135.7, 128.63, 128.57, 128.5, 128.3, 127.8, 126.9, 122.9, 122.7, 78.3, 75.4, 74.1, 66.5, 42.8, 37.1, 36.4; HRMS (ESI-TOF) *m*/*z*: [M-H]^−^ calcd. for C_28_H_25_O_4_: 425.1747, Found: 425.1764.

## Supplementary Material



## Data Availability

The data underlying this study are available in the published article and its Supporting Information.

## Notes

General information; preparation of starting materials; X-ray analysis data; NMR data; and NMR spectra of all compounds (PDF)
