# Supplementary material for: Catalyst-Controlled Divergent Annulation Reactions of Allenoates and Fully Substituted 1,3-Indandione Derived Electron-Deficient Alkenes
Source: J Org Chem. 2026 Jun 24;91(26):8930–49. doi: 10.1021/acs.joc.6c00628 (PMC13339656; doi:10.1021/acs.joc.6c00628)
Supplement: Supplementary file 1 [file jo6c00628_si_001.pdf]

Supporting Information

Catalyst-controlled Divergent Annulation Reactions of Allenoates and Fully Substituted 1,3-Indandione Derived Electron-Deficient Alkenes

Wu-Dong Yu, I-Ting Chen, Ting-Hung Chu, Po-Heng Lin and Jeng-Liang Han*

Department of Chemistry, National Chung Hsing University, 145 Xingda Rd., South Dist., Taichung City 402, Taiwan

E-mail: jlhan@nchu.edu.tw

Table of Contents:

1.	General Experimental Details	S2
2.	Preparation of Starting Materials	S3
3.	Computational Methods	S6
4.	References	S6
5.	Characterization Data of Starting Materials	S7
6.	Relative Configuration Determination of **16** and **17**	S10
7.	X-Ray Analysis Data	S12
8.	Copies of NMR Spectra of Products	S53
9.	Cartesian Coordinates	S139

1. General Experimental Details

All commercially available reagents were used without further purification unless otherwise stated. All reaction solvents were purified before use. Proton nuclear magnetic resonance (^{1}H NMR) spectra were recorded on a commercial instrument at 400 MHz. Carbon-13 nuclear magnetic resonance (^{13}C{^{1}H } NMR) spectra were recorded at 100 MHz. The proton signal for residual non-deuterated solvent (δ 7.26 for $CHCl_3$) was used as an internal reference for ^{1}H NMR spectra. For ^{13}C{^{1}H} NMR spectra, chemical shifts are reported relative to the δ 77.0 resonance of $CHCl_3$. Coupling constants are reported in Hz. Melting points were determined on a BUCHI B-545 melting point apparatus and are uncorrected. High resolution mass spectra were recorded on a commercial high-resolution mass spectrometer. The single crystal was measured by Bruker D8 VENTURE X-ray Single Crystal Diffractometer. Analytical thin-layer chromatography (TLC) was performed on silica gel 60 F254 pre-coated plates with visualization under UV light. Column chromatography was generally performed using 40-63 μm (230-400 mesh) silica gel, typically using a 50-100:1 weight ratio of silica gel to crude product.

2. Preparation of Starting Materials

2.1 Preparation of 2-(1-arylethylidene)-indene-1,3-diones 10a-h

Compounds **10** were prepared according to known procedures. [1,2]

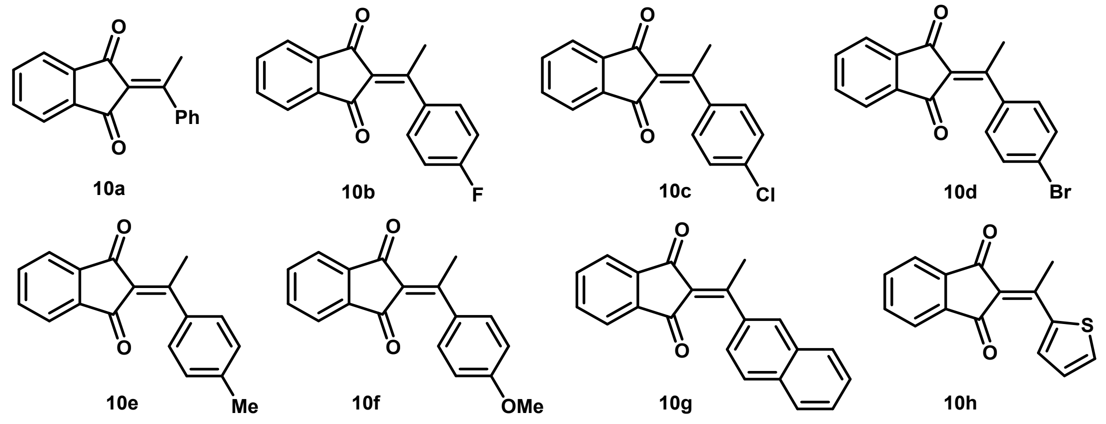

2.2 Preparation of Dialkyl-substituted 1,3-indanediones 10i-m

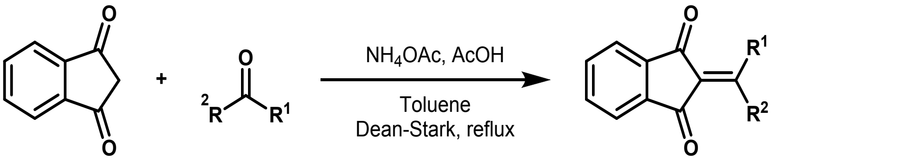

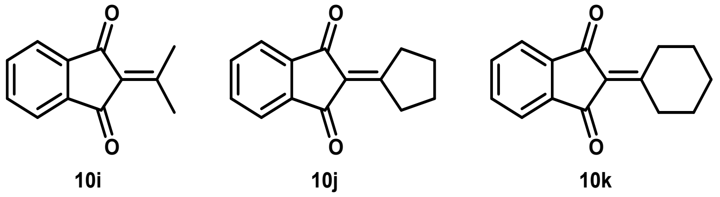

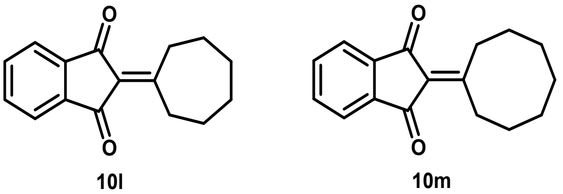

General Procedure for the Synthesis of 10i-m

A 50 mL round-bottom flask was charged with NH_4OAc (2.0 mmol, 0.2 equiv.), 1,3-indandione (10.0 mmol, 1.0 equiv.), ketone (15.0 mmol, 1.5 equiv.), AcOH (1.0 mmol, 0.1 equiv.), molecular sieves (1 g), and toluene (20.0 mL). The mixture was heated to reflux for 16 h (oil bath). Upon completion, as confirmed by TLC, the reaction mixture was filtered through a pad of Celite and washed with dichloromethane. The filtrate was concentrated under reduced pressure, and the crude product was purified by column chromatography using a gradient elution (Hexane/EtOAc = 20:1 to 10:1) to afford compounds **10i-m**.

2.3 Preparation of Allenoates 2

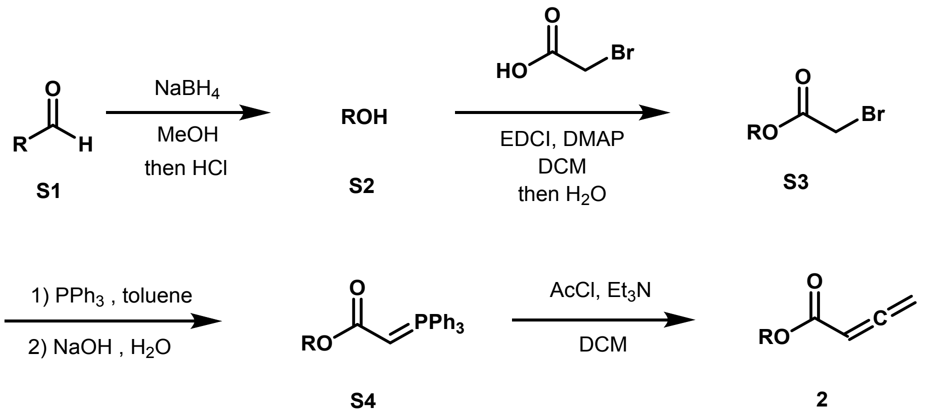

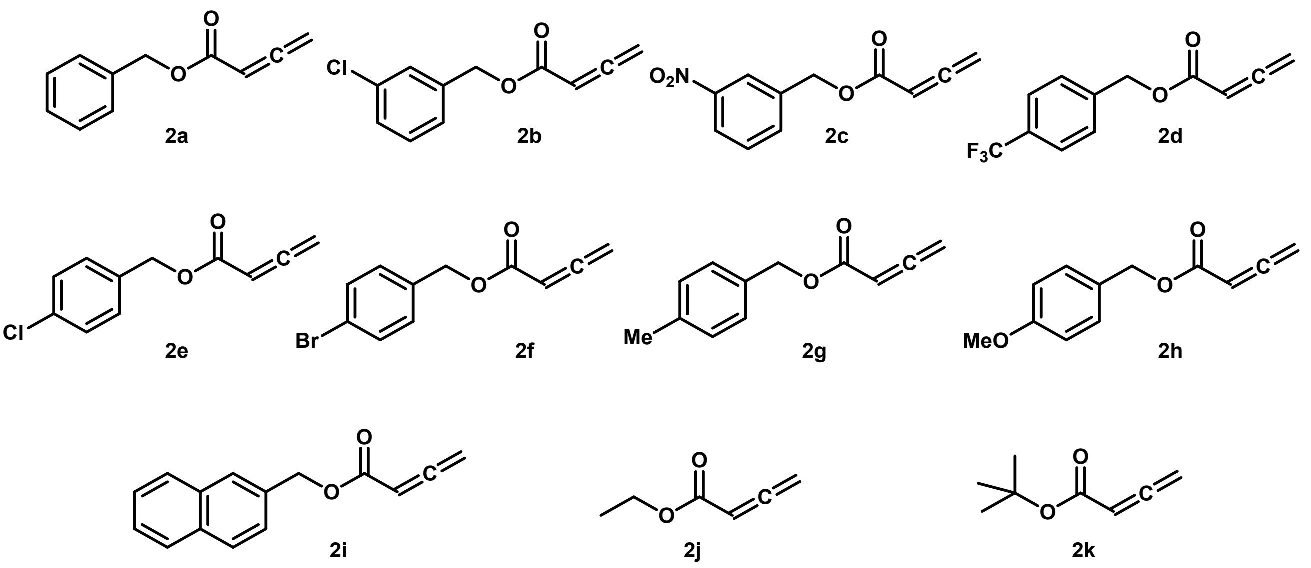

Step 1 – Reduction of Aldehyde S1

Aldehydes **S1** (20 mmol, 1.0 equiv.) was dissolved in 0.1 M methanol. Sodium borohydride ($NaBH_4$, 24 mmol, 1.2 equiv.) was added to the solution in an ice bath. The reaction mixture was stirred at room temperature for several hours. Upon completion, as confirmed by TLC, the reaction was quenched with 3% aqueous HCl. The solvent was removed under reduced pressure to dryness, and the residue was extracted several times with water and dichloromethane. The combined organic layers were dried over anhydrous Na_2SO_4, filtered, and concentrated under reduced pressure to afford alcohols **S2**, which was used in the next step without purification.

Step 2 – Esterification to Compound S3

Alcohols **S2** (1.0 equiv.) and bromoacetic acid (1.2 equiv.) were dissolved in 0.2 M dichloromethane. EDCI (3.0 equiv.) and DMAP (0.1 equiv.) were added sequentially. The reaction mixture was stirred at room temperature overnight and monitored by TLC. Upon completion, the reaction was quenched

with water and the product was extracted with dichloromethane. The combined organic extracts were dried over anhydrous Na_2SO_4, filtered, and concentrated under reduced pressure to yield compounds **S3**, which was used directly in the subsequent step without purification.

Step 3 – Preparation of Phosphonium ylides S4

In a 300 mL round-bottom flask equipped with a magnetic stir bar, triphenylphosphine (PPh_3, 2.0 equiv.) was dissolved in 0.2 M toluene. The ester (1.0 equiv.) was added dropwise over 15 min. The reaction mixture was stirred at room temperature overnight, and the resulting precipitate was collected by filtration, washed with n-hexane (3 × 5 mL), and dried to give the white phosphonium salt. This solid was dissolved in water (150 mL), and saturated NaOH solution was added dropwise until the pH exceeded 7 (monitored by indicator paper). After approximately 15 min, a white solid precipitated. The mixture was extracted with dichloromethane, and the combined organic layers were dried over anhydrous Na_2SO_4, filtered, and concentrated under reduced pressure to give compounds **S4**, which was used directly in the next step without purification.

Step 4 – Synthesis of Allenoates 2

A 100 mL two-neck round-bottom flask equipped with a magnetic stir bar was flame-dried under high vacuum and backfilled with nitrogen (three cycles). The stabilized ylides **S4** was dissolved in dichloromethane (30 mL) and triethylamine (Et_3N, 1.2 equiv.) and transferred into the reaction flask. After stirring for 10 min, acetyl chloride (1.0 equiv.) was added dropwise in an ice bath. The reaction mixture was then allowed to warm to room temperature and stirred for ~4 h, during which the solution turned clear yellow. The progress was monitored by TLC. Upon completion, the solvent was removed under reduced pressure, and the crude product was purified by column chromatography using a gradient elution (Hexane/EtOAc = 20:1 to 10:1) to afford compounds **2**.

2.4 Preparation of Allenoate 14

Compound **14** were prepared according to known procedures.[3]

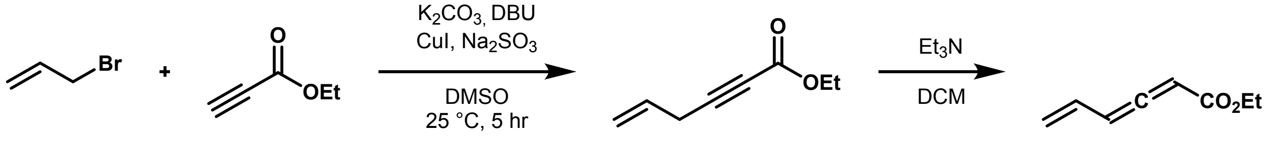

14

3. Computational Methods

Calculations were performed using the Gaussian 16 (Revision C.01) software package.[4] Geometry optimizations and transition state searches were carried out using Density Functional Theory (DFT) at the M06-2X [5]level of theory with the 6-31G(d,p)[6] basis set. Solvent effects were accounted for using the SMD (Solvation Model based on Density) model, utilizing the Solvent Accessible Surface (SAS) for calculations. Convergence was ensured using an ultrafine integration grid.

Single-point energies were further refined at the M06-2X-D3 level (incorporating Grimme's D3[7] dispersion correction) with the def2-TZVPP[8] basis set, also employing the SMD solvation model. All computed structures were visualized using CYLView20.[9]

4. References

1. Möhlmann, L.; Chang, G.-H; Reddy, G. M.; Lee, C.-J.; Lin, W. *Org. Lett.* **2016**, *18*, 688.
2. Kuan, J.-Y.; Chen, I.-T.; Lin, H; Han, J.-L. *Adv. Synth. Catal.* **2023**, *365*, 3493.
3. Feng, J.; Chen, Y.; Qin, W.; Huang, Y. *Org. Lett.* **2020**, *22*, 433.
4. Gaussian 16, Revision 1.1, Frisch, M. J.; Trucks, G. W.; Schlegel, H. B.; Scuseria, G. E.; Robb, M. A.; Cheeseman, J. R.; Scalmani, G.; Barone, V.; Petersson, G. A.; Nakatsuji, H.; Li, X.; Caricato, M.; Marenich, A. V.; Bloino, J.; Janesko, B. G.; Gomperts, R.; Mennucci, B.; Hratchian, H. P.; Ortiz, J. V.; Izmaylov, A. F.; Sonnenberg, J. L.; Williams-Young, D.; Ding, F.; Lipparini, F.; Egidi, F.; Goings, J.; Peng, B.; Petrone, A.; Henderson, T.; Ranasinghe, D.; Zakrzewski, V. G.; Gao, J.; Rega, N.; Zheng, G.; Liang, W.; Hada, M.; Ehara, M.; Toyota, K.; Fukuda, R.; Hasegawa, J.; Ishida, M.; Nakajima, T.; Honda, Y.; Kitao, O.; Nakai, H.; Vreven, T.; Throssell, K.; Montgomery, J. A., Jr.; Peralta, J. E.; Ogliaro, F.; Bearpark, M. J.; Heyd, J. J.; Brothers, E. N.; Kudin, K. N.; Staroverov, V. N.; Keith, T. A.; Kobayashi, R.; Normand, J.; Raghavachari, K.; Rendell, A. P.; Burant, J. C.; Iyengar, S. S.; Tomasi, J.; Cossi, M.; Millam, J. M.; Klene, M.; Adamo, C.; Cammi, R.; Ochterski, J. W.; Martin, R. L.; Morokuma, K.; Farkas, O.; Foresman, J. B.; Fox, D. J. Gaussian, Inc., Wallingford CT, 2016.
5. Zhao, Y.; Truhlar, D. G. *Theor. Chem. Acc.* **2008**, *120*, 215.
6. Raghavachari, K.; Binkley, J. S.; Seeger, R.; Pople, J. A. *J. Chem. Phys.* **1980**, *72*, 650.
7. Grimme, S.; Ehrlich, S.; Goerigk, L. *J. Comp. Chem.* **2011**, *32*, 1456.
8. Weigend, F.; Ahlrichs, R. *Phys. Chem. Chem. Phys.*, **2005**, *7,* 3297.
9. CYLview20; C. Y. Legault, Université de Sherbrooke, **2020** (http://www.cylview.org).

5. Characterization Data of Starting Materials

2-(Propan-2-ylidene)-1*H*-indene-1,3(2*H*)-dione (10i)

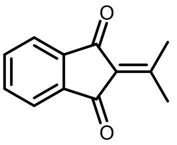

10i

Purified by silica gel column chromatography eluting with Hexane/EA 20:1 to 10:1; 80% yield (1488.0 mg); White solid; m.p.: 145-146 °C; ^{1}H NMR (400 MHz, $CDCl_3$): δ 7.90 (dd, *J* = 5.6, 3.1 Hz, 2H), 7.73 (dd, *J* = 5.6, 3.1 Hz, 2H), 2.60 (s, 6H); ^{13}C{^{1}H} NMR (101 MHz, $CDCl_3$): δ 191.3, 170.3, 140.9, 134.7, 127.1, 122.8, 23.7; HRMS (APCI) *m/z*: $[M+H]^+$ calcd. for $C_{12}H_{11}O_2$: 187.0754, Found: 187.0751.

2-Cyclopentylidene-1*H*-indene-1,3(2*H*)-dione (10j)

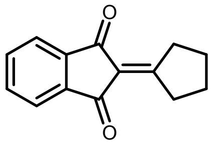

10j

Purified by silica gel column chromatography eluting with Hexane/EA 20:1 to 10:1; 75% yield (795.9 mg); Light yellow solid; m.p.: 174-175 °C; ^{1}H NMR (400 MHz, $CDCl_3$): δ 7.91 (dd, *J* = 5.6, 3.0 Hz, 2H), 7.74 (dd, *J* = 5.6, 3.0 Hz, 2H), 3.21-3.17 (m, 4H), 1.86-1.82 (m, 4H); ^{13}C{^{1}H} NMR (101 MHz, $CDCl_3$): δ 190.9, 180.8, 141.1, 134.6, 124.5, 122.8, 35.3, 25.5; HRMS (APCI) *m/z*: $[M+H]^+$ calcd. for $C_{14}H_{13}O_2$: 213.0910, Found: 213.0907.

2-Cyclohexylidene-1*H*-indene-1,3(2*H*)-dione (10k)

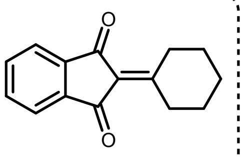

10k

Purified by silica gel column chromatography eluting with Hexane/EA 20:1 to 10:1; 70% yield (791.4 mg) ; Light yellow solid; m.p.: 100-101 °C; ^{1}H NMR (400 MHz, $CDCl_3$): δ 7.93-7.88 (m, 2H), 7.76-7.72 (m, 2H), 3.26 (t, *J* = 6.3 Hz, 4H), 1.86-1.80 (m, 4H), 1.73-1.67 (m, 2H); ^{13}C{^{1}H} NMR (101 MHz, $CDCl_3$): δ 191.8, 178.9, 140.9, 134.7, 124.4, 122.8, 31.2, 29.3, 26.0; HRMS (APCI) *m/z*: $[M+H]^+$ calcd. for $C_{15}H_{15}O_2$: 227.1067, Found: 227.1064.

2-Cycloheptylidene-1*H*-indene-1,3(2*H*)-dione (10l)

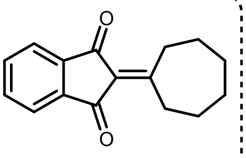

10l

Purified by silica gel column chromatography eluting with Hexane/EA 20:1 to 10:1; 37% yield (444.6 mg); Orange solid; m.p.: 87-88 °C; ^{1}H NMR (400 MHz, $CDCl_3$): δ 7.90-7.87 (m, 2H), 7.75-7.71 (m, 2H), 3.35-3.32 (m, 4H), 1.84-1.78 (m, 4H), 1.60-1.56 (m, 4H); ^{13}C{^{1}H} NMR (101 MHz, $CDCl_3$): δ 191.6, 181.5, 140.8, 134.6, 126.1, 122.7, 33.8, 29.1, 26.3; HRMS (APCI) *m/z*: $[M+H]^+$ calcd. for $C_{16}H_{17}O_2$: 241.1223, Found: 241.1221.

2-Cyclooctylidene-1*H*-indene-1,3(2*H*)-dione (10m)

Purified by silica gel column chromatography eluting with Hexane/EA 20:1 to 10:1; 21% yield (267

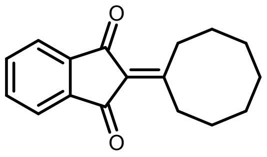

10m

mg); Light orange solid; m.p.: 83-84 °C; ^{1}H NMR (400 MHz, $CDCl_3$): δ 7.78-7.75 (m, 2H), 7.64-7.61 (m, 2H), 3.17-3.14 (m, 4H), 1.90-1.84 (m, 4H), 1.47-1.41 (m, 4H), 1.28-1.22 (m, 2H); $^{13}C\{^1H\}$ NMR (101 MHz, $CDCl_3$): δ 190.9, 184.6, 140.5, 134.3, 125.5, 122.5, 32.9, 27.5, 27.0, 25.8; HRMS (APCI) *m/z*: $[M+H]^+$ calcd. for $C_{17}H_{19}O_2$: 255.1380, Found: 255.1375.

3-Chlorobenzyl buta-2,3-dienoate (2b)

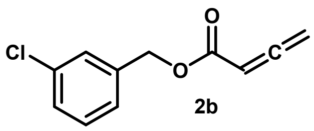

2b

Purified by silica gel column chromatography eluting with Hexane/EA 20:1 to 10:1 ; 32% yield (134 mg); colorless liquid ; ^{1}H NMR (400 MHz, $CDCl_3$): δ 7.37 – 7.36 (m, 1H), 7.30 – 7.29 (m, 2H), 7.27 – 7.22 (m, 1H), 5.69 (t, *J* = 6.6 Hz, 1H), 5.26 (d, *J* = 6.6 Hz, 2H), 5.16 (s, 2H); $^{13}C\{^1H\}$ NMR (101 MHz, $CDCl_3$): δ 216.0, 165.3, 137.8, 134.3, 129.7, 128.2, 128.0, 126.0, 87.6, 79.5, 65.6; HRMS (ESI-TOF) *m/z*: $[M+H]^+$ calcd. for $C_{11}H_{10}O_2Cl$: 209.0364, Found: 209.0374.

3-Nitrobenzyl buta-2,3-dienoate (2c)

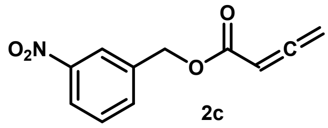

2c

Purified by silica gel column chromatography eluting with Hexane/EA 20:1 to 10:1 ; 35% yield (77 mg); colorless liquid ; ^{1}H NMR (400 MHz, $CDCl_3$): δ 8.25 – 8.24 (m, 1H), 8.19 – 8.16 (m, 1H), 7.71 – 7.68 (m, 1H), 7.55 (t, *J* = 7.9 Hz, 1H), 5.70 (t, *J* = 6.6 Hz, 1H), 5.29 (d, *J* = 6.8 Hz, 2H), 5.27 (s, 2H); $^{13}C\{^1H\}$ NMR (101 MHz, $CDCl_3$): δ 216.1, 165.1, 148.2, 137.9, 133.7, 129.5, 123.0, 122.6, 87.3, 79.6, 64.9; HRMS (ESI-TOF) *m/z*: $[M+H]^+$ calcd. for $C_{11}H_{10}O_4N$: 220.0604, Found: 220.0614.

4-(Trifluoromethyl)benzyl buta-2,3-dienoate (2d)

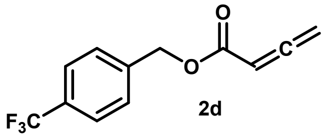

2d

Purified by silica gel column chromatography eluting with Hexane/EA 20:1 to 10:1; 30% yield (73 mg); colorless liquid; ^{1}H NMR (400 MHz, $CDCl_3$): δ 7.62 (d, *J* = 8.1 Hz, 2H), 7.48 (d, *J* = 8.0 Hz, 2H), 5.70 (t, *J* = 6.5 Hz, 1H), 5.26 (d, *J* = 6.6 Hz, 2H), 5.24 (s, 2H); $^{13}C\{^1H\}$ NMR (101 MHz, $CDCl_3$): δ 216.2, 165.3, 139.8, 130.5 (q, *J* = 32.2 Hz), 128.1, 128.0, 125.5 (q, *J* = 3.8 Hz), 87.6, 79.5, 65.6; ^{19}F NMR (376 MHz, $CDCl_3$): δ -62.5; HRMS (ESI-TOF) *m/z*: $[M+H]^+$ calcd. for $C_{12}H_{10}O_2F_3$: 243.0627, Found: 243.0636.

4-Chlorobenzyl buta-2,3-dienoate (2e)

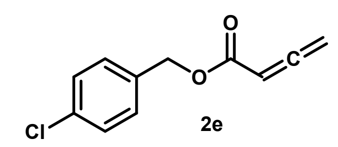

Purified by silica gel column chromatography eluting with Hexane/EA 20:1 to 10:1; 50% yield (103 mg); colorless liquid; ^{1}H NMR (400 MHz, $CDCl_3$): δ 7.35 – 7.29 (m, 4H), 5.67 (t, *J* = 6.5 Hz, 1H), 5.24 (d, *J* = 6.5 Hz, 2H), 5.15 (s, 2H);^{13}C{^{1}H} NMR (101 MHz, $CDCl_3$): δ 215.9, 165.3, 134.2, 134.0, 129.5, 129.4, 128.7, 128.6, 87.6, 79.4, 65.7; HRMS (ESI-TOF) *m/z*: $[M-H]^-$ calcd. for $C_{11}H_8O_2Cl$: 207.0207, Found: 207.0205.

4-Bromobenzyl buta-2,3-dienoate (2f)

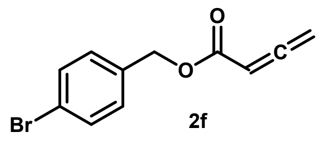

Purified by silica gel column chromatography eluting with Hexane/EA 20:1 to 10:1; 53% yield (133 mg); colorless liquid; ^{1}H NMR (400 MHz, $CDCl_3$): δ 7.51 – 7.47 (m, 2H), 7.25 – 7.23 (m, 2H), 5.67 (t, *J* = 6.5 Hz, 1H), 5.24 (d, *J* = 6.5 Hz, 2H), 5.13 (s, 2H); ^{13}C{^{1}H} NMR (101 MHz, $CDCl_3$): δ 216.0, 165.4, 134.8, 131.7, 131.6, 129.9, 129.8, 122.2, 87.6, 79.5, 65.7; HRMS (ESI-TOF) *m/z*: $[M-H]^-$ calcd. for $C_{11}H_8O_2Br$: 250.9702, Found: 250.9702.

4-Methylbenzyl buta-2,3-dienoate (2g)

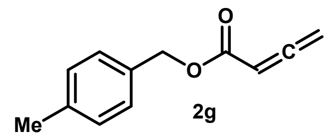

Purified by silica gel column chromatography eluting with Hexane/EA 20:1 to 10:1; 35% yield (66 mg); colorless liquid; ^{1}H NMR (400 MHz, $CDCl_3$): δ 7.27 (d, *J* = 7.7 Hz, 2H), 7.17 (d, *J* = 7.7 Hz, 2H), 5.67 (t, *J* = 6.5 Hz, 1H), 5.23 (d, *J* = 6.5 Hz, 2H), 5.15 (s, 2H), 2.35 (s, 3H);^{13}C{^{1}H} NMR (101 MHz, $CDCl_3$): δ 215.9, 165.5, 138.0, 133.0, 132.7, 129.1, 128.4, 128.3, 87.8, 79.3, 66.5, 21.1; HRMS (ESI-TOF) *m/z*: $[M+H]^+$ calcd. for $C_{12}H_{13}O_2$: 189.0910, Found: 189.0917.

4-Methoxybenzyl buta-2,3-dienoate (2h)

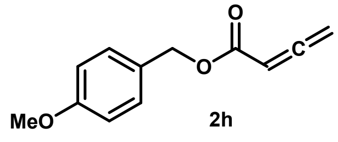

Purified by silica gel column chromatography eluting with Hexane/EA 20:1 to 10:1; 27% yield (55 mg); colorless liquid; ^{1}H NMR (400 MHz, $CDCl_3$): δ 7.33 – 7.29 (m, 2H), 6.91 – 6.87 (m, 2H), 5.66 (t, *J* = 6.5 Hz, 1H), 5.22 (d, *J* = 6.5 Hz, 2H), 5.12 (s, 2H), 3.81 (s, 3H); ^{13}C{^{1}H} NMR (101 MHz, $CDCl_3$): δ 215.8, 165.6, 159.6, 130.0, 127.9, 113.8, 87.9, 79.4, 66.4, 55.2; HRMS (ESI-TOF) m/z: $[M+H]^+$ calcd. for $C_{12}H_{12}O_3$: 204.0781, Found: 204.0789.

Naphthalen-2-ylmethyl buta-2,3-dienoate (2i)

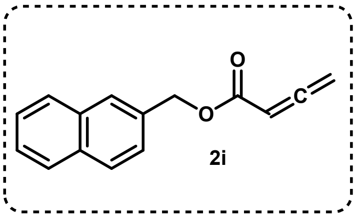

2i

Purified by silica gel column chromatography eluting with Hexane/EA 20:1 to 10:1; 42% yield (94 mg); colorless liquid; ^{1}H NMR (400 MHz, $CDCl_3$): δ 7.86 – 7.83 (m, 4H), 7.51 – 7.46 (m, 3H), 5.71 (t, J = 6.5 Hz, 1H), 5.36 (s, 2H), 5.25 (d, J = 6.5 Hz, 2H); $^{13}C\{^{1}H\}$ NMR (101 MHz, $CDCl_3$): δ 216.0, 165.6, 133.2, 133.11, 133.06, 128.3, 128.0, 127.7, 127.3, 126.2, 125.8, 87.8, 79.5, 66.8; HRMS (ESI-TOF) *m/z*: $[M+H]^+$ calcd. for $C_{15}H_{12}O_2$: 224.0832, Found: 224.0832.

6. Relative Configuration Determination of 16 and 17

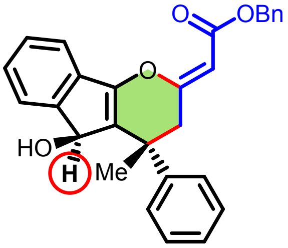

16

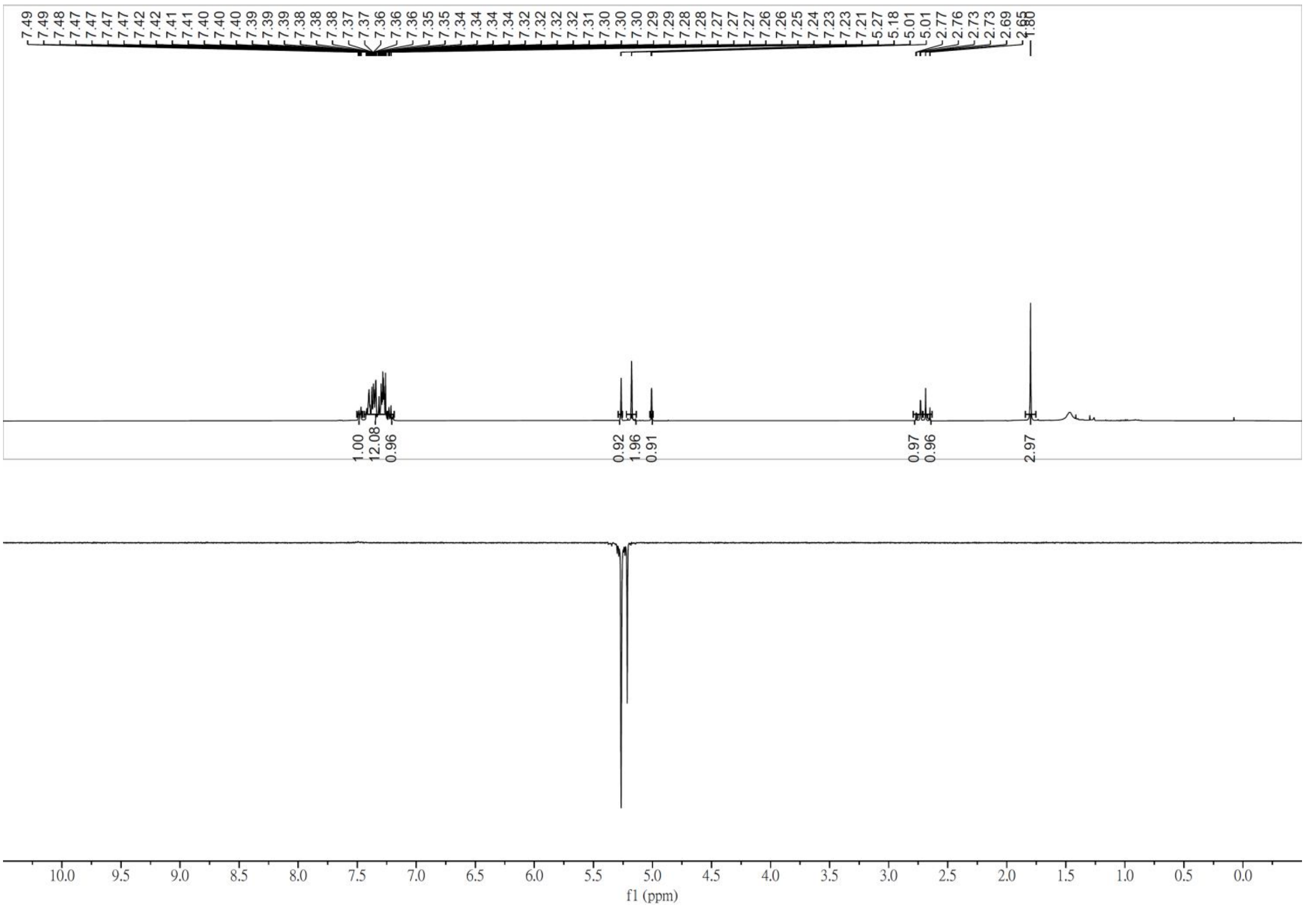

The relative configuration of compound **16** was determined by NOE. Because the circled proton

doesn't have any NOEs with other protons, the hydroxy group and methyl group are assigned to be *cis* configuration.

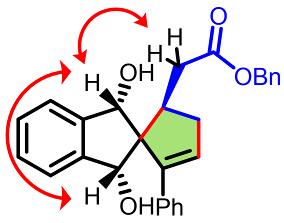

17

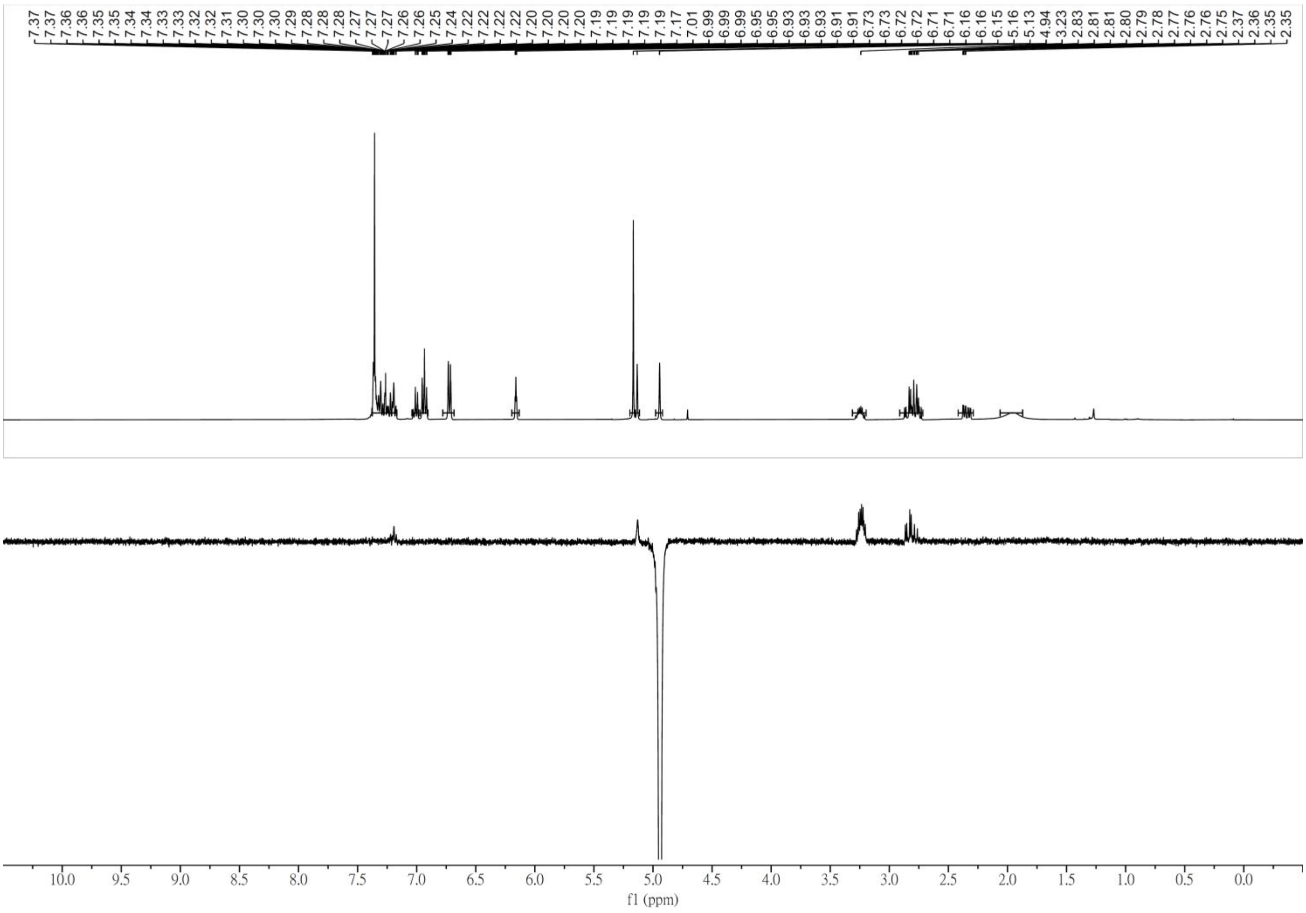

The relative configuration of compound **17** was determined by NOE. We found two benzylic protons have a NOE effect, hence these two protons are assigned to be *cis* configuration. In addition, one benzylic proton has a NOE effect with protons at the α-carbon of carbonyl group, so the ester group is assigned to be *cis* configuration with two benzylic protons.

7. X-Ray Analysis Data

CCDC 2479629 (11ad)

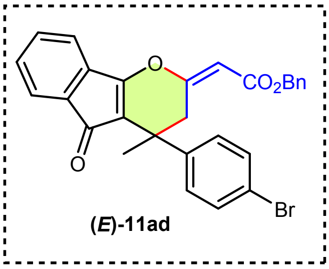

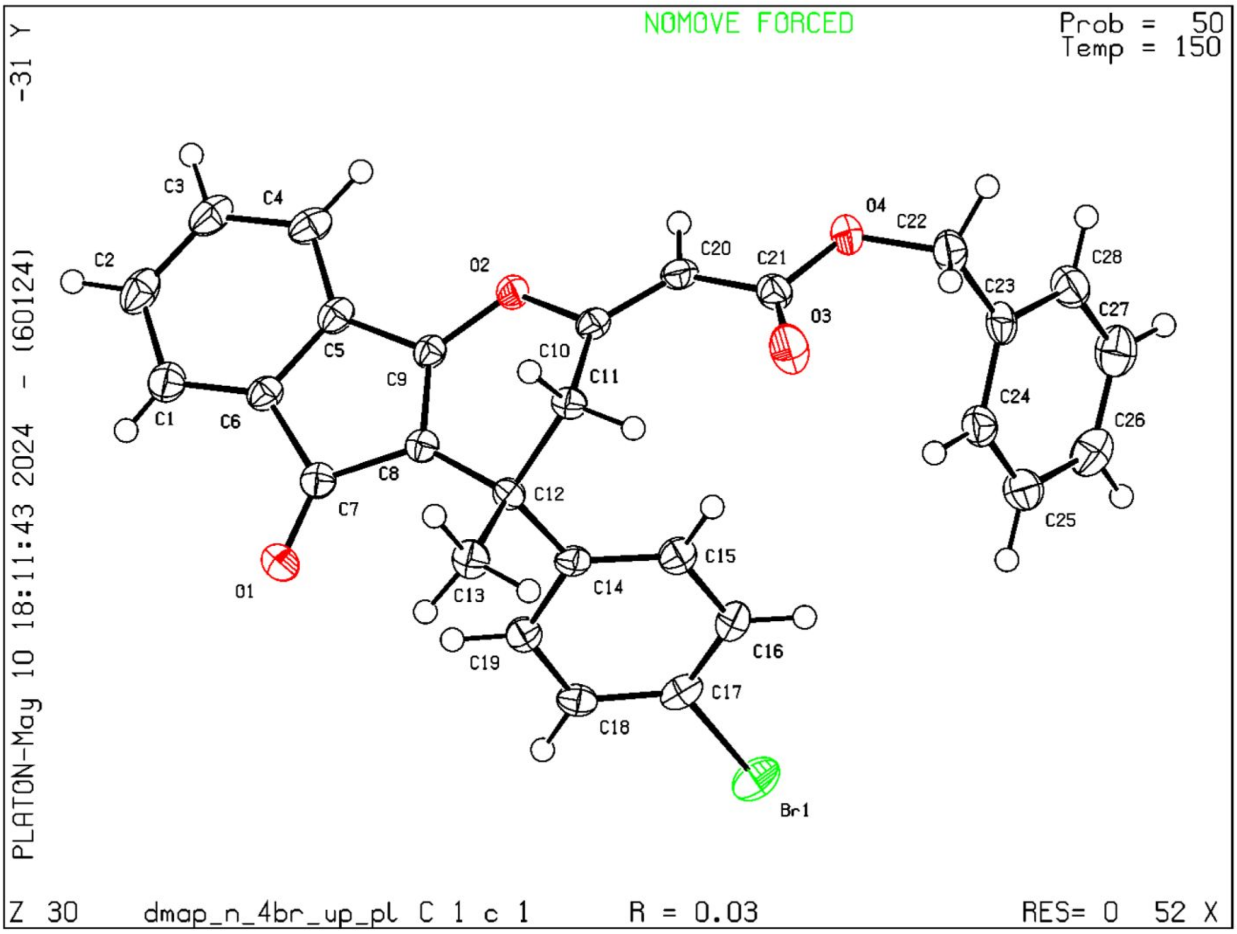

ORTEP drawing of **11ad** showing thermal ellipsoids at the 50% probability level

The crystal was obtained by slow evaporation of **11ad** in ether at room temperature.

Crystal data and structure refinement for **11ad**

Identification code	dmap_n_4br_up_pl	
Empirical formula	C28 H21 Br O4	
Formula weight	501.36	
Temperature	150.15 K	
Wavelength	0.71073 Å	
Crystal system	Monoclinic	
Space group	C 1 c 1	
Unit cell dimensions	a = 24.7670(17) Å	a= 90°.
	b = 9.0254(6) Å	b= 100.796(4)°.
	c = 10.1081(6) Å	g = 90°.
Volume	2219.5(3) $Å^3$	
Z	4	
Density (calculated)	1.500 Mg/m^3	
Absorption coefficient	1.886 mm^{-1}	
F(000)	1024	
Crystal size		
Theta range for data collection	3.060 to 26.390°.	
Index ranges	-30<=h<=30, -11<=k<=11, -12<=l<=12	
Reflections collected	32997	
Independent reflections	4516 [R(int) = 0.0604]	
Completeness to theta = 25.242°	99.8 %	
Absorption correction	None	
Refinement method	Full-matrix least-squares on F^2	
Data / restraints / parameters	4516 / 2 / 308	
Goodness-of-fit on F^2	1.043	
Final R indices [I>2sigma(I)]	R1 = 0.0261, wR2 = 0.0560	
R indices (all data)	R1 = 0.0348, wR2 = 0.0606	
Absolute structure parameter	0.022(9)	
Extinction coefficient	n/a	
Largest diff. peak and hole	0.206 and -0.304 e.$Å^{-3}$	

Atomic coordinates (x 10^4) and equivalent isotropic displacement parameters (Å^{2}x 10^3) for **11ad**. U(eq) is defined as one third of the trace of the orthogonalized U^{ij} tensor.

	x	y	z	U(eq)
Br(1)	6507(1)	1272(1)	2134(1)	36(1)
O(1)	3430(1)	1775(3)	976(3)	29(1)
O(2)	4123(1)	4853(3)	4697(2)	22(1)
O(3)	5129(1)	2377(3)	7923(3)	36(1)
O(4)	5455(1)	4680(3)	8448(3)	28(1)
C(1)	2822(2)	4809(5)	528(4)	29(1)
C(2)	2648(2)	6279(5)	594(4)	32(1)
C(3)	2879(2)	7180(5)	1658(4)	31(1)
C(4)	3283(2)	6664(5)	2697(4)	26(1)
C(5)	3451(1)	5215(4)	2627(4)	22(1)
C(6)	3226(1)	4300(4)	1539(4)	22(1)
C(7)	3508(1)	2816(4)	1758(3)	22(1)
C(8)	3883(1)	2898(4)	3074(3)	19(1)
C(9)	3844(1)	4290(4)	3534(3)	19(1)
C(10)	4415(1)	3852(4)	5613(3)	20(1)
C(11)	4267(1)	2255(4)	5383(4)	20(1)
C(12)	4241(1)	1753(4)	3905(3)	18(1)
C(13)	3976(2)	199(4)	3729(4)	25(1)
C(14)	4809(1)	1674(4)	3511(3)	19(1)
C(15)	5297(2)	1693(4)	4447(4)	25(1)
C(16)	5805(2)	1558(4)	4047(4)	26(1)
C(17)	5817(2)	1403(4)	2694(4)	26(1)
C(18)	5337(2)	1356(4)	1735(4)	23(1)
C(19)	4839(2)	1492(4)	2157(4)	22(1)
C(20)	4768(1)	4486(4)	6612(4)	24(1)
C(21)	5120(1)	3697(4)	7701(4)	24(1)
C(22)	5889(2)	4089(5)	9480(4)	31(1)
C(23)	6411(1)	3996(4)	8928(3)	25(1)
C(24)	6486(2)	2898(4)	8013(4)	28(1)
C(25)	6955(2)	2872(5)	7467(4)	32(1)
C(26)	7364(2)	3916(5)	7847(4)	35(1)
C(27)	7302(2)	4983(5)	8778(4)	38(1)
C(28)	6825(2)	5025(4)	9305(4)	32(1)

Bond lengths [Å] and angles [°] for **11ad**.

Br(1)-C(17)	1.903(3)
O(1)-C(7)	1.220(4)
O(2)-C(9)	1.347(4)
O(2)-C(10)	1.395(4)
O(3)-C(21)	1.212(5)
O(4)-C(21)	1.346(4)
O(4)-C(22)	1.452(4)
C(1)-H(1)	0.85(4)
C(1)-C(2)	1.401(6)
C(1)-C(6)	1.368(5)
C(2)-H(2)	0.9500
C(2)-C(3)	1.383(6)
C(3)-H(3)	0.9500
C(3)-C(4)	1.388(6)
C(4)-H(4)	0.98(5)
C(4)-C(5)	1.378(6)
C(5)-C(6)	1.404(5)
C(5)-C(9)	1.467(5)
C(6)-C(7)	1.507(5)
C(7)-C(8)	1.475(5)
C(8)-C(9)	1.349(5)
C(8)-C(12)	1.510(5)
C(10)-C(11)	1.494(5)
C(10)-C(20)	1.335(5)
C(11)-H(11A)	0.9900
C(11)-H(11B)	0.9900
C(11)-C(12)	1.551(5)
C(12)-C(13)	1.544(5)
C(12)-C(14)	1.534(5)
C(13)-H(13A)	0.9800
C(13)-H(13B)	0.9800
C(13)-H(13C)	0.9800
C(14)-C(15)	1.388(5)
C(14)-C(19)	1.394(5)
C(15)-H(15)	0.9500
C(15)-C(16)	1.397(5)

C(16)-H(16)	0.9500
C(16)-C(17)	1.381(5)
C(17)-C(18)	1.386(5)
C(18)-H(18)	0.9500
C(18)-C(19)	1.384(5)
C(19)-H(19)	0.9500
C(20)-H(20)	0.9500
C(20)-C(21)	1.455(5)
C(22)-H(22A)	0.9900
C(22)-H(22B)	0.9900
C(22)-C(23)	1.505(5)
C(23)-C(24)	1.392(5)
C(23)-C(28)	1.382(5)
C(24)-H(24)	0.9500
C(24)-C(25)	1.377(5)
C(25)-H(25)	0.9500
C(25)-C(26)	1.384(6)
C(26)-H(26)	0.9500
C(26)-C(27)	1.375(6)
C(27)-H(27)	0.9500
C(27)-C(28)	1.383(5)
C(28)-H(28)	0.9500

C(9)-O(2)-C(10)	116.9(3)
C(21)-O(4)-C(22)	117.2(3)
C(2)-C(1)-H(1)	125(3)
C(6)-C(1)-H(1)	117(3)
C(6)-C(1)-C(2)	118.1(4)
C(1)-C(2)-H(2)	119.7
C(3)-C(2)-C(1)	120.5(4)
C(3)-C(2)-H(2)	119.7
C(2)-C(3)-H(3)	119.1
C(2)-C(3)-C(4)	121.7(4)
C(4)-C(3)-H(3)	119.1
C(3)-C(4)-H(4)	124(3)
C(5)-C(4)-C(3)	117.5(4)
C(5)-C(4)-H(4)	119(3)
C(4)-C(5)-C(6)	121.3(3)

C(4)-C(5)-C(9)	132.8(4)
C(6)-C(5)-C(9)	105.9(3)
C(1)-C(6)-C(5)	121.0(3)
C(1)-C(6)-C(7)	131.4(3)
C(5)-C(6)-C(7)	107.6(3)
O(1)-C(7)-C(6)	125.2(3)
O(1)-C(7)-C(8)	128.2(3)
C(8)-C(7)-C(6)	106.5(3)
C(7)-C(8)-C(12)	132.3(3)
C(9)-C(8)-C(7)	106.5(3)
C(9)-C(8)-C(12)	121.2(3)
O(2)-C(9)-C(5)	120.2(3)
O(2)-C(9)-C(8)	126.4(3)
C(8)-C(9)-C(5)	113.4(3)
O(2)-C(10)-C(11)	116.1(3)
C(20)-C(10)-O(2)	114.1(3)
C(20)-C(10)-C(11)	129.7(3)
C(10)-C(11)-H(11A)	108.9
C(10)-C(11)-H(11B)	108.9
C(10)-C(11)-C(12)	113.4(3)
H(11A)-C(11)-H(11B)	107.7
C(12)-C(11)-H(11A)	108.9
C(12)-C(11)-H(11B)	108.9
C(8)-C(12)-C(11)	104.5(3)
C(8)-C(12)-C(13)	111.4(3)
C(8)-C(12)-C(14)	111.0(3)
C(13)-C(12)-C(11)	108.5(3)
C(14)-C(12)-C(11)	112.9(3)
C(14)-C(12)-C(13)	108.5(3)
C(12)-C(13)-H(13A)	109.5
C(12)-C(13)-H(13B)	109.5
C(12)-C(13)-H(13C)	109.5
H(13A)-C(13)-H(13B)	109.5
H(13A)-C(13)-H(13C)	109.5
H(13B)-C(13)-H(13C)	109.5
C(15)-C(14)-C(12)	123.1(3)
C(15)-C(14)-C(19)	118.2(3)
C(19)-C(14)-C(12)	118.6(3)

C(14)-C(15)-H(15)	119.4
C(14)-C(15)-C(16)	121.1(4)
C(16)-C(15)-H(15)	119.4
C(15)-C(16)-H(16)	120.5
C(17)-C(16)-C(15)	118.9(3)
C(17)-C(16)-H(16)	120.5
C(16)-C(17)-Br(1)	119.2(3)
C(16)-C(17)-C(18)	121.4(3)
C(18)-C(17)-Br(1)	119.4(3)
C(17)-C(18)-H(18)	120.7
C(19)-C(18)-C(17)	118.7(3)
C(19)-C(18)-H(18)	120.7
C(14)-C(19)-H(19)	119.1
C(18)-C(19)-C(14)	121.7(4)
C(18)-C(19)-H(19)	119.1
C(10)-C(20)-H(20)	117.4
C(10)-C(20)-C(21)	125.2(3)
C(21)-C(20)-H(20)	117.4
O(3)-C(21)-O(4)	123.7(3)
O(3)-C(21)-C(20)	127.6(3)
O(4)-C(21)-C(20)	108.7(3)
O(4)-C(22)-H(22A)	109.9
O(4)-C(22)-H(22B)	109.9
O(4)-C(22)-C(23)	109.1(3)
H(22A)-C(22)-H(22B)	108.3
C(23)-C(22)-H(22A)	109.9
C(23)-C(22)-H(22B)	109.9
C(24)-C(23)-C(22)	121.1(3)
C(28)-C(23)-C(22)	120.3(3)
C(28)-C(23)-C(24)	118.5(3)
C(23)-C(24)-H(24)	119.8
C(25)-C(24)-C(23)	120.4(4)
C(25)-C(24)-H(24)	119.8
C(24)-C(25)-H(25)	119.8
C(24)-C(25)-C(26)	120.4(4)
C(26)-C(25)-H(25)	119.8
C(25)-C(26)-H(26)	120.1
C(27)-C(26)-C(25)	119.8(4)

C(27)-C(26)-H(26)	120.1
C(26)-C(27)-H(27)	120.1
C(26)-C(27)-C(28)	119.7(4)
C(28)-C(27)-H(27)	120.1
C(23)-C(28)-C(27)	121.2(4)
C(23)-C(28)-H(28)	119.4
C(27)-C(28)-H(28)	119.4

Symmetry transformations used to generate equivalent atoms:

Anisotropic displacement parameters ($Å^2$x 10^3) for **11ad**. The anisotropic displacement factor exponent takes the form: $-2\pi^2[h^2 a^{*2}U^{11} + ... + 2 h k a^* b^* U^{12}]$

	U^{11}	U^{22}	U^{33}	U^{23}	U^{13}	U^{12}
Br(1)	26(1)	39(1)	47(1)	3(1)	17(1)	4(1)
O(1)	32(1)	29(1)	25(1)	-5(1)	1(1)	1(1)
O(2)	23(1)	17(1)	24(1)	-1(1)	-1(1)	3(1)
O(3)	41(2)	25(2)	36(2)	4(1)	-7(1)	-5(1)
O(4)	22(1)	31(2)	29(1)	-5(1)	-3(1)	0(1)
C(1)	26(2)	39(2)	23(2)	1(2)	6(2)	5(2)
C(2)	28(2)	44(3)	27(2)	13(2)	9(2)	12(2)
C(3)	31(2)	31(2)	33(2)	10(2)	11(2)	12(2)
C(4)	28(2)	25(2)	29(2)	6(2)	14(2)	5(2)
C(5)	19(2)	26(2)	24(2)	4(2)	9(1)	2(1)
C(6)	21(2)	24(2)	23(2)	1(2)	7(1)	2(1)
C(7)	20(2)	26(2)	20(2)	2(1)	7(1)	-1(1)
C(8)	18(2)	20(2)	20(2)	0(1)	6(1)	-2(1)
C(9)	18(2)	21(2)	19(2)	2(1)	5(1)	2(1)
C(10)	19(2)	20(2)	20(2)	1(1)	6(1)	2(1)
C(11)	21(2)	19(2)	21(2)	-1(1)	6(1)	-2(1)
C(12)	19(2)	16(2)	19(2)	-1(1)	3(1)	-2(1)
C(13)	25(2)	20(2)	29(2)	1(2)	4(2)	-4(1)
C(14)	25(2)	12(2)	22(2)	1(1)	5(1)	1(1)
C(15)	26(2)	22(2)	26(2)	2(2)	5(2)	1(1)
C(16)	21(2)	28(2)	30(2)	2(2)	2(2)	3(1)
C(17)	23(2)	19(2)	37(2)	3(2)	12(2)	3(1)
C(18)	30(2)	17(2)	23(2)	0(1)	9(2)	-1(1)
C(19)	24(2)	20(2)	23(2)	1(2)	4(2)	0(1)
C(20)	23(2)	19(2)	29(2)	-4(2)	6(2)	3(2)
C(21)	22(2)	26(2)	24(2)	-4(2)	4(1)	-3(2)
C(22)	26(2)	41(2)	24(2)	-2(2)	-1(2)	1(2)
C(23)	25(2)	32(2)	16(2)	5(2)	-2(1)	1(2)
C(24)	30(2)	27(2)	26(2)	0(2)	-2(2)	2(2)
C(25)	36(2)	35(2)	25(2)	1(2)	3(2)	5(2)
C(26)	29(2)	41(2)	37(2)	10(2)	9(2)	6(2)
C(27)	29(2)	40(2)	43(3)	4(2)	4(2)	-5(2)
C(28)	32(2)	32(2)	30(2)	-3(2)	1(2)	-2(2)

Hydrogen coordinates (x 10^4) and isotropic displacement parameters (Å^{2}x 10^3) for **11ad**.

	x	y	z	U(eq)
H(1)	2688(17)	4200(50)	-90(40)	28(11)
H(2)	2369	6661	-96	39
H(3)	2758	8177	1677	37
H(4)	3431(18)	7240(50)	3510(50)	41(12)
H(11A)	3905	2076	5635	24
H(11B)	4542	1642	5980	24
H(13A)	4223	-523	4257	37
H(13B)	3913	-81	2776	37
H(13C)	3625	214	4042	37
H(15)	5285	1799	5377	30
H(16)	6137	1573	4694	32
H(18)	5350	1234	808	27
H(19)	4508	1460	1506	27
H(20)	4792	5537	6611	28
H(22A)	5785	3092	9755	37
H(22B)	5944	4741	10282	37
H(24)	6212	2163	7763	34
H(25)	6999	2133	6826	39
H(26)	7687	3896	7466	42
H(27)	7585	5688	9059	45
H(28)	6781	5774	9937	38

Torsion angles [°] for **11ad**.

Br(1)-C(17)-C(18)-C(19)	-178.3(3)
O(1)-C(7)-C(8)-C(9)	177.4(3)
O(1)-C(7)-C(8)-C(12)	-5.8(6)
O(2)-C(10)-C(11)-C(12)	48.3(4)
O(2)-C(10)-C(20)-C(21)	179.6(3)
O(4)-C(22)-C(23)-C(24)	-74.8(4)
O(4)-C(22)-C(23)-C(28)	104.0(4)
C(1)-C(2)-C(3)-C(4)	-0.8(6)
C(1)-C(6)-C(7)-O(1)	3.6(6)
C(1)-C(6)-C(7)-C(8)	-176.4(4)
C(2)-C(1)-C(6)-C(5)	1.3(5)
C(2)-C(1)-C(6)-C(7)	-178.8(4)
C(2)-C(3)-C(4)-C(5)	0.6(6)
C(3)-C(4)-C(5)-C(6)	0.6(5)
C(3)-C(4)-C(5)-C(9)	-177.6(4)
C(4)-C(5)-C(6)-C(1)	-1.6(5)
C(4)-C(5)-C(6)-C(7)	178.5(3)
C(4)-C(5)-C(9)-O(2)	-0.4(6)
C(4)-C(5)-C(9)-C(8)	179.7(4)
C(5)-C(6)-C(7)-O(1)	-176.6(3)
C(5)-C(6)-C(7)-C(8)	3.4(4)
C(6)-C(1)-C(2)-C(3)	-0.2(6)
C(6)-C(5)-C(9)-O(2)	-178.8(3)
C(6)-C(5)-C(9)-C(8)	1.3(4)
C(6)-C(7)-C(8)-C(9)	-2.6(4)
C(6)-C(7)-C(8)-C(12)	174.2(3)
C(7)-C(8)-C(9)-O(2)	-179.0(3)
C(7)-C(8)-C(9)-C(5)	0.9(4)
C(7)-C(8)-C(12)-C(11)	-150.0(3)
C(7)-C(8)-C(12)-C(13)	-33.0(5)
C(7)-C(8)-C(12)-C(14)	88.1(4)
C(8)-C(12)-C(14)-C(15)	131.7(3)
C(8)-C(12)-C(14)-C(19)	-52.0(4)
C(9)-O(2)-C(10)-C(11)	-16.0(4)
C(9)-O(2)-C(10)-C(20)	167.2(3)
C(9)-C(5)-C(6)-C(1)	177.0(3)

C(9)-C(5)-C(6)-C(7)	-2.8(4)
C(9)-C(8)-C(12)-C(11)	26.5(4)
C(9)-C(8)-C(12)-C(13)	143.4(3)
C(9)-C(8)-C(12)-C(14)	-95.5(4)
C(10)-O(2)-C(9)-C(5)	169.2(3)
C(10)-O(2)-C(9)-C(8)	-10.9(5)
C(10)-C(11)-C(12)-C(8)	-49.9(3)
C(10)-C(11)-C(12)-C(13)	-168.9(3)
C(10)-C(11)-C(12)-C(14)	70.8(4)
C(10)-C(20)-C(21)-O(3)	-4.4(6)
C(10)-C(20)-C(21)-O(4)	174.5(3)
C(11)-C(10)-C(20)-C(21)	3.3(6)
C(11)-C(12)-C(14)-C(15)	14.7(5)
C(11)-C(12)-C(14)-C(19)	-168.9(3)
C(12)-C(8)-C(9)-O(2)	3.7(5)
C(12)-C(8)-C(9)-C(5)	-176.3(3)
C(12)-C(14)-C(15)-C(16)	177.4(3)
C(12)-C(14)-C(19)-C(18)	-177.6(3)
C(13)-C(12)-C(14)-C(15)	-105.5(4)
C(13)-C(12)-C(14)-C(19)	70.8(4)
C(14)-C(15)-C(16)-C(17)	0.0(5)
C(15)-C(14)-C(19)-C(18)	-1.1(5)
C(15)-C(16)-C(17)-Br(1)	178.3(3)
C(15)-C(16)-C(17)-C(18)	-1.0(5)
C(16)-C(17)-C(18)-C(19)	1.0(5)
C(17)-C(18)-C(19)-C(14)	0.1(5)
C(19)-C(14)-C(15)-C(16)	1.0(5)
C(20)-C(10)-C(11)-C(12)	-135.5(4)
C(21)-O(4)-C(22)-C(23)	96.1(4)
C(22)-O(4)-C(21)-O(3)	7.0(5)
C(22)-O(4)-C(21)-C(20)	-171.9(3)
C(22)-C(23)-C(24)-C(25)	176.8(3)
C(22)-C(23)-C(28)-C(27)	-178.1(4)
C(23)-C(24)-C(25)-C(26)	1.5(6)
C(24)-C(23)-C(28)-C(27)	0.7(6)
C(24)-C(25)-C(26)-C(27)	0.2(6)
C(25)-C(26)-C(27)-C(28)	-1.5(6)
C(26)-C(27)-C(28)-C(23)	1.0(6)

C(28)-C(23)-C(24)-C(25)	-2.0(5)

Symmetry transformations used to generate equivalent atoms:

CCDC 2479639 (13ac)

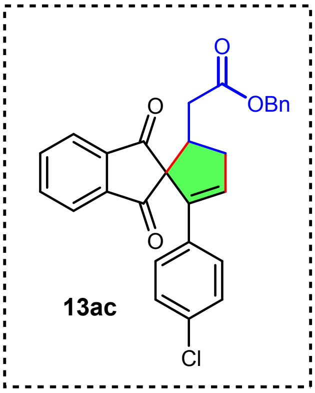

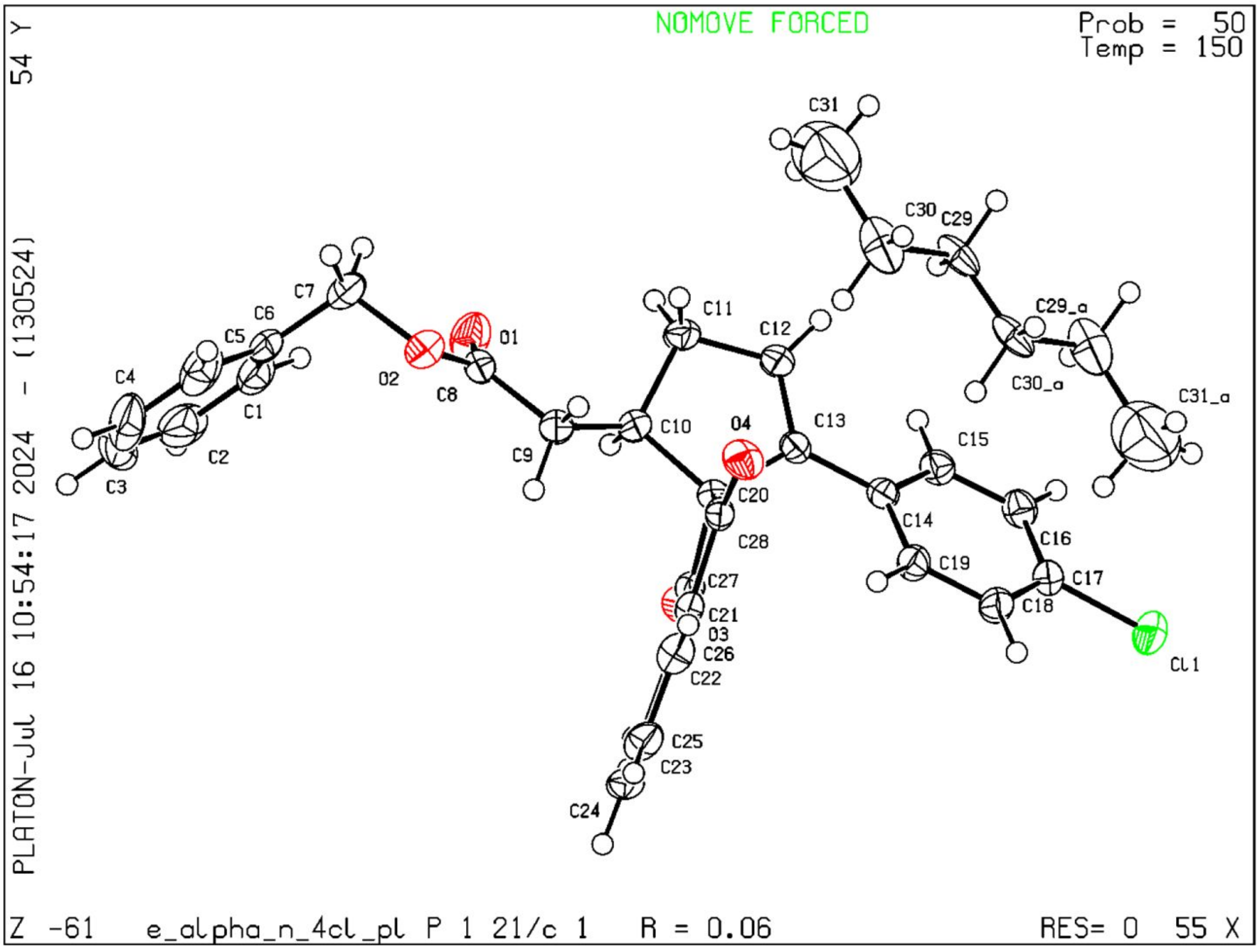

ORTEP drawing of **13ac** showing thermal ellipsoids at the 50% probability level

The crystal was obtained by slow evaporation of **13ac** in ether at room temperature.

Crystal data and structure refinement for **13ac**

Identification code	e_alpha_n_4cl_pl	
Empirical formula	C31 H28 Cl O4	
Formula weight	499.98	
Temperature	150.15 K	
Wavelength	0.71076 Å	
Crystal system	Monoclinic	
Space group	P 1 21/c 1	
Unit cell dimensions	a = 11.3576(8) Å	α= 90°.
	b = 10.4827(7) Å	β= 101.732(4)°.
	c = 21.1999(16) Å	γ = 90°.
Volume	2471.3(3) $Å^3$	
Z	4	
Density (calculated)	1.344 Mg/m^3	
Absorption coefficient	0.191 mm^{-1}	
F(000)	1052	
Crystal size	? x ? x ? mm^3	
Theta range for data collection	2.971 to 25.025°.	
Index ranges	-13<=h<=13, -12<=k<=12, -25<=l<=25	
Reflections collected	61513	
Independent reflections	4339 [R(int) = 0.0383]	
Completeness to theta = 25.025°	99.3 %	
Absorption correction	None	
Refinement method	Full-matrix least-squares on F^2	
Data / restraints / parameters	4339 / 0 / 326	
Goodness-of-fit on F^2	1.062	
Final R indices [I>2sigma(I)]	R1 = 0.0592, wR2 = 0.1645	
R indices (all data)	R1 = 0.0647, wR2 = 0.1717	
Extinction coefficient	n/a	
Largest diff. peak and hole	0.631 and -0.621 e.$Å^{-3}$	

Atomic coordinates (x 10^4) and equivalent isotropic displacement parameters (Å^{2}x 10^3) For **13ac**. U(eq) is defined as one third of the trace of the orthogonalized U^{ij} tensor.

	x	y	z	U(eq)
Cl(1)	685(1)	1867(1)	-1561(1)	42(1)
O(1)	2390(2)	6810(2)	3096(1)	44(1)
O(2)	3908(2)	5797(2)	3748(1)	35(1)
O(3)	216(2)	3431(2)	1537(1)	34(1)
O(4)	4434(2)	3149(2)	1803(1)	35(1)
C(1)	1978(3)	6842(3)	4553(1)	39(1)
C(2)	1270(4)	6568(3)	4988(2)	60(1)
C(3)	1747(6)	6023(3)	5569(2)	79(2)
C(4)	2937(6)	5739(3)	5715(2)	76(2)
C(5)	3669(4)	6005(3)	5280(2)	51(1)
C(6)	3183(3)	6559(2)	4695(1)	34(1)
C(7)	3975(3)	6833(3)	4222(1)	38(1)
C(8)	3071(2)	5916(2)	3213(1)	27(1)
C(9)	3098(2)	4798(2)	2774(1)	29(1)
C(10)	2436(2)	5064(2)	2090(1)	28(1)
C(11)	2942(3)	6140(2)	1738(1)	32(1)
C(12)	2631(2)	5734(2)	1050(1)	29(1)
C(13)	2293(2)	4520(2)	975(1)	23(1)
C(14)	1919(2)	3849(2)	353(1)	24(1)
C(15)	1325(2)	4518(2)	-186(1)	28(1)
C(16)	959(2)	3926(2)	-775(1)	30(1)
C(17)	1188(2)	2636(3)	-827(1)	28(1)
C(18)	1780(3)	1936(2)	-304(1)	31(1)
C(19)	2136(2)	2553(2)	284(1)	29(1)
C(20)	2347(2)	3876(2)	1624(1)	23(1)
C(21)	1248(2)	3079(2)	1688(1)	22(1)
C(22)	1671(2)	1842(2)	1989(1)	21(1)
C(23)	1013(2)	870(2)	2202(1)	29(1)
C(24)	1637(3)	-177(2)	2497(1)	35(1)
C(25)	2881(3)	-262(2)	2569(1)	35(1)
C(26)	3529(2)	699(2)	2356(1)	31(1)
C(27)	2917(2)	1760(2)	2069(1)	22(1)
C(28)	3394(2)	2937(2)	1824(1)	23(1)

C(29)	5062(2)	5654(3)	146(1)	35(1)
C(30)	5725(3)	5570(4)	803(2)	55(1)
C(31)	5842(6)	7030(7)	1087(3)	117(2)

Bond lengths [Å] and angles [°] for **13ac**

Cl(1)-C(17)	1.742(2)
O(1)-C(8)	1.208(3)
O(2)-C(7)	1.472(3)
O(2)-C(8)	1.329(3)
O(3)-C(21)	1.209(3)
O(4)-C(28)	1.212(3)
C(1)-H(1)	0.9500
C(1)-C(2)	1.371(5)
C(1)-C(6)	1.373(4)
C(2)-H(2)	0.9500
C(2)-C(3)	1.367(7)
C(3)-H(3)	0.9500
C(3)-C(4)	1.356(7)
C(4)-H(4)	0.9500
C(4)-C(5)	1.390(6)
C(5)-H(5)	0.9500
C(5)-C(6)	1.379(4)
C(6)-C(7)	1.504(4)
C(7)-H(7A)	0.9900
C(7)-H(7B)	0.9900
C(8)-C(9)	1.501(3)
C(9)-H(9A)	0.9900
C(9)-H(9B)	0.9900
C(9)-C(10)	1.518(3)
C(10)-H(10)	1.0000
C(10)-C(11)	1.528(3)
C(10)-C(20)	1.579(3)
C(11)-H(11A)	0.9900
C(11)-H(11B)	0.9900
C(11)-C(12)	1.492(4)
C(12)-H(12)	0.9500
C(12)-C(13)	1.330(3)
C(13)-C(14)	1.478(3)
C(13)-C(20)	1.523(3)
C(14)-C(15)	1.393(3)
C(14)-C(19)	1.394(4)

C(15)-H(15)	0.9500
C(15)-C(16)	1.381(4)
C(16)-H(16)	0.9500
C(16)-C(17)	1.385(4)
C(17)-C(18)	1.385(4)
C(18)-H(18)	0.9500
C(18)-C(19)	1.389(4)
C(19)-H(19)	0.9500
C(20)-C(21)	1.531(3)
C(20)-C(28)	1.535(3)
C(21)-C(22)	1.481(3)
C(22)-C(23)	1.392(3)
C(22)-C(27)	1.393(3)
C(23)-H(23)	0.9500
C(23)-C(24)	1.386(4)
C(24)-H(24)	0.9500
C(24)-C(25)	1.392(4)
C(25)-H(25)	0.9500
C(25)-C(26)	1.377(4)
C(26)-H(26)	0.9500
C(26)-C(27)	1.386(3)
C(27)-C(28)	1.482(3)
C(29)-C(29)#1	1.499(6)
C(29)-H(29A)	0.9900
C(29)-H(29B)	0.9900
C(29)-C(30)	1.446(4)
C(30)-H(30A)	0.9900
C(30)-H(30B)	0.9900
C(30)-C(31)	1.640(8)
C(31)-H(31A)	0.9800
C(31)-H(31B)	0.9800
C(31)-H(31C)	0.9800

C(8)-O(2)-C(7)	116.3(2)
C(2)-C(1)-H(1)	119.9
C(2)-C(1)-C(6)	120.2(3)
C(6)-C(1)-H(1)	119.9
C(1)-C(2)-H(2)	119.5

C(3)-C(2)-C(1)	121.0(4)
C(3)-C(2)-H(2)	119.5
C(2)-C(3)-H(3)	120.3
C(4)-C(3)-C(2)	119.4(3)
C(4)-C(3)-H(3)	120.3
C(3)-C(4)-H(4)	119.7
C(3)-C(4)-C(5)	120.5(3)
C(5)-C(4)-H(4)	119.7
C(4)-C(5)-H(5)	120.1
C(6)-C(5)-C(4)	119.8(4)
C(6)-C(5)-H(5)	120.1
C(1)-C(6)-C(5)	119.1(3)
C(1)-C(6)-C(7)	121.2(3)
C(5)-C(6)-C(7)	119.7(3)
O(2)-C(7)-C(6)	111.1(2)
O(2)-C(7)-H(7A)	109.4
O(2)-C(7)-H(7B)	109.4
C(6)-C(7)-H(7A)	109.4
C(6)-C(7)-H(7B)	109.4
H(7A)-C(7)-H(7B)	108.0
O(1)-C(8)-O(2)	124.4(2)
O(1)-C(8)-C(9)	124.9(2)
O(2)-C(8)-C(9)	110.8(2)
C(8)-C(9)-H(9A)	109.1
C(8)-C(9)-H(9B)	109.1
C(8)-C(9)-C(10)	112.4(2)
H(9A)-C(9)-H(9B)	107.9
C(10)-C(9)-H(9A)	109.1
C(10)-C(9)-H(9B)	109.1
C(9)-C(10)-H(10)	107.0
C(9)-C(10)-C(11)	116.1(2)
C(9)-C(10)-C(20)	114.1(2)
C(11)-C(10)-H(10)	107.0
C(11)-C(10)-C(20)	105.05(19)
C(20)-C(10)-H(10)	107.0
C(10)-C(11)-H(11A)	111.1
C(10)-C(11)-H(11B)	111.1
H(11A)-C(11)-H(11B)	109.1

C(12)-C(11)-C(10)	103.2(2)
C(12)-C(11)-H(11A)	111.1
C(12)-C(11)-H(11B)	111.1
C(11)-C(12)-H(12)	123.4
C(13)-C(12)-C(11)	113.3(2)
C(13)-C(12)-H(12)	123.4
C(12)-C(13)-C(14)	125.9(2)
C(12)-C(13)-C(20)	111.0(2)
C(14)-C(13)-C(20)	123.1(2)
C(15)-C(14)-C(13)	119.7(2)
C(15)-C(14)-C(19)	118.1(2)
C(19)-C(14)-C(13)	122.2(2)
C(14)-C(15)-H(15)	119.3
C(16)-C(15)-C(14)	121.4(2)
C(16)-C(15)-H(15)	119.3
C(15)-C(16)-H(16)	120.5
C(15)-C(16)-C(17)	119.0(2)
C(17)-C(16)-H(16)	120.5
C(16)-C(17)-Cl(1)	119.3(2)
C(18)-C(17)-Cl(1)	119.2(2)
C(18)-C(17)-C(16)	121.5(2)
C(17)-C(18)-H(18)	120.8
C(17)-C(18)-C(19)	118.5(2)
C(19)-C(18)-H(18)	120.8
C(14)-C(19)-H(19)	119.2
C(18)-C(19)-C(14)	121.5(2)
C(18)-C(19)-H(19)	119.2
C(13)-C(20)-C(10)	101.62(18)
C(13)-C(20)-C(21)	115.95(19)
C(13)-C(20)-C(28)	114.87(19)
C(21)-C(20)-C(10)	108.96(19)
C(21)-C(20)-C(28)	102.97(18)
C(28)-C(20)-C(10)	112.71(19)
O(3)-C(21)-C(20)	125.0(2)
O(3)-C(21)-C(22)	126.4(2)
C(22)-C(21)-C(20)	108.53(19)
C(23)-C(22)-C(21)	129.2(2)
C(23)-C(22)-C(27)	121.0(2)

C(27)-C(22)-C(21)	109.7(2)
C(22)-C(23)-H(23)	121.1
C(24)-C(23)-C(22)	117.8(2)
C(24)-C(23)-H(23)	121.1
C(23)-C(24)-H(24)	119.5
C(23)-C(24)-C(25)	121.0(2)
C(25)-C(24)-H(24)	119.5
C(24)-C(25)-H(25)	119.5
C(26)-C(25)-C(24)	121.0(2)
C(26)-C(25)-H(25)	119.5
C(25)-C(26)-H(26)	120.7
C(25)-C(26)-C(27)	118.5(2)
C(27)-C(26)-H(26)	120.7
C(22)-C(27)-C(28)	109.9(2)
C(26)-C(27)-C(22)	120.6(2)
C(26)-C(27)-C(28)	129.4(2)
O(4)-C(28)-C(20)	125.3(2)
O(4)-C(28)-C(27)	126.4(2)
C(27)-C(28)-C(20)	108.23(19)
C(29)#1-C(29)-H(29A)	109.8
C(29)#1-C(29)-H(29B)	109.8
H(29A)-C(29)-H(29B)	108.3
C(30)-C(29)-C(29)#1	109.2(3)
C(30)-C(29)-H(29A)	109.8
C(30)-C(29)-H(29B)	109.8
C(29)-C(30)-H(30A)	110.4
C(29)-C(30)-H(30B)	110.4
C(29)-C(30)-C(31)	106.5(3)
H(30A)-C(30)-H(30B)	108.6
C(31)-C(30)-H(30A)	110.4
C(31)-C(30)-H(30B)	110.4
C(30)-C(31)-H(31A)	109.5
C(30)-C(31)-H(31B)	109.5
C(30)-C(31)-H(31C)	109.5
H(31A)-C(31)-H(31B)	109.5
H(31A)-C(31)-H(31C)	109.5
H(31B)-C(31)-H(31C)	109.5

Anisotropic displacement parameters ($Å^2$x 10^3) for **13ac**. The anisotropic displacement factor exponent takes the form: $-2 \pi ^2[h^2 a^{*2}U^{11} + ... + 2 h k a^* b^* U^{12}]$

	U^{11}	U^{22}	U^{33}	U^{23}	U^{13}	U^{12}
Cl(1)	56(1)	43(1)	25(1)	-7(1)	5(1)	-6(1)
O(1)	59(1)	34(1)	38(1)	-4(1)	8(1)	15(1)
O(2)	35(1)	33(1)	35(1)	-5(1)	4(1)	-2(1)
O(3)	24(1)	32(1)	44(1)	6(1)	5(1)	6(1)
O(4)	23(1)	44(1)	39(1)	1(1)	10(1)	-2(1)
C(1)	54(2)	29(1)	33(1)	-7(1)	6(1)	1(1)
C(2)	60(2)	49(2)	81(3)	-29(2)	38(2)	-13(2)
C(3)	159(5)	32(2)	71(3)	-16(2)	83(3)	-25(2)
C(4)	173(5)	27(2)	28(2)	4(1)	24(2)	12(2)
C(5)	77(2)	34(2)	39(2)	-8(1)	1(2)	14(2)
C(6)	54(2)	20(1)	31(1)	-9(1)	12(1)	-5(1)
C(7)	44(2)	34(2)	38(2)	-14(1)	9(1)	-10(1)
C(8)	32(1)	26(1)	25(1)	0(1)	11(1)	-2(1)
C(9)	37(1)	24(1)	27(1)	0(1)	9(1)	-1(1)
C(10)	35(1)	22(1)	28(1)	-2(1)	9(1)	-2(1)
C(11)	47(2)	21(1)	28(1)	1(1)	5(1)	-6(1)
C(12)	39(1)	23(1)	25(1)	3(1)	6(1)	-3(1)
C(13)	25(1)	22(1)	23(1)	2(1)	7(1)	1(1)
C(14)	25(1)	22(1)	25(1)	1(1)	8(1)	-1(1)
C(15)	33(1)	23(1)	29(1)	3(1)	9(1)	4(1)
C(16)	32(1)	31(1)	26(1)	5(1)	5(1)	4(1)
C(17)	32(1)	32(1)	22(1)	-2(1)	8(1)	-4(1)
C(18)	44(2)	22(1)	28(1)	0(1)	9(1)	2(1)
C(19)	38(1)	24(1)	23(1)	2(1)	5(1)	2(1)
C(20)	26(1)	20(1)	23(1)	0(1)	6(1)	-1(1)
C(21)	25(1)	21(1)	21(1)	-2(1)	6(1)	1(1)
C(22)	24(1)	20(1)	19(1)	-2(1)	3(1)	0(1)
C(23)	33(1)	27(1)	27(1)	1(1)	4(1)	-8(1)
C(24)	52(2)	23(1)	28(1)	2(1)	4(1)	-11(1)
C(25)	52(2)	21(1)	27(1)	2(1)	-3(1)	4(1)
C(26)	32(1)	29(1)	29(1)	-1(1)	-1(1)	8(1)
C(27)	25(1)	23(1)	19(1)	-3(1)	4(1)	2(1)
C(28)	24(1)	26(1)	19(1)	-2(1)	5(1)	-1(1)

C(29)	27(1)	49(2)	30(1)	24(1)	7(1)	2(1)
C(30)	40(2)	86(3)	39(2)	13(2)	10(1)	2(2)
C(31)	117(5)	133(6)	98(4)	20(4)	19(4)	-16(4)

Hydrogen coordinates (x 10^4) and isotropic displacement parameters ($Å^2$x 10^3) for **13ac**.

	x	y	z	U(eq)
H(1)	1633	7229	4153	47
H(2)	435	6760	4884	72
H(3)	1250	5843	5869	95
H(4)	3272	5357	6118	91
H(5)	4502	5805	5385	62
H(7A)	3722	7644	3996	46
H(7B)	4817	6932	4457	46
H(9A)	2728	4051	2943	35
H(9B)	3945	4580	2770	35
H(10)	1595	5308	2113	33
H(11A)	2560	6966	1802	39
H(11B)	3823	6218	1888	39
H(12)	2671	6288	700	35
H(15)	1168	5402	-148	33
H(16)	556	4395	-1139	36
H(18)	1938	1053	-346	37
H(19)	2537	2081	647	35
H(23)	163	924	2148	35
H(24)	1211	-848	2652	42
H(25)	3289	-995	2768	42
H(26)	4377	636	2406	37
H(29A)	5492	6219	-106	42
H(29B)	4256	6021	138	42
H(30A)	6532	5201	816	66
H(30B)	5292	5024	1062	66
H(31A)	6123	7595	780	175
H(31B)	6418	7046	1500	175
H(31C)	5055	7321	1151	175

Torsion angles [°] for **4t**.

Cl(1)-C(17)-C(18)-C(19)	-177.7(2)
O(1)-C(8)-C(9)-C(10)	16.3(4)
O(2)-C(8)-C(9)-C(10)	-163.0(2)
O(3)-C(21)-C(22)-C(23)	-4.8(4)
O(3)-C(21)-C(22)-C(27)	177.7(2)
C(1)-C(2)-C(3)-C(4)	-0.5(5)
C(1)-C(6)-C(7)-O(2)	-83.7(3)
C(2)-C(1)-C(6)-C(5)	-0.4(4)
C(2)-C(1)-C(6)-C(7)	179.1(3)
C(2)-C(3)-C(4)-C(5)	0.3(5)
C(3)-C(4)-C(5)-C(6)	-0.1(5)
C(4)-C(5)-C(6)-C(1)	0.1(4)
C(4)-C(5)-C(6)-C(7)	-179.4(3)
C(5)-C(6)-C(7)-O(2)	95.8(3)
C(6)-C(1)-C(2)-C(3)	0.6(5)
C(7)-O(2)-C(8)-O(1)	0.8(4)
C(7)-O(2)-C(8)-C(9)	-179.8(2)
C(8)-O(2)-C(7)-C(6)	90.4(3)
C(8)-C(9)-C(10)-C(11)	62.5(3)
C(8)-C(9)-C(10)-C(20)	-175.1(2)
C(9)-C(10)-C(11)-C(12)	149.6(2)
C(9)-C(10)-C(20)-C(13)	-151.3(2)
C(9)-C(10)-C(20)-C(21)	85.8(2)
C(9)-C(10)-C(20)-C(28)	-27.8(3)
C(10)-C(11)-C(12)-C(13)	-14.1(3)
C(10)-C(20)-C(21)-O(3)	65.0(3)
C(10)-C(20)-C(21)-C(22)	-112.5(2)
C(10)-C(20)-C(28)-O(4)	-67.9(3)
C(10)-C(20)-C(28)-C(27)	109.8(2)
C(11)-C(10)-C(20)-C(13)	-23.0(2)
C(11)-C(10)-C(20)-C(21)	-145.9(2)
C(11)-C(10)-C(20)-C(28)	100.4(2)
C(11)-C(12)-C(13)-C(14)	179.3(2)
C(11)-C(12)-C(13)-C(20)	-1.3(3)
C(12)-C(13)-C(14)-C(15)	-32.5(4)
C(12)-C(13)-C(14)-C(19)	147.8(3)

C(12)-C(13)-C(20)-C(10)	15.4(3)
C(12)-C(13)-C(20)-C(21)	133.4(2)
C(12)-C(13)-C(20)-C(28)	-106.6(2)
C(13)-C(14)-C(15)-C(16)	-179.9(2)
C(13)-C(14)-C(19)-C(18)	-180.0(2)
C(13)-C(20)-C(21)-O(3)	-48.8(3)
C(13)-C(20)-C(21)-C(22)	133.7(2)
C(13)-C(20)-C(28)-O(4)	47.9(3)
C(13)-C(20)-C(28)-C(27)	-134.4(2)
C(14)-C(13)-C(20)-C(10)	-165.1(2)
C(14)-C(13)-C(20)-C(21)	-47.2(3)
C(14)-C(13)-C(20)-C(28)	72.9(3)
C(14)-C(15)-C(16)-C(17)	0.2(4)
C(15)-C(14)-C(19)-C(18)	0.3(4)
C(15)-C(16)-C(17)-Cl(1)	177.88(19)
C(15)-C(16)-C(17)-C(18)	-0.4(4)
C(16)-C(17)-C(18)-C(19)	0.5(4)
C(17)-C(18)-C(19)-C(14)	-0.5(4)
C(19)-C(14)-C(15)-C(16)	-0.1(4)
C(20)-C(10)-C(11)-C(12)	22.6(3)
C(20)-C(13)-C(14)-C(15)	148.1(2)
C(20)-C(13)-C(14)-C(19)	-31.6(3)
C(20)-C(21)-C(22)-C(23)	172.6(2)
C(20)-C(21)-C(22)-C(27)	-4.8(3)
C(21)-C(20)-C(28)-O(4)	174.9(2)
C(21)-C(20)-C(28)-C(27)	-7.4(2)
C(21)-C(22)-C(23)-C(24)	-177.1(2)
C(21)-C(22)-C(27)-C(26)	178.5(2)
C(21)-C(22)-C(27)-C(28)	-0.1(3)
C(22)-C(23)-C(24)-C(25)	-0.9(4)
C(22)-C(27)-C(28)-O(4)	-177.4(2)
C(22)-C(27)-C(28)-C(20)	4.9(3)
C(23)-C(22)-C(27)-C(26)	0.8(3)
C(23)-C(22)-C(27)-C(28)	-177.8(2)
C(23)-C(24)-C(25)-C(26)	0.8(4)
C(24)-C(25)-C(26)-C(27)	0.1(4)
C(25)-C(26)-C(27)-C(22)	-0.9(4)
C(25)-C(26)-C(27)-C(28)	177.4(2)

C(26)-C(27)-C(28)-O(4)	4.2(4)
C(26)-C(27)-C(28)-C(20)	-173.4(2)
C(27)-C(22)-C(23)-C(24)	0.1(3)
C(28)-C(20)-C(21)-O(3)	-175.1(2)
C(28)-C(20)-C(21)-C(22)	7.4(2)
C(29)#1-C(29)-C(30)-C(31)	179.2(4)

Symmetry transformations used to generate equivalent atoms:

CCDC 2479640 (15c)

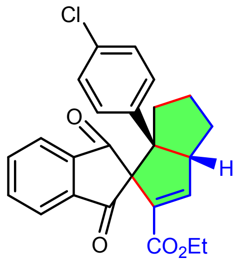

15c

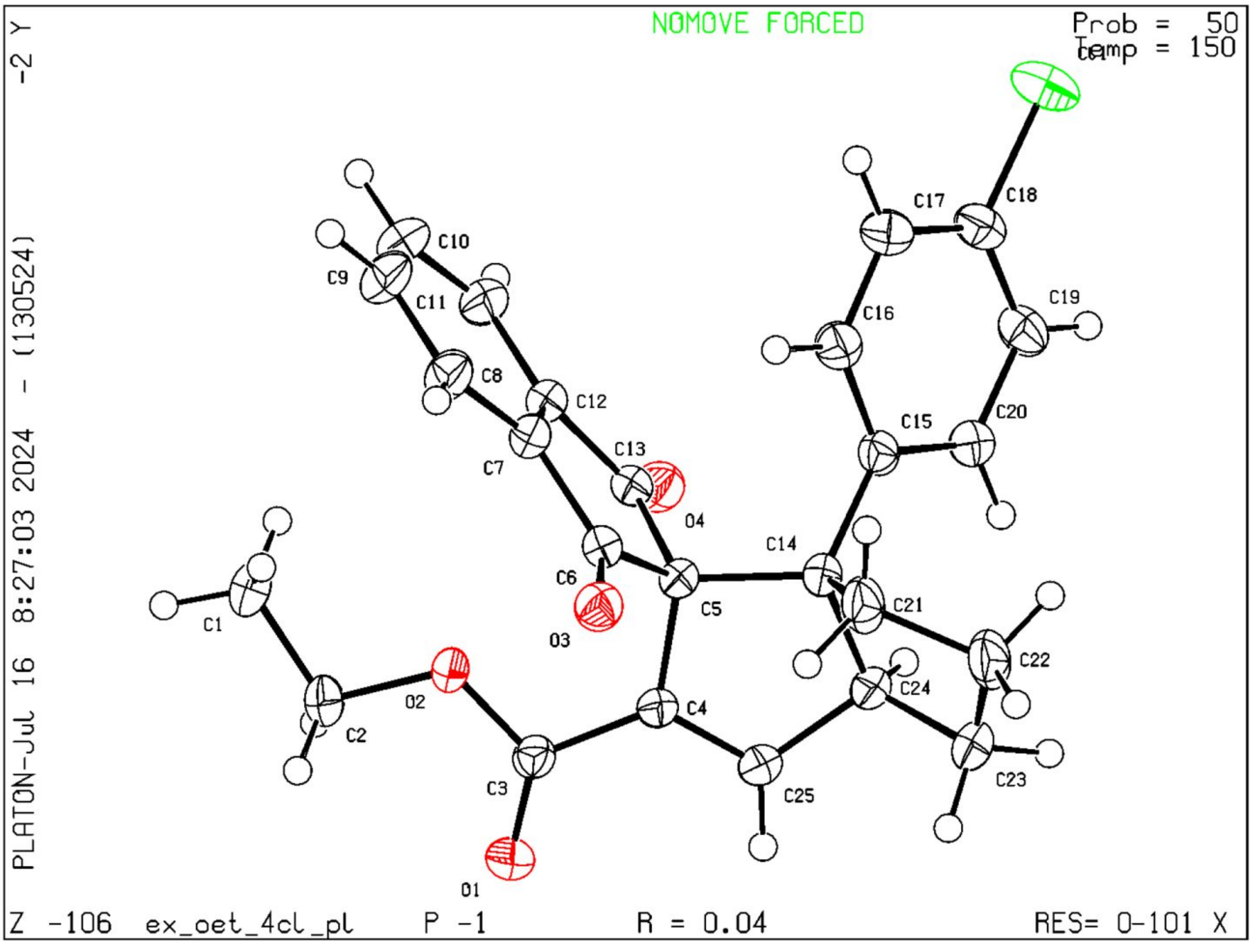

ORTEP drawing of **15c** showing thermal ellipsoids at the 50% probability level

The crystal was obtained by slow evaporation of **15c** in ether at room temperature.

Crystal data and structure refinement for **15c**

Identification code	ex_oet_4cl_pl	
Empirical formula	C25 H21 Cl O4	
Formula weight	420.87	
Temperature	150.15 K	
Wavelength	0.71076 Å	
Crystal system	Triclinic	
Space group	P-1	
Unit cell dimensions	a = 9.7801(4) Å	α= 113.701(2)°.
	b = 10.6063(5) Å	β= 102.788(2)°.
	c = 11.6409(5) Å	γ = 99.061(2)°.
Volume	1036.36(8) $Å^3$	
Z	2	
Density (calculated)	1.349 Mg/m^3	
Absorption coefficient	0.214 mm^{-1}	
F(000)	440	
Crystal size	? x ? x ? mm^3	
Theta range for data collection	2.485 to 26.410°.	
Index ranges	-12<=h<=12, -13<=k<=13, -14<=l<=14	
Reflections collected	23514	
Independent reflections	4236 [R(int) = 0.0308]	
Completeness to theta = 25.243°	99.4 %	
Absorption correction	None	
Refinement method	Full-matrix least-squares on F^2	
Data / restraints / parameters	4236 / 0 / 272	
Goodness-of-fit on F^2	1.067	
Final R indices [I>2sigma(I)]	R1 = 0.0446, wR2 = 0.0947	
R indices (all data)	R1 = 0.0619, wR2 = 0.1119	
Extinction coefficient	n/a	
Largest diff. peak and hole	0.248 and -0.289 e.$Å^{-3}$	

Atomic coordinates (x 10^4) and equivalent isotropic displacement parameters ($Å^2$x 10^3) for **15c**. U(eq) is defined as one third of the trace of the orthogonalized U^{ij} tensor.

	x	y	z	U(eq)
Cl(1)	8108(1)	9483(1)	9473(1)	45(1)
O(1)	-1561(1)	4111(2)	1811(1)	32(1)
O(2)	665(1)	4632(1)	1603(1)	26(1)
O(3)	1688(2)	8143(2)	2768(1)	30(1)
O(4)	2945(2)	5063(2)	4573(1)	33(1)
C(1)	1457(2)	3469(2)	-233(2)	36(1)
C(2)	172(2)	3449(2)	280(2)	30(1)
C(3)	-307(2)	4831(2)	2266(2)	24(1)
C(4)	396(2)	6007(2)	3620(2)	24(1)
C(5)	1980(2)	6858(2)	4107(2)	23(1)
C(6)	2355(2)	7450(2)	3170(2)	25(1)
C(7)	3595(2)	6966(2)	2788(2)	28(1)
C(8)	4290(2)	7246(2)	1958(2)	35(1)
C(9)	5377(2)	6595(3)	1688(2)	43(1)
C(10)	5756(2)	5685(3)	2219(2)	44(1)
C(11)	5059(2)	5396(3)	3040(2)	37(1)
C(12)	3961(2)	6054(2)	3309(2)	28(1)
C(13)	2993(2)	5874(2)	4078(2)	25(1)
C(14)	2159(2)	8017(2)	5553(2)	26(1)
C(15)	3642(2)	8341(2)	6551(2)	25(1)
C(16)	4917(2)	8823(2)	6316(2)	30(1)
C(17)	6292(2)	9148(2)	7192(2)	31(1)
C(18)	6395(2)	9022(2)	8340(2)	30(1)
C(19)	5163(2)	8565(2)	8615(2)	33(1)
C(20)	3790(2)	8218(2)	7714(2)	30(1)
C(21)	1911(3)	9445(2)	5612(2)	37(1)
C(22)	1360(3)	10029(3)	6795(2)	47(1)
C(23)	257(3)	8723(3)	6611(2)	53(1)
C(24)	814(2)	7423(2)	5890(2)	34(1)
C(25)	-210(2)	6318(2)	4579(2)	30(1)

Bond lengths [Å] and angles [°] for **15c**.

Cl(1)-C(18)	1.7408(19)
O(1)-C(3)	1.208(2)
O(2)-C(2)	1.452(2)
O(2)-C(3)	1.343(2)
O(3)-C(6)	1.216(2)
O(4)-C(13)	1.212(2)
C(1)-H(1A)	0.9800
C(1)-H(1B)	0.9800
C(1)-H(1C)	0.9800
C(1)-C(2)	1.506(3)
C(2)-H(2A)	0.9900
C(2)-H(2B)	0.9900
C(3)-C(4)	1.477(3)
C(4)-C(5)	1.515(2)
C(4)-C(25)	1.328(3)
C(5)-C(6)	1.541(3)
C(5)-C(13)	1.544(2)
C(5)-C(14)	1.586(2)
C(6)-C(7)	1.478(3)
C(7)-C(8)	1.392(3)
C(7)-C(12)	1.385(3)
C(8)-H(8)	0.9500
C(8)-C(9)	1.383(3)
C(9)-H(9)	0.9500
C(9)-C(10)	1.393(4)
C(10)-H(10)	0.9500
C(10)-C(11)	1.389(3)
C(11)-H(11)	0.9500
C(11)-C(12)	1.395(3)
C(12)-C(13)	1.480(3)
C(14)-C(15)	1.532(2)
C(14)-C(21)	1.549(3)
C(14)-C(24)	1.564(3)
C(15)-C(16)	1.398(3)
C(15)-C(20)	1.390(3)
C(16)-H(16)	0.9500

C(16)-C(17)	1.390(3)
C(17)-H(17)	0.9500
C(17)-C(18)	1.379(3)
C(18)-C(19)	1.378(3)
C(19)-H(19)	0.9500
C(19)-C(20)	1.396(3)
C(20)-H(20)	0.9500
C(21)-H(21B)	0.9900
C(21)-H(21A)	0.9900
C(21)-C(22)	1.522(3)
C(22)-H(22B)	0.9900
C(22)-H(22A)	0.9900
C(22)-C(23)	1.522(4)
C(23)-H(23A)	0.9900
C(23)-H(23B)	0.9900
C(23)-C(24)	1.552(3)
C(24)-H(24)	1.0000
C(24)-C(25)	1.490(3)
C(25)-H(25)	0.9500

C(3)-O(2)-C(2)	117.16(14)
H(1A)-C(1)-H(1B)	109.5
H(1A)-C(1)-H(1C)	109.5
H(1B)-C(1)-H(1C)	109.5
C(2)-C(1)-H(1A)	109.5
C(2)-C(1)-H(1B)	109.5
C(2)-C(1)-H(1C)	109.5
O(2)-C(2)-C(1)	106.30(16)
O(2)-C(2)-H(2A)	110.5
O(2)-C(2)-H(2B)	110.5
C(1)-C(2)-H(2A)	110.5
C(1)-C(2)-H(2B)	110.5
H(2A)-C(2)-H(2B)	108.7
O(1)-C(3)-O(2)	123.78(17)
O(1)-C(3)-C(4)	126.04(17)
O(2)-C(3)-C(4)	110.14(15)
C(3)-C(4)-C(5)	122.43(15)
C(25)-C(4)-C(3)	124.50(17)

C(25)-C(4)-C(5)	112.59(16)
C(4)-C(5)-C(6)	112.45(14)
C(4)-C(5)-C(13)	110.65(15)
C(4)-C(5)-C(14)	102.49(14)
C(6)-C(5)-C(13)	102.17(14)
C(6)-C(5)-C(14)	115.94(15)
C(13)-C(5)-C(14)	113.44(14)
O(3)-C(6)-C(5)	125.05(17)
O(3)-C(6)-C(7)	126.08(18)
C(7)-C(6)-C(5)	108.78(16)
C(8)-C(7)-C(6)	128.44(19)
C(12)-C(7)-C(6)	109.97(17)
C(12)-C(7)-C(8)	121.45(19)
C(7)-C(8)-H(8)	121.2
C(9)-C(8)-C(7)	117.5(2)
C(9)-C(8)-H(8)	121.2
C(8)-C(9)-H(9)	119.4
C(8)-C(9)-C(10)	121.2(2)
C(10)-C(9)-H(9)	119.4
C(9)-C(10)-H(10)	119.3
C(11)-C(10)-C(9)	121.4(2)
C(11)-C(10)-H(10)	119.3
C(10)-C(11)-H(11)	121.4
C(10)-C(11)-C(12)	117.2(2)
C(12)-C(11)-H(11)	121.4
C(7)-C(12)-C(11)	121.18(19)
C(7)-C(12)-C(13)	110.19(16)
C(11)-C(12)-C(13)	128.56(19)
O(4)-C(13)-C(5)	124.53(17)
O(4)-C(13)-C(12)	126.96(18)
C(12)-C(13)-C(5)	108.47(16)
C(15)-C(14)-C(5)	112.99(14)
C(15)-C(14)-C(21)	108.71(16)
C(15)-C(14)-C(24)	114.74(16)
C(21)-C(14)-C(5)	113.26(15)
C(21)-C(14)-C(24)	101.85(16)
C(24)-C(14)-C(5)	104.83(15)
C(16)-C(15)-C(14)	119.34(17)

C(20)-C(15)-C(14)	123.05(17)
C(20)-C(15)-C(16)	117.58(17)
C(15)-C(16)-H(16)	119.1
C(17)-C(16)-C(15)	121.88(18)
C(17)-C(16)-H(16)	119.1
C(16)-C(17)-H(17)	120.6
C(18)-C(17)-C(16)	118.85(19)
C(18)-C(17)-H(17)	120.6
C(17)-C(18)-Cl(1)	119.34(16)
C(19)-C(18)-Cl(1)	119.60(16)
C(19)-C(18)-C(17)	121.04(18)
C(18)-C(19)-H(19)	120.3
C(18)-C(19)-C(20)	119.43(19)
C(20)-C(19)-H(19)	120.3
C(15)-C(20)-C(19)	121.20(19)
C(15)-C(20)-H(20)	119.4
C(19)-C(20)-H(20)	119.4
C(14)-C(21)-H(21B)	111.1
C(14)-C(21)-H(21A)	111.1
H(21B)-C(21)-H(21A)	109.1
C(22)-C(21)-C(14)	103.31(18)
C(22)-C(21)-H(21B)	111.1
C(22)-C(21)-H(21A)	111.1
C(21)-C(22)-H(22B)	111.1
C(21)-C(22)-H(22A)	111.1
C(21)-C(22)-C(23)	103.42(18)
H(22B)-C(22)-H(22A)	109.0
C(23)-C(22)-H(22B)	111.1
C(23)-C(22)-H(22A)	111.1
C(22)-C(23)-H(23A)	110.7
C(22)-C(23)-H(23B)	110.7
C(22)-C(23)-C(24)	105.39(18)
H(23A)-C(23)-H(23B)	108.8
C(24)-C(23)-H(23A)	110.7
C(24)-C(23)-H(23B)	110.7
C(14)-C(24)-H(24)	110.2
C(23)-C(24)-C(14)	106.66(19)
C(23)-C(24)-H(24)	110.2

C(25)-C(24)-C(14)	103.80(15)
C(25)-C(24)-C(23)	115.38(17)
C(25)-C(24)-H(24)	110.2
C(4)-C(25)-C(24)	113.16(17)
C(4)-C(25)-H(25)	123.4
C(24)-C(25)-H(25)	123.4

Symmetry transformations used to generate equivalent atoms:

Anisotropic displacement parameters ($Å^2$x 10^3) for **15c**. The anisotropic displacement factor exponent takes the form: $-2\pi^2[h^2 a^{*2}U^{11} + ... + 2 h k a^* b^* U^{12}]$

	U^{11}	U^{22}	U^{33}	U^{23}	U^{13}	U^{12}
Cl(1)	28(1)	49(1)	48(1)	24(1)	-4(1)	4(1)
O(1)	22(1)	37(1)	29(1)	11(1)	7(1)	3(1)
O(2)	25(1)	28(1)	20(1)	5(1)	10(1)	6(1)
O(3)	30(1)	31(1)	30(1)	14(1)	10(1)	10(1)
O(4)	33(1)	38(1)	34(1)	19(1)	11(1)	14(1)
C(1)	40(1)	36(1)	29(1)	7(1)	17(1)	15(1)
C(2)	33(1)	27(1)	23(1)	3(1)	9(1)	7(1)
C(3)	24(1)	29(1)	23(1)	13(1)	9(1)	9(1)
C(4)	20(1)	29(1)	21(1)	10(1)	6(1)	9(1)
C(5)	21(1)	27(1)	21(1)	9(1)	7(1)	9(1)
C(6)	22(1)	26(1)	20(1)	6(1)	6(1)	5(1)
C(7)	25(1)	29(1)	24(1)	7(1)	10(1)	6(1)
C(8)	33(1)	39(1)	33(1)	13(1)	17(1)	8(1)
C(9)	36(1)	53(1)	40(1)	15(1)	24(1)	10(1)
C(10)	28(1)	58(2)	41(1)	13(1)	18(1)	18(1)
C(11)	29(1)	48(1)	32(1)	12(1)	11(1)	18(1)
C(12)	23(1)	34(1)	23(1)	7(1)	7(1)	10(1)
C(13)	21(1)	29(1)	21(1)	8(1)	4(1)	8(1)
C(14)	24(1)	31(1)	20(1)	8(1)	7(1)	10(1)
C(15)	26(1)	23(1)	22(1)	7(1)	6(1)	9(1)
C(16)	29(1)	31(1)	28(1)	13(1)	8(1)	6(1)
C(17)	26(1)	31(1)	35(1)	15(1)	8(1)	4(1)
C(18)	26(1)	27(1)	31(1)	11(1)	2(1)	6(1)
C(19)	32(1)	37(1)	26(1)	15(1)	3(1)	8(1)
C(20)	27(1)	36(1)	26(1)	12(1)	8(1)	9(1)
C(21)	43(1)	39(1)	24(1)	7(1)	6(1)	24(1)
C(22)	47(1)	53(1)	25(1)	0(1)	4(1)	29(1)
C(23)	33(1)	76(2)	28(1)	0(1)	12(1)	21(1)
C(24)	23(1)	51(1)	22(1)	10(1)	10(1)	11(1)
C(25)	22(1)	42(1)	25(1)	11(1)	9(1)	10(1)

Hydrogen coordinates (x 10^4) and isotropic displacement parameters (Å^{2}x 10^3) for **15c**.

	x	y	z	U(eq)
H(1A)	1176	2690	-1133	53
H(1B)	2263	3341	341	53
H(1C)	1768	4389	-241	53
H(2A)	-654	3571	-294	37
H(2B)	-148	2529	303	37
H(8)	4026	7861	1592	42
H(9)	5875	6771	1129	52
H(10)	6508	5253	2015	53
H(11)	5318	4776	3402	44
H(16)	4842	8932	5535	36
H(17)	7146	9453	7004	37
H(19)	5248	8488	9412	39
H(20)	2942	7892	7898	36
H(21B)	2832	10111	5749	44
H(21A)	1177	9279	4792	44
H(22B)	891	10789	6787	56
H(22A)	2166	10422	7636	56
H(23A)	-726	8627	6072	63
H(23B)	211	8798	7476	63
H(24)	1110	6971	6477	41
H(25)	-1196	5881	4443	36

Torsion angles [°] for **15c**

Cl(1)-C(18)-C(19)-C(20)	178.99(16)
O(1)-C(3)-C(4)-C(5)	-179.87(18)
O(1)-C(3)-C(4)-C(25)	8.8(3)
O(2)-C(3)-C(4)-C(5)	2.1(2)
O(2)-C(3)-C(4)-C(25)	-169.28(18)
O(3)-C(6)-C(7)-C(8)	3.1(3)
O(3)-C(6)-C(7)-C(12)	-172.47(18)
C(2)-O(2)-C(3)-O(1)	-2.0(3)
C(2)-O(2)-C(3)-C(4)	176.03(15)
C(3)-O(2)-C(2)-C(1)	179.91(16)
C(3)-C(4)-C(5)-C(6)	51.6(2)
C(3)-C(4)-C(5)-C(13)	-61.9(2)
C(3)-C(4)-C(5)-C(14)	176.79(16)
C(3)-C(4)-C(25)-C(24)	172.17(18)
C(4)-C(5)-C(6)-O(3)	52.0(2)
C(4)-C(5)-C(6)-C(7)	-124.89(16)
C(4)-C(5)-C(13)-O(4)	-51.7(2)
C(4)-C(5)-C(13)-C(12)	125.99(16)
C(4)-C(5)-C(14)-C(15)	142.26(16)
C(4)-C(5)-C(14)-C(21)	-93.57(18)
C(4)-C(5)-C(14)-C(24)	16.62(18)
C(5)-C(4)-C(25)-C(24)	0.1(3)
C(5)-C(6)-C(7)-C(8)	179.94(19)
C(5)-C(6)-C(7)-C(12)	4.3(2)
C(5)-C(14)-C(15)-C(16)	57.4(2)
C(5)-C(14)-C(15)-C(20)	-124.5(2)
C(5)-C(14)-C(21)-C(22)	151.74(17)
C(5)-C(14)-C(24)-C(23)	-139.07(16)
C(5)-C(14)-C(24)-C(25)	-16.8(2)
C(6)-C(5)-C(13)-O(4)	-171.58(18)
C(6)-C(5)-C(13)-C(12)	6.07(18)
C(6)-C(5)-C(14)-C(15)	-94.87(19)
C(6)-C(5)-C(14)-C(21)	29.3(2)
C(6)-C(5)-C(14)-C(24)	139.49(16)
C(6)-C(7)-C(8)-C(9)	-176.1(2)
C(6)-C(7)-C(12)-C(11)	177.01(18)

C(6)-C(7)-C(12)-C(13)	-0.3(2)
C(7)-C(8)-C(9)-C(10)	0.4(3)
C(7)-C(12)-C(13)-O(4)	173.68(19)
C(7)-C(12)-C(13)-C(5)	-3.9(2)
C(8)-C(7)-C(12)-C(11)	1.0(3)
C(8)-C(7)-C(12)-C(13)	-176.23(18)
C(8)-C(9)-C(10)-C(11)	0.0(4)
C(9)-C(10)-C(11)-C(12)	0.1(3)
C(10)-C(11)-C(12)-C(7)	-0.6(3)
C(10)-C(11)-C(12)-C(13)	176.1(2)
C(11)-C(12)-C(13)-O(4)	-3.3(3)
C(11)-C(12)-C(13)-C(5)	179.09(19)
C(12)-C(7)-C(8)-C(9)	-0.9(3)
C(13)-C(5)-C(6)-O(3)	170.60(18)
C(13)-C(5)-C(6)-C(7)	-6.24(18)
C(13)-C(5)-C(14)-C(15)	22.9(2)
C(13)-C(5)-C(14)-C(21)	147.11(17)
C(13)-C(5)-C(14)-C(24)	-102.71(17)
C(14)-C(5)-C(6)-O(3)	-65.5(2)
C(14)-C(5)-C(6)-C(7)	117.64(17)
C(14)-C(5)-C(13)-O(4)	62.9(2)
C(14)-C(5)-C(13)-C(12)	-119.47(17)
C(14)-C(15)-C(16)-C(17)	179.30(18)
C(14)-C(15)-C(20)-C(19)	-178.06(18)
C(14)-C(21)-C(22)-C(23)	-44.2(2)
C(14)-C(24)-C(25)-C(4)	11.0(2)
C(15)-C(14)-C(21)-C(22)	-81.8(2)
C(15)-C(14)-C(24)-C(23)	96.4(2)
C(15)-C(14)-C(24)-C(25)	-141.32(17)
C(15)-C(16)-C(17)-C(18)	-1.5(3)
C(16)-C(15)-C(20)-C(19)	0.1(3)
C(16)-C(17)-C(18)-Cl(1)	-177.87(16)
C(16)-C(17)-C(18)-C(19)	0.8(3)
C(17)-C(18)-C(19)-C(20)	0.4(3)
C(18)-C(19)-C(20)-C(15)	-0.8(3)
C(20)-C(15)-C(16)-C(17)	1.1(3)
C(21)-C(14)-C(15)-C(16)	-69.2(2)
C(21)-C(14)-C(15)-C(20)	108.9(2)

C(21)-C(14)-C(24)-C(23)	-20.8(2)
C(21)-C(14)-C(24)-C(25)	101.45(18)
C(21)-C(22)-C(23)-C(24)	30.3(2)
C(22)-C(23)-C(24)-C(14)	-5.5(2)
C(22)-C(23)-C(24)-C(25)	-120.2(2)
C(23)-C(24)-C(25)-C(4)	127.4(2)
C(24)-C(14)-C(15)-C(16)	177.53(17)
C(24)-C(14)-C(15)-C(20)	-4.4(3)
C(24)-C(14)-C(21)-C(22)	39.72(19)
C(25)-C(4)-C(5)-C(6)	-136.09(18)
C(25)-C(4)-C(5)-C(13)	110.36(19)
C(25)-C(4)-C(5)-C(14)	-10.9(2)

Symmetry transformations used to generate equivalent atoms:

8. Copies of NMR Spectra of Products

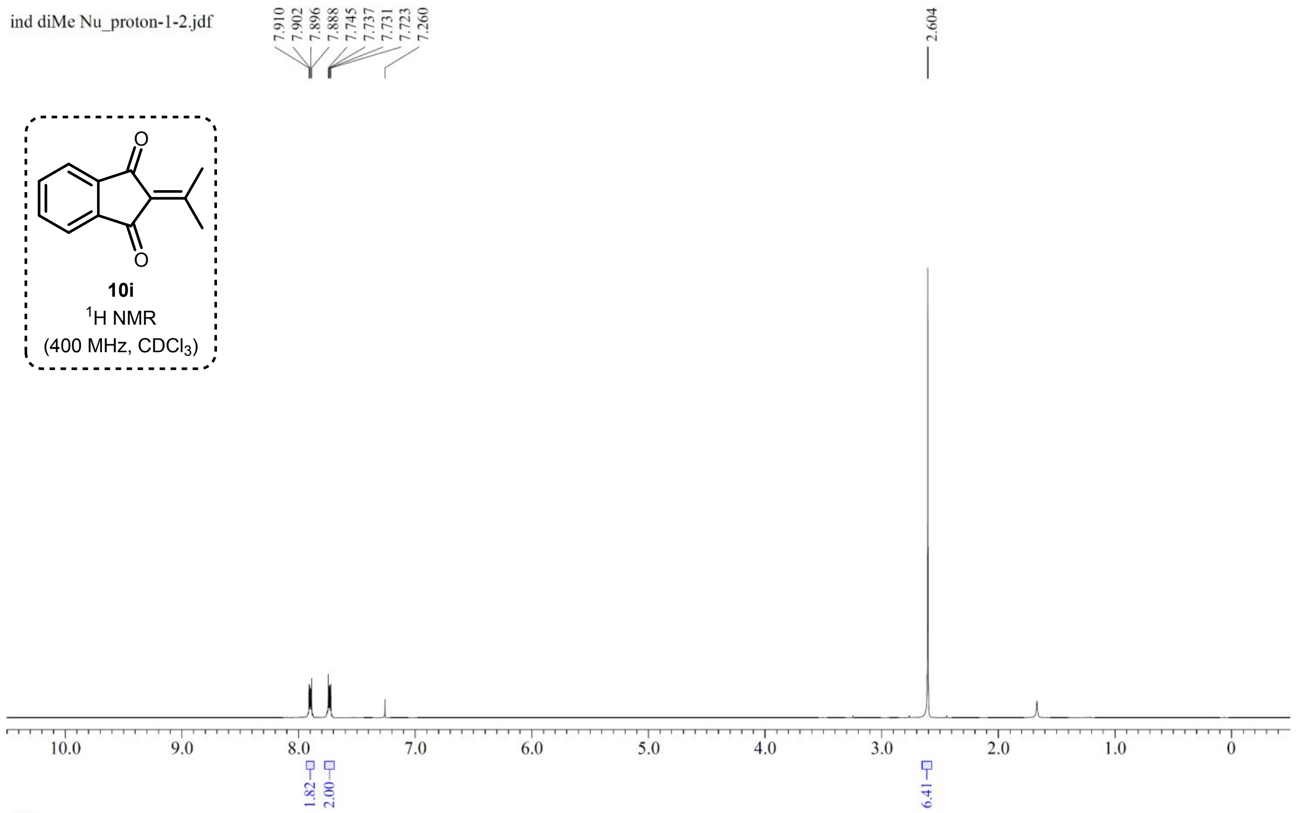

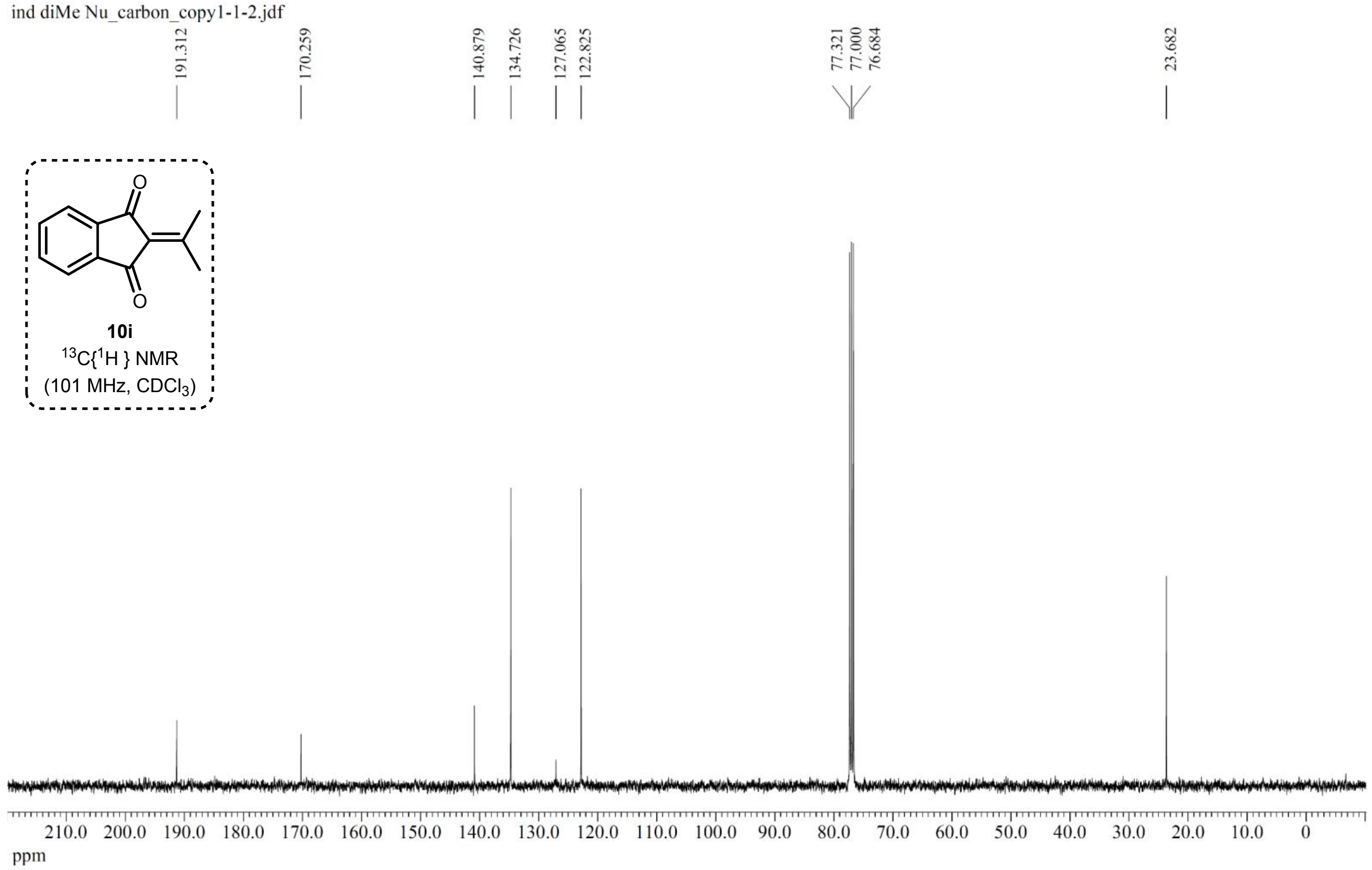

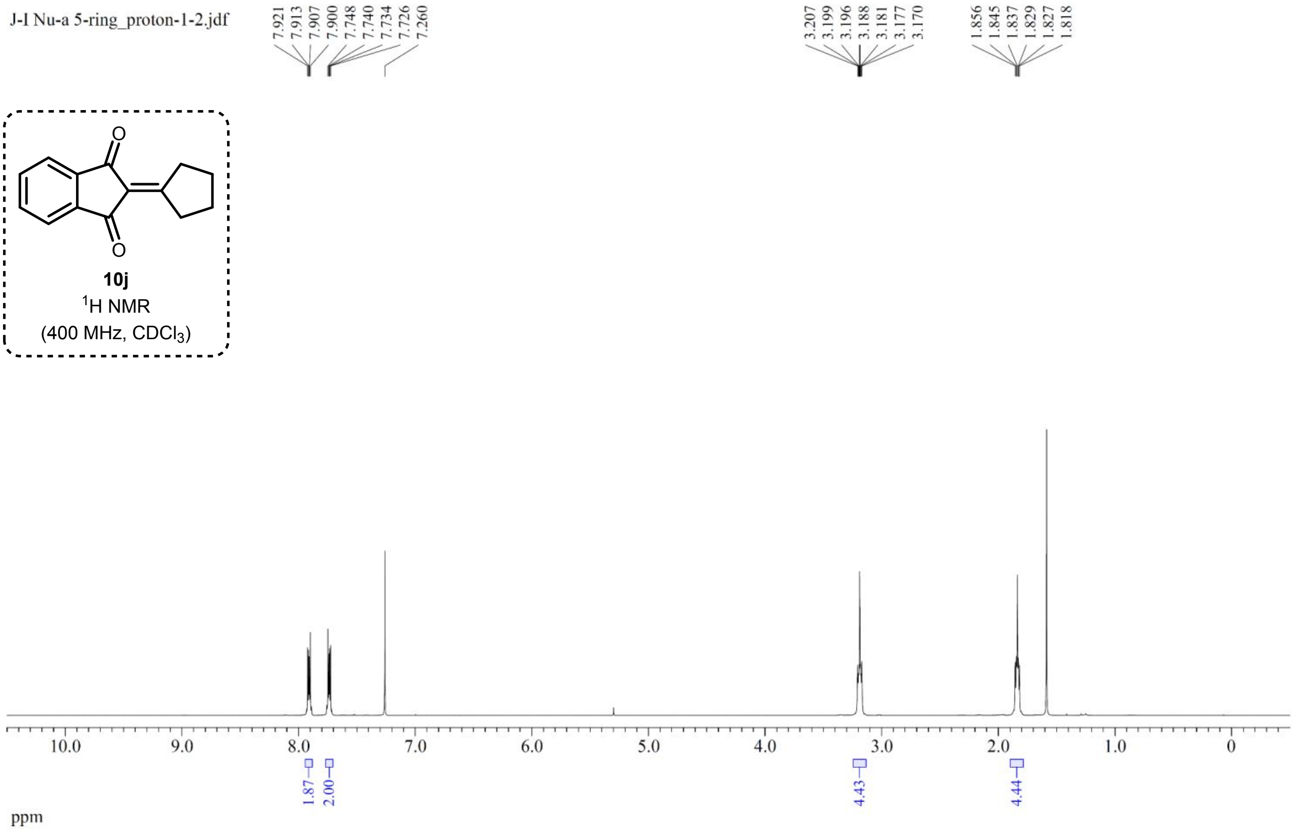
J-I Nu-a 5-ring_proton-1-2.jdf
7.921
7.913
7.907
7.900
7.748
7.740
7.734
7.726
7.260
3.207
3.199
3.196
3.188
3.181
3.177
3.170
1.856
1.845
1.837
1.829
1.827
1.818
10j
1H NMR
(400 MHz, CDCl3)
10.0
9.0
8.0
7.0
6.0
5.0
4.0
3.0
2.0
1.0
0
1.87
2.00
4.43
4.44
ppm

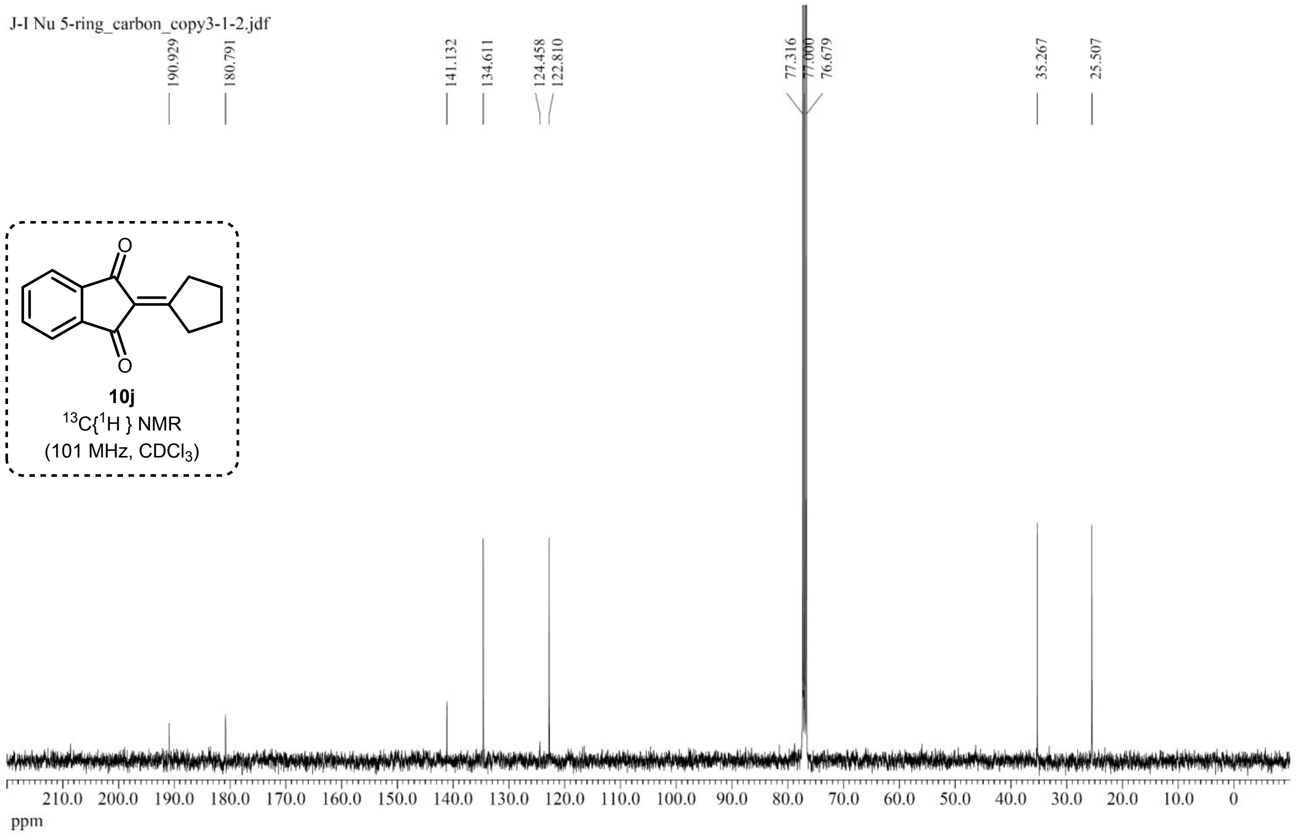
J-I Nu 5-ring_carbon_copy3-1-2.jdf
190.929
180.791
141.132
134.611
124.458
122.810
77.316
77.000
76.679
35.267
25.507
10j
13C{1H } NMR
(101 MHz, CDCl3)
210.0
200.0
190.0
180.0
170.0
160.0
150.0
140.0
130.0
120.0
110.0
100.0
90.0
80.0
70.0
60.0
50.0
40.0
30.0
20.0
10.0
0
ppm

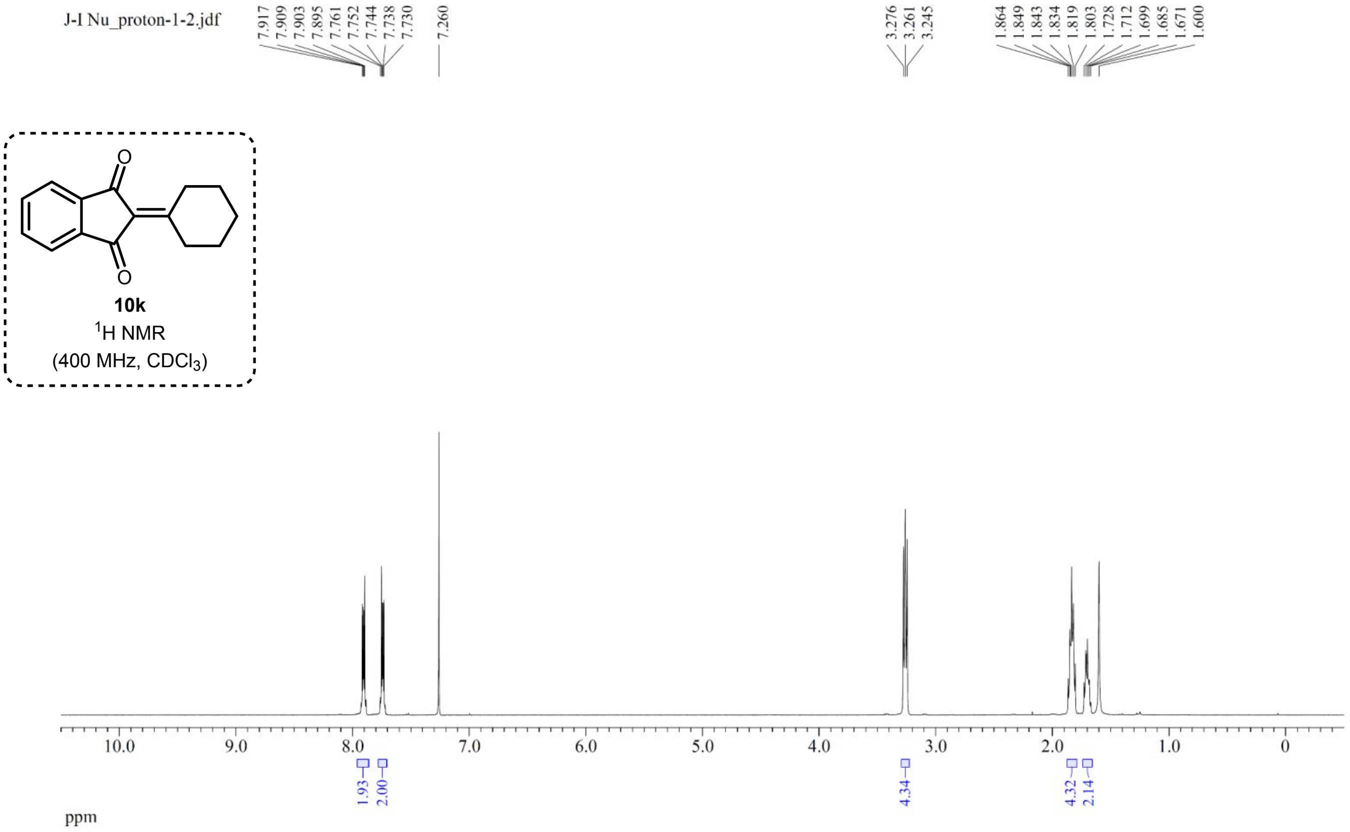
J-I Nu_proton-1-2.jdf
7.917
7.909
7.903
7.895
7.761
7.752
7.744
7.738
7.730
7.260
3.276
3.261
3.245
1.864
1.849
1.843
1.834
1.819
1.803
1.728
1.712
1.699
1.685
1.671
1.600
O
O
10k
1H NMR
(400 MHz, CDCl3)
10.0 9.0 8.0 7.0 6.0 5.0 4.0 3.0 2.0 1.0 0
1.93
2.00
4.34
4.32
2.14
ppm

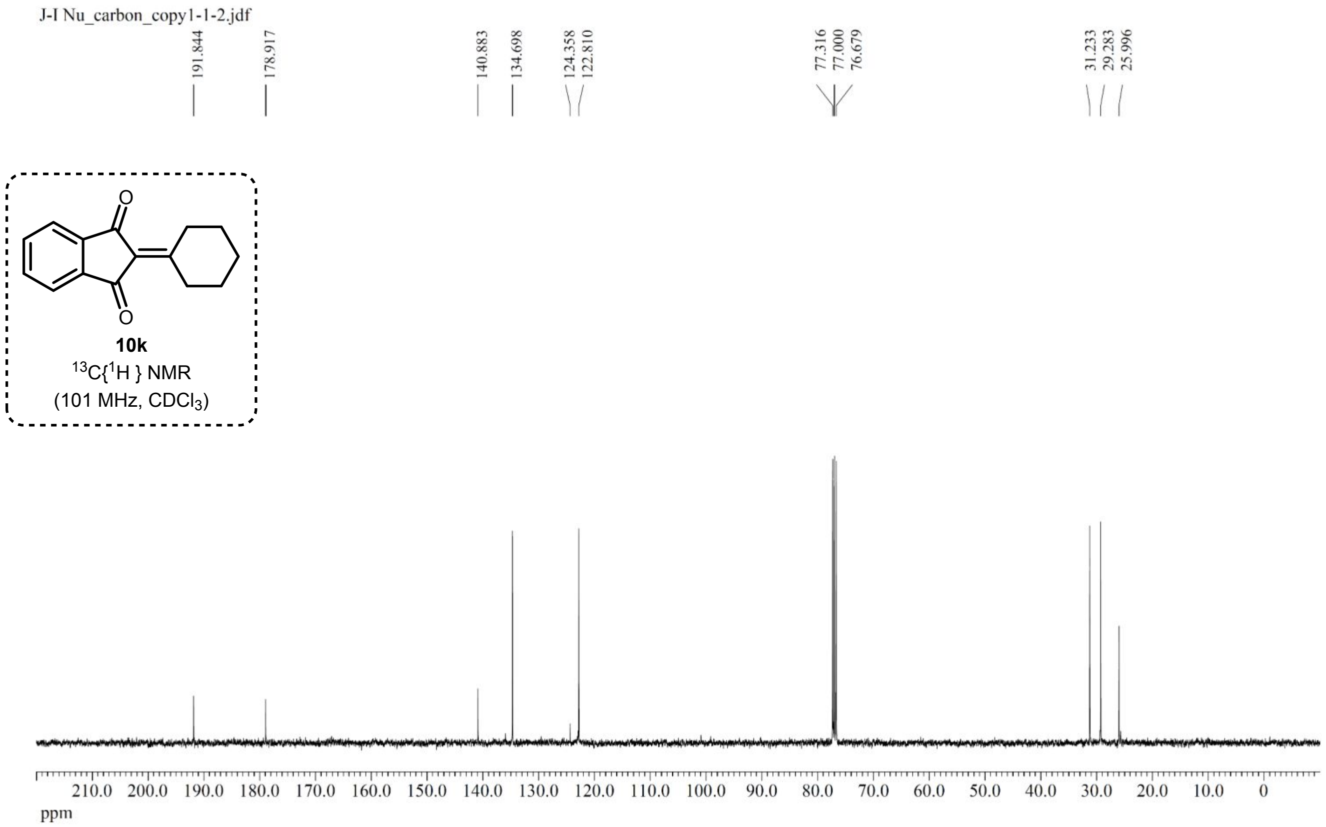
J-I Nu_carbon_copy1-1-2.jdf
191.844
178.917
140.883
134.698
124.358
122.810
77.316
77.000
76.679
31.233
29.283
25.996
O
O
10k
13C{1H } NMR
(101 MHz, CDCl3)
210.0 200.0 190.0 180.0 170.0 160.0 150.0 140.0 130.0 120.0 110.0 100.0 90.0 80.0 70.0 60.0 50.0 40.0 30.0 20.0 10.0 0
ppm

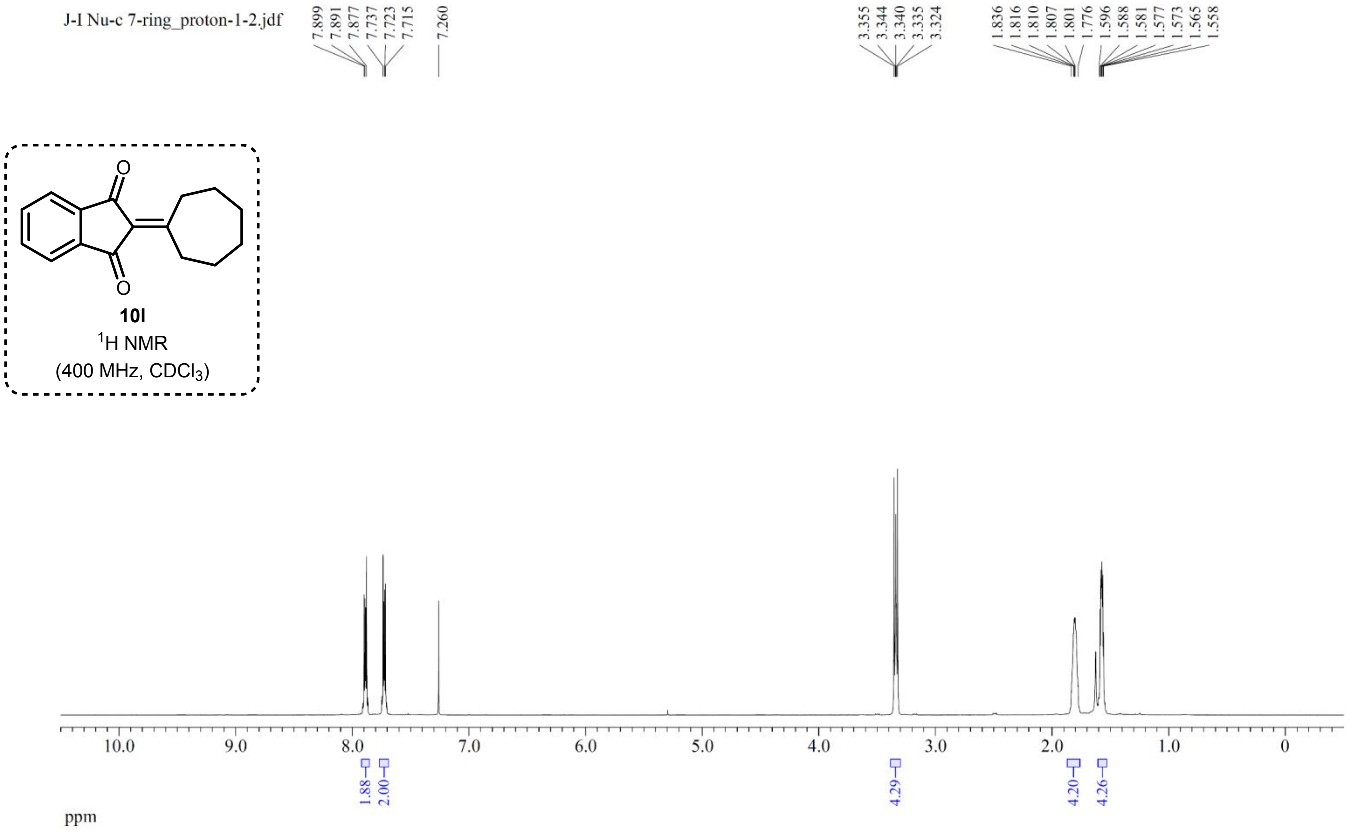
J-I Nu-c 7-ring_proton-1-2.jdf
7.899
7.891
7.877
7.737
7.723
7.715
7.260
3.355
3.344
3.340
3.335
3.324
1.836
1.816
1.810
1.807
1.801
1.776
1.596
1.588
1.581
1.577
1.573
1.565
1.558
10l
1H NMR
(400 MHz, CDCl3)
1.88
2.00
4.29
4.20
4.26
10.0
9.0
8.0
7.0
6.0
5.0
4.0
3.0
2.0
1.0
0
ppm

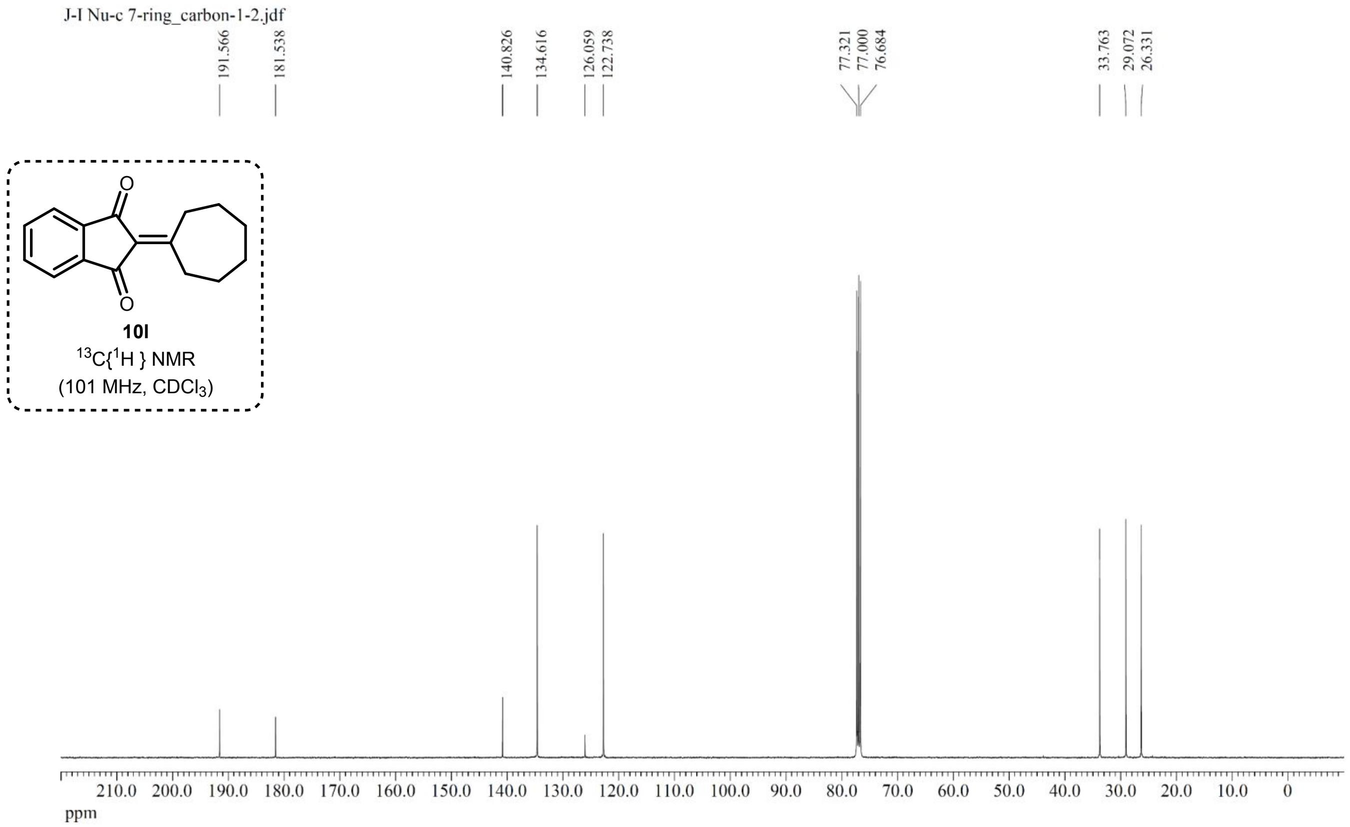
J-I Nu-c 7-ring_carbon-1-2.jdf
191.566
181.538
140.826
134.616
126.059
122.738
77.321
77.000
76.684
33.763
29.072
26.331
10l
13C{1H } NMR
(101 MHz, CDCl3)
210.0
200.0
190.0
180.0
170.0
160.0
150.0
140.0
130.0
120.0
110.0
100.0
90.0
80.0
70.0
60.0
50.0
40.0
30.0
20.0
10.0
0
ppm

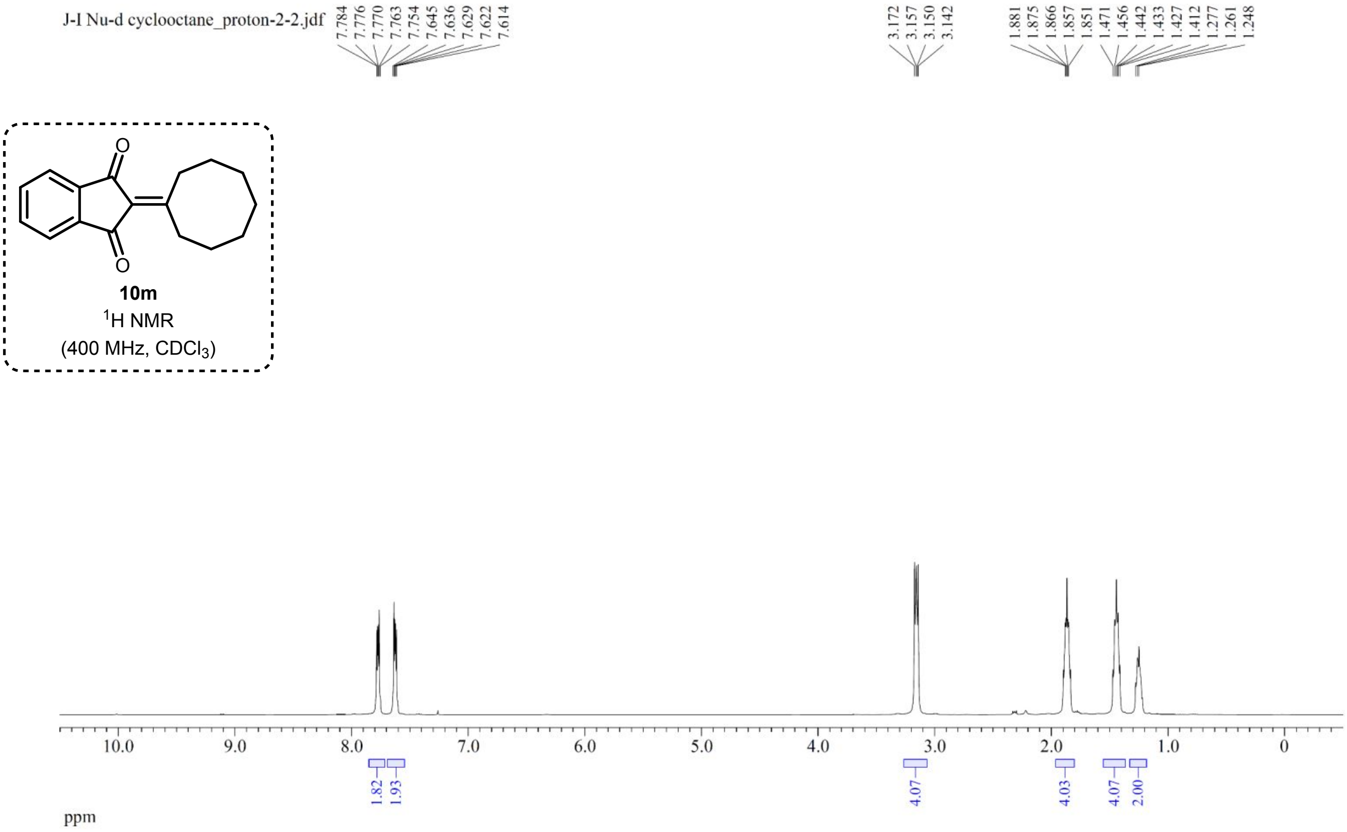

10m
^{1}H NMR
(400 MHz, $CDCl_3$)

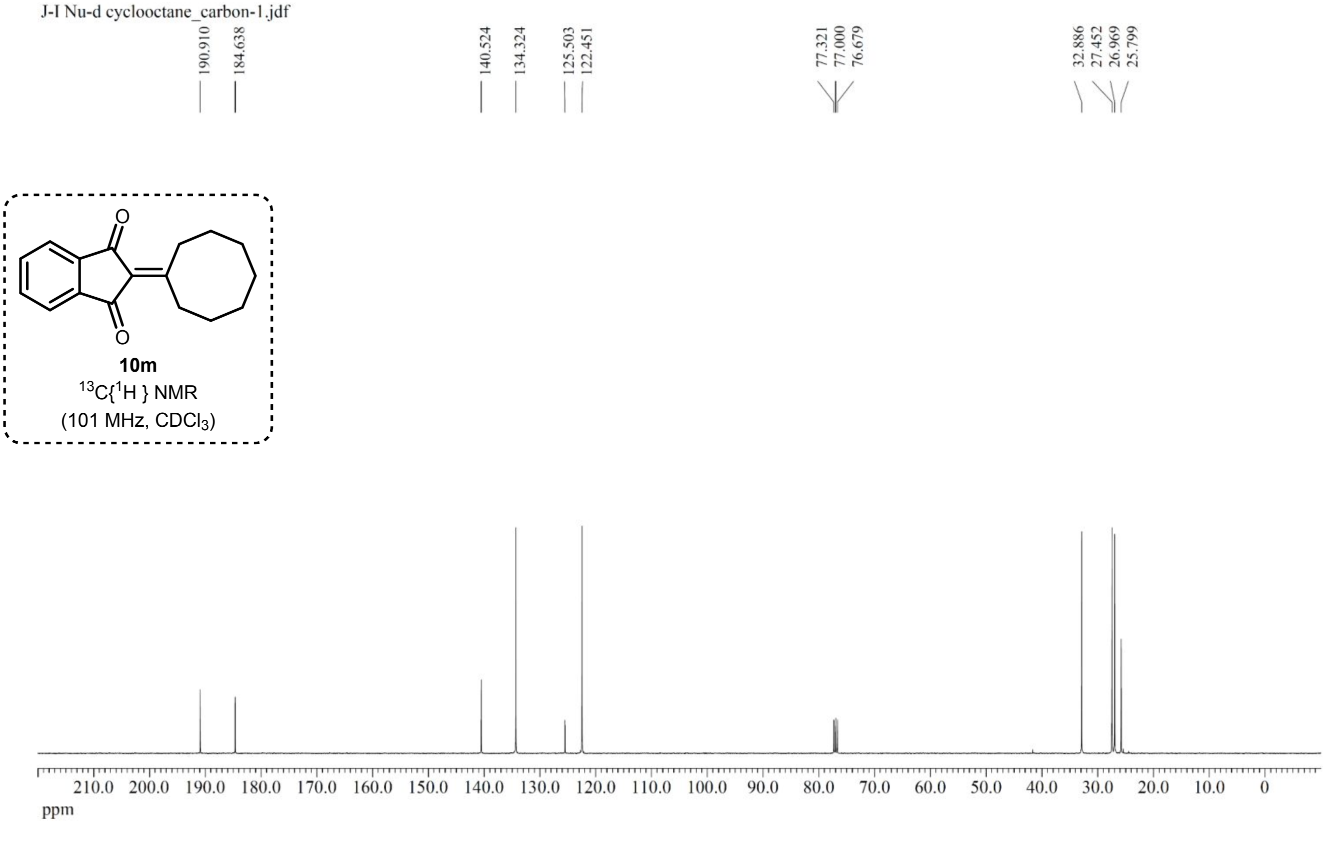

10m
$^{13}C\{^{1}H\}$ NMR
(101 MHz, $CDCl_3$)

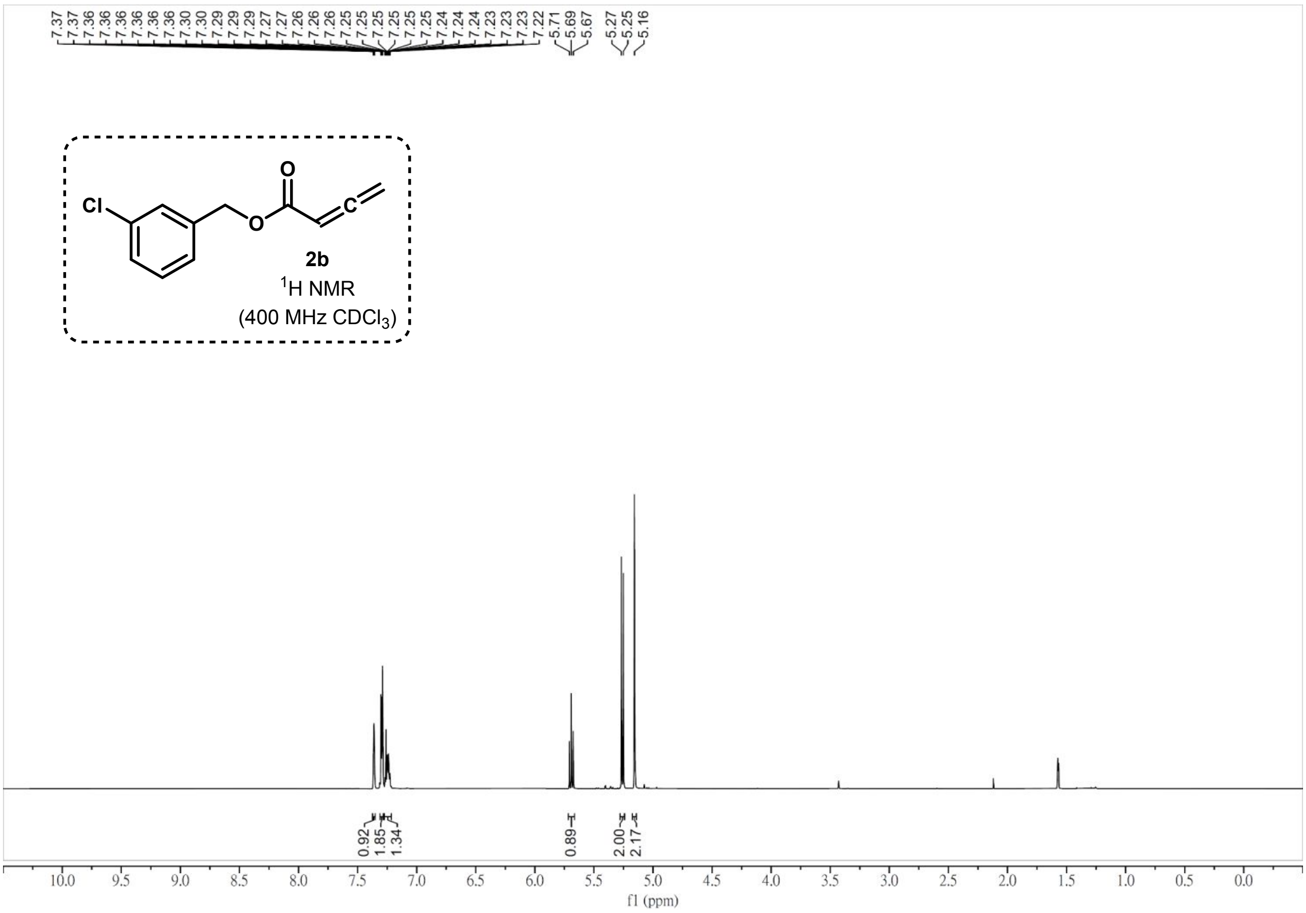
7.37
7.37
7.36
7.36
7.36
7.36
7.36
7.30
7.30
7.29
7.29
7.29
7.27
7.27
7.26
7.26
7.26
7.25
7.25
7.25
7.25
7.25
7.25
7.24
7.24
7.24
7.23
7.23
7.23
7.22
5.71
5.69
5.67
5.27
5.25
5.16
Cl
O
O
C
2b
1H NMR
(400 MHz CDCl3)
0.92
1.85
1.34
0.89
2.00
2.17
f1 (ppm)

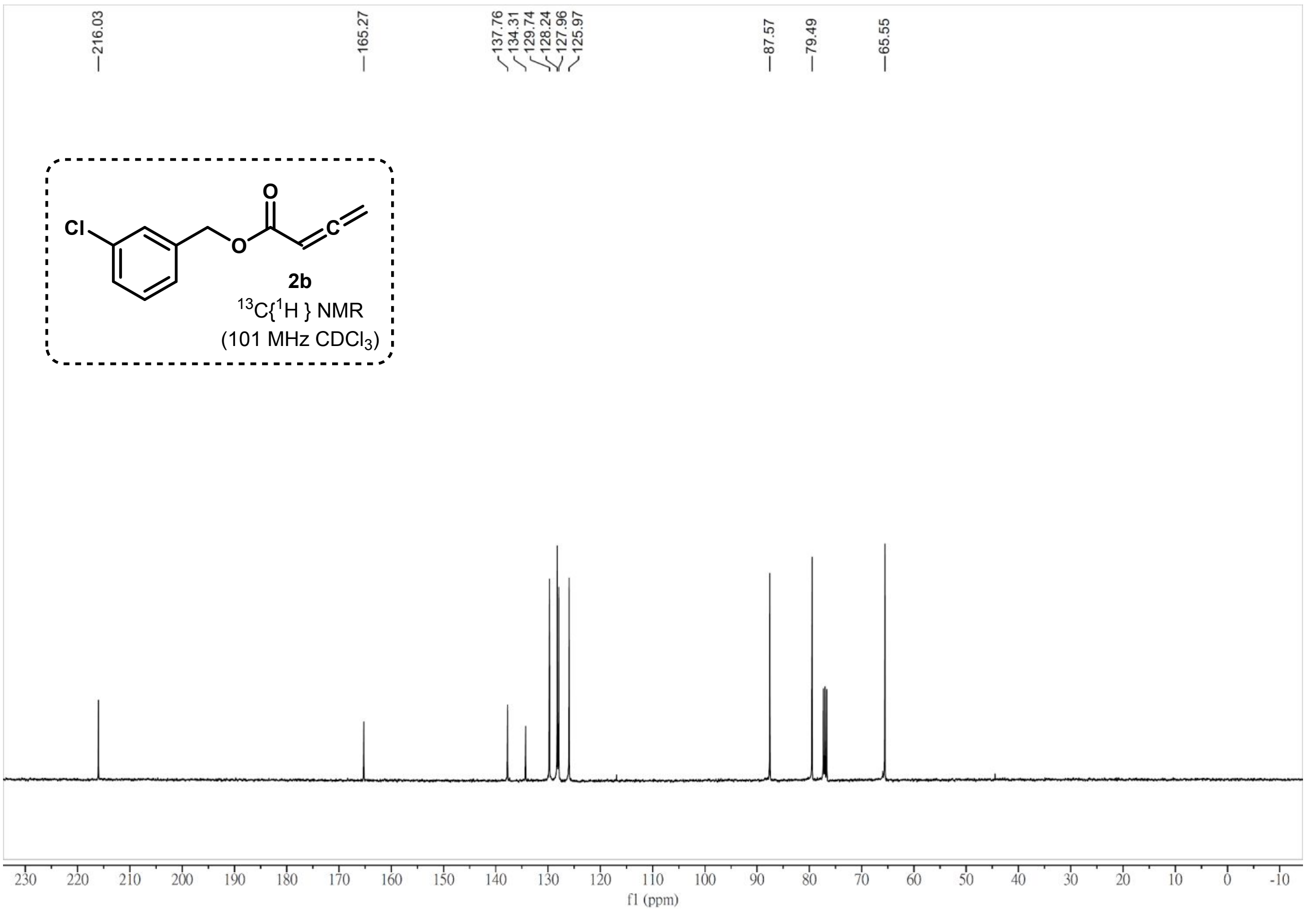
216.03
165.27
137.76
134.31
129.74
128.24
127.96
125.97
87.57
79.49
65.55
Cl
O
O
C
2b
13C{1H } NMR
(101 MHz CDCl3)
f1 (ppm)

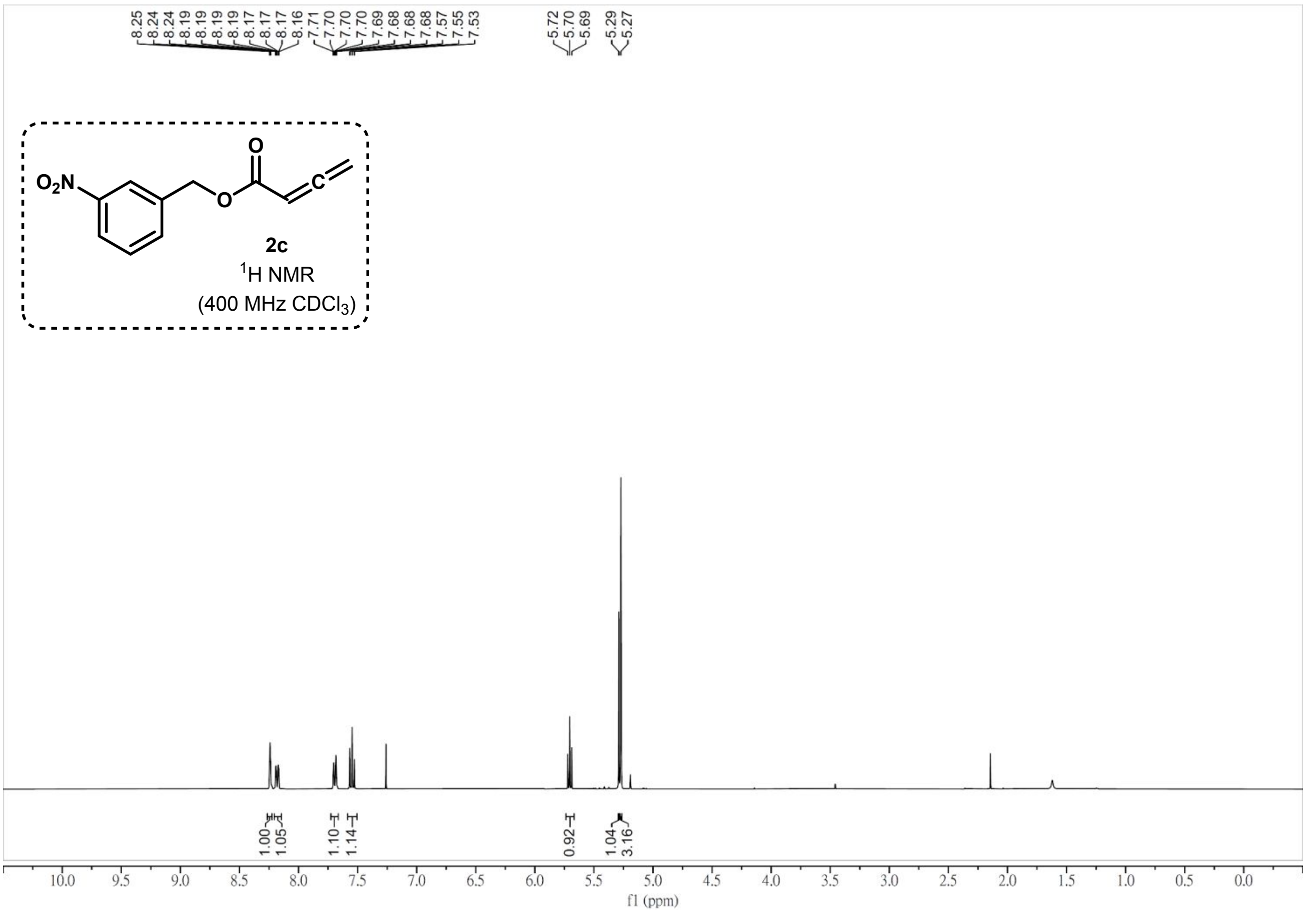

8.25
8.24
8.24
8.19
8.19
8.19
8.19
8.17
8.17
8.17
8.16
7.71
7.70
7.70
7.70
7.69
7.68
7.68
7.68
7.57
7.55
7.53
5.72
5.70
5.69
5.29
5.27
O2N
O
O
C
2c
1H NMR
(400 MHz CDCl3)
1.00
1.05
1.10
1.14
0.92
1.04
3.16
f1 (ppm)

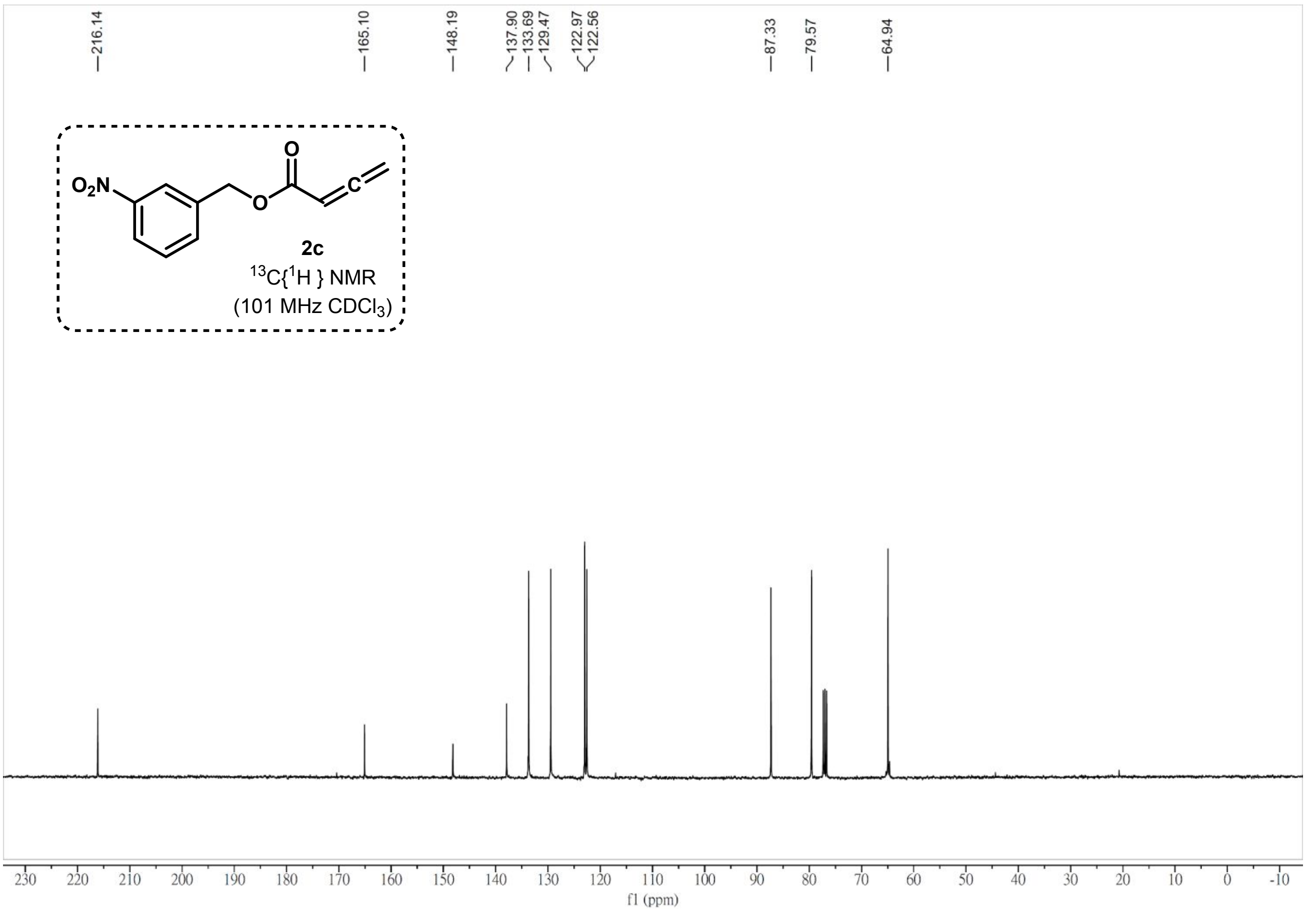

216.14
165.10
148.19
137.90
133.69
129.47
122.97
122.56
87.33
79.57
64.94
O2N
O
O
C
2c
13C{1H } NMR
(101 MHz CDCl3)
f1 (ppm)

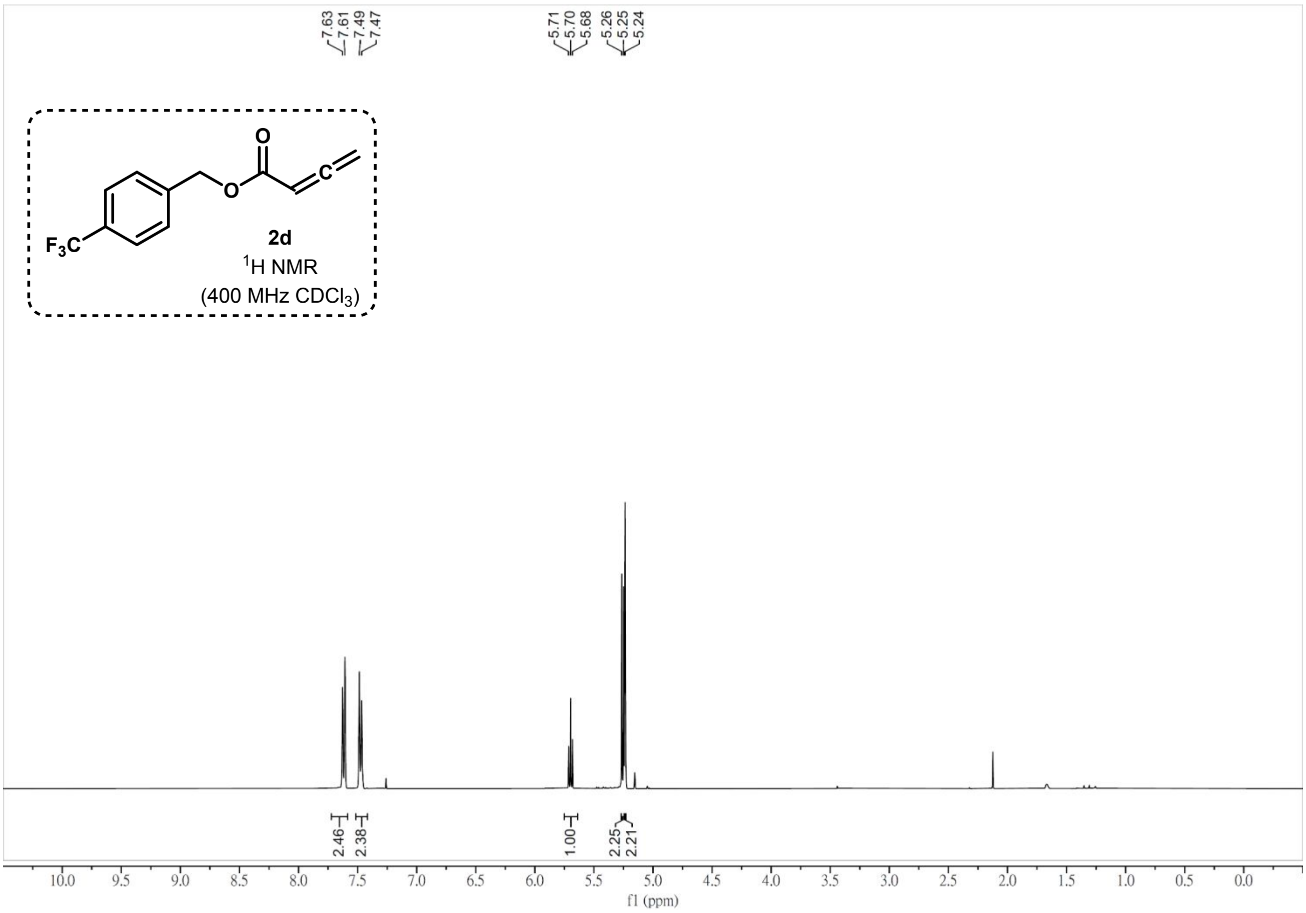
7.63
7.61
7.49
7.47
5.71
5.70
5.68
5.26
5.25
5.24
2d
1H NMR
(400 MHz CDCl3)
2.46
2.38
1.00
2.25
2.21
f1 (ppm)

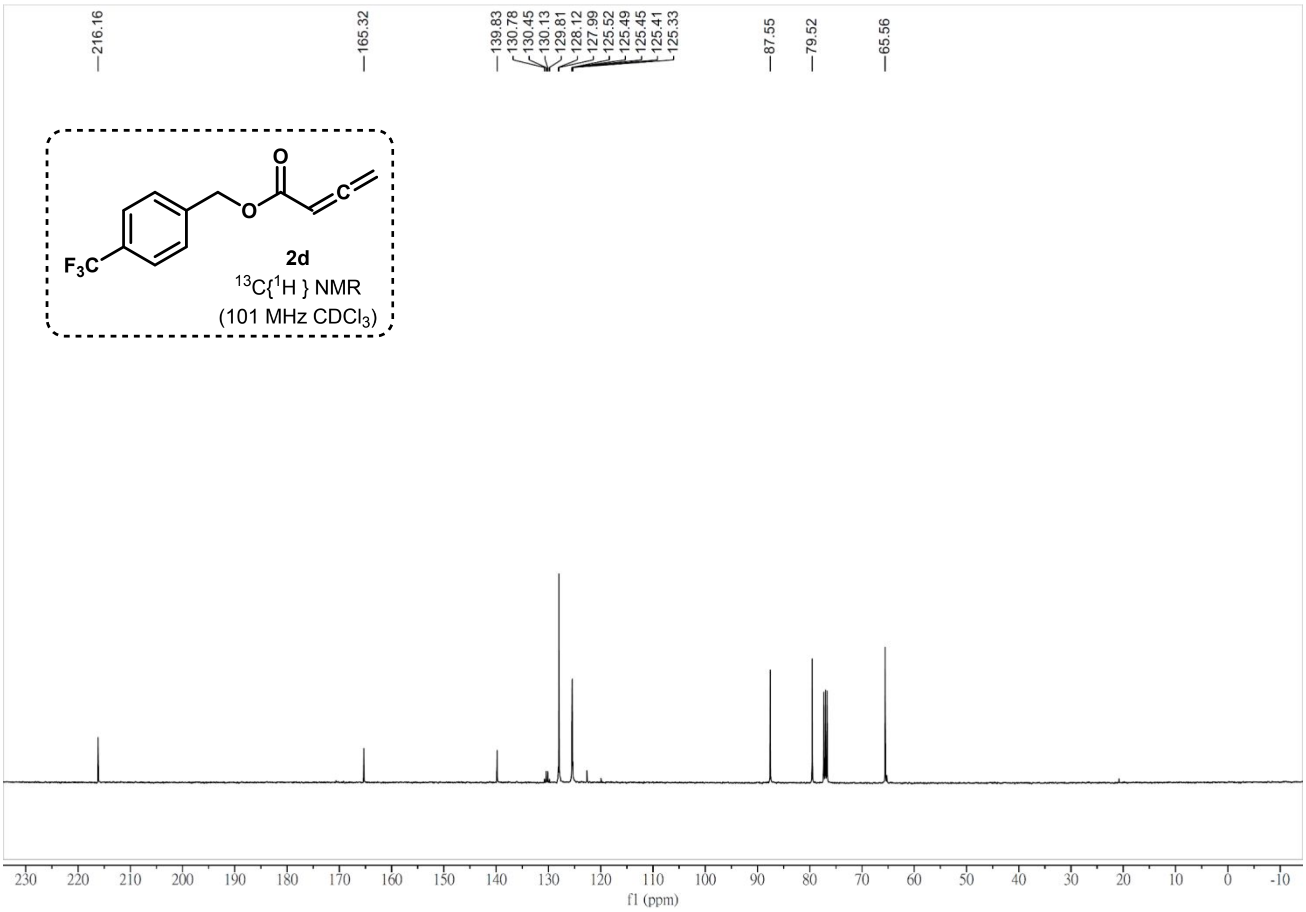
216.16
165.32
139.83
130.78
130.45
130.13
129.81
128.12
127.99
125.52
125.49
125.45
125.41
125.33
87.55
79.52
65.56
2d
13C{1H } NMR
(101 MHz CDCl3)
f1 (ppm)

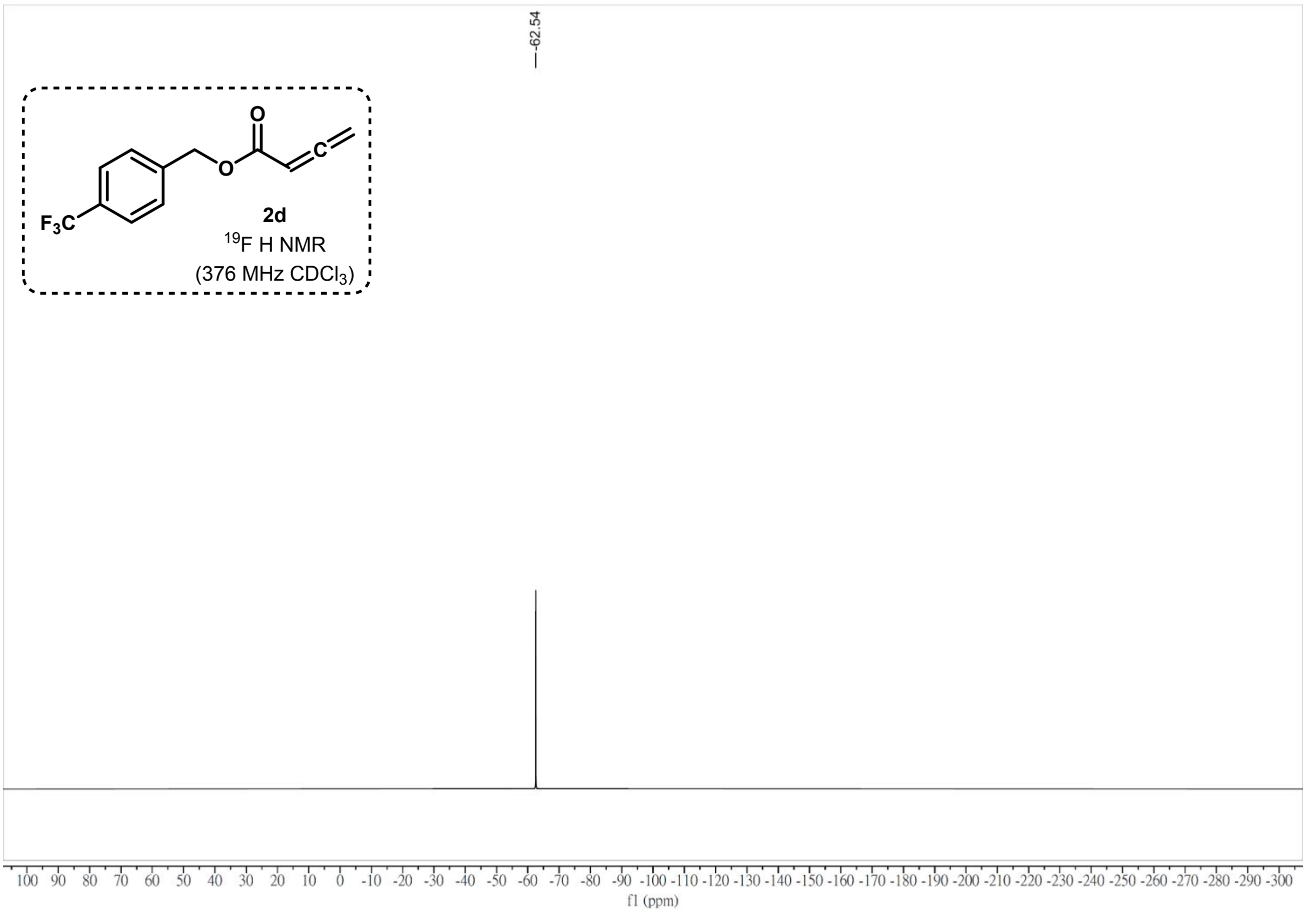

-62.54
F_3C
O
O
C
2d
^{19}F H NMR
(376 MHz $CDCl_3$)
100 90 80 70 60 50 40 30 20 10 0 -10 -20 -30 -40 -50 -60 -70 -80 -90 -100 -110 -120 -130 -140 -150 -160 -170 -180 -190 -200 -210 -220 -230 -240 -250 -260 -270 -280 -290 -300
f1 (ppm)

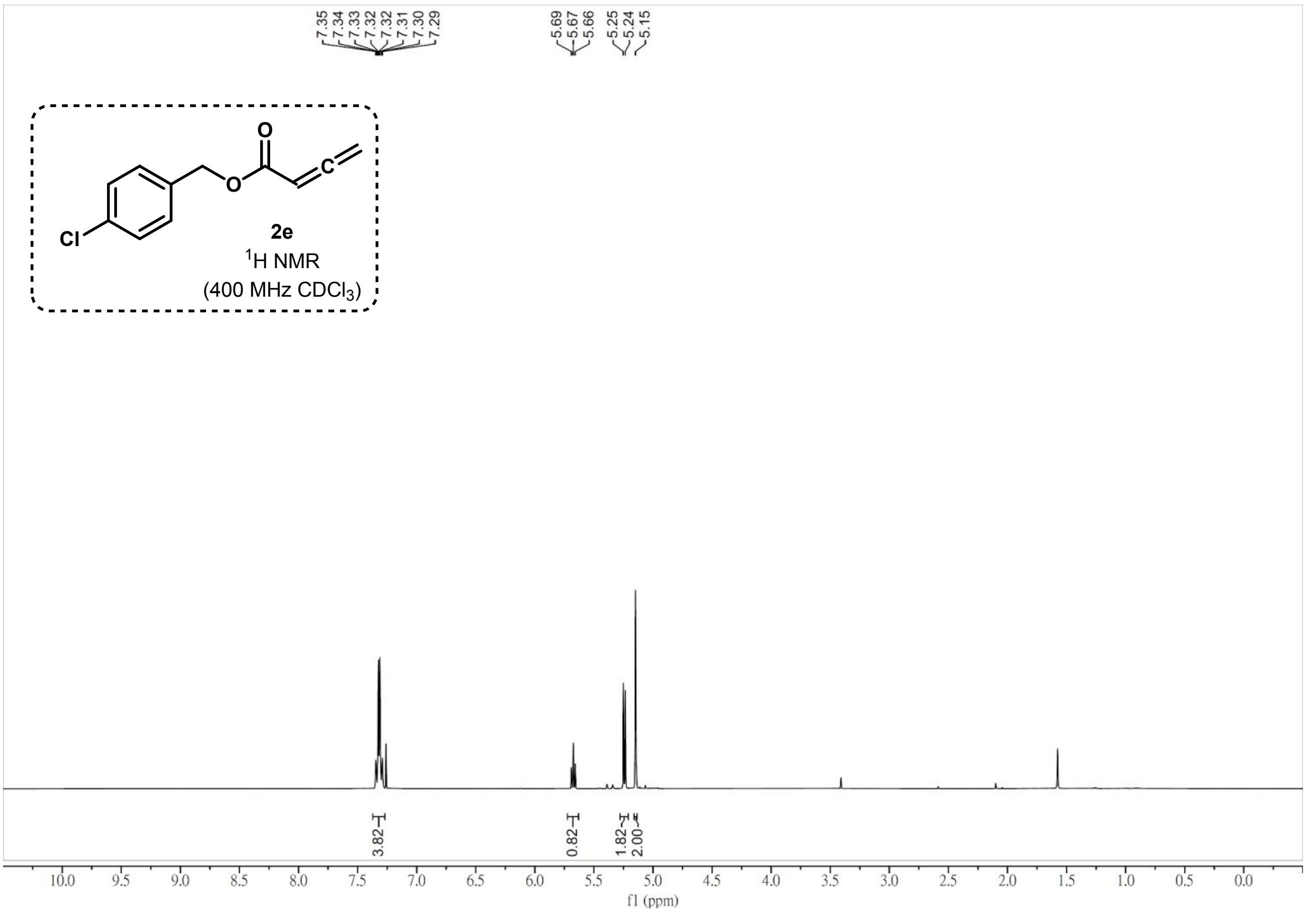
7.35
7.34
7.33
7.32
7.32
7.31
7.30
7.29
5.69
5.67
5.66
5.25
5.24
5.15
O
O
Cl
2e
^{1}H NMR
(400 MHz $CDCl_3$)
3.82
0.82
1.82
2.00
10.0 9.5 9.0 8.5 8.0 7.5 7.0 6.5 6.0 5.5 5.0 4.5 4.0 3.5 3.0 2.5 2.0 1.5 1.0 0.5 0.0
f1 (ppm)

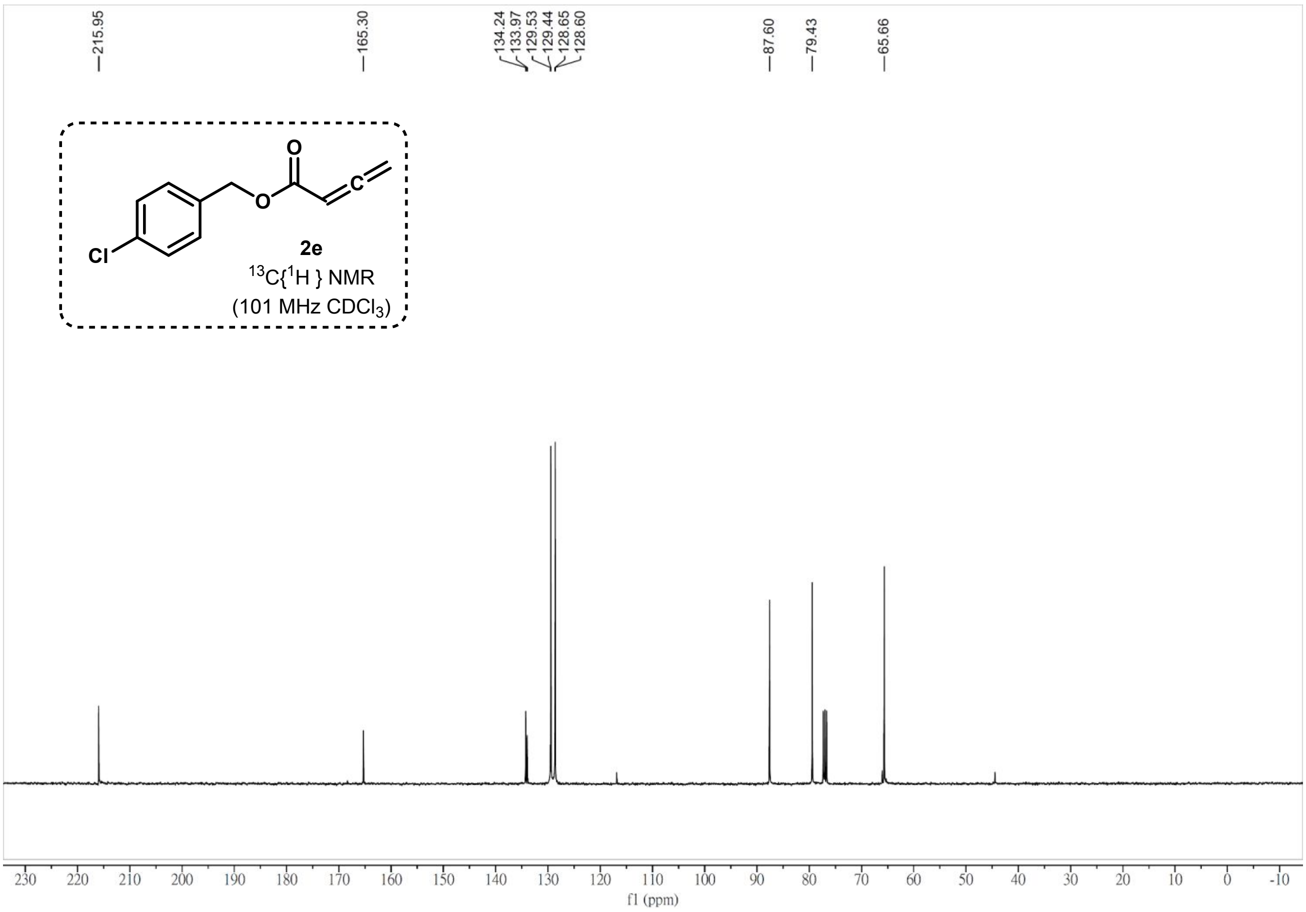
215.95
165.30
134.24
133.97
129.53
129.44
128.65
128.60
87.60
79.43
65.66
O
O
Cl
2e
^{13}C{^{1}H } NMR
(101 MHz $CDCl_3$)
230 220 210 200 190 180 170 160 150 140 130 120 110 100 90 80 70 60 50 40 30 20 10 0 -10
f1 (ppm)

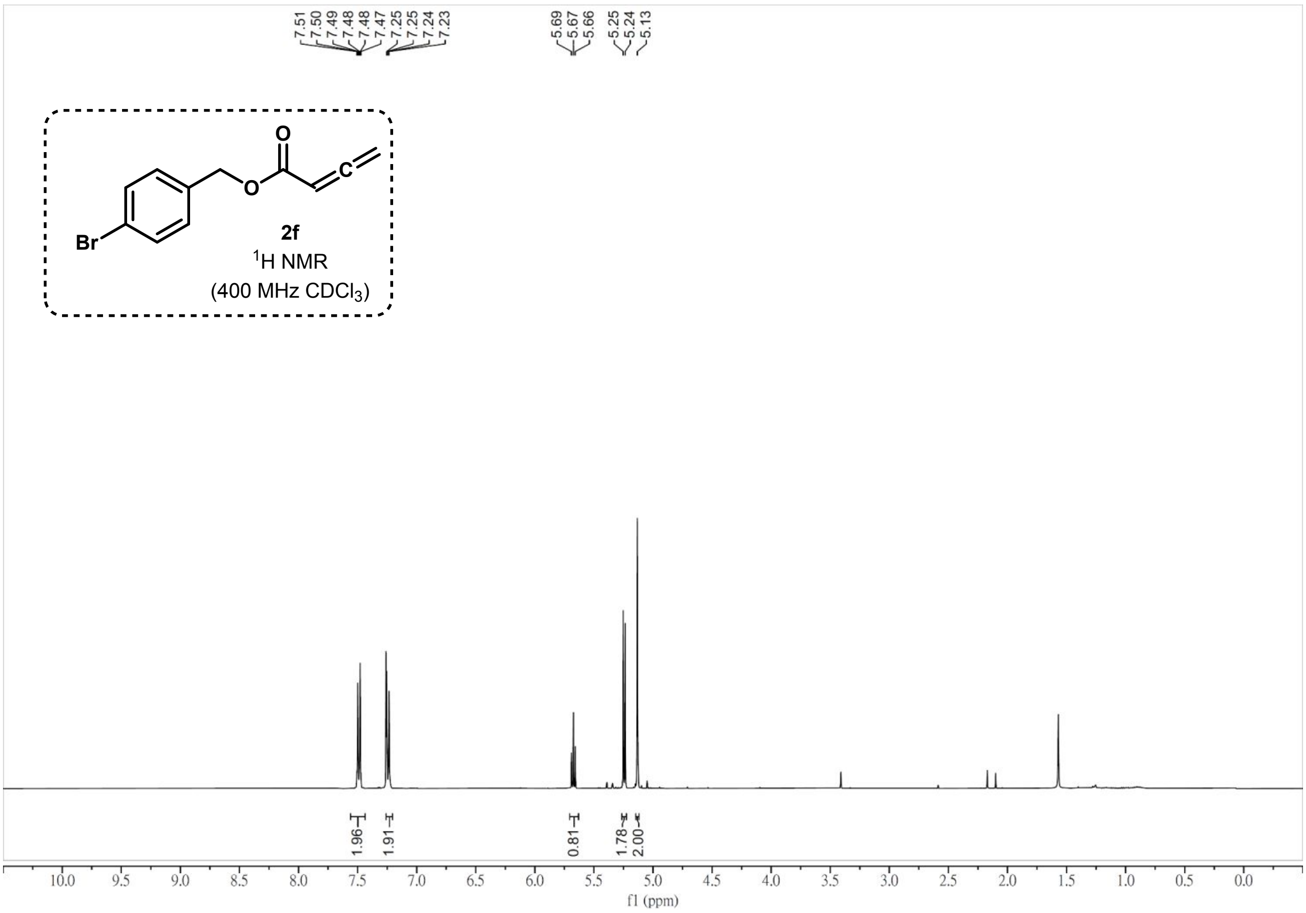
7.51
7.50
7.49
7.48
7.48
7.47
7.25
7.25
7.24
7.23
5.69
5.67
5.66
5.25
5.24
5.13
2f
^{1}H NMR
(400 MHz CDCl$_3$)
1.96
1.91
0.81
1.78
2.00
f1 (ppm)

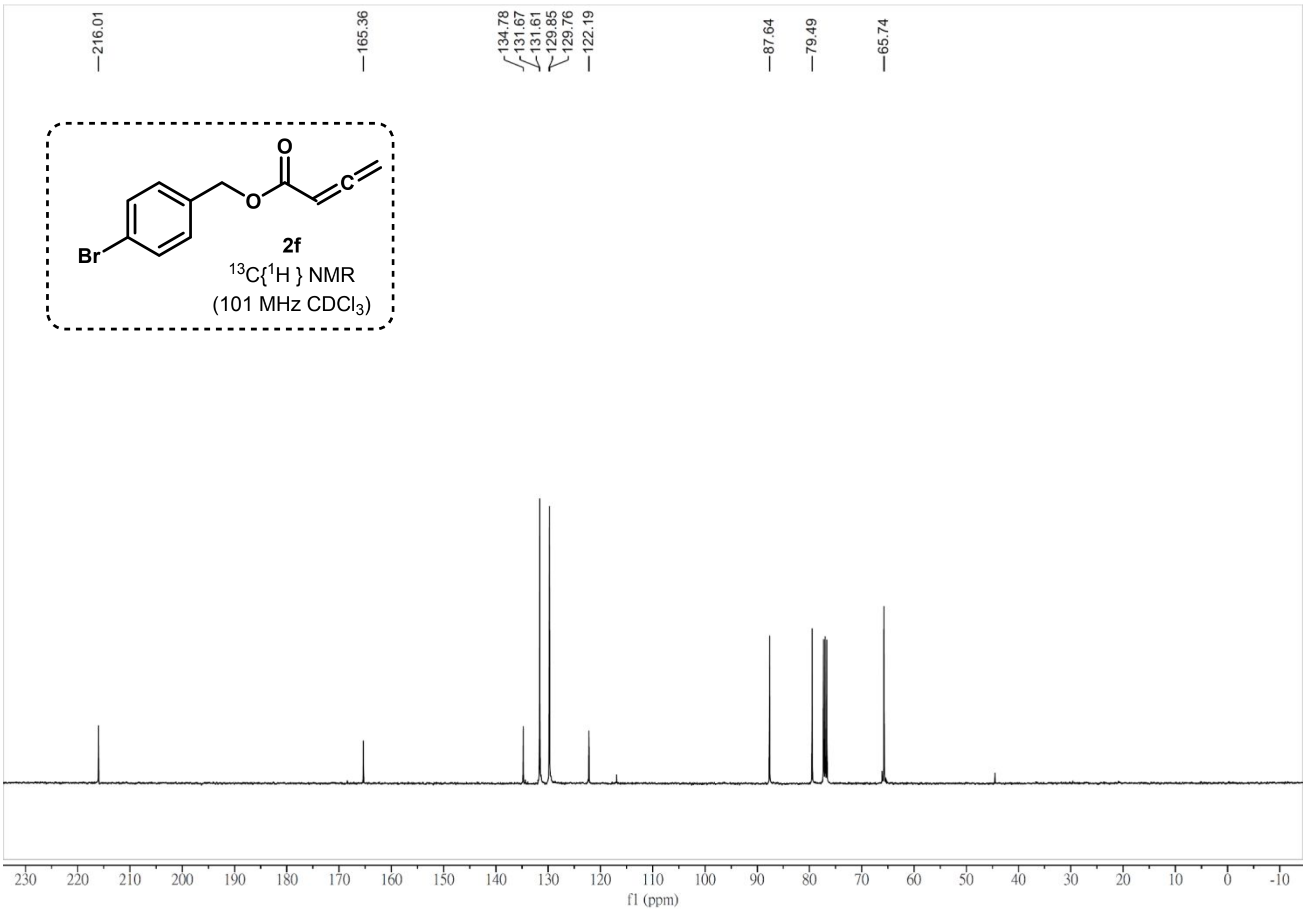
216.01
165.36
134.78
131.67
131.61
129.85
129.76
122.19
87.64
79.49
65.74
2f
^{13}C{^{1}H } NMR
(101 MHz CDCl$_3$)
f1 (ppm)

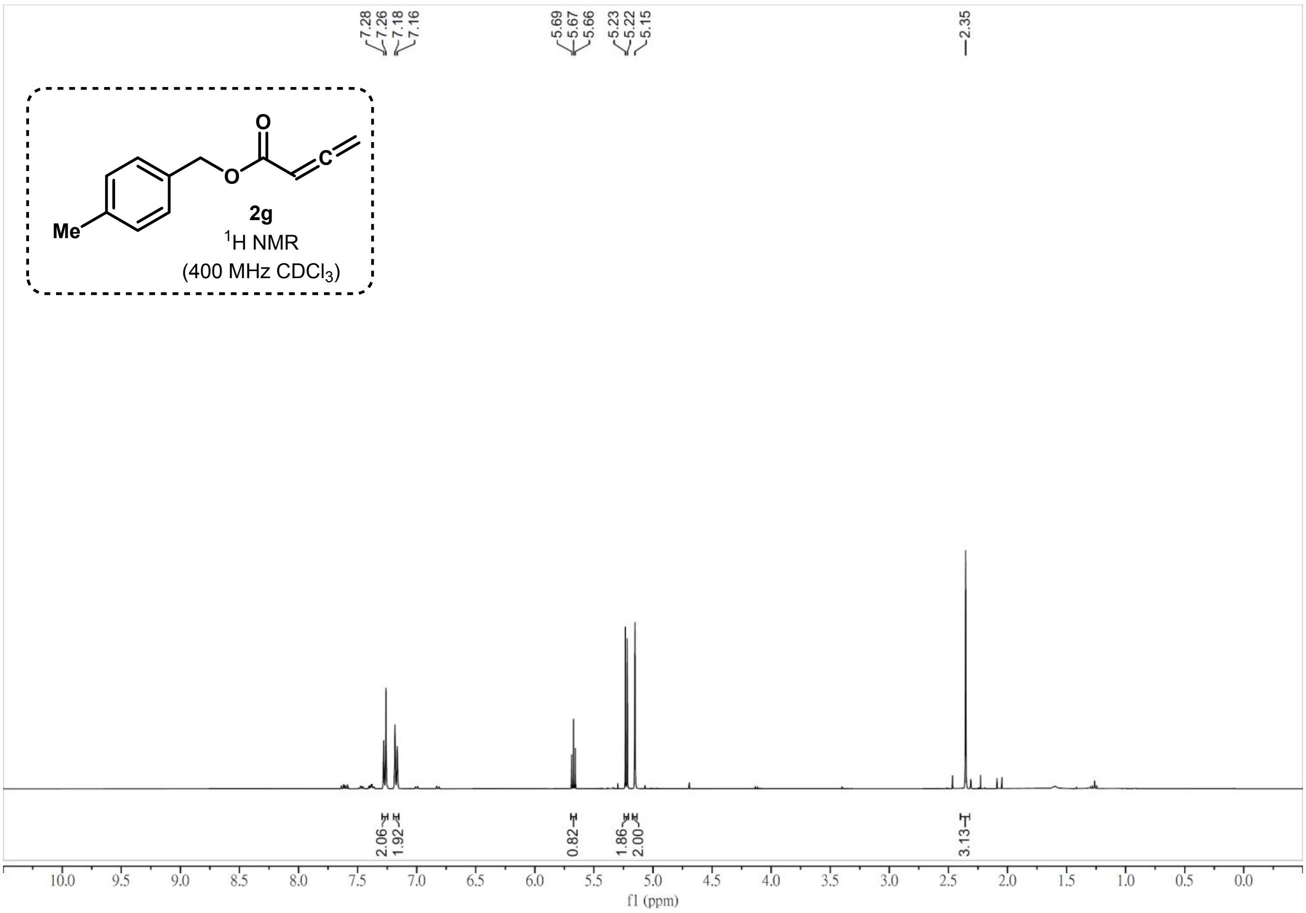
7.28
7.26
7.18
7.16
5.69
5.67
5.66
5.23
5.22
5.15
2.35
O
O
C
Me
2g
1H NMR
(400 MHz CDCl3)
2.06
1.92
0.82
1.86
2.00
3.13
f1 (ppm)

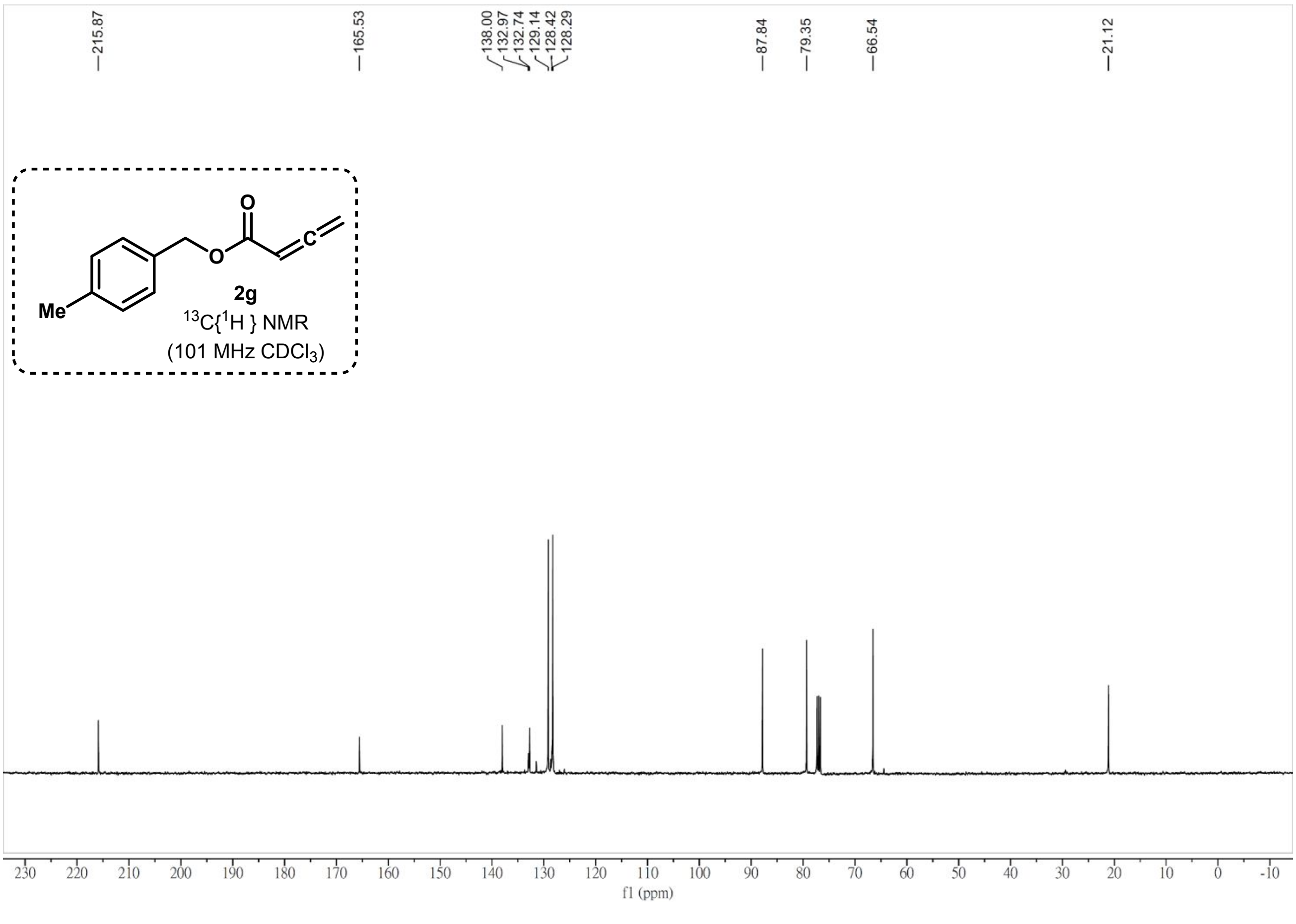
215.87
165.53
138.00
132.97
132.74
129.14
128.42
128.29
87.84
79.35
66.54
21.12
O
O
C
Me
2g
13C{1H } NMR
(101 MHz CDCl3)
f1 (ppm)

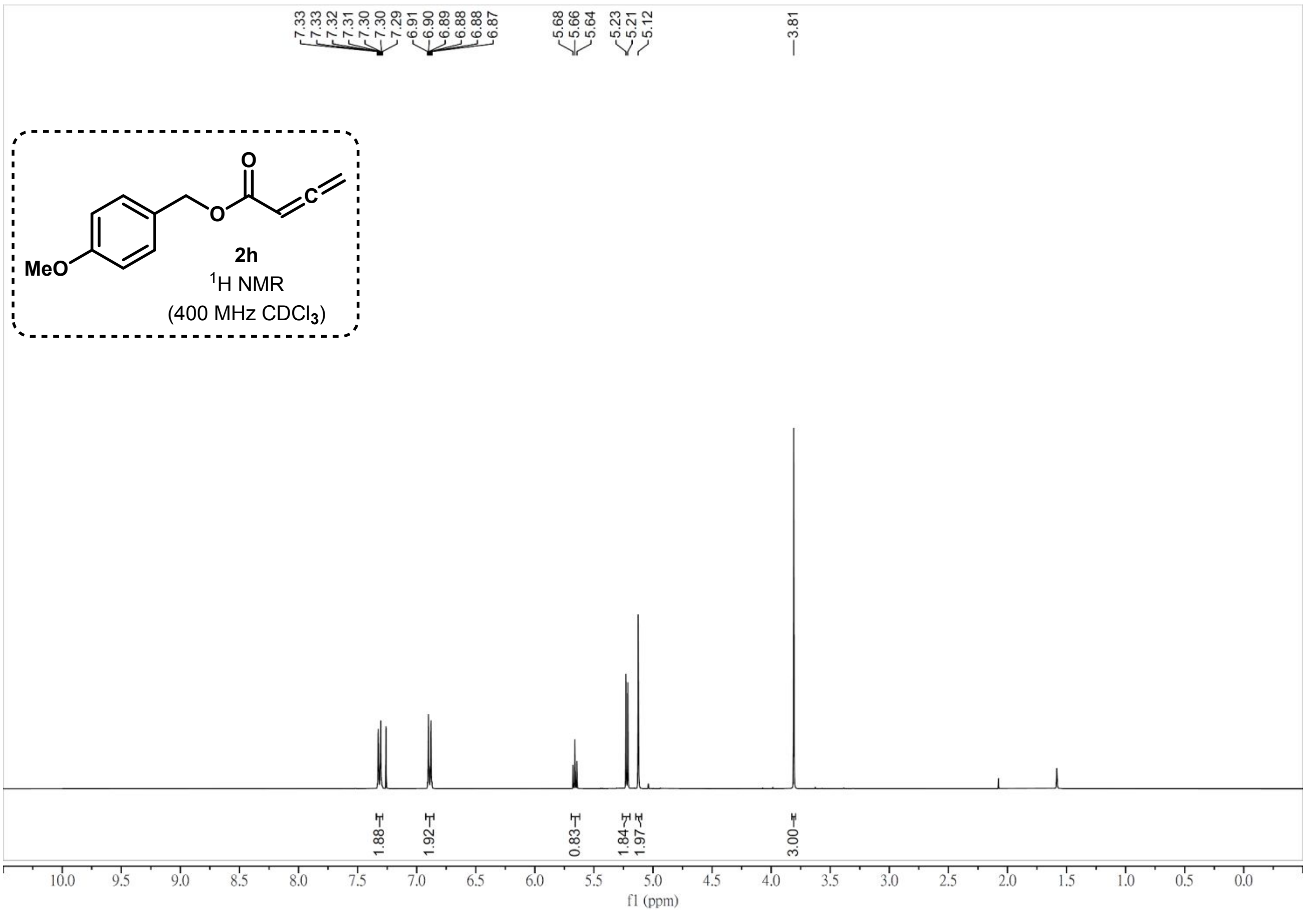

7.33
7.33
7.32
7.31
7.30
7.30
7.29
6.91
6.90
6.89
6.88
6.88
6.87
5.68
5.66
5.64
5.23
5.21
5.12
3.81
MeO
O
O
C
2h
^{1}H NMR
(400 MHz CDCl$_3$)
1.88
1.92
0.83
1.84
1.97
3.00
f1 (ppm)

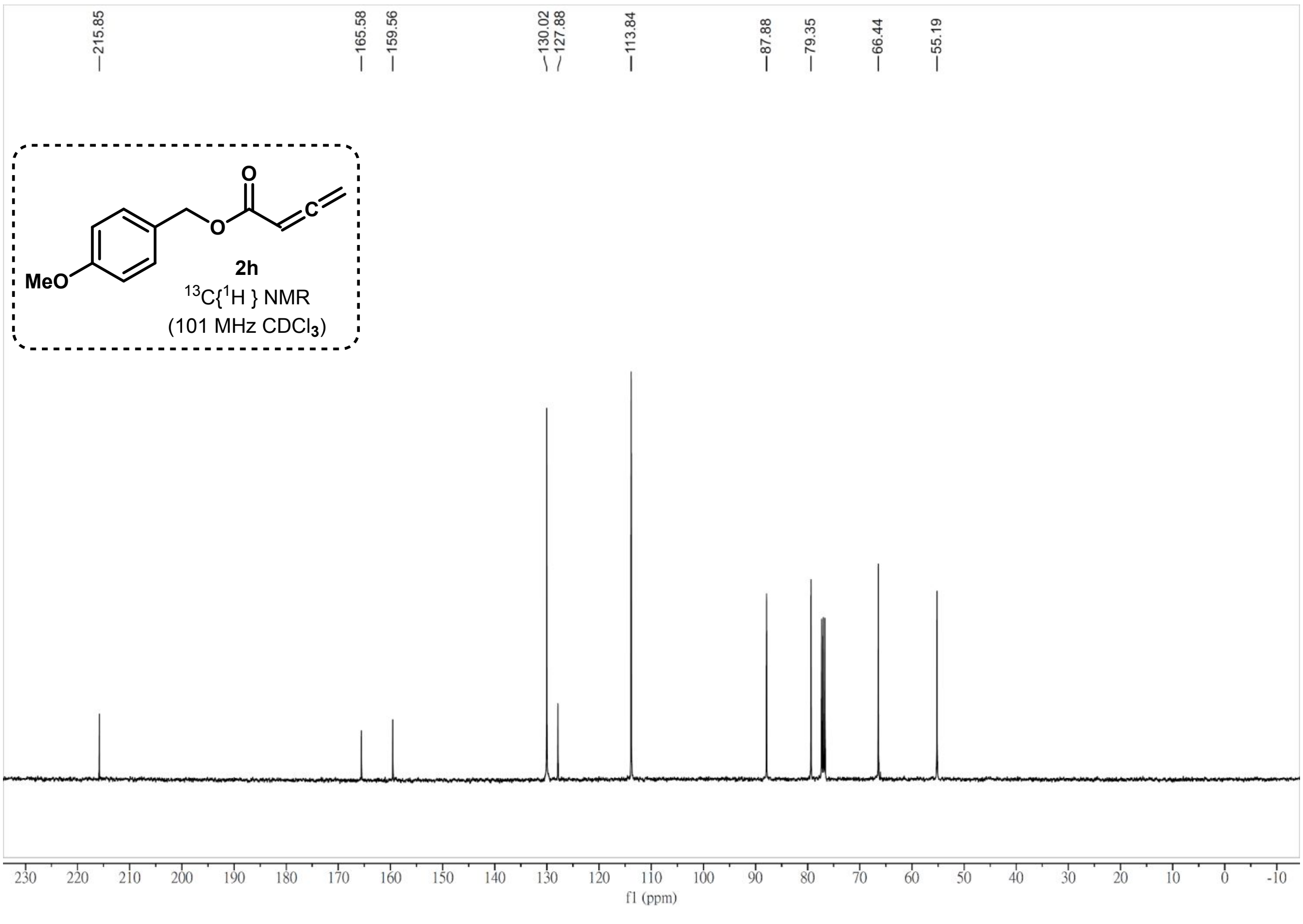

215.85
165.58
159.56
130.02
127.88
113.84
87.88
79.35
66.44
55.19
MeO
O
O
C
2h
^{13}C{^{1}H } NMR
(101 MHz CDCl$_3$)
f1 (ppm)

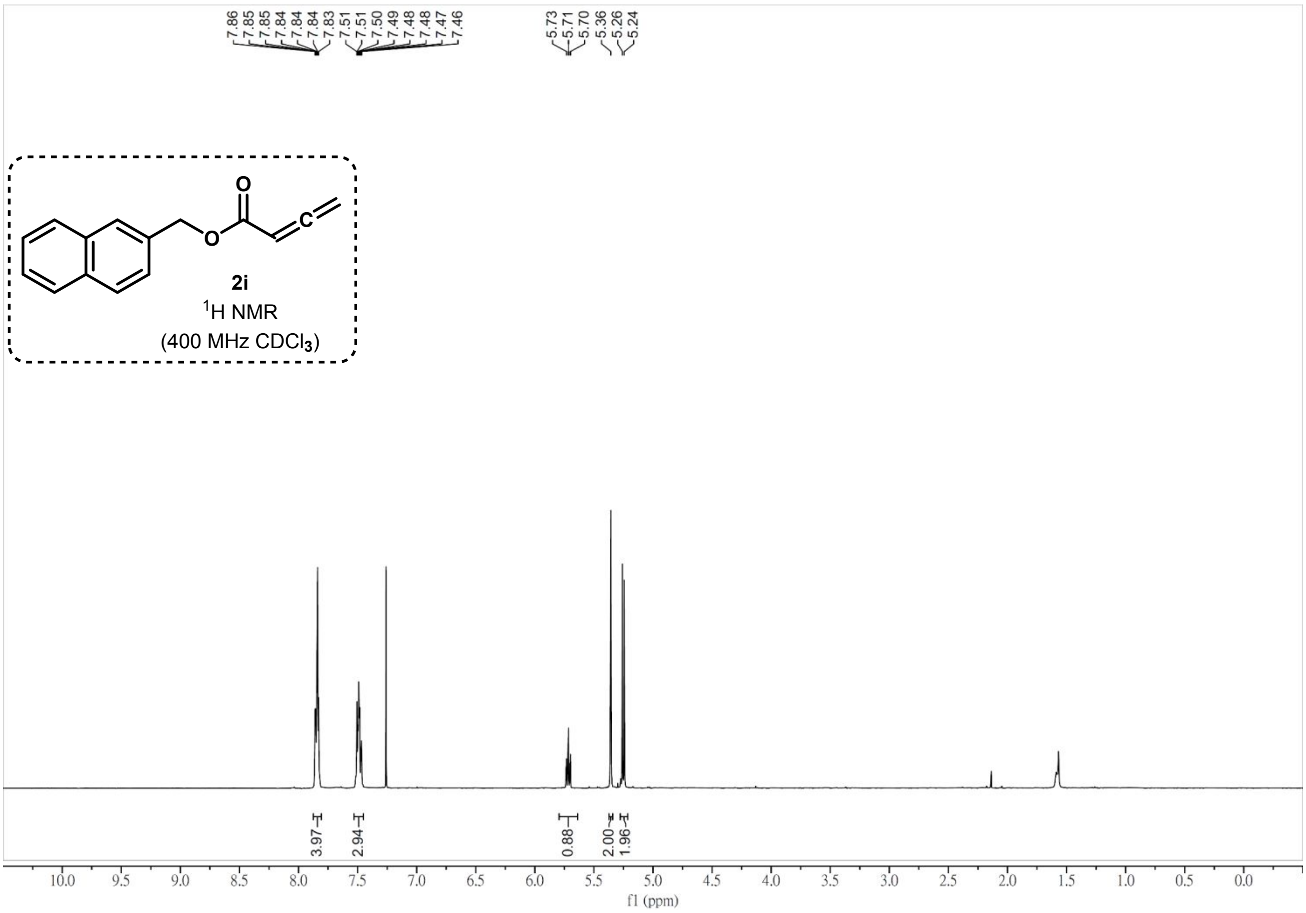
7.86
7.85
7.85
7.84
7.84
7.84
7.83
7.51
7.51
7.50
7.49
7.48
7.48
7.47
7.46
5.73
5.71
5.70
5.36
5.26
5.24
O
O
C
2i
1H NMR
(400 MHz CDCl3)
3.97
2.94
0.88
2.00
1.96
f1 (ppm)

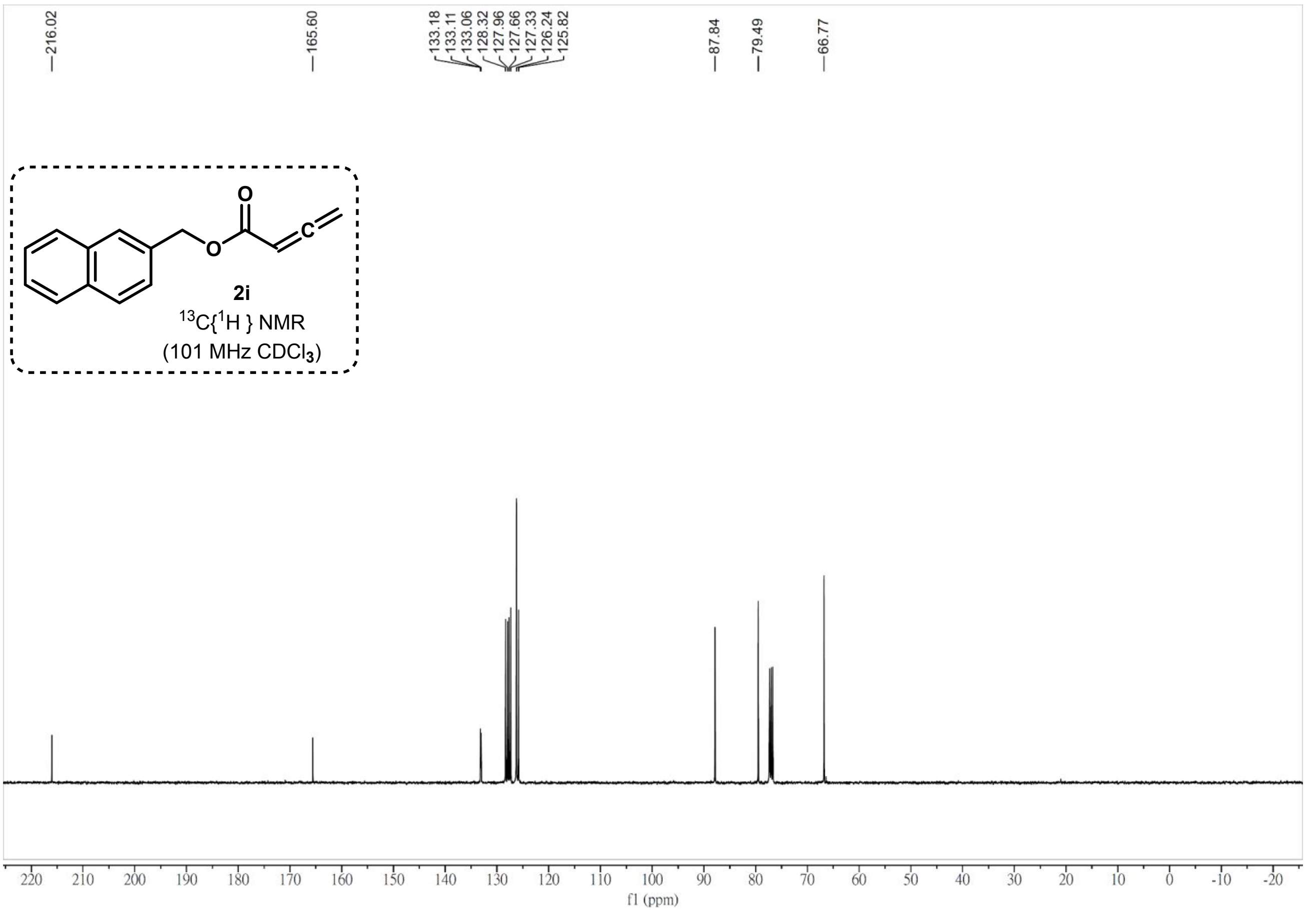
216.02
165.60
133.18
133.11
133.06
128.32
127.96
127.66
127.33
126.24
125.82
87.84
79.49
66.77
O
O
C
2i
13C{1H } NMR
(101 MHz CDCl3)
f1 (ppm)

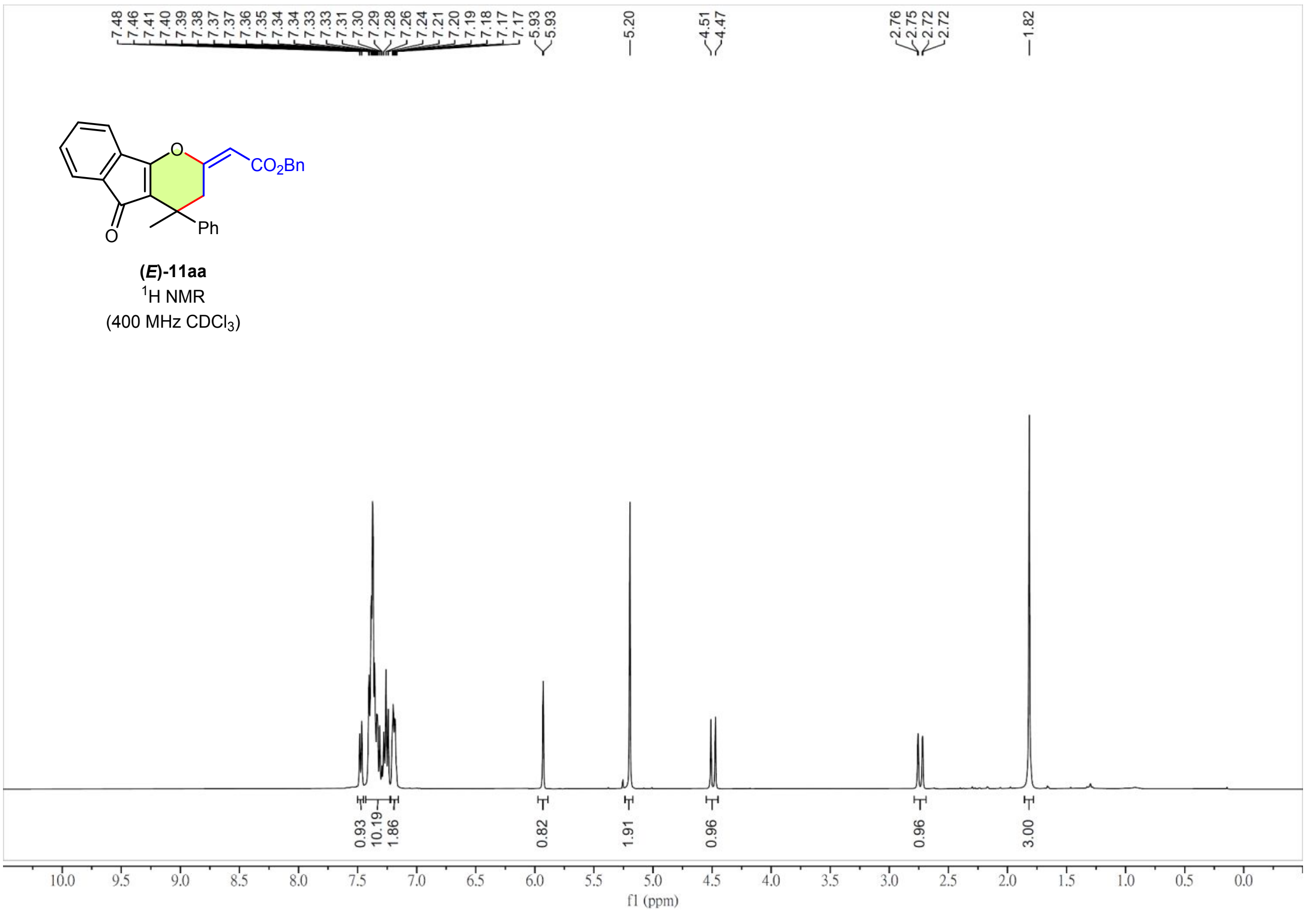
7.48
7.46
7.41
7.40
7.39
7.38
7.37
7.37
7.36
7.35
7.34
7.33
7.33
7.31
7.30
7.29
7.28
7.26
7.24
7.21
7.20
7.19
7.18
7.17
7.17
5.93
5.93
5.20
4.51
4.47
2.76
2.75
2.72
2.72
1.82
O
CO_2Bn
O
Ph
(*E*)-11aa
^1H NMR
(400 MHz $CDCl_3$)
0.93
10.19
1.86
0.82
1.91
0.96
0.96
3.00
f1 (ppm)

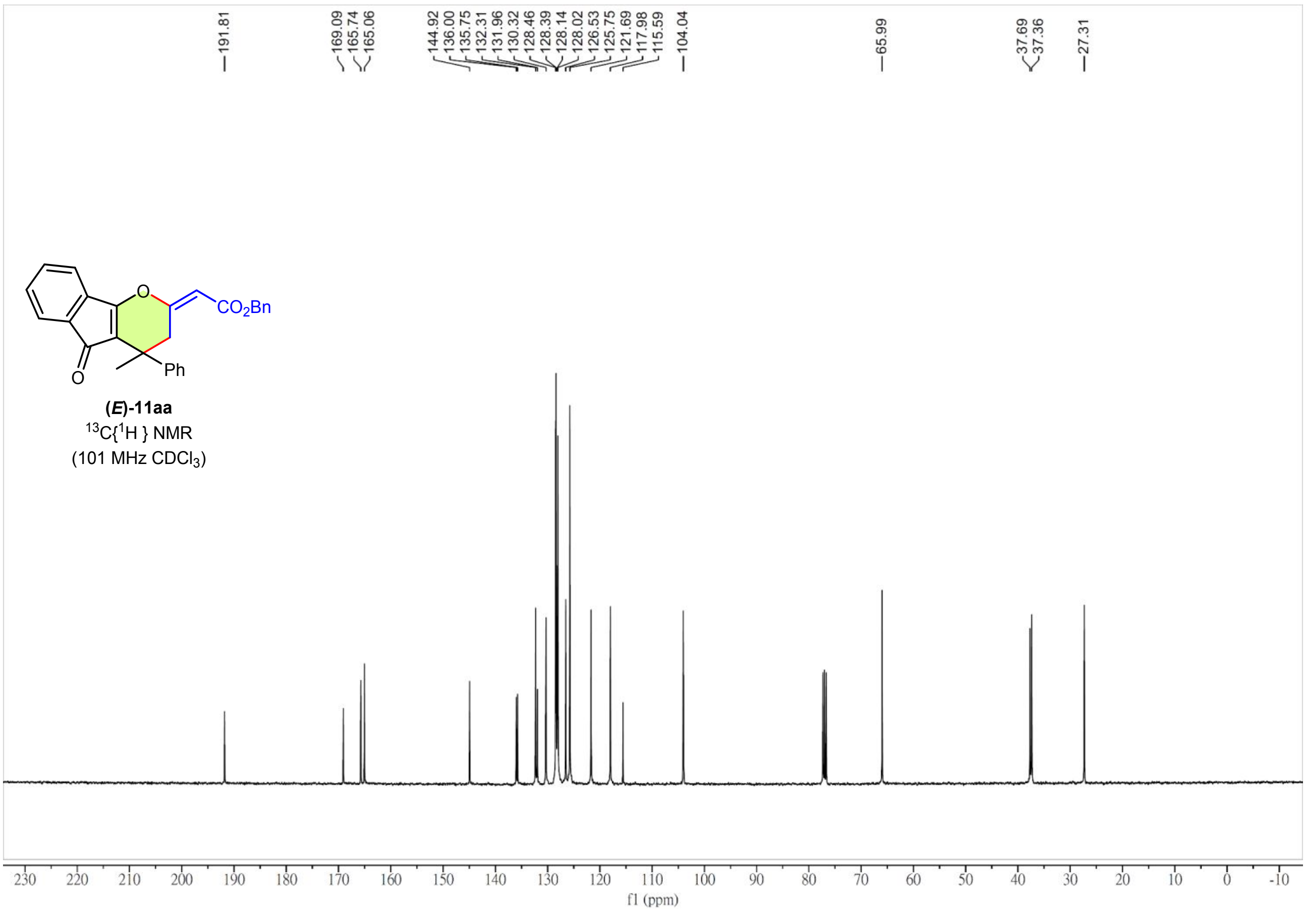
191.81
169.09
165.74
165.06
144.92
136.00
135.75
132.31
131.96
130.32
128.46
128.39
128.14
128.02
126.53
125.75
121.69
117.98
115.59
104.04
65.99
37.69
37.36
27.31
O
CO_2Bn
O
Ph
(*E*)-11aa
$^{13}C\{^1H\}$ NMR
(101 MHz $CDCl_3$)
f1 (ppm)

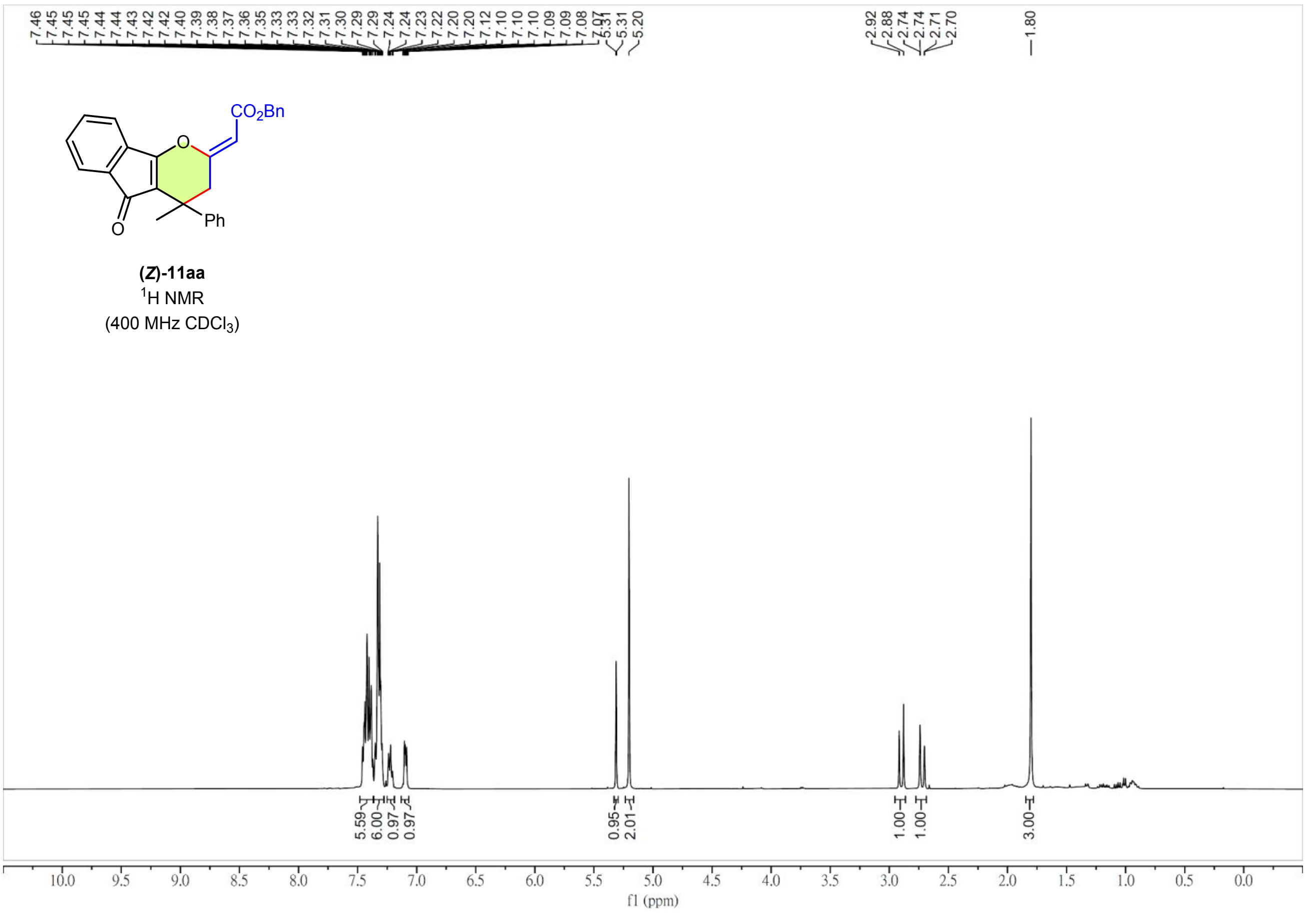
CO2Bn
O
O
Ph
(Z)-11aa
1H NMR
(400 MHz CDCl3)
7.46 7.45 7.45 7.44 7.44 7.43 7.42 7.42 7.40 7.39 7.38 7.37 7.36 7.35 7.33 7.33 7.32 7.31 7.30 7.29 7.29 7.24 7.24 7.23 7.22 7.20 7.20 7.12 7.10 7.10 7.10 7.09 7.09 7.08 7.07 5.31 5.31 5.20
2.92 2.88 2.74 2.74 2.71 2.70
1.80
5.59 6.00 0.97 0.97 0.95 2.01 1.00 1.00 3.00
f1 (ppm)

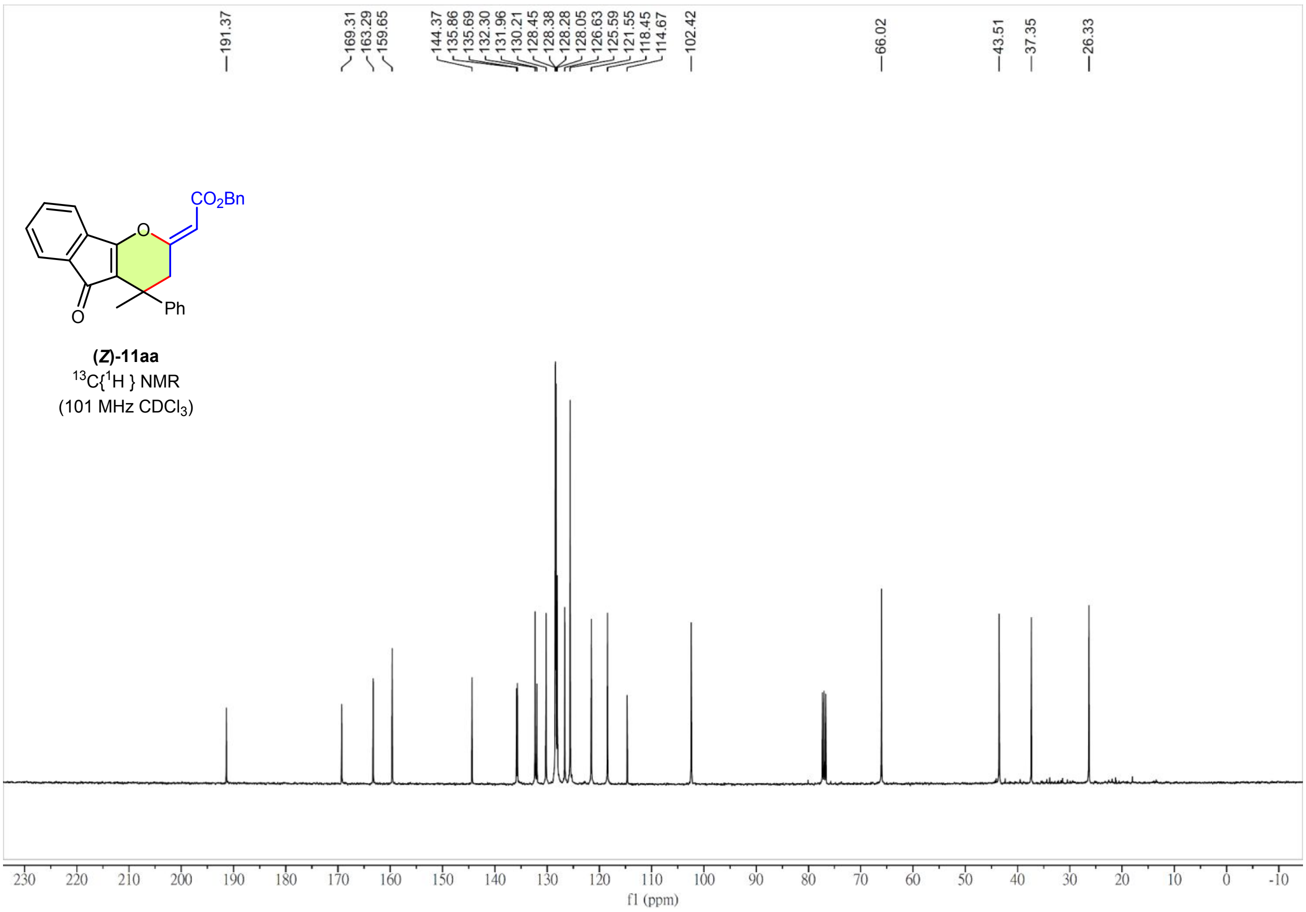
CO2Bn
O
O
Ph
(Z)-11aa
13C{1H } NMR
(101 MHz CDCl3)
191.37
169.31 163.29 159.65
144.37 135.86 135.69 132.30 131.96 130.21 128.45 128.38 128.28 128.05 126.63 125.59 121.55 118.45 114.67
102.42
66.02
43.51 37.35
26.33
f1 (ppm)

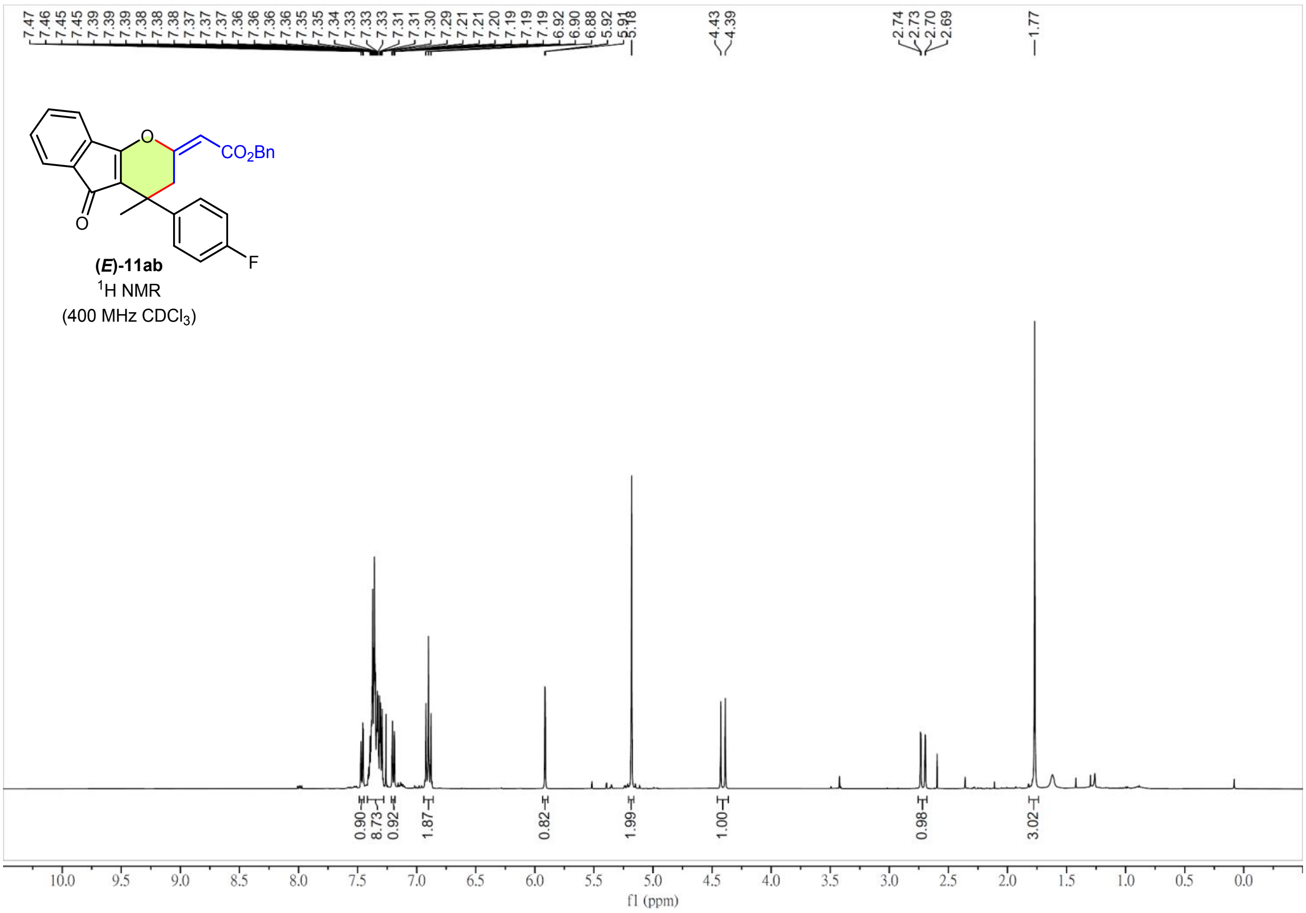
CO2Bn
O
O
F
(E)-11ab
1H NMR
(400 MHz CDCl3)
f1 (ppm)

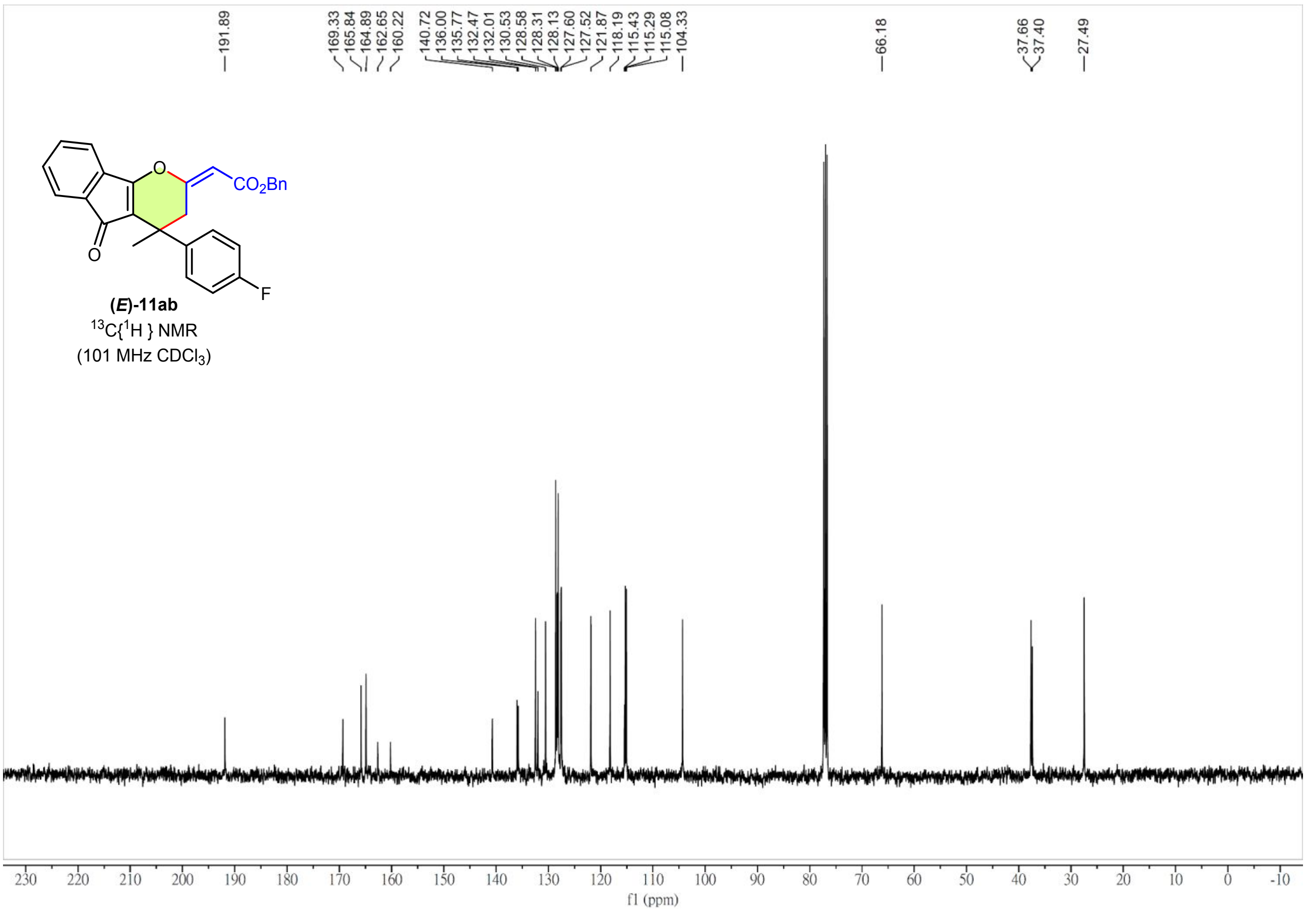
CO2Bn
O
O
F
(E)-11ab
13C{1H } NMR
(101 MHz CDCl3)
f1 (ppm)

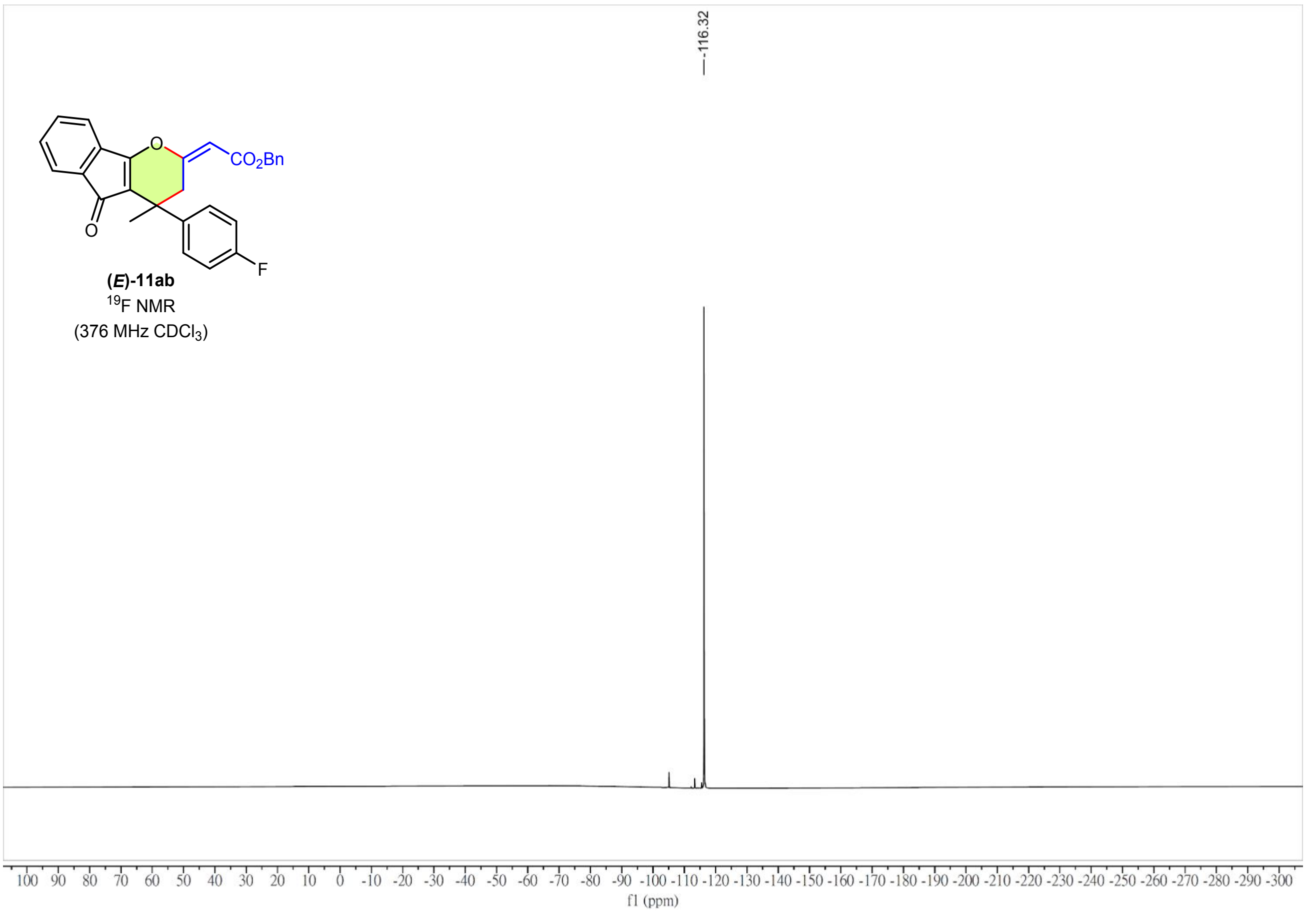

-116.32
CO2Bn
O
O
F
(E)-11ab
19F NMR
(376 MHz CDCl3)
100 90 80 70 60 50 40 30 20 10 0 -10 -20 -30 -40 -50 -60 -70 -80 -90 -100 -110 -120 -130 -140 -150 -160 -170 -180 -190 -200 -210 -220 -230 -240 -250 -260 -270 -280 -290 -300
f1 (ppm)

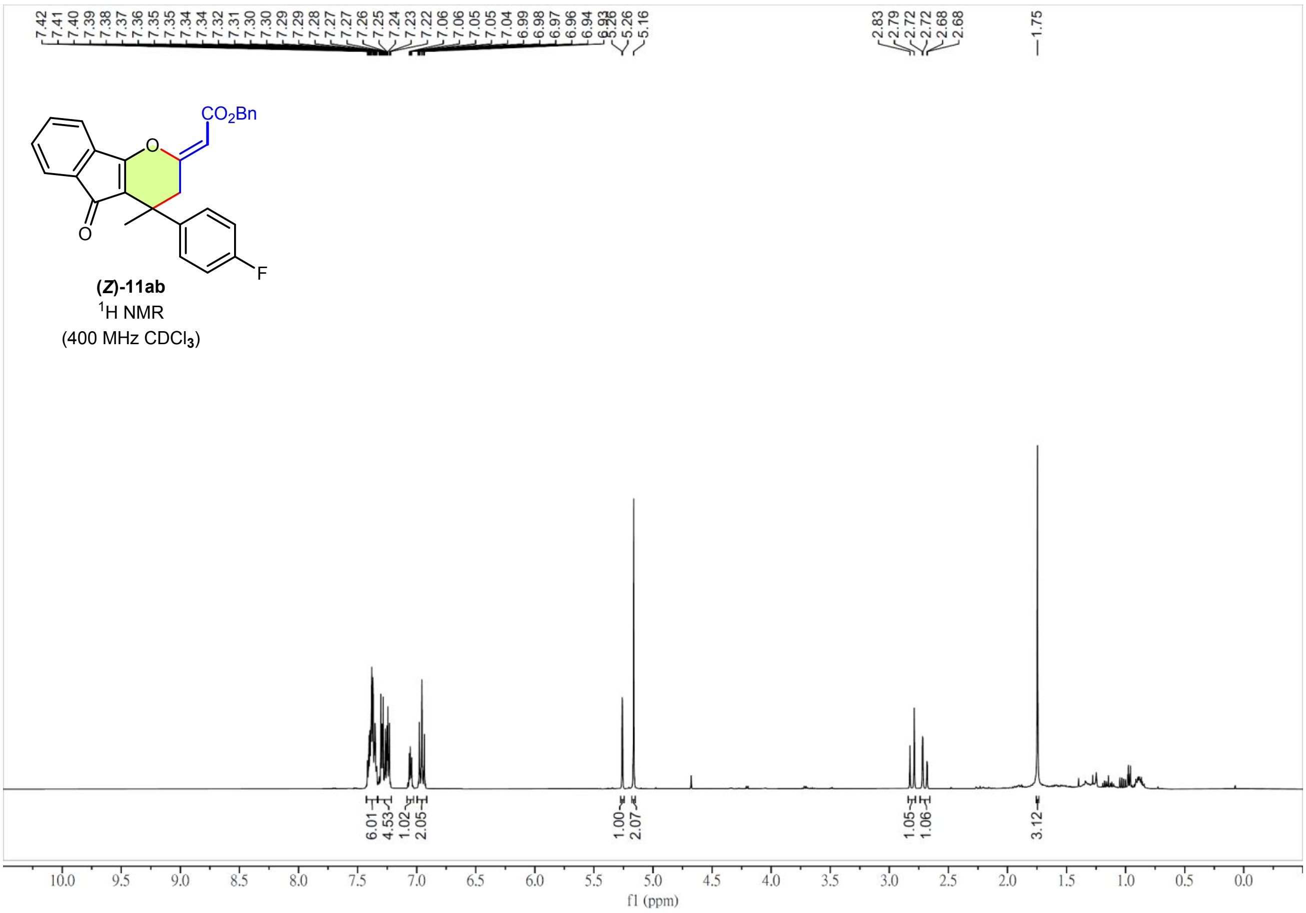
(Z)-11ab
1H NMR
(400 MHz CDCl3)
f1 (ppm)

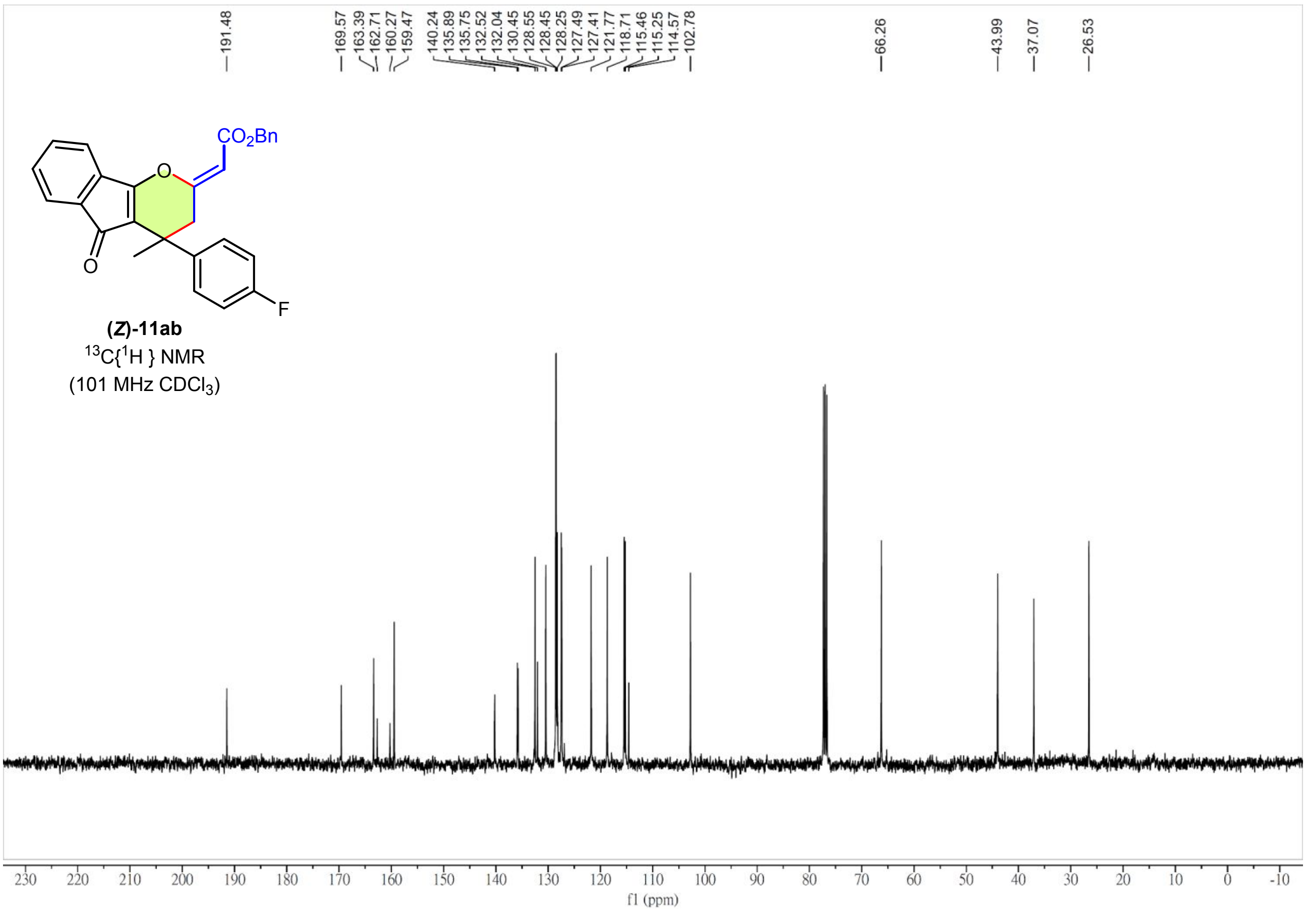
(Z)-11ab
13C{1H } NMR
(101 MHz CDCl3)
f1 (ppm)

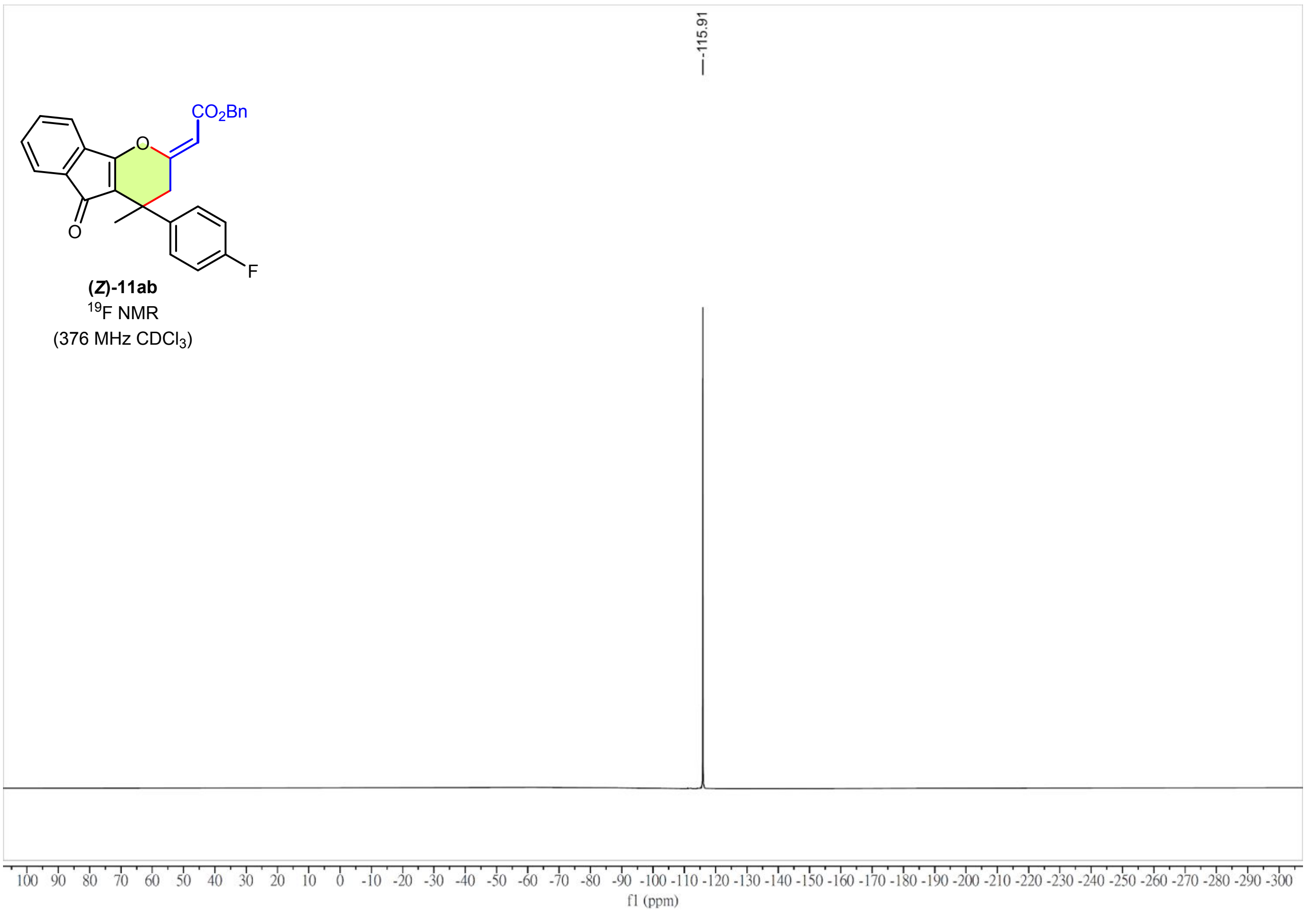

-115.91
CO2Bn
O
O
F
(Z)-11ab
19F NMR
(376 MHz CDCl3)
100 90 80 70 60 50 40 30 20 10 0 -10 -20 -30 -40 -50 -60 -70 -80 -90 -100 -110 -120 -130 -140 -150 -160 -170 -180 -190 -200 -210 -220 -230 -240 -250 -260 -270 -280 -290 -300
f1 (ppm)

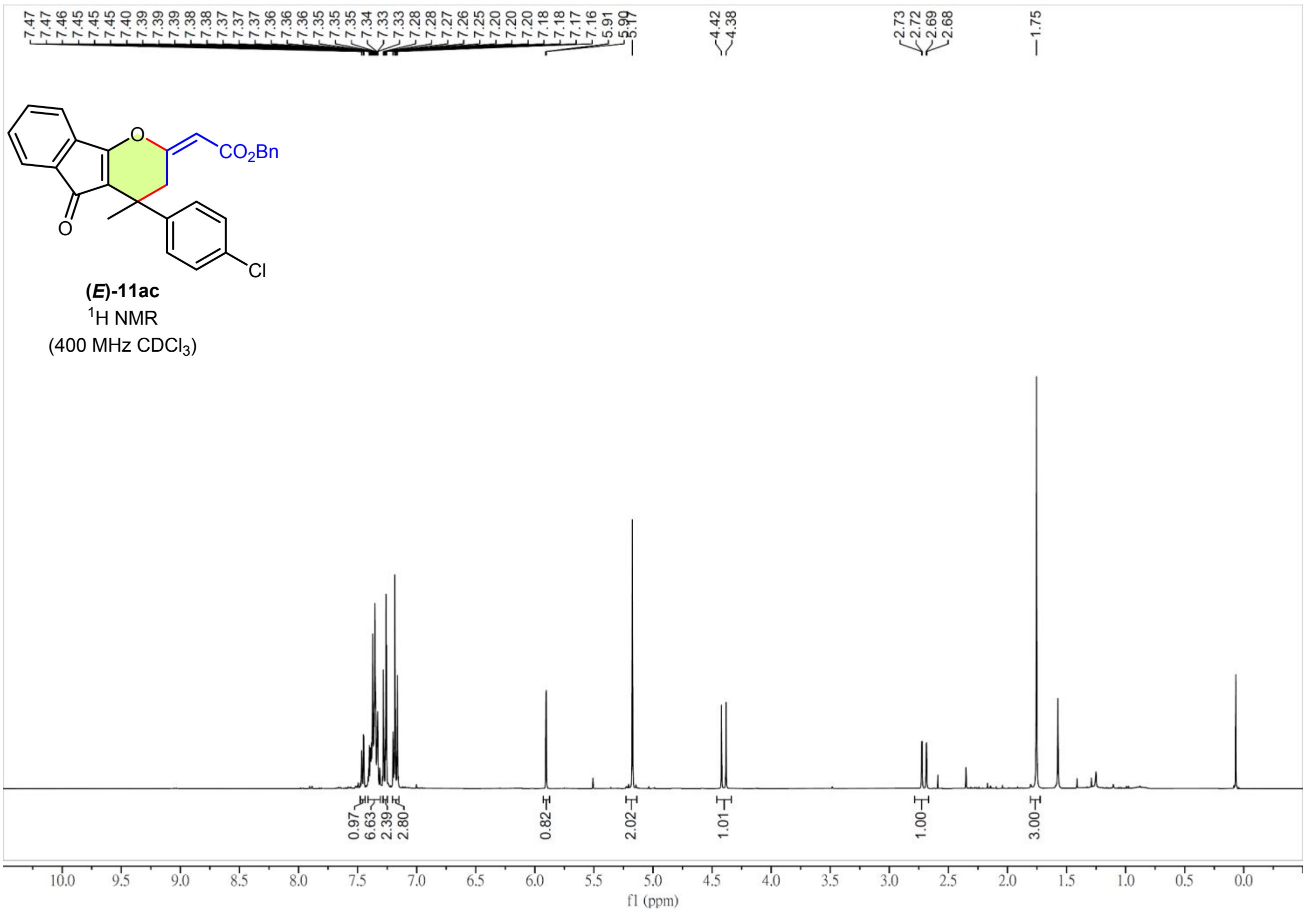

(*E*)-11ac
^{1}H NMR
(400 MHz $CDCl_3$)

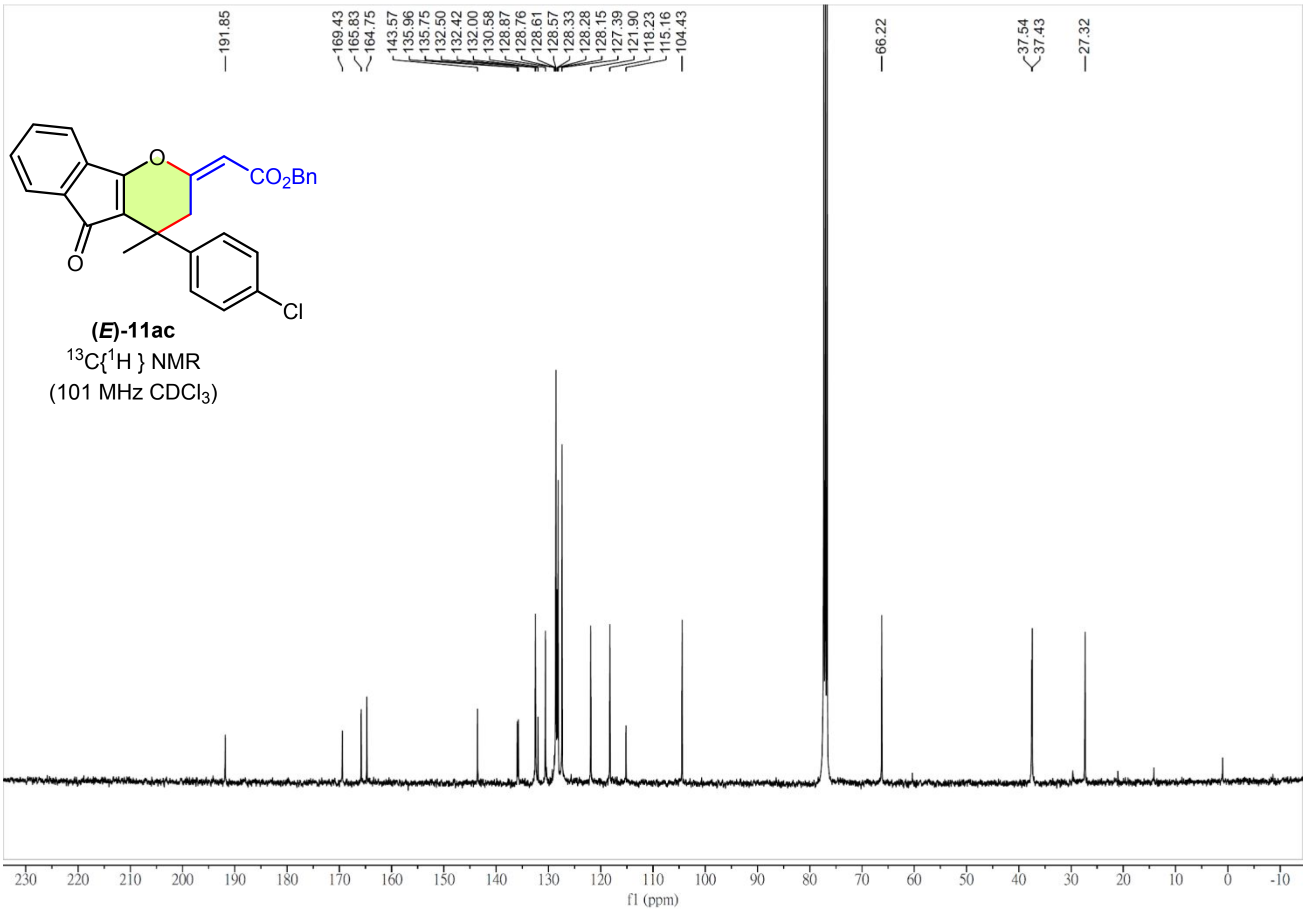

(*E*)-11ac
$^{13}C\{^1H\}$ NMR
(101 MHz $CDCl_3$)

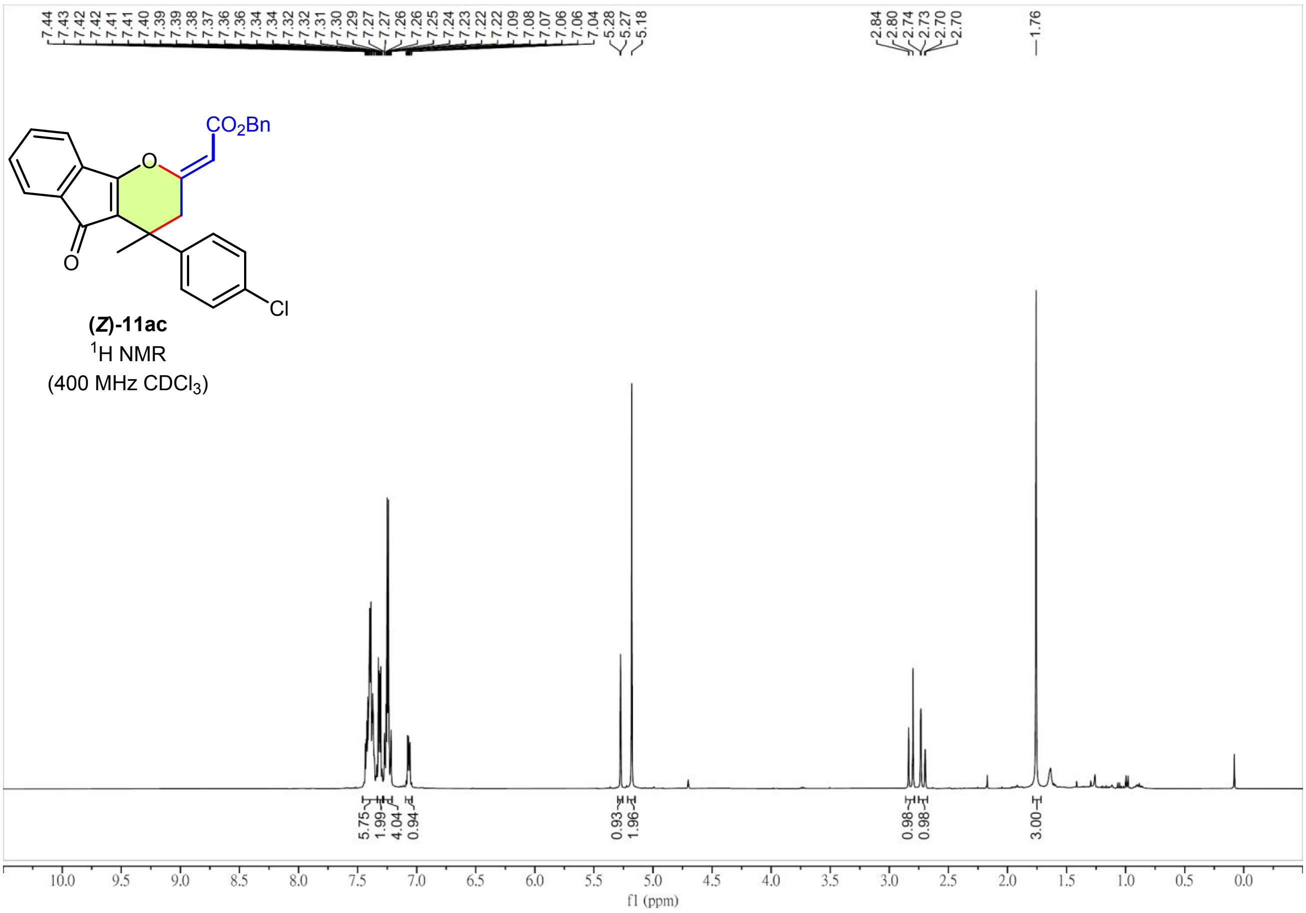
7.44 7.43 7.42 7.42 7.41 7.40 7.39 7.39 7.38 7.37 7.36 7.36 7.34 7.34 7.32 7.32 7.31 7.30 7.29 7.27 7.27 7.26 7.26 7.25 7.24 7.23 7.22 7.22 7.09 7.08 7.07 7.06 7.06 7.04 5.28 5.27 5.18
2.84 2.80 2.74 2.73 2.70 2.70
1.76
CO2Bn
O
O
Cl
(Z)-11ac
1H NMR
(400 MHz CDCl3)
5.75 1.99 4.04 0.94
0.93 1.96
0.98 0.98
3.00
f1 (ppm)

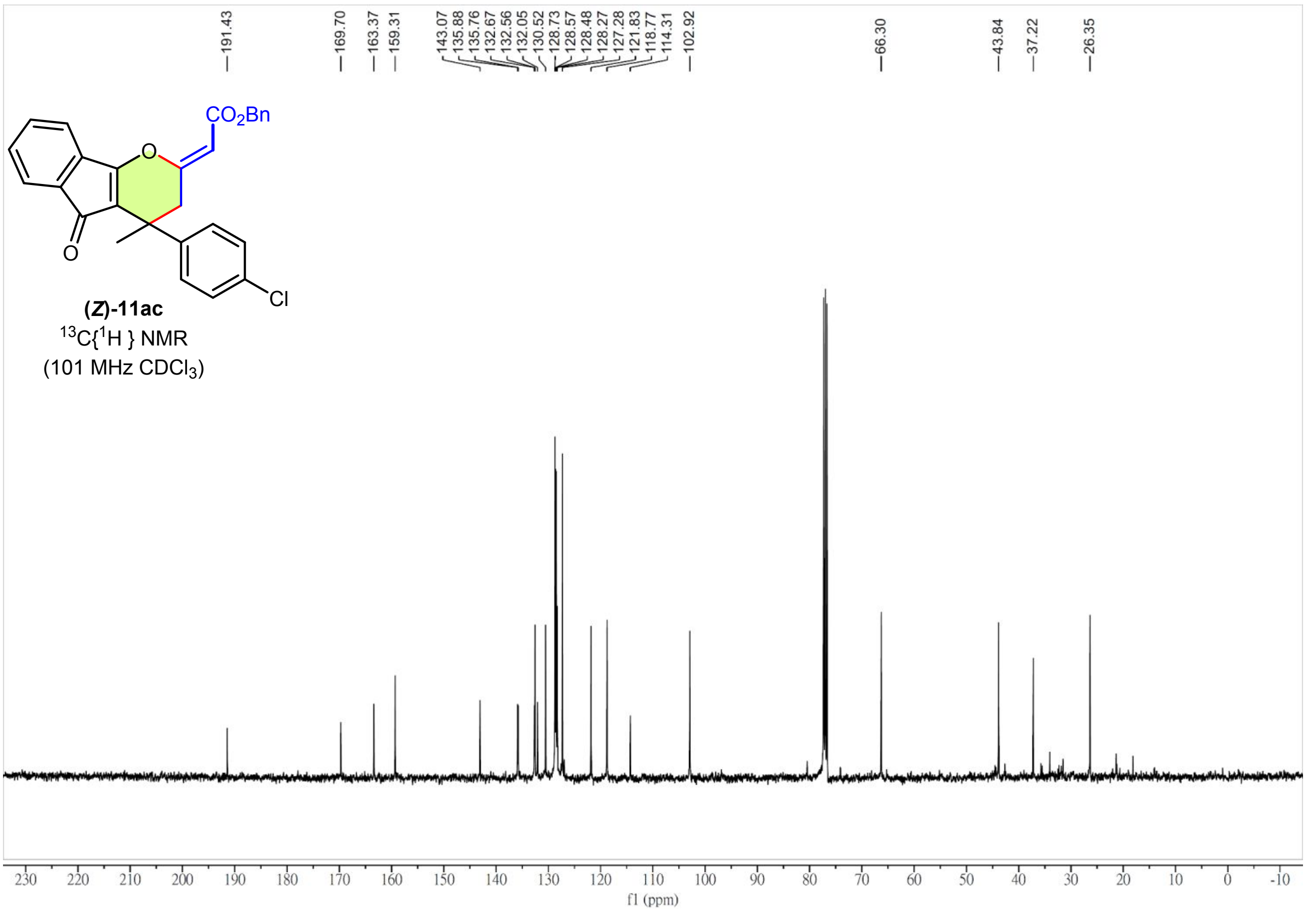
191.43
169.70 163.37 159.31
143.07 135.88 135.76 132.67 132.56 132.05 130.52 128.73 128.57 128.48 128.27 127.28 121.83 118.77 114.31
102.92
66.30
43.84 37.22 26.35
CO2Bn
O
O
Cl
(Z)-11ac
13C{1H } NMR
(101 MHz CDCl3)
f1 (ppm)

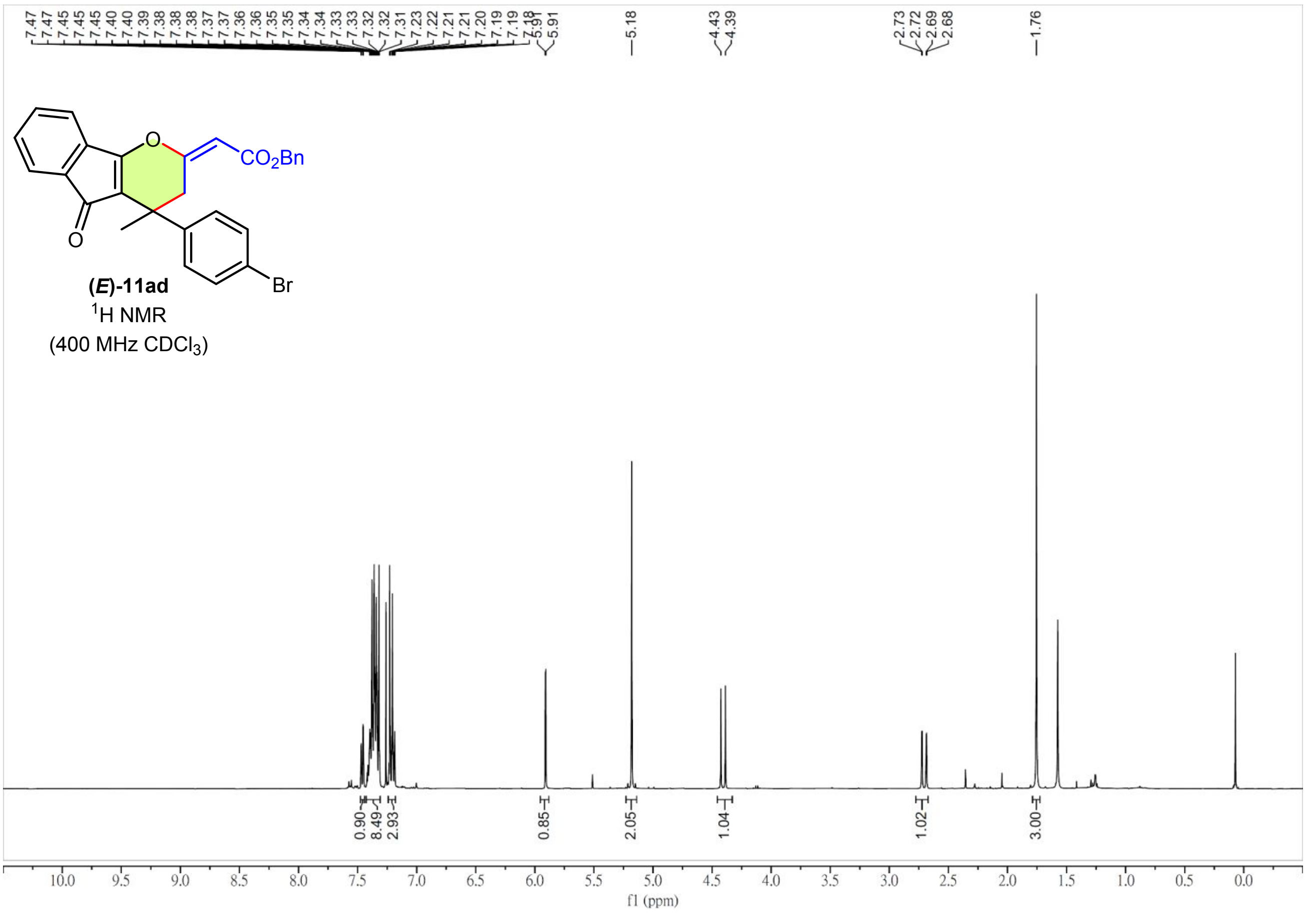
(E)-11ad
1H NMR
(400 MHz CDCl3)
f1 (ppm)

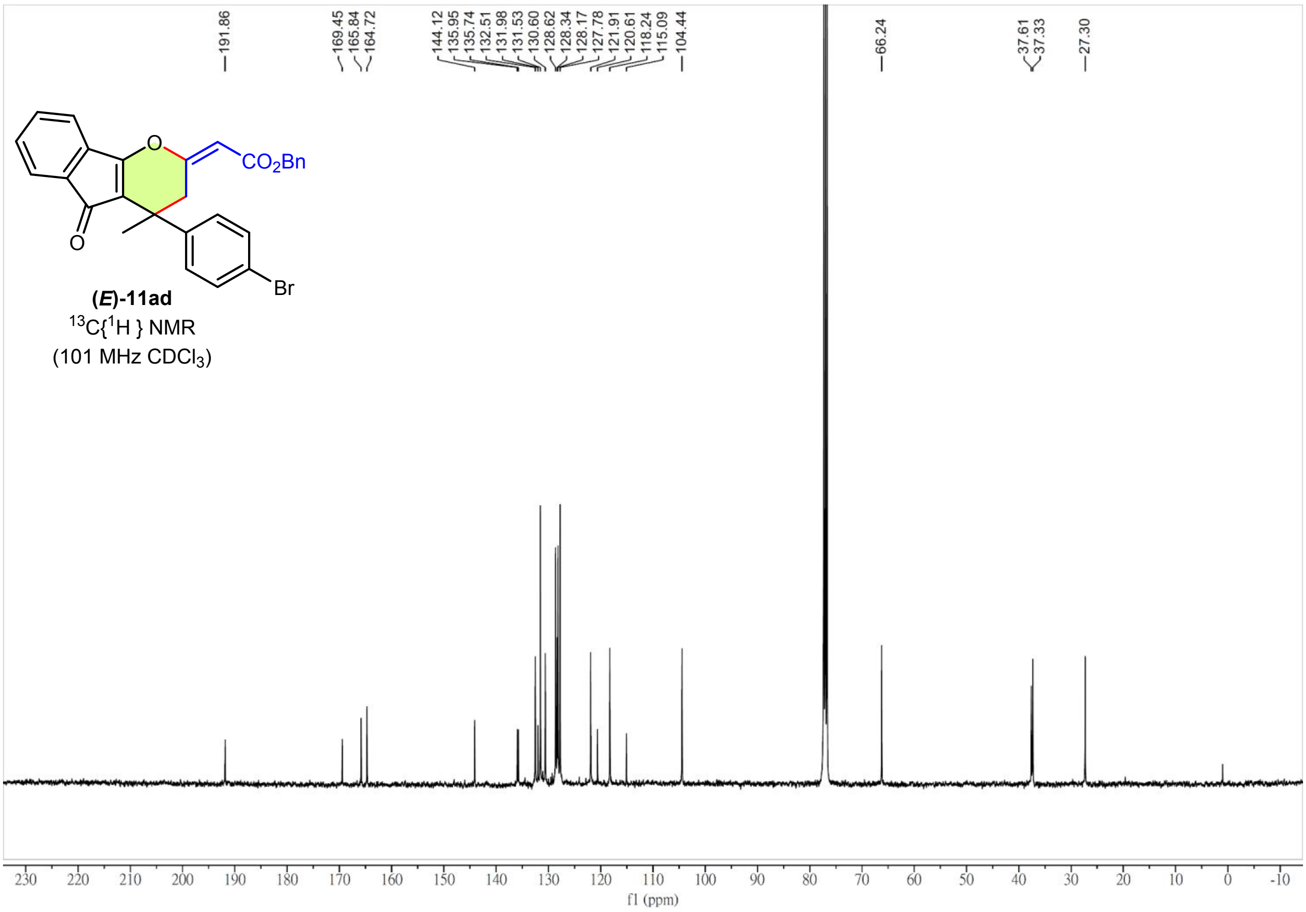
(E)-11ad
13C{1H } NMR
(101 MHz CDCl3)
f1 (ppm)

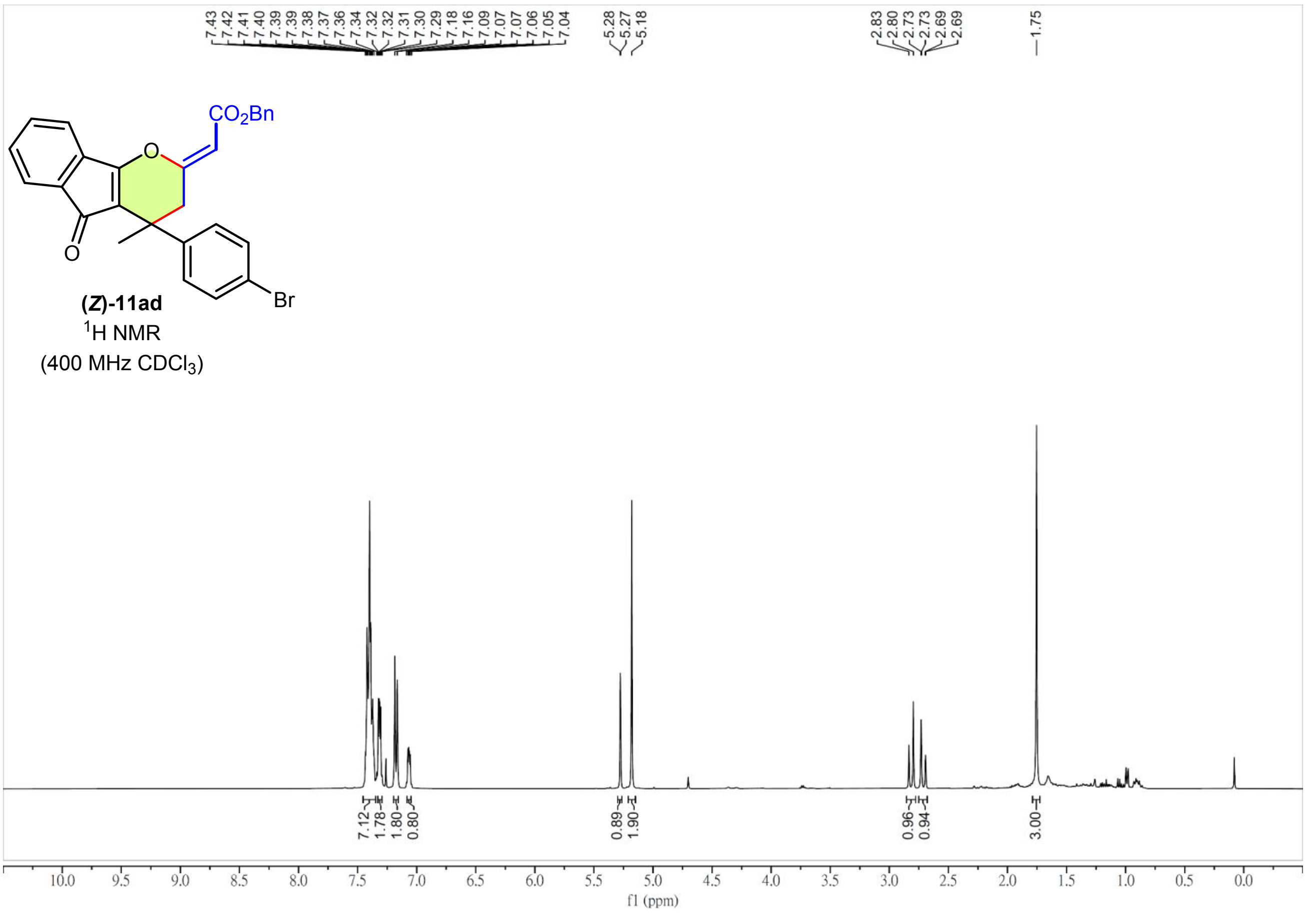
7.43 7.42 7.41 7.40 7.39 7.39 7.38 7.37 7.36 7.34 7.32 7.32 7.31 7.30 7.29 7.18 7.16 7.09 7.07 7.07 7.06 7.05 7.04
5.28 5.27 5.18
2.83 2.80 2.73 2.73 2.69 2.69
1.75
CO2Bn
O
O
Br
(Z)-11ad
1H NMR
(400 MHz CDCl3)
7.12 1.78 1.80 0.80
0.89 1.90
0.96 0.94
3.00
f1 (ppm)

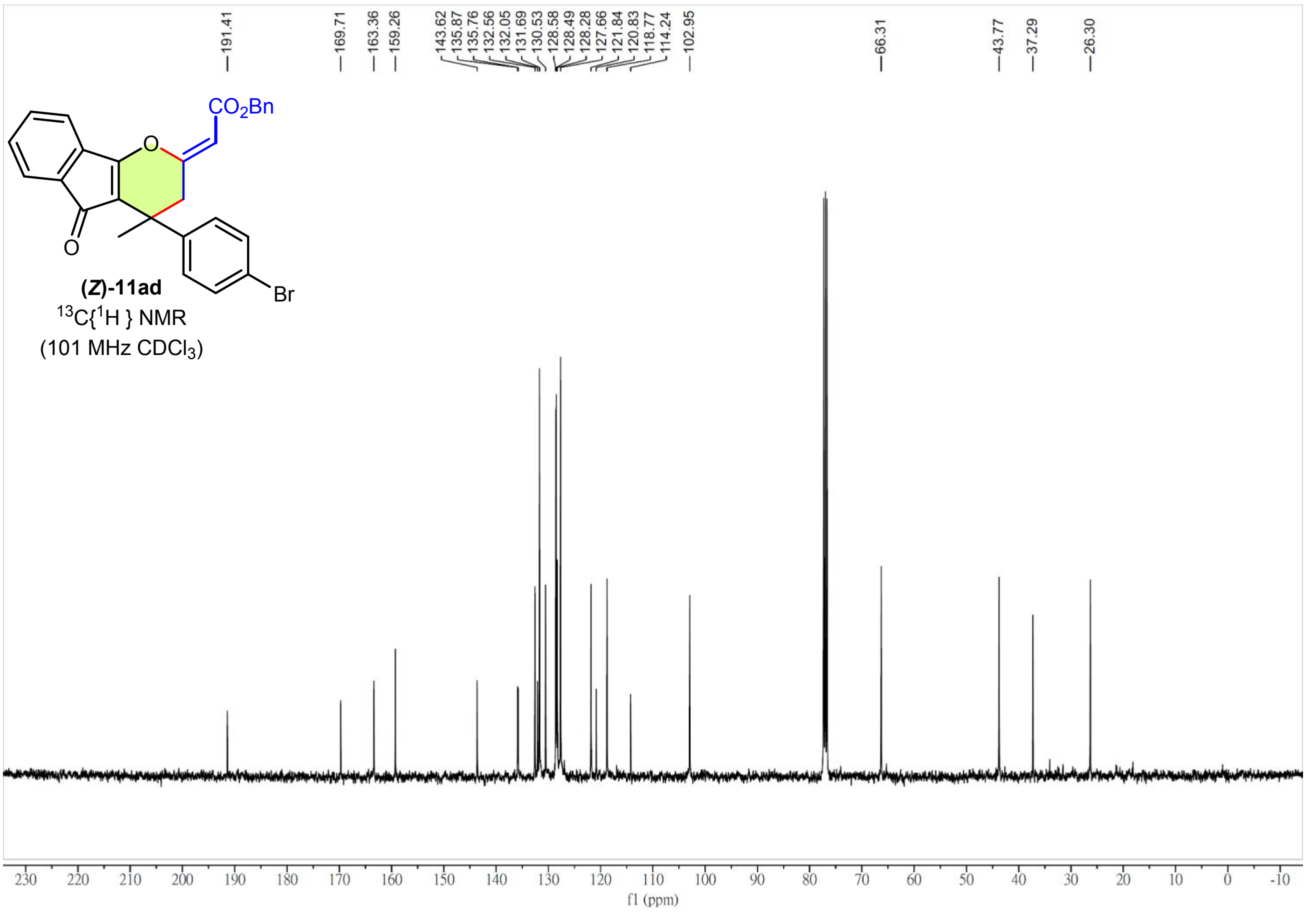
191.41
169.71 163.36 159.26
143.62 135.87 135.76 132.56 132.05 131.69 130.53 128.58 128.49 128.28 127.66 121.84 120.83 118.77 114.24
102.95
66.31
43.77 37.29 26.30
CO2Bn
O
O
Br
(Z)-11ad
13C{1H } NMR
(101 MHz CDCl3)
f1 (ppm)

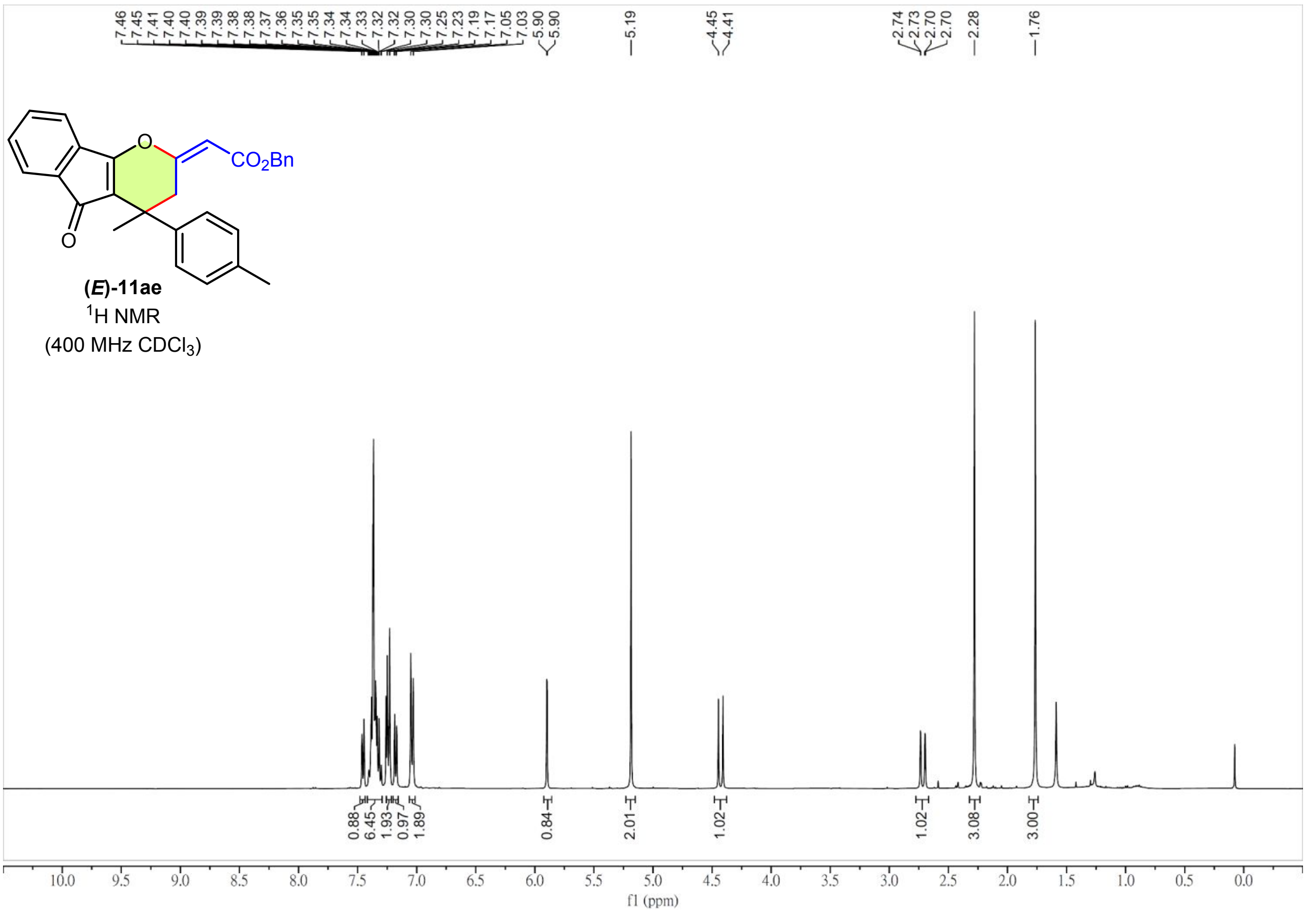

7.46 7.45 7.41 7.40 7.39 7.39 7.38 7.38 7.37 7.36 7.35 7.35 7.34 7.34 7.33 7.32 7.32 7.30 7.30 7.25 7.23 7.19 7.17 7.05 7.03 5.90 5.90
5.19
4.45 4.41
2.74 2.73 2.70 2.70
2.28
1.76
CO2Bn
O
O
(E)-11ae
1H NMR
(400 MHz CDCl3)
0.88 6.45 1.93 0.97 1.89
0.84
2.01
1.02
1.02
3.08
3.00
f1 (ppm)

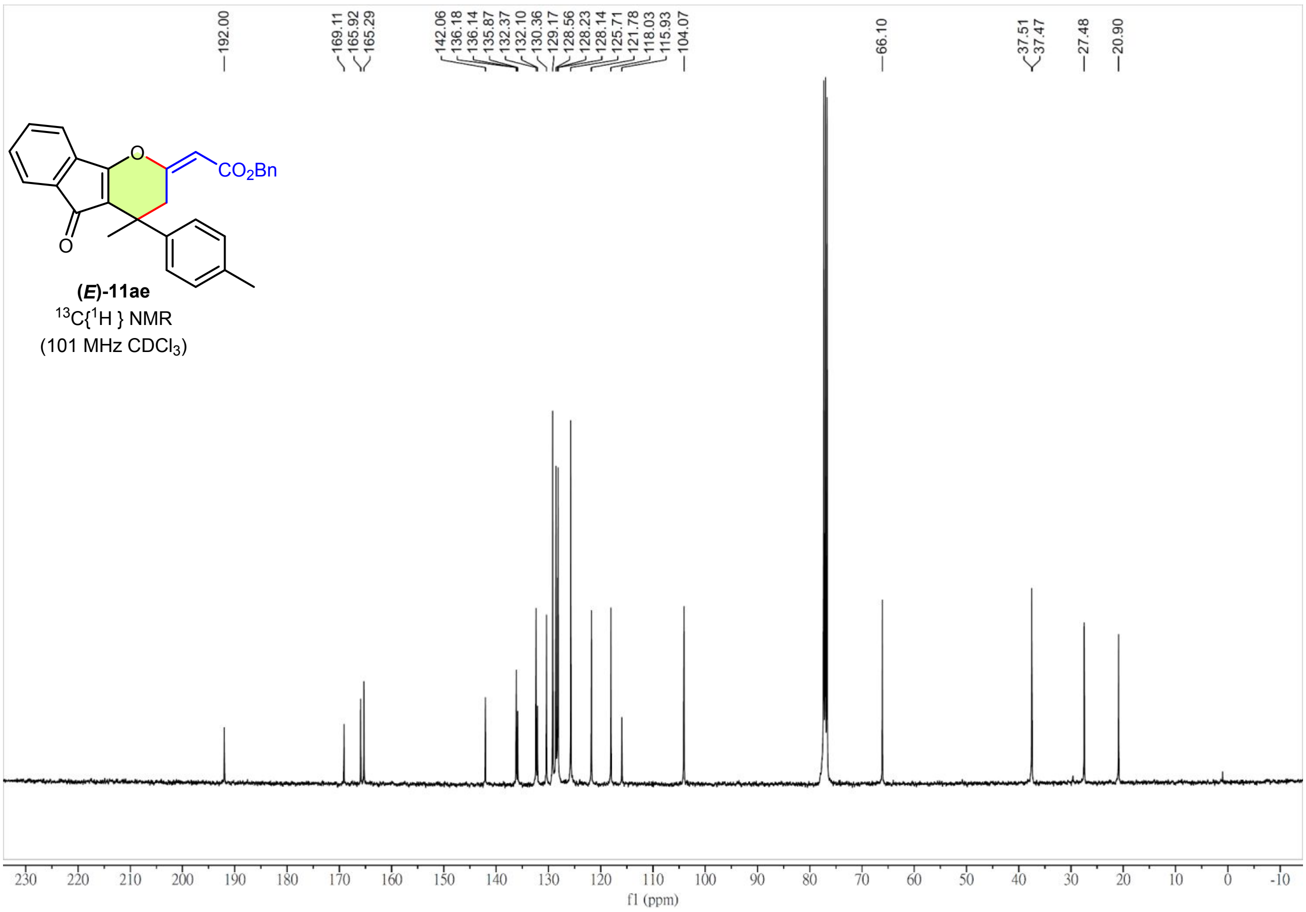

192.00
169.11 165.92 165.29
142.06 136.18 136.14 135.87 132.37 132.10 130.36 129.17 128.56 128.23 128.14 125.71 121.78 118.03 115.93
104.07
66.10
37.51 37.47
27.48
20.90
CO2Bn
O
O
(E)-11ae
13C{1H } NMR
(101 MHz CDCl3)
f1 (ppm)

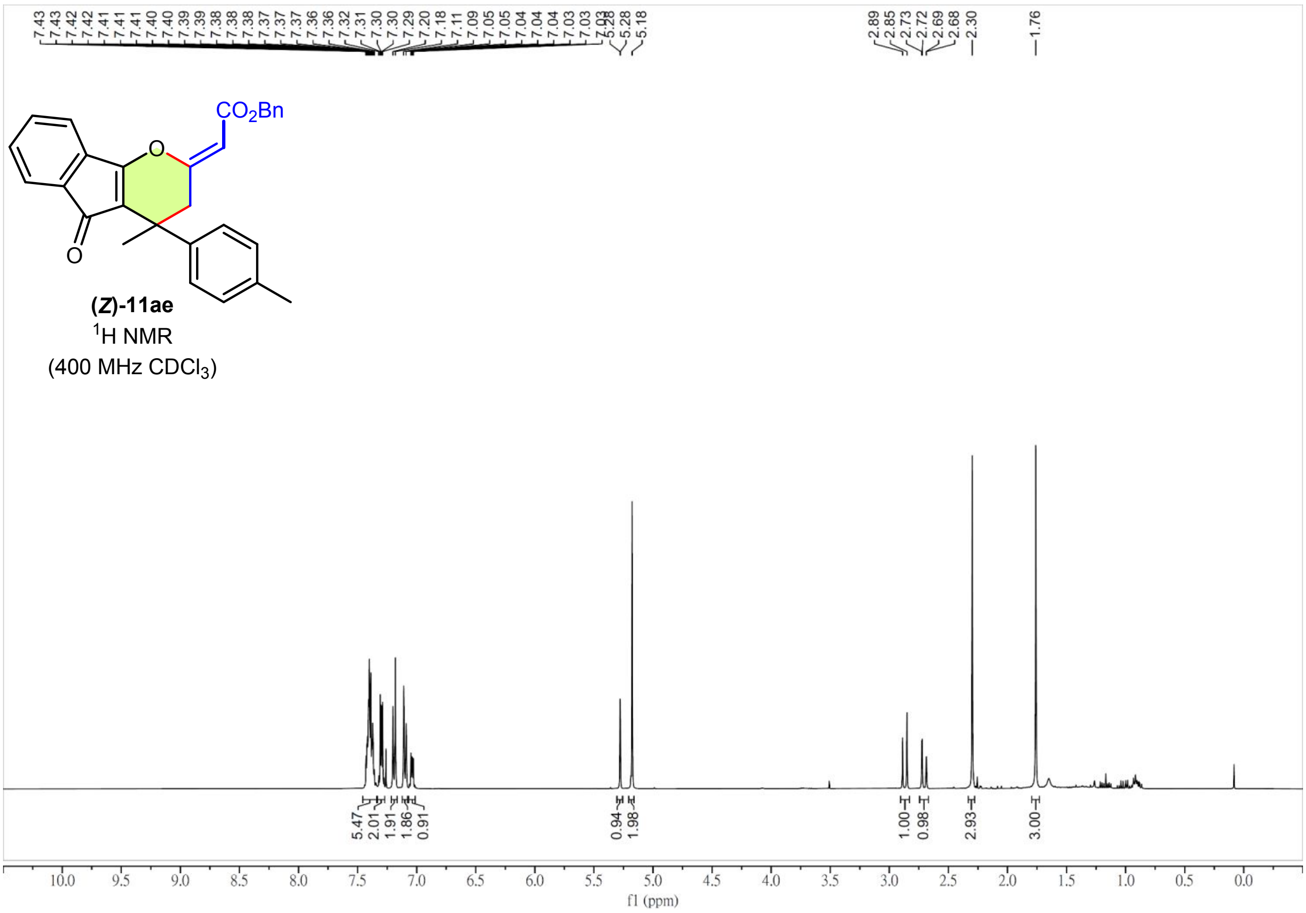

CO2Bn
O
O
(Z)-11ae
1H NMR
(400 MHz CDCl3)
f1 (ppm)

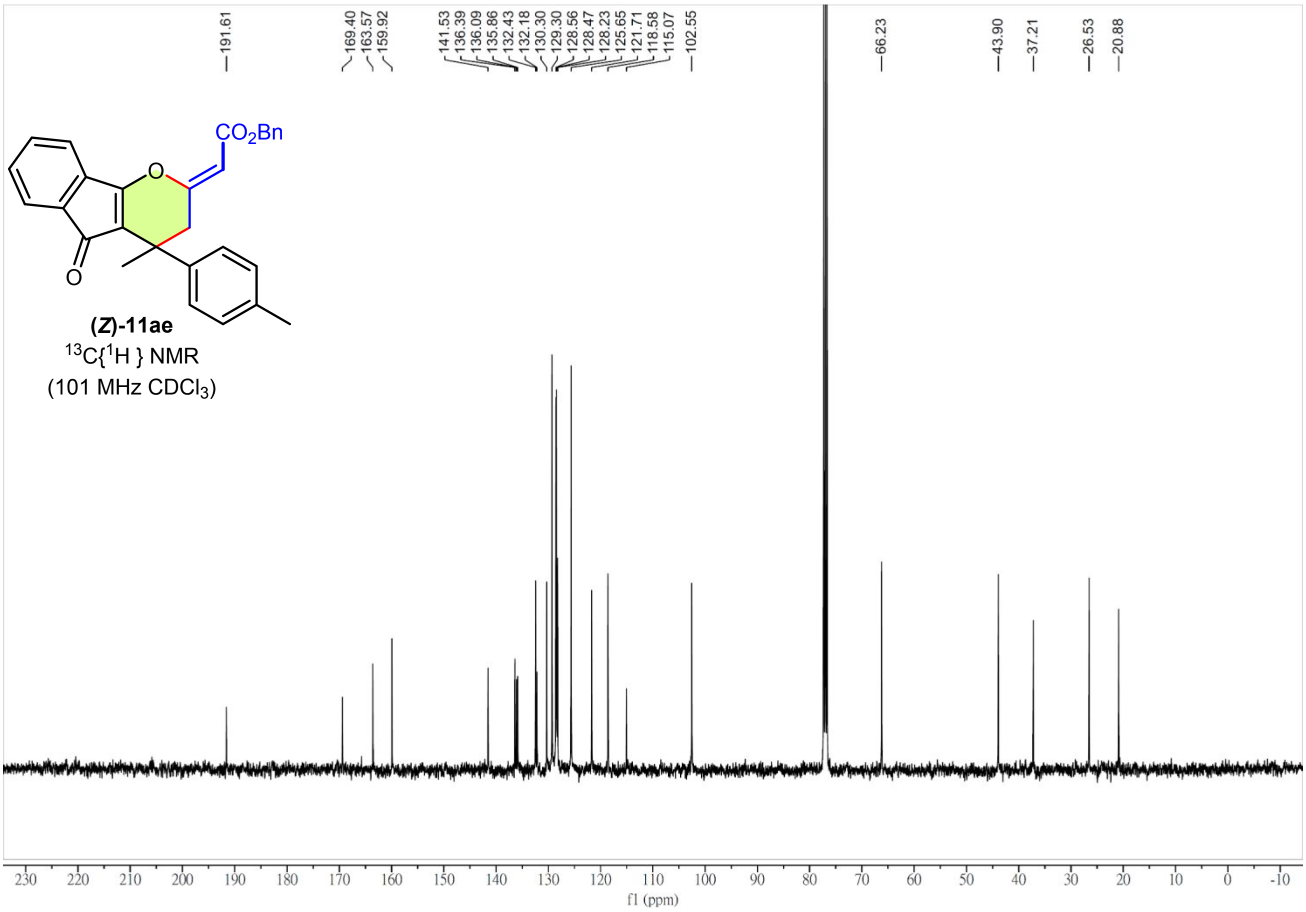

CO2Bn
O
O
(Z)-11ae
13C{1H } NMR
(101 MHz CDCl3)
f1 (ppm)

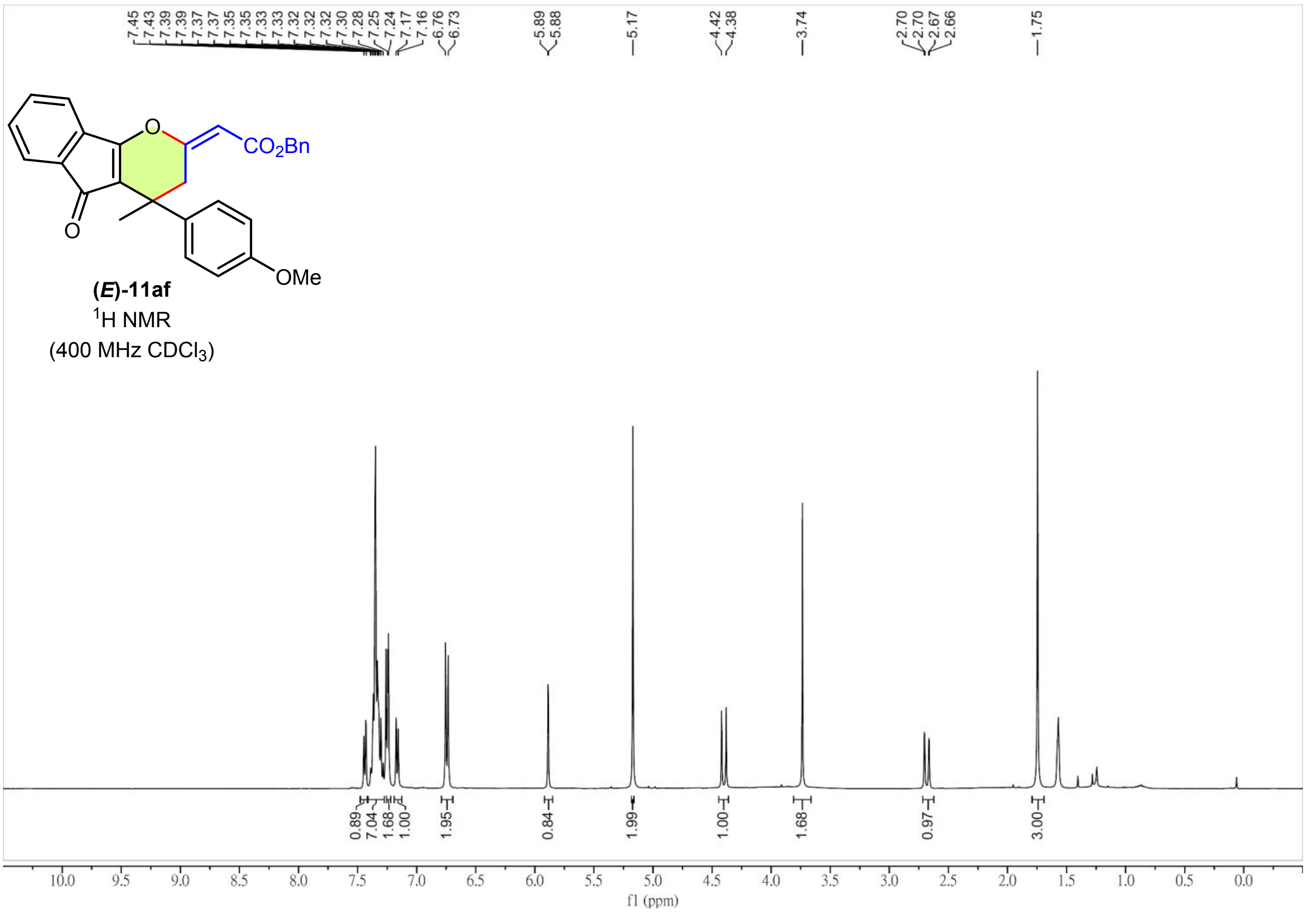

(E)-11af
1H NMR
(400 MHz CDCl3)

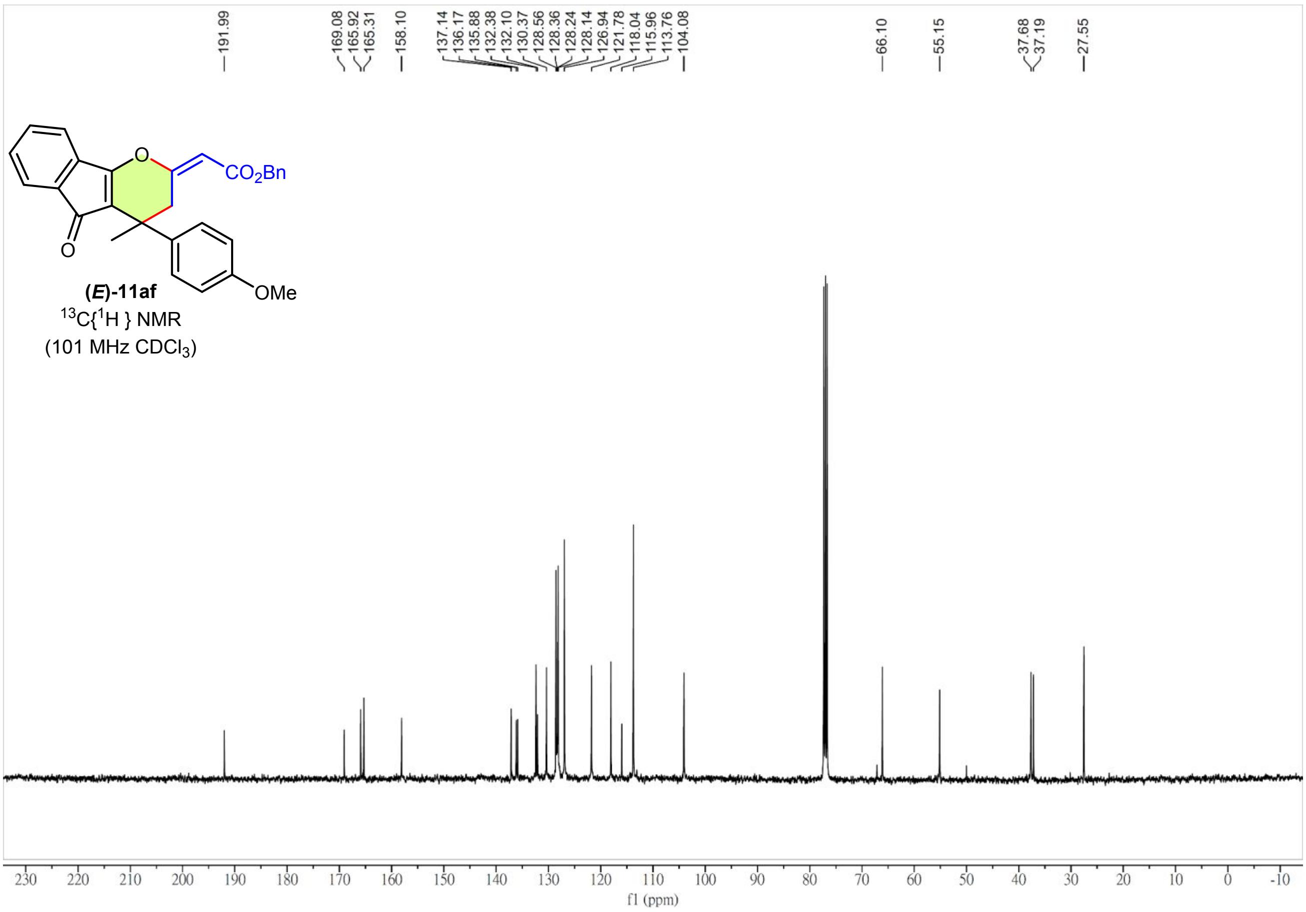

(E)-11af
13C{1H } NMR
(101 MHz CDCl3)

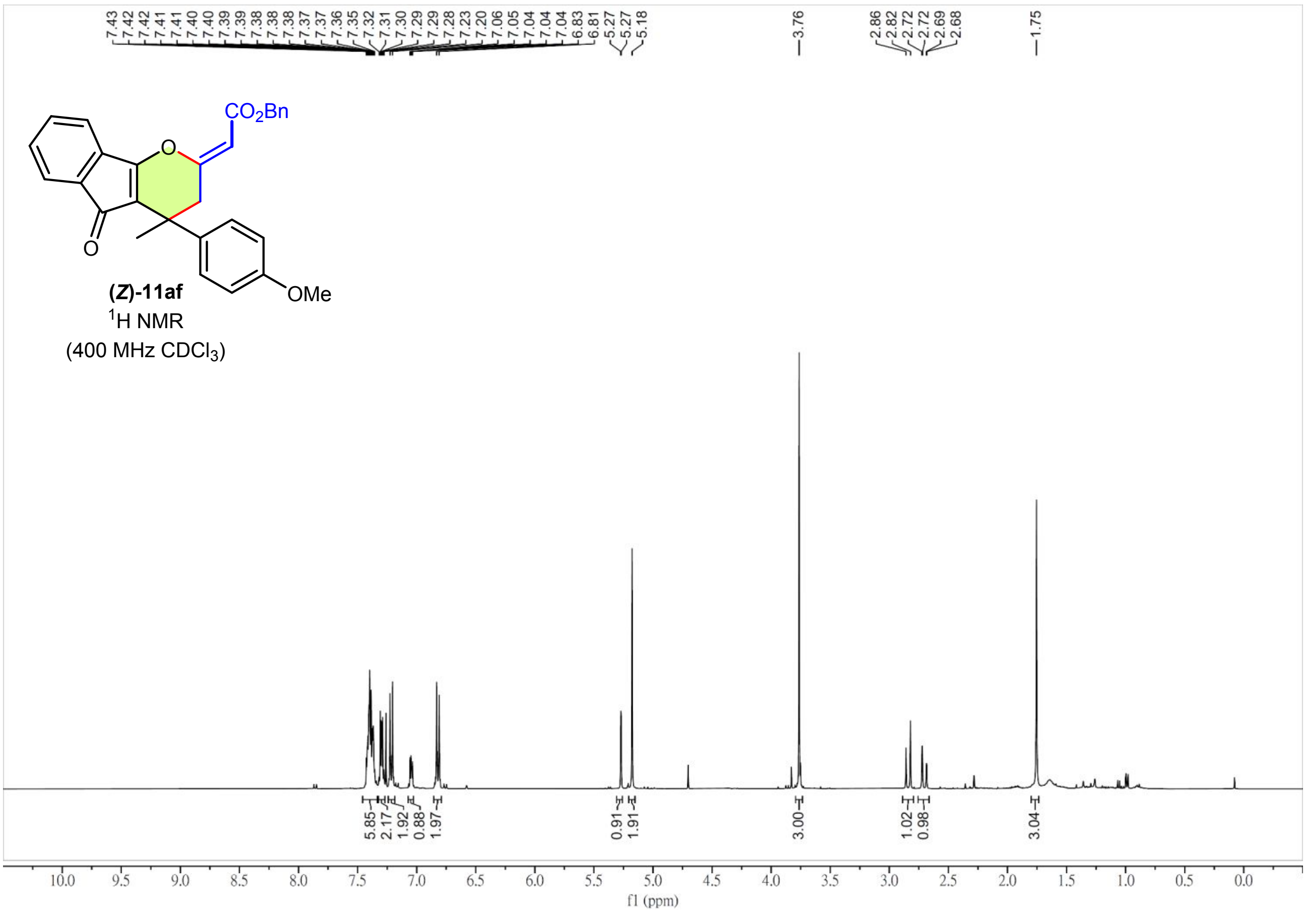
7.43 7.42 7.42 7.41 7.40 7.39 7.39 7.38 7.38 7.38 7.37 7.37 7.36 7.35 7.32 7.31 7.30 7.29 7.29 7.28 7.23 7.20 7.06 7.05 7.04 7.04 7.04 6.83 6.81 5.27 5.27 5.18
3.76
2.86 2.82 2.72 2.72 2.69 2.68
1.75
CO_2Bn
O
OMe
(Z)-11af
^1H NMR
(400 MHz $CDCl_3$)
5.85 2.17 1.92 0.88 1.97 0.91 1.91 3.00 1.02 0.98 3.04
f1 (ppm)

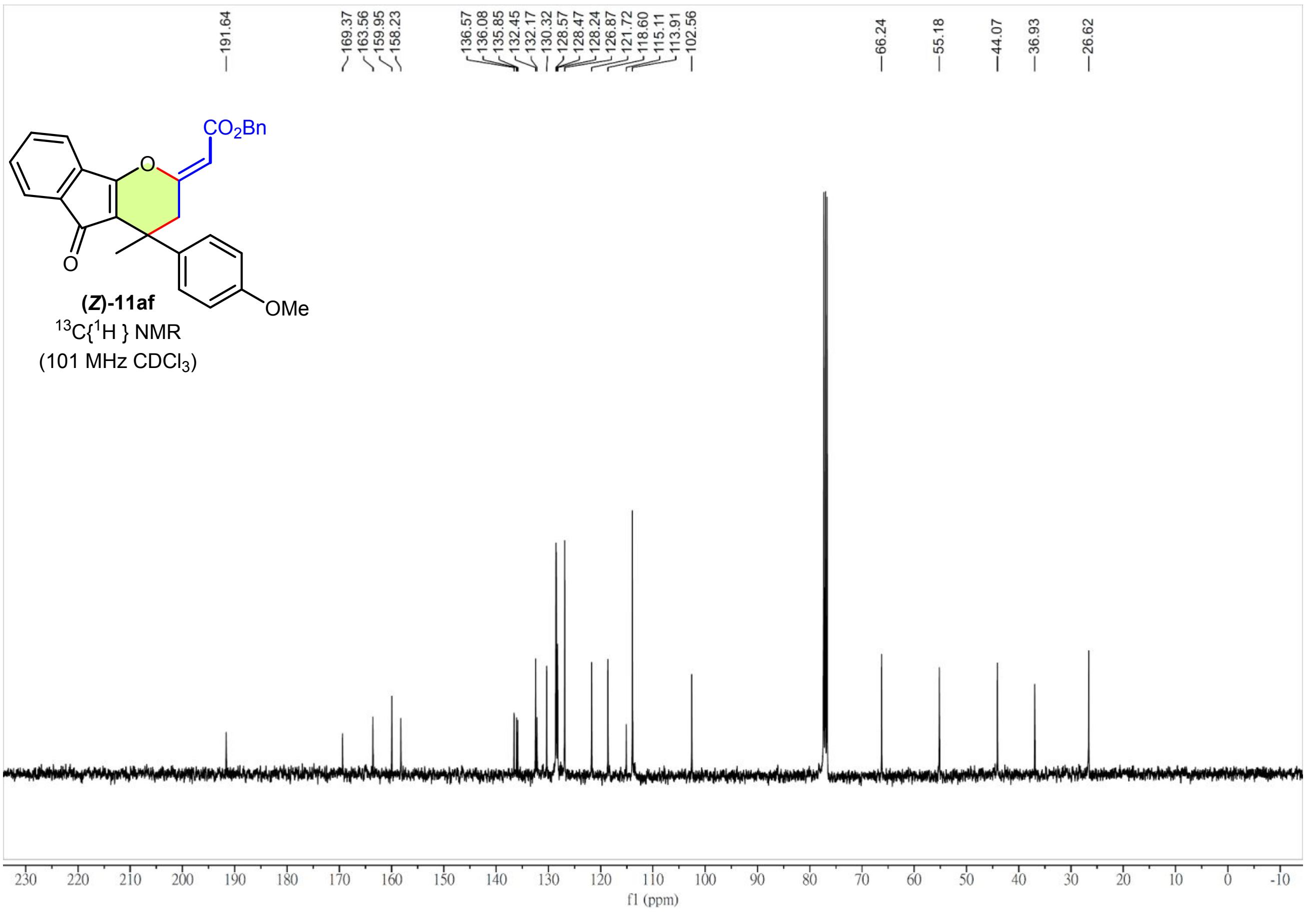
191.64
169.37 163.56 159.95 158.23
136.57 136.08 135.85 132.45 132.17 130.32 128.57 128.47 128.24 126.87 121.72 118.60 115.11 113.91 102.56
66.24
55.18
44.07
36.93
26.62
CO_2Bn
O
OMe
(Z)-11af
$^{13}C\{^1H\}$ NMR
(101 MHz $CDCl_3$)
f1 (ppm)

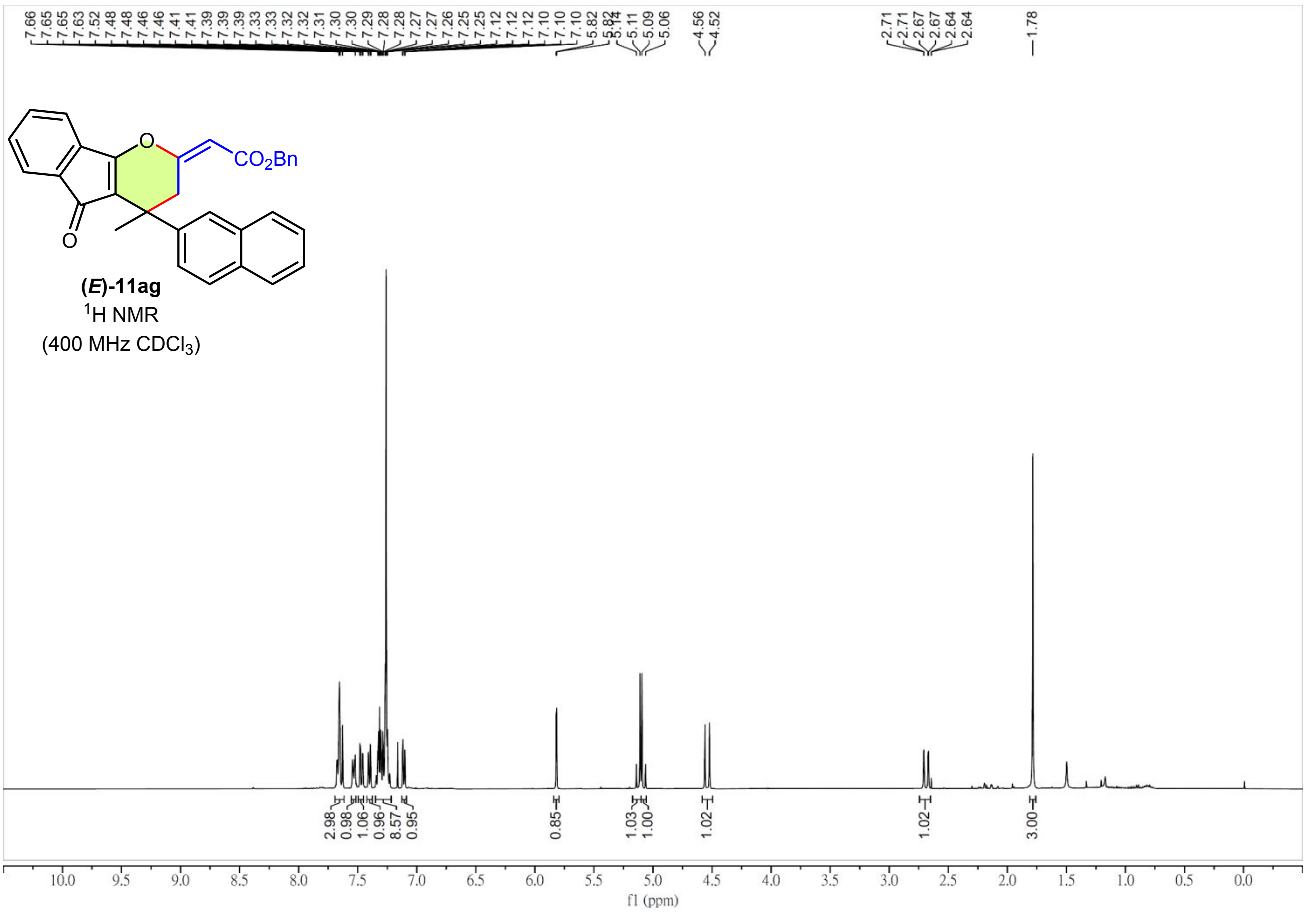

CO2Bn
O
(E)-11ag
1H NMR
(400 MHz CDCl3)
7.66 7.65 7.65 7.63 7.52 7.48 7.48 7.46 7.46 7.41 7.41 7.39 7.39 7.39 7.33 7.33 7.32 7.32 7.31 7.30 7.30 7.29 7.28 7.28 7.27 7.26 7.25 7.25 7.12 7.12 7.12 7.10 7.10 7.10 5.82 5.14 5.11 5.09 5.06 4.56 4.52 2.71 2.71 2.67 2.64 2.64 1.78
2.98 0.98 1.06 0.96 8.57 0.95 0.85 1.03 1.00 1.02 1.02 3.00
f1 (ppm)

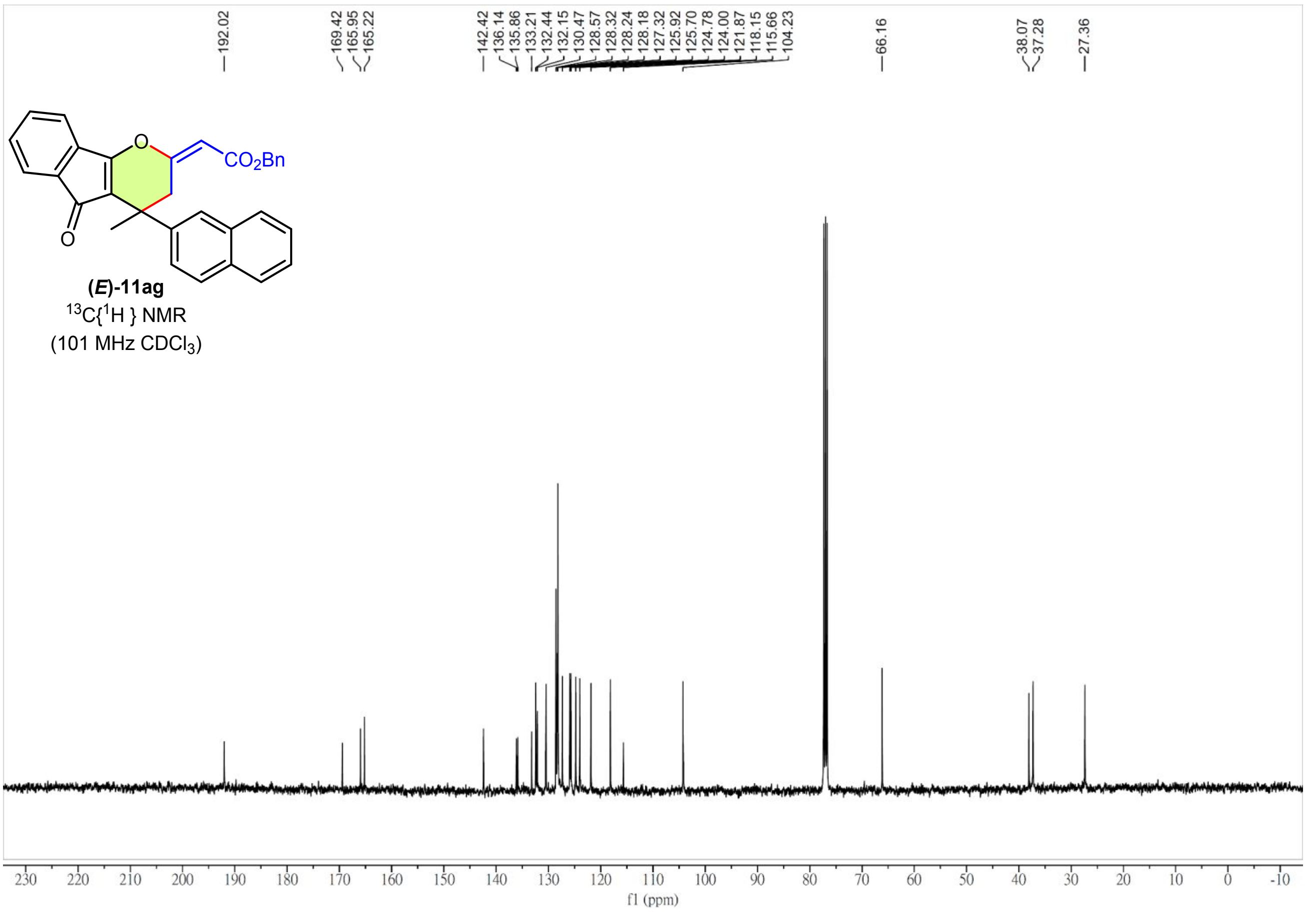

CO2Bn
O
(E)-11ag
13C{1H } NMR
(101 MHz CDCl3)
192.02 169.42 165.95 165.22 142.42 136.14 135.86 133.21 132.44 132.15 130.47 128.57 128.32 128.24 128.18 127.32 125.92 125.70 124.78 124.00 121.87 118.15 115.66 104.23 66.16 38.07 37.28 27.36
f1 (ppm)

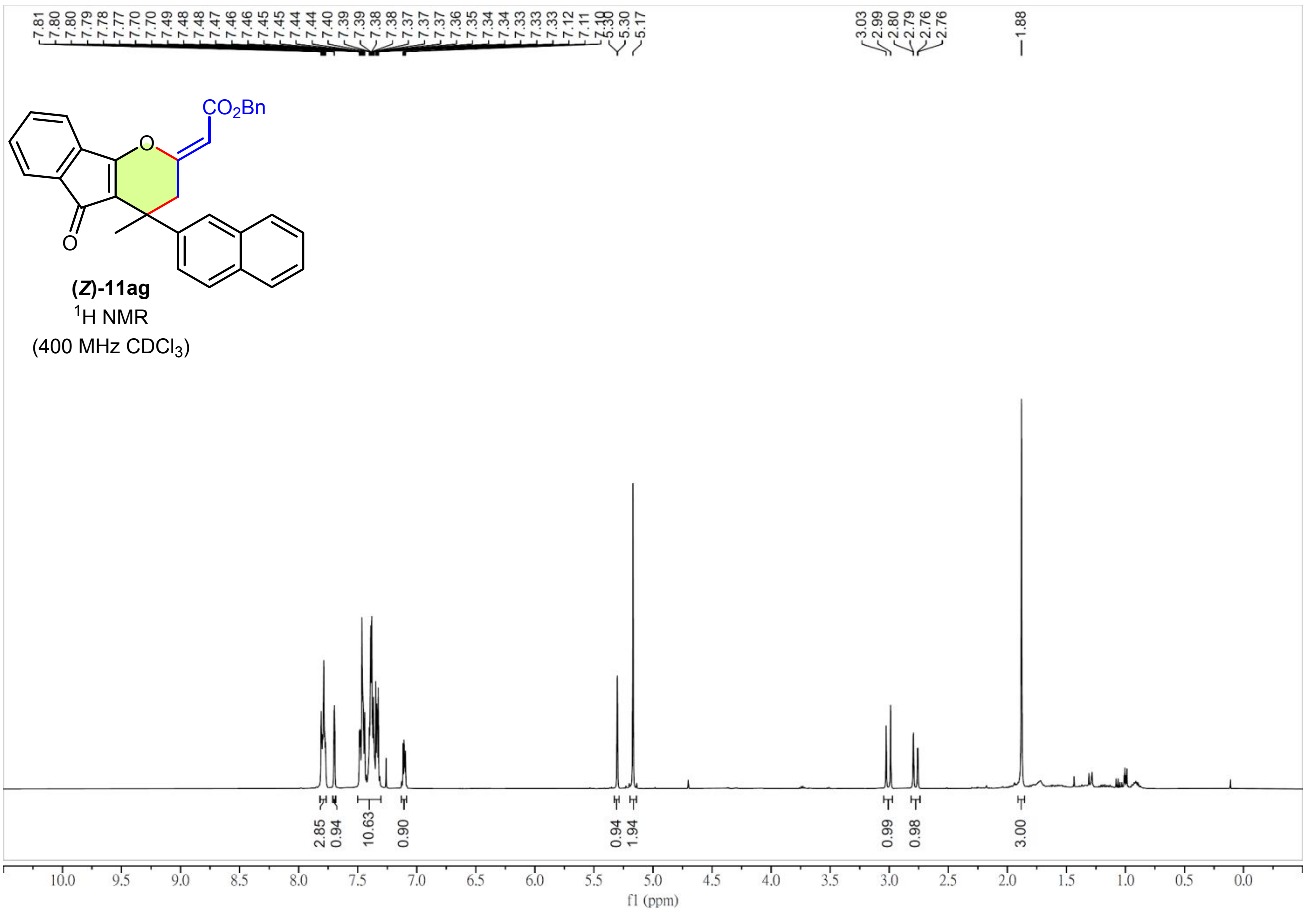
(Z)-11ag
^{1}H NMR
(400 MHz CDCl$_3$)
f1 (ppm)

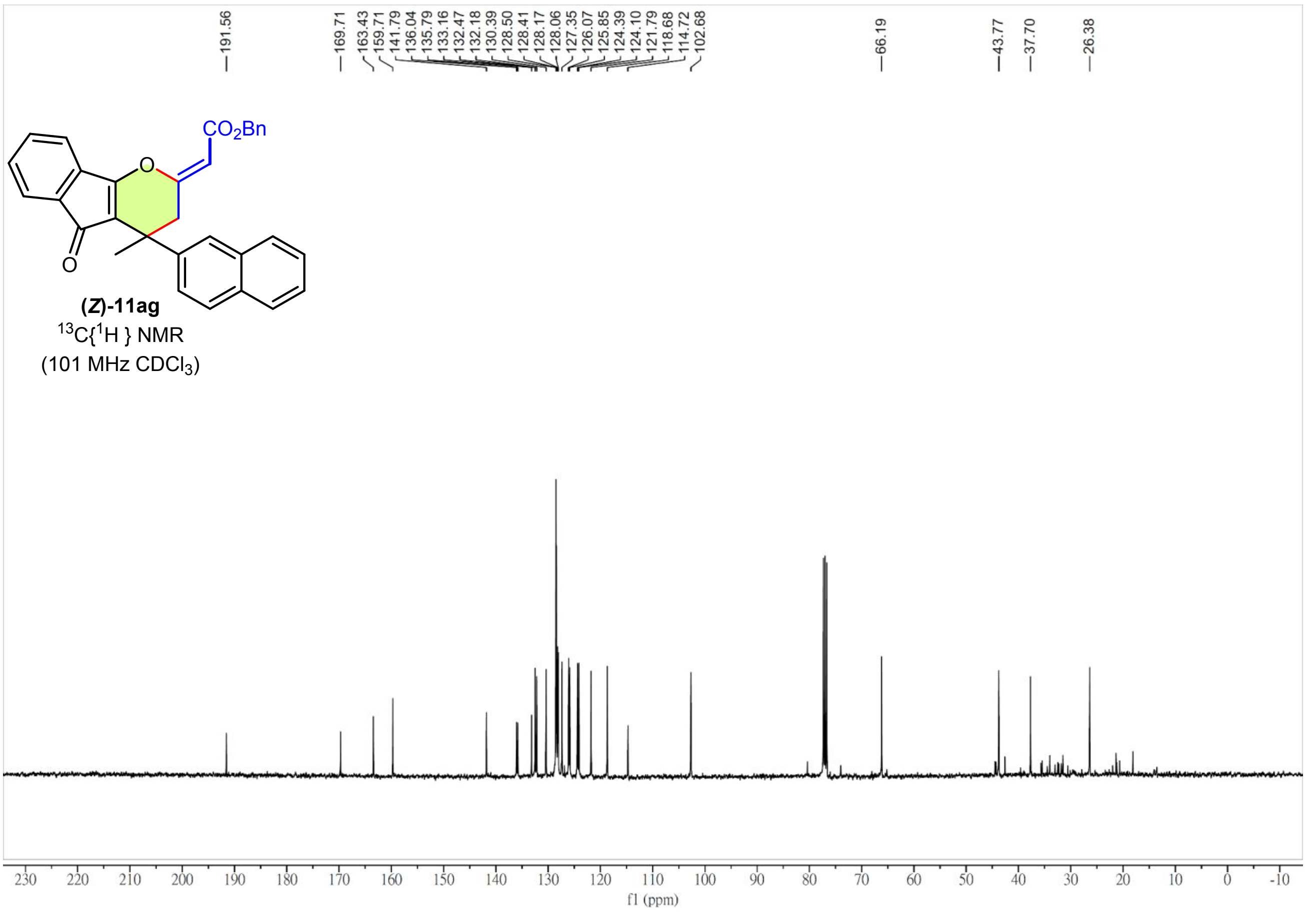
(Z)-11ag
^{13}C{^{1}H } NMR
(101 MHz CDCl$_3$)
f1 (ppm)

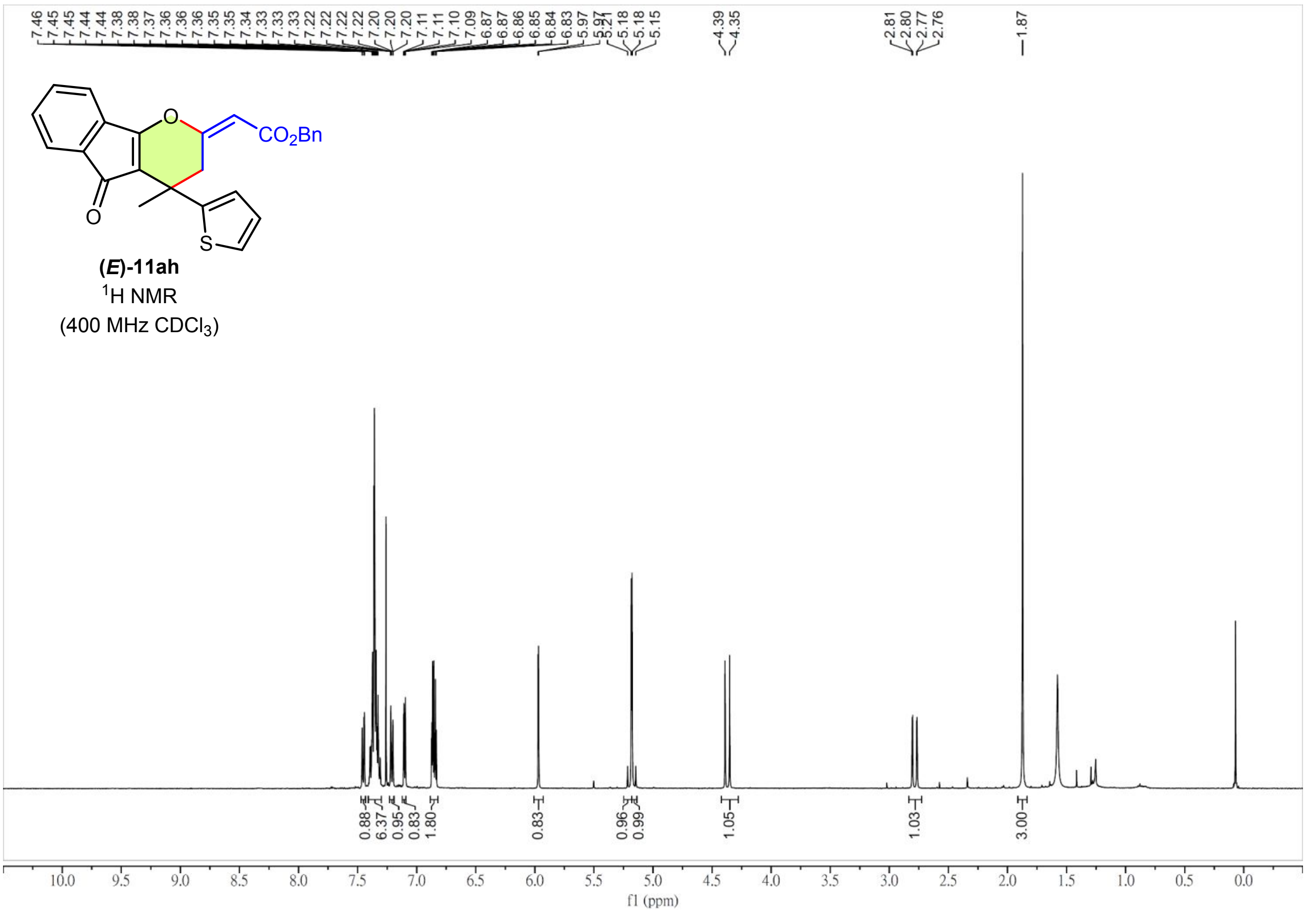

(*E*)-11ah
^{1}H NMR
(400 MHz $CDCl_3$)

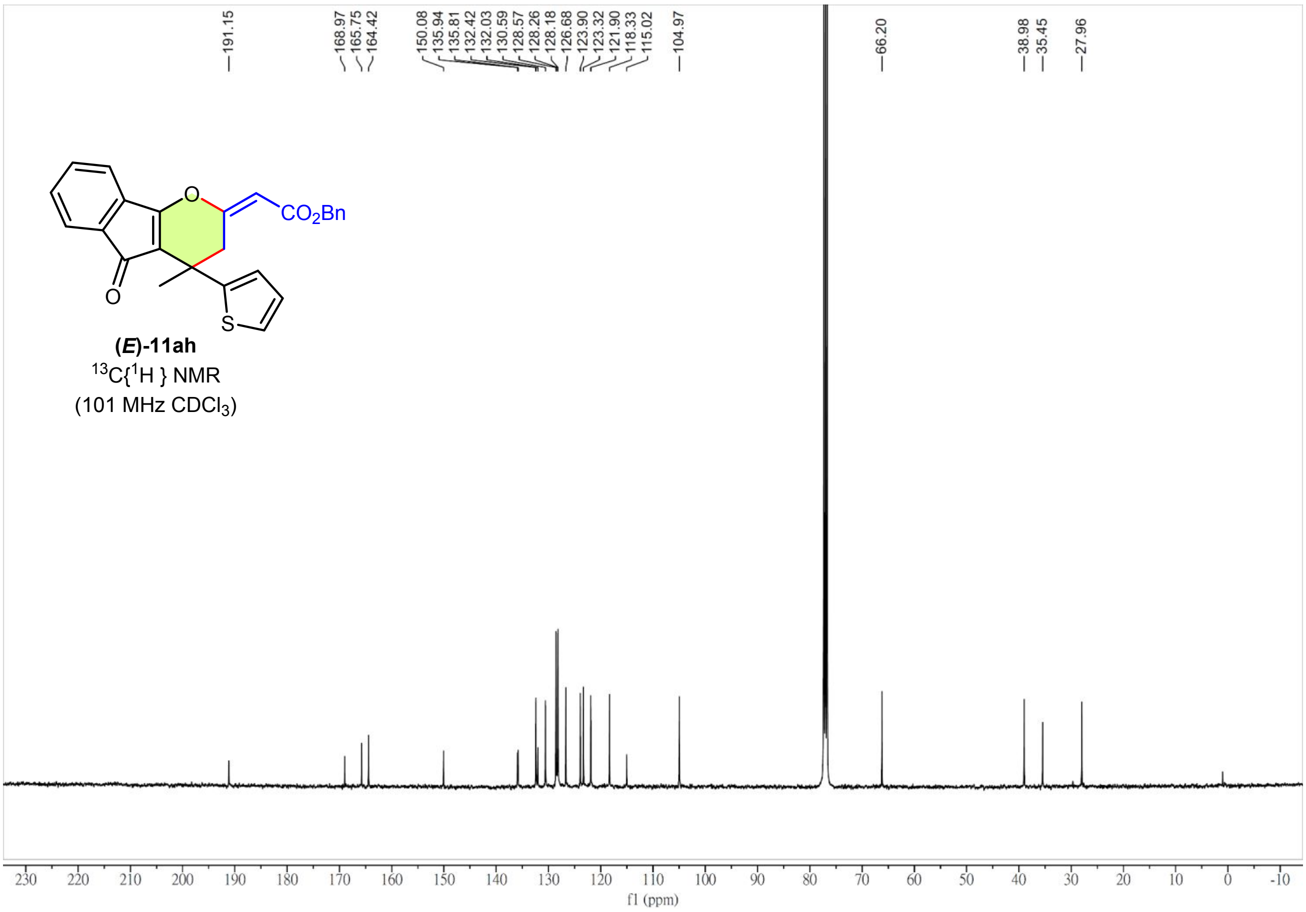

(*E*)-11ah
$^{13}C\{^1H\}$ NMR
(101 MHz $CDCl_3$)

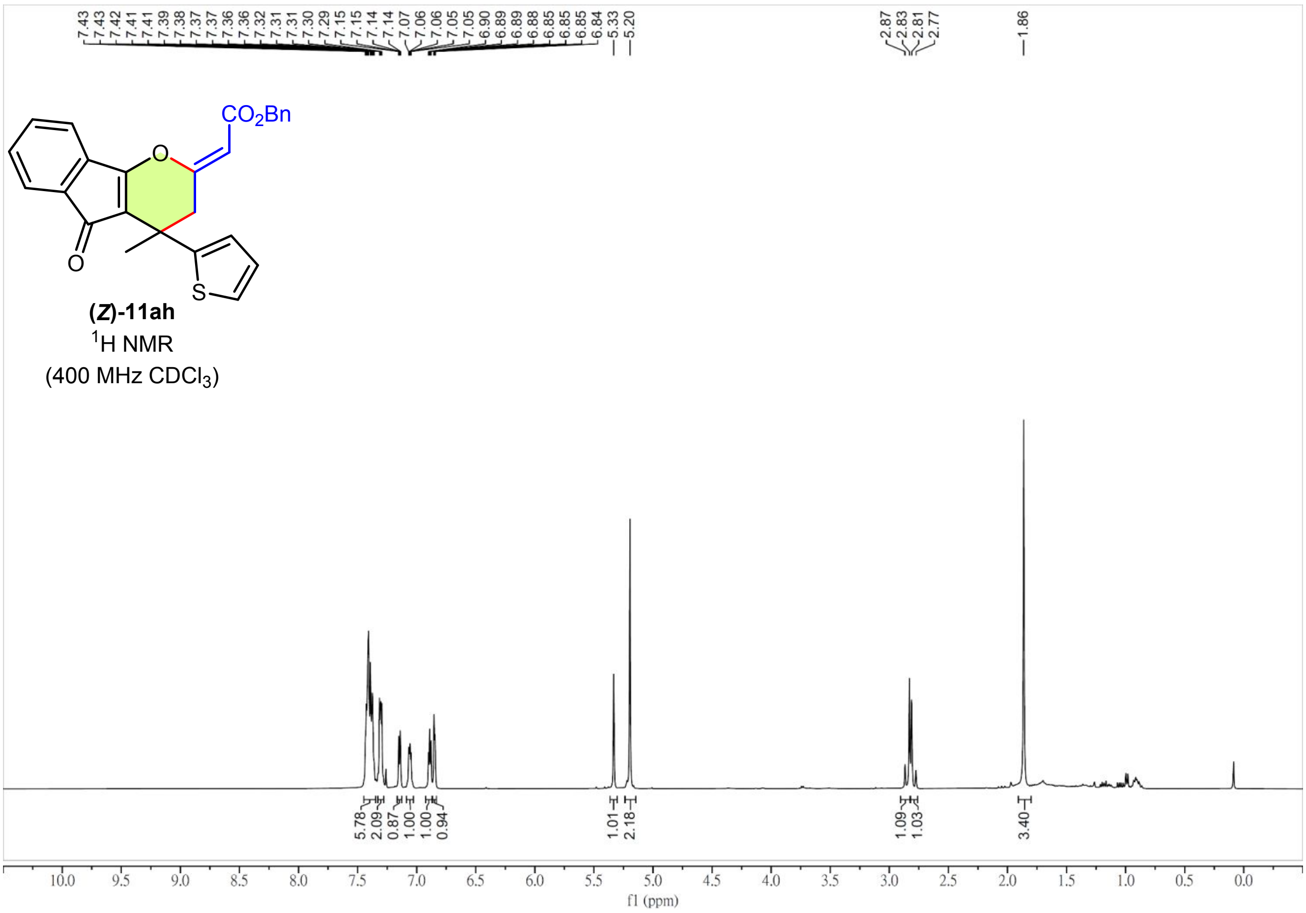
7.43 7.43 7.42 7.41 7.41 7.39 7.38 7.37 7.37 7.36 7.36 7.36 7.32 7.31 7.30 7.29 7.15 7.15 7.14 7.14 7.07 7.06 7.06 7.05 7.05 6.90 6.89 6.89 6.88 6.85 6.85 6.85 6.84 5.33 5.20
2.87 2.83 2.81 2.77
1.86
CO2Bn
O
S
O
(Z)-11ah
1H NMR
(400 MHz CDCl3)
5.78 2.09 0.87 1.00 1.00 0.94
1.01 2.18
1.09 1.03
3.40
10.0 9.5 9.0 8.5 8.0 7.5 7.0 6.5 6.0 5.5 5.0 4.5 4.0 3.5 3.0 2.5 2.0 1.5 1.0 0.5 0.0
f1 (ppm)

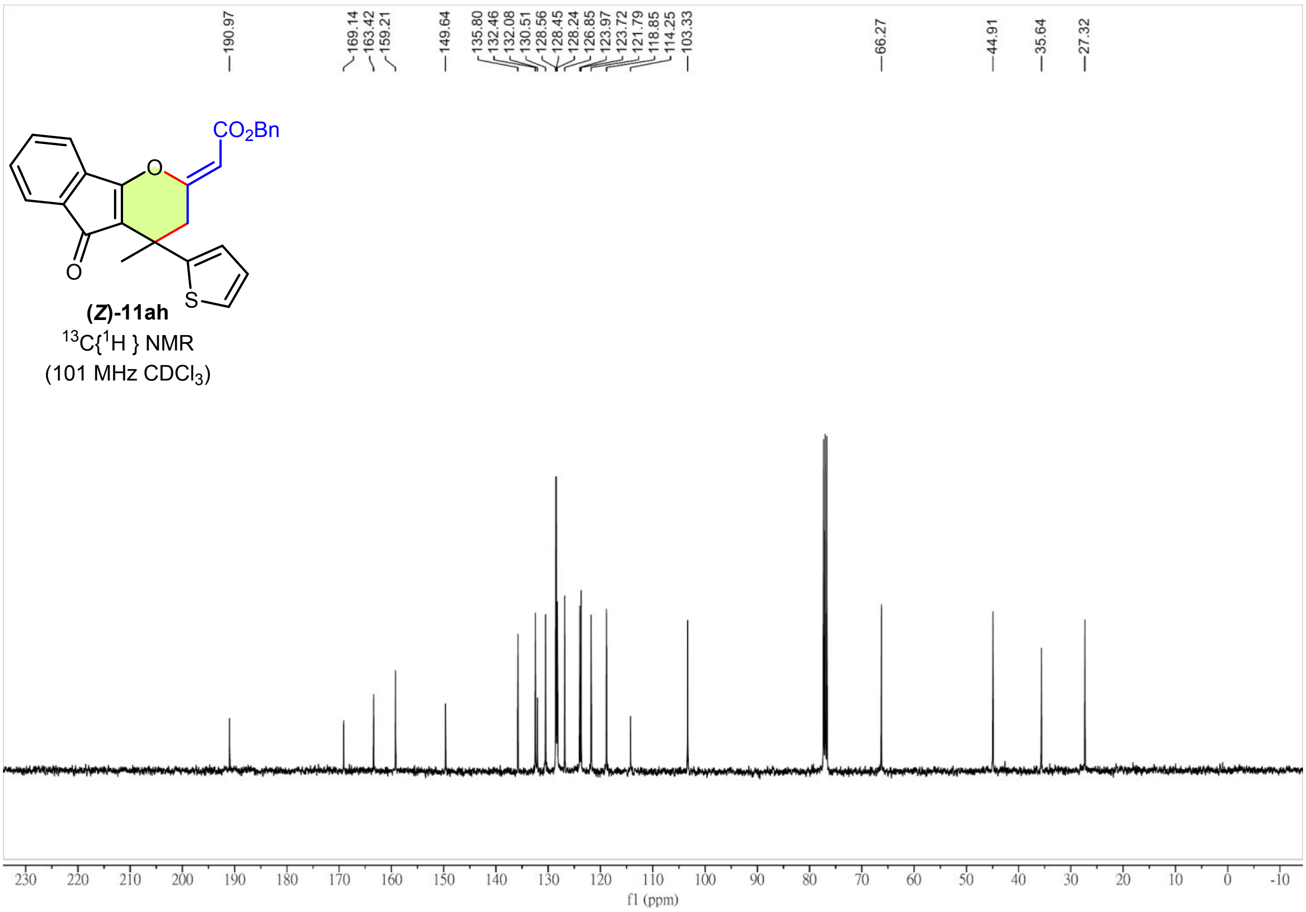
190.97
169.14 163.42 159.21
149.64
135.80 132.46 132.08 130.51 128.56 128.45 128.24 126.85 123.97 123.72 121.79 118.85 114.25 103.33
66.27
44.91
35.64
27.32
CO2Bn
O
S
O
(Z)-11ah
13C{1H } NMR
(101 MHz CDCl3)
230 220 210 200 190 180 170 160 150 140 130 120 110 100 90 80 70 60 50 40 30 20 10 0 -10
f1 (ppm)

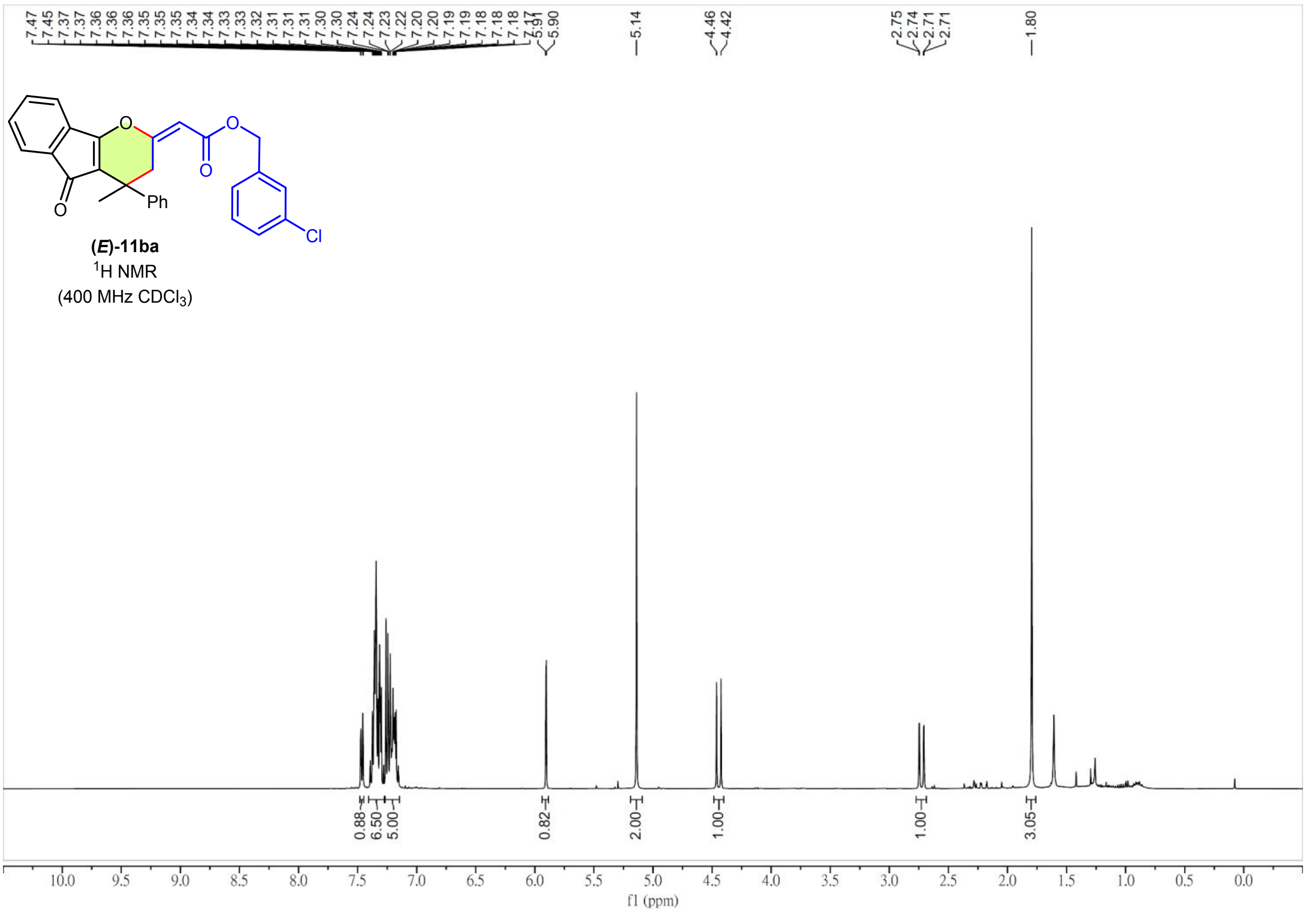
(E)-11ba
^{1}H NMR
(400 MHz CDCl$_3$)
7.47 7.45 7.37 7.37 7.36 7.36 7.35 7.35 7.34 7.34 7.33 7.33 7.32 7.31 7.31 7.30 7.24 7.24 7.23 7.22 7.20 7.20 7.19 7.19 7.18 7.18 7.17 5.91 5.90
5.14
4.46 4.42
2.75 2.74 2.71 2.71
1.80
0.88 6.50 5.00 0.82 2.00 1.00 1.00 3.05
f1 (ppm)

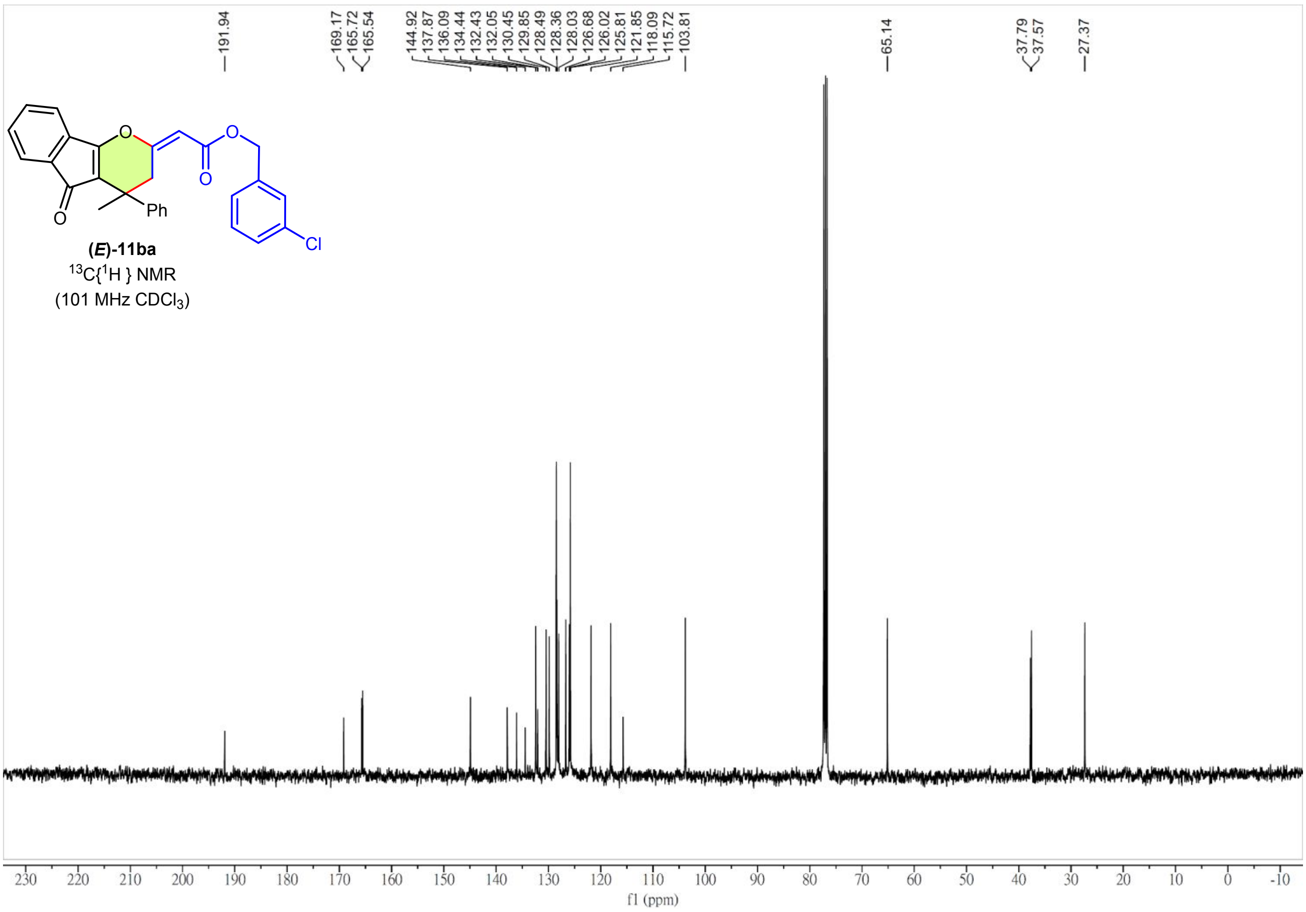
(E)-11ba
^{13}C{^{1}H } NMR
(101 MHz CDCl$_3$)
191.94
169.17 165.72 165.54
144.92 137.87 136.09 134.44 132.43 132.05 130.45 129.85 128.49 128.36 128.03 126.68 126.02 125.81 121.85 118.09 115.72 103.81
65.14
37.79 37.57
27.37
f1 (ppm)

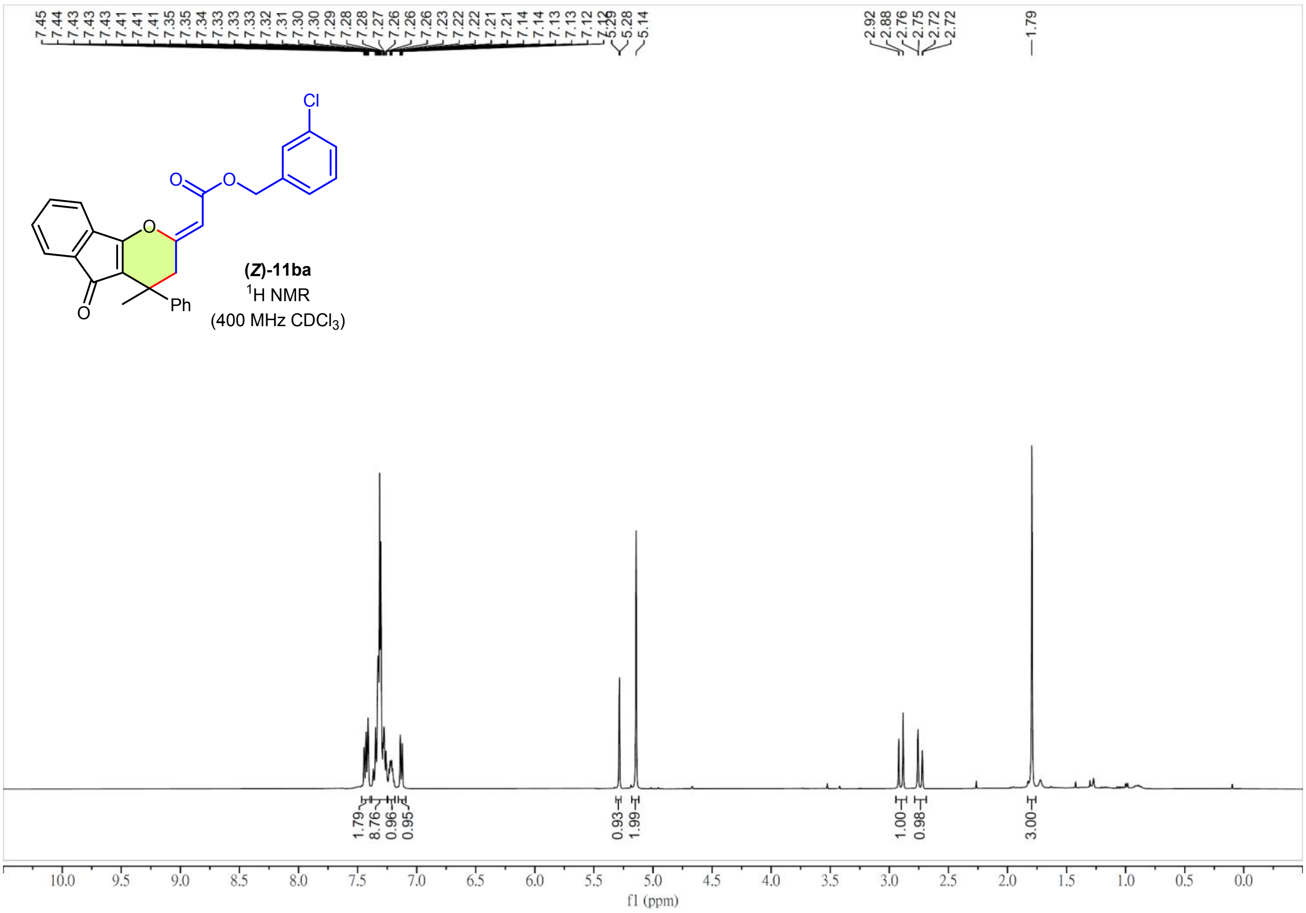

(*Z*)-11ba
^{1}H NMR
(400 MHz $CDCl_3$)

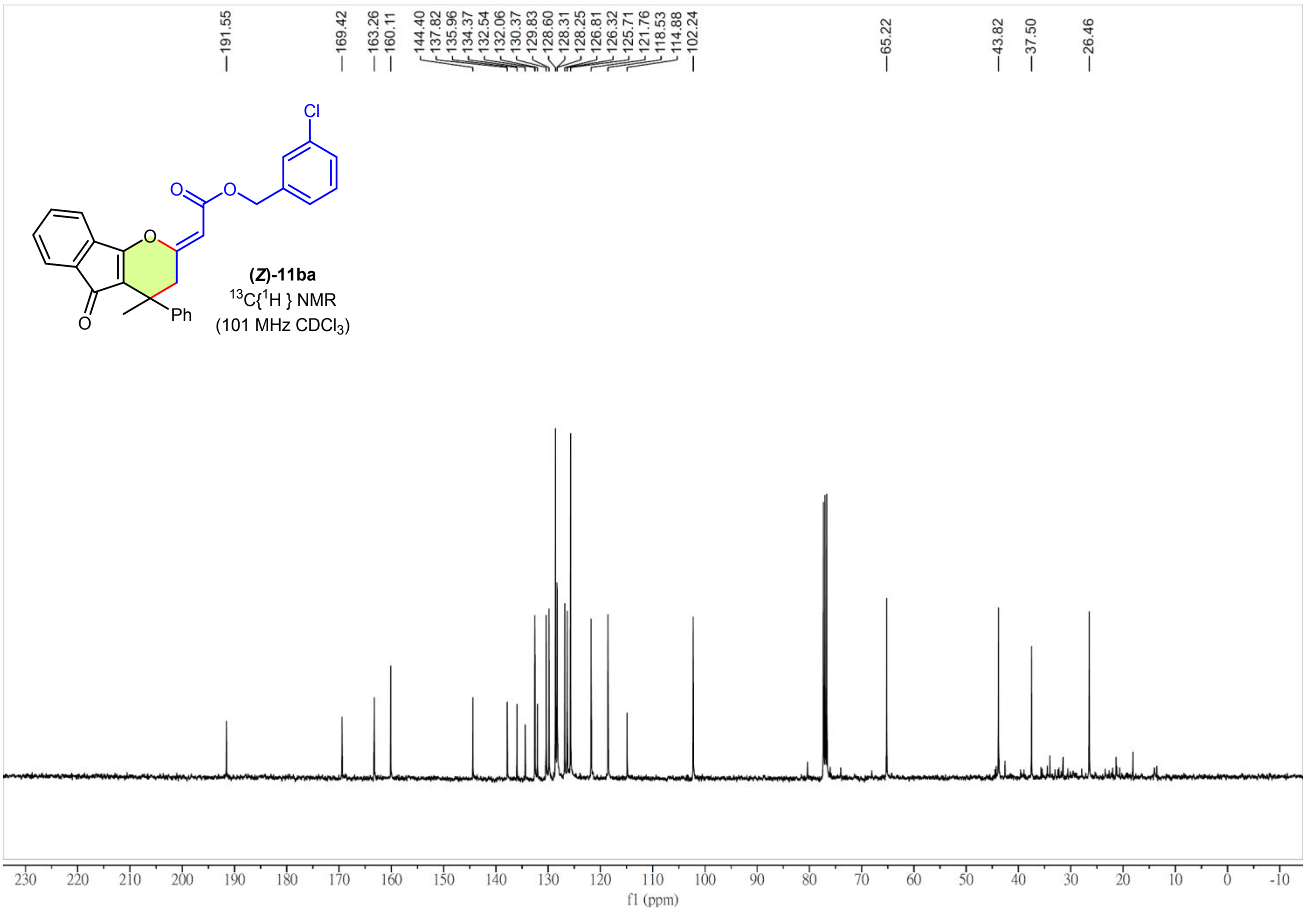

(*Z*)-11ba
$^{13}C\{^{1}H\}$ NMR
(101 MHz $CDCl_3$)

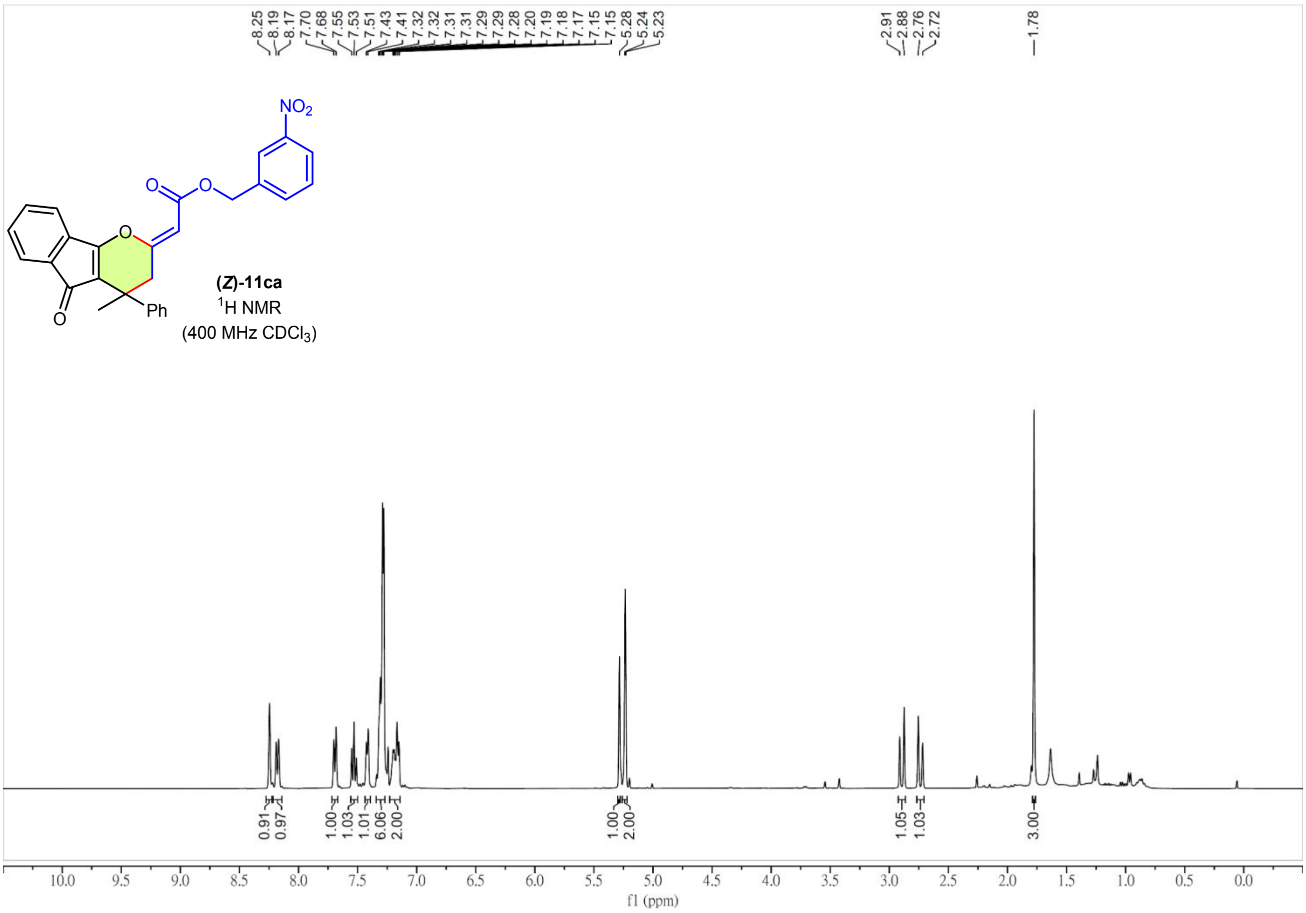
8.25 8.19 8.17 7.70 7.68 7.55 7.53 7.51 7.43 7.41 7.32 7.31 7.31 7.29 7.29 7.28 7.20 7.19 7.18 7.17 7.15 7.15 5.28 5.24 5.23
2.91 2.88 2.76 2.72
1.78
NO2
O
O
O
O
Ph
(Z)-11ca
1H NMR
(400 MHz CDCl3)
0.91 0.97 1.00 1.03 1.01 6.06 2.00 1.00 2.00 1.05 1.03 3.00
10.0 9.5 9.0 8.5 8.0 7.5 7.0 6.5 6.0 5.5 5.0 4.5 4.0 3.5 3.0 2.5 2.0 1.5 1.0 0.5 0.0
f1 (ppm)

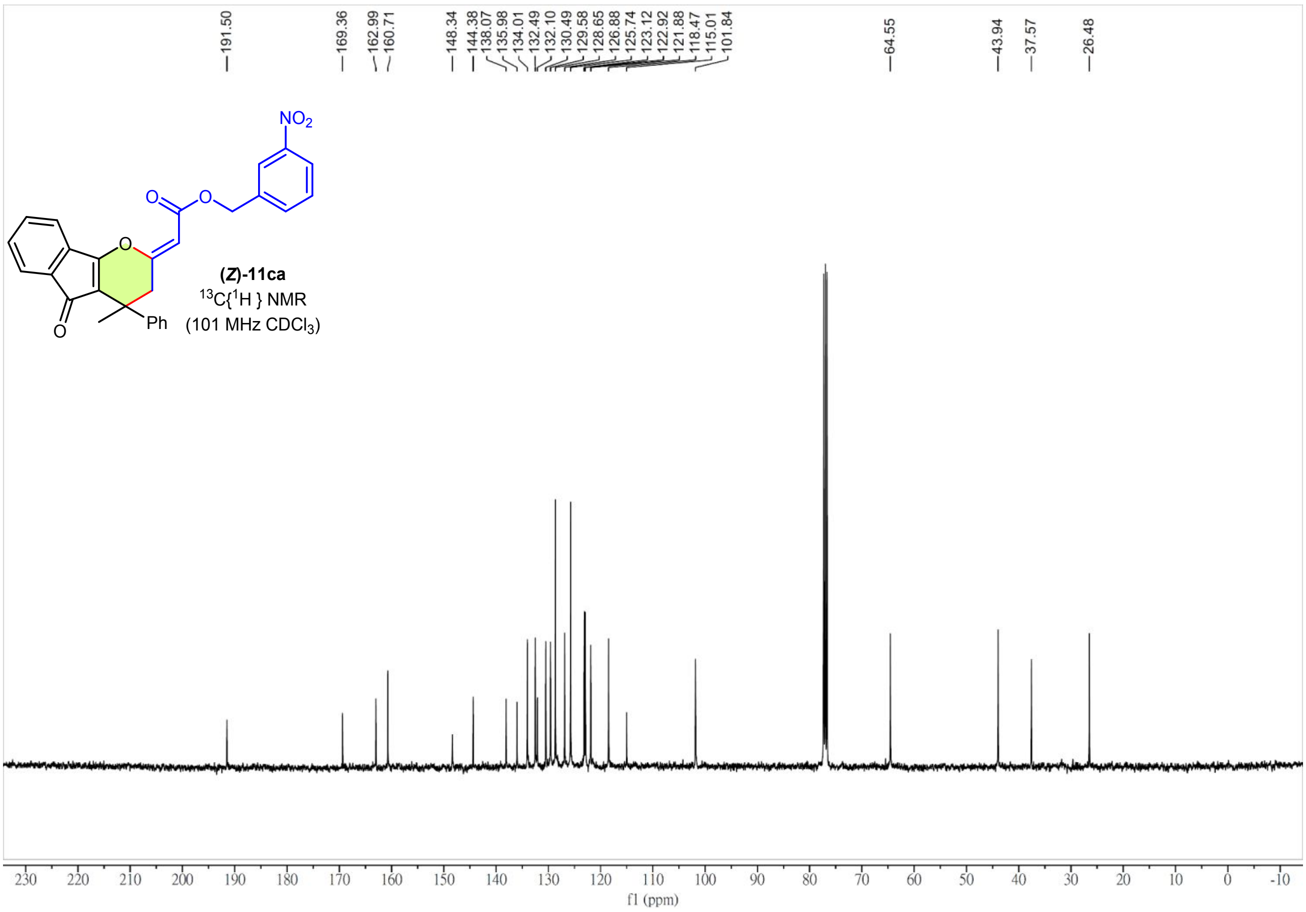
191.50
169.36 162.99 160.71
148.34 144.38 138.07 135.98 134.01 132.49 132.10 130.49 129.58 128.65 126.88 125.74 123.12 122.92 121.88 118.47 115.01 101.84
64.55
43.94 37.57 26.48
NO2
O
O
O
O
Ph
(Z)-11ca
13C{1H } NMR
(101 MHz CDCl3)
230 220 210 200 190 180 170 160 150 140 130 120 110 100 90 80 70 60 50 40 30 20 10 0 -10
f1 (ppm)

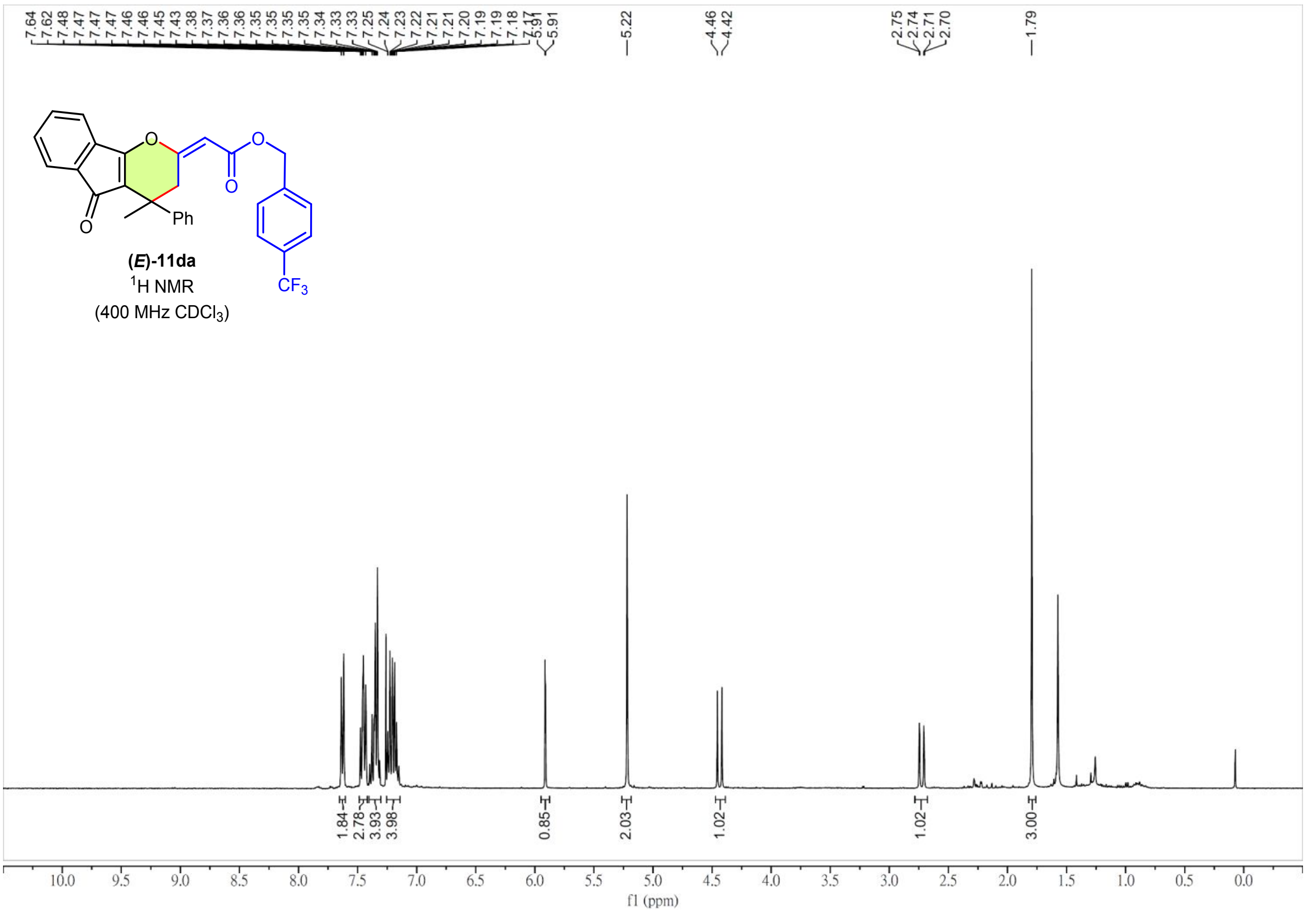
(E)-11da
1H NMR
(400 MHz CDCl3)
f1 (ppm)

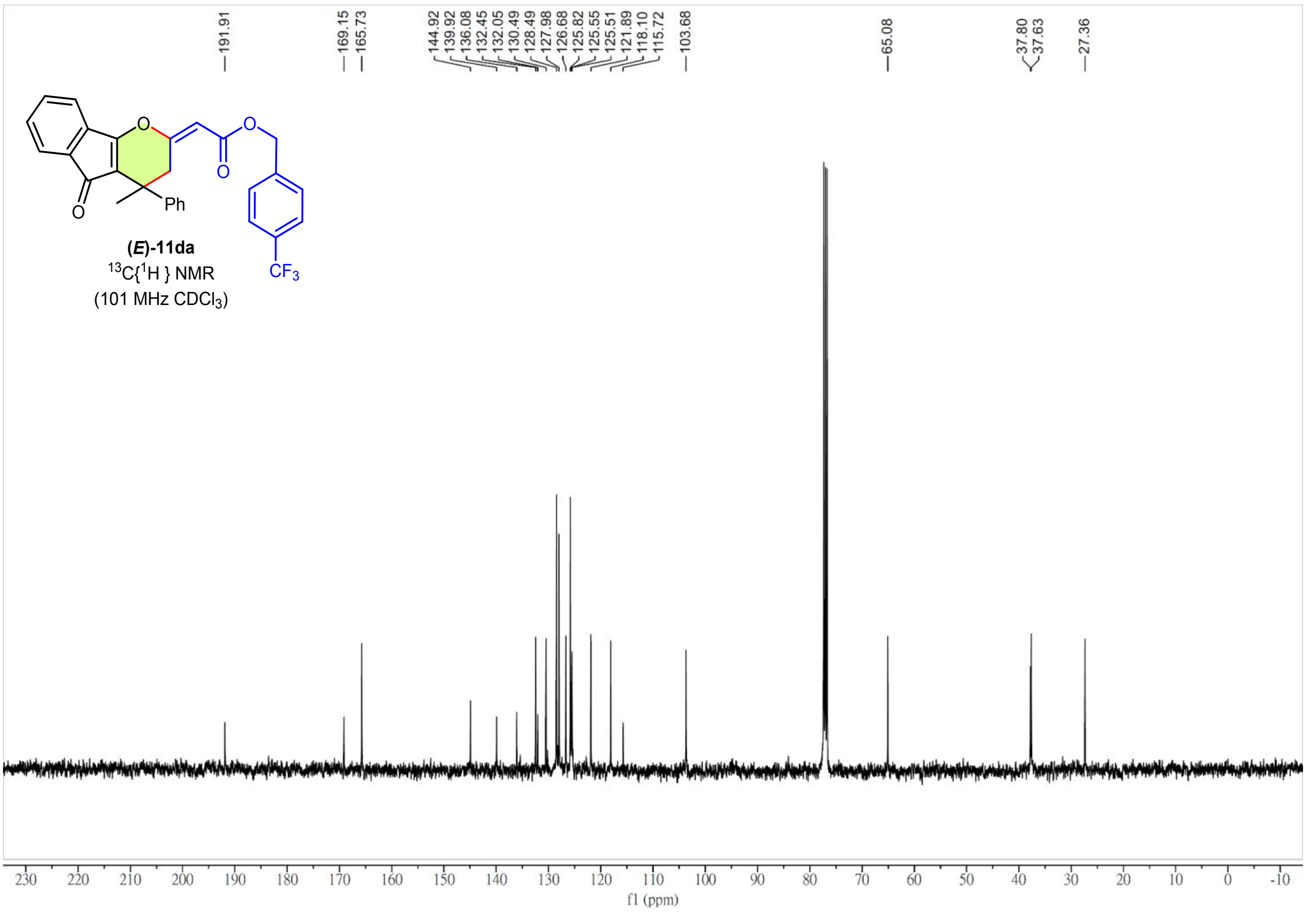
(E)-11da
13C{1H } NMR
(101 MHz CDCl3)
f1 (ppm)

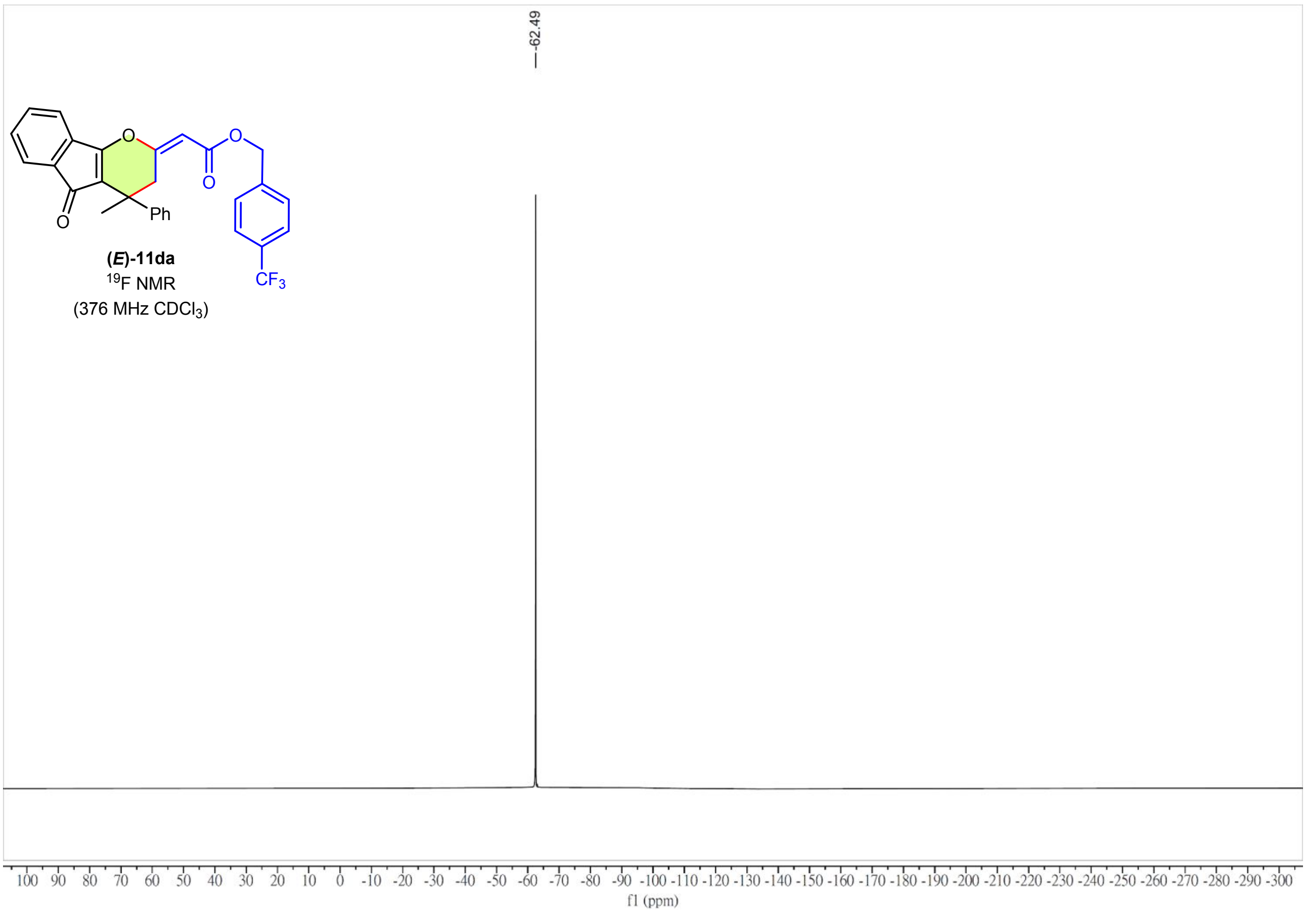

-62.49
O
O
O
O
Ph
CF3
(E)-11da
19F NMR
(376 MHz CDCl3)
100 90 80 70 60 50 40 30 20 10 0 -10 -20 -30 -40 -50 -60 -70 -80 -90 -100 -110 -120 -130 -140 -150 -160 -170 -180 -190 -200 -210 -220 -230 -240 -250 -260 -270 -280 -290 -300
f1 (ppm)

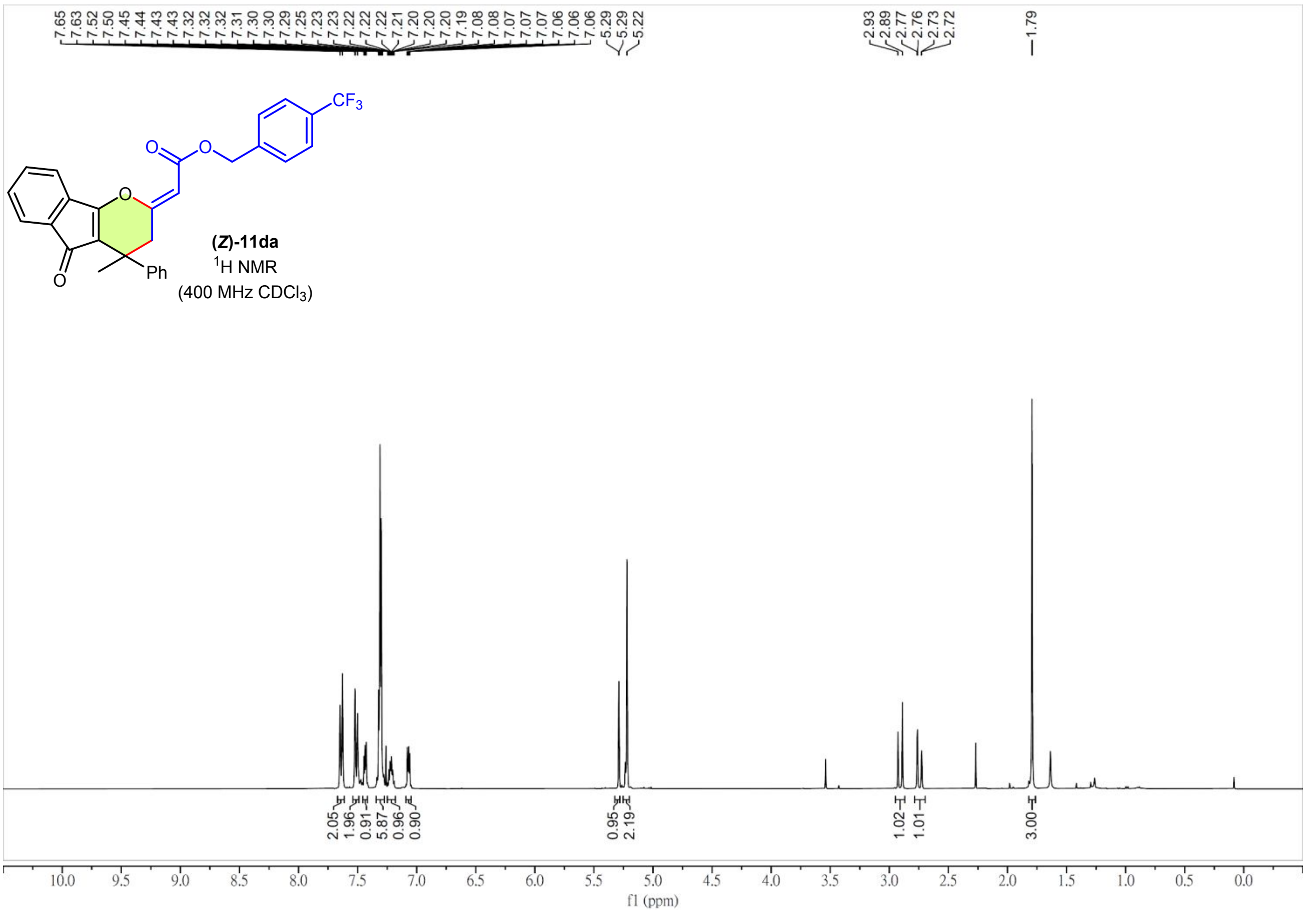
7.65 7.63 7.52 7.50 7.45 7.44 7.43 7.43 7.32 7.32 7.32 7.31 7.30 7.29 7.25 7.23 7.23 7.22 7.22 7.22 7.21 7.20 7.20 7.20 7.19 7.08 7.08 7.07 7.07 7.07 7.06 7.06 7.06 5.29 5.29 5.22
2.93 2.89 2.77 2.76 2.73 2.72
1.79
CF3
O
O
O
O
Ph
(Z)-11da
1H NMR
(400 MHz CDCl3)
2.05 1.96 0.91 5.87 0.96 0.90
0.95 2.19
1.02 1.01
3.00
10.0 9.5 9.0 8.5 8.0 7.5 7.0 6.5 6.0 5.5 5.0 4.5 4.0 3.5 3.0 2.5 2.0 1.5 1.0 0.5 0.0
f1 (ppm)

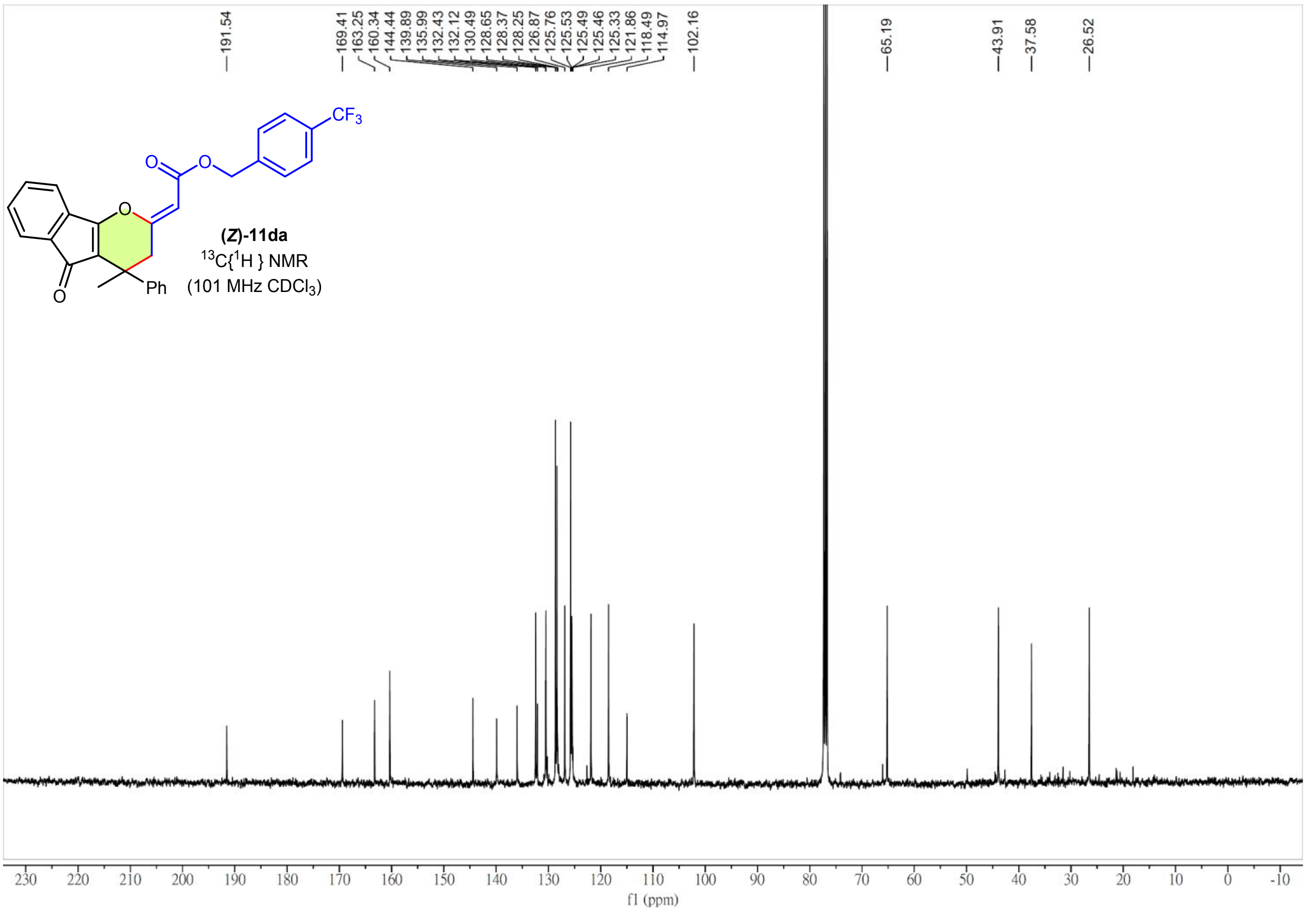
191.54
169.41 163.25 160.34 144.44 139.89 135.99 132.43 132.12 130.49 128.65 128.37 128.25 126.87 125.76 125.53 125.49 125.46 125.33 121.86 118.49 114.97
102.16
65.19
43.91
37.58
26.52
CF3
O
O
O
O
Ph
(Z)-11da
13C{1H } NMR
(101 MHz CDCl3)
230 220 210 200 190 180 170 160 150 140 130 120 110 100 90 80 70 60 50 40 30 20 10 0 -10
f1 (ppm)

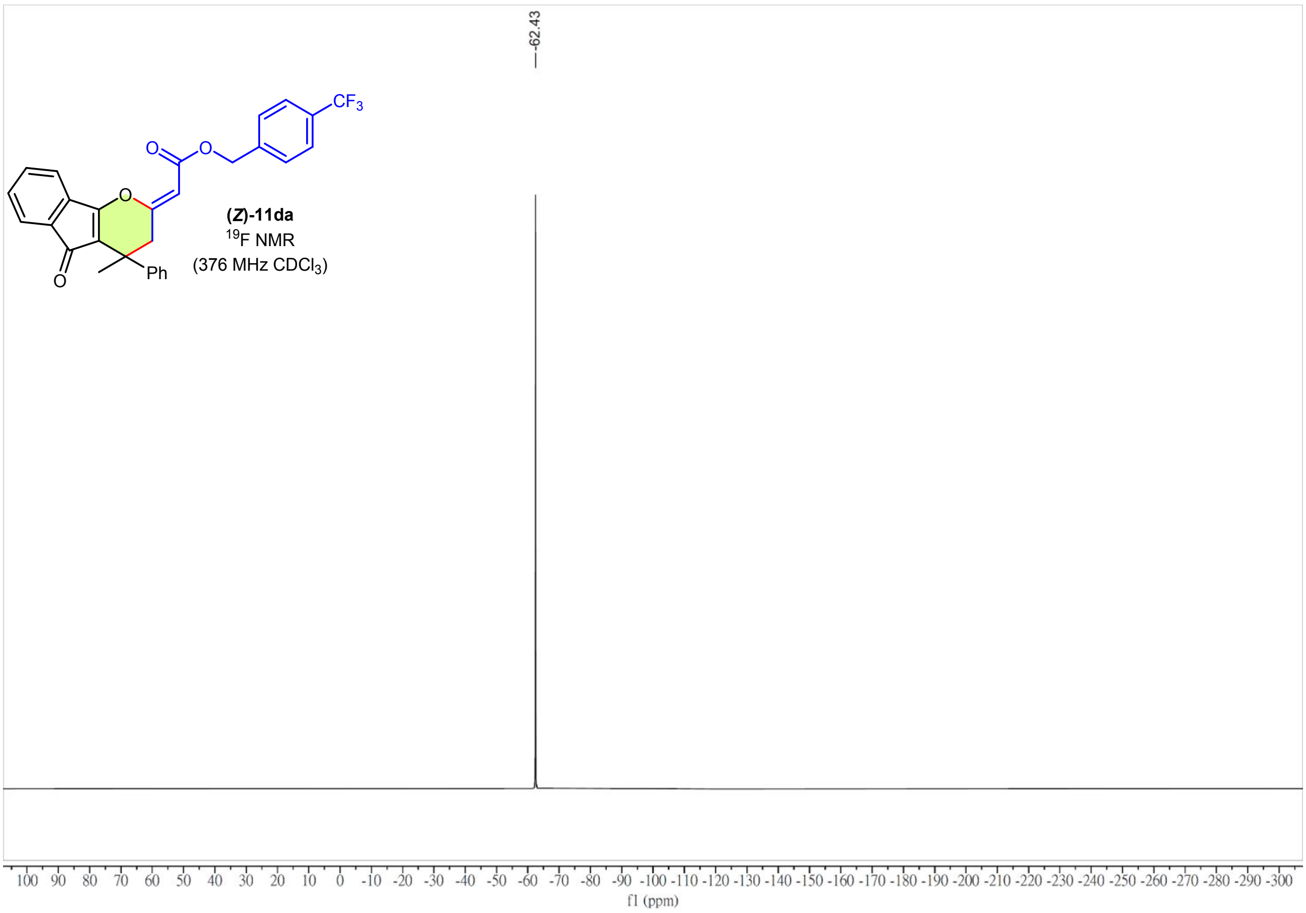

-62.43
CF3
O
O
O
O
Ph
(Z)-11da
19F NMR
(376 MHz CDCl3)
100 90 80 70 60 50 40 30 20 10 0 -10 -20 -30 -40 -50 -60 -70 -80 -90 -100 -110 -120 -130 -140 -150 -160 -170 -180 -190 -200 -210 -220 -230 -240 -250 -260 -270 -280 -290 -300
f1 (ppm)

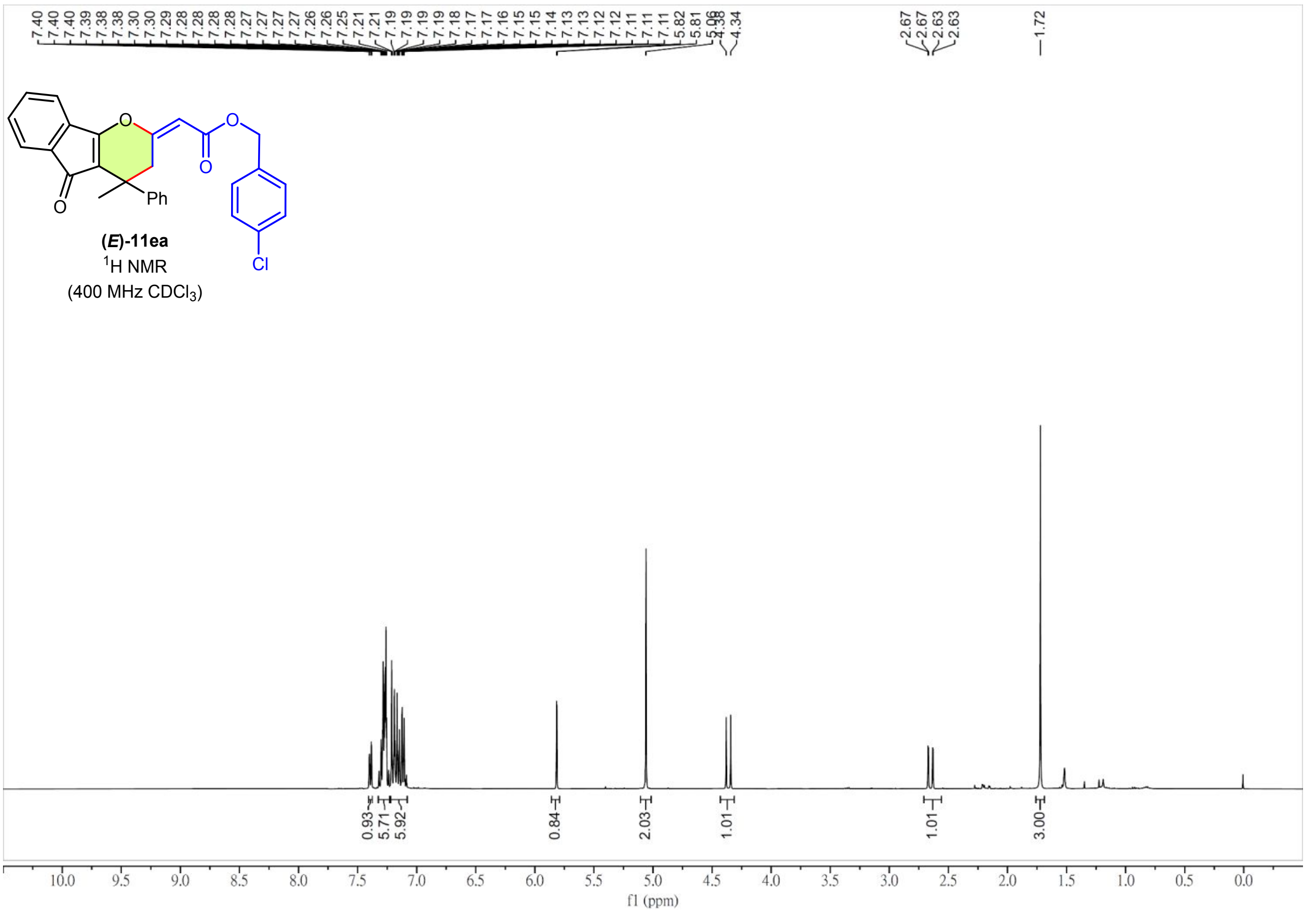
(E)-11ea
1H NMR
(400 MHz CDCl3)
f1 (ppm)

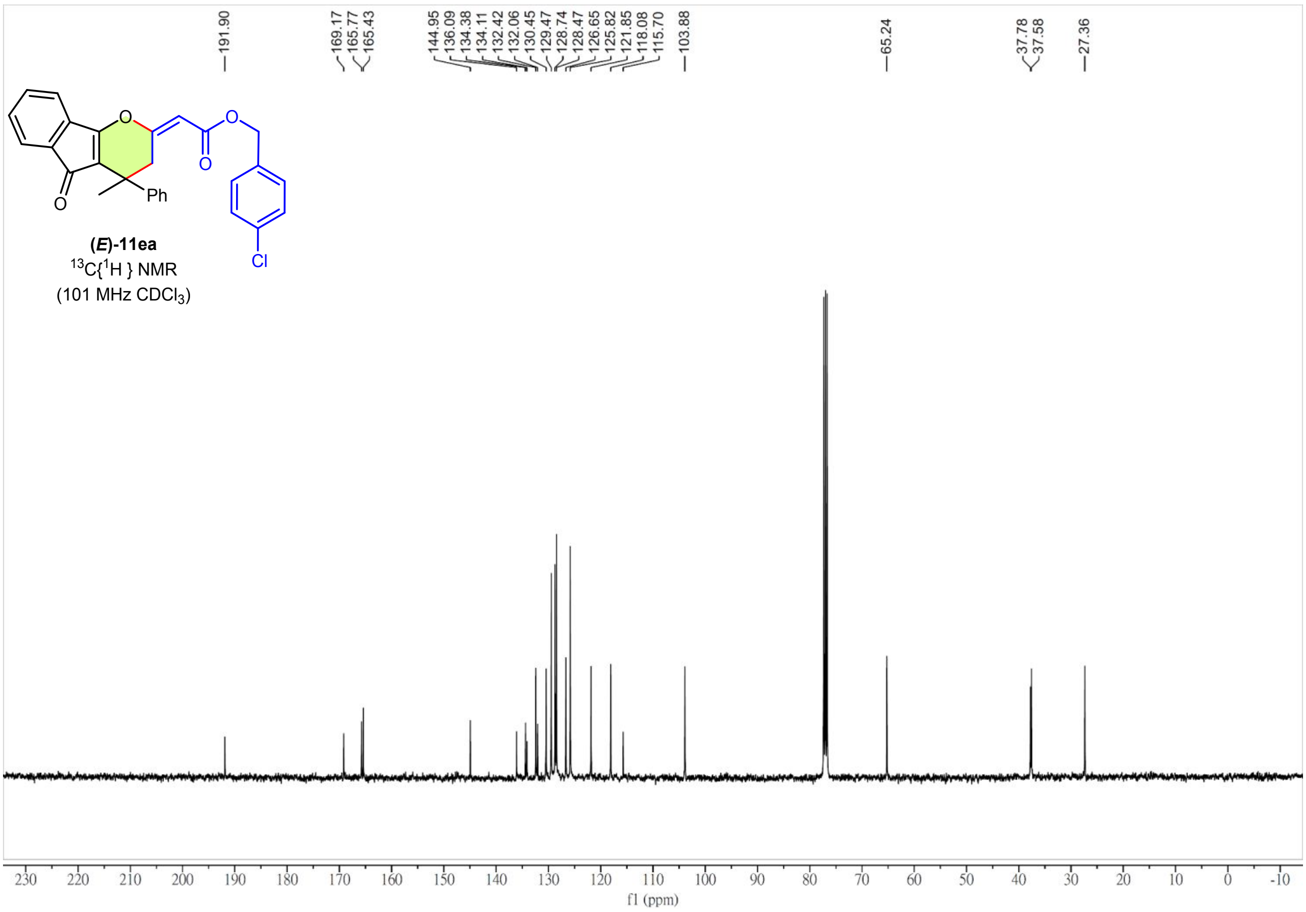
(E)-11ea
13C{1H } NMR
(101 MHz CDCl3)
f1 (ppm)

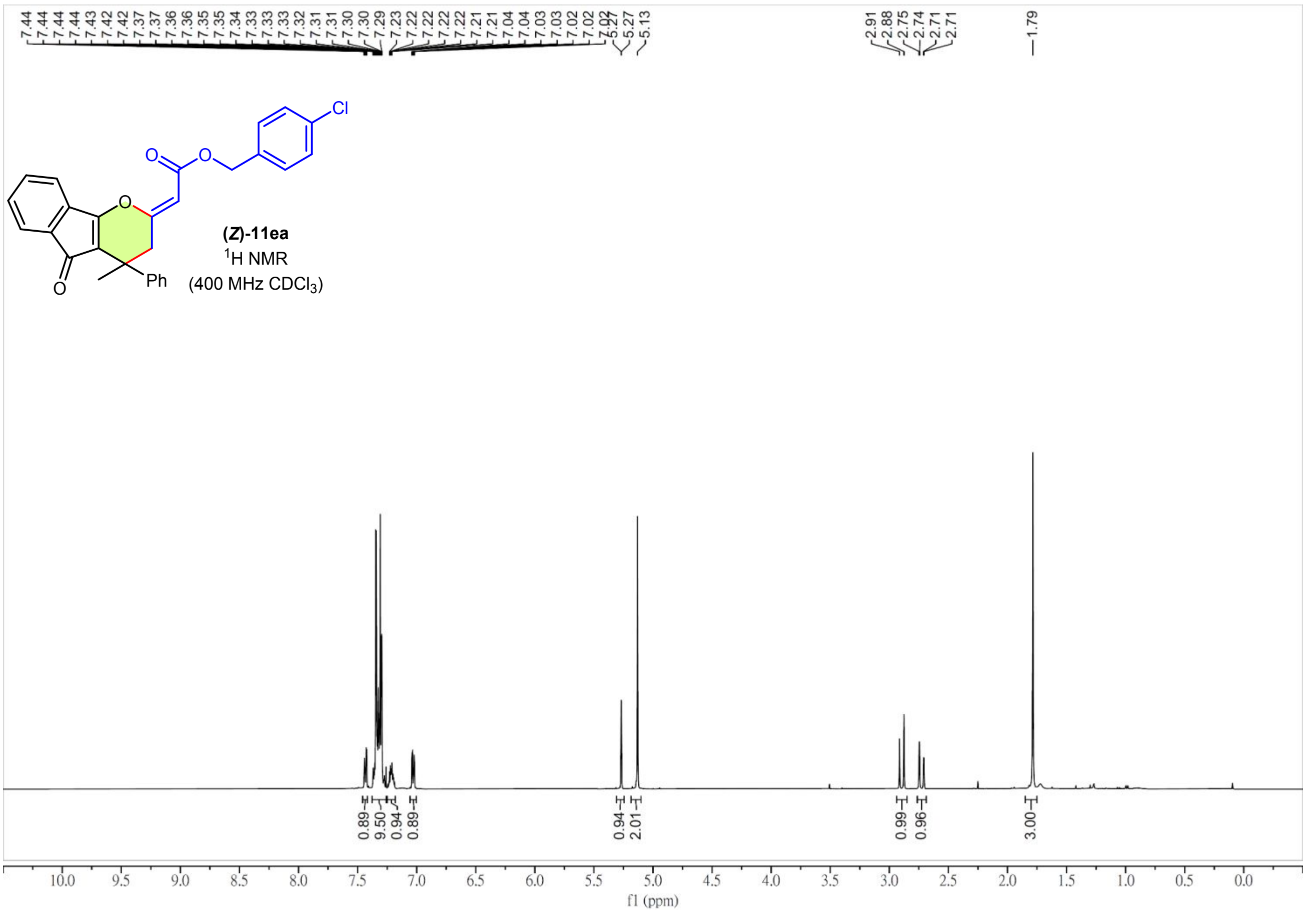

(Z)-11ea
^{1}H NMR
(400 MHz CDCl$_3$)
f1 (ppm)

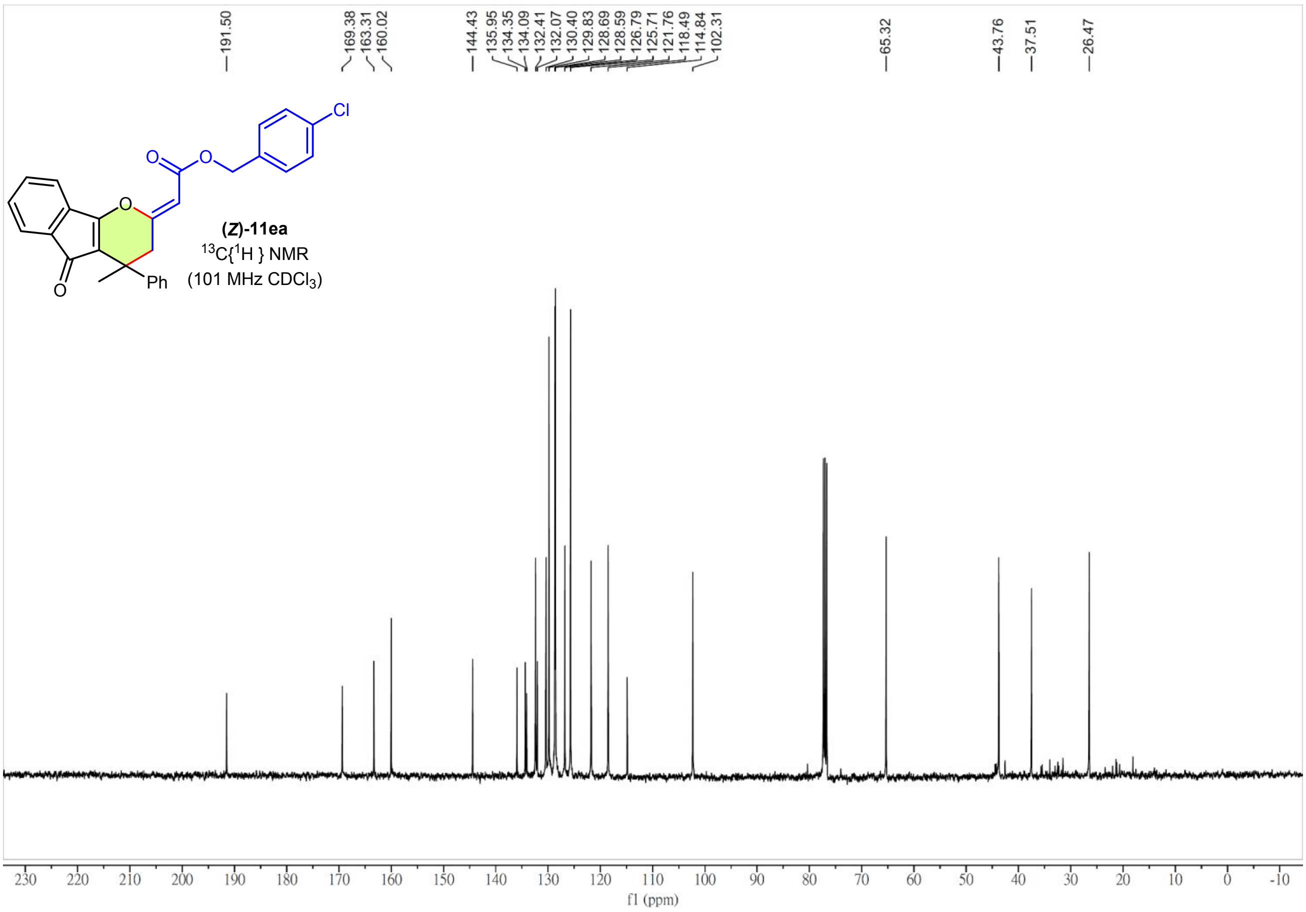

(Z)-11ea
^{13}C{^{1}H } NMR
(101 MHz CDCl$_3$)
f1 (ppm)

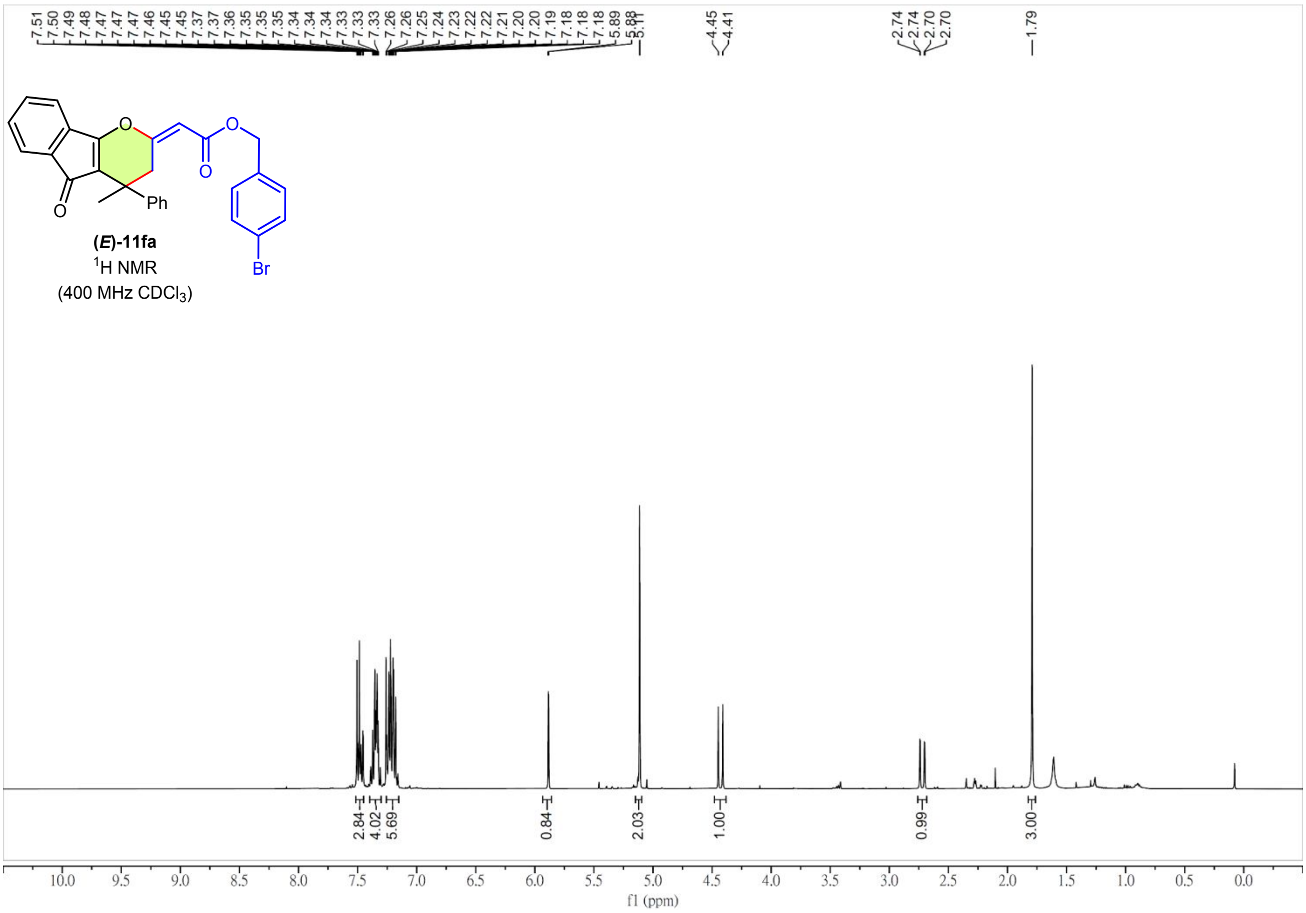
7.51 7.50 7.49 7.48 7.47 7.47 7.46 7.45 7.45 7.37 7.36 7.35 7.35 7.35 7.34 7.34 7.33 7.33 7.33 7.26 7.26 7.25 7.24 7.23 7.22 7.22 7.21 7.20 7.20 7.19 7.18 7.18 7.18 5.89 5.88 5.11
4.45 4.41
2.74 2.74 2.70 2.70
1.79
O
O
O
Ph
O
Br
(E)-11fa
1H NMR
(400 MHz CDCl3)
2.84 4.02 5.69 0.84 2.03 1.00 0.99 3.00
10.0 9.5 9.0 8.5 8.0 7.5 7.0 6.5 6.0 5.5 5.0 4.5 4.0 3.5 3.0 2.5 2.0 1.5 1.0 0.5 0.0
f1 (ppm)

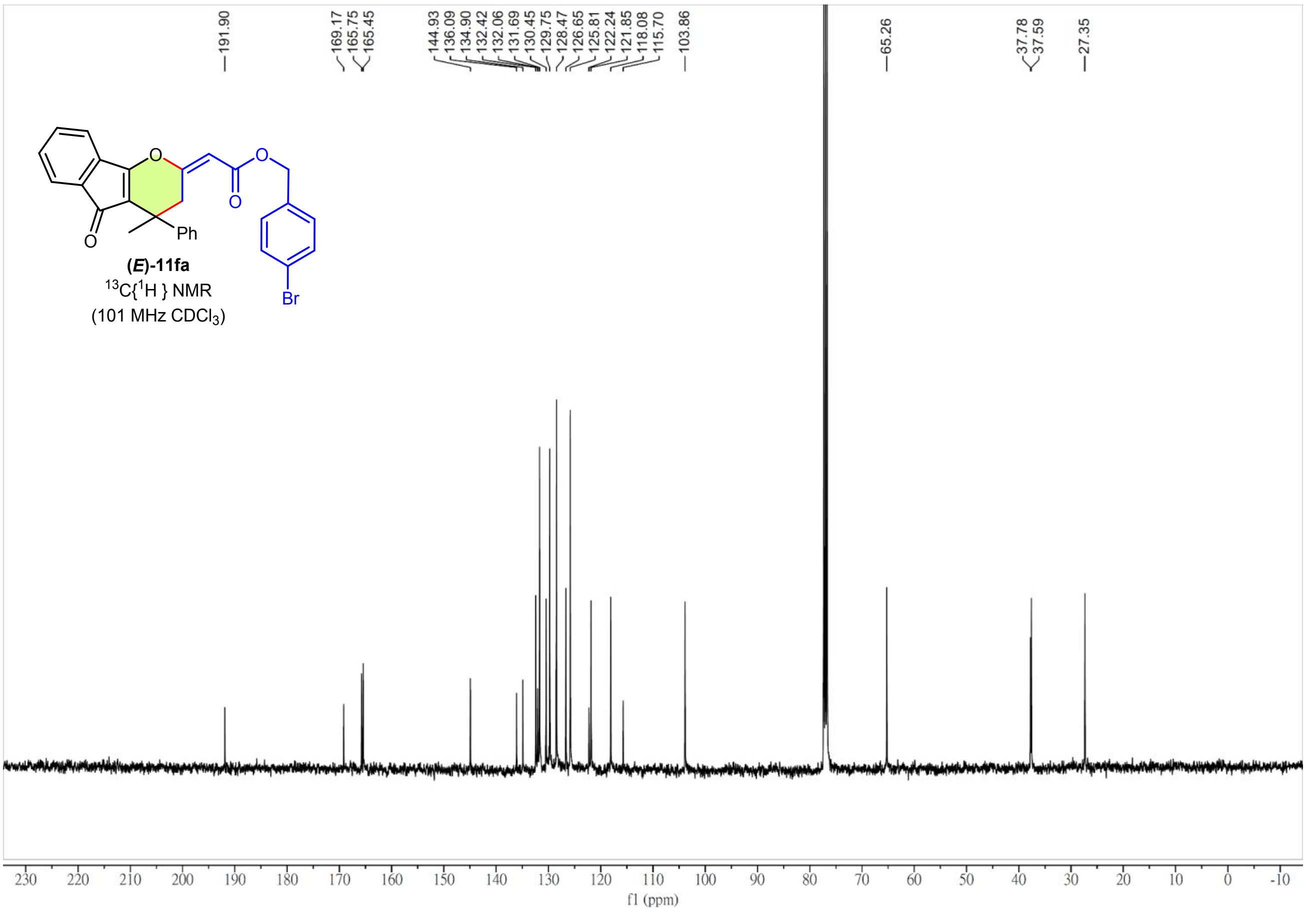
191.90
169.17 165.75 165.45
144.93 136.09 134.90 132.42 132.06 131.69 130.45 129.75 128.47 126.65 125.81 122.24 121.85 118.08 115.70
103.86
65.26
37.78 37.59
27.35
O
O
O
Ph
O
Br
(E)-11fa
13C{1H } NMR
(101 MHz CDCl3)
230 220 210 200 190 180 170 160 150 140 130 120 110 100 90 80 70 60 50 40 30 20 10 0 -10
f1 (ppm)

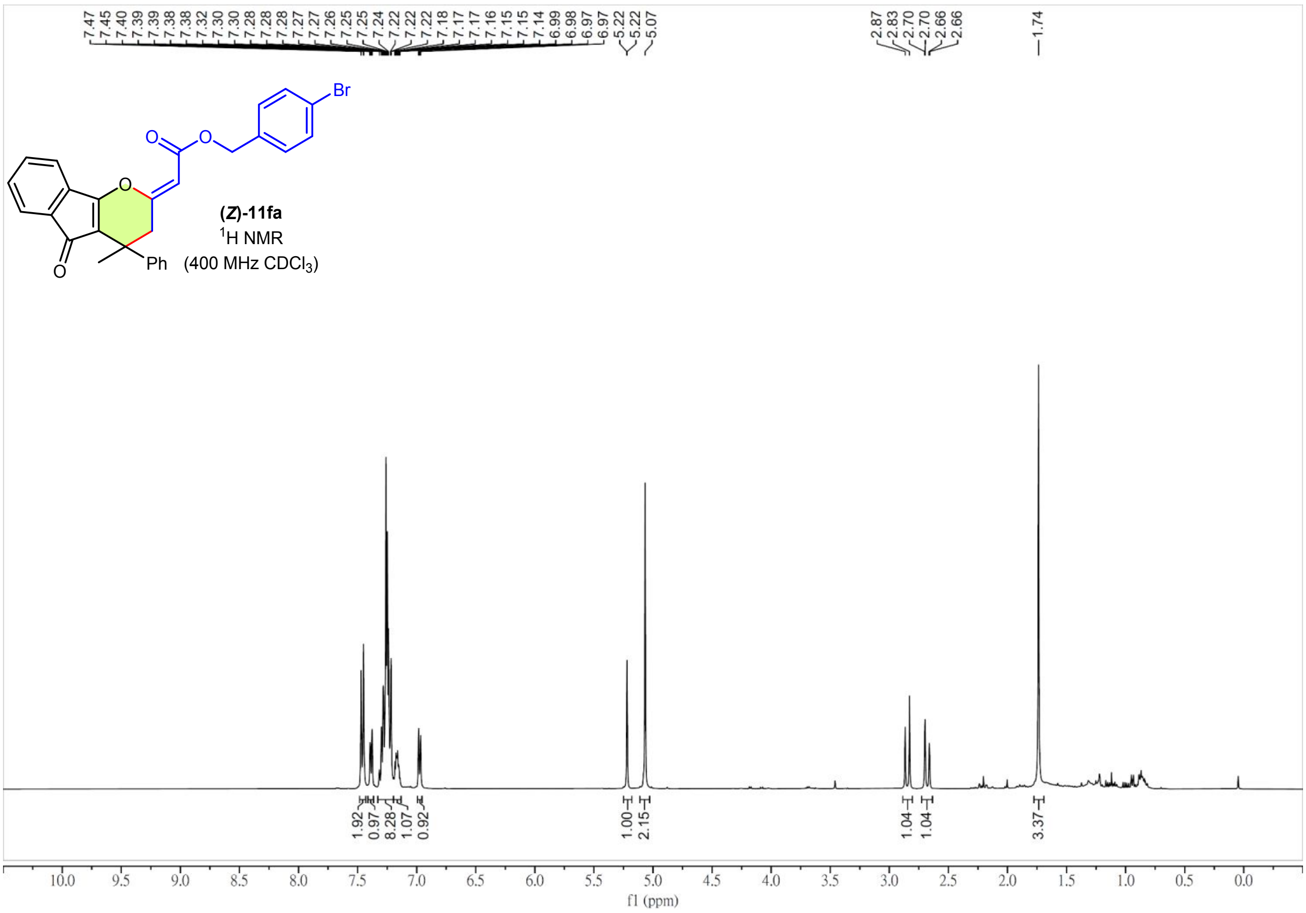
7.47 7.45 7.40 7.39 7.39 7.38 7.38 7.32 7.30 7.28 7.28 7.28 7.27 7.27 7.26 7.25 7.25 7.24 7.22 7.22 7.22 7.18 7.17 7.17 7.16 7.15 7.15 7.14 6.99 6.98 6.97 6.97 5.22 5.22 5.07
2.87 2.83 2.70 2.70 2.66 2.66
1.74
Br
O
O
O
O
Ph
(Z)-11fa
1H NMR
(400 MHz CDCl3)
1.92 0.97 8.28 1.07 0.92
1.00 2.15
1.04 1.04
3.37
f1 (ppm)

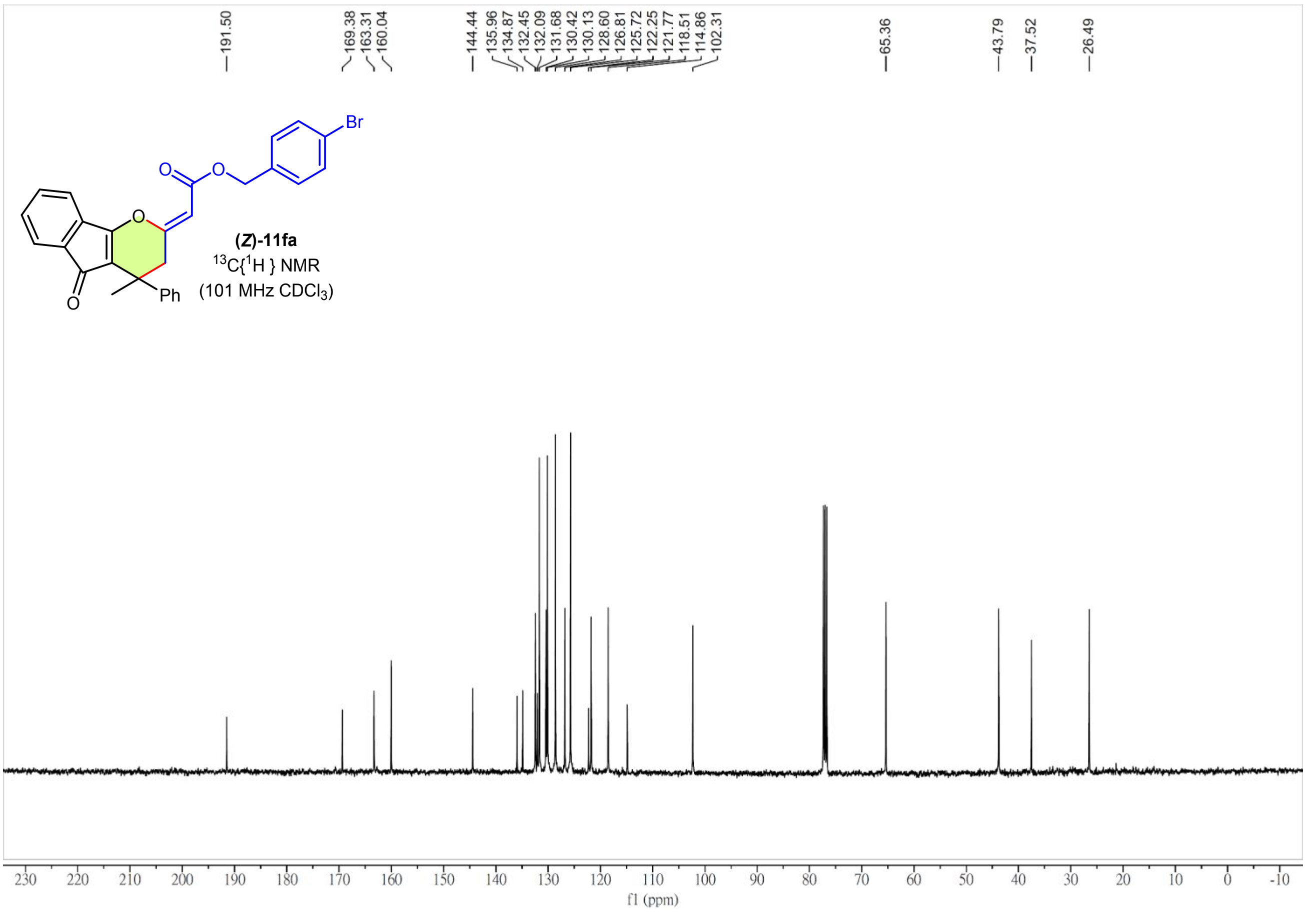
191.50
169.38 163.31 160.04
144.44 135.96 134.87 132.45 132.09 131.68 130.42 130.13 128.60 126.81 125.72 122.25 121.77 118.51 114.86 102.31
65.36
43.79
37.52
26.49
Br
O
O
O
O
Ph
(Z)-11fa
13C{1H } NMR
(101 MHz CDCl3)
f1 (ppm)

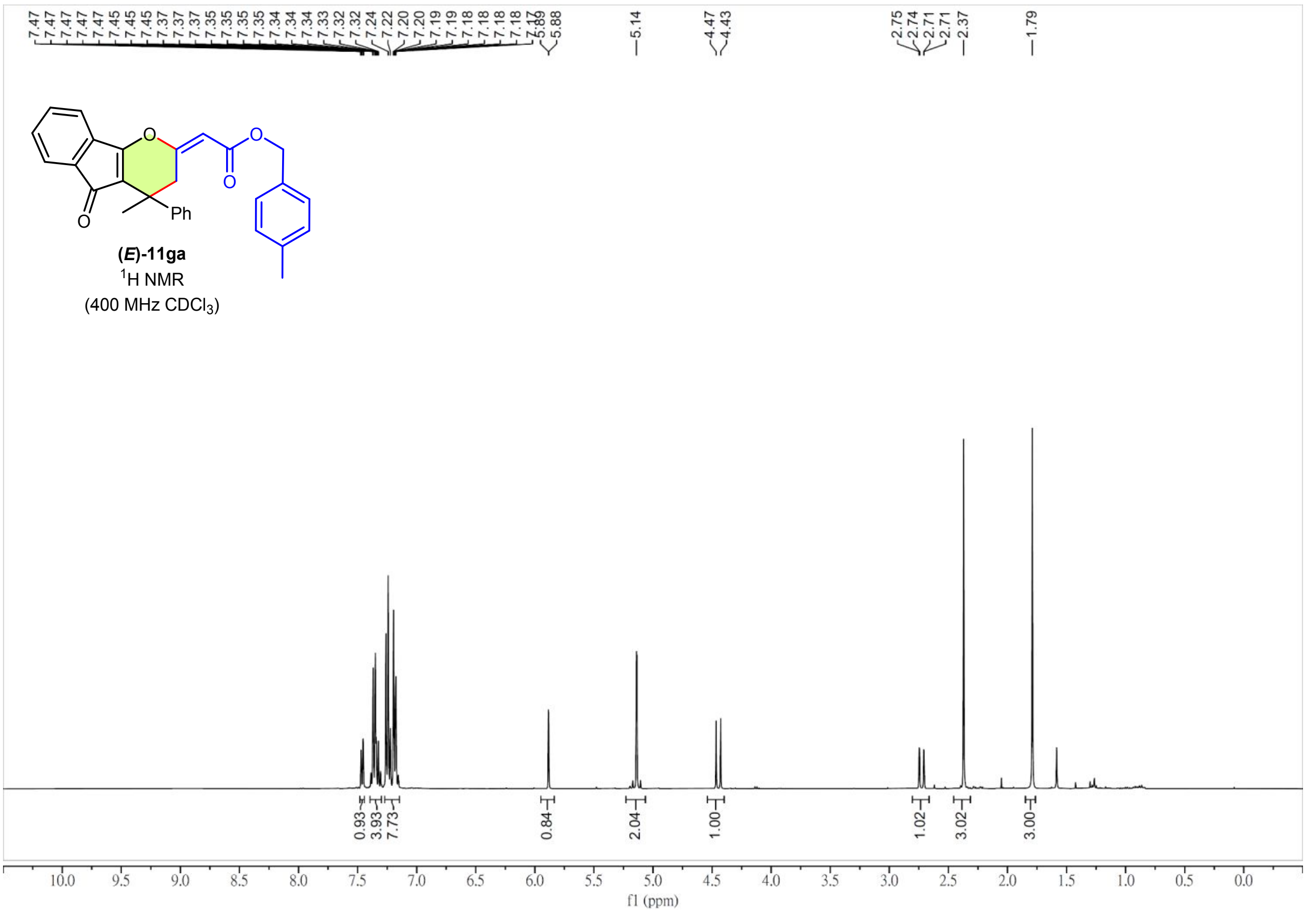
7.47 7.47 7.47 7.47 7.45 7.45 7.37 7.37 7.35 7.35 7.35 7.34 7.34 7.33 7.32 7.32 7.24 7.22 7.20 7.20 7.19 7.19 7.18 7.18 7.18 7.18 7.17 5.89 5.88
5.14
4.47 4.43
2.75 2.74 2.71 2.71 2.37
1.79
Ph
(E)-11ga
^{1}H NMR
(400 MHz CDCl$_3$)
0.93 3.93 7.73 0.84 2.04 1.00 1.02 3.02 3.00
f1 (ppm)

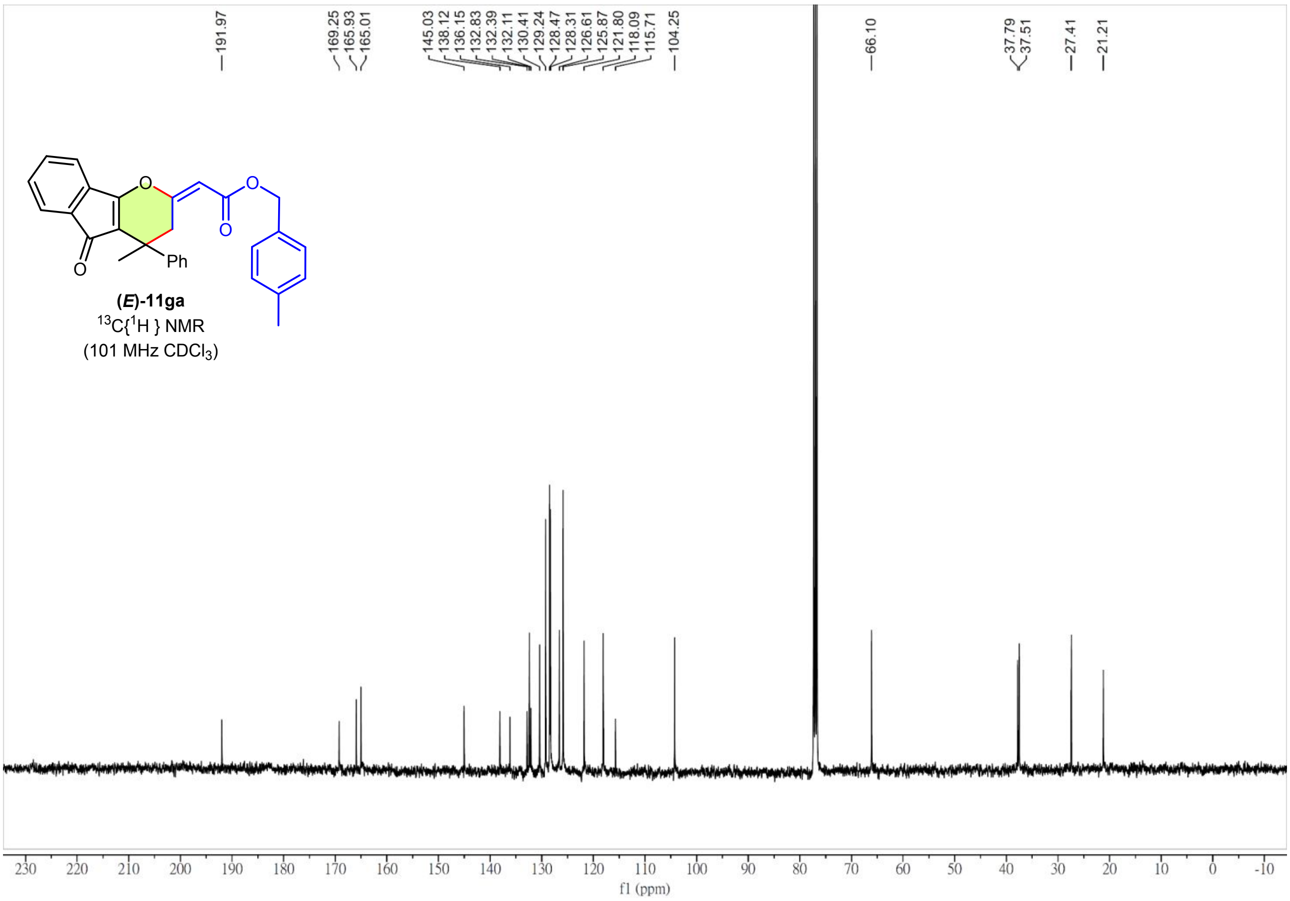
191.97
169.25 165.93 165.01
145.03 138.12 136.15 132.83 132.39 132.11 130.41 129.24 128.47 128.31 126.61 125.87 121.80 118.09 115.71
104.25
66.10
37.79 37.51
27.41
21.21
Ph
(E)-11ga
^{13}C{^{1}H } NMR
(101 MHz CDCl$_3$)
f1 (ppm)

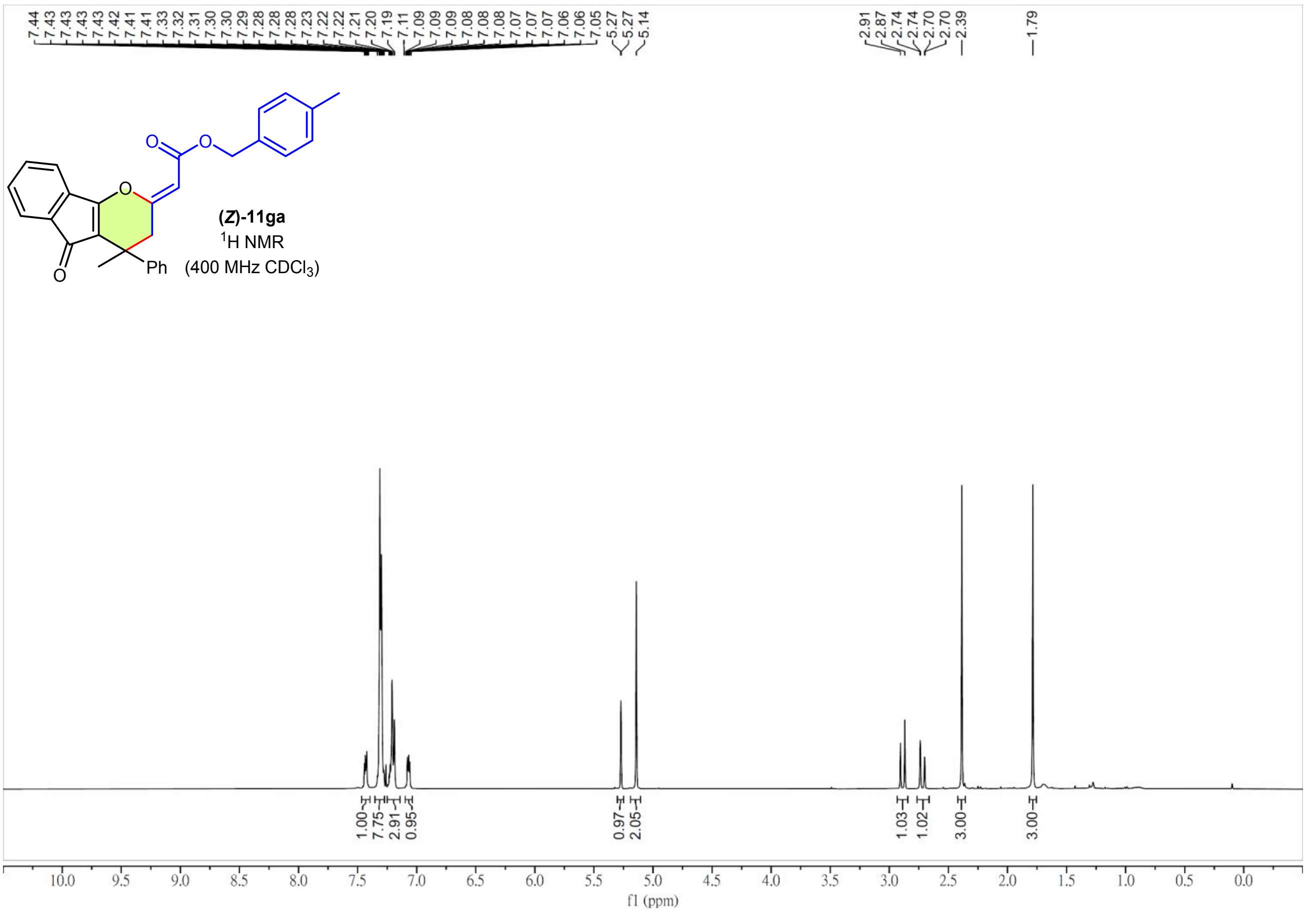
7.44
7.43
7.43
7.43
7.42
7.41
7.41
7.33
7.32
7.31
7.30
7.30
7.29
7.28
7.28
7.28
7.23
7.22
7.22
7.21
7.20
7.19
7.11
7.09
7.09
7.09
7.08
7.08
7.07
7.07
7.07
7.06
7.06
7.05
5.27
5.27
5.14
2.91
2.87
2.74
2.74
2.70
2.70
2.39
1.79
(Z)-11ga
1H NMR
(400 MHz CDCl3)
O
O
O
O
Ph
1.00
7.75
2.91
0.95
0.97
2.05
1.03
1.02
3.00
3.00
f1 (ppm)

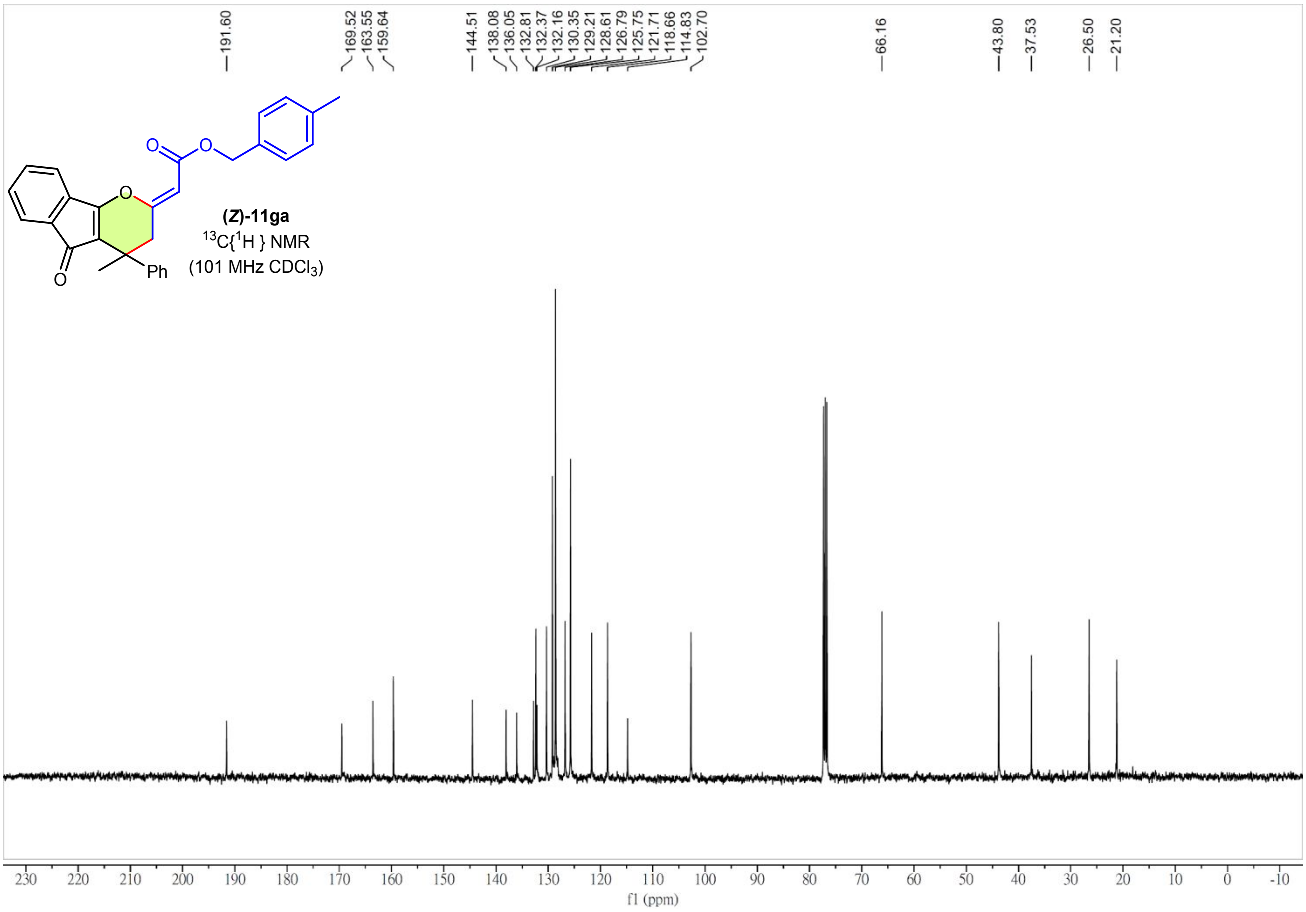
191.60
169.52
163.55
159.64
144.51
138.08
136.05
132.81
132.37
132.16
130.35
129.21
128.61
126.79
125.75
121.71
118.66
114.83
102.70
66.16
43.80
37.53
26.50
21.20
(Z)-11ga
13C{1H } NMR
(101 MHz CDCl3)
O
O
O
O
Ph
f1 (ppm)

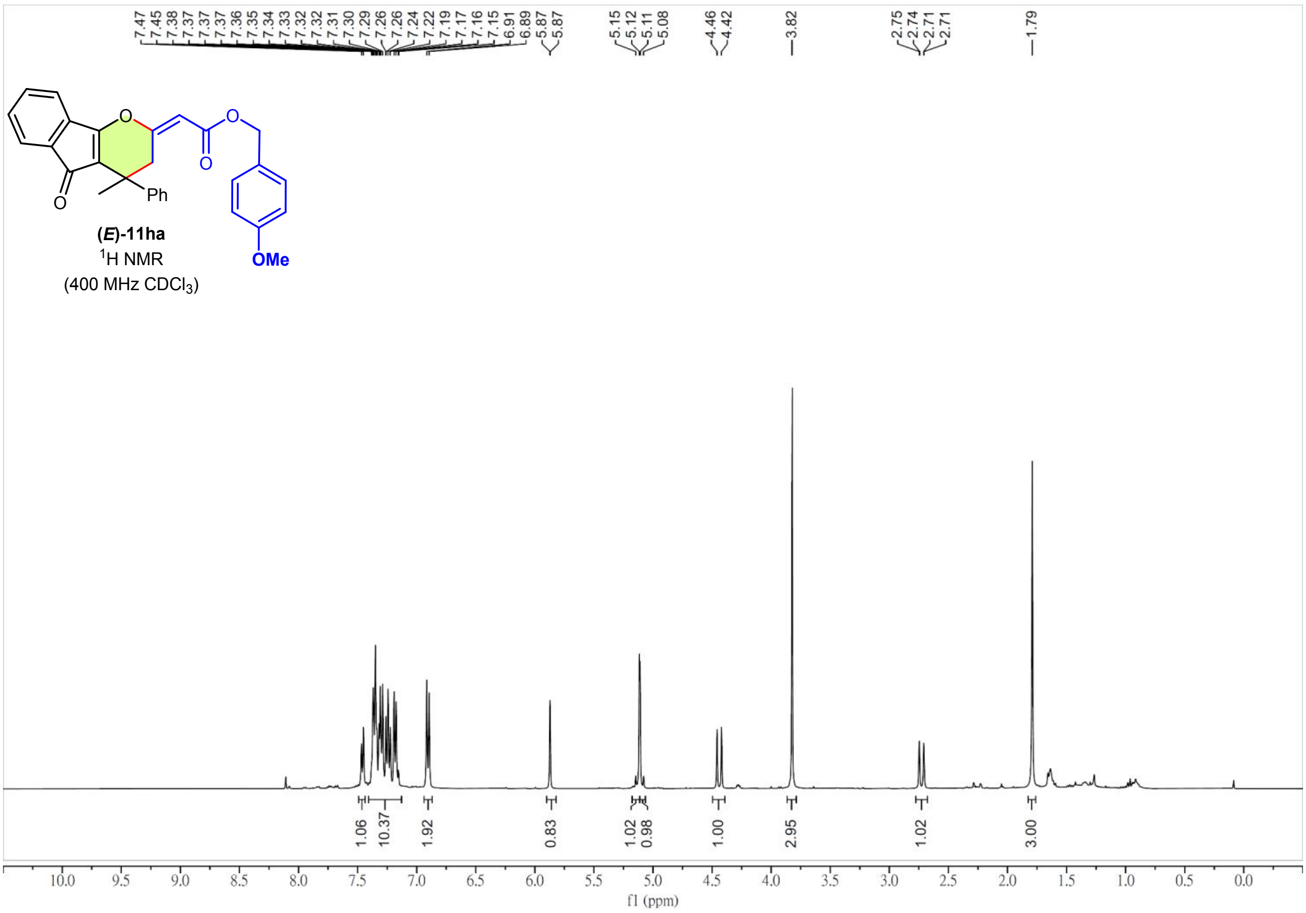
(E)-11ha
1H NMR
(400 MHz CDCl3)
OMe
Ph
O
7.47 7.45 7.38 7.37 7.37 7.37 7.36 7.35 7.34 7.33 7.32 7.32 7.31 7.30 7.29 7.26 7.26 7.24 7.22 7.19 7.17 7.16 7.15 6.91 6.89 5.87 5.87
5.15 5.12 5.11 5.08
4.46 4.42
3.82
2.75 2.74 2.71 2.71
1.79
1.06 10.37 1.92 0.83 1.02 0.98 1.00 2.95 1.02 3.00
f1 (ppm)

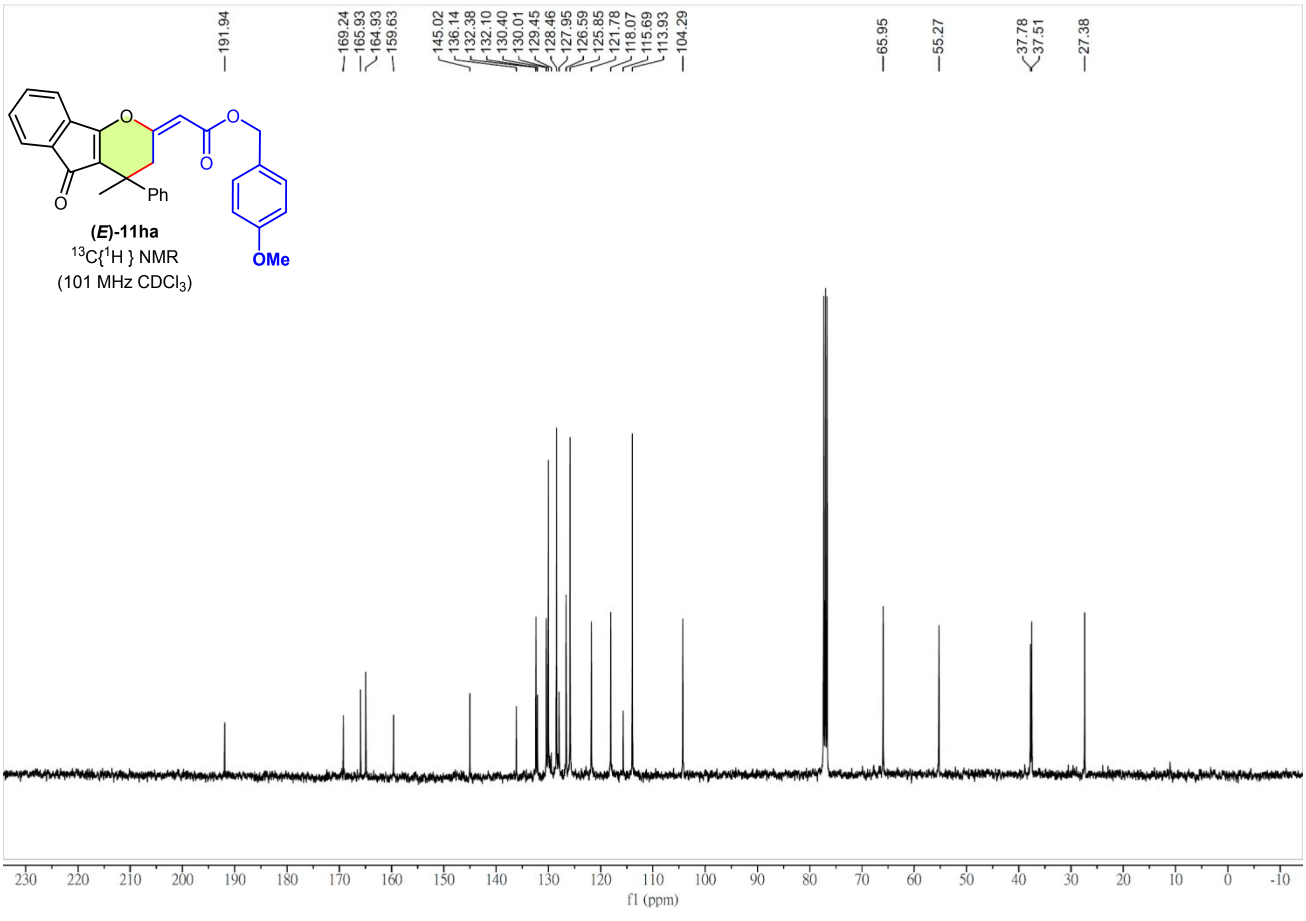
(E)-11ha
13C{1H } NMR
(101 MHz CDCl3)
OMe
Ph
O
191.94
169.24 165.93 164.93 159.63
145.02 136.14 132.38 132.10 130.40 130.01 129.45 128.46 127.95 126.59 125.85 121.78 118.07 115.69 113.93 104.29
65.95
55.27
37.78 37.51
27.38
f1 (ppm)

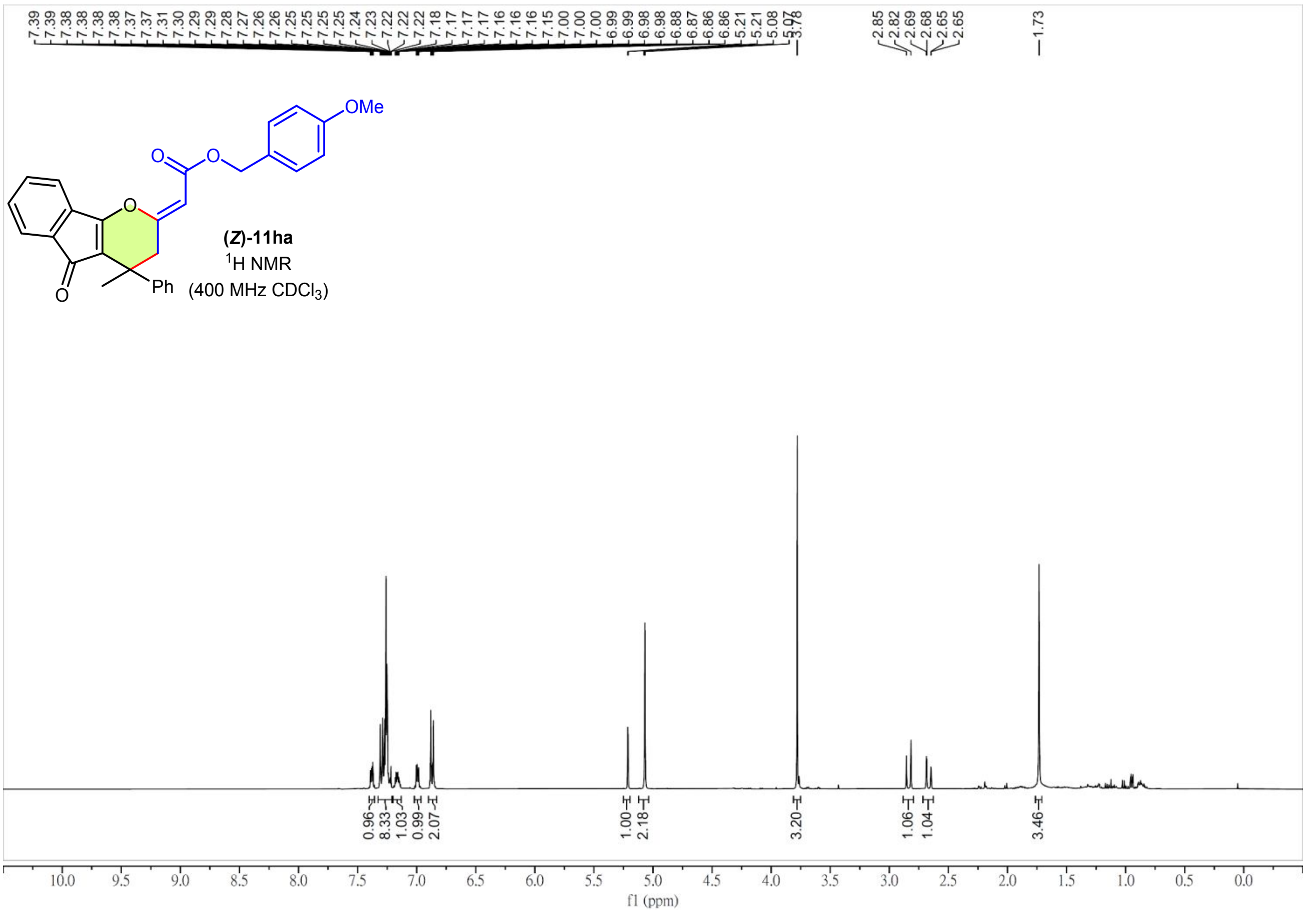
(Z)-11ha
^{1}H NMR
(400 MHz CDCl$_3$)
OMe
f1 (ppm)

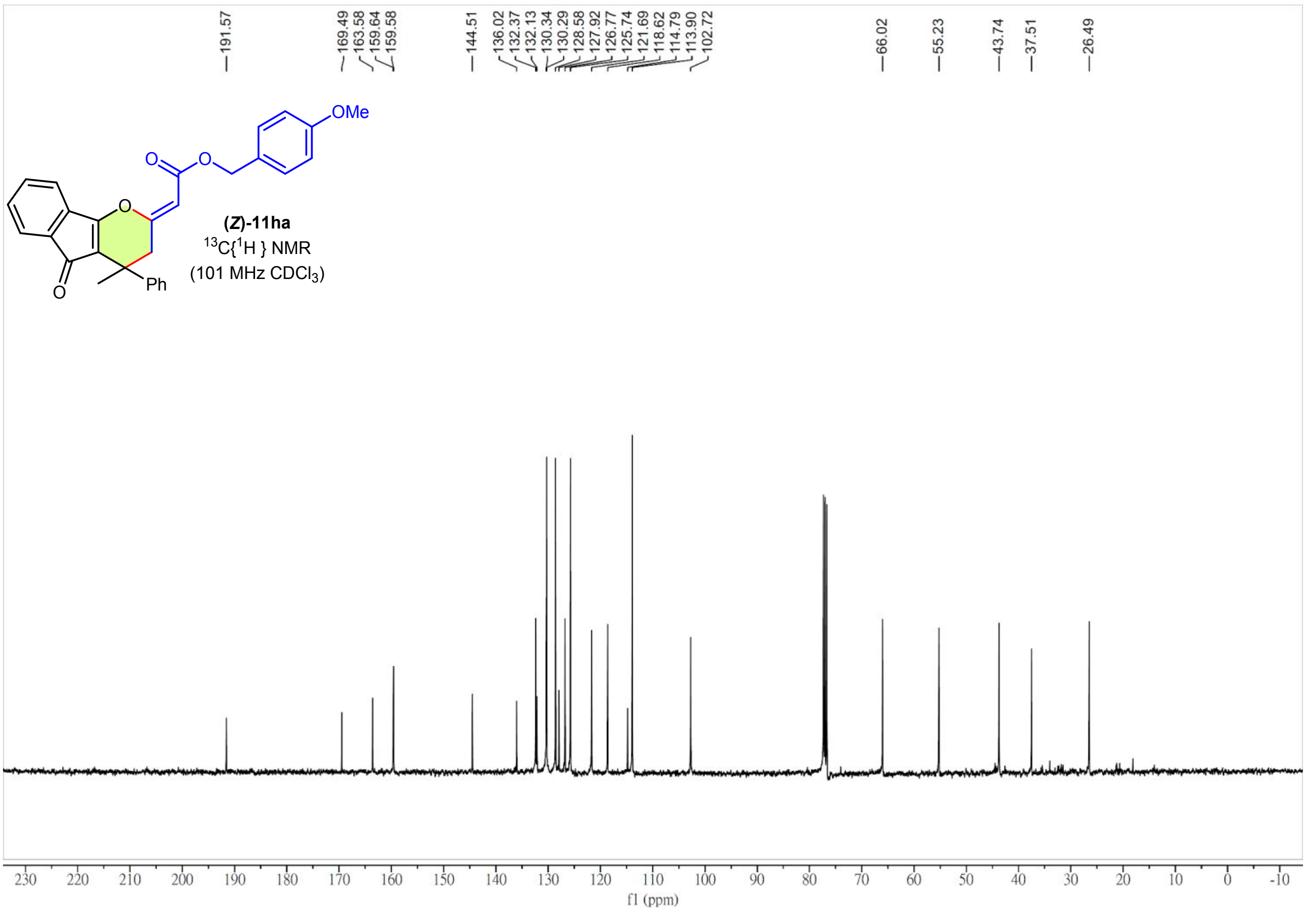
(Z)-11ha
^{13}C{^{1}H } NMR
(101 MHz CDCl$_3$)
OMe
f1 (ppm)

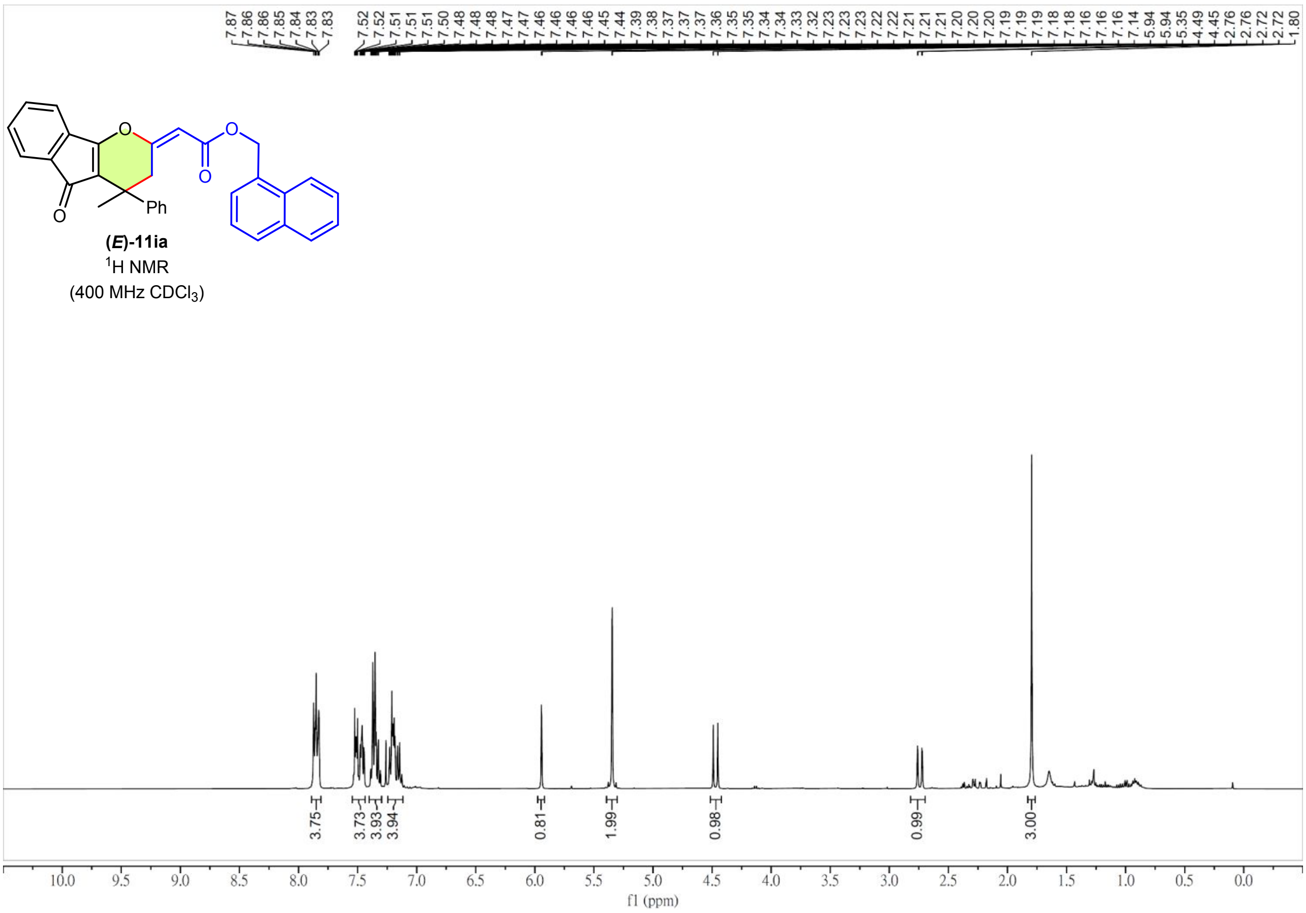

(E)-11ia
1H NMR
(400 MHz CDCl3)
7.87 7.86 7.86 7.85 7.84 7.83 7.83
7.52 7.52 7.51 7.51 7.50 7.48 7.48 7.48 7.47 7.47 7.46 7.46 7.46 7.45 7.44 7.39 7.38 7.37 7.37 7.37 7.36 7.35 7.35 7.34 7.33 7.32 7.23 7.23 7.23 7.22 7.22 7.21 7.21 7.21 7.20 7.20 7.19 7.19 7.19 7.19 7.18 7.16 7.16 7.14 5.94 5.94 5.35 4.49 4.45 2.76 2.72 1.80
3.75 3.73 3.93 3.94 0.81 1.99 0.98 0.99 3.00
10.0 9.5 9.0 8.5 8.0 7.5 7.0 6.5 6.0 5.5 5.0 4.5 4.0 3.5 3.0 2.5 2.0 1.5 1.0 0.5 0.0
f1 (ppm)

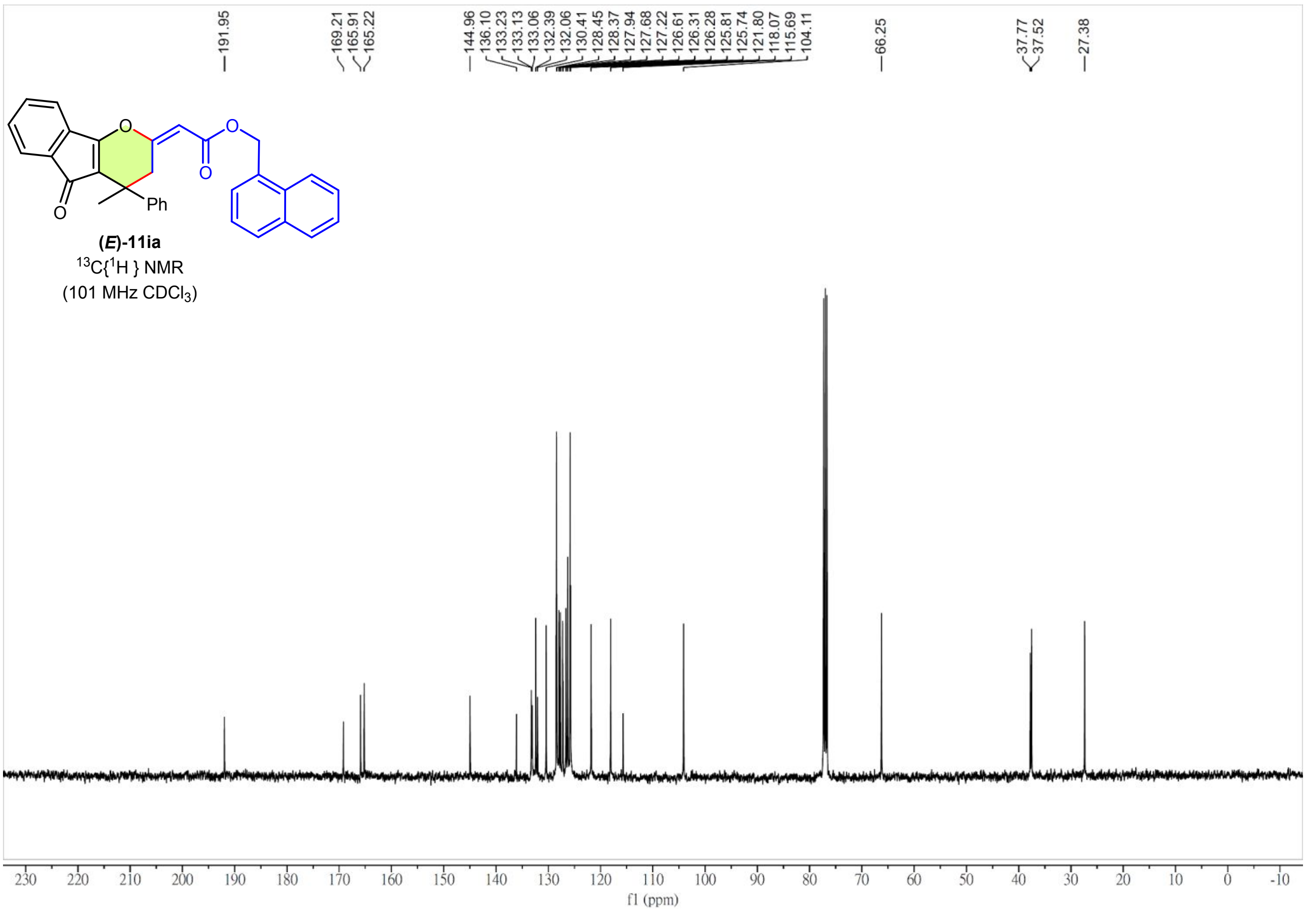

(E)-11ia
13C{1H } NMR
(101 MHz CDCl3)
191.95
169.21 165.91 165.22
144.96 136.10 133.23 133.13 133.06 132.39 132.06 130.41 128.45 128.37 127.94 127.68 127.22 126.61 126.31 126.28 125.81 125.74 121.80 118.07 115.69 104.11
66.25
37.77 37.52
27.38
230 220 210 200 190 180 170 160 150 140 130 120 110 100 90 80 70 60 50 40 30 20 10 0 -10
f1 (ppm)

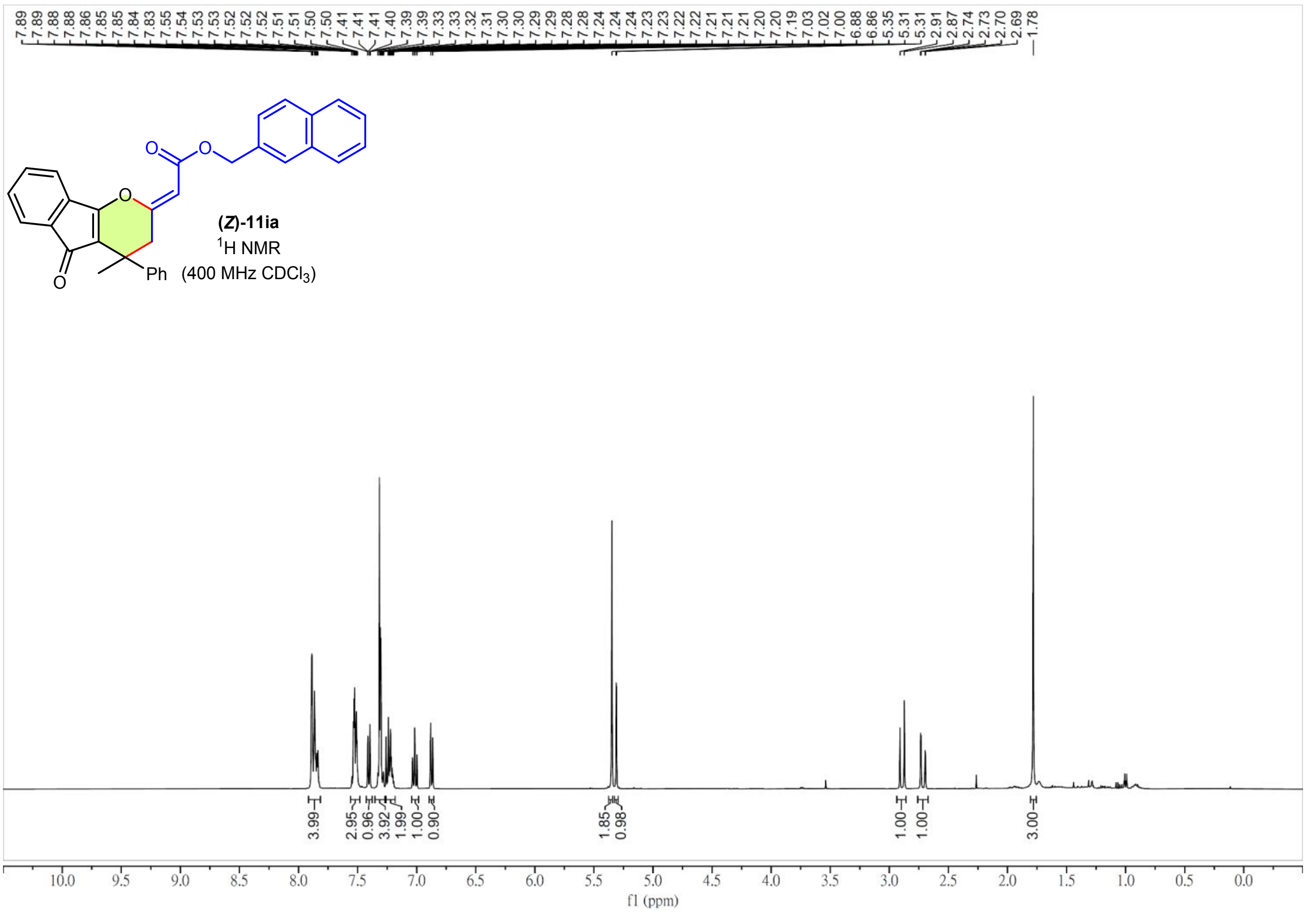
(Z)-11ia
1H NMR
(400 MHz CDCl3)
O
Ph
f1 (ppm)

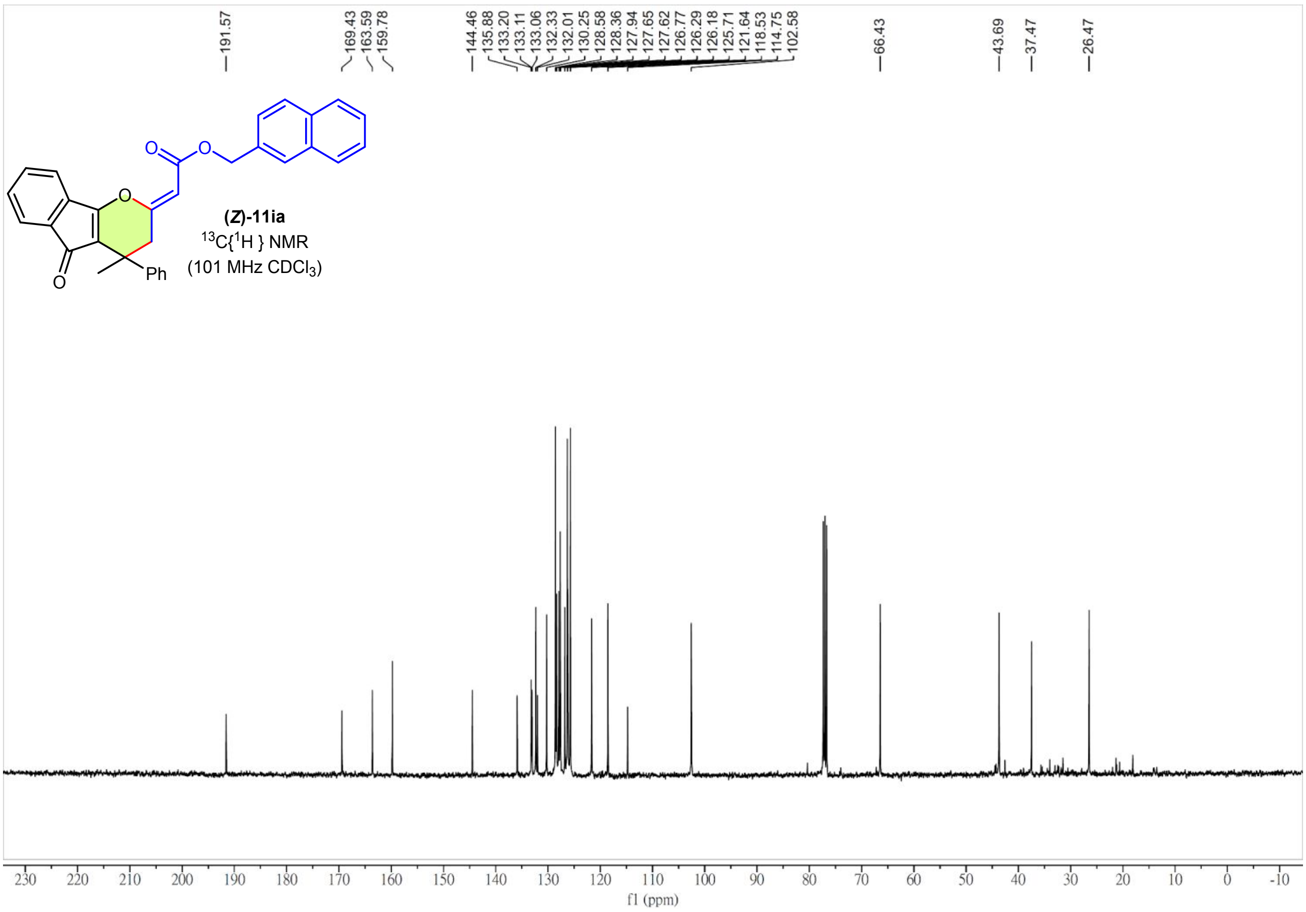
(Z)-11ia
13C{1H } NMR
(101 MHz CDCl3)
O
Ph
f1 (ppm)

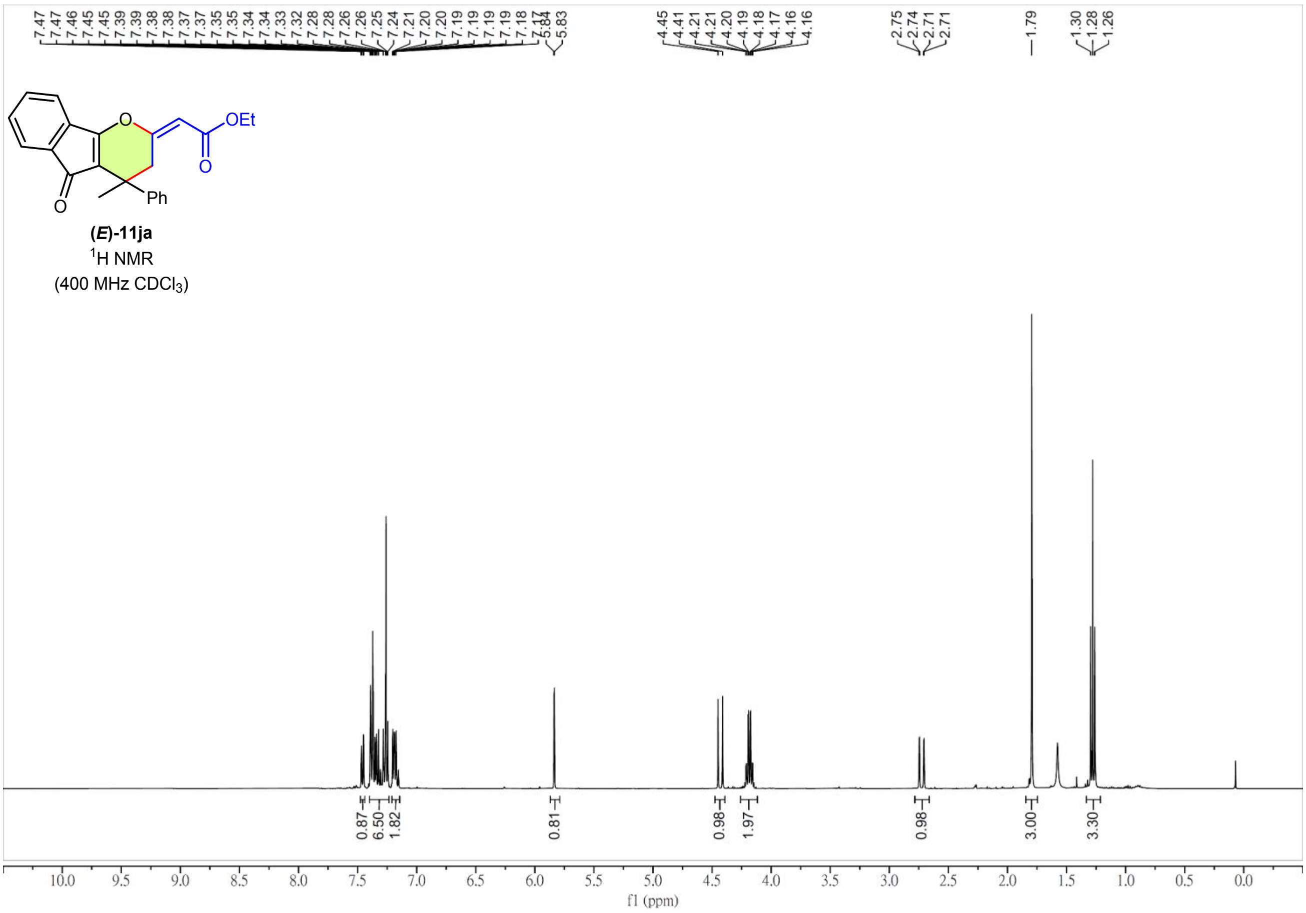

7.47 7.47 7.46 7.45 7.45 7.39 7.39 7.38 7.38 7.37 7.37 7.35 7.35 7.34 7.34 7.33 7.32 7.28 7.28 7.26 7.26 7.25 7.24 7.21 7.21 7.20 7.20 7.19 7.19 7.19 7.19 7.18 7.17 5.84 5.83
4.45 4.41 4.21 4.21 4.20 4.19 4.18 4.17 4.16 4.16
2.75 2.74 2.71 2.71
1.79
1.30 1.28 1.26
OEt
O
O
O
Ph
(*E*)-11ja
^{1}H NMR
(400 MHz CDCl$_3$)
0.87 6.50 1.82 0.81 0.98 1.97 0.98 3.00 3.30
f1 (ppm)

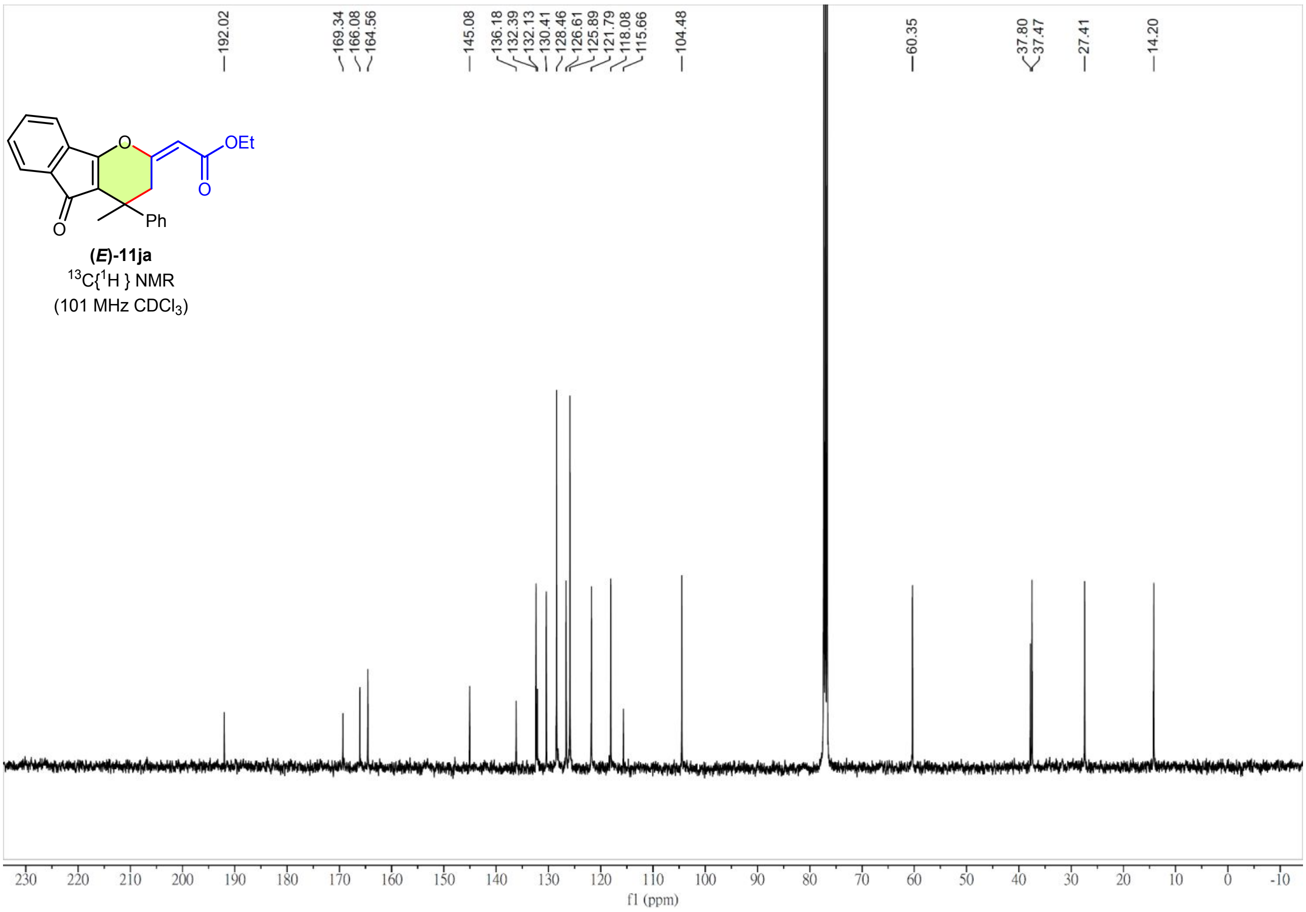

192.02
169.34 166.08 164.56
145.08
136.18 132.39 132.13 130.41 128.46 126.61 125.89 121.79 118.08 115.66
104.48
60.35
37.80 37.47
27.41
14.20
OEt
O
O
O
Ph
(*E*)-11ja
^{13}C{^{1}H } NMR
(101 MHz CDCl$_3$)
f1 (ppm)

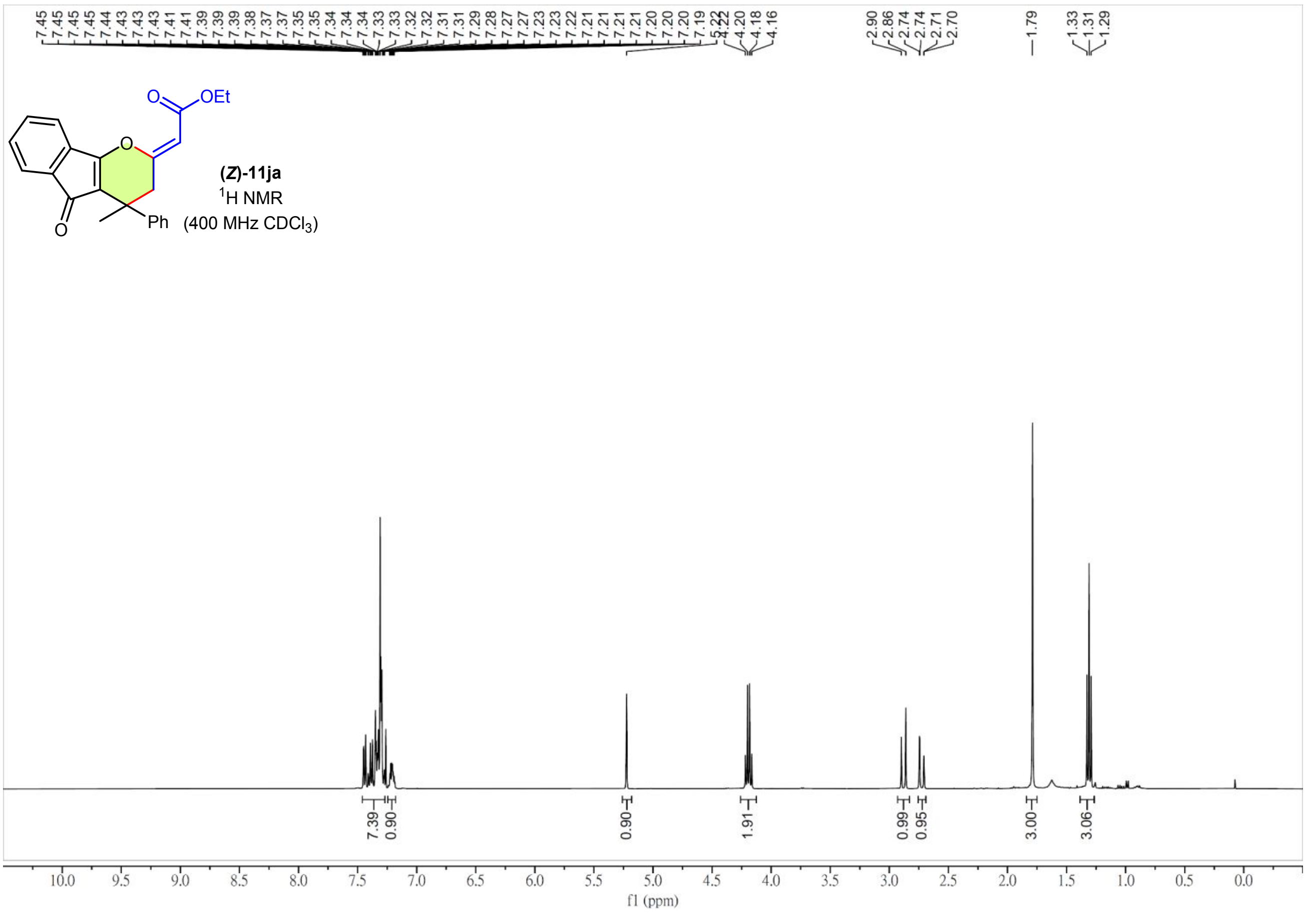
(Z)-11ja
^{1}H NMR
(400 MHz CDCl$_3$)
f1 (ppm)

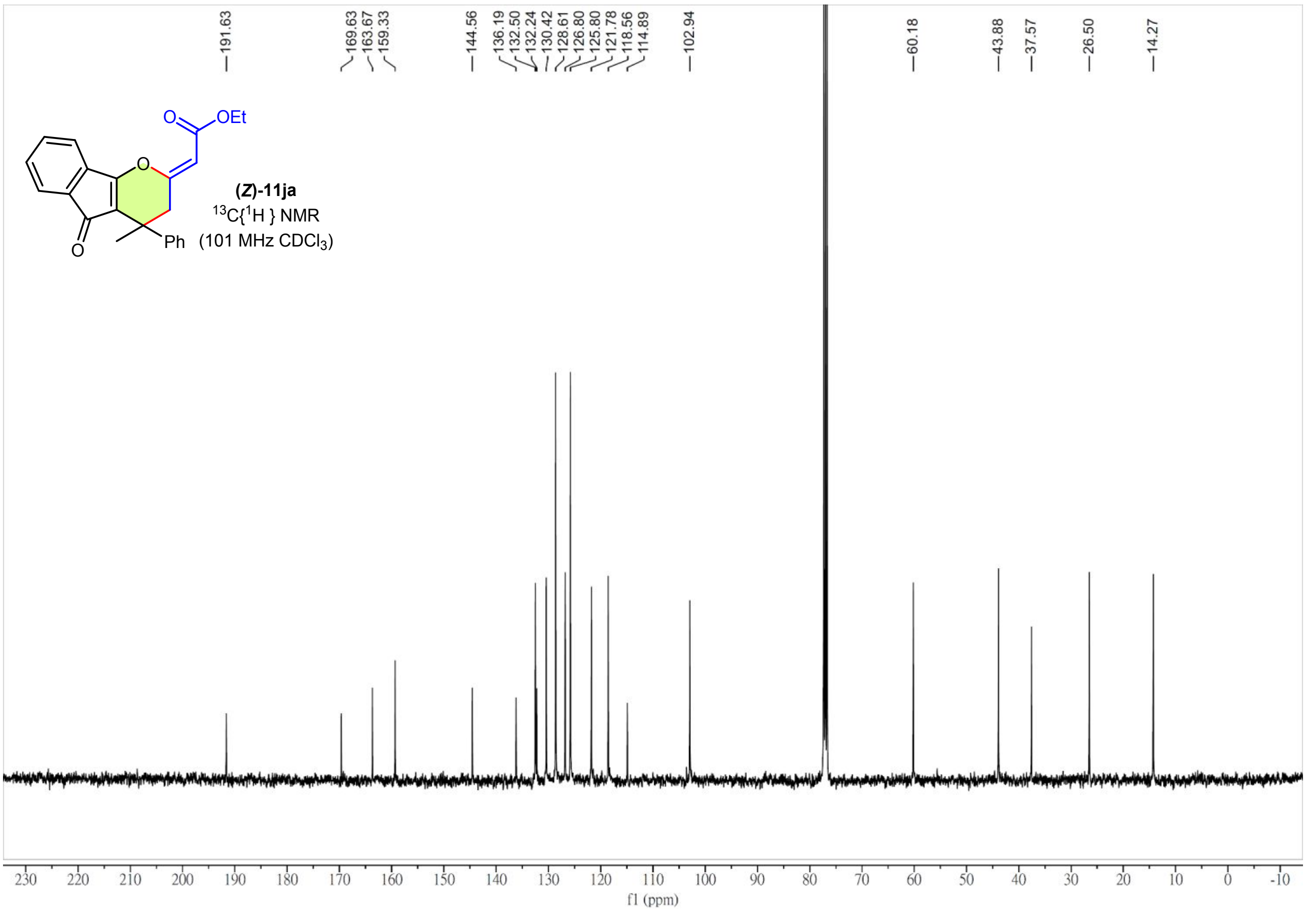
(Z)-11ja
^{13}C{^{1}H } NMR
(101 MHz CDCl$_3$)
f1 (ppm)

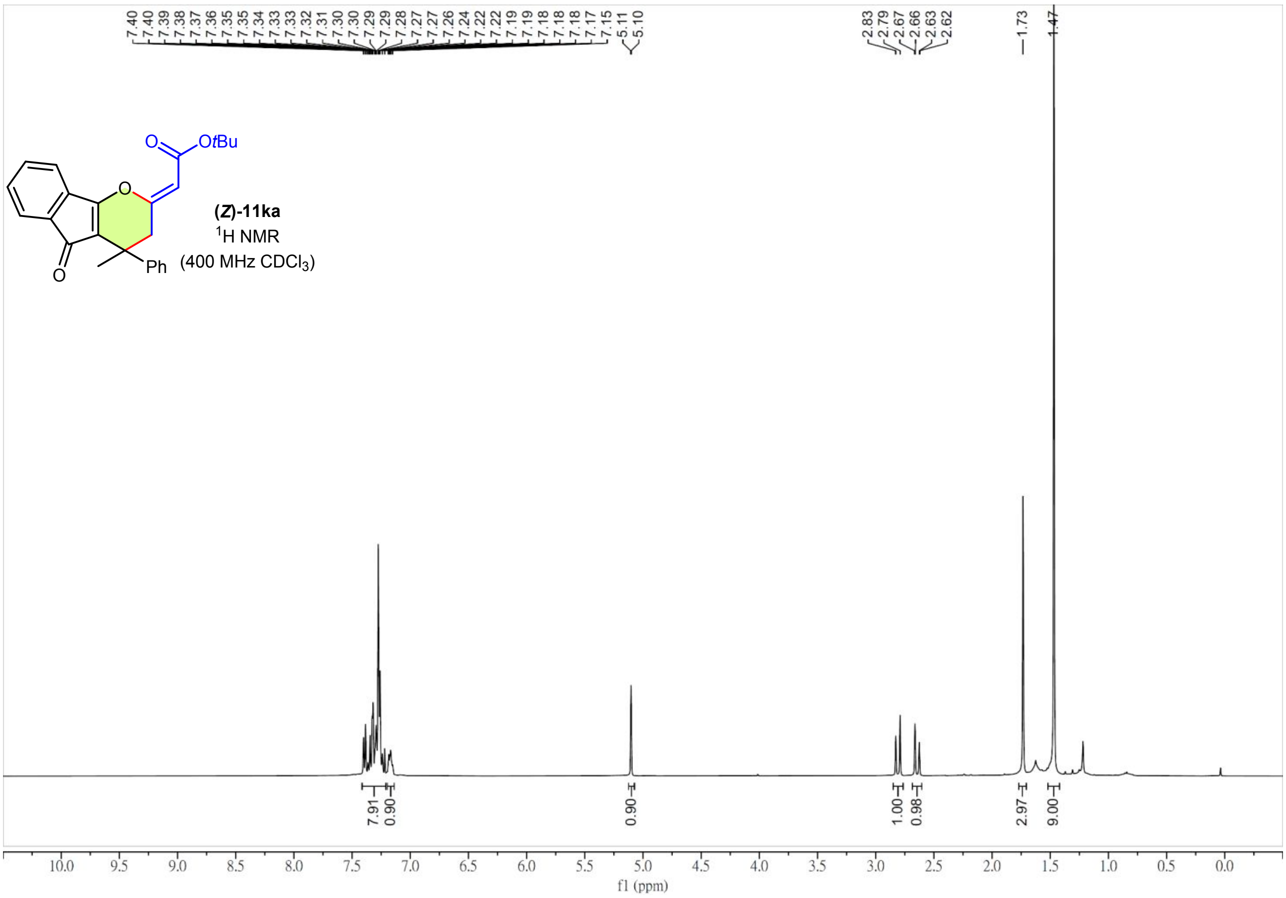
7.40 7.40 7.39 7.38 7.37 7.36 7.35 7.35 7.34 7.33 7.33 7.32 7.31 7.30 7.30 7.29 7.29 7.28 7.27 7.27 7.26 7.24 7.22 7.22 7.19 7.19 7.18 7.18 7.18 7.17 7.15 5.11 5.10
2.83 2.79 2.67 2.66 2.63 2.62
1.73
1.47
O
OtBu
O
O
Ph
(Z)-11ka
1H NMR
(400 MHz CDCl3)
7.91 0.90 0.90 1.00 0.98 2.97 9.00
10.0 9.5 9.0 8.5 8.0 7.5 7.0 6.5 6.0 5.5 5.0 4.5 4.0 3.5 3.0 2.5 2.0 1.5 1.0 0.5 0.0
f1 (ppm)

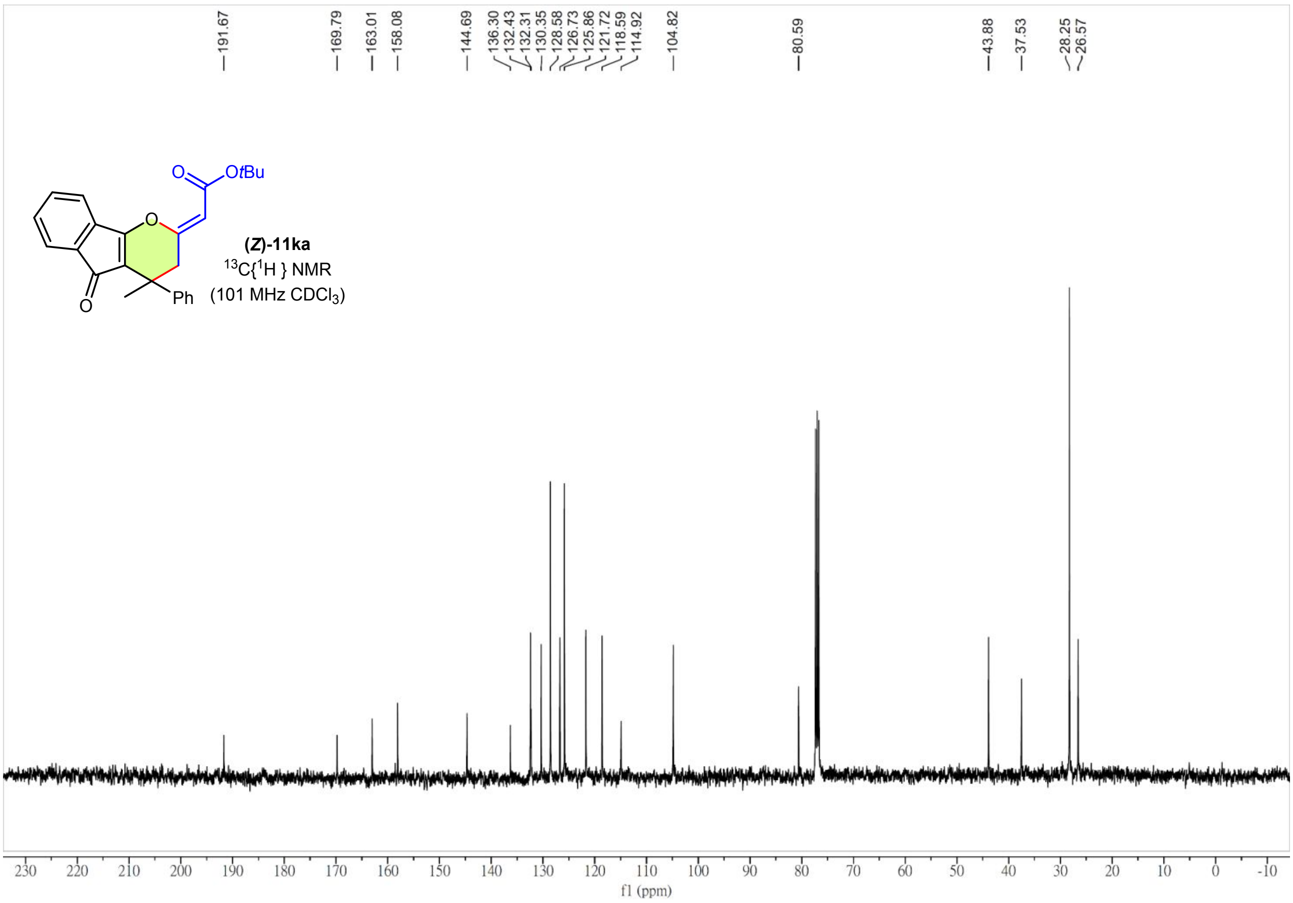
191.67
169.79
163.01
158.08
144.69
136.30 132.43 132.31 130.35 128.58 126.73 125.86 121.72 118.59 114.92
104.82
80.59
43.88
37.53
28.25 26.57
O
OtBu
O
O
Ph
(Z)-11ka
13C{1H } NMR
(101 MHz CDCl3)
230 220 210 200 190 180 170 160 150 140 130 120 110 100 90 80 70 60 50 40 30 20 10 0 -10
f1 (ppm)

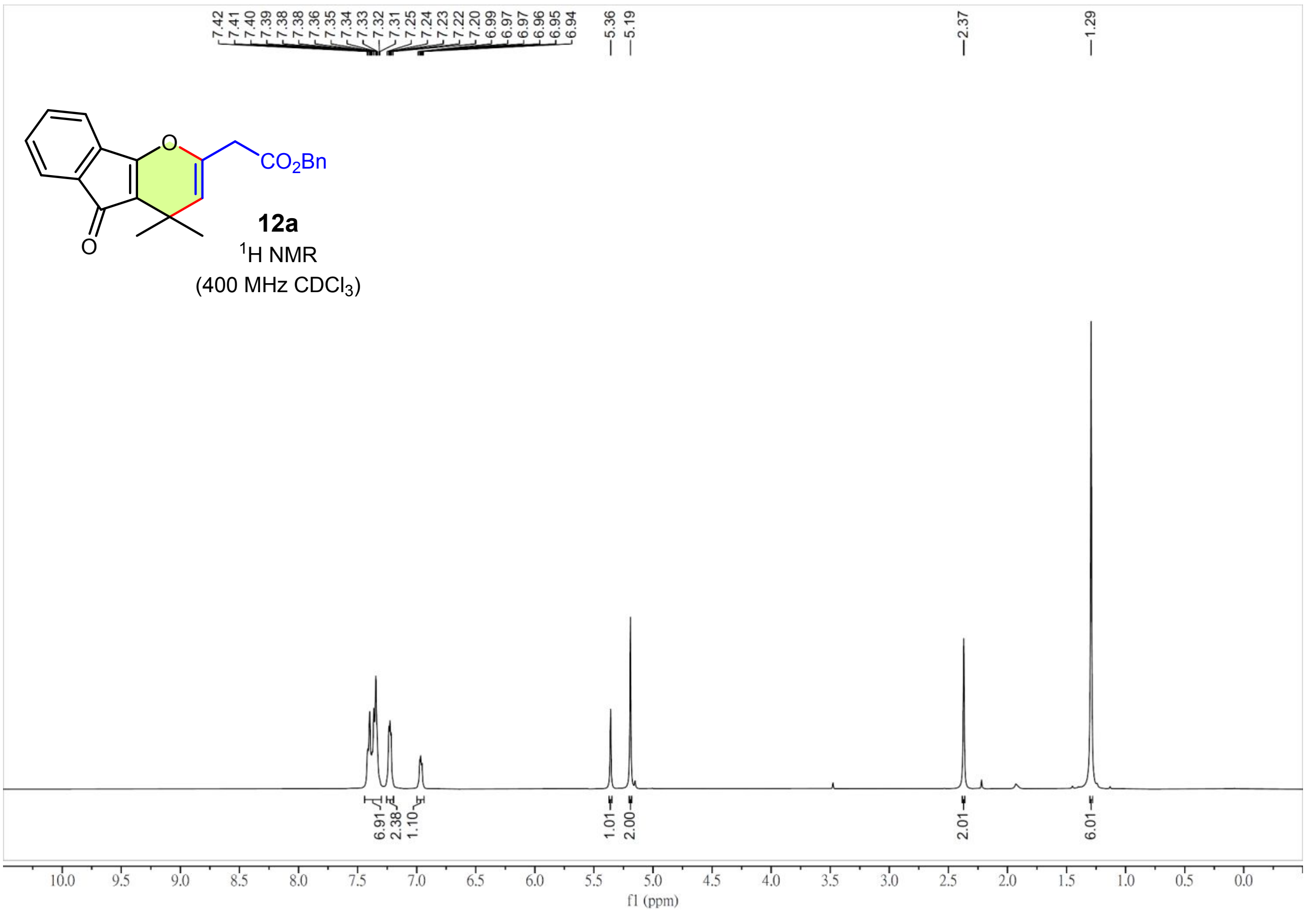
7.42
7.41
7.40
7.39
7.38
7.38
7.36
7.35
7.34
7.33
7.32
7.31
7.25
7.24
7.23
7.22
7.20
6.99
6.97
6.97
6.96
6.95
6.94
5.36
5.19
2.37
1.29
CO2Bn
O
O
12a
1H NMR
(400 MHz CDCl3)
6.91
2.38
1.10
1.01
2.00
2.01
6.01
f1 (ppm)

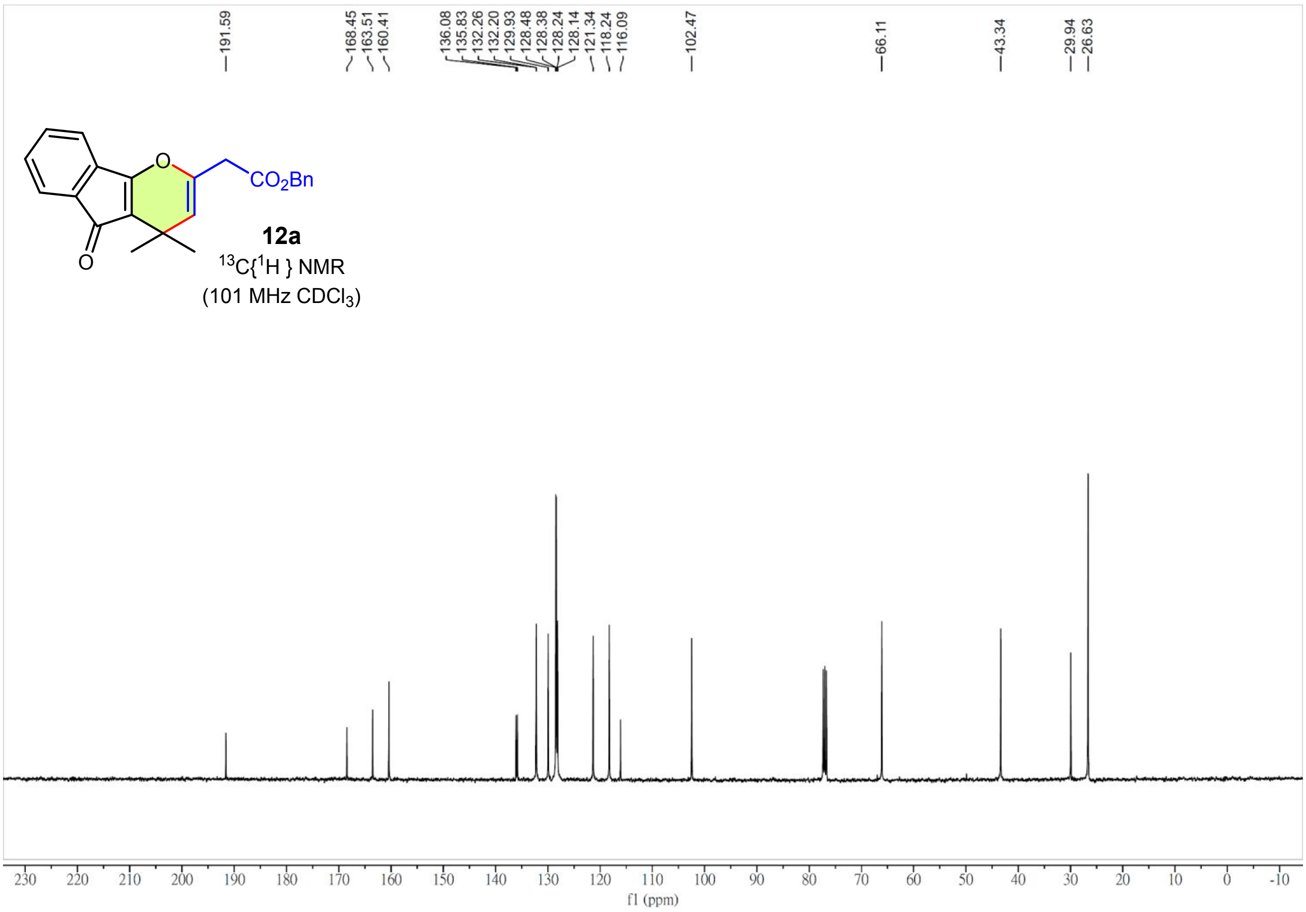
191.59
168.45
163.51
160.41
136.08
135.83
132.26
132.20
129.93
128.48
128.38
128.24
128.14
121.34
118.24
116.09
102.47
66.11
43.34
29.94
26.63
CO2Bn
O
O
12a
13C{1H } NMR
(101 MHz CDCl3)
f1 (ppm)

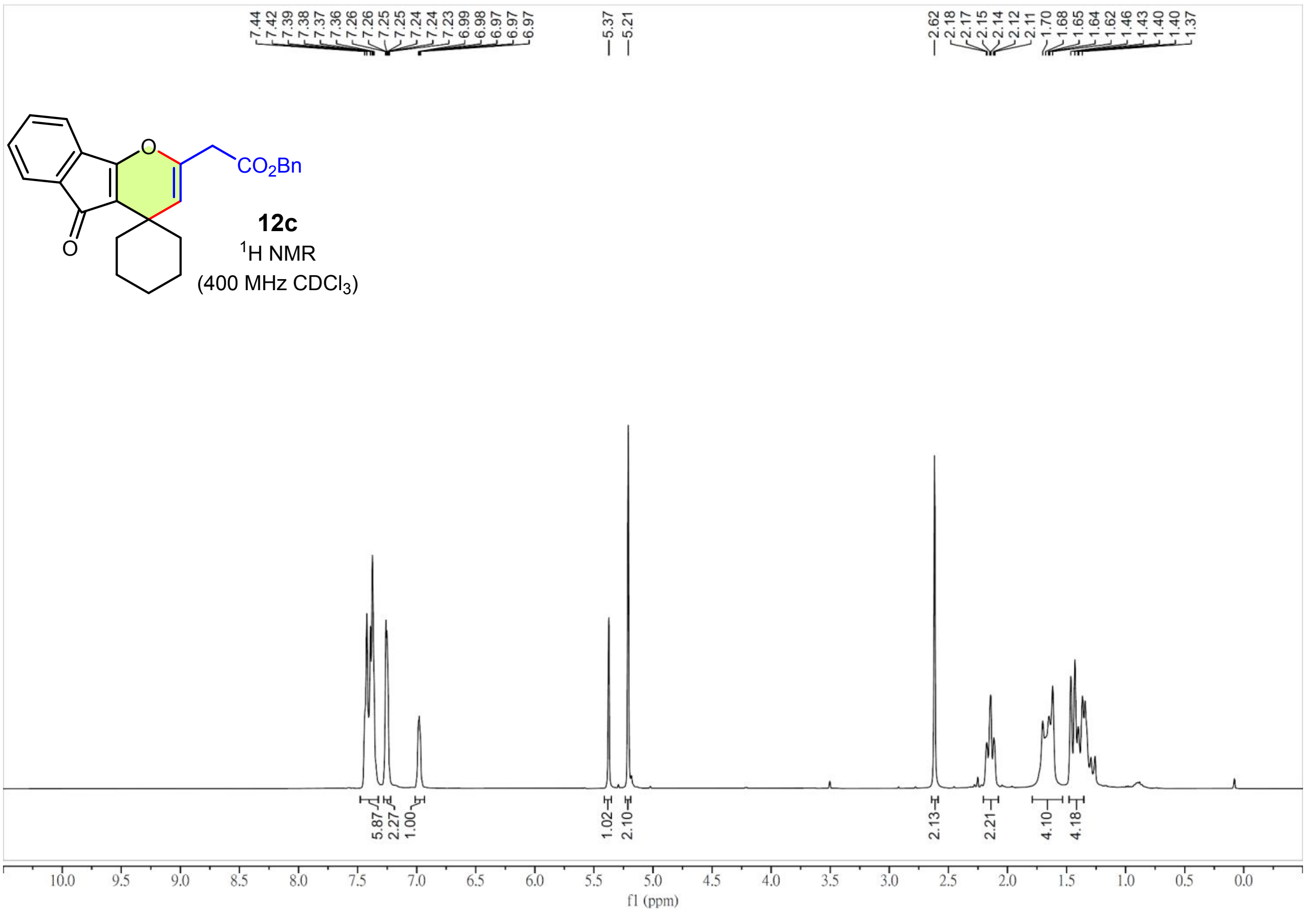
7.44
7.42
7.39
7.38
7.37
7.36
7.26
7.25
7.25
7.24
7.24
7.23
6.99
6.98
6.97
6.97
5.37
5.21
2.62
2.18
2.17
2.15
2.14
2.12
2.11
1.70
1.68
1.65
1.64
1.62
1.46
1.43
1.40
1.40
1.37
CO2Bn
O
O
12c
1H NMR
(400 MHz CDCl3)
5.87
2.27
1.00
1.02
2.10
2.13
2.21
4.10
4.18
10.0
9.5
9.0
8.5
8.0
7.5
7.0
6.5
6.0
5.5
5.0
4.5
4.0
3.5
3.0
2.5
2.0
1.5
1.0
0.5
0.0
f1 (ppm)

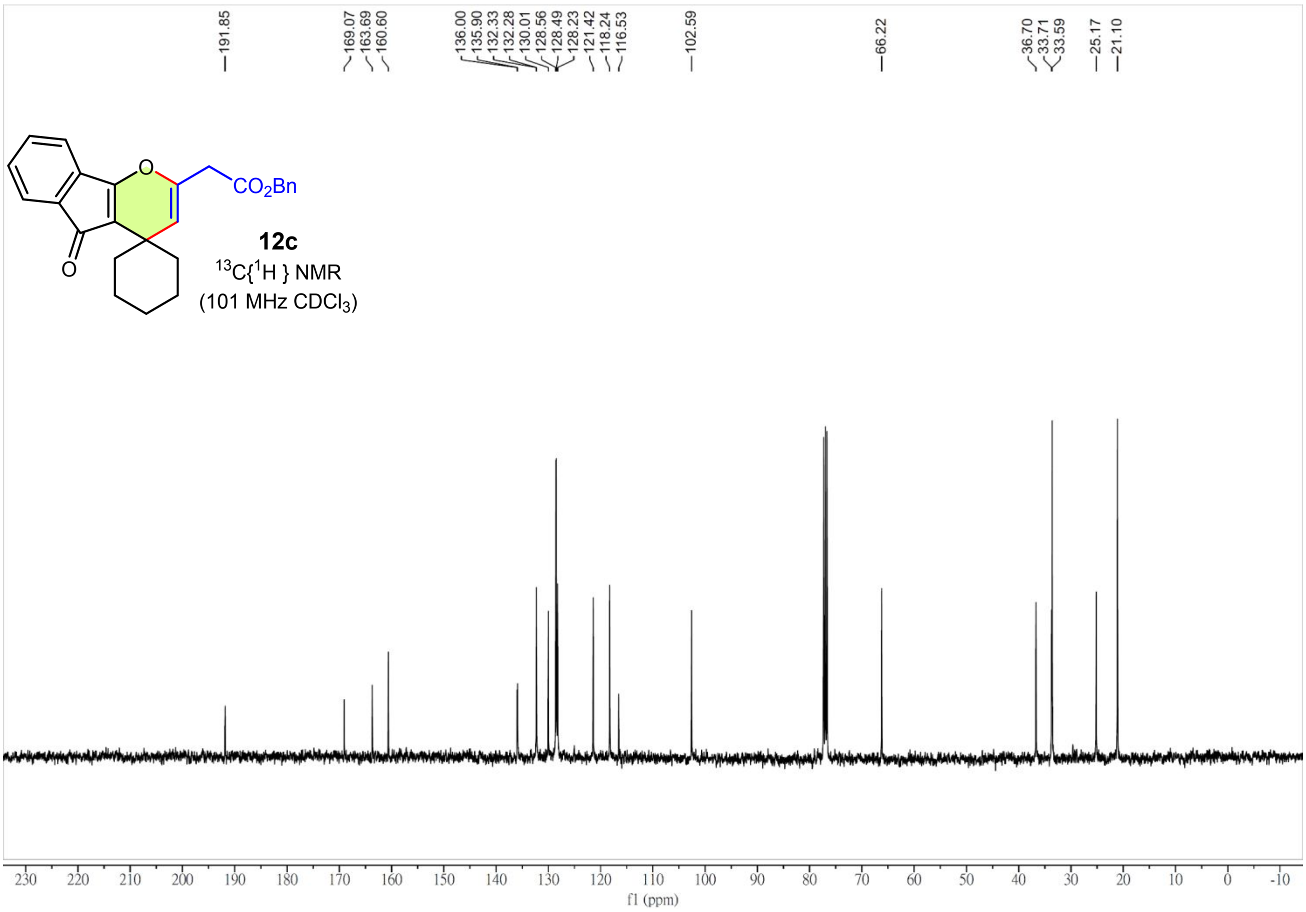
191.85
169.07
163.69
160.60
136.00
135.90
132.33
132.28
130.01
128.56
128.49
128.23
121.42
118.24
116.53
102.59
66.22
36.70
33.71
33.59
25.17
21.10
CO2Bn
O
O
12c
13C{1H } NMR
(101 MHz CDCl3)
230
220
210
200
190
180
170
160
150
140
130
120
110
100
90
80
70
60
50
40
30
20
10
0
-10
f1 (ppm)

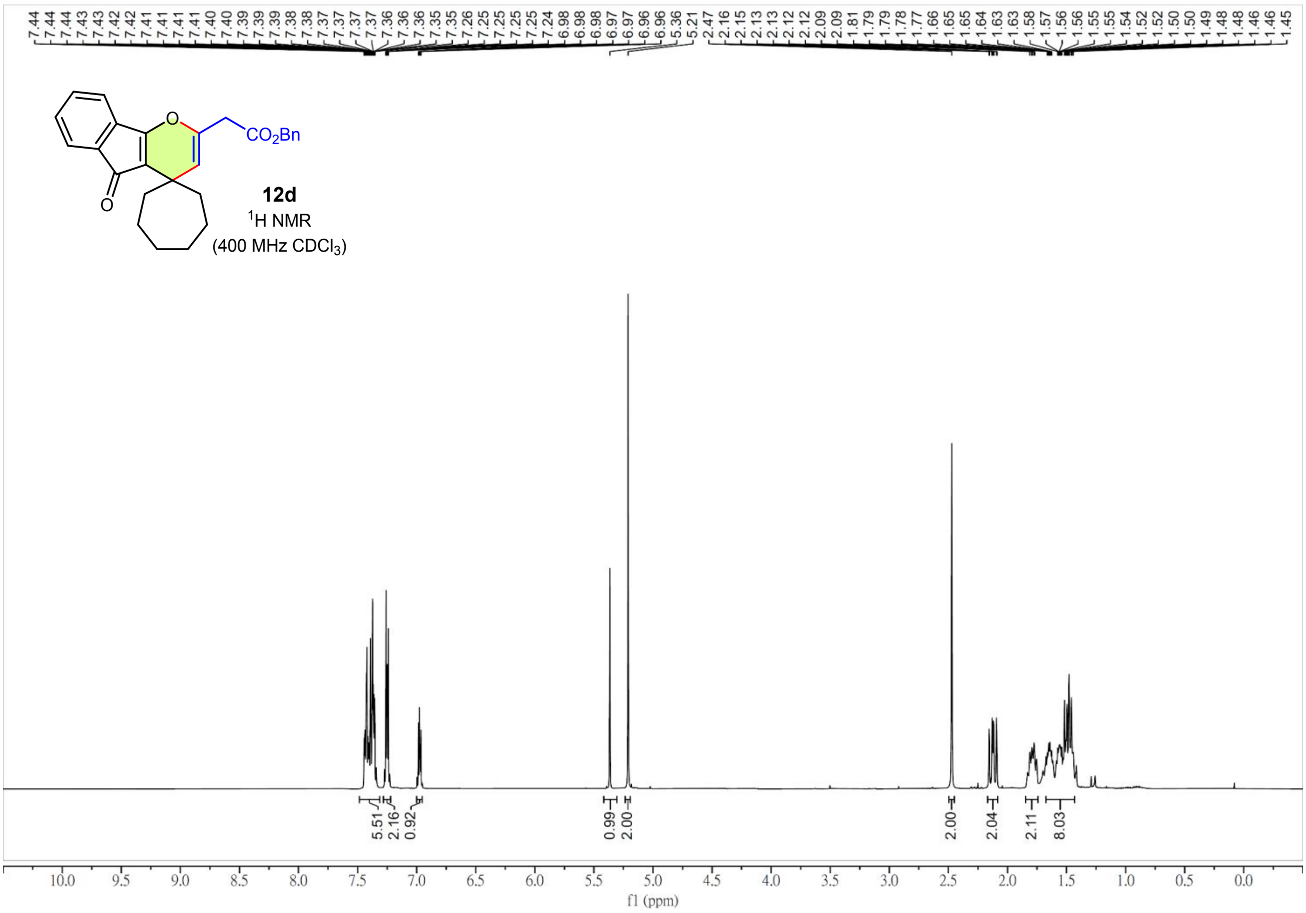
12d
1H NMR
(400 MHz CDCl3)
f1 (ppm)

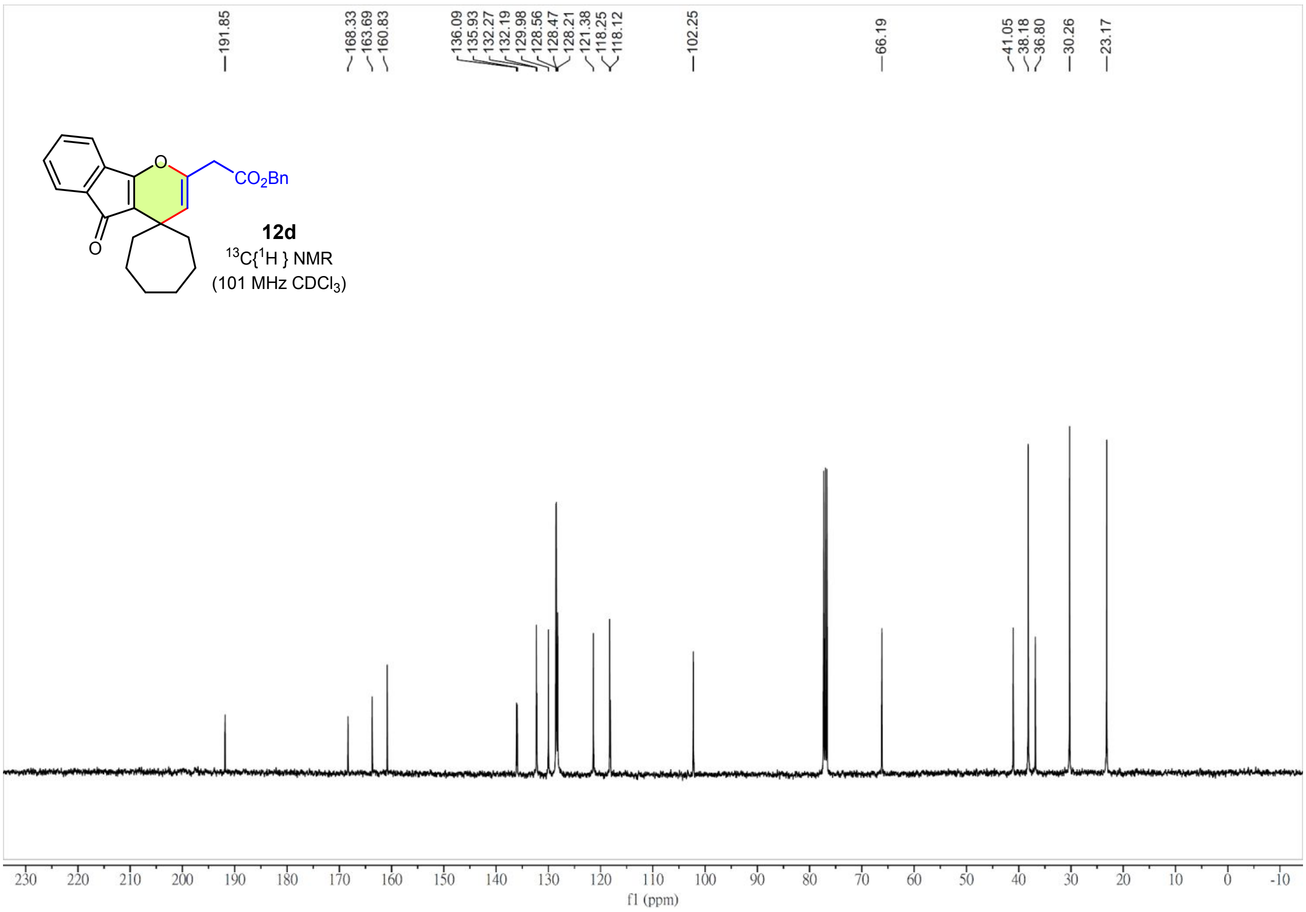
12d
13C{1H } NMR
(101 MHz CDCl3)
f1 (ppm)

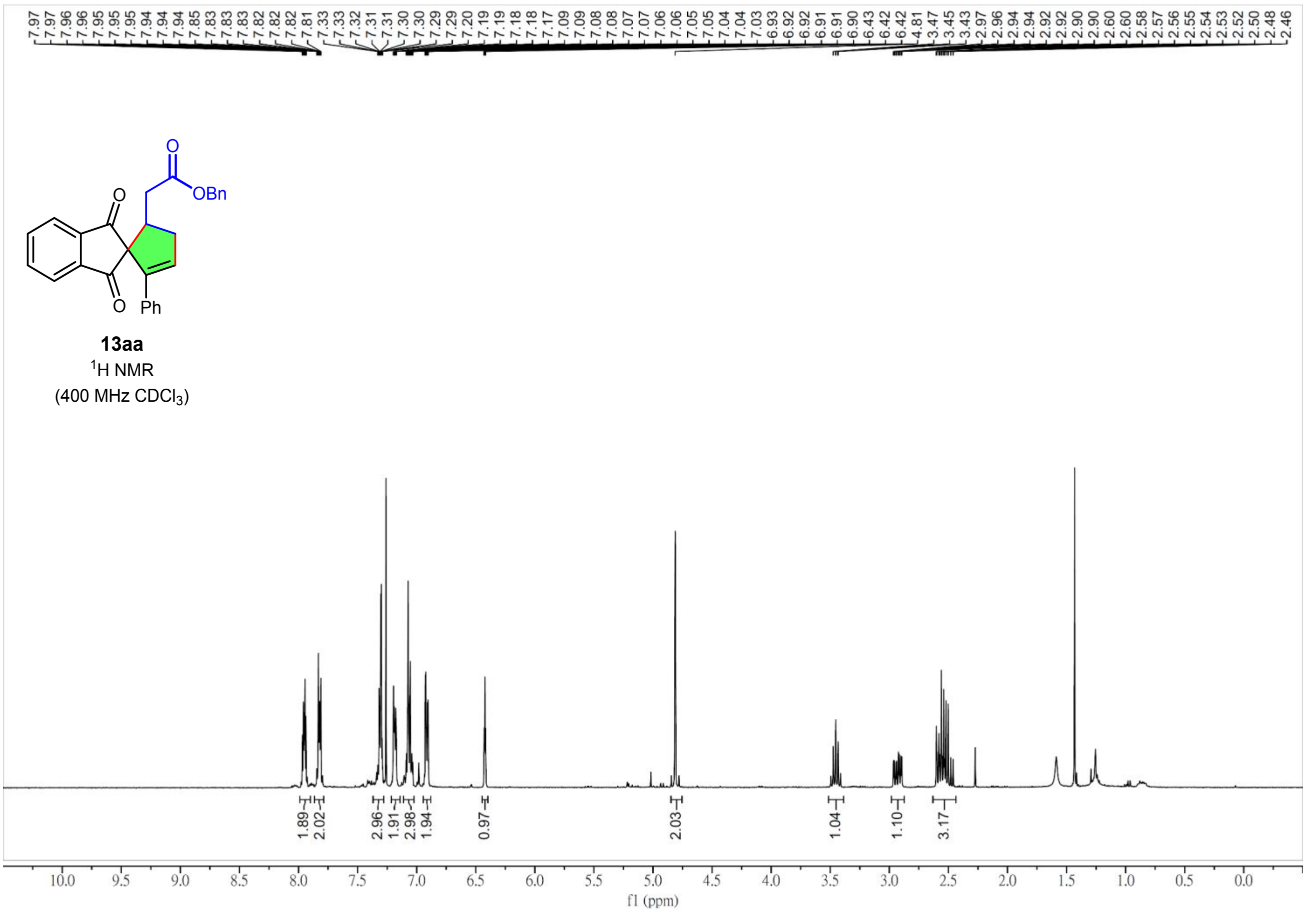
13aa
^{1}H NMR
(400 MHz CDCl$_3$)
f1 (ppm)

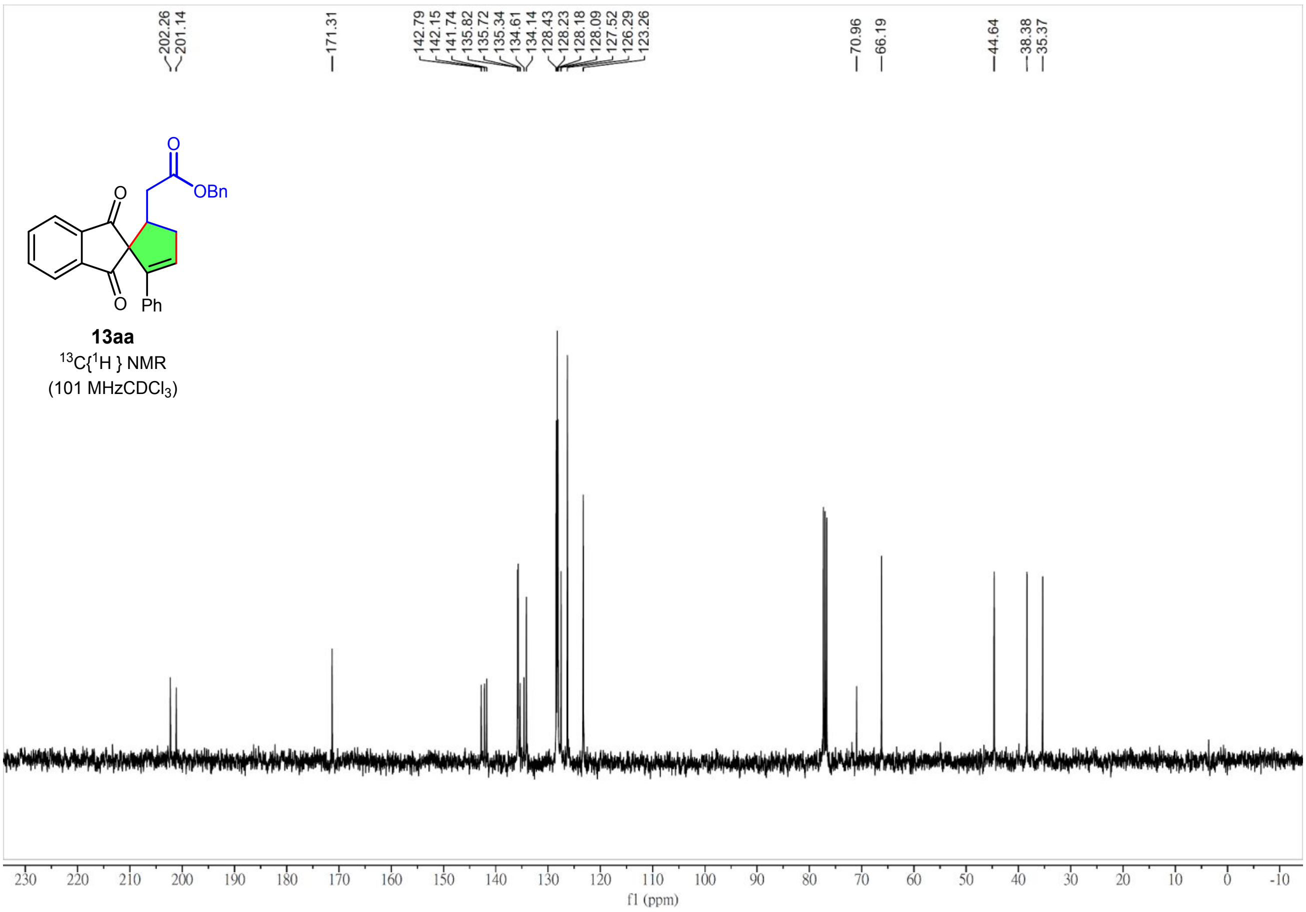
13aa
^{13}C{^{1}H } NMR
(101 MHzCDCl$_3$)
f1 (ppm)

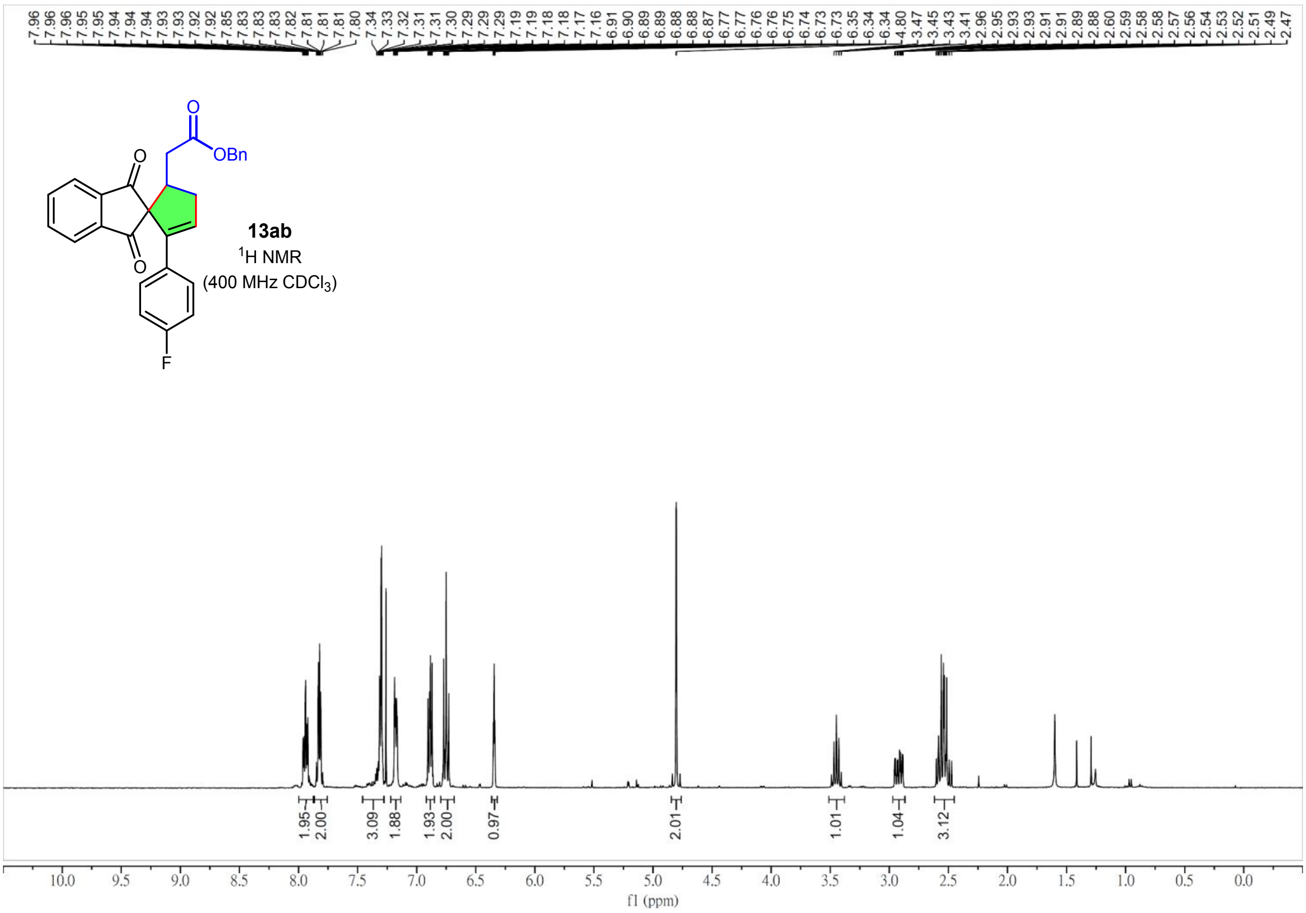
O
OBn
O
O
F
13ab
1H NMR
(400 MHz CDCl3)
1.95
2.00
3.09
1.88
1.93
2.00
0.97
2.01
1.01
1.04
3.12
f1 (ppm)

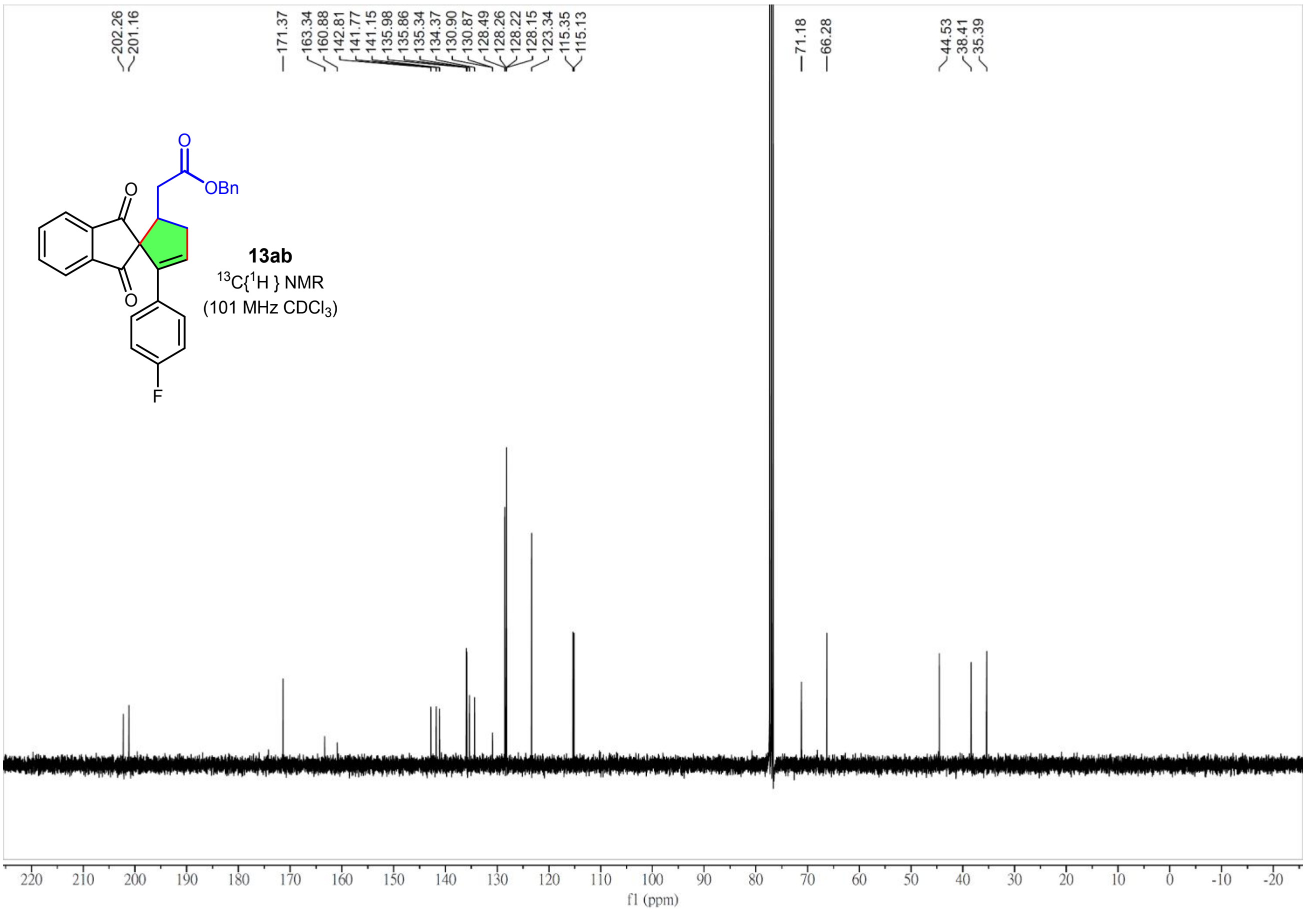
202.26
201.16
171.37
163.34
160.88
142.81
141.77
141.15
135.98
135.86
135.34
134.37
130.90
130.87
128.49
128.26
128.22
128.15
123.34
115.35
115.13
71.18
66.28
44.53
38.41
35.39
O
OBn
O
O
F
13ab
13C{1H } NMR
(101 MHz CDCl3)
f1 (ppm)

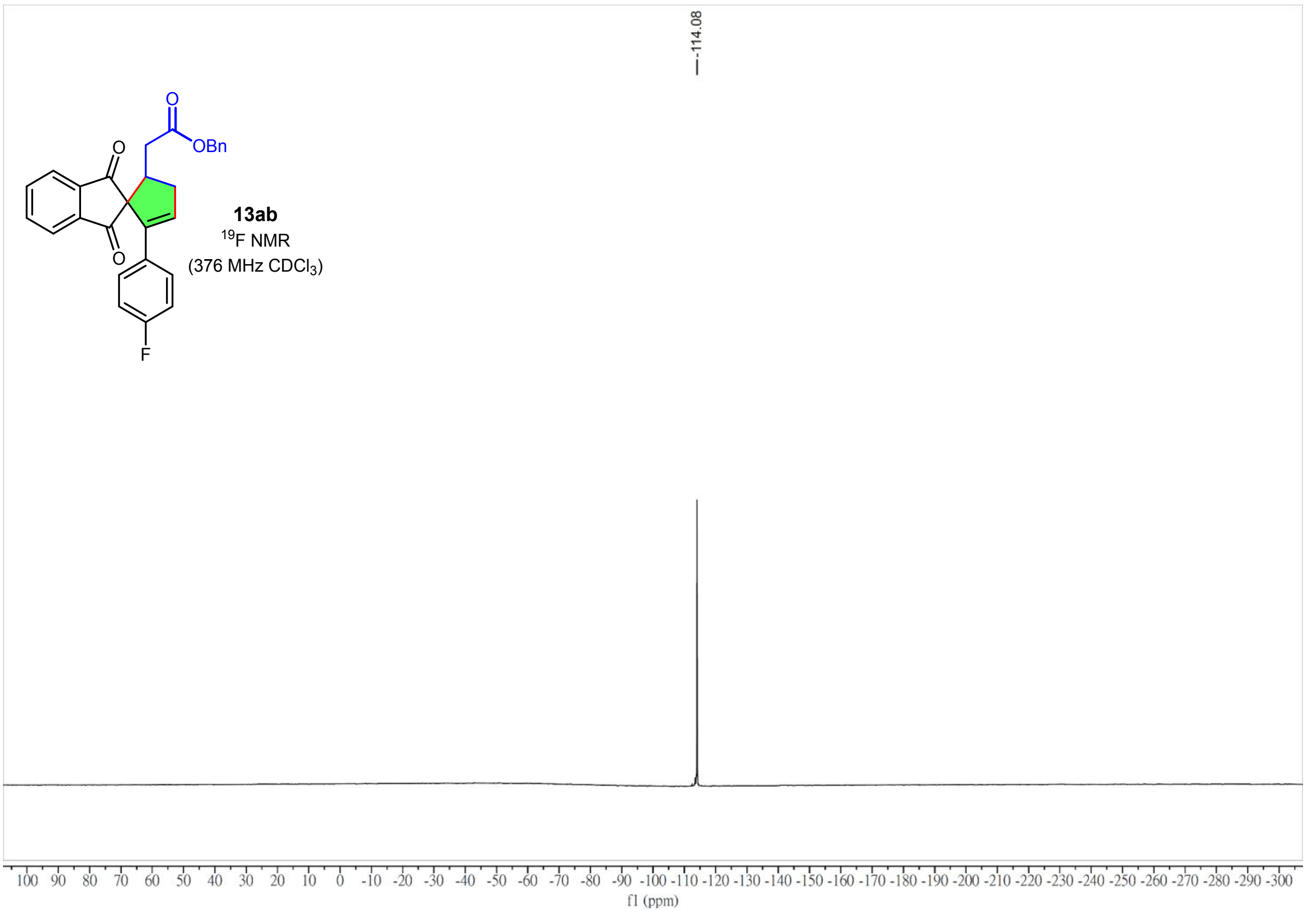
-114.08
O
OBn
O
O
F
13ab
^{19}F NMR
(376 MHz CDCl$_3$)
100 90 80 70 60 50 40 30 20 10 0 -10 -20 -30 -40 -50 -60 -70 -80 -90 -100 -110 -120 -130 -140 -150 -160 -170 -180 -190 -200 -210 -220 -230 -240 -250 -260 -270 -280 -290 -300
f1 (ppm)

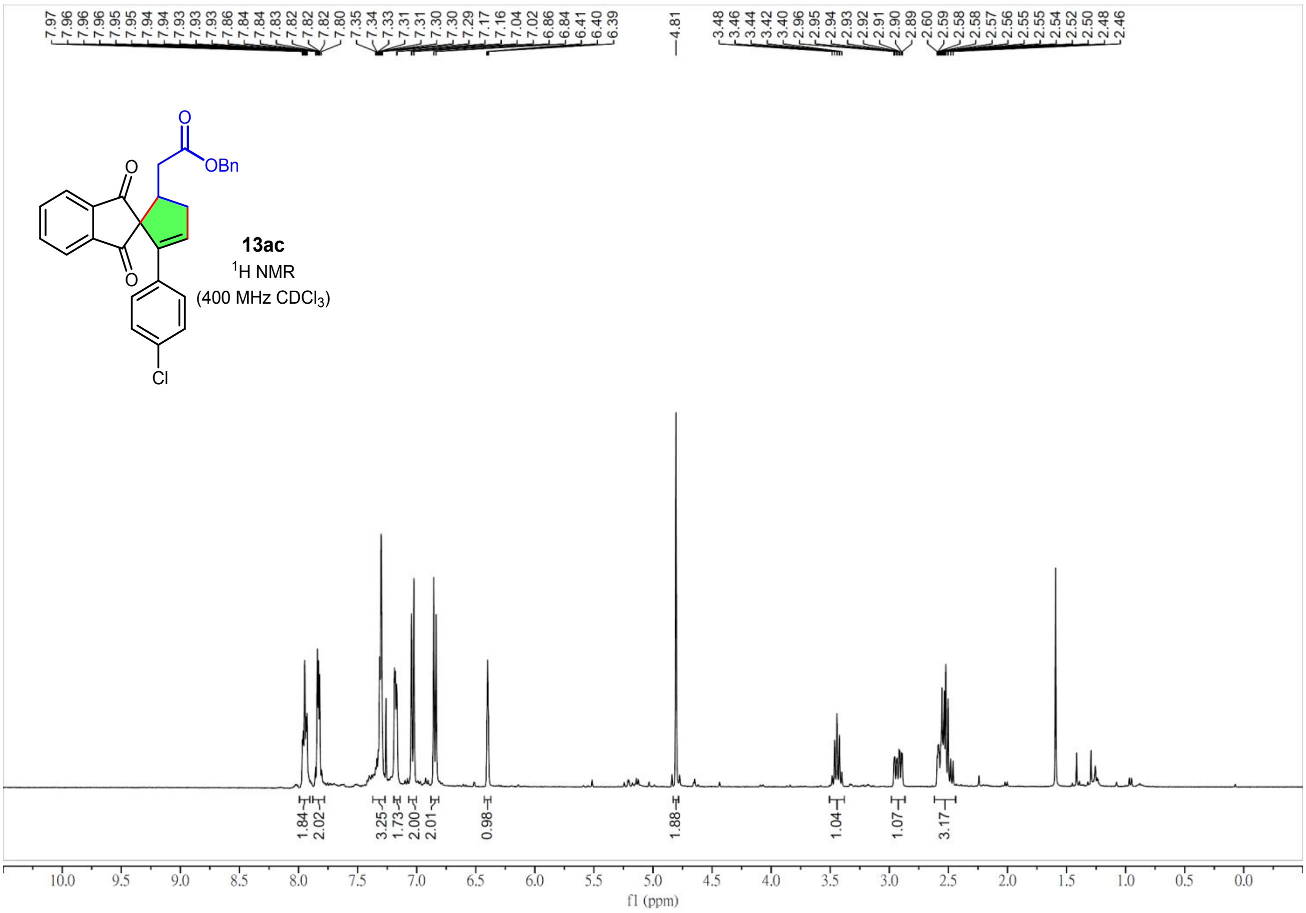
13ac
^{1}H NMR
(400 MHz CDCl$_3$)
f1 (ppm)

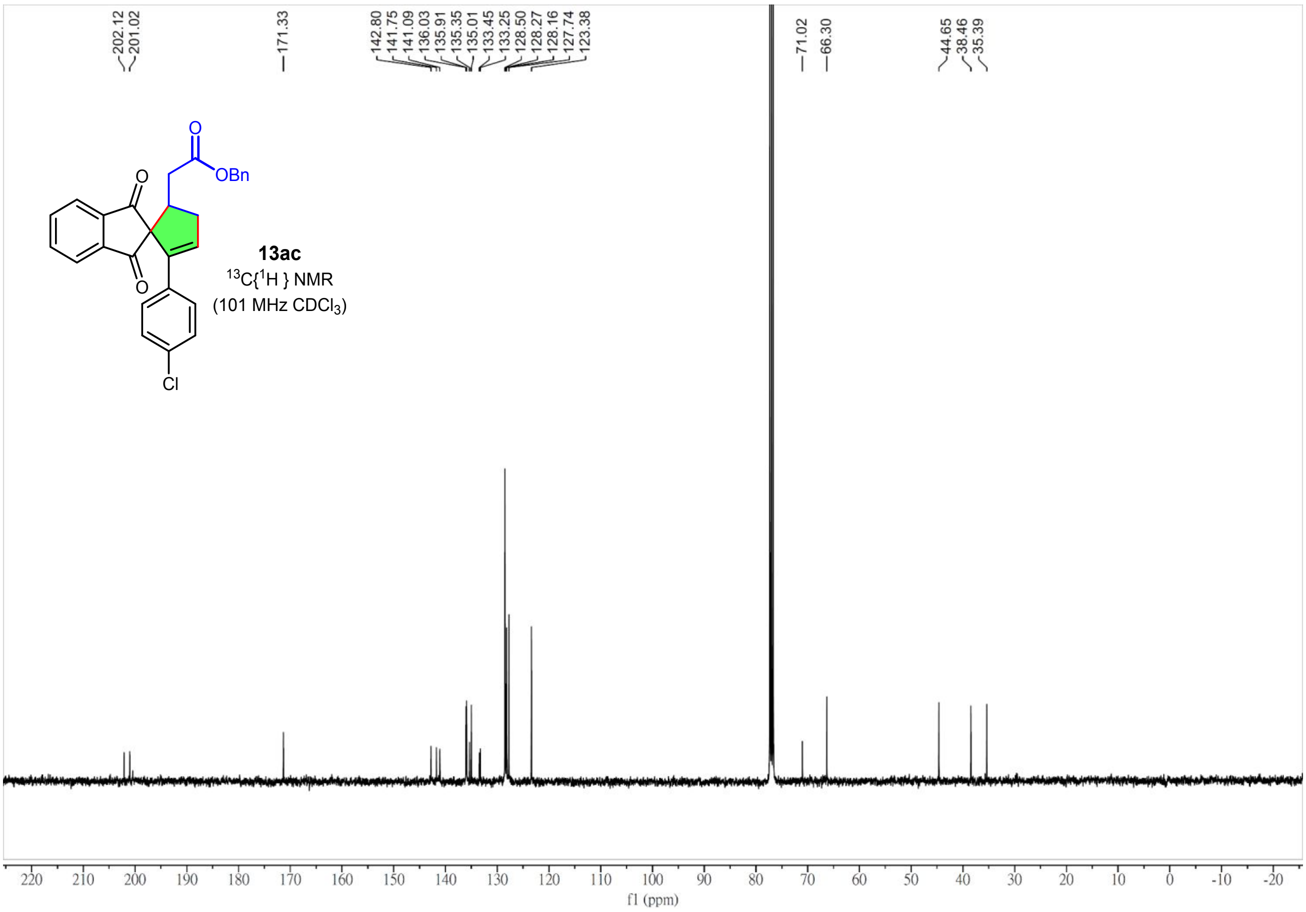
13ac
^{13}C{^{1}H } NMR
(101 MHz CDCl$_3$)
f1 (ppm)

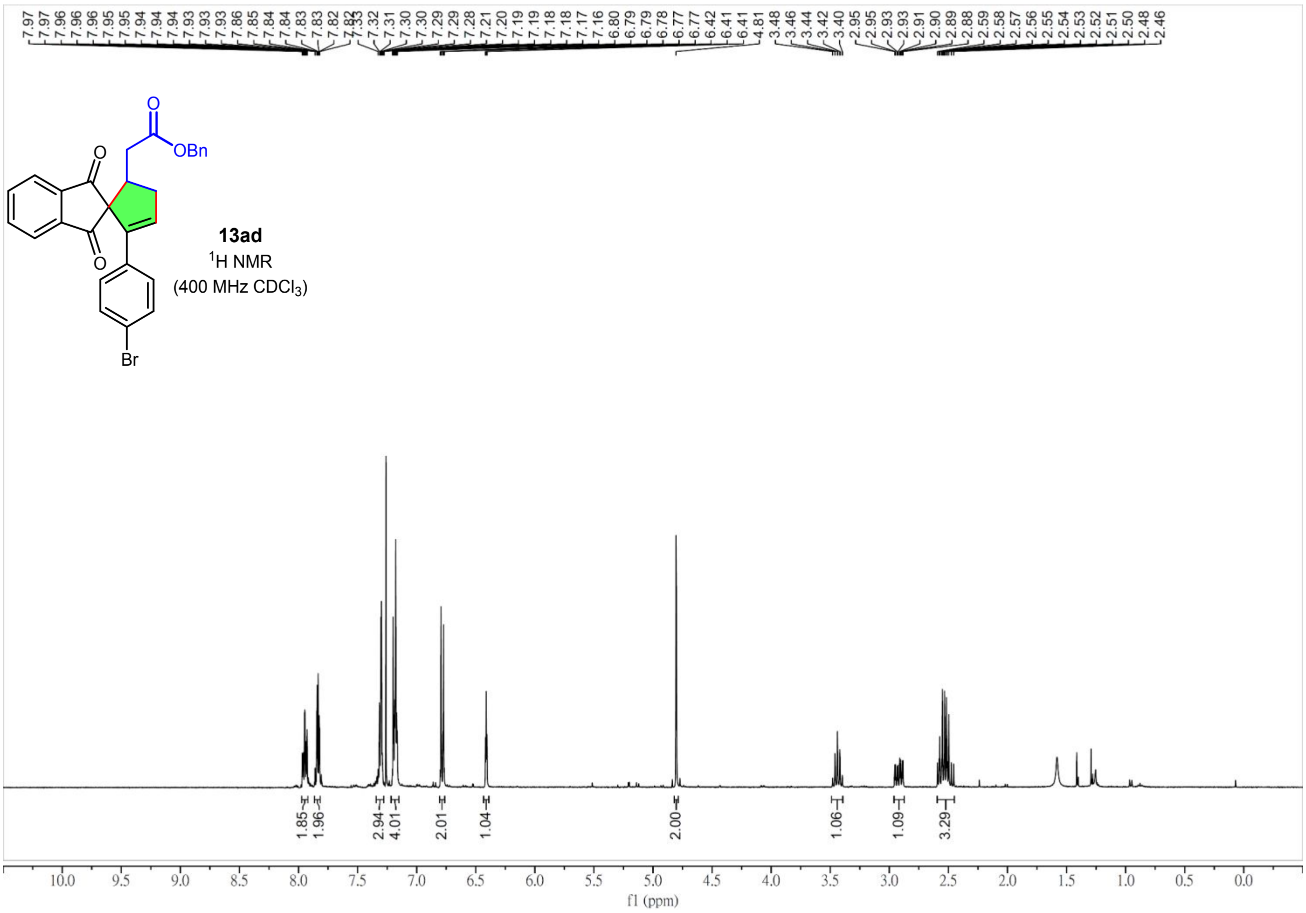
13ad
1H NMR
(400 MHz CDCl3)
OBn
Br
f1 (ppm)

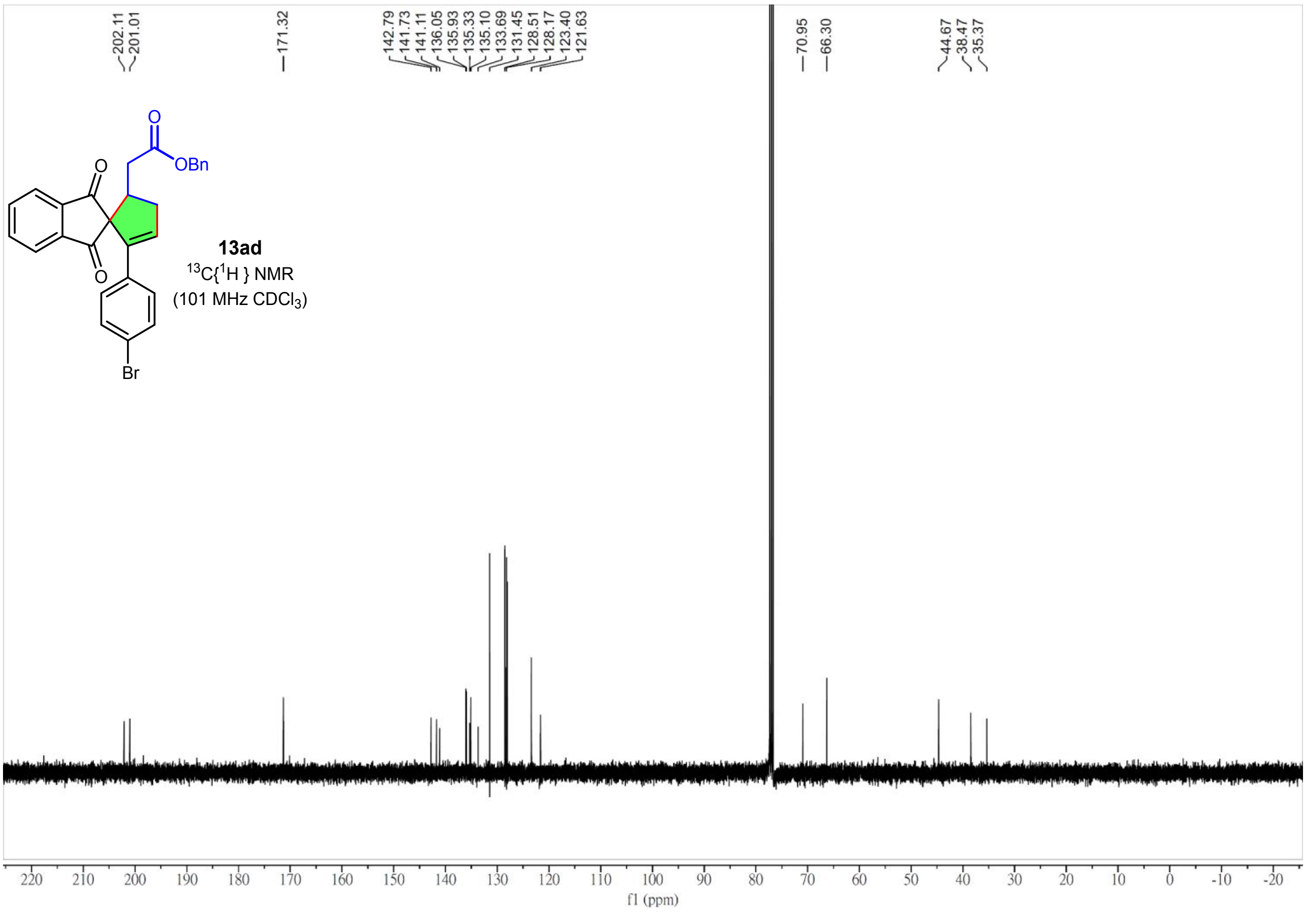
13ad
13C{1H } NMR
(101 MHz CDCl3)
OBn
Br
f1 (ppm)

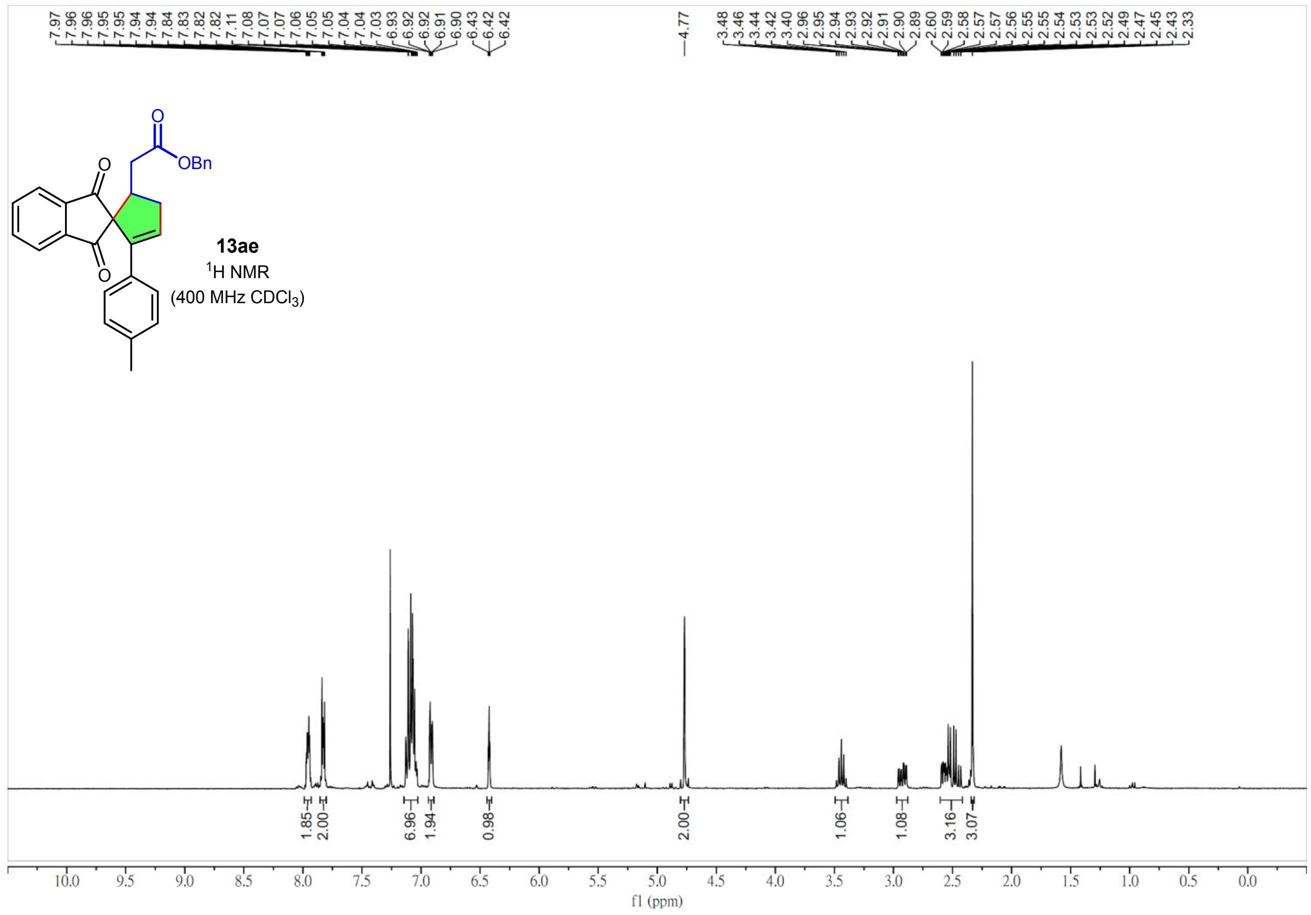

13ae
^{1}H NMR
(400 MHz CDCl$_3$)
OBn
f1 (ppm)

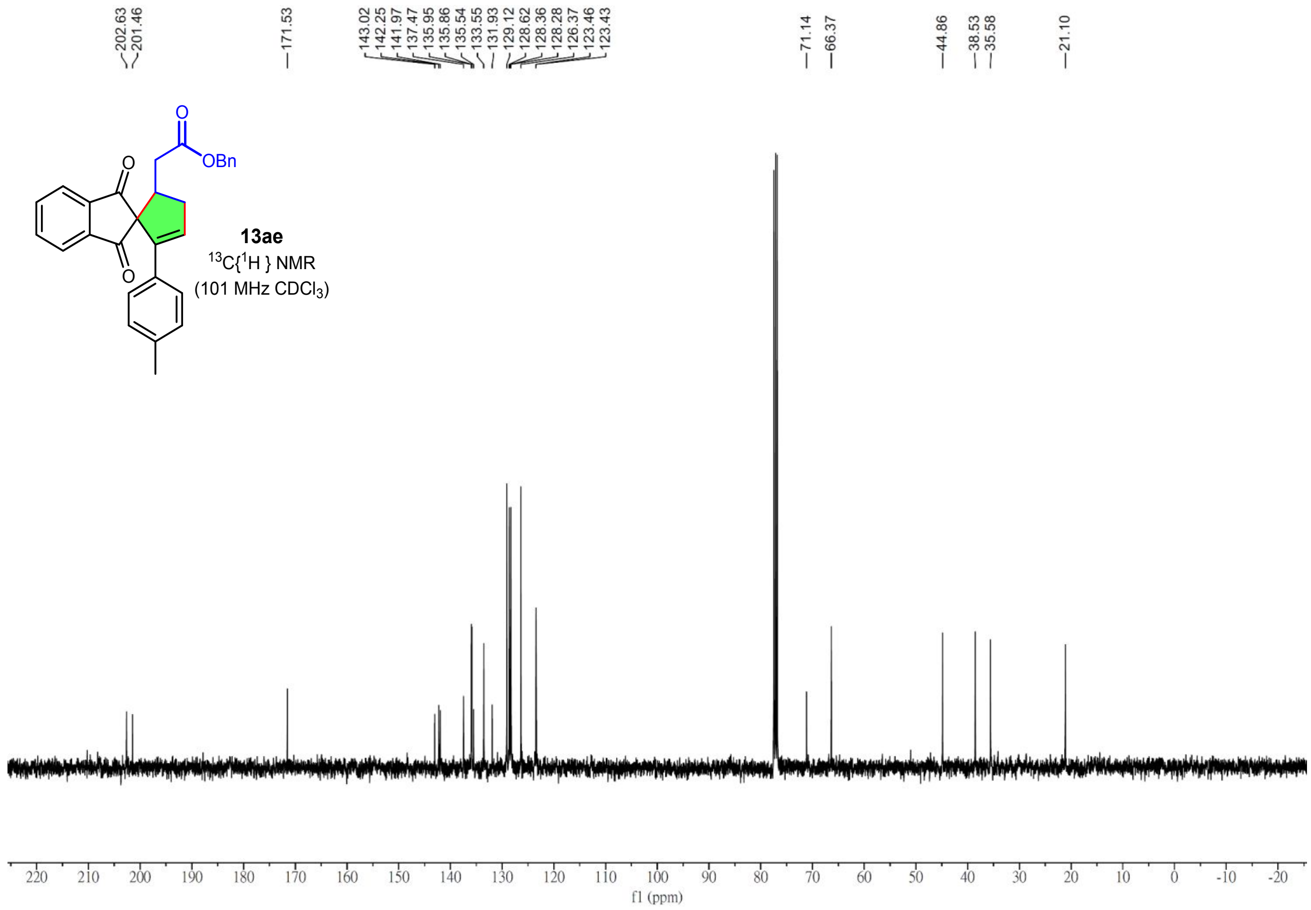

13ae
^{13}C{^{1}H } NMR
(101 MHz CDCl$_3$)
OBn
f1 (ppm)

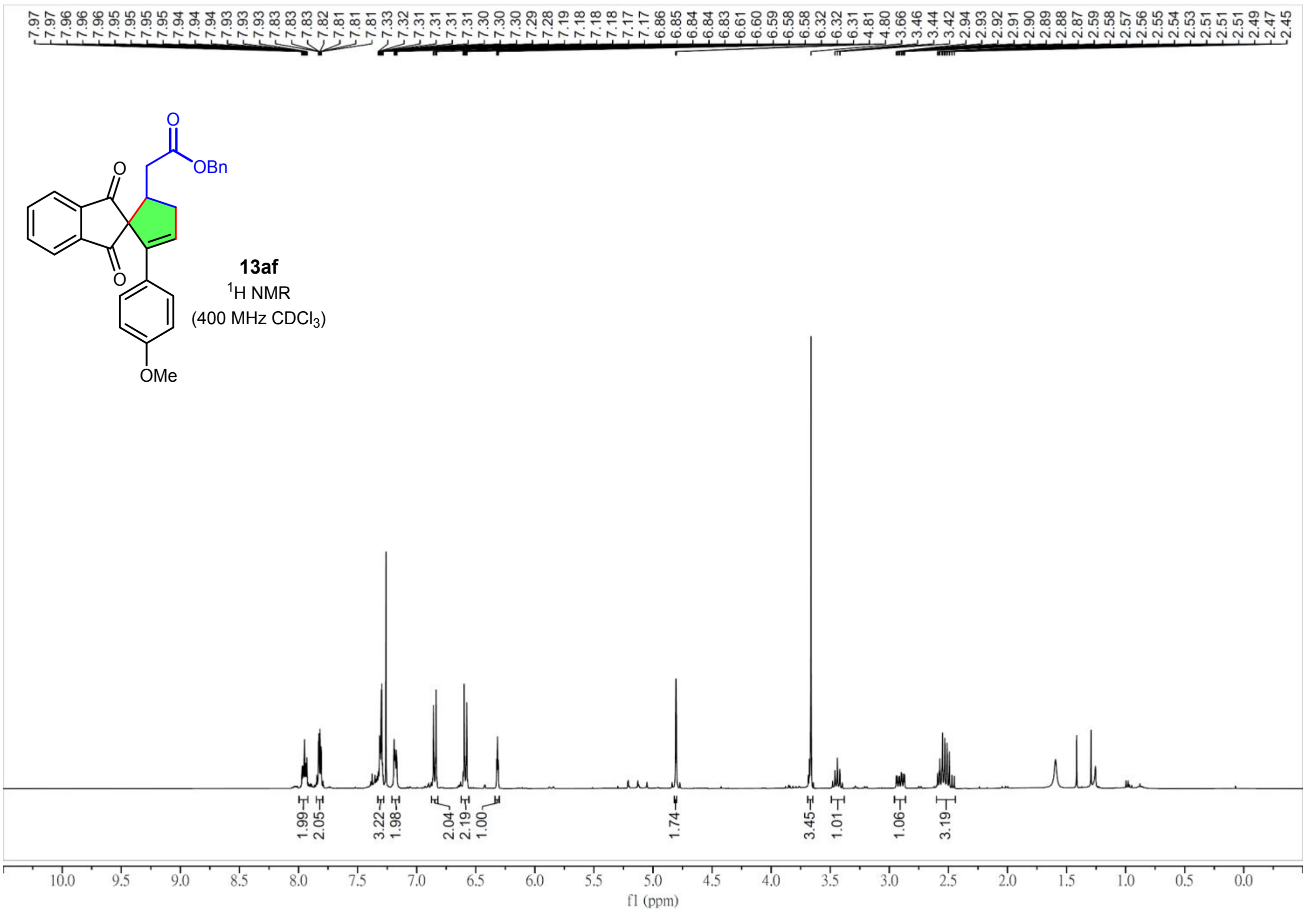
O
OBn
O
O
OMe
13af
1H NMR
(400 MHz CDCl3)
f1 (ppm)

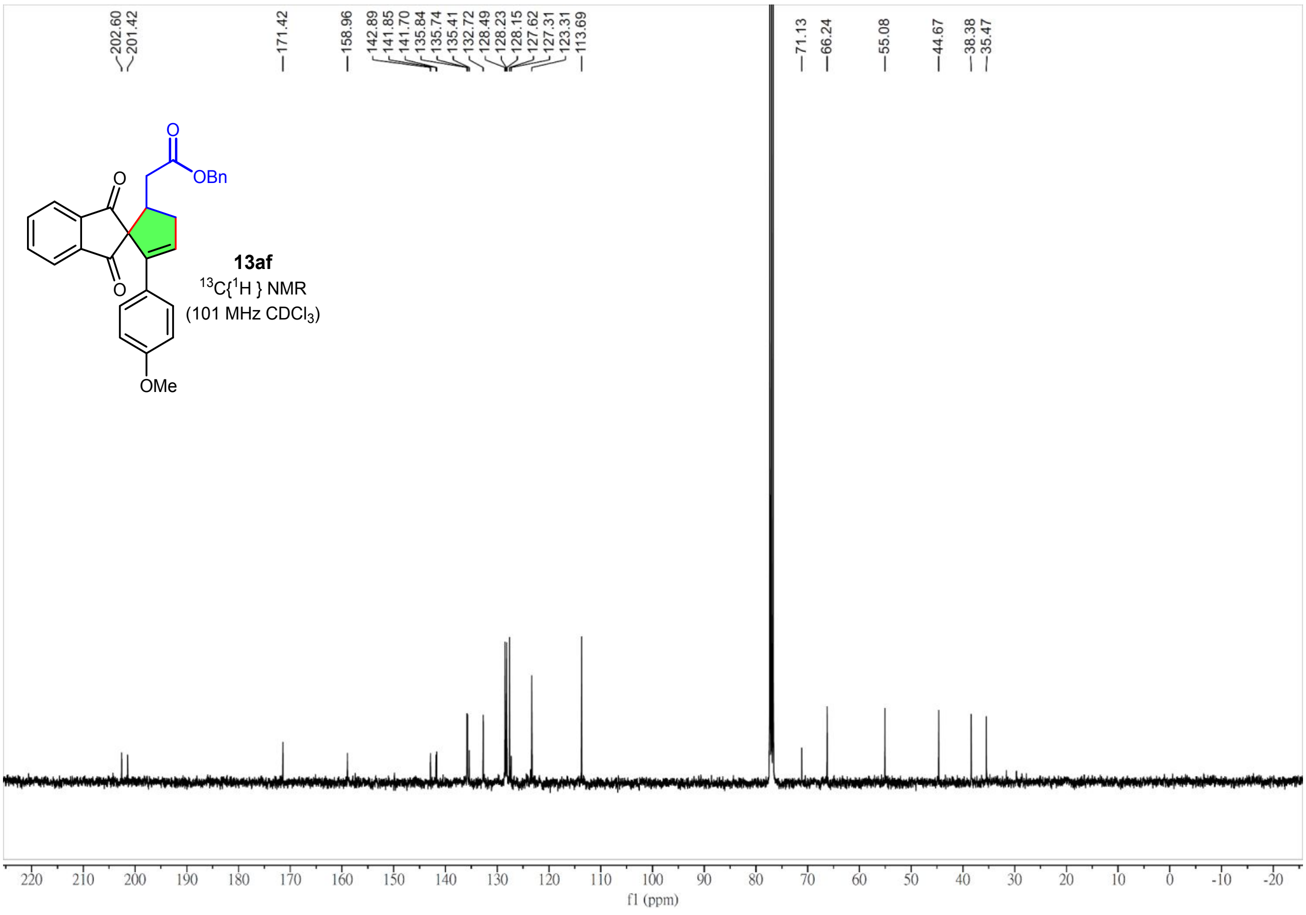
O
OBn
O
O
OMe
13af
13C{1H } NMR
(101 MHz CDCl3)
f1 (ppm)

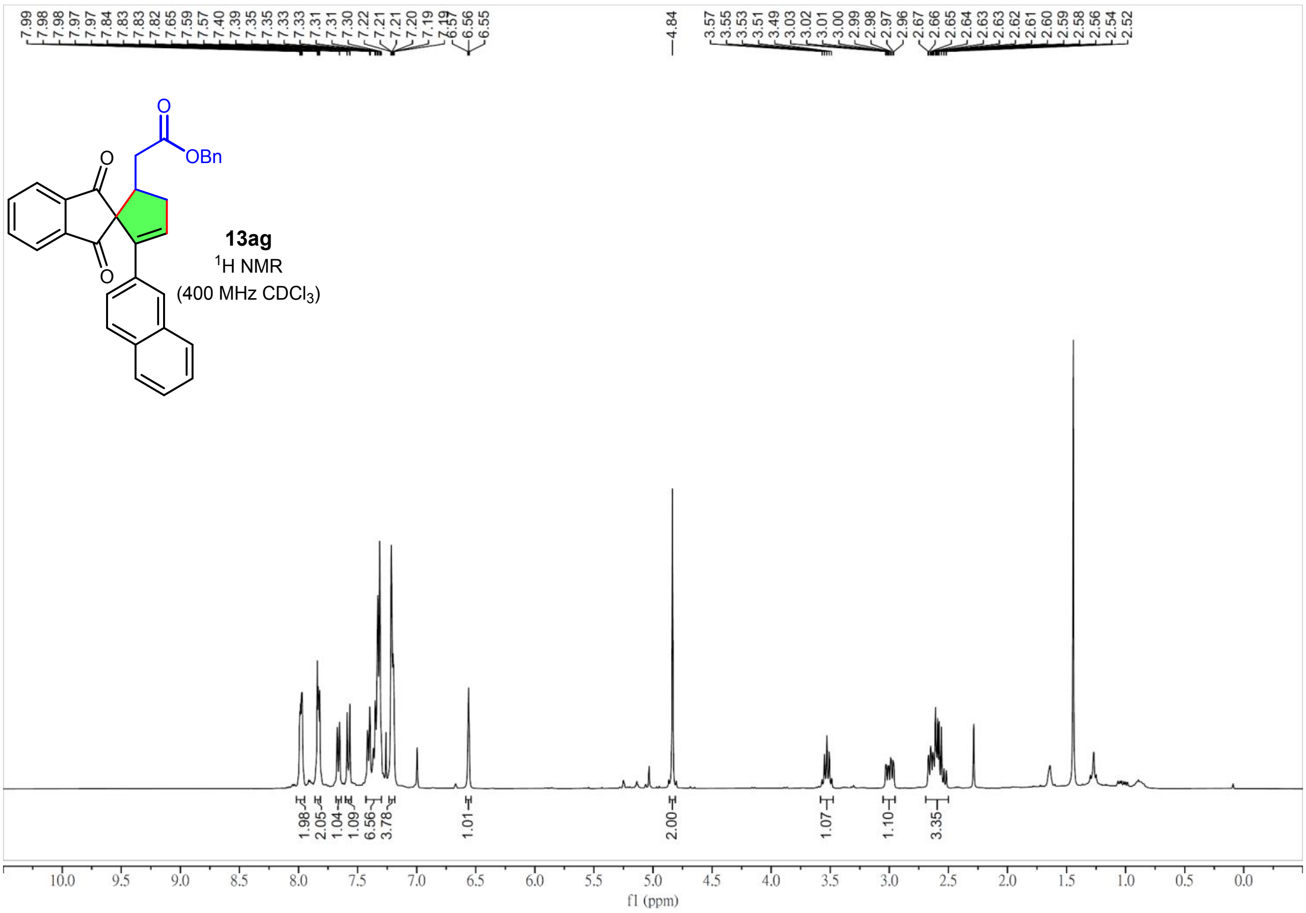
13ag
^{1}H NMR
(400 MHz CDCl$_3$)
f1 (ppm)

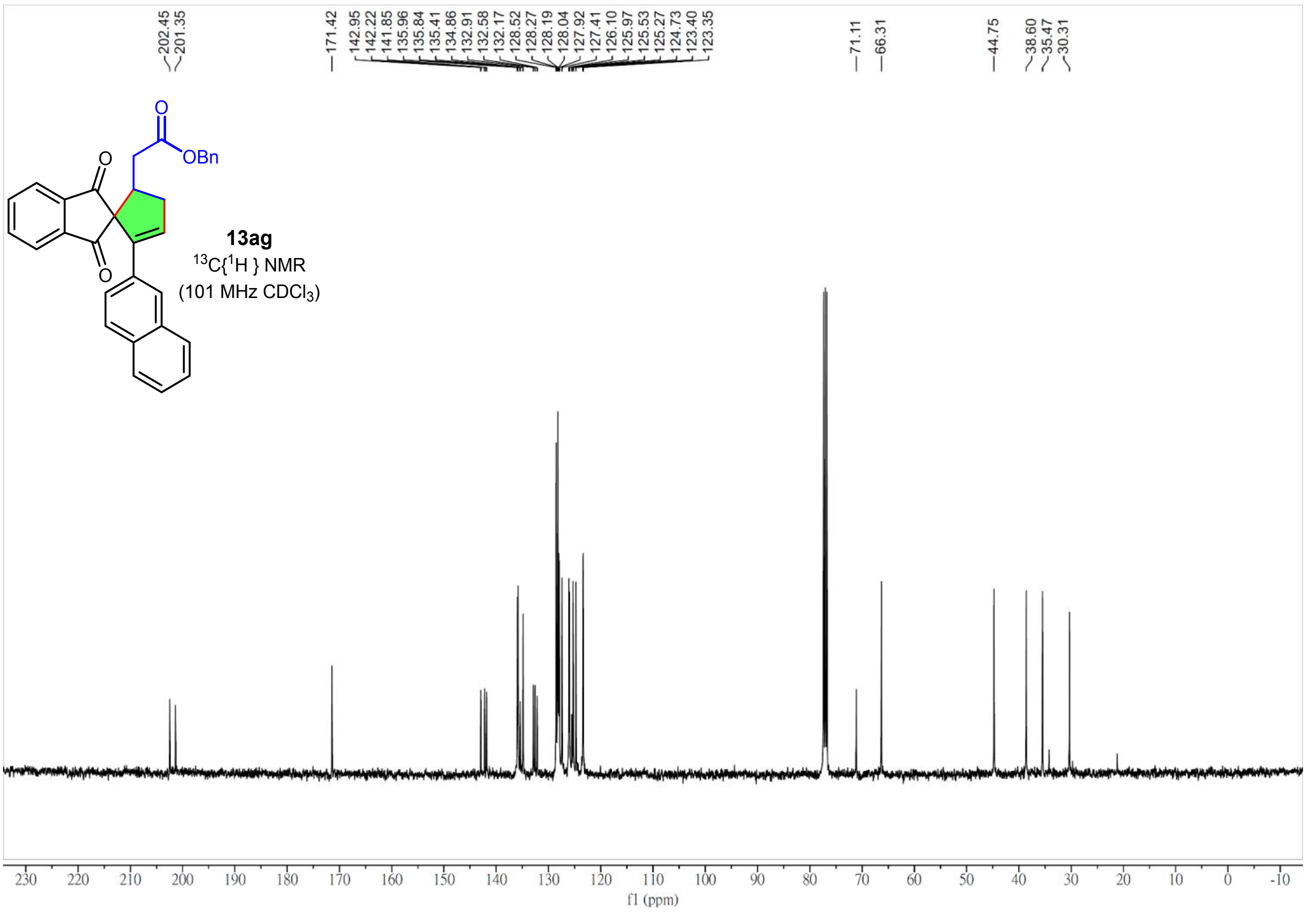
13ag
^{13}C{^{1}H } NMR
(101 MHz CDCl$_3$)
f1 (ppm)

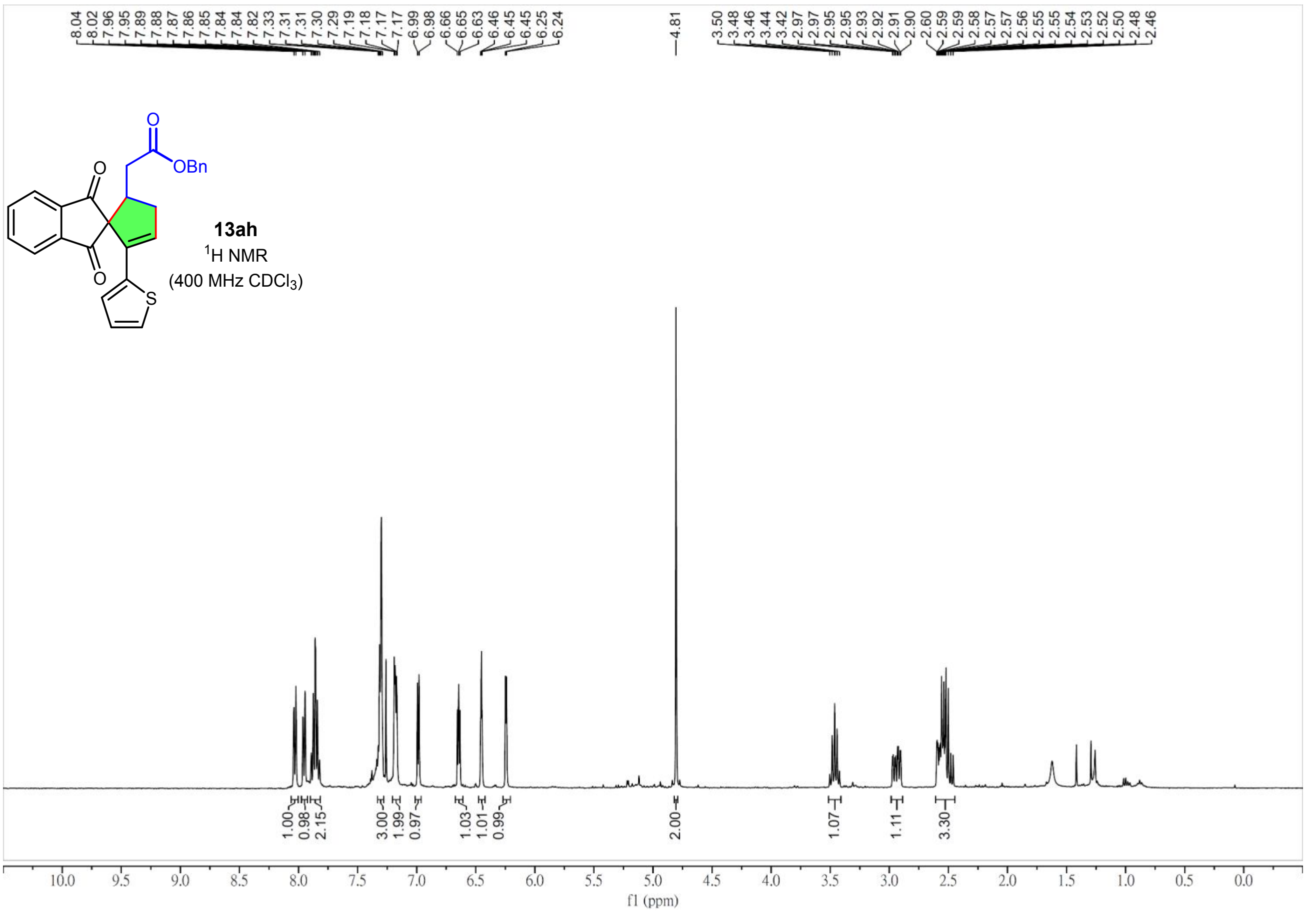
8.04
8.02
7.96
7.95
7.89
7.88
7.87
7.86
7.85
7.84
7.84
7.82
7.33
7.31
7.30
7.29
7.19
7.18
7.17
7.17
6.99
6.98
6.66
6.65
6.63
6.46
6.45
6.45
6.25
6.24
4.81
3.50
3.48
3.46
3.44
3.42
2.97
2.97
2.95
2.95
2.93
2.92
2.91
2.90
2.60
2.59
2.59
2.58
2.57
2.57
2.56
2.55
2.55
2.54
2.53
2.52
2.50
2.48
2.46
OBn
13ah
1H NMR
(400 MHz CDCl3)
1.00
0.98
2.15
3.00
1.99
0.97
1.03
1.01
0.99
2.00
1.07
1.11
3.30
f1 (ppm)
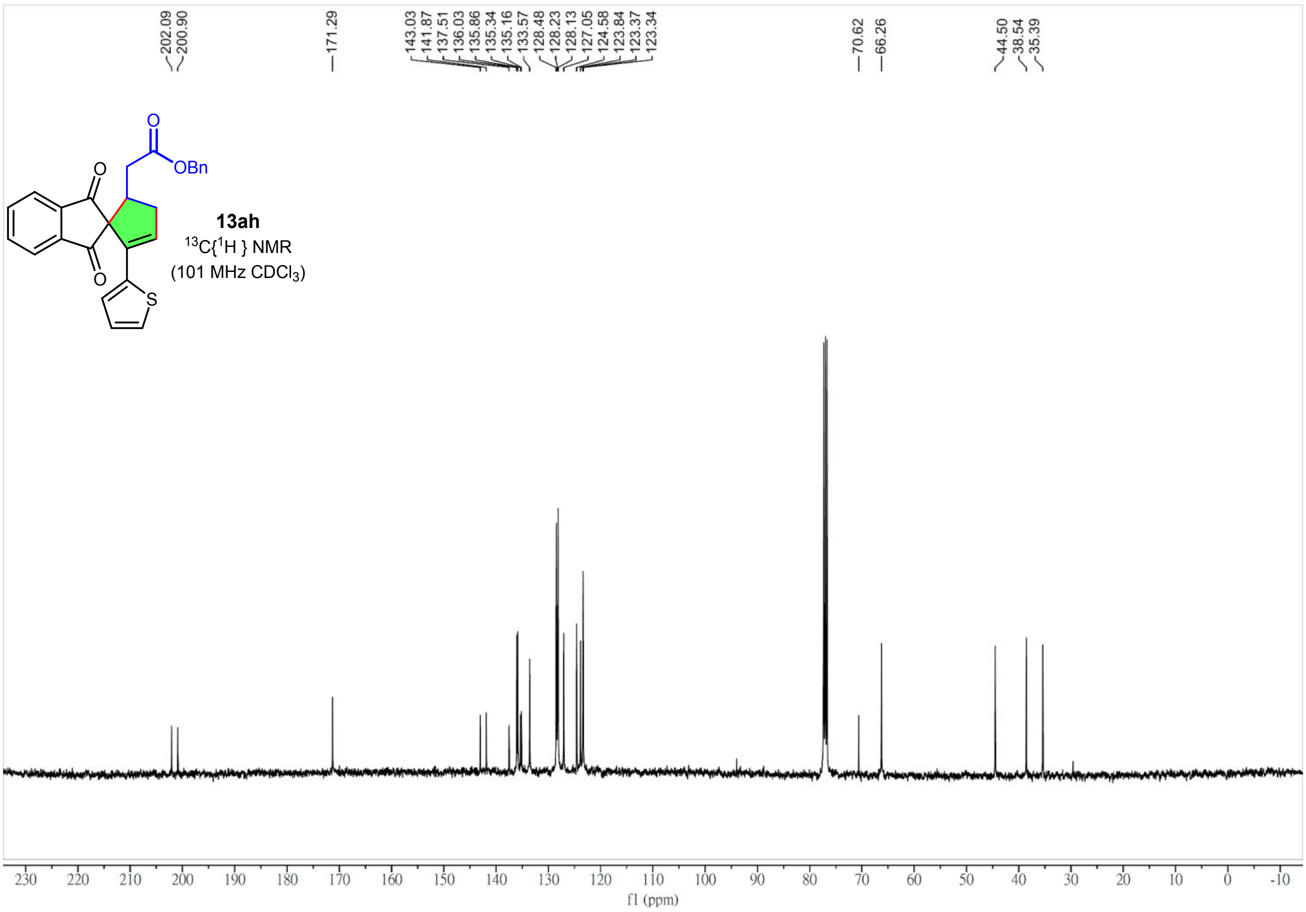
202.09
200.90
171.29
143.03
141.87
137.51
136.03
135.86
135.34
135.16
133.57
128.48
128.23
128.13
127.05
124.58
123.84
123.37
123.34
70.62
66.26
44.50
38.54
35.39
OBn
13ah
13C{1H } NMR
(101 MHz CDCl3)
f1 (ppm)

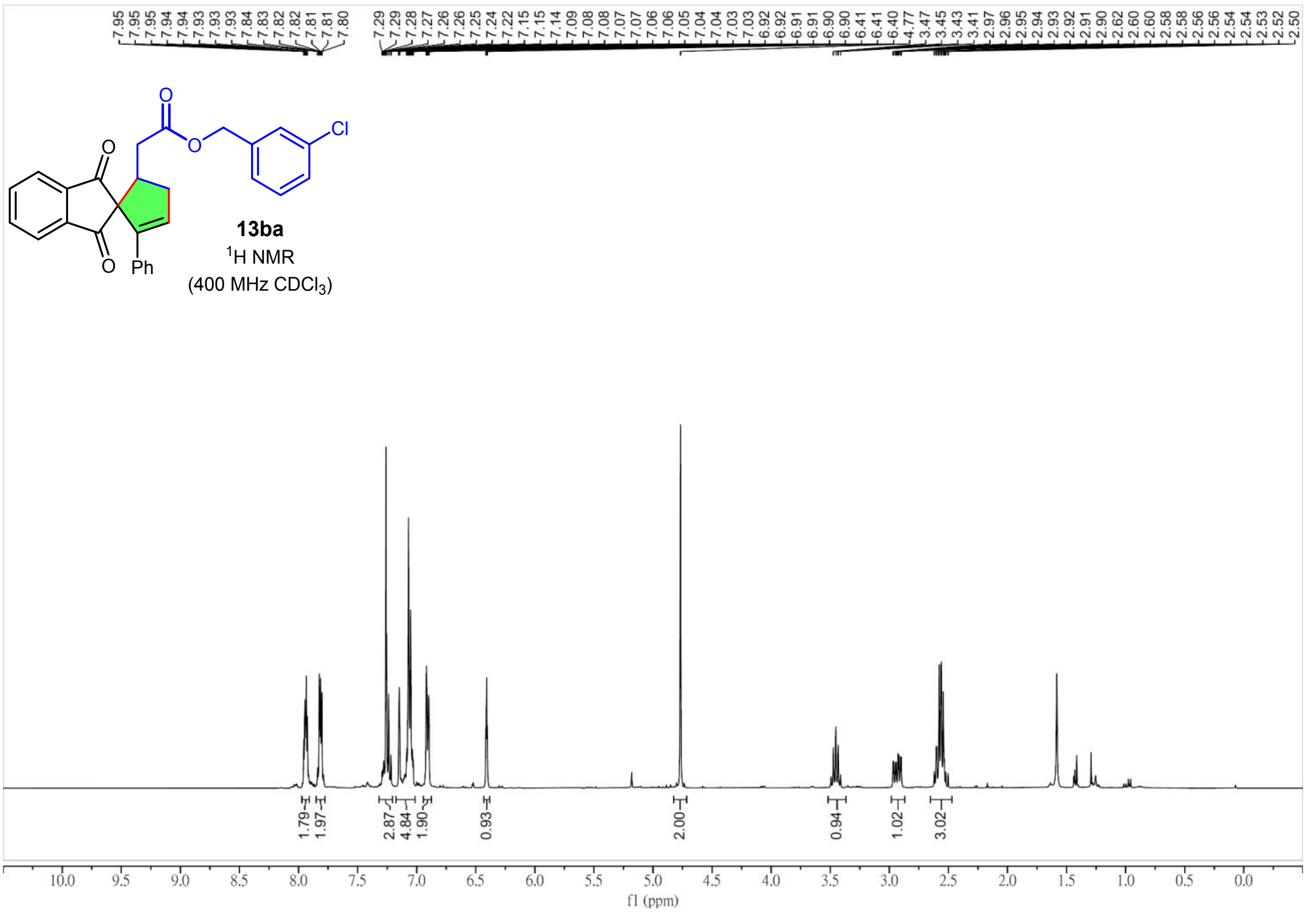

13ba

^{1}H NMR

(400 MHz $CDCl_3$)

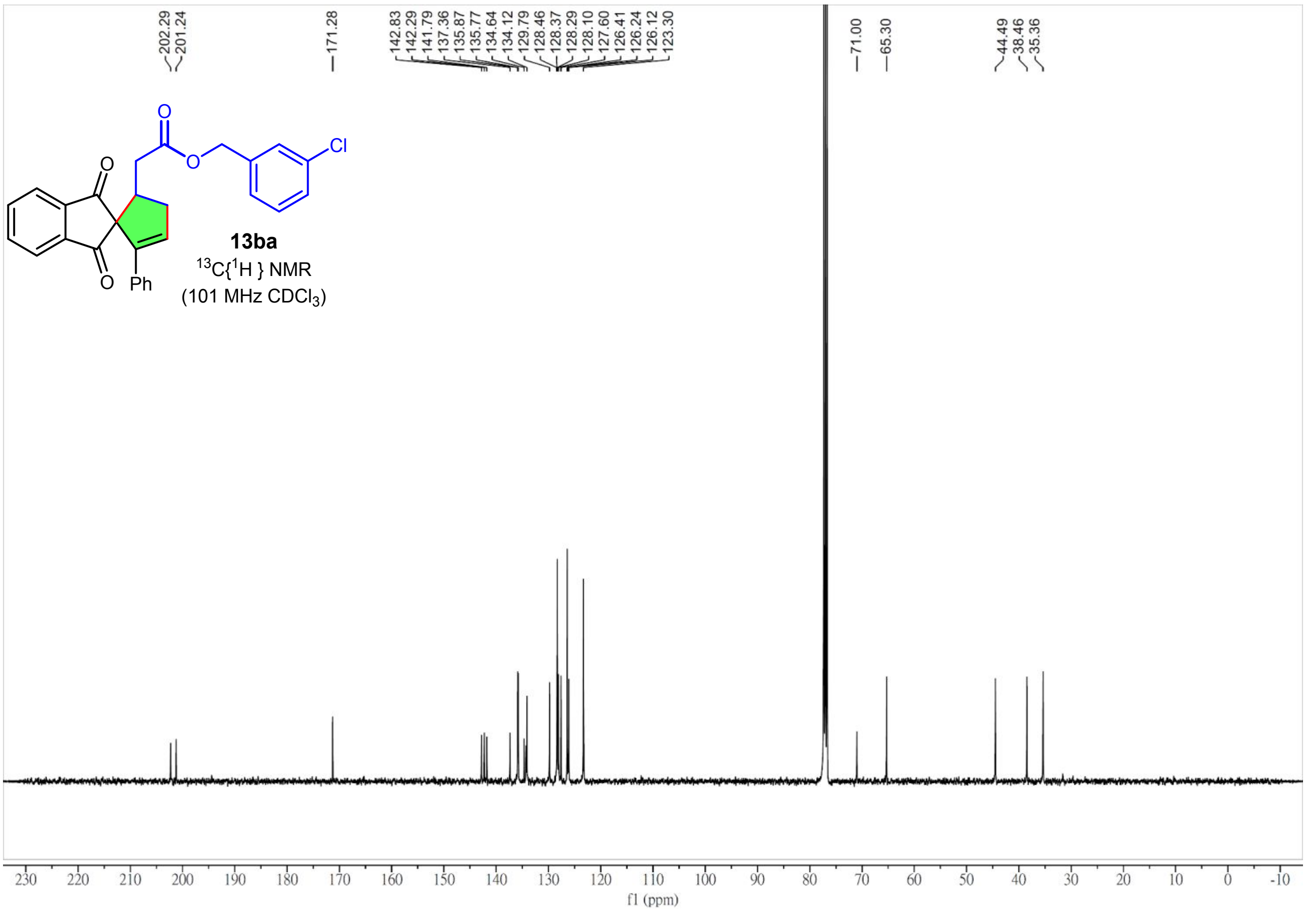

13ba

$^{13}C\{^1H\}$ NMR

(101 MHz $CDCl_3$)

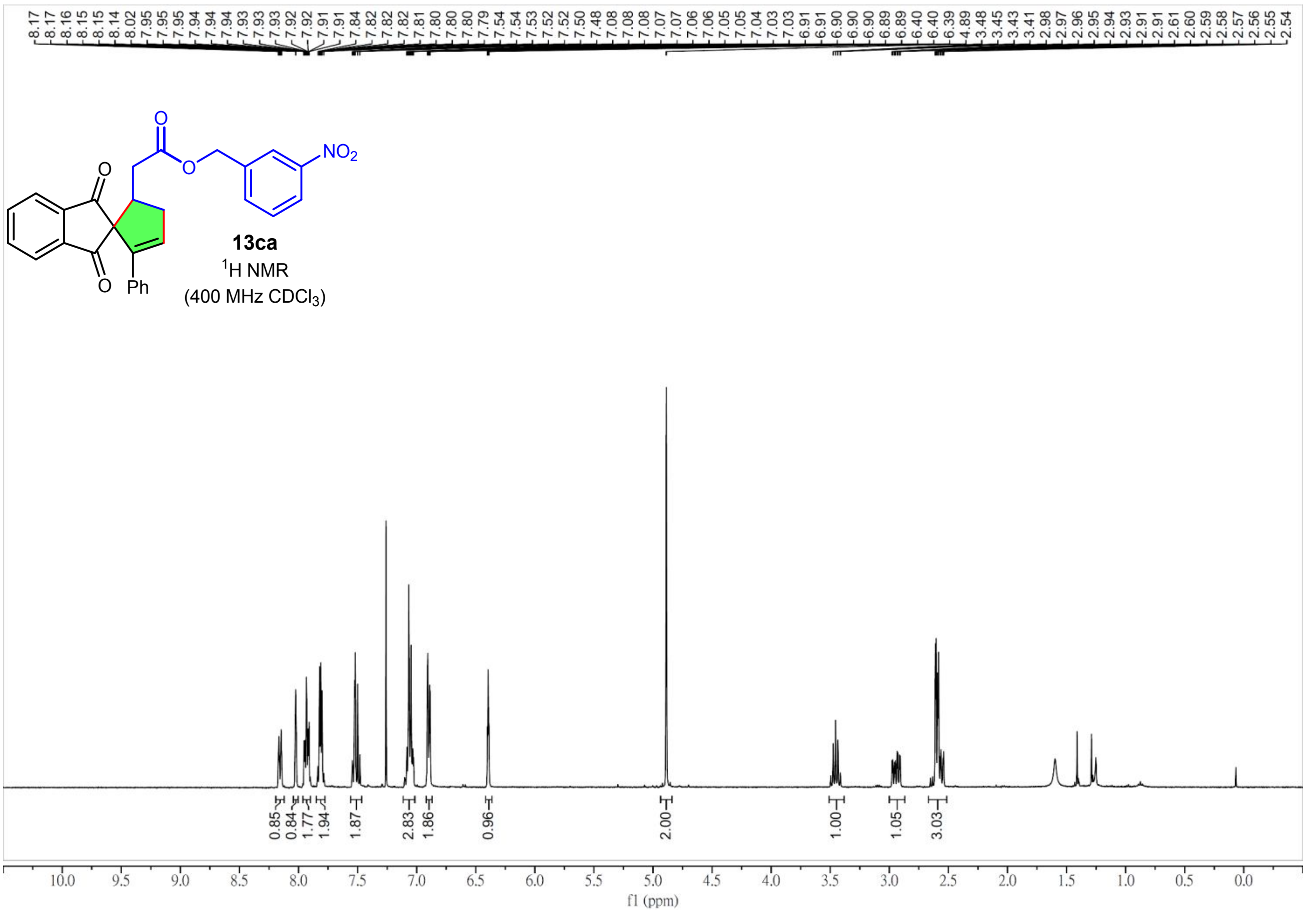
13ca
^{1}H NMR
(400 MHz CDCl$_3$)
f1 (ppm)

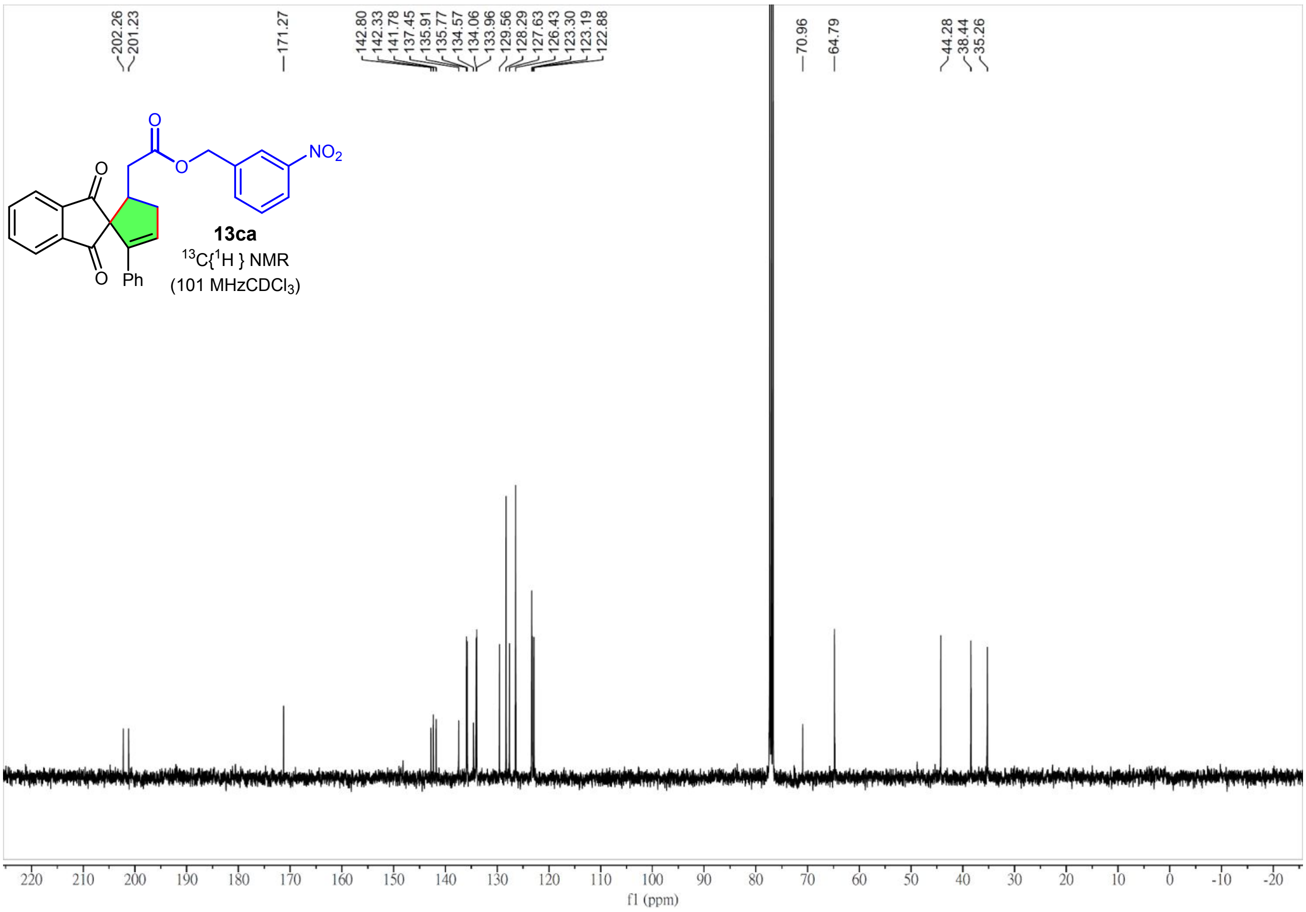
13ca
^{13}C{^{1}H } NMR
(101 MHzCDCl$_3$)
f1 (ppm)

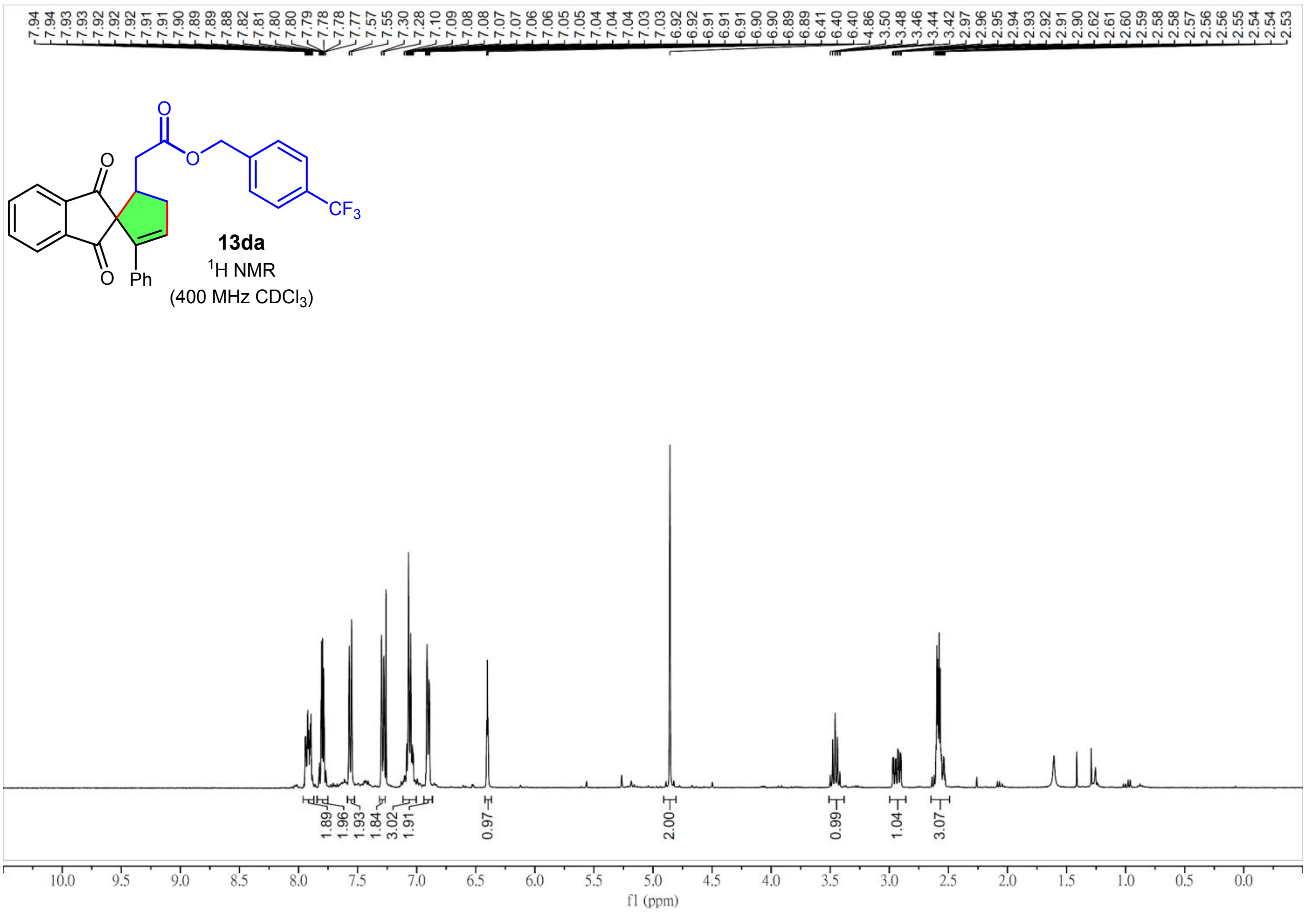
13da
^{1}H NMR
(400 MHz $CDCl_3$)
f1 (ppm)

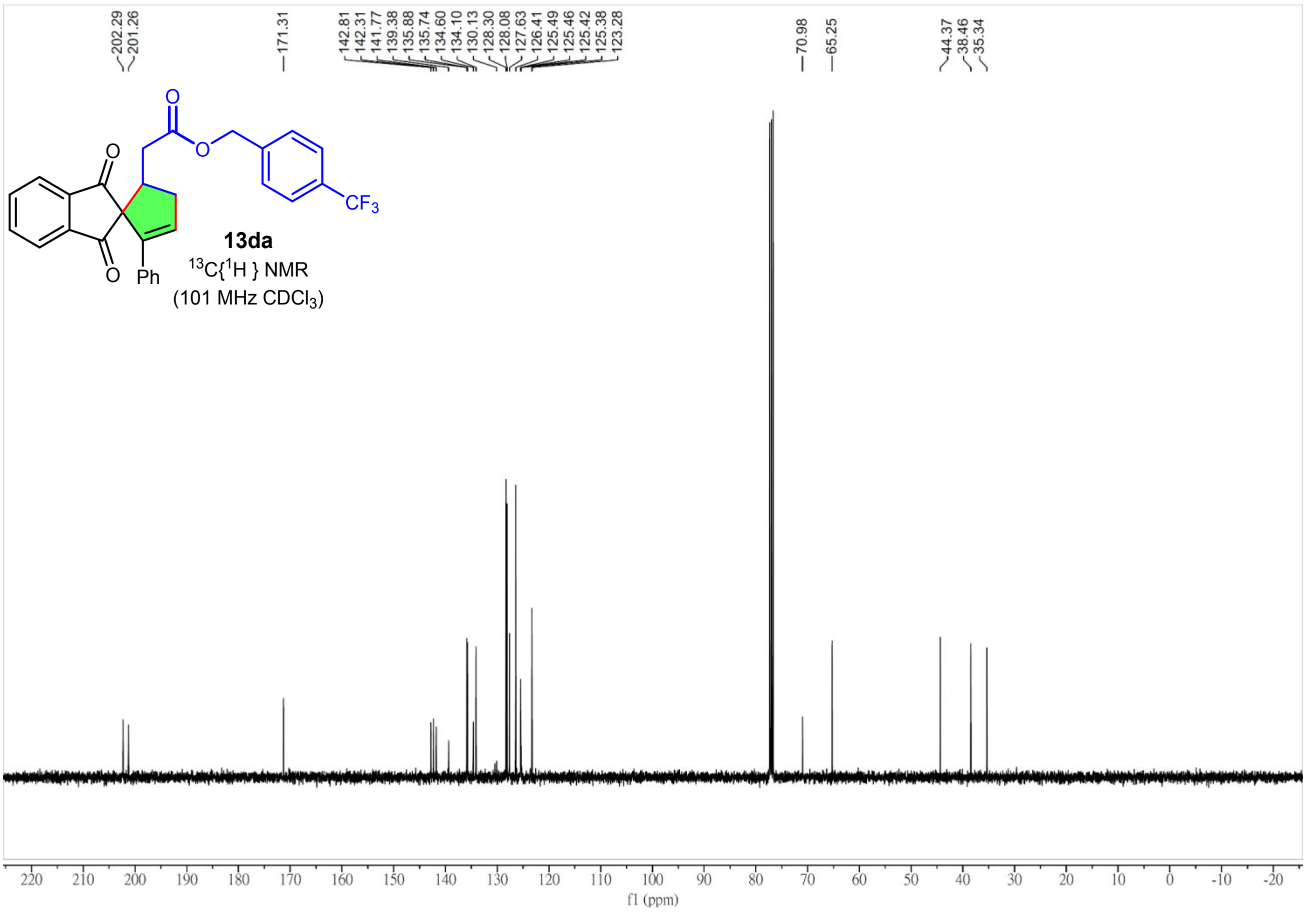
13da
$^{13}C\{^{1}H\}$ NMR
(101 MHz $CDCl_3$)
f1 (ppm)

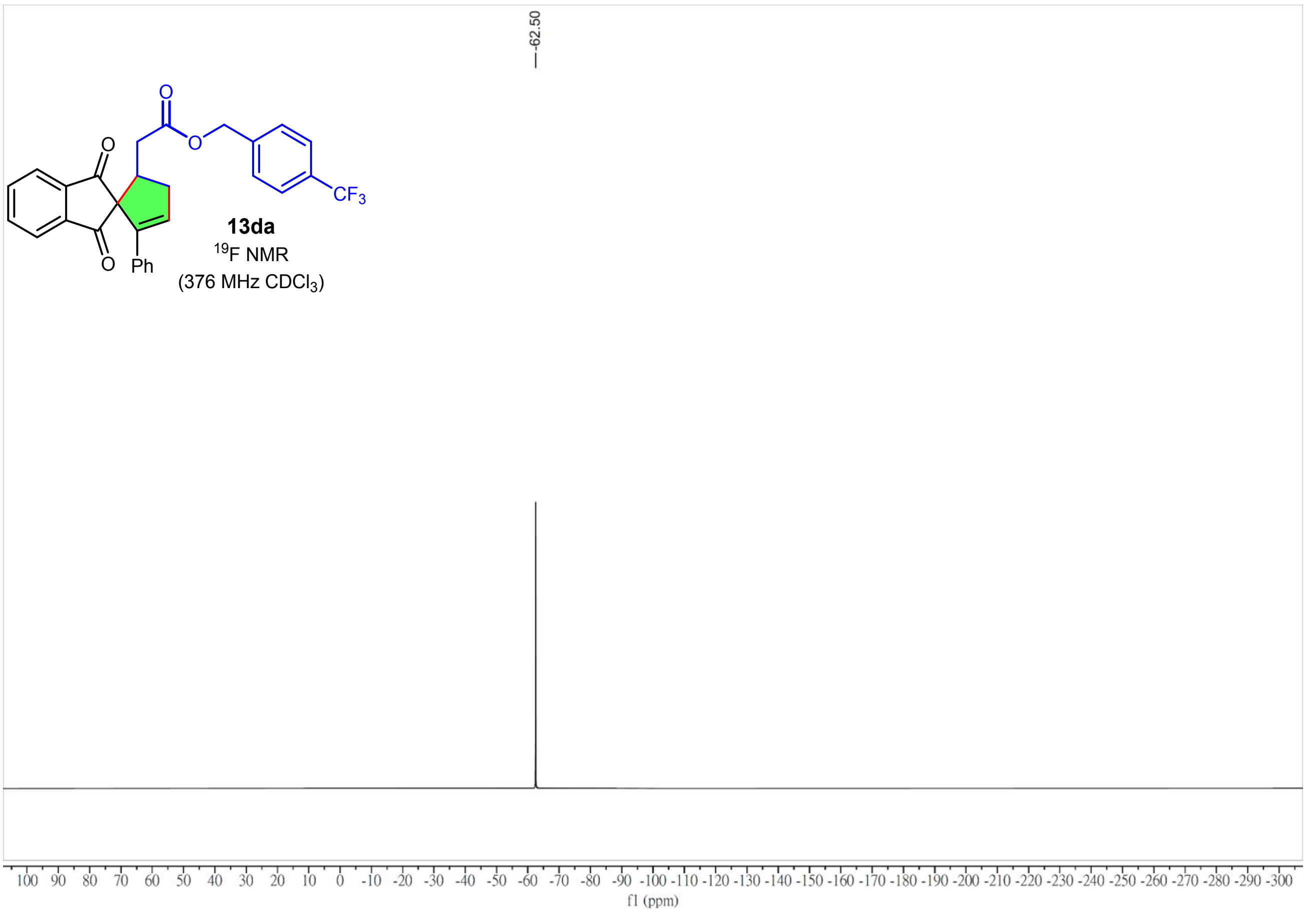

-62.50
O
O
O
O
CF3
Ph
13da
19F NMR
(376 MHz CDCl3)
100 90 80 70 60 50 40 30 20 10 0 -10 -20 -30 -40 -50 -60 -70 -80 -90 -100 -110 -120 -130 -140 -150 -160 -170 -180 -190 -200 -210 -220 -230 -240 -250 -260 -270 -280 -290 -300
f1 (ppm)

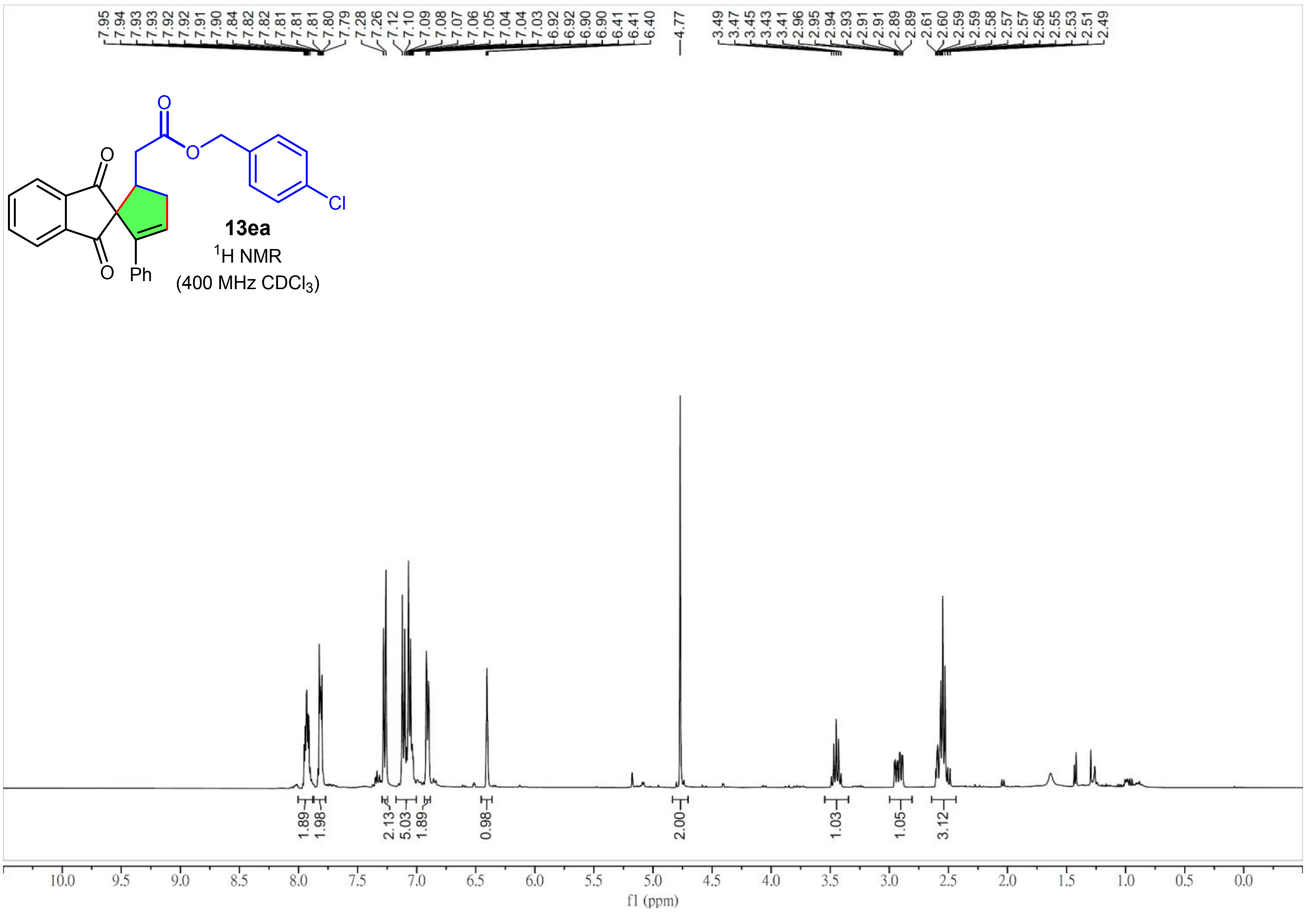
7.95 7.94 7.93 7.93 7.92 7.92 7.91 7.90 7.84 7.82 7.82 7.81 7.81 7.80 7.79 7.28 7.26 7.12 7.10 7.09 7.08 7.07 7.06 7.05 7.04 7.04 7.03 6.92 6.92 6.90 6.90 6.41 6.41 6.40
4.77
3.49 3.47 3.45 3.43 3.41 2.96 2.95 2.94 2.93 2.91 2.91 2.89 2.89 2.61 2.60 2.59 2.59 2.58 2.57 2.57 2.56 2.55 2.53 2.51 2.49
O
O
O
O
Cl
Ph
13ea
^{1}H NMR
(400 MHz $CDCl_3$)
1.89 1.98 2.13 5.03 1.89 0.98 2.00 1.03 1.05 3.12
10.0 9.5 9.0 8.5 8.0 7.5 7.0 6.5 6.0 5.5 5.0 4.5 4.0 3.5 3.0 2.5 2.0 1.5 1.0 0.5 0.0
f1 (ppm)

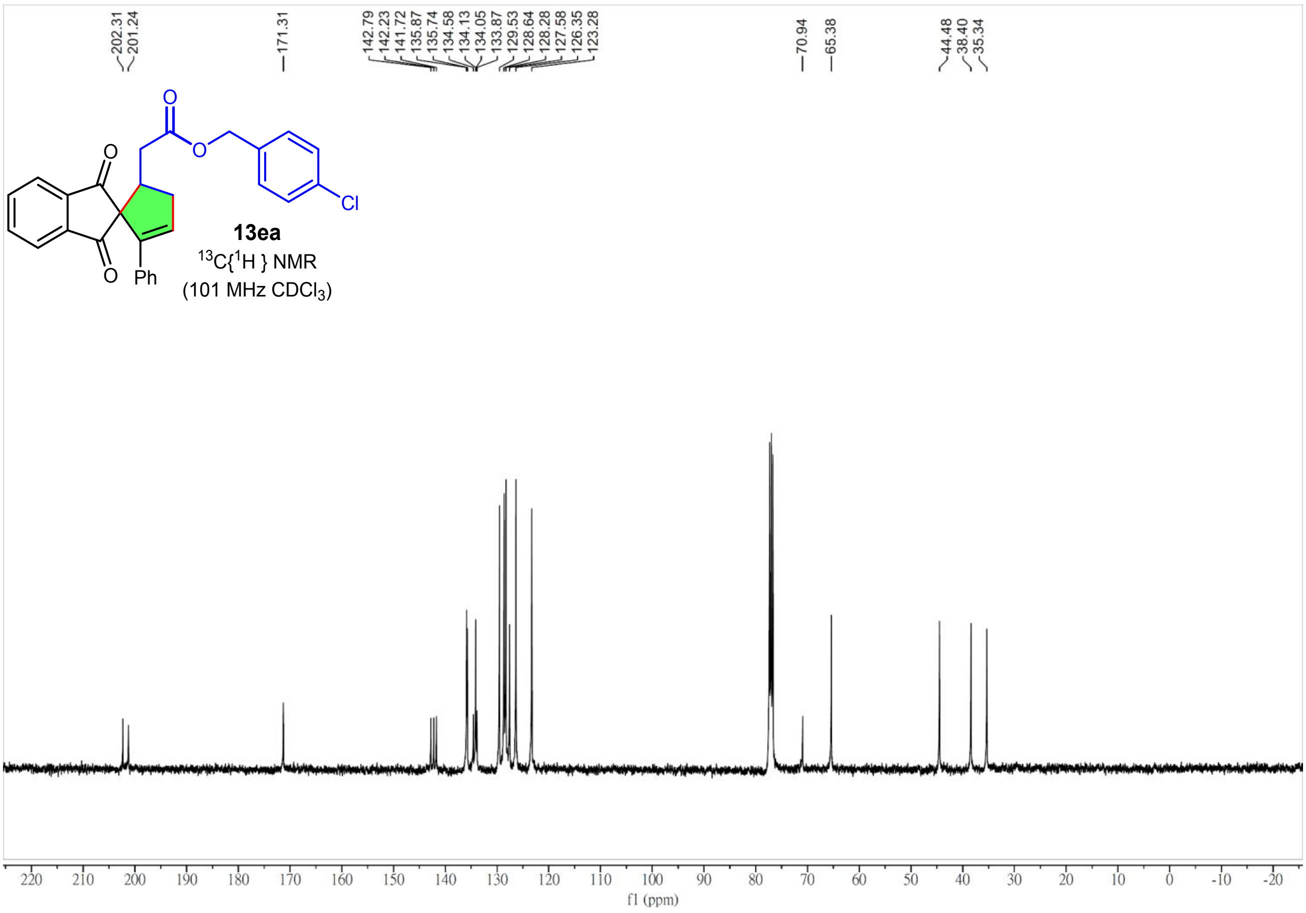
202.31 201.24
171.31
142.79 142.23 141.72 135.87 135.74 134.58 134.13 134.05 133.87 129.53 128.64 128.28 127.58 126.35 123.28
70.94
65.38
44.48 38.40 35.34
O
O
O
O
Cl
Ph
13ea
^{13}C{^{1}H } NMR
(101 MHz $CDCl_3$)
220 210 200 190 180 170 160 150 140 130 120 110 100 90 80 70 60 50 40 30 20 10 0 -10 -20
f1 (ppm)

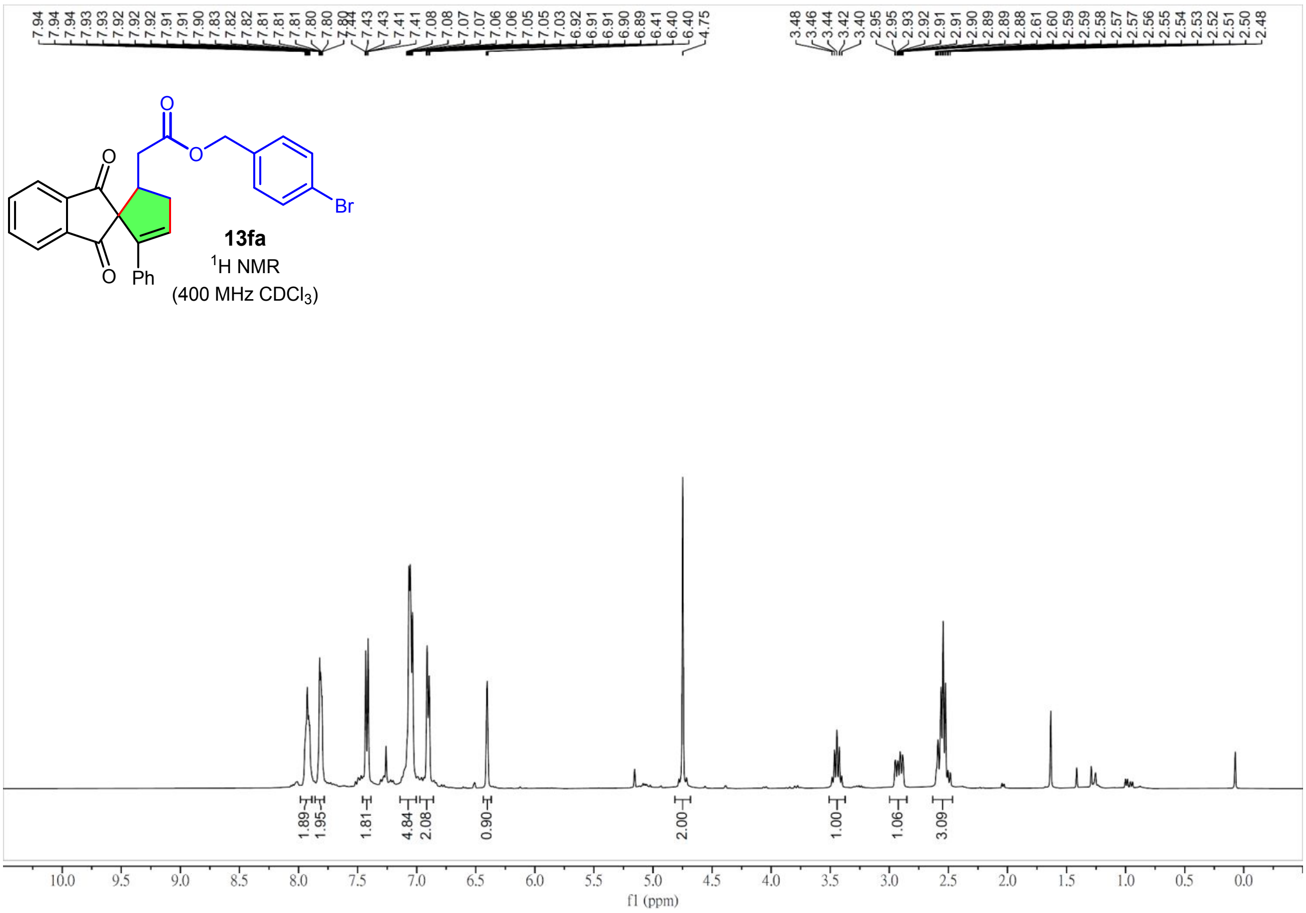

7.94
7.94
7.94
7.93
7.93
7.92
7.92
7.92
7.91
7.91
7.90
7.83
7.82
7.82
7.81
7.81
7.80
7.80
7.44
7.43
7.43
7.41
7.41
7.08
7.08
7.07
7.07
7.06
7.06
7.05
7.05
7.03
6.92
6.91
6.91
6.90
6.89
6.41
6.40
6.40
4.75
3.48
3.46
3.44
3.42
3.40
2.95
2.95
2.93
2.92
2.91
2.91
2.90
2.89
2.89
2.88
2.61
2.60
2.59
2.59
2.58
2.57
2.57
2.56
2.55
2.54
2.53
2.52
2.51
2.50
2.48
O
O
O
O
Br
Ph
13fa
1H NMR
(400 MHz CDCl3)
1.89
1.95
1.81
4.84
2.08
0.90
2.00
1.00
1.06
3.09
10.0 9.5 9.0 8.5 8.0 7.5 7.0 6.5 6.0 5.5 5.0 4.5 4.0 3.5 3.0 2.5 2.0 1.5 1.0 0.5 0.0
f1 (ppm)

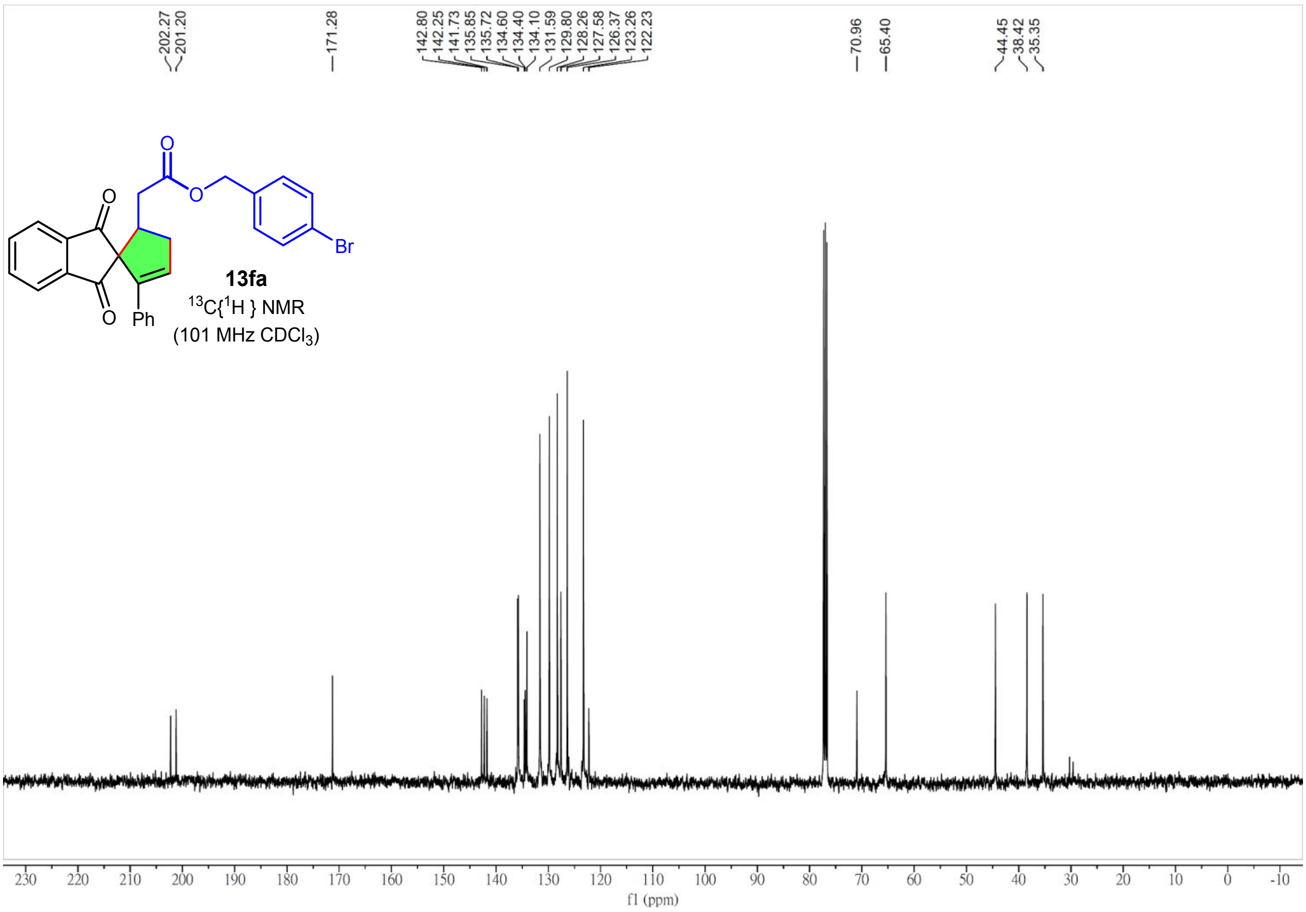

202.27
201.20
171.28
142.80
142.25
141.73
135.85
135.72
134.60
134.40
134.10
131.59
129.80
128.26
127.58
126.37
123.26
122.23
70.96
65.40
44.45
38.42
35.35
O
O
O
O
Br
Ph
13fa
13C{1H } NMR
(101 MHz CDCl3)
230 220 210 200 190 180 170 160 150 140 130 120 110 100 90 80 70 60 50 40 30 20 10 0 -10
f1 (ppm)

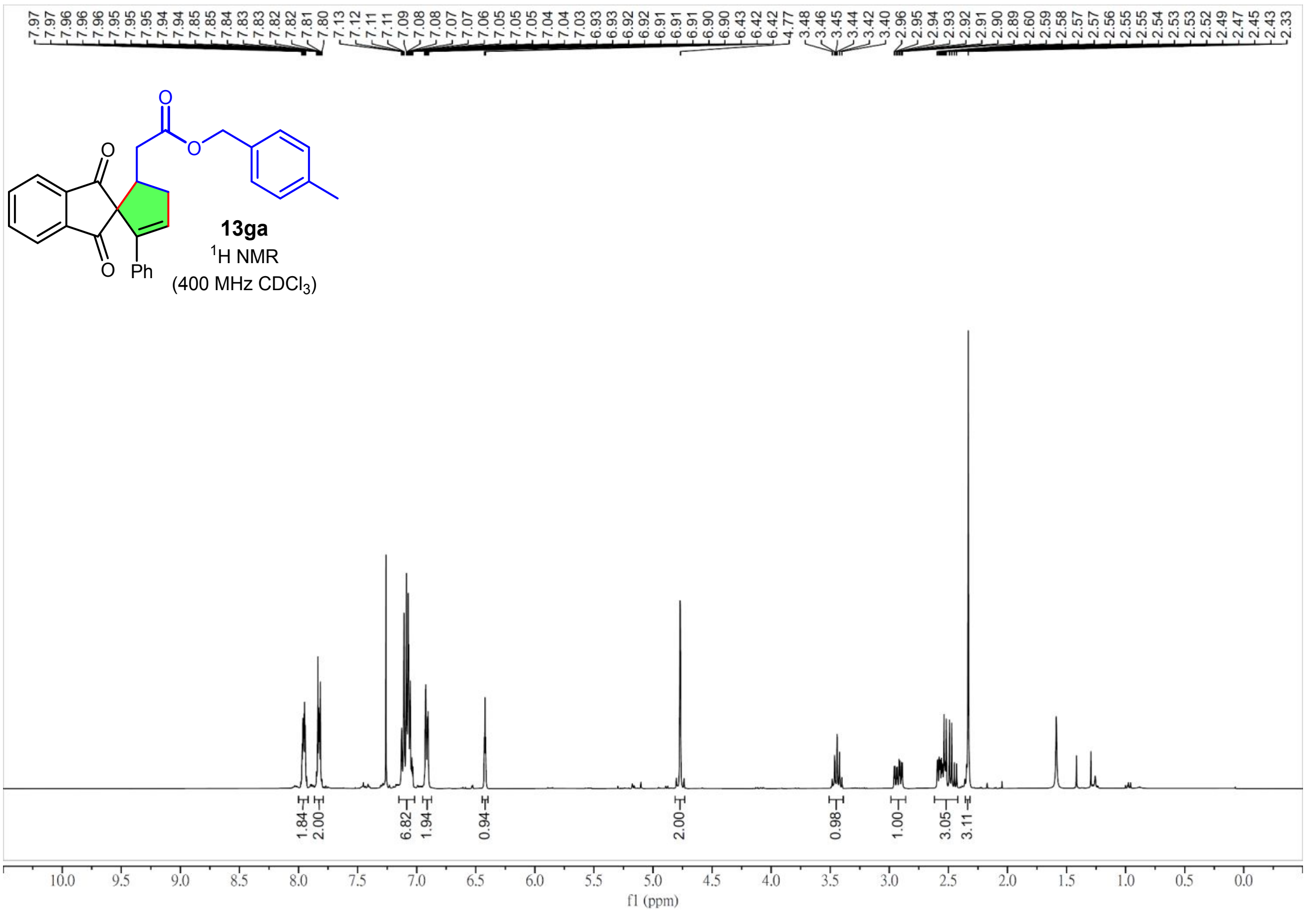
13ga
1H NMR
(400 MHz CDCl3)
f1 (ppm)

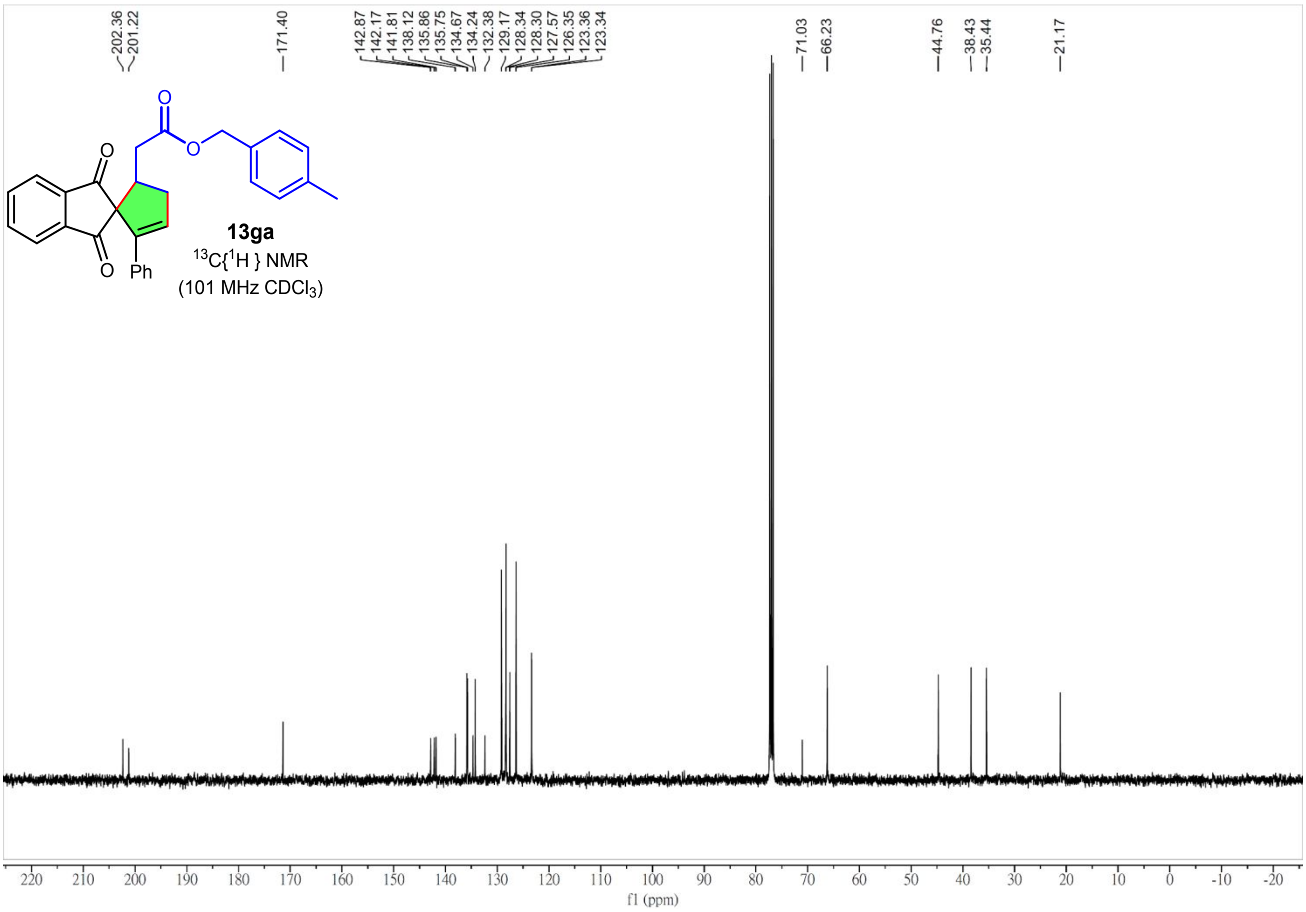
13ga
13C{1H } NMR
(101 MHz CDCl3)
f1 (ppm)

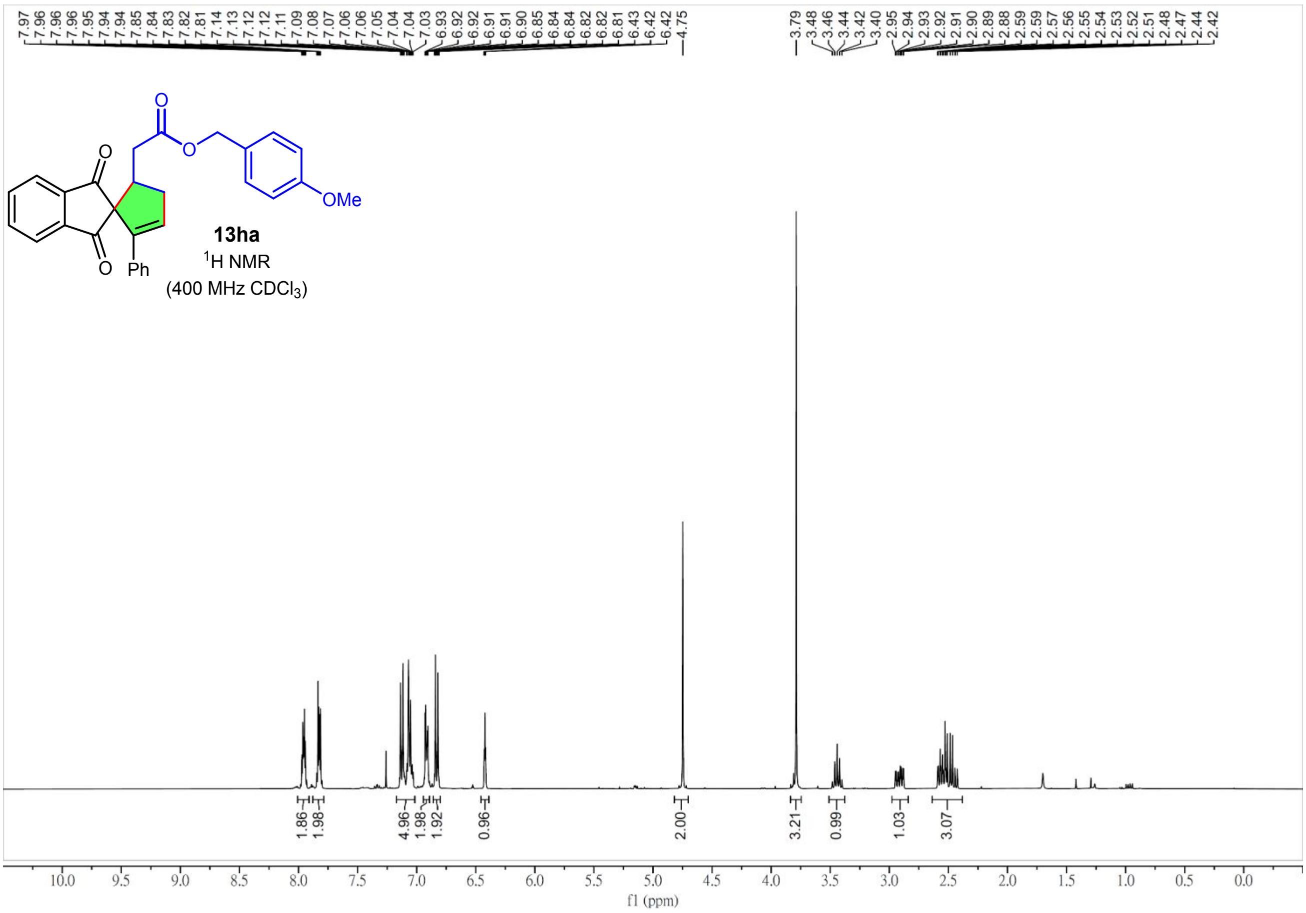
7.97 7.96 7.96 7.95 7.94 7.94 7.85 7.84 7.83 7.82 7.81 7.14 7.13 7.12 7.12 7.11 7.09 7.08 7.07 7.06 7.06 7.05 7.04 7.04 7.03 6.93 6.92 6.92 6.91 6.91 6.90 6.85 6.84 6.84 6.82 6.82 6.81 6.43 6.42 6.42 4.75
3.79 3.48 3.46 3.44 3.42 3.40 2.95 2.94 2.93 2.92 2.91 2.90 2.89 2.88 2.59 2.59 2.57 2.56 2.55 2.54 2.53 2.52 2.51 2.48 2.47 2.44 2.42
O
O
O
O
OMe
Ph
13ha
1H NMR
(400 MHz CDCl3)
1.86 1.98 4.96 1.98 1.92 0.96 2.00 3.21 0.99 1.03 3.07
f1 (ppm)

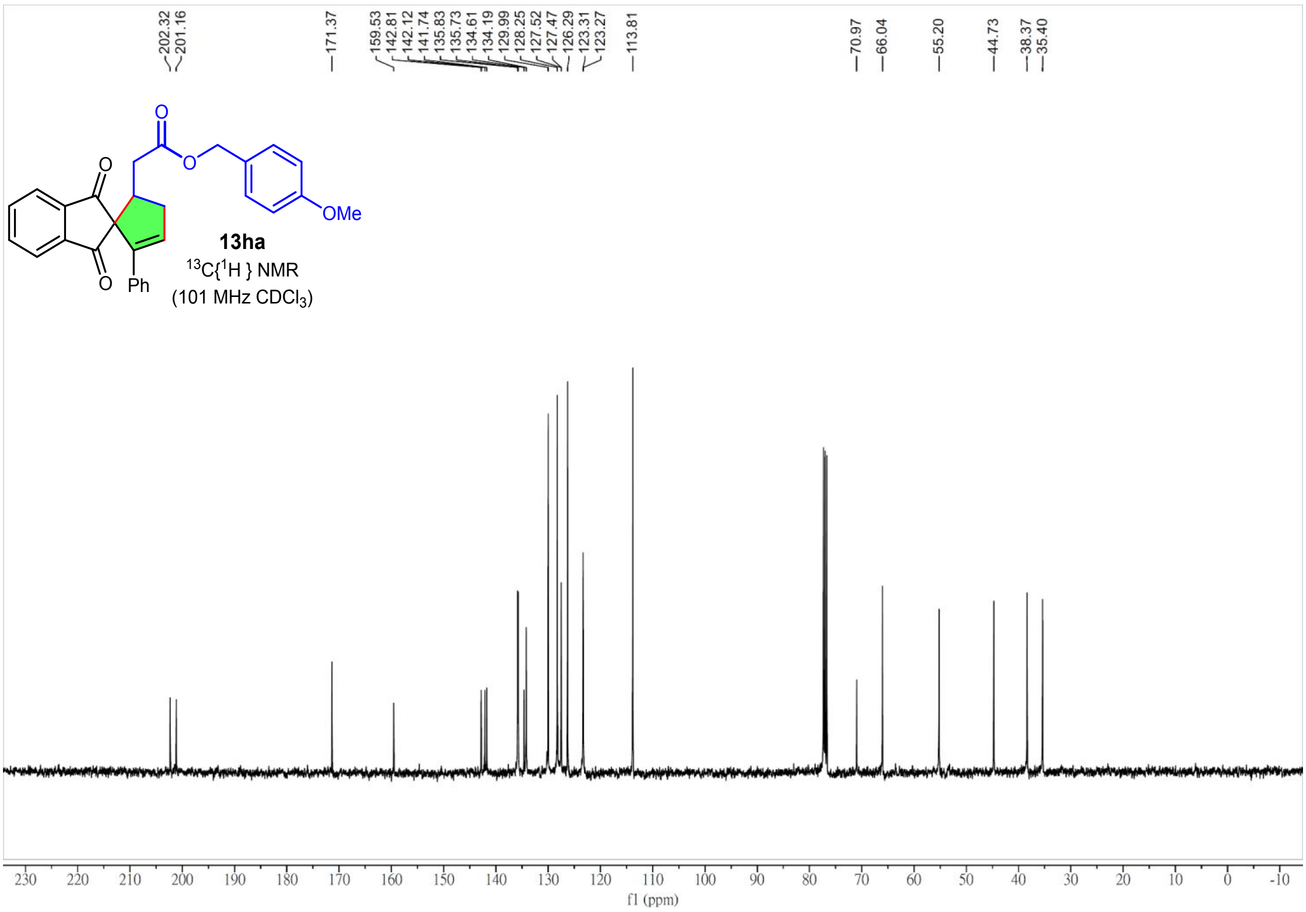
202.32 201.16 171.37 159.53 142.81 142.12 141.74 135.83 135.73 134.61 134.19 129.99 128.25 127.52 127.47 126.29 123.31 123.27 113.81 70.97 66.04 55.20 44.73 38.37 35.40
O
O
O
O
OMe
Ph
13ha
13C{1H } NMR
(101 MHz CDCl3)
f1 (ppm)

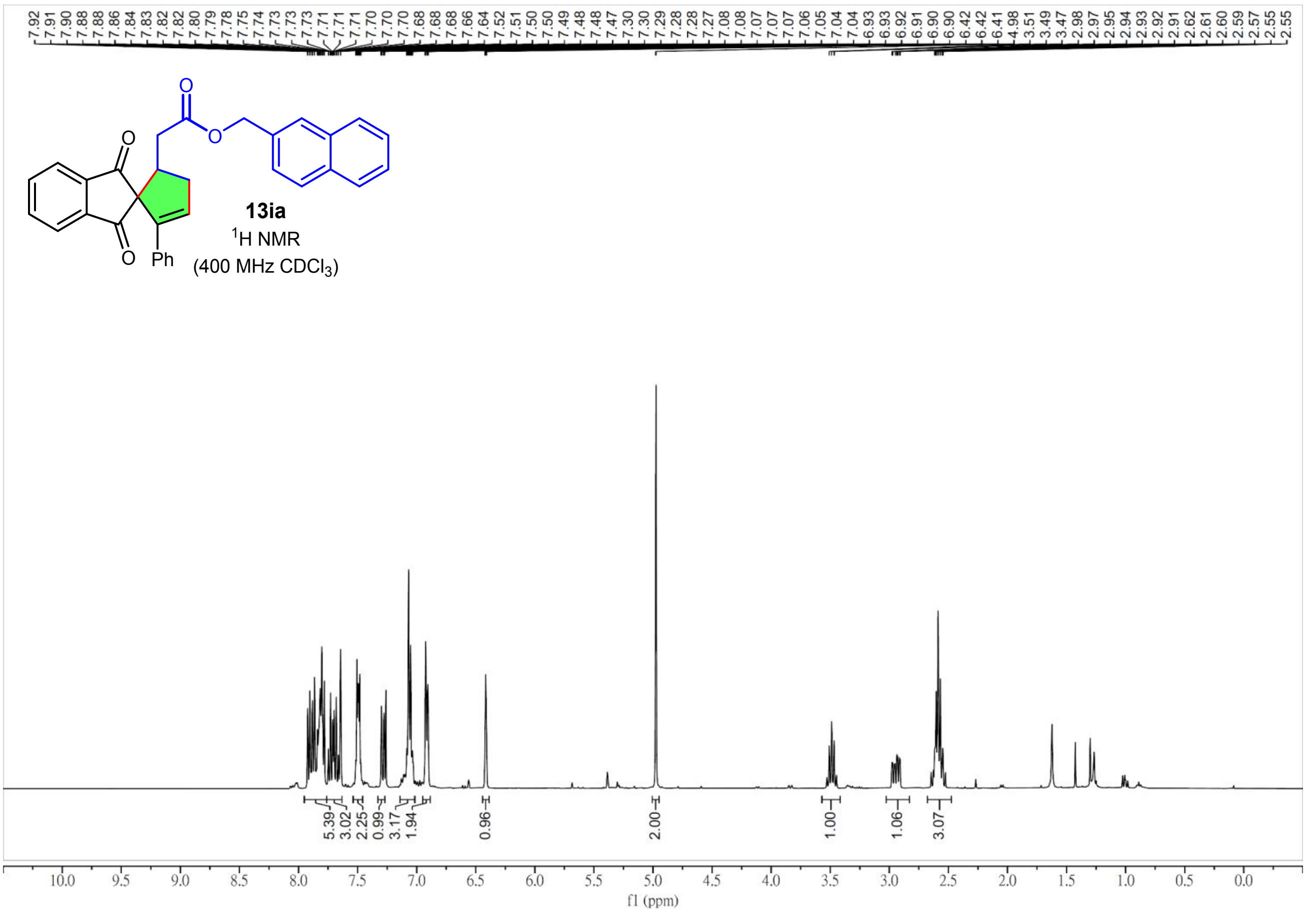
13ia
^{1}H NMR
(400 MHz CDCl$_3$)
f1 (ppm)

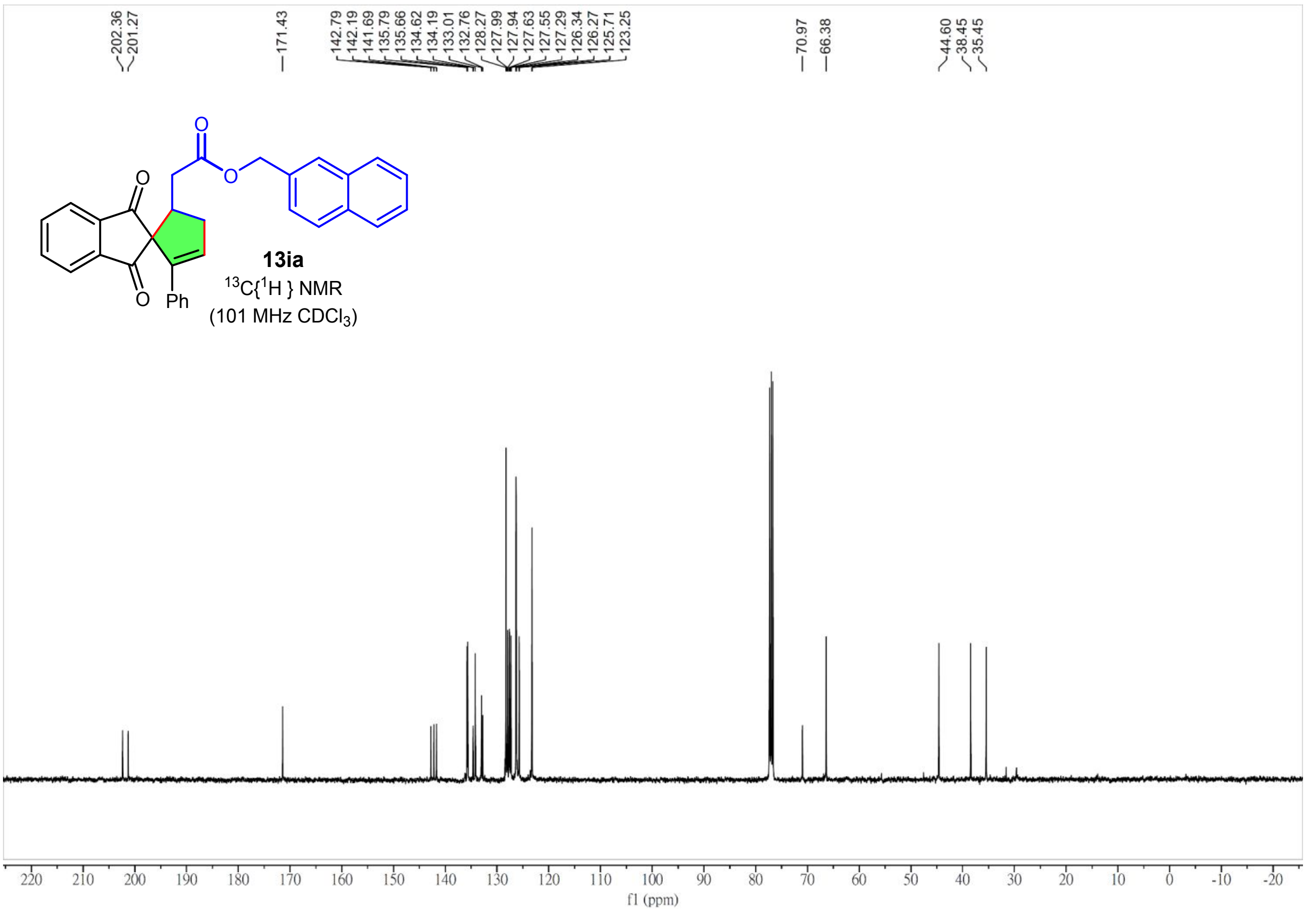
13ia
^{13}C{^{1}H } NMR
(101 MHz CDCl$_3$)
f1 (ppm)

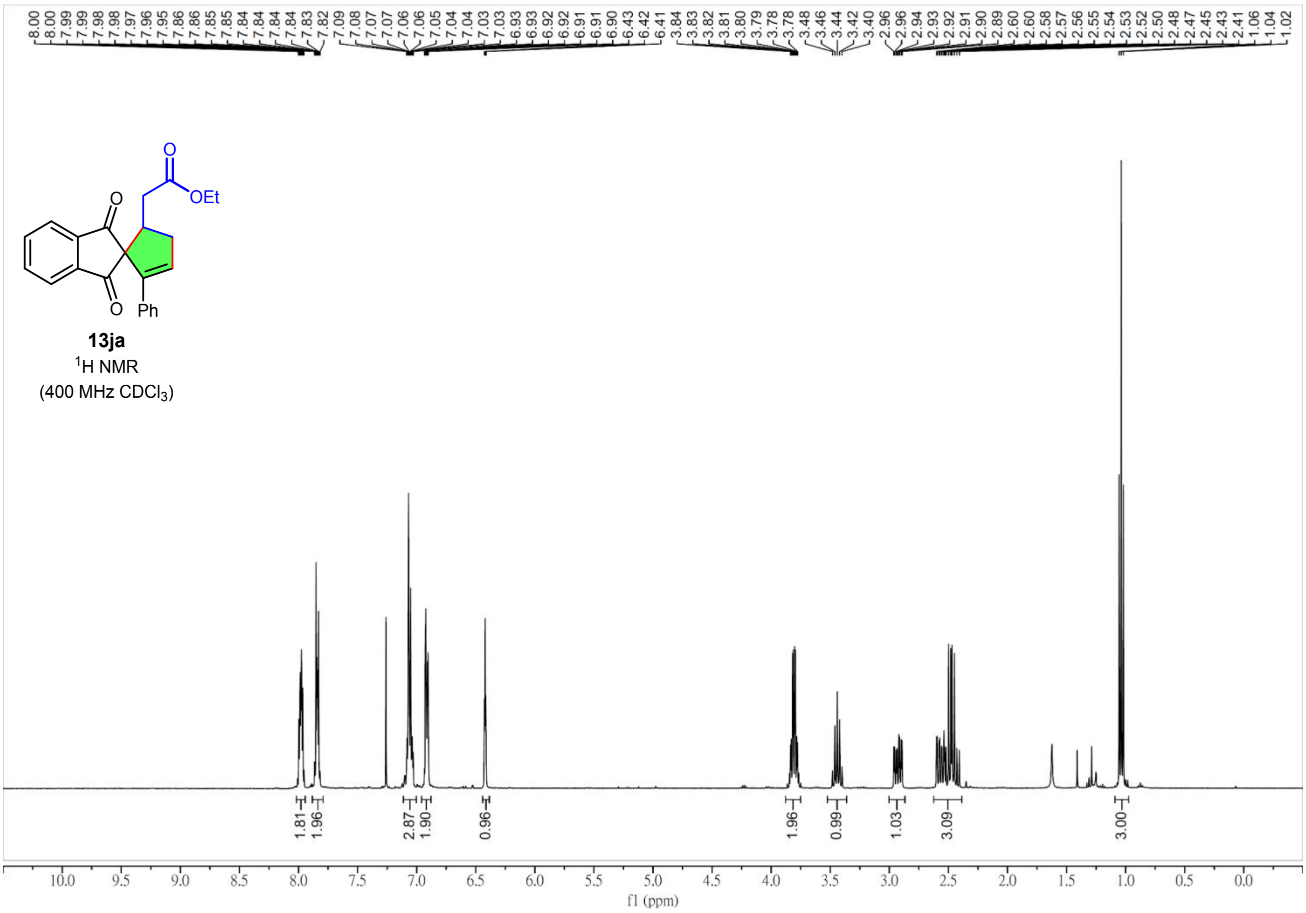
O
OEt
O
O
Ph
13ja
1H NMR
(400 MHz CDCl3)
f1 (ppm)

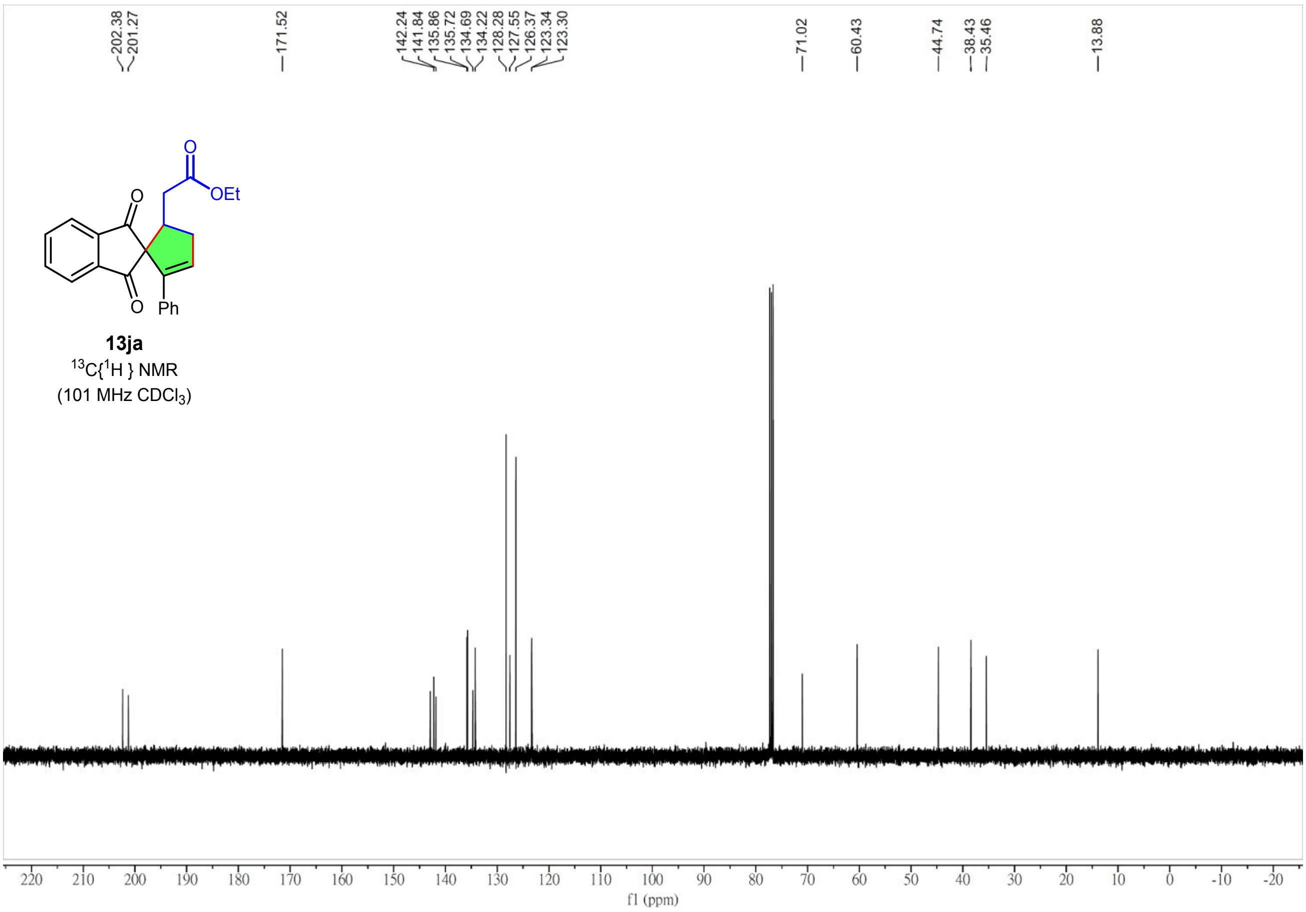
202.38
201.27
171.52
142.24
141.84
135.86
135.72
134.69
134.22
128.28
127.55
126.37
123.34
123.30
71.02
60.43
44.74
38.43
35.46
13.88
O
OEt
O
O
Ph
13ja
13C{1H } NMR
(101 MHz CDCl3)
f1 (ppm)

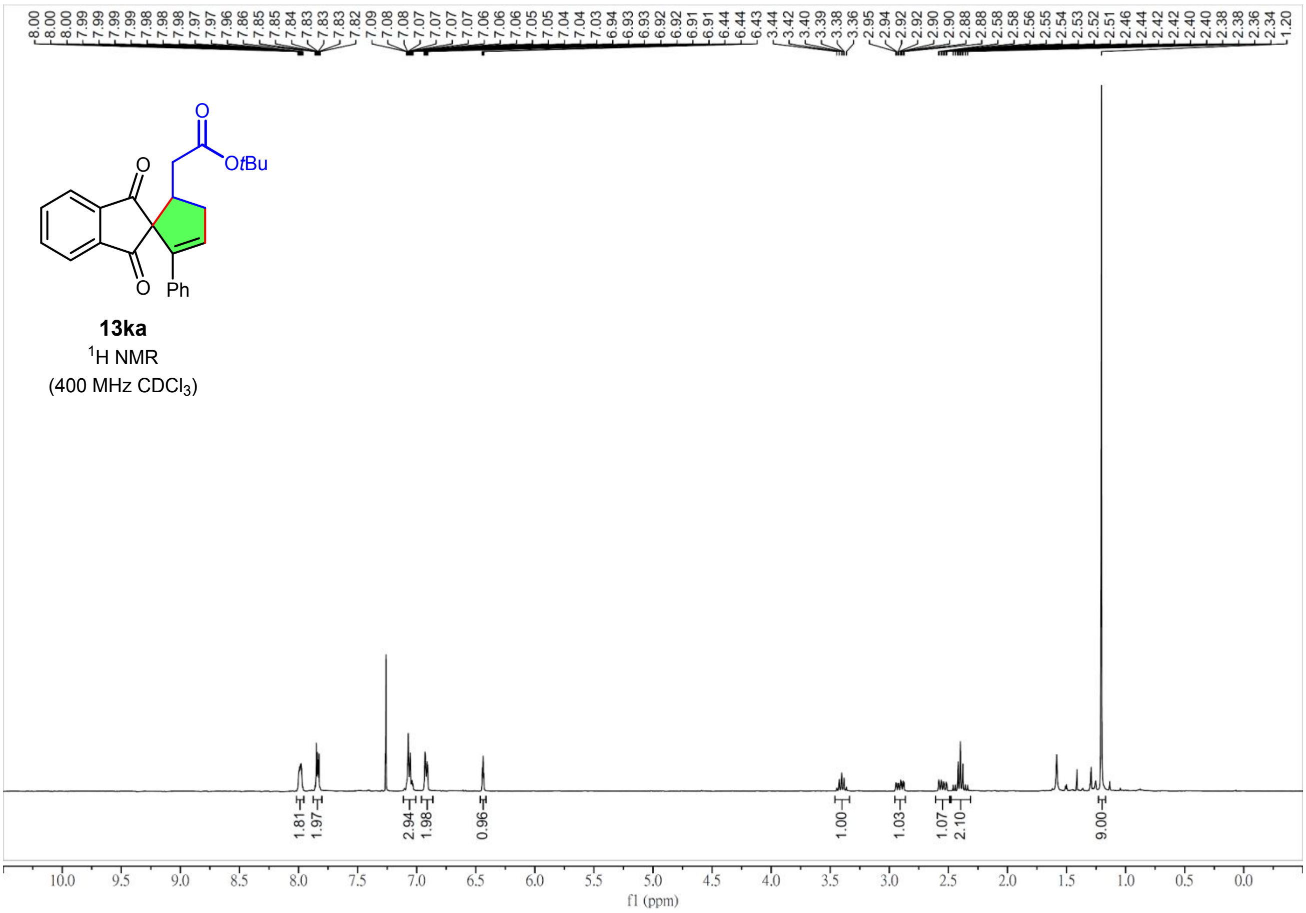

13ka
1H NMR
(400 MHz CDCl3)
OtBu
Ph
f1 (ppm)

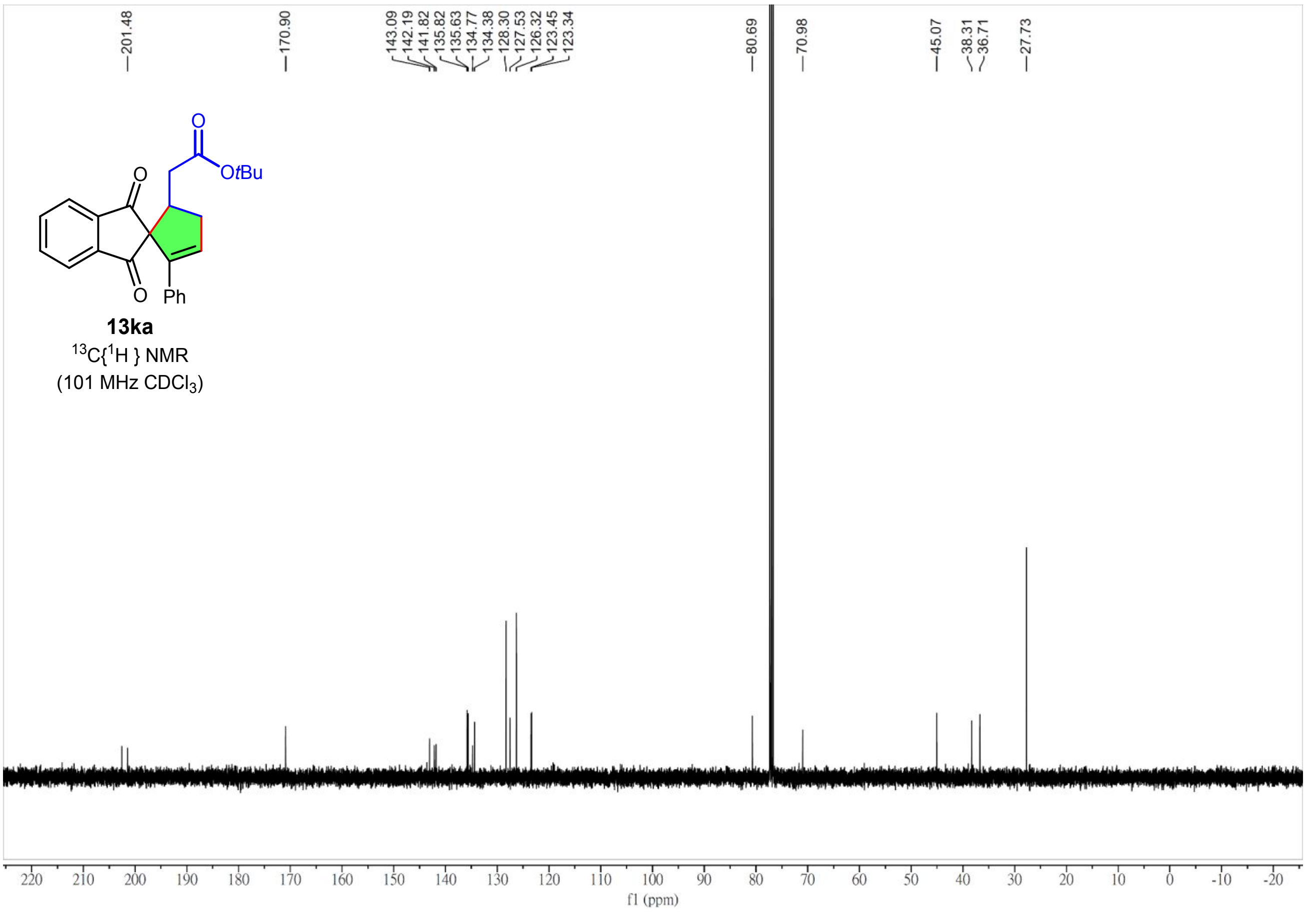

13ka
13C{1H } NMR
(101 MHz CDCl3)
OtBu
Ph
f1 (ppm)

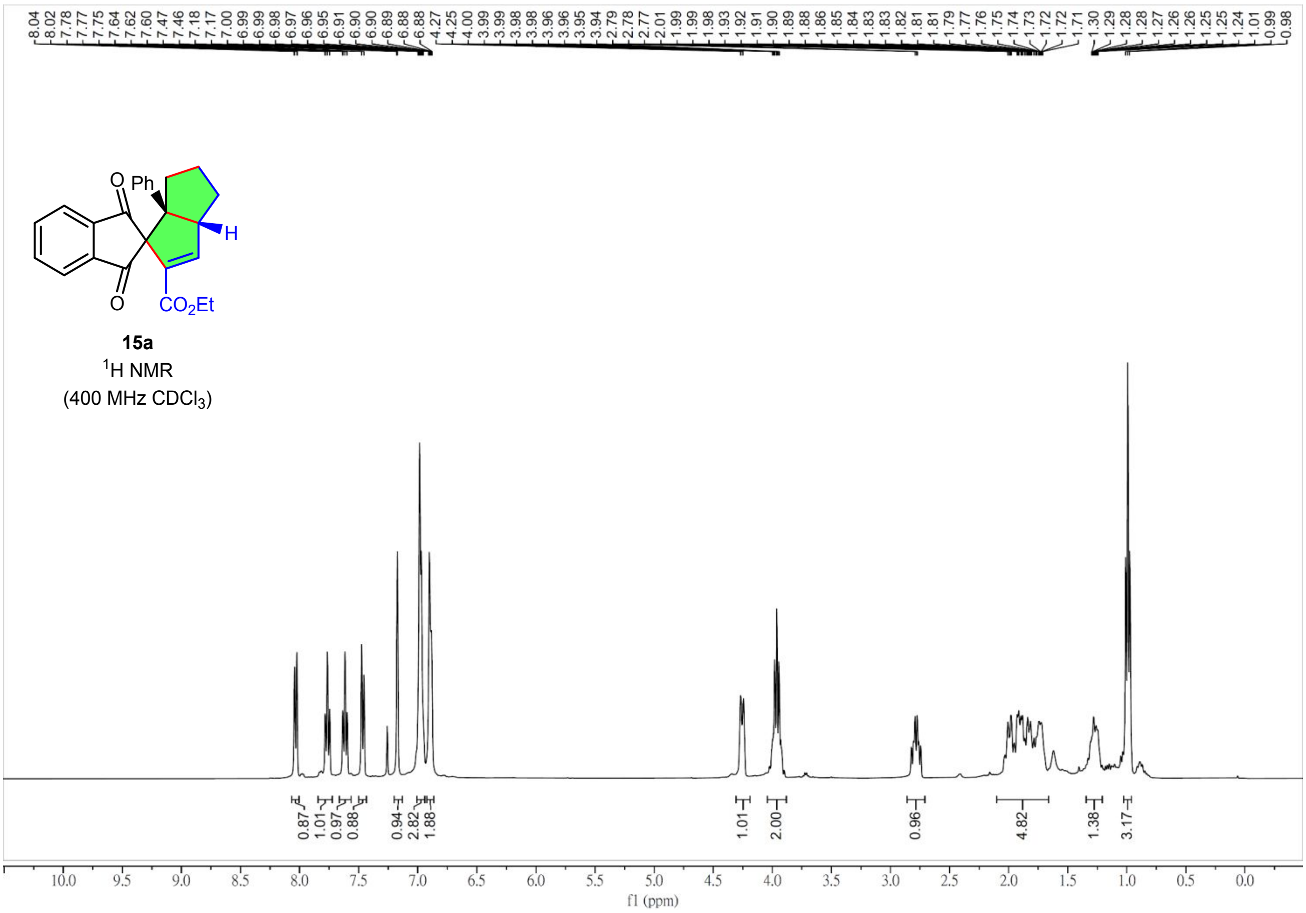
8.04 8.02 7.78 7.77 7.75 7.64 7.62 7.60 7.47 7.46 7.18 7.17 7.00 6.99 6.99 6.98 6.97 6.96 6.95 6.91 6.90 6.90 6.89 6.88 6.88 4.27 4.25 4.00 3.99 3.99 3.98 3.98 3.96 3.96 3.95 3.94 2.79 2.78 2.77 2.01 1.99 1.99 1.98 1.93 1.92 1.91 1.90 1.89 1.88 1.86 1.85 1.84 1.83 1.83 1.82 1.81 1.81 1.79 1.77 1.76 1.75 1.74 1.73 1.72 1.72 1.71 1.30 1.29 1.28 1.28 1.27 1.26 1.26 1.25 1.25 1.24 1.01 0.99 0.98
O
Ph
H
O
CO2Et
15a
1H NMR
(400 MHz CDCl3)
0.87 1.01 0.97 0.88 0.94 2.82 1.88 1.01 2.00 0.96 4.82 1.38 3.17
10.0 9.5 9.0 8.5 8.0 7.5 7.0 6.5 6.0 5.5 5.0 4.5 4.0 3.5 3.0 2.5 2.0 1.5 1.0 0.5 0.0
f1 (ppm)

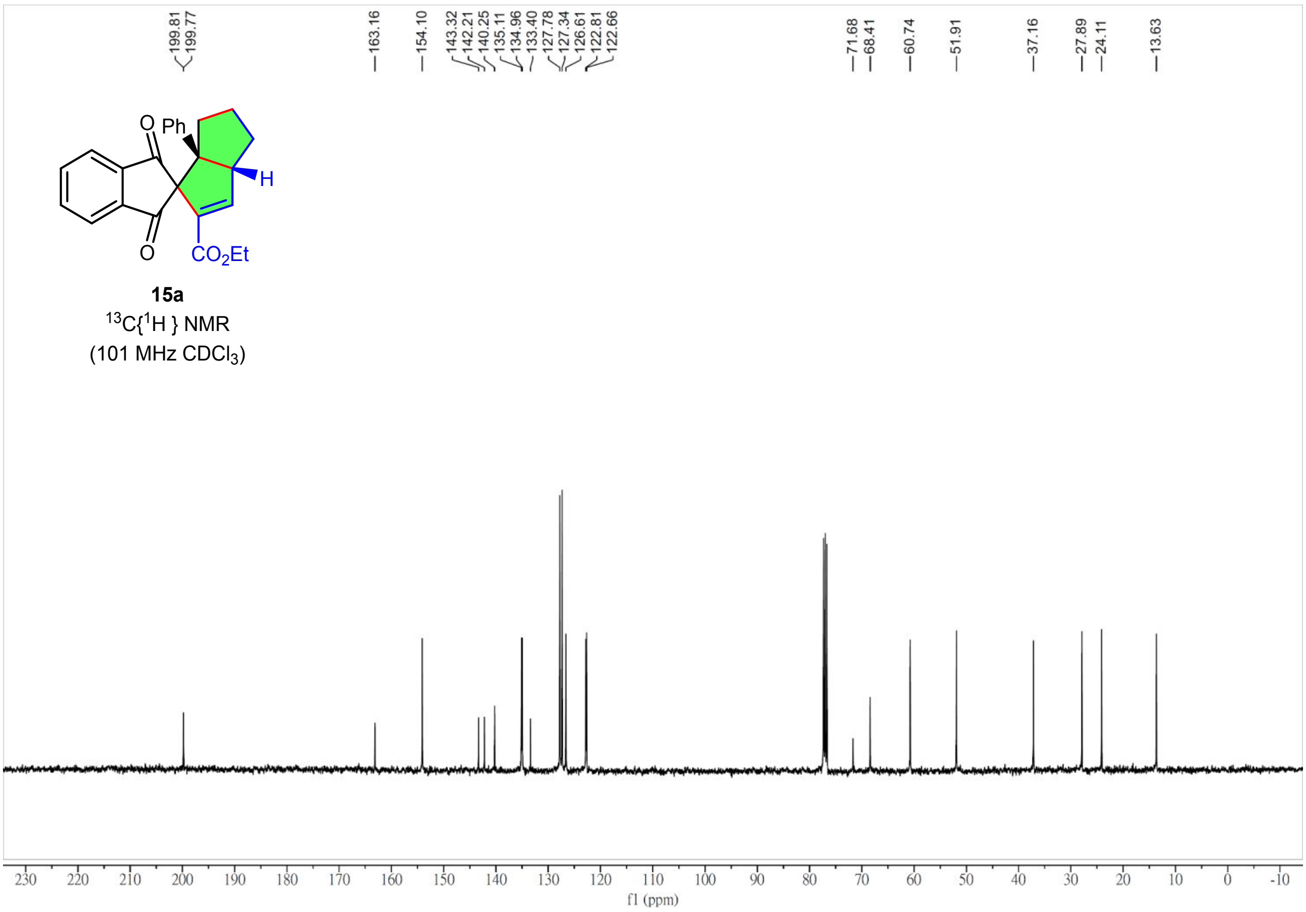
199.81 199.77 163.16 154.10 143.32 142.21 140.25 135.11 134.96 133.40 127.78 127.34 126.61 122.81 122.66 71.68 68.41 60.74 51.91 37.16 27.89 24.11 13.63
O
Ph
H
O
CO2Et
15a
13C{1H } NMR
(101 MHz CDCl3)
230 220 210 200 190 180 170 160 150 140 130 120 110 100 90 80 70 60 50 40 30 20 10 0 -10
f1 (ppm)

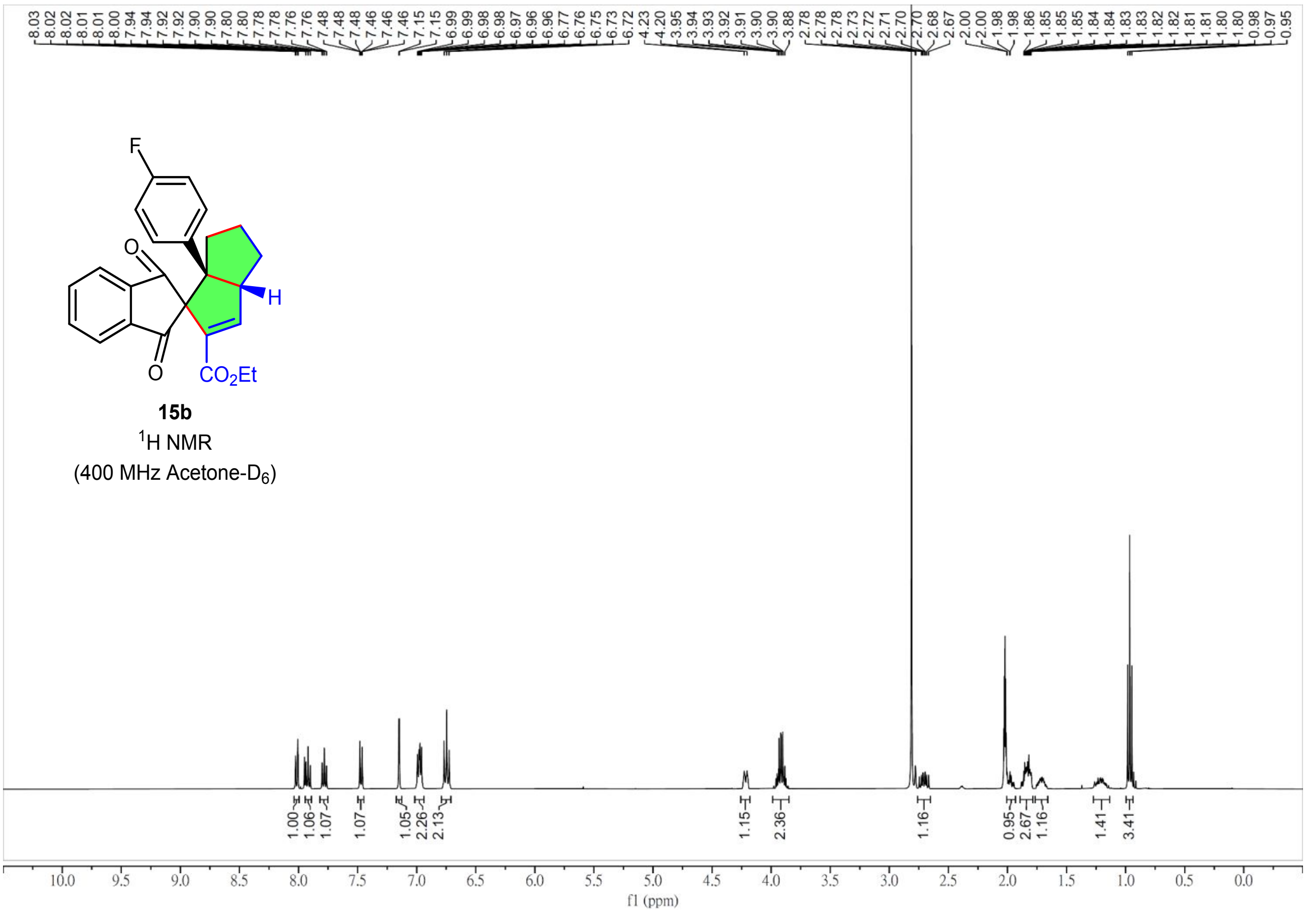
15b
1H NMR
(400 MHz Acetone-D6)
f1 (ppm)

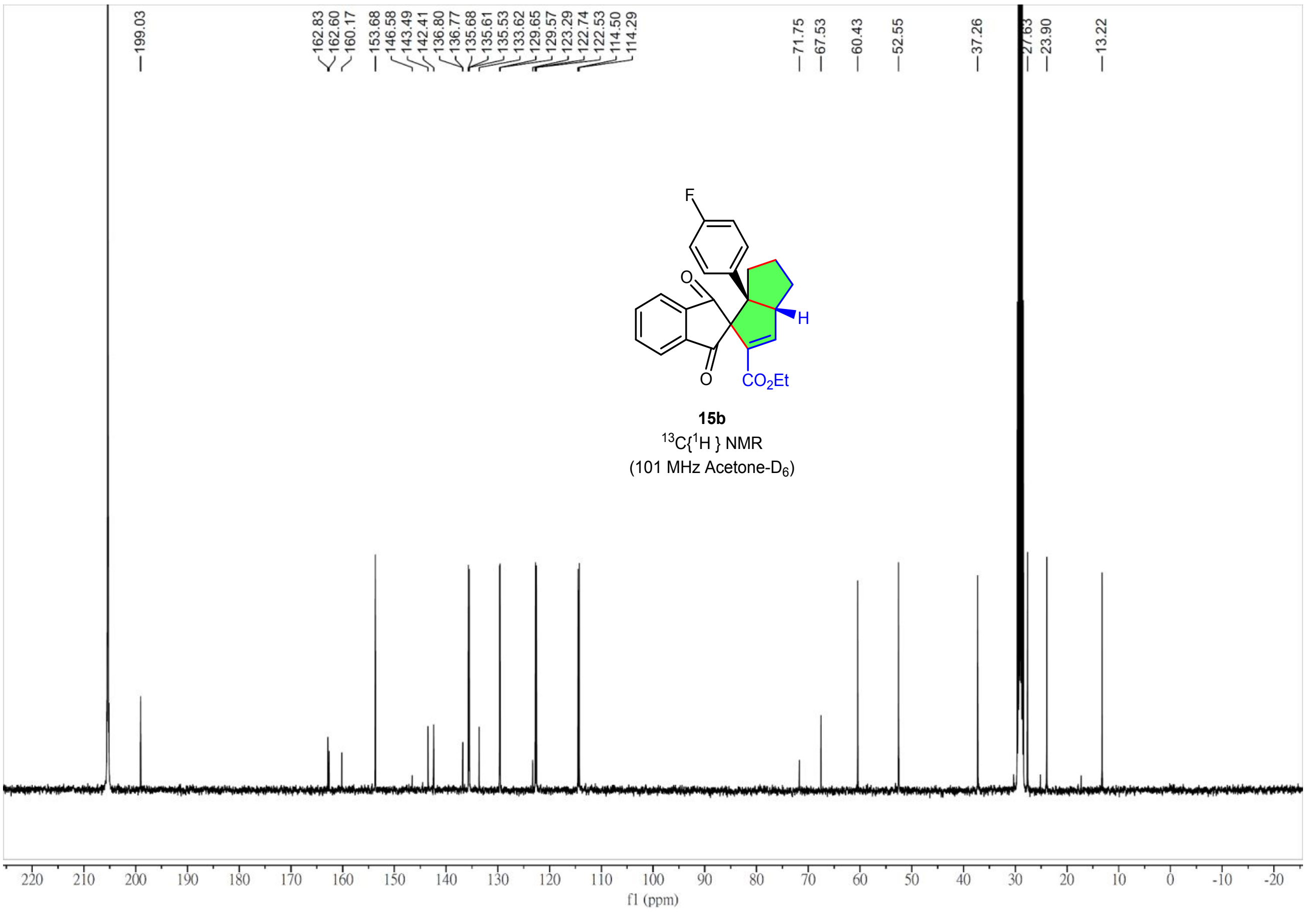
199.03
162.83
162.60
160.17
153.68
146.58
143.49
142.41
136.80
136.77
135.68
135.61
135.53
133.62
129.65
129.57
123.29
122.74
122.53
114.50
114.29
71.75
67.53
60.43
52.55
37.26
27.63
23.90
13.22
15b
13C{1H } NMR
(101 MHz Acetone-D6)
f1 (ppm)

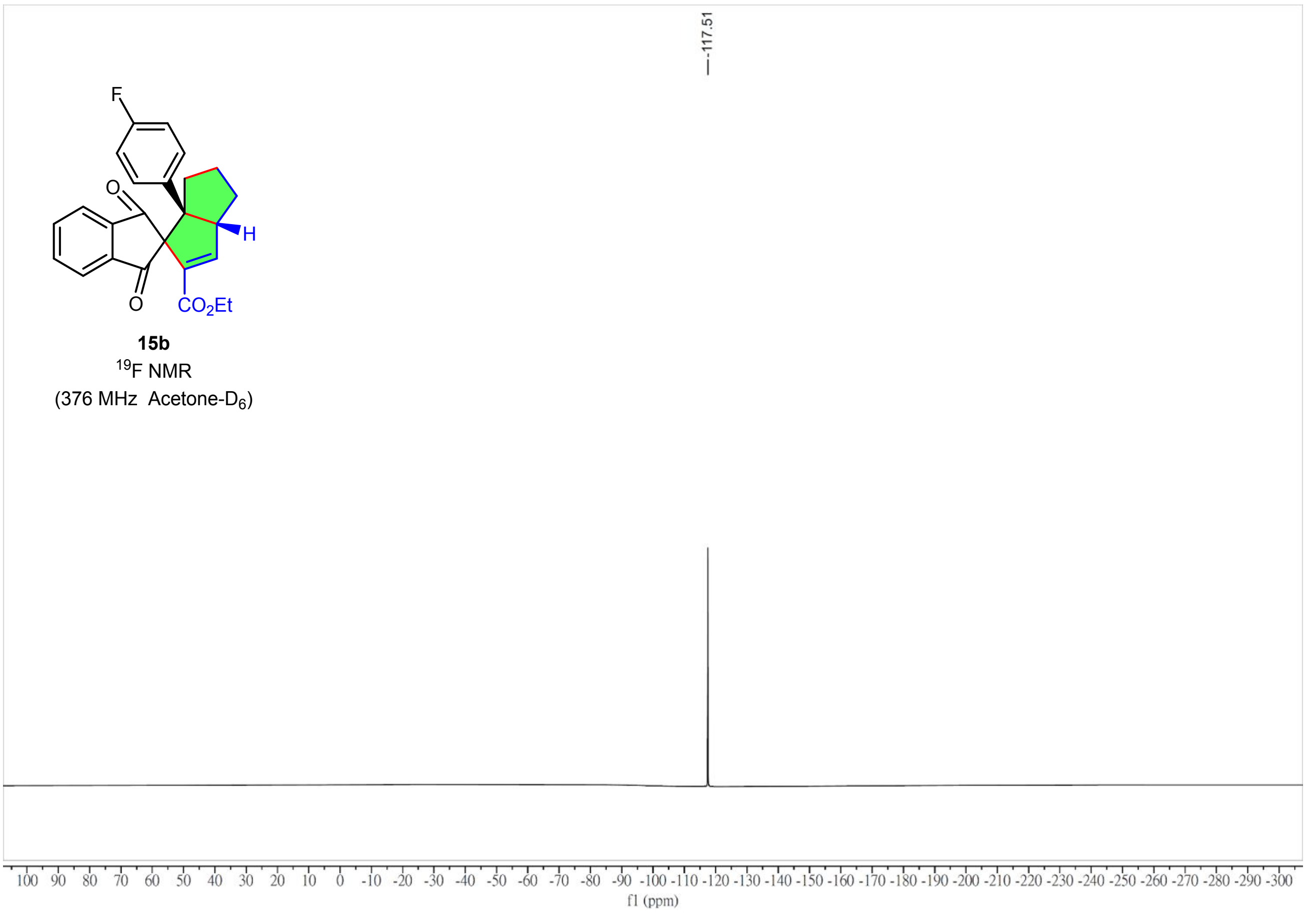

-117.51
F
O
H
O
CO2Et
15b
19F NMR
(376 MHz Acetone-D6)
100 90 80 70 60 50 40 30 20 10 0 -10 -20 -30 -40 -50 -60 -70 -80 -90 -100 -110 -120 -130 -140 -150 -160 -170 -180 -190 -200 -210 -220 -230 -240 -250 -260 -270 -280 -290 -300
f1 (ppm)

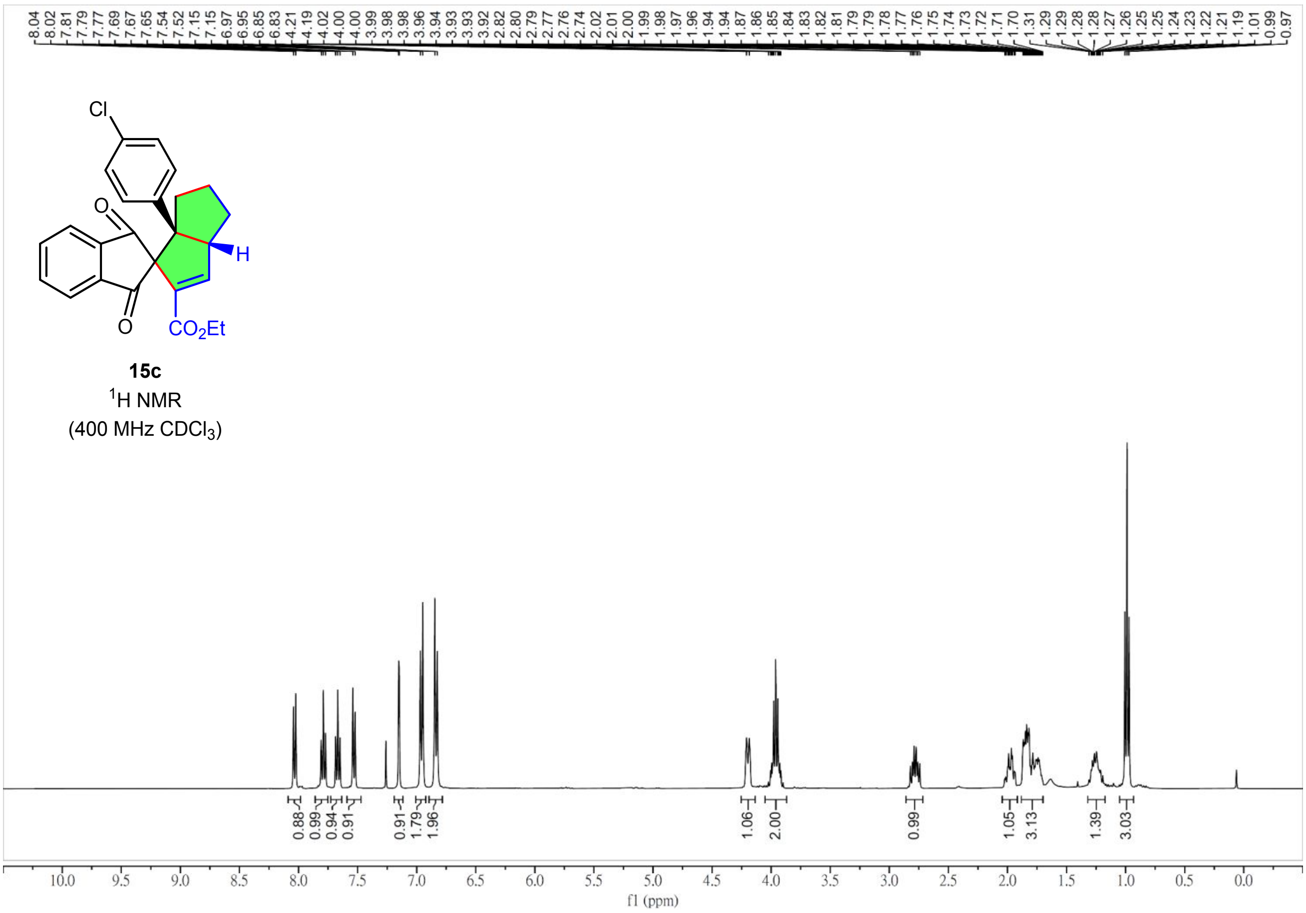

15c
^{1}H NMR
(400 MHz $CDCl_3$)

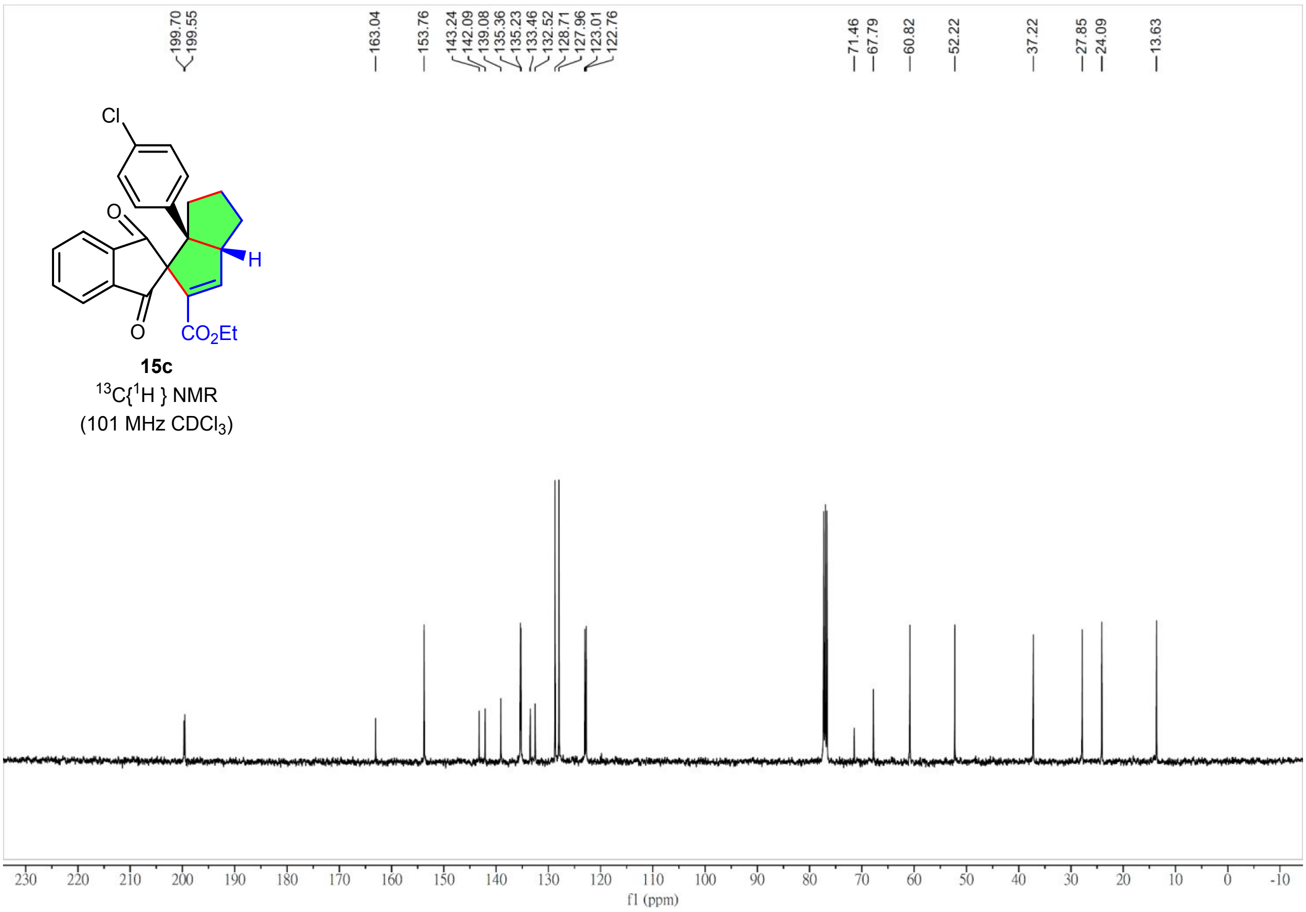

15c
$^{13}C\{^1H\}$ NMR
(101 MHz $CDCl_3$)

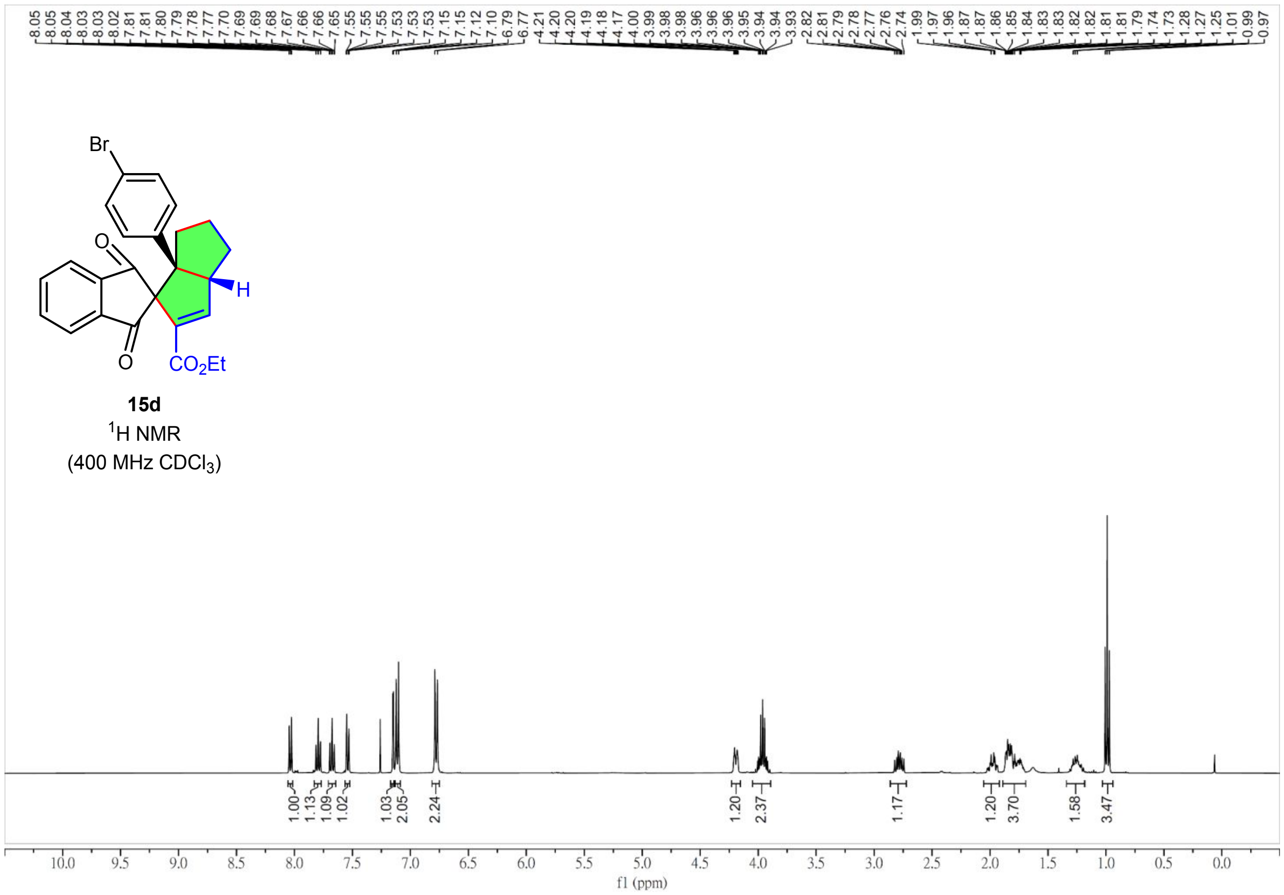
8.05 8.05 8.04 8.03 8.03 8.02 7.81 7.81 7.80 7.79 7.78 7.77 7.70 7.69 7.69 7.68 7.67 7.66 7.66 7.65 7.55 7.55 7.55 7.53 7.53 7.53 7.15 7.15 7.12 7.10 6.79 6.77 4.21 4.20 4.20 4.19 4.18 4.17 4.00 3.99 3.98 3.98 3.96 3.96 3.96 3.95 3.94 3.94 3.93 2.82 2.81 2.79 2.79 2.77 2.76 2.74 1.99 1.97 1.96 1.87 1.87 1.86 1.85 1.84 1.83 1.83 1.82 1.82 1.81 1.81 1.79 1.74 1.73 1.28 1.27 1.25 1.01 0.99 0.97
Br
O
H
O
CO_2Et
15d
^1H NMR
(400 MHz $CDCl_3$)
1.00 1.13 1.09 1.02 1.03 2.05 2.24 1.20 2.37 1.17 1.20 3.70 1.58 3.47
10.0 9.5 9.0 8.5 8.0 7.5 7.0 6.5 6.0 5.5 5.0 4.5 4.0 3.5 3.0 2.5 2.0 1.5 1.0 0.5 0.0
f1 (ppm)

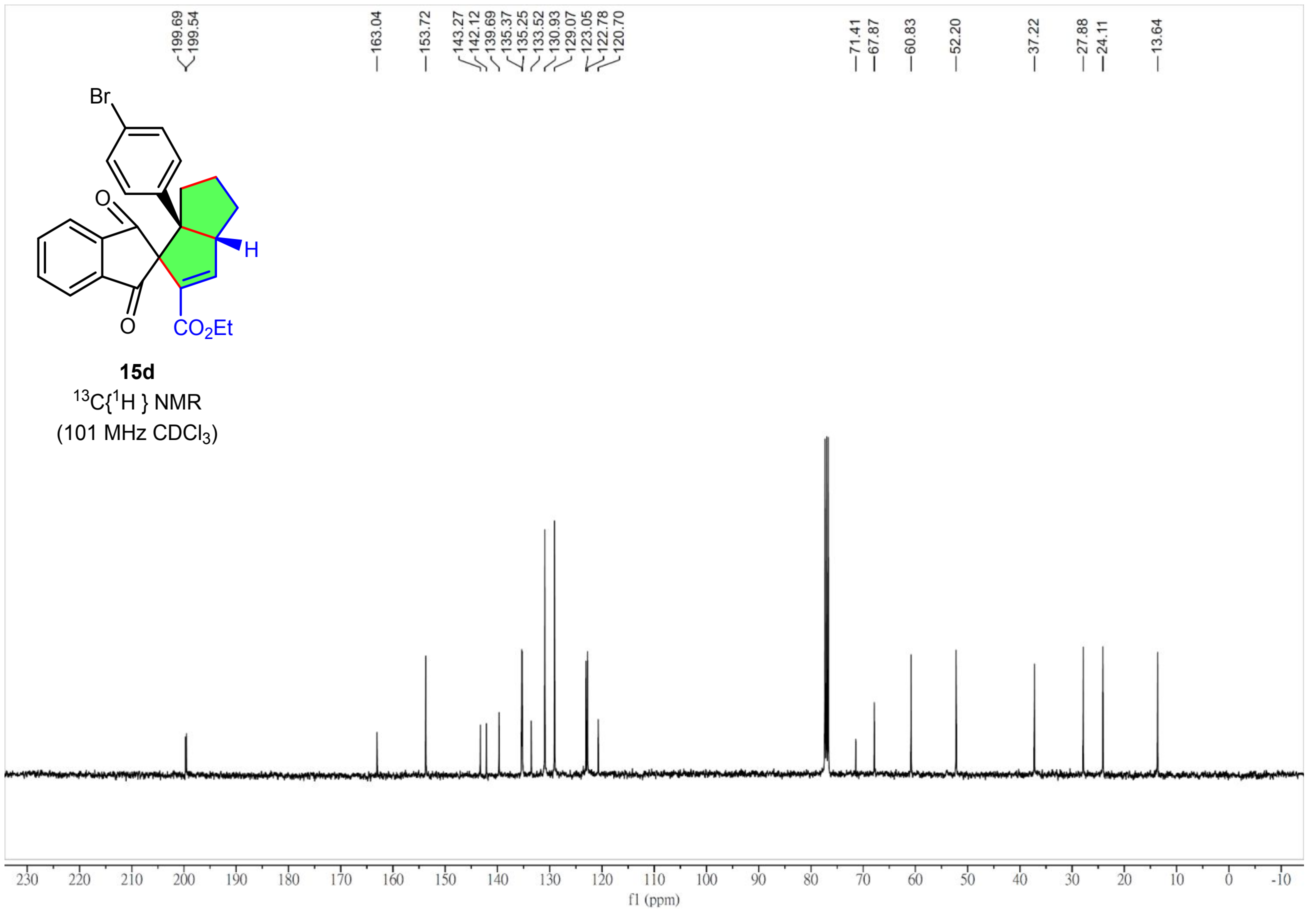
199.69 199.54 163.04 153.72 143.27 142.12 139.69 135.37 135.25 133.52 130.93 129.07 123.05 122.78 120.70 71.41 67.87 60.83 52.20 37.22 27.88 24.11 13.64
Br
O
H
O
CO_2Et
15d
$^{13}C\{^1H\}$ NMR
(101 MHz $CDCl_3$)
230 220 210 200 190 180 170 160 150 140 130 120 110 100 90 80 70 60 50 40 30 20 10 0 -10
f1 (ppm)

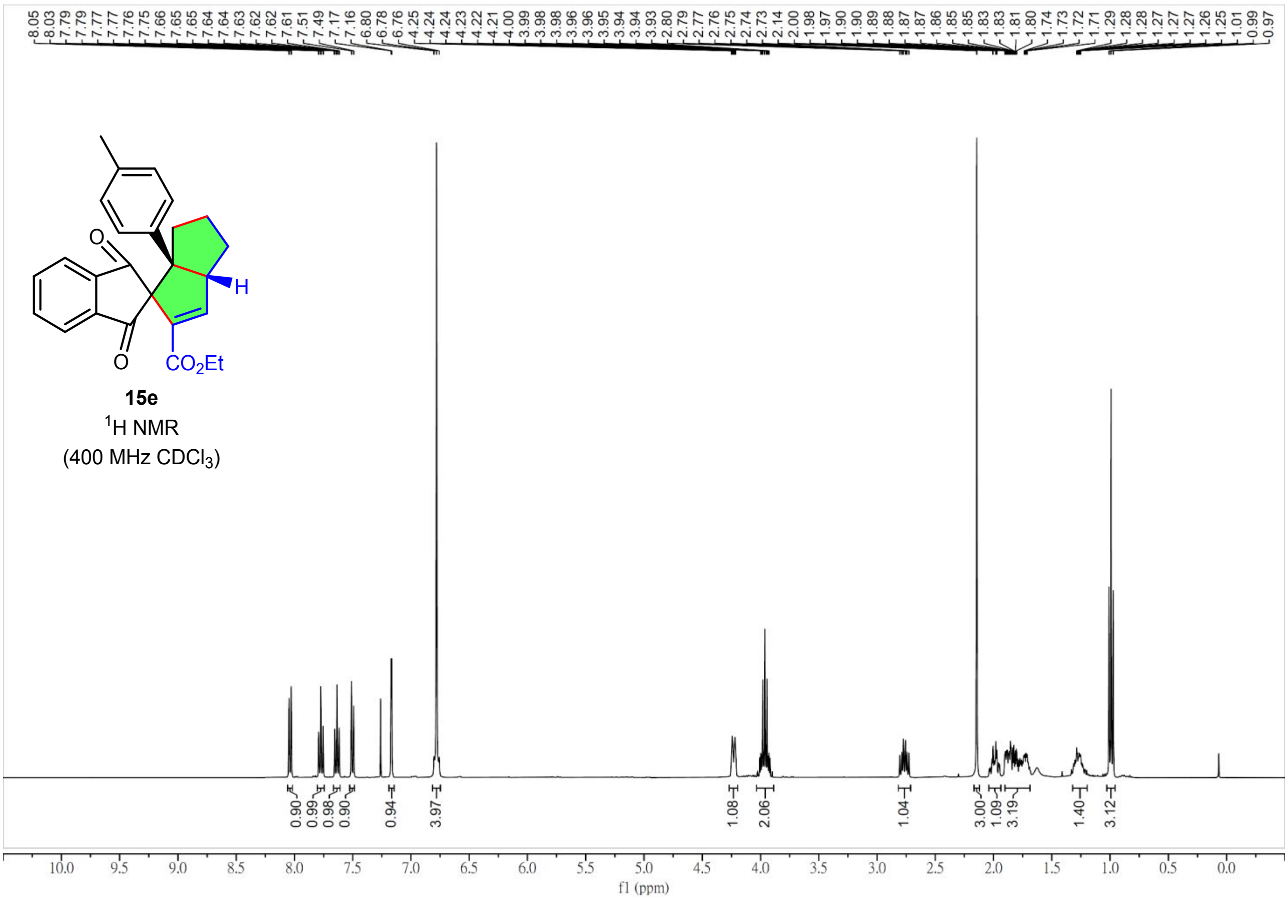
8.05 8.03 7.79 7.79 7.77 7.77 7.76 7.75 7.66 7.65 7.64 7.63 7.62 7.62 7.61 7.51 7.49 7.17 7.16 6.80 6.78 6.76 4.25 4.24 4.24 4.23 4.22 4.21 4.00 3.99 3.98 3.98 3.96 3.96 3.95 3.94 3.94 3.93 2.80 2.79 2.77 2.76 2.75 2.74 2.73 2.14 2.00 1.98 1.97 1.90 1.90 1.89 1.88 1.87 1.87 1.86 1.85 1.85 1.83 1.83 1.81 1.80 1.74 1.73 1.72 1.71 1.29 1.28 1.28 1.27 1.27 1.27 1.26 1.25 1.01 0.99 0.97
O
H
O
CO2Et
15e
1H NMR
(400 MHz CDCl3)
0.90 0.99 0.98 0.90 0.94 3.97 1.08 2.06 1.04 3.00 1.09 3.19 1.40 3.12
10.0 9.5 9.0 8.5 8.0 7.5 7.0 6.5 6.0 5.5 5.0 4.5 4.0 3.5 3.0 2.5 2.0 1.5 1.0 0.5 0.0
f1 (ppm)

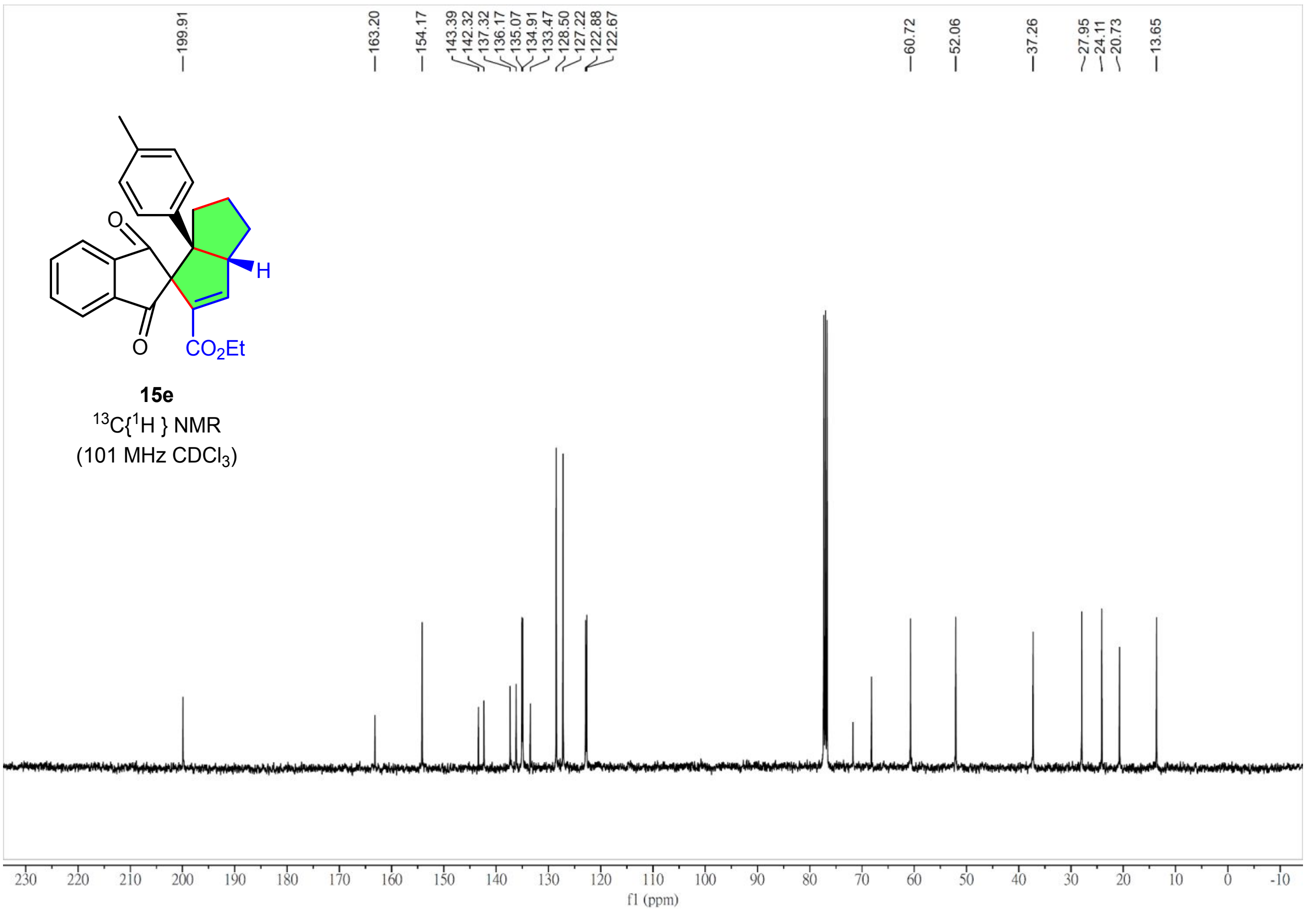
199.91
163.20
154.17
143.39 142.32 137.32 136.17 135.07 134.91 133.47 128.50 127.22 122.88 122.67
60.72
52.06
37.26
27.95 24.11 20.73
13.65
O
H
O
CO2Et
15e
13C{1H } NMR
(101 MHz CDCl3)
230 220 210 200 190 180 170 160 150 140 130 120 110 100 90 80 70 60 50 40 30 20 10 0 -10
f1 (ppm)

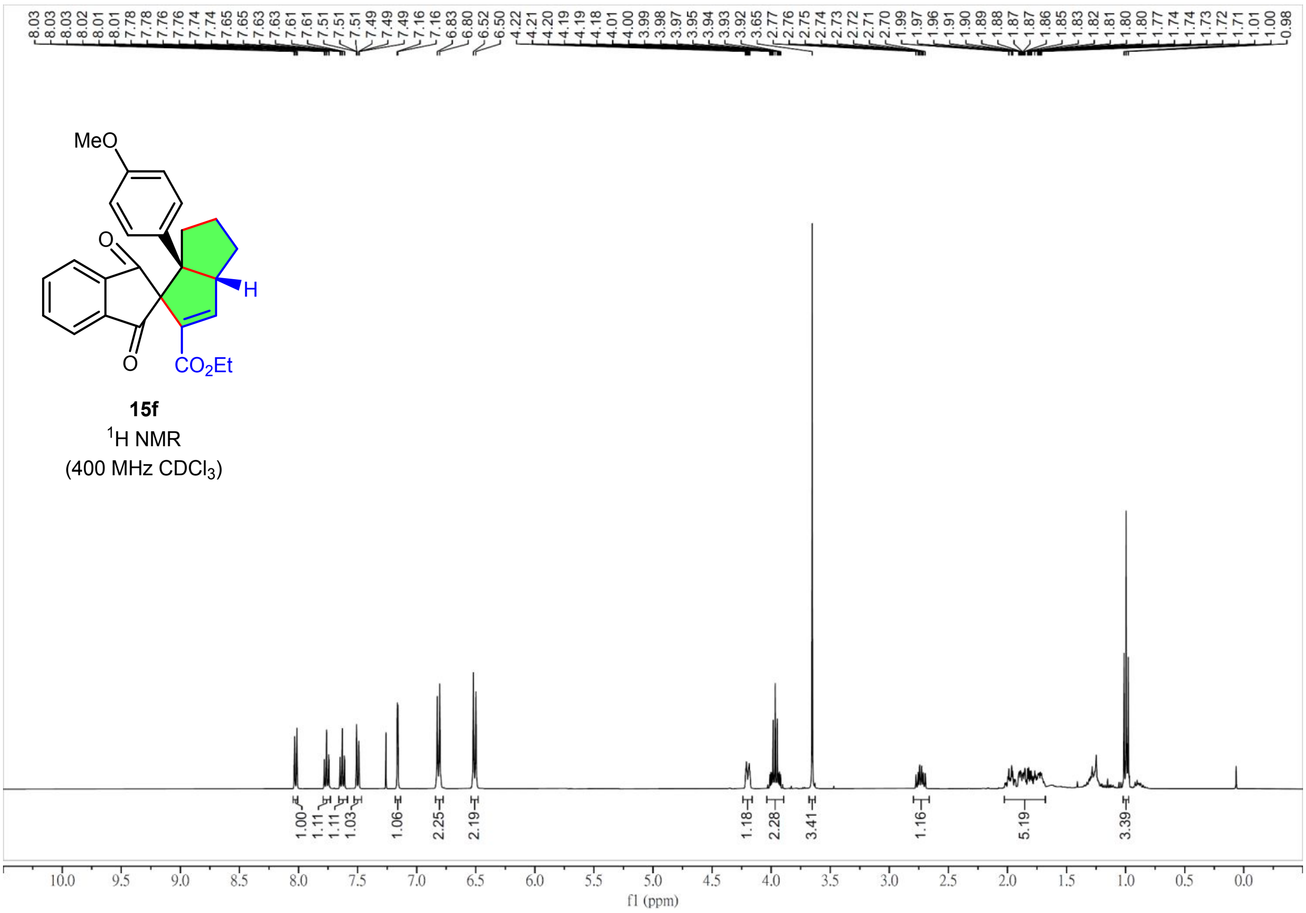

8.03 8.03 8.02 8.01 8.01 7.78 7.78 7.76 7.76 7.74 7.74 7.65 7.65 7.63 7.63 7.61 7.61 7.51 7.51 7.51 7.49 7.49 7.16 7.16 6.83 6.80 6.52 6.50 4.22 4.21 4.20 4.19 4.19 4.18 4.01 4.00 3.99 3.98 3.97 3.95 3.94 3.93 3.92 3.65 2.77 2.76 2.75 2.74 2.73 2.72 2.71 2.70 1.99 1.97 1.96 1.91 1.90 1.89 1.88 1.87 1.87 1.86 1.85 1.83 1.82 1.81 1.80 1.77 1.74 1.74 1.73 1.72 1.71 1.01 1.00 0.98
MeO
O
H
O
CO2Et
15f
1H NMR
(400 MHz CDCl3)
1.00 1.11 1.11 1.03 1.06 2.25 2.19 1.18 2.28 3.41 1.16 5.19 3.39
10.0 9.5 9.0 8.5 8.0 7.5 7.0 6.5 6.0 5.5 5.0 4.5 4.0 3.5 3.0 2.5 2.0 1.5 1.0 0.5 0.0
f1 (ppm)

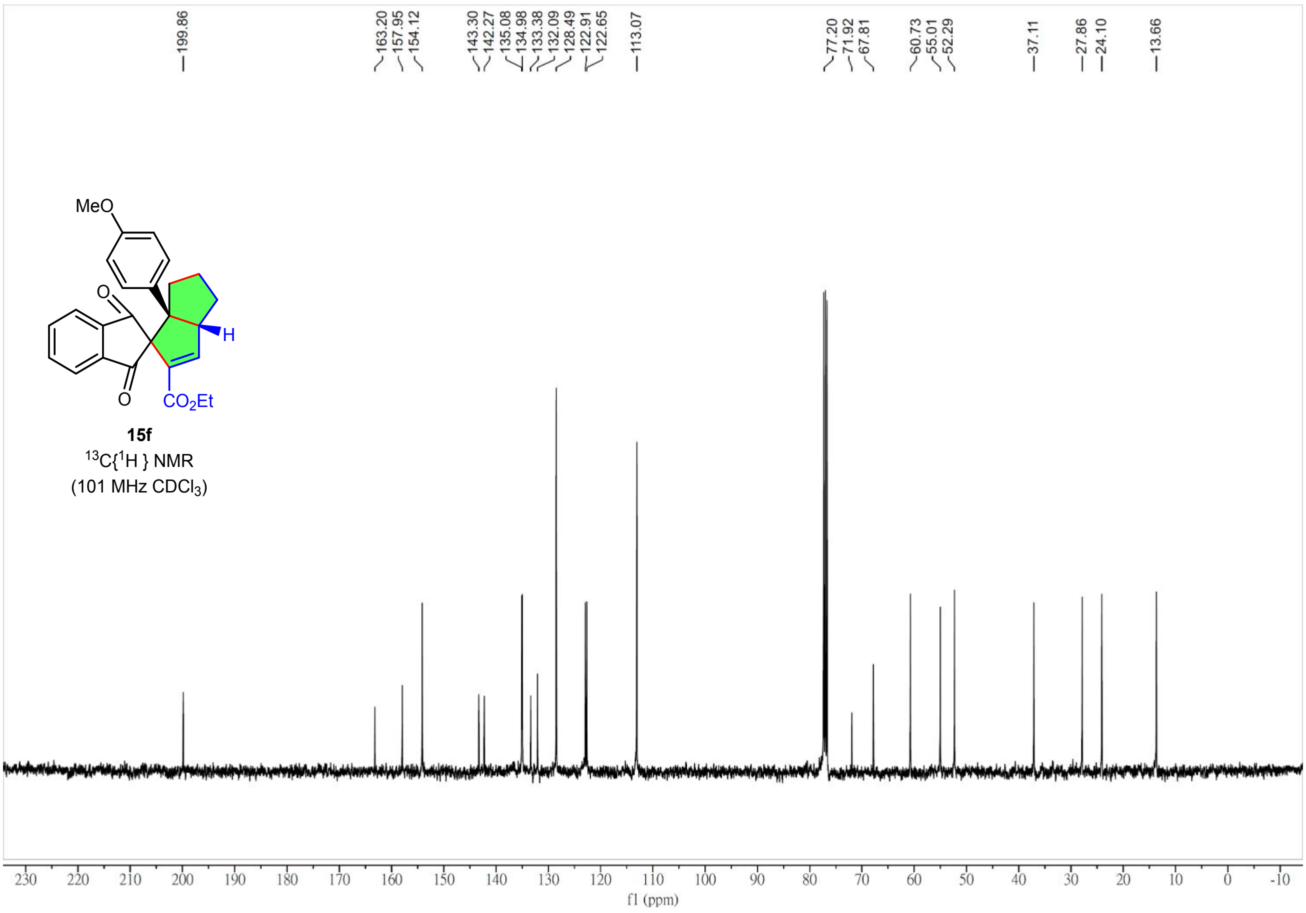

199.86 163.20 157.95 154.12 143.30 142.27 135.08 134.98 133.38 132.09 128.49 122.91 122.65 113.07 77.20 71.92 67.81 60.73 55.01 52.29 37.11 27.86 24.10 13.66
MeO
O
H
O
CO2Et
15f
13C{1H } NMR
(101 MHz CDCl3)
230 220 210 200 190 180 170 160 150 140 130 120 110 100 90 80 70 60 50 40 30 20 10 0 -10
f1 (ppm)

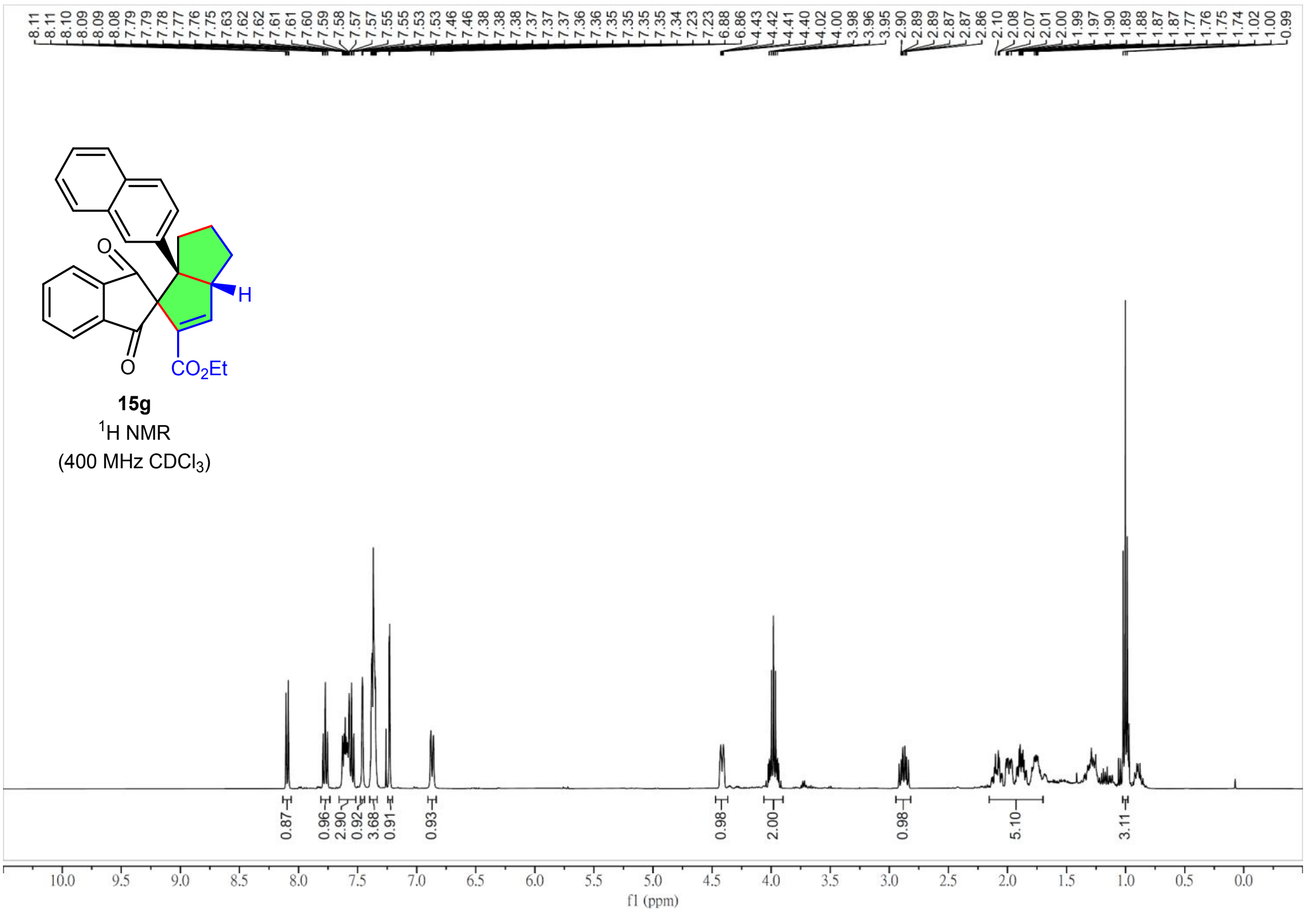
15g
1H NMR
(400 MHz CDCl3)
f1 (ppm)

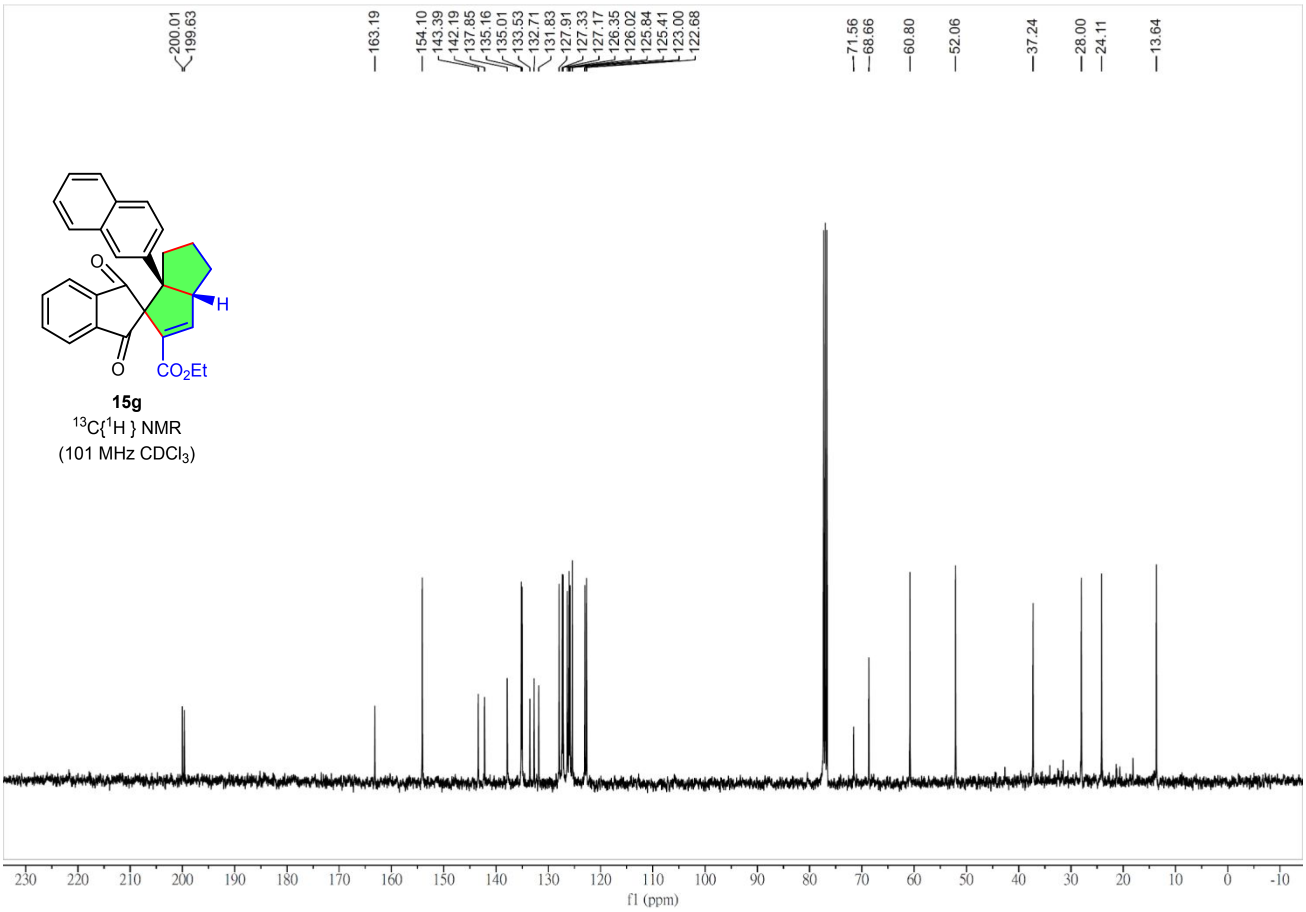
15g
13C{1H } NMR
(101 MHz CDCl3)
f1 (ppm)

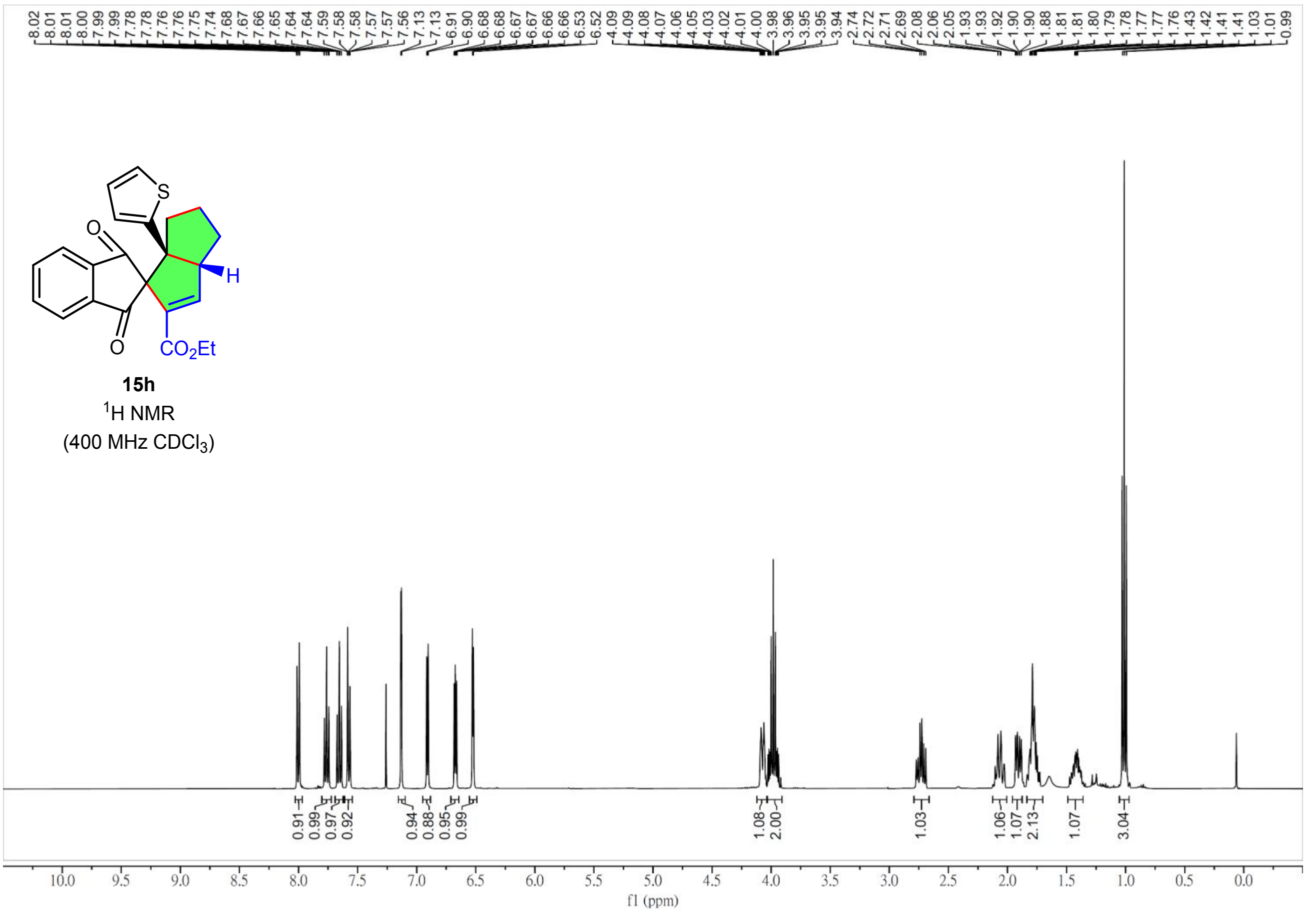

15h
^1H NMR
(400 MHz $CDCl_3$)

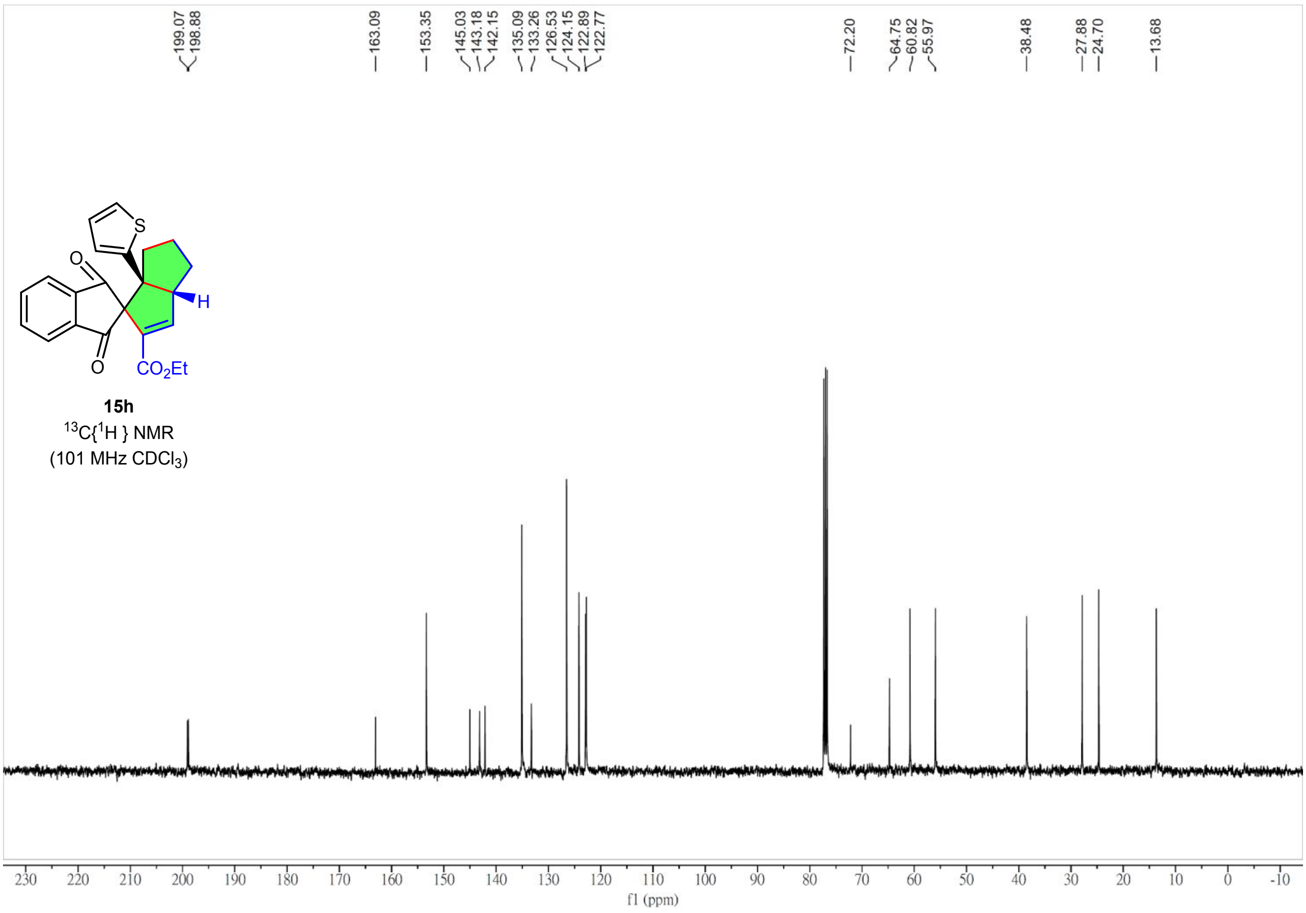

15h
$^{13}C\{^1H\}$ NMR
(101 MHz $CDCl_3$)

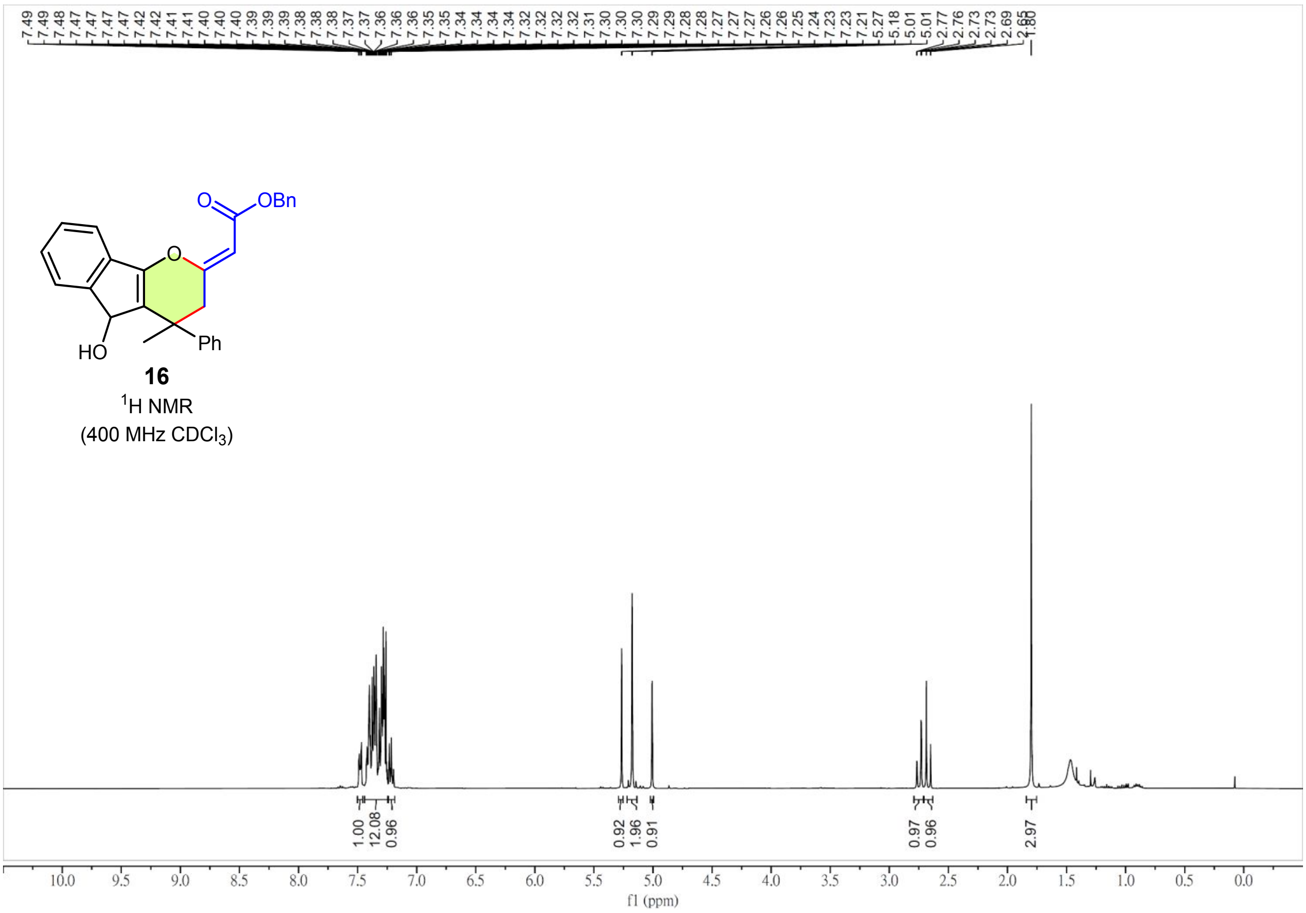
7.49 7.49 7.48 7.47 7.47 7.47 7.42 7.42 7.41 7.41 7.40 7.40 7.39 7.39 7.39 7.38 7.38 7.38 7.37 7.36 7.36 7.36 7.35 7.35 7.34 7.34 7.34 7.32 7.32 7.32 7.32 7.31 7.30 7.30 7.29 7.29 7.28 7.28 7.27 7.27 7.27 7.26 7.25 7.24 7.23 7.23 7.21 5.27 5.18 5.01 5.01 2.77 2.76 2.73 2.69 2.65 1.80
O
OBn
O
HO
Ph
16
1H NMR
(400 MHz CDCl3)
1.00 12.08 0.96 0.92 1.96 0.91 0.97 0.96 2.97
f1 (ppm)

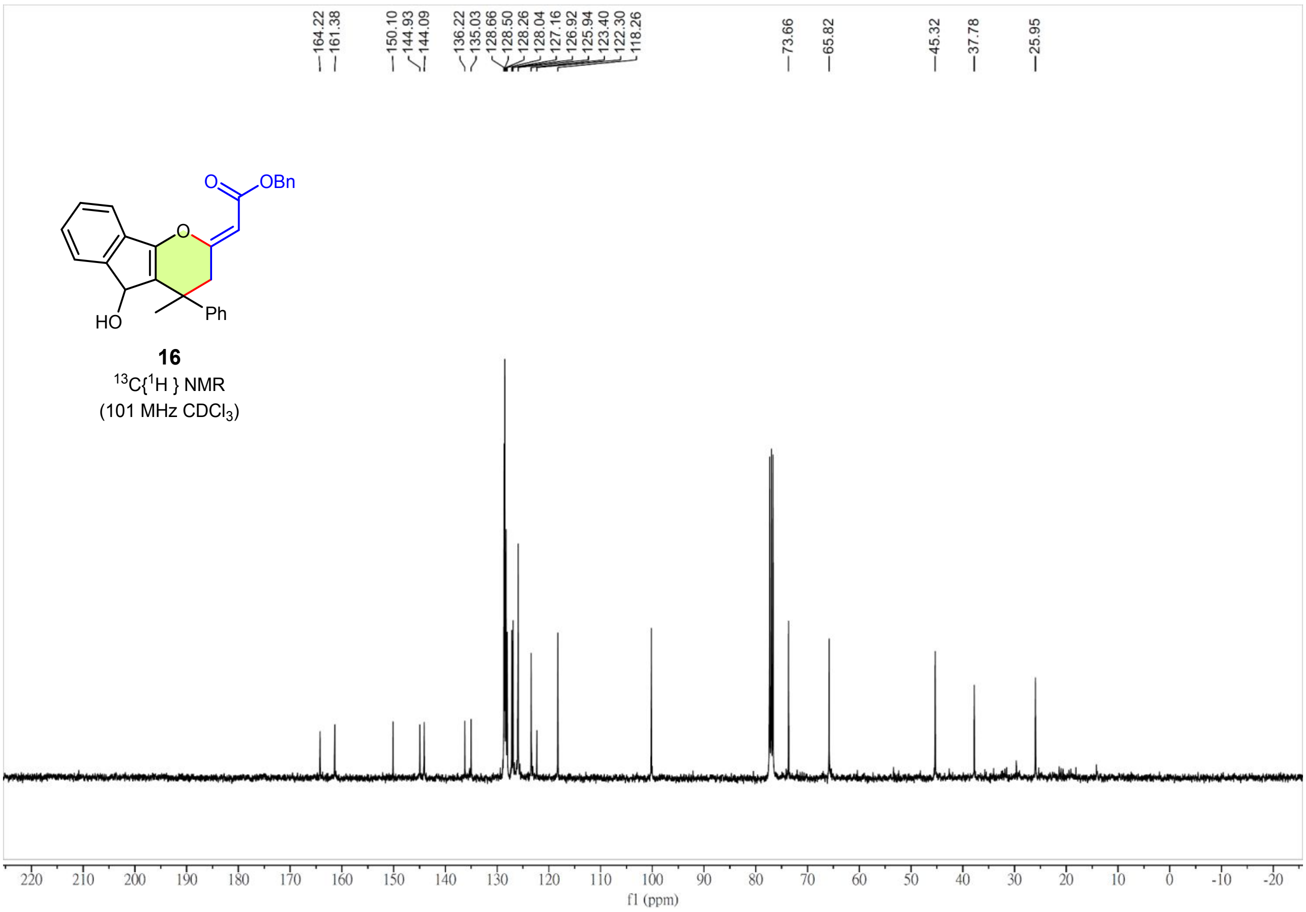
164.22 161.38 150.10 144.93 144.09 136.22 135.03 128.66 128.50 128.26 128.04 127.16 126.92 125.94 123.40 122.30 118.26 73.66 65.82 45.32 37.78 25.95
O
OBn
O
HO
Ph
16
13C{1H } NMR
(101 MHz CDCl3)
f1 (ppm)

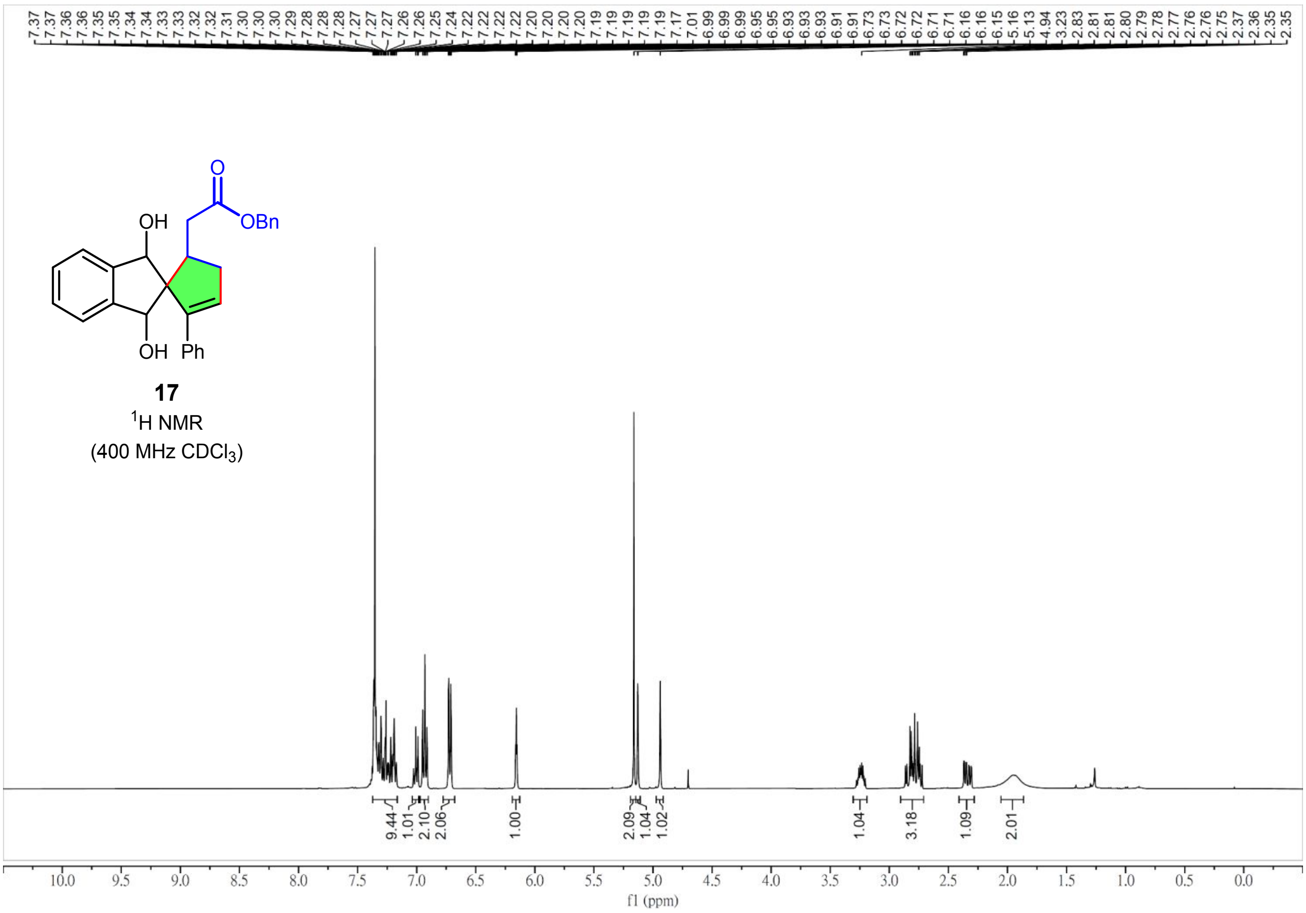

17
^{1}H NMR
(400 MHz $CDCl_3$)

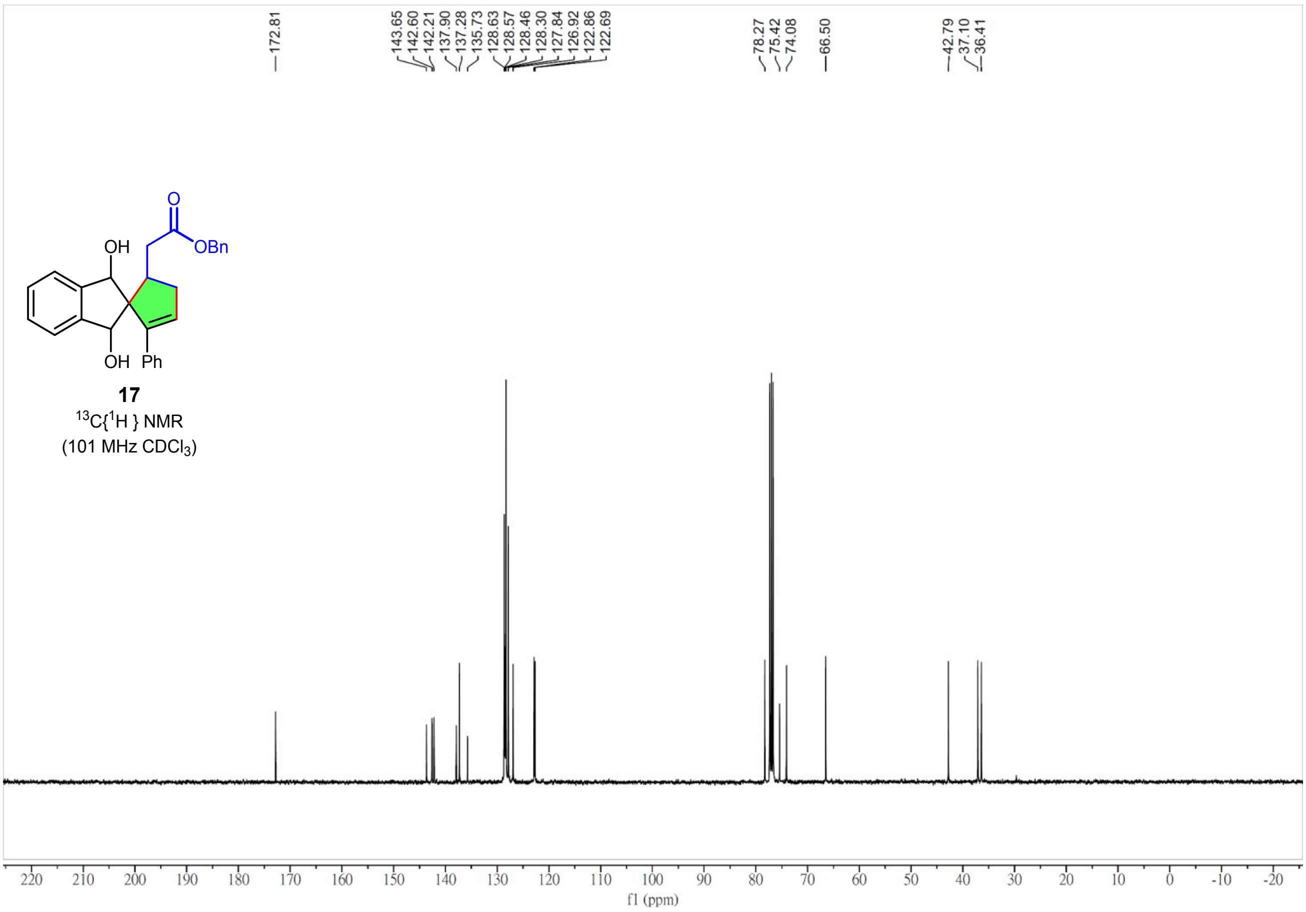

17
$^{13}C\{^{1}H\}$ NMR
(101 MHz $CDCl_3$)

9. Cartesian Coordinates

A+10a

Optimization energy :

E(M06-2X/6-31G(d,p)/IEFPCM(Toluene, SAS)) = -1531.650746 Hartree

Single point energy :

E(M06-2X-D3/def2-TZVPP/SMD(Toluene, SAS))= -1532.252795 Hartree

Thermal correction to Gibbs Free Energy= 0.0 kcal/mol

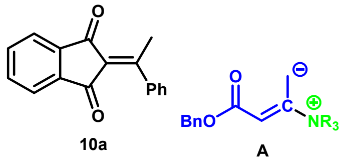

Symbol	X	Y	Z
C	-1.16158	-0.99582	-1.72424
C	-2.07095	-0.80542	-0.73534
C	-2.72524	-1.64595	0.194002
C	-2.79769	-3.06836	0.143059
O	-3.32859	-3.79356	0.975911
O	-2.21994	-3.60921	-0.98158
C	-2.44395	-4.99467	-1.14185
H	-3.49782	-5.19888	-1.36413
H	-1.82393	-5.30794	-1.9851
H	-2.17221	-5.55357	-0.24337
H	-3.17265	-1.20242	1.076533
H	-0.93159	-0.20351	-2.42827
H	-0.76974	-1.98514	-1.90619
C	-1.48477	1.561127	-0.44407
C	-1.78199	2.883624	-0.24038
C	-3.13249	3.297519	-0.13491
C	-4.11622	2.279115	-0.24444
C	-3.73945	0.975363	-0.43677
N	-2.44315	0.616971	-0.54286
H	-0.4652	1.203054	-0.50592
H	-0.95036	3.566544	-0.1305
H	-5.17237	2.502255	-0.17911
H	-4.44285	0.15446	-0.51318
N	-3.46692	4.590284	0.069568
C	-4.86295	4.969432	0.202722

H	-4.92561	6.04348	0.372112
H	-5.42765	4.735027	-0.70678
H	-5.33439	4.462362	1.052407
C	-2.42415	5.59647	0.185017
H	-2.88608	6.573084	0.323193
H	-1.77245	5.399054	1.043642
H	-1.81031	5.633917	-0.72157
C	3.606738	1.901916	0.07032
C	4.375708	0.764128	-0.13166
C	5.718573	0.849148	-0.46869
C	6.269799	2.122238	-0.60333
C	5.493248	3.270471	-0.4031
C	4.145161	3.174116	-0.06416
C	2.213092	1.504056	0.42664
C	3.535334	-0.46235	0.059295
H	6.304579	-0.05135	-0.62305
H	7.316831	2.230531	-0.86937
H	5.953062	4.247702	-0.51372
H	3.530038	4.05367	0.097649
O	1.307834	2.310672	0.584013
O	3.976283	-1.58816	-0.02629
C	2.165681	0.014056	0.445824
C	1.114989	-0.74246	0.906966
C	0.969722	-2.18812	0.660114
C	0.202327	-2.9844	1.525693
C	1.48826	-2.78183	-0.50251
C	0.020189	-4.33985	1.277601
H	-0.25817	-2.55215	2.40666
C	1.301873	-4.13133	-0.75425
H	2.008688	-2.16742	-1.22586
C	0.580698	-4.91736	0.143627
H	-0.59021	-4.92945	1.953396
H	1.70817	-4.56979	-1.66015
H	0.432285	-5.97448	-0.058
C	0.07085	-0.12573	1.797137
H	0.267584	-0.46937	2.821619
H	0.113173	0.960183	1.800285
H	-0.92992	-0.48331	1.533473

TS-1

Optimization energy :

E(M06-2X/6-31G(d,p)/IEFPCM(Toluene, SAS)) = -1531.643433 Hartree

Number of imaginary frequency=1 (-230.98)

Single point energy :

E(M06-2X-D3/def2-TZVPP/SMD(Toluene, SAS))= -1532.243178 Hartree

Thermal correction to Gibbs Free Energy= 6.03 kcal/mol

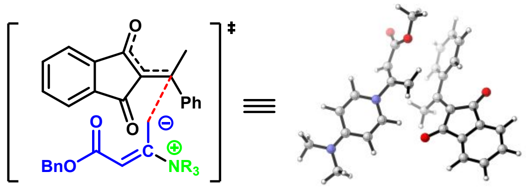

Symbol	X	Y	Z
C	0.193201	0.647394	-1.09616
C	1.315682	1.258488	-0.55748
C	1.523118	2.544711	-0.0735
C	0.807029	3.751176	-0.42802
O	1.082647	4.856762	0.003145
O	-0.1737	3.567851	-1.34963
C	-0.73775	4.76698	-1.85713
H	-0.00978	5.297847	-2.48019
H	-1.59431	4.464156	-2.4611
H	-1.05637	5.426586	-1.04897
H	2.347262	2.73013	0.605747
H	0.281158	-0.32216	-1.57538
H	-0.60396	1.288458	-1.43538
C	2.308625	-0.94876	-0.06331
C	3.375327	-1.79235	0.105211
C	4.702959	-1.31259	-0.00782
C	4.839874	0.062003	-0.33581
C	3.730323	0.846117	-0.49921
N	2.473271	0.365085	-0.34659
H	1.288166	-1.31506	0.020312
H	3.154933	-2.82669	0.332759
H	5.809688	0.516756	-0.48474
H	3.794824	1.88905	-0.78009
N	5.772605	-2.11289	0.172641
C	7.116778	-1.57343	0.046886
H	7.837585	-2.35886	0.269147

H	7.308734	-1.21065	-0.9694
H	7.279969	-0.7528	0.753895
C	5.579405	-3.52235	0.476354
H	6.552381	-3.99979	0.582432
H	5.029582	-3.6524	1.41485
H	5.034222	-4.02994	-0.32692
C	-2.49162	-3.19474	-0.16883
C	-3.69646	-2.50953	-0.09498
C	-4.90467	-3.14738	-0.31104
C	-4.87139	-4.51166	-0.61455
C	-3.65875	-5.20053	-0.69323
C	-2.44475	-4.54502	-0.47034
C	-1.3786	-2.23783	0.129853
C	-3.43014	-1.0623	0.233072
H	-5.83722	-2.59506	-0.2472
H	-5.79927	-5.04674	-0.79386
H	-3.6635	-6.26048	-0.92969
H	-1.49416	-5.06675	-0.52464
O	-0.19022	-2.57205	0.100037
O	-4.32898	-0.25536	0.394963
C	-1.96505	-0.92889	0.373845
C	-1.20981	0.183296	0.818232
C	-1.74958	1.558506	0.804786
C	-1.25199	2.53517	1.680028
C	-2.71632	1.953146	-0.13979
C	-1.73408	3.843719	1.64757
H	-0.49432	2.281552	2.411546
C	-3.20851	3.244467	-0.1594
H	-3.0697	1.234311	-0.86781
C	-2.72242	4.196952	0.741276
H	-1.32266	4.582835	2.327101
H	-3.96553	3.519897	-0.88755
H	-3.10496	5.213571	0.719532
C	-0.13359	-0.08728	1.84592
H	-0.58832	0.056091	2.835381
H	0.214338	-1.11691	1.798744
H	0.710992	0.605053	1.776369

B

Optimization energy :

E(M06-2X/6-31G(d,p)/IEFPCM(Toluene, SAS)) = -1531.67408 Hartree

Single point energy :

E(M06-2X-D3/def2-TZVPP/SMD(Toluene, SAS))= -1532.273274 Hartree

Thermal correction to Gibbs Free Energy= -12.85 kcal/mol

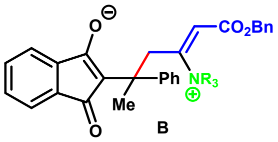

B

Symbol	X	Y	Z
C	0.010614	-0.54917	-0.8302
C	-1.21915	-1.34058	-0.50119
C	-1.30945	-2.65802	-0.24934
C	-0.34545	-3.74588	-0.55696
O	-0.47257	-4.84743	-0.07609
O	0.561811	-3.43591	-1.48584
C	1.412976	-4.51536	-1.87551
H	0.837202	-5.25363	-2.44137
H	2.184342	-4.07049	-2.50257
H	1.856577	-4.98762	-0.99949
H	-2.19476	-3.06923	0.223135
H	-0.25451	0.379868	-1.33447
H	0.617944	-1.13752	-1.51679
C	-2.40244	0.773738	-0.07697
C	-3.54193	1.504255	0.128433
C	-4.82051	0.897182	0.088198
C	-4.83534	-0.48361	-0.26029
C	-3.66619	-1.15486	-0.46166
N	-2.44552	-0.5658	-0.32159
H	-1.42946	1.281122	-0.09217
H	-3.41345	2.563141	0.308865
H	-5.76123	-1.02222	-0.40954
H	-3.66668	-2.18652	-0.78195
N	-5.95055	1.581899	0.325629
C	-7.24296	0.91628	0.24892
H	-8.02094	1.616096	0.549552
H	-7.45979	0.579302	-0.77104
H	-7.28009	0.05699	0.926104
C	-5.88556	3.007225	0.621199

H	-6.8921	3.376616	0.810237
H	-5.27803	3.192999	1.512594
H	-5.46447	3.5671	-0.22062
C	2.143115	3.299857	-0.38206
C	3.349332	2.758236	0.048232
C	4.502569	3.512279	0.090793
C	4.423311	4.851401	-0.322
C	3.215627	5.39391	-0.75271
C	2.048063	4.616338	-0.78446
C	1.0923	2.225086	-0.28336
C	3.111432	1.309104	0.434167
H	5.433869	3.074028	0.437948
H	5.312474	5.475516	-0.30317
H	3.178652	6.434073	-1.0647
H	1.097481	5.027937	-1.11119
O	-0.12142	2.463737	-0.50872
O	3.988127	0.611771	0.93262
C	1.724999	1.025479	0.11535
C	0.925629	-0.21946	0.444322
C	1.797191	-1.46048	0.653945
C	1.515418	-2.41197	1.632987
C	2.872437	-1.70818	-0.21141
C	2.287287	-3.57097	1.758664
H	0.690312	-2.26325	2.321983
C	3.654128	-2.84397	-0.07802
H	3.105399	-0.97751	-0.98043
C	3.365551	-3.78519	0.913604
H	2.038297	-4.29895	2.524929
H	4.49957	-2.99619	-0.7432
H	3.977681	-4.6765	1.019119
C	0.097307	0.082026	1.711048
H	0.774057	0.09701	2.570316
H	-0.35438	1.073871	1.643882
H	-0.69099	-0.65431	1.917547

TS-2

Optimization energy :

E(M06-2X/6-31G(d,p)/IEFPCM(Toluene, SAS)) = -1531.633307 Hartree

Number of imaginary frequency=1 (-1247.15)

Single point energy :

E(M06-2X-D3/def2-TZVPP/SMD(Toluene, SAS))= -1532.233873 Hartree

Thermal correction to Gibbs Free Energy= 11.87 kcal/mol

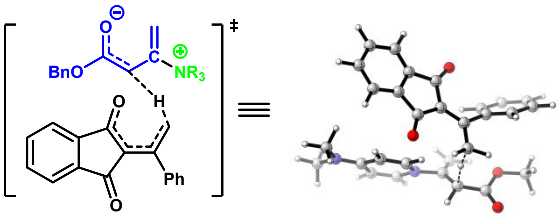

Symbol	X	Y	Z
C	1.53724	-2.15345	-1.73748
C	1.072786	-2.33436	-0.49011
C	1.725151	-2.38276	0.805759
C	3.176781	-2.37184	0.960773
O	3.771424	-2.7746	1.939814
O	3.81212	-1.71861	-0.04534
C	5.198215	-1.491	0.17035
H	5.735044	-2.43659	0.286233
H	5.549943	-0.95299	-0.71002
H	5.355181	-0.88468	1.066437
H	1.248577	-3.02847	1.540709
H	0.860974	-1.98442	-2.56895
H	2.600315	-2.10877	-1.92148
C	-1.039	-1.63646	0.524065
C	-2.40473	-1.57083	0.573342
C	-3.18975	-2.33086	-0.32993
C	-2.47907	-3.1755	-1.22605
C	-1.11004	-3.1804	-1.22501
N	-0.39131	-2.40587	-0.38163
H	-0.43158	-1.0506	1.202051
H	-2.8313	-0.90314	1.310848
H	-2.9919	-3.8232	-1.92388
H	-0.52697	-3.80065	-1.89397
N	-4.53389	-2.25717	-0.3335
C	-5.30676	-3.05449	-1.2714

H	-6.36682	-2.86447	-1.11064
H	-5.06549	-2.79504	-2.30866
H	-5.12797	-4.12487	-1.12151
C	-5.20805	-1.33014	0.565277
H	-6.28069	-1.37823	0.383264
H	-5.02444	-1.59159	1.612999
H	-4.87227	-0.30157	0.391812
C	-2.18025	2.271365	0.807059
C	-1.67745	2.973114	-0.28062
C	-2.47986	3.822237	-1.02294
C	-3.81832	3.950934	-0.64118
C	-4.32365	3.248519	0.456233
C	-3.50328	2.395331	1.200141
C	-1.08631	1.42723	1.392661
C	-0.21757	2.633773	-0.45622
H	-2.0701	4.364829	-1.86948
H	-4.47728	4.609271	-1.19944
H	-5.36487	3.377975	0.737079
H	-3.87365	1.857574	2.069146
O	-1.2753	0.665372	2.343238
O	0.473375	3.126766	-1.32841
C	0.116178	1.655111	0.600317
C	1.381638	1.126424	0.900818
C	2.562335	1.413811	0.032532
C	3.744093	1.88717	0.609855
C	2.545165	1.138734	-1.33796
C	4.879998	2.101239	-0.16684
H	3.764685	2.107369	1.673592
C	3.685198	1.324365	-2.10834
H	1.633562	0.764105	-1.79103
C	4.854556	1.814079	-1.52761
H	5.784282	2.487008	0.294459
H	3.659763	1.095015	-3.16942
H	5.741028	1.971094	-2.13536
C	1.623618	0.218387	1.964154
H	2.662606	0.140592	2.28963
H	0.890428	0.171219	2.765296
H	1.569165	-1.00528	1.381789

D-1$_{DMAP}$+10a-1

Optimization energy :

E(M06-2X/6-31G(d,p)/IEFPCM(Toluene, SAS)) = -1531.673274 Hartree

Single point energy :

E(M06-2X-D3/def2-TZVPP/SMD(Toluene, SAS))= -1532.279121 Hartree

Thermal correction to Gibbs Free Energy= -16.52 kcal/mol

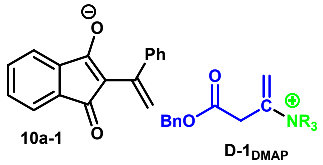

Symbol	X	Y	Z
C	3.237489	0.709571	0.799179
C	2.557049	1.019013	-0.29817
C	3.020712	0.863188	-1.72662
C	4.519495	0.667484	-1.81041
O	5.312305	1.533449	-2.07505
O	4.85954	-0.60109	-1.54259
C	6.262073	-0.87075	-1.5869
H	6.79352	-0.25803	-0.85465
H	6.366906	-1.92823	-1.34956
H	6.658456	-0.65982	-2.58244
H	2.774991	1.760968	-2.30031
H	2.797189	0.774688	1.789875
H	4.249625	0.329473	0.726409
C	0.223089	1.098931	-0.97869
C	-1.06503	1.533074	-0.8432
C	-1.41531	2.45377	0.175759
C	-0.34034	2.946063	0.972732
C	0.928104	2.478874	0.778385
N	1.216874	1.551733	-0.17131
H	0.460394	0.323185	-1.70309
H	-1.79895	1.10211	-1.5125
H	-0.49902	3.679557	1.751514
H	1.769216	2.826387	1.364556
N	-2.68881	2.823779	0.379418
C	-3.01775	3.749389	1.451092
H	-4.08529	3.961353	1.420584
H	-2.77712	3.32629	2.433709

H	-2.4828	4.696856	1.328696
C	-3.75886	2.172317	-0.37501
H	-4.71792	2.534922	-0.00801
H	-3.68558	2.407225	-1.4418
H	-3.72433	1.084911	-0.24477
C	-2.91268	-1.32004	-1.44037
C	-3.28694	-1.61877	-0.13312
C	-4.60307	-1.52646	0.275869
C	-5.55637	-1.11559	-0.6695
C	-5.1804	-0.80979	-1.97651
C	-3.83935	-0.90942	-2.38023
C	-1.40764	-1.43361	-1.54681
C	-2.03409	-1.94449	0.659613
H	-4.8768	-1.75835	1.301291
H	-6.60076	-1.03297	-0.38175
H	-5.93706	-0.49613	-2.69031
H	-3.52762	-0.67554	-3.39466
O	-0.80201	-1.12633	-2.58871
O	-2.0621	-2.23574	1.849652
C	-0.9195	-1.78999	-0.25388
C	0.493759	-1.86411	0.089277
C	0.925381	-1.49782	1.472461
C	2.034057	-2.10772	2.070662
C	0.290733	-0.46115	2.168456
C	2.512564	-1.67778	3.305604
H	2.514519	-2.93685	1.559545
C	0.768298	-0.02413	3.399294
H	-0.58704	0.003573	1.727979
C	1.887027	-0.62796	3.973673
H	3.371403	-2.17094	3.751896
H	0.258593	0.783233	3.918874
H	2.254606	-0.29609	4.940205
C	1.452753	-2.11479	-0.83088
H	2.508267	-2.05516	-0.57647
H	1.189888	-2.37851	-1.84931
H	2.522858	-0.00299	-2.17932

TS-3

Optimization energy :

E(M06-2X/6-31G(d,p)/IEFPCM(Toluene, SAS)) = -1531.655153 Hartree

Number of imaginary frequency=1 (-483.02)

Single point energy :

E(M06-2X-D3/def2-TZVPP/SMD(Toluene, SAS))= -1532.252233 Hartree

Thermal correction to Gibbs Free Energy= 0.35 kcal/mol

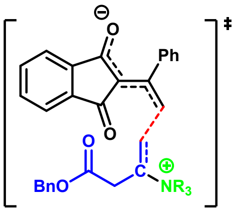

Symbol	X	Y	Z
C	2.220539	2.177201	0.881617
C	0.995852	2.437955	1.489099
C	0.353288	3.795108	1.40298
C	-0.20658	4.142422	0.028105
O	0.479821	4.427462	-0.92063
O	-1.5474	4.126193	0.004364
C	-2.12423	4.354258	-1.28412
H	-1.77173	3.594573	-1.98204
H	-3.20225	4.278951	-1.141
H	-1.85828	5.35121	-1.64353
H	1.127072	4.542901	1.59918
H	2.840696	1.337424	1.180969
H	2.807796	3.065884	0.663347
C	-1.18033	1.367004	1.653369
C	-1.94707	0.236157	1.75344
C	-1.35183	-1.02469	1.995372
C	0.02016	-0.99118	2.358094
C	0.726787	0.172273	2.276641
N	0.171186	1.342075	1.833916
H	-1.60756	2.301106	1.315678
H	-3.00329	0.329946	1.539376
H	0.53628	-1.88363	2.687712
H	1.775736	0.218783	2.534119
N	-2.04446	-2.1811	1.911931
C	-1.35016	-3.45325	2.037222

H	-2.03399	-4.25436	1.757277
H	-0.48874	-3.48952	1.361129
H	-1.01969	-3.63391	3.068393
C	-3.46295	-2.14574	1.607083
H	-3.83177	-3.16654	1.512514
H	-4.0266	-1.64907	2.407164
H	-3.65136	-1.62895	0.659531
C	-1.8828	-0.61259	-1.48968
C	-1.40449	-1.90665	-1.31861
C	-2.2229	-3.00831	-1.48965
C	-3.55384	-2.77892	-1.86417
C	-4.03402	-1.47982	-2.03924
C	-3.19931	-0.3716	-1.84228
C	-0.758	0.35179	-1.22252
C	0.057455	-1.84174	-0.94477
H	-1.8295	-4.01217	-1.3546
H	-4.22226	-3.61989	-2.0249
H	-5.06877	-1.32977	-2.33338
H	-3.55935	0.645113	-1.9715
O	-0.92726	1.571934	-1.18024
O	0.699893	-2.84496	-0.65509
C	0.44251	-0.42882	-0.97796
C	1.747834	0.122768	-0.91204
C	2.958412	-0.73235	-0.71689
C	4.065593	-0.56571	-1.55997
C	3.069456	-1.63885	0.344124
C	5.240186	-1.28119	-1.35399
H	3.991025	0.117171	-2.40124
C	4.24936	-2.34095	0.563316
H	2.211251	-1.80654	0.983035
C	5.339369	-2.16615	-0.28476
H	6.078513	-1.14608	-2.0308
H	4.314013	-3.0359	1.395322
H	6.257173	-2.72175	-0.11756
C	2.005713	1.503868	-0.99295
H	3.019681	1.781351	-1.27223
H	1.228451	2.170484	-1.34499
H	-0.42414	3.917701	2.163628

E$_{DMAP}$

Optimization energy :

E(M06-2X/6-31G(d,p)/IEFPCM(Toluene, SAS)) = -1531.666088 Hartree

Single point energy :

E(M06-2X-D3/def2-TZVPP/SMD(Toluene, SAS))= -1532.259208 Hartree

Thermal correction to Gibbs Free Energy= -4.02 kcal/mol

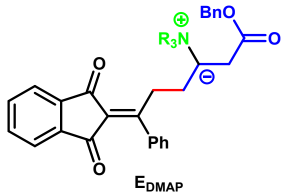

E$_{DMAP}$

Symbol	X	Y	Z
C	1.998653	2.393473	0.684688
C	0.730052	2.513469	1.443154
C	-0.0428	3.79815	1.38249
C	-0.68593	4.09784	0.033598
O	-0.07498	4.460265	-0.94252
O	-2.01844	3.924778	0.049307
C	-2.65984	4.112781	-1.21192
H	-2.2426	3.424393	-1.94758
H	-3.7159	3.902263	-1.04116
H	-2.53003	5.142979	-1.55288
H	0.658275	4.622004	1.554939
H	2.800323	1.827352	1.176931
H	2.390826	3.403125	0.535262
C	-1.31254	1.235176	1.741638
C	-1.93526	0.015528	1.834881
C	-1.2054	-1.18394	1.978616
C	0.169064	-1.00944	2.23717
C	0.755477	0.22602	2.1491
N	0.06045	1.359357	1.769334
H	-1.86789	2.126418	1.488733
H	-3.01303	0.007279	1.727486
H	0.798345	-1.84843	2.509105
H	1.809258	0.370352	2.339539
N	-1.7894	-2.42116	1.89063
C	-0.97438	-3.60789	2.028559
H	-1.57647	-4.48306	1.780463

H	-0.12148	-3.57913	1.33914
H	-0.59612	-3.73726	3.053322
C	-3.21425	-2.52099	1.680045
H	-3.48339	-3.56901	1.543407
H	-3.78656	-2.13	2.533983
H	-3.51992	-1.97763	0.77669
C	-1.79016	-0.79088	-1.50158
C	-1.17369	-2.02734	-1.35574
C	-1.87703	-3.21058	-1.52461
C	-3.22678	-3.11749	-1.87033
C	-3.84455	-1.87136	-2.03019
C	-3.13191	-0.68638	-1.8387
C	-0.80098	0.295493	-1.21359
C	0.270522	-1.8291	-1.00019
H	-1.37948	-4.16828	-1.4025
H	-3.80888	-4.0217	-2.02129
H	-4.89472	-1.83088	-2.30357
H	-3.5984	0.288006	-1.94814
O	-1.09694	1.478497	-1.17556
O	1.037424	-2.74788	-0.78021
C	0.511274	-0.35689	-0.96995
C	1.706087	0.296044	-0.80094
C	3.009739	-0.41638	-0.63787
C	4.082812	-0.08051	-1.47561
C	3.228164	-1.36673	0.36564
C	5.327603	-0.6805	-1.3212
H	3.934632	0.63506	-2.27879
C	4.479107	-1.94694	0.537939
H	2.403391	-1.65923	1.001599
C	5.533471	-1.60812	-0.30512
H	6.137553	-0.41806	-1.99472
H	4.626947	-2.67533	1.329283
H	6.507724	-2.0691	-0.17474
C	1.821393	1.78378	-0.78287
H	2.7372	2.0651	-1.31167
H	0.974072	2.277681	-1.25141
H	-0.80512	3.844518	2.167986

D+10a

Optimization energy :

E(M06-2X/6-31G(d,p)/IEFPCM(Toluene, SAS)) = -2185.537555 Hartree

Single point energy :

E(M06-2X-D3/def2-TZVPP/SMD(Toluene, SAS))= -2186.28917 Hartree

Thermal correction to Gibbs Free Energy= 0.00 kcal/mol

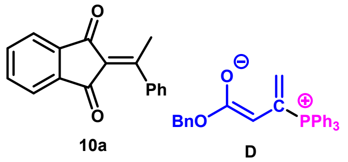

Symbol	X	Y	Z
C	-0.22392	0.769863	-1.90718
C	-0.238	-0.35656	-1.12896
C	0.60004	-1.49546	-1.07644
C	1.672749	-1.82231	-1.96796
O	2.360226	-2.83191	-1.90726
O	1.892918	-0.88768	-2.95001
C	2.913849	-1.24602	-3.87062
H	2.641676	-2.15659	-4.41312
H	2.994944	-0.41003	-4.56904
H	3.864544	-1.40471	-3.35938
H	0.345842	-2.32664	-0.43373
H	-0.96956	1.546806	-1.80578
H	0.515131	0.871943	-2.69153
C	2.721384	-0.8016	1.594429
C	3.652837	-1.14875	0.620974
C	4.46346	-2.26366	0.759019
C	4.336943	-3.01331	1.928294
C	3.429861	-2.64276	2.927381
C	2.604502	-1.52896	2.770323
C	1.935454	0.387053	1.1371
C	3.621294	-0.13796	-0.48251
H	5.15857	-2.53544	-0.0283
H	4.952791	-3.89583	2.071637
H	3.370853	-3.23401	3.836806
H	1.893579	-1.22677	3.533359
O	0.938724	0.811552	1.697541
O	4.383559	-0.12071	-1.42824

C	2.580674	0.866822	-0.11583
C	2.306733	1.994306	-0.82267
C	1.520338	3.13004	-0.26863
C	0.653386	3.874519	-1.08062
C	1.724571	3.557872	1.048591
C	0.001404	4.998448	-0.58891
H	0.473281	3.556126	-2.10292
C	1.077982	4.68651	1.541186
H	2.412324	3.010377	1.683845
C	0.217737	5.414133	0.723493
H	-0.67629	5.551431	-1.2323
H	1.259836	5.005259	2.562929
H	-0.27879	6.301984	1.104591
C	2.957554	2.220595	-2.16372
H	4.015287	2.47277	-2.02233
H	2.927125	1.314192	-2.7687
H	2.486484	3.048943	-2.69589
P	-1.74196	-0.50626	-0.13917
C	-1.63117	-1.0783	1.587006
C	-2.69015	-0.7411	2.442205
C	-0.60424	-1.89917	2.059111
C	-2.73324	-1.24253	3.738487
H	-3.48252	-0.08154	2.100168
C	-0.65835	-2.40094	3.356049
H	0.250593	-2.12909	1.436051
C	-1.7208	-2.08137	4.19471
H	-3.55734	-0.97241	4.390867
H	0.144289	-3.04235	3.707216
H	-1.75641	-2.47755	5.204781
C	-2.61801	1.072248	-0.0305
C	-3.89254	1.232255	-0.57714
C	-1.99627	2.122048	0.659671
C	-4.55774	2.44623	-0.42519
H	-4.36455	0.415388	-1.11508
C	-2.67532	3.326526	0.80824
H	-0.99321	1.994714	1.066924
C	-3.95149	3.487534	0.270432
H	-5.54894	2.574129	-0.8482

H	-2.19871	4.141062	1.342673
H	-4.47472	4.430942	0.393842
C	-2.80838	-1.69599	-1.00867
C	-2.64502	-1.84992	-2.38993
C	-3.79899	-2.41592	-0.33471
C	-3.48153	-2.71301	-3.08914
H	-1.86212	-1.29897	-2.90399
C	-4.63188	-3.27624	-1.0435
H	-3.91523	-2.31755	0.740393
C	-4.47435	-3.42348	-2.4187
H	-3.35077	-2.83646	-4.15928
H	-5.39798	-3.83714	-0.51794
H	-5.12221	-4.09931	-2.96833

TS-4

Optimization energy :

E(M06-2X/6-31G(d,p)/IEFPCM(Toluene, SAS)) = -2185.522335 Hartree

Number of imaginary frequency=1 (-1185.66)

Single point energy :

E(M06-2X-D3/def2-TZVPP/SMD(Toluene, SAS))= -2186.27315 Hartree

Thermal correction to Gibbs Free Energy= 10.05 kcal/mol

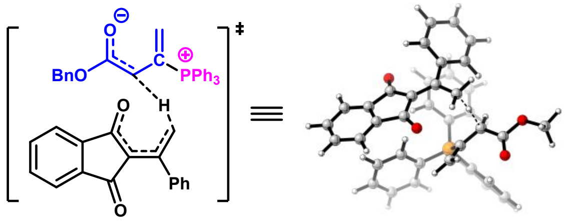

Symbol	X	Y	Z
C	-3.66437	-1.06705	1.457696
C	-3.99344	-0.72217	0.153344
C	-4.9178	-1.45039	-0.57731
C	-5.523	-2.54363	0.05024
C	-5.18877	-2.89359	1.362197
C	-4.24294	-2.15852	2.083993
C	-2.61626	-0.1111	1.96481
C	-3.17641	0.472199	-0.26564
H	-5.15559	-1.17064	-1.59914
H	-6.2607	-3.1332	-0.48607
H	-5.67146	-3.75031	1.823216
H	-3.96532	-2.42139	3.100187
O	-2.08633	-0.23587	3.059648
O	-3.20305	0.901573	-1.41263
C	-2.37966	0.880342	0.902686
C	-1.5428	2.001848	1.051018
C	-1.4607	3.066857	0.011778
C	-2.59608	3.552154	-0.64869
C	-0.22197	3.660531	-0.26767
C	-2.49197	4.584713	-1.5721
H	-3.56614	3.122676	-0.42879
C	-0.11727	4.688832	-1.19866
H	0.669869	3.307899	0.241706
C	-1.25151	5.152988	-1.85728
H	-3.38561	4.951737	-2.06779

H	0.855312	5.124294	-1.40943
H	-1.17369	5.9597	-2.58046
C	-0.67652	2.168775	2.166001
H	-0.8837	1.557198	3.039747
H	-0.39184	3.197811	2.384341
C	0.728482	-1.17271	2.209621
C	1.237678	-0.38166	1.245041
C	1.758628	0.969299	1.417501
C	2.639682	1.218778	2.54886
O	2.841095	0.492156	3.505805
O	3.223098	2.443308	2.446779
C	4.053452	2.794407	3.543292
H	4.888183	2.095063	3.644034
H	4.427493	3.795957	3.327381
H	3.487452	2.796445	4.478607
H	0.490021	1.66531	1.77993
H	0.385751	-2.18424	2.019806
H	0.668464	-0.80855	3.228755
P	1.450596	-1.05035	-0.40801
C	3.182727	-1.53797	-0.58593
C	3.706985	-1.83798	-1.84995
C	3.988089	-1.62487	0.553737
C	5.03111	-2.24355	-1.96843
H	3.084593	-1.74436	-2.73696
C	5.315005	-2.02627	0.421514
H	3.58476	-1.37174	1.532053
C	5.832909	-2.33808	-0.83187
H	5.438576	-2.47859	-2.94627
H	5.944043	-2.08999	1.303189
H	6.868208	-2.65028	-0.92723
C	1.095753	0.212026	-1.6561
C	-0.22749	0.438111	-2.04554
C	2.120358	1.035055	-2.14296
C	-0.52949	1.481105	-2.91481
H	-1.0373	-0.18111	-1.67709
C	1.812864	2.063074	-3.02677
H	3.148796	0.877332	-1.83124
C	0.49148	2.285447	-3.41097

H	-1.56826	1.667648	-3.164
H	2.607076	2.698055	-3.40646
H	0.255057	3.103834	-4.08384
C	0.387391	-2.4876	-0.66918
C	0.873048	-3.66561	-1.24341
C	-0.95479	-2.39769	-0.2798
C	0.01005	-4.73882	-1.44157
H	1.917857	-3.75301	-1.52384
C	-1.81152	-3.47365	-0.4813
H	-1.32239	-1.49187	0.193795
C	-1.32709	-4.64202	-1.06532
H	0.386836	-5.65452	-1.88553
H	-2.85	-3.3949	-0.17274
H	-1.99357	-5.48471	-1.22054
H	2.036744	1.518631	0.521568

D-1+10a-1

Optimization energy :

E(M06-2X/6-31G(d,p)/IEFPCM(Toluene, SAS)) = -2185.545447 Hartree

Single point energy :

E(M06-2X-D3/def2-TZVPP/SMD(Toluene, SAS))= -2186.300093 Hartree

Thermal correction to Gibbs Free Energy= -6.85 kcal/mol

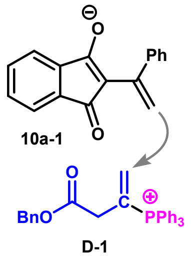

Symbol	X	Y	Z
C	1.164414	-2.60835	-1.66082
C	1.682236	-2.91564	-0.40669
C	1.237005	-4.01036	0.308186
C	0.255725	-4.8242	-0.2821
C	-0.24959	-4.52737	-1.54733
C	0.199177	-3.39993	-2.25409
C	1.826751	-1.33716	-2.14633
C	2.7322	-1.87525	-0.05323
H	1.650794	-4.23249	1.287889
H	-0.10384	-5.70398	0.245071
H	-0.99333	-5.18211	-1.99428
H	-0.18004	-3.15971	-3.24418
O	1.520547	-0.80462	-3.22371
O	3.367912	-1.92791	0.999854
C	2.743416	-0.91187	-1.12984
C	3.615715	0.254314	-1.25868
C	4.330404	0.778328	-0.05442
C	5.689815	1.094936	-0.11974
C	3.653057	1.028998	1.142461
C	6.350578	1.648018	0.974501
H	6.229922	0.888764	-1.03933
C	4.304404	1.585826	2.234839
H	2.596504	0.789256	1.207722
C	5.660801	1.896865	2.156204
H	7.409676	1.879314	0.903467

H	3.752866	1.775767	3.151846
H	6.17492	2.32592	3.011304
C	3.778439	0.924031	-2.41869
H	3.296969	0.601782	-3.333
H	4.394573	1.818245	-2.44852
C	-1.22315	-0.44073	-2.20024
C	-0.9037	0.313039	-1.14364
C	0.132807	1.40876	-1.20738
C	-0.44529	2.678461	-1.80994
O	-1.34527	2.723651	-2.61275
O	0.203995	3.756563	-1.36055
C	-0.21508	4.994379	-1.93317
H	-1.26474	5.186631	-1.69744
H	0.42202	5.757144	-1.48756
H	-0.08939	4.976583	-3.018
H	0.951633	1.083806	-1.86959
H	-2.01697	-1.18097	-2.17331
H	-0.64849	-0.34908	-3.11811
P	-1.90883	0.196007	0.342578
C	-2.75488	1.783627	0.525984
C	-2.59919	2.582471	1.662356
C	-3.52121	2.237952	-0.55637
C	-3.2376	3.81754	1.727016
H	-1.97439	2.252902	2.485835
C	-4.16095	3.468731	-0.47838
H	-3.58442	1.65004	-1.46842
C	-4.02286	4.254666	0.664486
H	-3.11429	4.439374	2.607379
H	-4.75069	3.820804	-1.31798
H	-4.51875	5.218651	0.719908
C	-0.94539	-0.18154	1.813862
C	-1.60026	-0.24501	3.053722
C	0.397941	-0.5369	1.701509
C	-0.88646	-0.62254	4.181409
H	-2.65985	-0.01212	3.132327
C	1.107555	-0.92516	2.83978
H	0.891939	-0.52359	0.73158
C	0.465334	-0.95706	4.072187

H	-1.38526	-0.66855	5.144056
H	2.148987	-1.21325	2.726336
H	1.015965	-1.25899	4.957678
C	-3.09134	-1.15615	0.173724
C	-4.47173	-0.95547	0.139403
C	-2.55211	-2.44857	0.099704
C	-5.31802	-2.05554	0.023542
H	-4.88556	0.046022	0.208605
C	-3.40667	-3.53557	-0.02556
H	-1.47458	-2.609	0.132205
C	-4.78696	-3.33896	-0.06183
H	-6.39248	-1.90663	0.000174
H	-2.98688	-4.53401	-0.09168
H	-5.45157	-4.19214	-0.15427
H	0.581727	1.64442	-0.23776

TS-5

Optimization energy :

E(M06-2X/6-31G(d,p)/IEFPCM(Toluene, SAS)) = -2185.53801 Hartree

Number of imaginary frequency=1 (-232.42)

Single point energy :

E(M06-2X-D3/def2-TZVPP/SMD(Toluene, SAS))= -2186.288668 Hartree

Thermal correction to Gibbs Free Energy= 0.32 kcal/mol

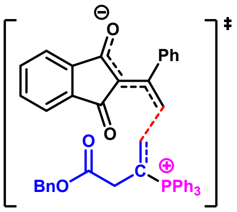

Symbol	X	Y	Z
C	1.071145	3.415432	0.186392
C	1.857272	3.071792	-0.90582
C	2.026291	3.934658	-1.97345
C	1.390461	5.180967	-1.90744
C	0.60248	5.526013	-0.80754
C	0.427131	4.636123	0.258875
C	1.047211	2.256254	1.147227
C	2.383187	1.672315	-0.70078
H	2.63827	3.647044	-2.82349
H	1.51183	5.892097	-2.71973
H	0.120048	6.499216	-0.78325
H	-0.19608	4.880552	1.113998
O	0.365929	2.276812	2.173756
O	2.980222	1.064513	-1.58534
C	1.940768	1.234468	0.616101
C	2.386041	0.11476	1.381553
C	3.630561	-0.66767	1.08452
C	4.525205	-0.88858	2.14362
C	3.91937	-1.26223	-0.15009
C	5.662173	-1.67142	1.980666
H	4.326276	-0.42089	3.103402
C	5.050677	-2.05937	-0.30801
H	3.284668	-1.07843	-1.00401
C	5.926361	-2.26967	0.751923
H	6.342001	-1.81322	2.815689

H	5.249905	-2.50765	-1.27779
H	6.810788	-2.88616	0.620978
C	1.655669	-0.32872	2.466825
H	0.854784	0.27843	2.867384
H	2.075493	-1.09013	3.118477
C	0.341066	-1.70321	1.326048
C	-0.19693	-1.23907	0.144026
C	0.50527	-1.43006	-1.18115
C	1.071529	-2.83003	-1.36447
O	0.709688	-3.82882	-0.78941
O	2.025888	-2.83192	-2.3036
C	2.648351	-4.0953	-2.52927
H	1.916056	-4.8297	-2.87321
H	3.401582	-3.92108	-3.2969
H	3.113926	-4.45714	-1.60936
H	1.317505	-0.70795	-1.33935
H	-0.26493	-1.75981	2.224216
H	1.149881	-2.42492	1.267171
P	-1.79947	-0.53244	0.133117
C	-2.84933	-1.53339	-0.96963
C	-3.96127	-0.98583	-1.61685
C	-2.54178	-2.89047	-1.12371
C	-4.77023	-1.79758	-2.40498
H	-4.18927	0.071153	-1.5131
C	-3.35571	-3.69343	-1.91804
H	-1.66218	-3.30799	-0.63649
C	-4.46818	-3.14928	-2.55459
H	-5.6326	-1.37249	-2.90835
H	-3.11406	-4.7441	-2.04229
H	-5.09902	-3.77842	-3.17498
C	-1.9462	1.169609	-0.48175
C	-2.97184	2.001138	-0.01762
C	-1.10737	1.60793	-1.51198
C	-3.15984	3.255905	-0.58915
H	-3.61952	1.672356	0.790015
C	-1.31249	2.856209	-2.08891
H	-0.28021	0.99299	-1.8555
C	-2.33638	3.679044	-1.62862

H	-3.94864	3.902324	-0.2178
H	-0.64609	3.19998	-2.87279
H	-2.47923	4.660269	-2.07054
C	-2.5228	-0.57208	1.788491
C	-3.46533	-1.54557	2.129496
C	-2.08227	0.358884	2.737922
C	-3.96877	-1.58911	3.426321
H	-3.80385	-2.26488	1.389675
C	-2.59162	0.301358	4.031291
H	-1.34339	1.115596	2.474639
C	-3.53216	-0.66802	4.374398
H	-4.70229	-2.34277	3.693765
H	-2.24885	1.018454	4.770079
H	-3.92672	-0.70554	5.385183
H	-0.18431	-1.29188	-2.02696

E

Optimization energy :

E(M06-2X/6-31G(d,p)/IEFPCM(Toluene, SAS)) = -2185.563737 Hartree

Single point energy :

E(M06-2X-D3/def2-TZVPP/SMD(Toluene, SAS))= -2186.310144 Hartree

Thermal correction to Gibbs Free Energy= -13.16 kcal/mol

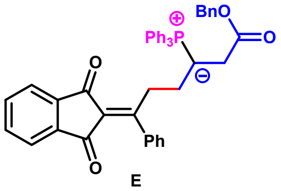

E

Symbol	X	Y	Z
C	2.531702	2.93279	0.08758
C	3.640525	2.353731	-0.5151
C	4.519555	3.104379	-1.28519
C	4.247293	4.461924	-1.43408
C	3.128126	5.046541	-0.82432
C	2.253576	4.28679	-0.05164
C	1.767924	1.901217	0.852552
C	3.689631	0.893305	-0.1924
H	5.382899	2.636487	-1.74771
H	4.90978	5.082167	-2.03016
H	2.94579	6.108374	-0.95939
H	1.377372	4.717338	0.423233
O	0.773581	2.159226	1.503294
O	4.570762	0.148295	-0.56328
C	2.48773	0.598135	0.659166
C	2.180472	-0.5823	1.262911
C	2.952965	-1.83922	1.052486
C	3.344046	-2.60494	2.159455
C	3.223669	-2.33269	-0.22868
C	4.001163	-3.8183	1.990968
H	3.157787	-2.23665	3.163639
C	3.854295	-3.5598	-0.39662
H	2.937222	-1.75774	-1.10074
C	4.248666	-4.30534	0.711002
H	4.312611	-4.38643	2.861918
H	4.04481	-3.92678	-1.40078

H	4.748882	-5.2598	0.577813
C	0.977354	-0.73097	2.142332
H	0.592592	0.239702	2.445688
H	1.264659	-1.29031	3.041153
C	-0.15739	-1.5462	1.444841
C	-0.70326	-1.03825	0.135882
C	-0.06922	-1.49263	-1.15249
C	0.036037	-3.00558	-1.29207
O	-0.64286	-3.83047	-0.73
O	0.991777	-3.34046	-2.18192
C	1.169128	-4.7406	-2.36881
H	0.241452	-5.2061	-2.71099
H	1.945833	-4.84673	-3.12664
H	1.480376	-5.21399	-1.43374
H	0.938708	-1.0923	-1.33417
H	-0.95321	-1.63057	2.193669
H	0.208698	-2.56954	1.289261
P	-1.99917	0.032906	0.053942
C	-3.28551	-0.54764	-1.12921
C	-4.14252	0.310383	-1.82522
C	-3.41116	-1.93244	-1.29242
C	-5.125	-0.21182	-2.66116
H	-4.03923	1.386402	-1.72508
C	-4.39257	-2.4487	-2.13456
H	-2.72408	-2.5924	-0.76496
C	-5.25143	-1.59021	-2.81562
H	-5.78808	0.459437	-3.19834
H	-4.48126	-3.52337	-2.26082
H	-6.01614	-1.99401	-3.47252
C	-1.72967	1.776849	-0.49956
C	-2.57143	2.831062	-0.13278
C	-0.64807	2.032611	-1.34514
C	-2.33707	4.117669	-0.61269
H	-3.40902	2.649926	0.536782
C	-0.42005	3.314436	-1.83579
H	0.034438	1.224281	-1.6008
C	-1.2658	4.358773	-1.46886
H	-2.9914	4.931961	-0.31618

H	0.426065	3.501845	-2.49035
H	-1.08508	5.361736	-1.8443
C	-2.77517	0.216191	1.686007
C	-3.88749	-0.55295	2.032572
C	-2.20173	1.083285	2.623502
C	-4.42486	-0.45802	3.313429
H	-4.33017	-1.22617	1.303749
C	-2.74177	1.16847	3.903291
H	-1.33683	1.682824	2.347932
C	-3.85211	0.400581	4.247516
H	-5.29133	-1.05489	3.580373
H	-2.29428	1.837106	4.63207
H	-4.27229	0.472307	5.246207
H	-0.65989	-1.17369	-2.02621

TS-6

Optimization energy :

E(M06-2X/6-31G(d,p)/IEFPCM(Toluene, SAS)) = -2185.515384 Hartree

Number of imaginary frequency=1 (-311.00)

Single point energy :

E(M06-2X-D3/def2-TZVPP/SMD(Toluene, SAS))= -2186.26504 Hartree

Thermal correction to Gibbs Free Energy= 15.14 kcal/mol

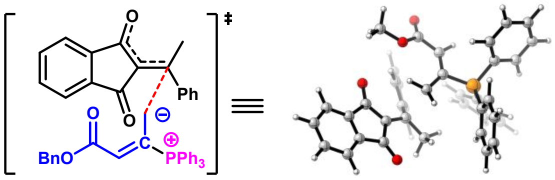

Symbol	X	Y	Z
C	-4.94697	1.417519	0.214377
C	-5.01838	0.240534	-0.51946
C	-6.13011	-0.06645	-1.28472
C	-7.18921	0.845346	-1.28461
C	-7.11845	2.027819	-0.54395
C	-5.98657	2.331075	0.217658
C	-3.6174	1.477296	0.917315
C	-3.73747	-0.52714	-0.3379
H	-6.1668	-0.98753	-1.85856
H	-8.08079	0.636152	-1.86854
H	-7.95561	2.71946	-0.56514
H	-5.91094	3.24849	0.79353
O	-3.27002	2.430023	1.602784
O	-3.4719	-1.5362	-0.98238
C	-2.9244	0.215312	0.623074
C	-1.64727	-0.13037	1.117103
C	-1.25727	-1.55531	1.39559
C	-1.18196	-2.55286	0.417802
C	-0.93162	-1.91006	2.716036
C	-0.75094	-3.83945	0.732622
H	-1.47883	-2.30645	-0.58945
C	-0.54697	-3.207	3.04006
H	-0.99573	-1.17334	3.509632
C	-0.43098	-4.17482	2.044913
H	-0.66131	-4.57409	-0.06215
H	-0.32908	-3.45633	4.075219

H	-0.10592	-5.18127	2.291995
C	-1.00515	0.860839	2.069674
H	-0.99792	1.86598	1.652733
H	-1.58212	0.927755	2.999857
H	0.012211	0.53838	2.313056
C	-0.45549	0.30229	-0.72678
C	0.799514	-0.31814	-0.89832
C	1.157031	-1.53367	-1.46426
C	0.312567	-2.4157	-2.25996
O	0.601853	-3.56076	-2.55081
O	-0.83416	-1.83157	-2.67745
C	-1.7545	-2.69765	-3.33219
H	-2.02538	-3.53406	-2.68237
H	-2.63664	-2.0927	-3.53435
H	-1.32259	-3.08805	-4.25752
H	2.163845	-1.92295	-1.34925
H	-0.48296	1.368755	-0.52856
H	-1.25891	-0.08384	-1.34018
P	2.223407	0.53875	-0.17011
C	3.620911	0.38937	-1.32337
C	4.934704	0.320201	-0.85439
C	3.371131	0.400704	-2.70229
C	5.992554	0.276073	-1.7578
H	5.138503	0.281151	0.21074
C	4.434153	0.362875	-3.5972
H	2.349496	0.408788	-3.07036
C	5.743726	0.302365	-3.12617
H	7.010929	0.213547	-1.38867
H	4.236936	0.366598	-4.6641
H	6.570566	0.26464	-3.82828
C	2.711984	-0.1579	1.423996
C	3.59926	0.541583	2.257032
C	2.236214	-1.41799	1.799295
C	4.016719	-0.02685	3.453058
H	3.953691	1.530016	1.975048
C	2.663713	-1.97652	3.002566
H	1.5338	-1.95839	1.167833
C	3.550539	-1.28853	3.822636

H	4.701043	0.514827	4.097794
H	2.285494	-2.951	3.291701
H	3.877788	-1.7309	4.758355
C	1.909652	2.312887	0.039662
C	1.968444	3.14008	-1.08843
C	1.555686	2.853298	1.279384
C	1.666594	4.491276	-0.97521
H	2.251189	2.732702	-2.05428
C	1.257352	4.208634	1.385271
H	1.503293	2.222704	2.159998
C	1.309994	5.025645	0.260752
H	1.71054	5.126781	-1.85339
H	0.975164	4.620365	2.348408
H	1.071788	6.080977	0.346359

B$_{PPh3}$

Optimization energy :

E(M06-2X/6-31G(d,p)/IEFPCM(Toluene, SAS)) = -2185.56307 Hartree

Single point energy :

E(M06-2X-D3/def2-TZVPP/SMD(Toluene, SAS))= -2186.311107 Hartree

Thermal correction to Gibbs Free Energy= -13.77 kcal/mol

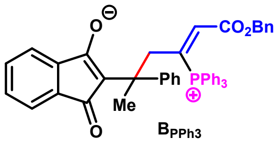

B$_{PPh3}$

Symbol	X	Y	Z
C	-1.00691	-1.58332	0.354863
C	0.302559	-1.32801	-0.3534
C	0.684776	-1.95585	-1.47695
C	-0.03955	-3.01601	-2.23956
O	-0.0171	-3.07305	-3.44407
O	-0.64776	-3.9283	-1.46646
C	-1.33935	-4.94954	-2.18629
H	-0.64294	-5.5151	-2.80932
H	-1.78466	-5.59454	-1.42959
H	-2.11229	-4.51317	-2.82423
H	1.614304	-1.69608	-1.9743
H	-0.93215	-1.27435	1.397617
H	-1.19025	-2.6592	0.345346
C	-1.27638	2.800982	0.059702
C	-2.42901	2.94567	-0.71117
C	-2.87795	4.183431	-1.11628
C	-2.13321	5.306953	-0.72395
C	-0.98758	5.163626	0.052106
C	-0.54102	3.897037	0.46107
C	-1.06168	1.338932	0.302853
C	-2.9862	1.563485	-0.95094
H	-3.77746	4.276699	-1.71757
H	-2.45547	6.299094	-1.02516
H	-0.43038	6.047685	0.348343
H	0.349472	3.777829	1.069805
O	-0.04622	0.933267	1.015194
O	-4.01641	1.324048	-1.55191

C	-2.05332	0.612987	-0.30061
C	-2.26061	-0.87735	-0.26195
C	-3.44022	-1.27104	0.646639
C	-3.88028	-2.60053	0.675925
C	-4.06929	-0.35583	1.491701
C	-4.91859	-2.99847	1.509918
H	-3.40161	-3.34237	0.040778
C	-5.11193	-0.75026	2.327657
H	-3.74176	0.678594	1.499937
C	-5.54326	-2.07124	2.340002
H	-5.24072	-4.03569	1.511015
H	-5.58821	-0.01566	2.970235
H	-6.35694	-2.37797	2.99007
C	-2.5429	-1.3611	-1.6973
H	-3.46476	-0.89897	-2.05463
H	-1.73793	-1.06251	-2.37637
H	-2.65722	-2.44698	-1.74877
P	1.509857	-0.05931	0.306387
C	1.757655	1.456957	-0.68097
C	2.562274	2.485253	-0.18605
C	1.137122	1.590917	-1.9242
C	2.738801	3.64594	-0.93279
H	3.038194	2.389165	0.786294
C	1.310711	2.76113	-2.65997
H	0.50675	0.796519	-2.31361
C	2.109854	3.787487	-2.16748
H	3.362473	4.444699	-0.54323
H	0.813704	2.867545	-3.61882
H	2.240512	4.698424	-2.74283
C	2.018107	-0.03459	2.083059
C	3.375657	0.127643	2.390538
C	1.102205	-0.17936	3.131461
C	3.80288	0.161076	3.714255
H	4.110268	0.226618	1.598382
C	1.541412	-0.19205	4.451251
H	0.044688	-0.25361	2.919672
C	2.888931	-0.01411	4.747758
H	4.855658	0.309544	3.93202

H	0.819145	-0.32442	5.250449
H	3.224821	-0.00827	5.780107
C	3.06817	-0.9757	-0.27214
C	3.338899	-2.23276	0.291733
C	3.962117	-0.47551	-1.22066
C	4.460624	-2.96135	-0.07596
H	2.658855	-2.64619	1.034511
C	5.093263	-1.20716	-1.59443
H	3.788021	0.489874	-1.68497
C	5.34557	-2.44697	-1.02497
H	4.648219	-3.93079	0.375615
H	5.773327	-0.79858	-2.3357
H	6.2242	-3.01445	-1.31578
